# Genome-wide study of pineapple (*Ananas comosus* L.) bHLH transcription factors indicates that cryptochrome-interacting bHLH2 (*Ac*CIB2) participates in flowering time regulation and abiotic stress response

**DOI:** 10.1186/s12864-020-07152-2

**Published:** 2020-10-22

**Authors:** Mohammad Aslam, Bello Hassan Jakada, Beenish Fakher, Joseph G. Greaves, Xiaoping Niu, Zhenxia Su, Yan Cheng, Shijiang Cao, Xiaomei Wang, Yuan Qin

**Affiliations:** 1 College of Life Science, Fujian Provincial Key Laboratory of Haixia Applied Plant Systems Biology, Key Laboratory of Genetics, Breeding and Multiple Utilization of Crops, Ministry of Education, State Key Laboratory of Ecological Pest Control for Fujian and Taiwan Crops, Fujian Agriculture and Forestry University, Fuzhou, 350002 Fujian China; 2 Guangxi Key Lab of Sugarcane Biology, State Key Laboratory for Conservation and Utilization of Subtropical Agro-Bioresources, College of Agriculture, Guangxi University, Nanning, 530004 Guangxi China; 3 College of Agriculture, Fujian Agriculture and Forestry University, Fuzhou, 350002 Fujian China; 4 College of Plant Protection, Fujian Agriculture and Forestry University, Fuzhou, 350002 Fujian China; 5 College of Forestry, Fujian Agriculture and Forestry University, Fuzhou, 350002 Fujian China; 6 Horticulture Research Institute, Guangxi Academy of Agricultural Sciences, Nanning Investigation Station of South Subtropical Fruit Trees, Ministry of Agriculture, Nanning, 530007 China

**Keywords:** bHLH, CIB2, Flowering time, Abiotic stress, Pineapple

## Abstract

**Background:**

Transcription factors (TFs) are essential regulators of growth and development in eukaryotes. Basic-helix-loop-helix (bHLHs) is one of the most significant TFs families involved in several critical regulatory functions. Cryptochrome-interacting bHLH (CIB) and cryptochromes form an extensive regulatory network to mediate a plethora of pathways. Although bHLHs regulate critical biological processes in plants, the information about pineapple bHLHs remains unexplored.

**Results:**

Here, we identified a total of 121 bHLH proteins in the pineapple genome. The identified genes were renamed based on the ascending order of their gene ID and classified into 18 subgroups by phylogenetic analysis. We found that bHLH genes are expressed in different organs and stages of pineapple development. Furthermore, by the ectopic expression of *Ac*CIB2 in *Arabidopsis* and complementation of *Atcib2* mutant, we verified the involvement of *Ac*CIB2 in photomorphogenesis and abiotic stress response.

**Conclusions:**

Our findings revealed that *Ac*CIB2 plays an essential role in flowering time regulation and abiotic stress response. The present study provides additional insights into the current knowledge of bHLH genes and suggests their potential role in various biological processes during pineapple development.

**Supplementary information:**

**Supplementary information** accompanies this paper at 10.1186/s12864-020-07152-2.

## Background

Transcription factors (TFs) are vital proteins that participate in crucial physiological functions in different tissues during different stages of development and physiological responses. TFs could repress or activate the expression of their target genes, resulting in the regulation of the development and physiological response. TFs could repress or activate the target gene expression resulting in the regulation of  development and response [1, 2]. Basic-helix-loop-helix (bHLH) are among the most significant and functionally important class of TF family, which is widely distributed among eukaryotes [3, 4]. bHLH proteins are characterized by a bHLH domain of approximately 60 amino acid sequences with two conserved regions, the basic region and a helix-loop-helix (HLH) region [5]. The basic domain comprises 10 to 15 amino acids, while HLH contains approximately 40 amino acids. The basic region is found at the N-terminus of the domain and modulates DNA binding, whereas the HLH region of the domain facilitates dimerization through protein-protein interaction [6]. Generally, bHLH TFs regulate their target after forming homo or heterodimers by interacting with bHLHs and other regulatory proteins [7, 8]. In plants, bHLHs play crucial roles in gene expression during regulatory and developmental processes, including transcriptional regulation, chromosome segregation, general transcriptional enhancement, hormonal signaling, wounding, response to environmental cues, metabolism regulation, flower and fruit development [3, 9–12].

In angiosperms, a successful transition from the vegetative phase to the reproductive stage, followed by fertilization, is essential for seed formation. Plant starts to flower in response to a plethora of environmental signals, including photoperiod, which ensures their reproductive success [13]. Plants encode numerous photo-receptors that participate in light signaling and regulate many aspects of growth and development [14]. Several photoreceptors have been reported in plants, including cryptochromes, phytochromes, phototropins, UV Resistance locus 8 (UVR8), and Zeitlupe family members (ZTL, FKF1, and LKP2) [15–17]. CRYs are photolyase-associated blue-light receptors, and they interact with different proteins in the presence of blue-light to mediate a plethora of functions, such as inhibition of hypocotyl elongation and flowering initiation [9, 18–20]. In *Arabidopsis*, three cryptochromes are encoded: cryptochrome 1 (CRY1), cryptochrome 2 (CRY2), and cryptochrome 3 (CRY3) [14, 17, 21, 22]. CRY1 participates in blue light-dependent de-etiolation responses and inhibition of hypocotyl elongation. It also acts redundantly with CRY2 and is partly involved in floral initiation [9, 14]. However, the primary function of CRY2 is the regulation of flowering in response to blue light [18, 23, 24]. CRY3 is found in chloroplasts and mitochondrion and reported to repair UV-induced single-stranded DNA damage [14].

In response to blue-light, cryptochromes interact with different proteins to regulate photomorphogenesis. Several proteins are known to interact with cryptochrome, including CRY2-interacting bHLH proteins (CIBs) [16]. CIBs belong to BEE/CIB subfamily of bHLH and interact with cryptochrome to regulate floral initiation by activating FLOWERING LOCUS T (FT) [9]. CIBs act redundantly in the CRY-CIB pathway to promote flower induction [9]. CIB1 was the first among CIBs to be identified in plants that positively regulates floral initiation [25]. Similarly, CIB2 and other CIBs also regulate flowering individually or after dimerization [9].

Comprehensive characterization and functional analysis of bHLH TFs have been performed in several important crop plants, including Chinese cabbage and Brassica [5, 7, 11]. However, no study of this essential TF family is reported in pineapple, an economically crucial perennial fruit crop belonging to *Bromeliaceae*. Similar to other plants, pineapple also encodes several bHLHs, including CIBs. Here, by performing a genome-wide study, we identified 121 bHLH proteins and characterized them comprehensively. Further, we also described *Ac*CIB2 (*Ac*bHLH8) functions by ectopically expressing it and complementing the *Arabidopsis cib2* mutant. Here we show that *Ac*CIB2 is involved in flowering time regulation and also participates in abiotic stress response.

## Results

### Identification and characterization of pineapple bHLH genes

We identified 121 *Ac*bHLH proteins in pineapple and named them based on the ascending order of their gene ID. The bHLH genes of pineapple showed high similarity to those in *Arabidopsis*. We further characterized the pineapple bHLH proteins based on their molecular weight, isoelectric point, amino acid, and open reading frame (ORF) length, respectively (Additional Table S1). The molecular weight of *Ac*bHLH proteins ranged from 1.04 kDa to 345.97 kDa. *Ac*bHLH17 (Aco001282) have a higher molecular weight of 345.97 kDa, followed by *Ac*bHLH99 (Aco016776) with 92.57 kDa. *Ac*bHLH15 (Aco001136) has the lowest molecular weight of 1.04 kDa among the pineapple bHLH proteins. The pineapple bHLH proteins also have different isoelectric point values, ranging from *Ac*bHLH30 (Aco002151) with the highest of 10.76, and *Ac*bHLH53 recording the smallest value of 4.73. Consistently, the pineapple bHLHs have different ORF size, where *Ac*bHLH17 has the most extended ORF sequence, while *Ac*bHLH91 (Aco004138) has the shortest ORF (Additional Table S1).

The exon-intron analysis suggests that most of the *Ac*bHLH possess introns, and with forty-nine introns, *Ac*bHLH17 had the maximum number of introns. However, *Ac*bHLH18, *Ac*bHLH45, *Ac*bHLH64, *Ac*bHLH70, *Ac*bHLH76 and *Ac*bHLH83 were intronless. Besides, twenty-eight *Ac*bHLH genes did not have the UTRs, and seven *Ac*bHLH genes only had 5′ UTR, and sixteen *Ac*bHLH genes had 3′ UTR only (Additional Figure S2).

### Phylogenetic analysis, chromosome location, and motif analysis

**Fig. 1:**
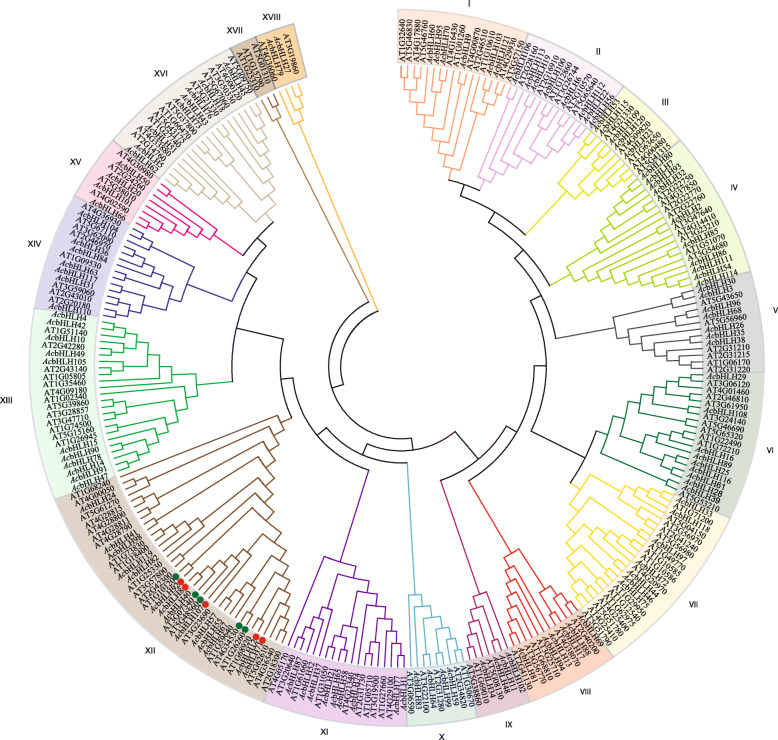
Phylogenetic tree showing the relationship between bHLH genes of pineapple and *Arabidopsis*. Different colors indicate different groups. Prefix ‘Ac’ indicates *Ananas comosus* and ‘AT’ refers to *Arabidopsis thaliana*. Red circles represent the pineapple CIB genes and the green represents *Arabidopsis* CIB genes

The phylogenetic tree divided pineapple bHLH proteins into eighteen groups (from I to XVIII) with their corresponding *Arabidopsis* homologs (Fig. 1). Interestingly, all the *Ac*CIBs were in the group XII with *Arabidopsis* CIBs (Fig. 1). The pineapple CIB genes grouped with their corresponding *Arabidopsis* homolog suggest that they may have a similar biological function in photomorphogenesis and developmental responses.

**Fig. 2:**
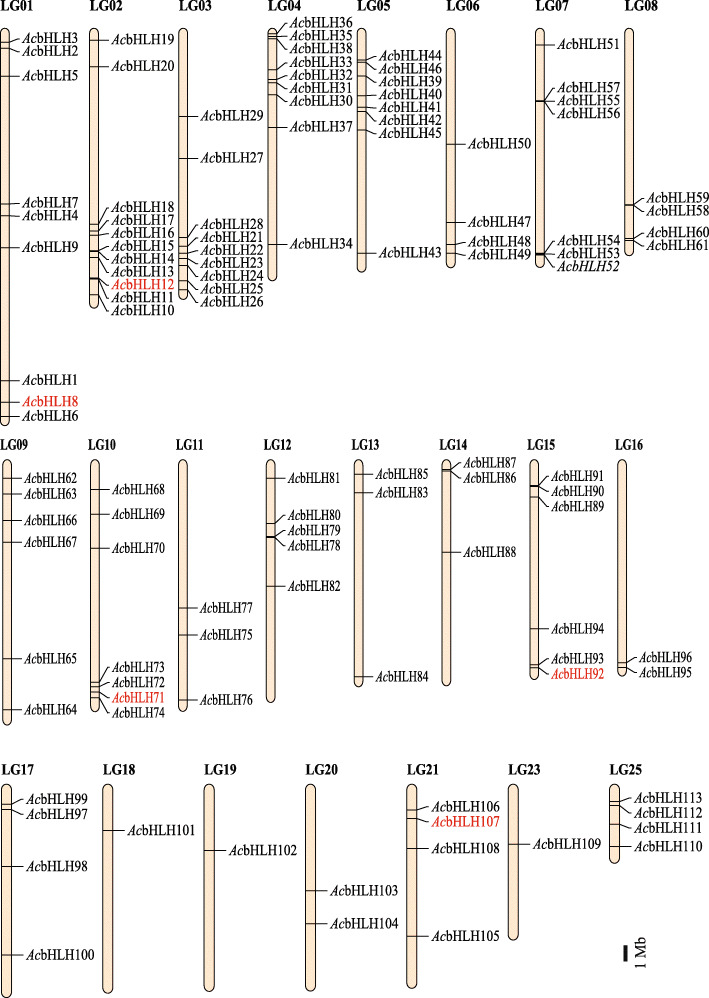
Chromosomal locations of pineapple bHLH genes. The bHLH genes of pineapple were mapped to different chromosomes using MapChart. Each *Ac*bHLH is noted on the right side of its respective chromosome. Gene IDs in red represent pineapple CIB genes. The scale is in megabases (Mb)

We then studied the distribution of *Ac*bHLHs on pineapple chromosomes and found that pineapple bHLH genes are distributed unevenly on 23 linkage groups (LG). Only two pineapple linkage groups, LG 22 and LG 24, do not possess bHLH genes. Few linkage groups have a higher density of bHLH genes (up to 11 genes), whereas few have only one, and all the *Ac*bHLHs are distributed on different LGs (Fig. 2). These findings indicate that there is no direct correlation between bHLH gene distribution and linkage groups length.

**Fig. 3:**
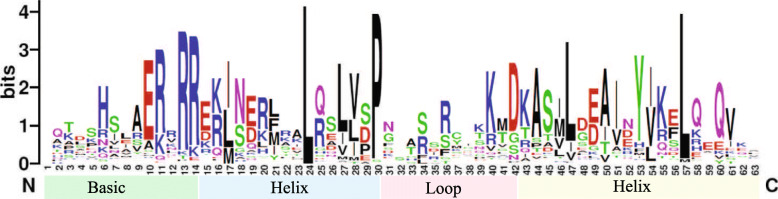
Sequence characteristics of the bHLH domains. Multiple sequence alignments were conducted with the bHLH domains of all pineapple bHLH candidate and results were visualized using Weblogo online software

To further characterize the *Ac*bHLH proteins, we retrieved the amino acid sequences from the bHLH domain region and aligned them (Additional Figure S3). The pineapple bHLH domain analysis indicates that the average length of the *Ac*bHLH domain was approximately 50 aa, which ranged from 34 to 56 aa (Fig. 3, Additional Table S4). Further, the study of conserved motif distribution of *Ac*bHLH superfamily using the MEME program resulted in the identification of ten different motifs distributed among *Ac*bHLH proteins (Additional Figure S5). The numbers of these motifs in bHLHs proteins were different, which could be responsible for the functional diversity of *Ac*bHLH proteins. The number of motifs on each *Ac*bHLH ranged from 1 to 9. For example, *Ac*bHLH34, *Ac*bHLH46, *Ac*bHLH47 and *Ac*bHLH90 had only one motif whereas, *Ac*bHLH108 and *Ac*bHLH60 had a maximum of 8 and 9 motifs, respectively (Additional Figure S5). Moreover, the Pfam domain search indicated that some on the *Ac*bHLH protein possessed other domains in addition to the bHLH domain. For example, eight *Ac*bHLHs (*Ac*bHLH9, *Ac*bHLH20, *Ac*bHLH23, *Ac*bHLH60, *Ac*bHLH70, *Ac*bHLH99, *Ac*bHLH109, and *Ac*bHLH120) have extra bHLH-MYC_N domain downstream in addition to bHLH domain. The structural information further suggests that pineapple *Ac*bHLHs genes have a close similarity with other reported bHLH proteins, and may also be performing a similar physiological function.

### Expression profile of pineapple bHLH genes in pineapple

**Fig. 4:**
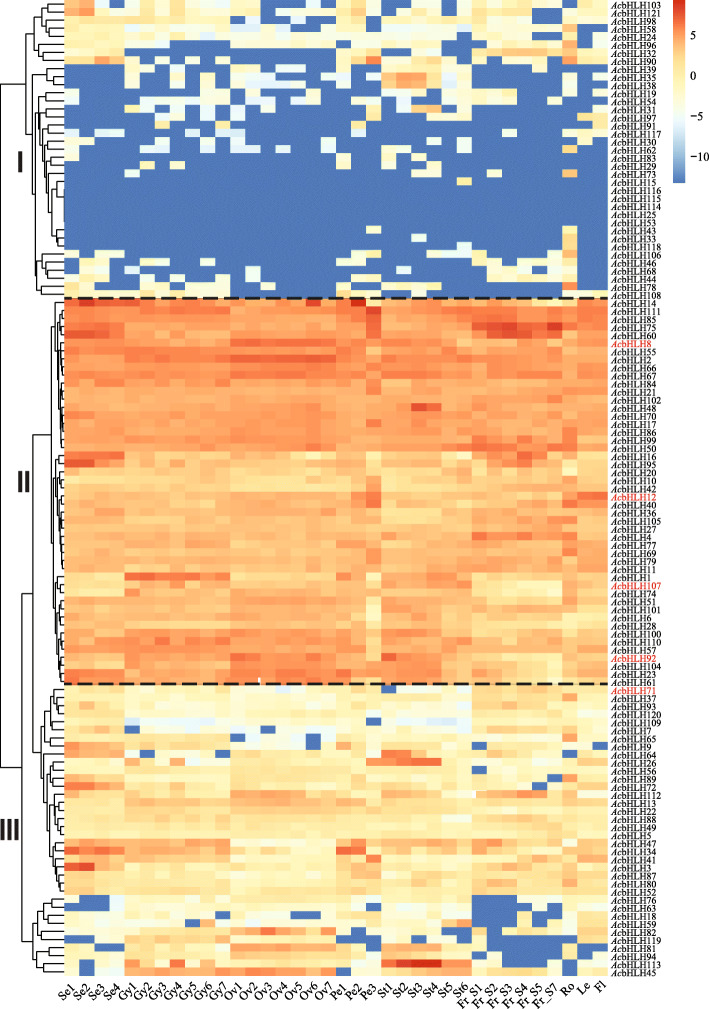
Expression profiles of the pineapple bHLH genes. Hierarchical clustering of expression profiles of bHLHs in different organs and developmental stages. Red color represents a high level of transcript abundance, and blue color represents low transcript abundance. The right side of the figure shows the scale. Different groups i.e. group I, group II and group III represent low expressed, highly expressed, and moderately expressed bHLH genes, respectively. Gene IDs in red represent pineapple CIB genes. Details of the samples are mentioned at the bottom of each lane: sepal Se1–Se4, gynoecium Gy1- Gy7, ovule Ov1–Ov7, petal Pe1–Pe3, stamen St1–St6, fruit ‘Fr_S1–Fr_S7’, root ‘Ro’, leaf ‘Le’, and flower ‘Fl’ where ‘S’ is the abbreviation for ‘stage

We performed the expression profile analysis of pineapple bHLH genes using RNA-seq from various developmental stages of different organs to understand their possible functions in pineapple. The altered expression patterns of the majority of *Ac*bHLH in the selected samples suggest that the pineapple bHLH genes play different roles in a specific organ or developmental stage. The expression profile of *Ac*bHLH genes mainly clustered into three different groups according to their expression pattern. Low expressed bHLH genes were gathered together in group I, highly expressed genes formed group II and the moderately expressed genes formed group III (Fig. 4).

Overall, the majority of bHLH showed stage-specific and organ-specific expression, suggesting the specificity of bHLH proteins during pineapple development. Hierarchical clustering into three distinct groups indicates a correlation between biological function and the expression pattern at a particular stage and organ development. Depending on the developmental requirements, the expression of the *Ac*bHLHs was a stage and/or organ-specific. For example, *Ac*bHLH113, a homolog of ABORTED MICROSPORES (AMS), had relatively high expression levels in the different stages of stamen development, indicating that it might be playing a crucial role in pineapple anther development (Fig. 4). Most of the pineapple CIB genes except *Ac*CIB1 (*Ac*bHLH71) were in group II, displaying a high expression level in the stages of flower and fruit development, suggesting that the pineapple *Ac*CIB1 may not be the primary regulator of flowering. The remaining CIBs, i.e. CIB2 (*Ac*bHLH8), CIB3 (*Ac*bHLH107), CIB4 (*Ac*bHLH92) and CIB5 (*Ac*bHLH12) are in group II with highly expressed bHLH genes. Interestingly, *Ac*CIB2 showed a high expression in all the stages of ovule, stamen development and had a relatively higher expression in flower, suggesting that *Ac*CIB2 may be playing a crucial role in flower development (Fig. 4). These results indicate that *Ac*bHLHs genes are expressed at different stages of pineapple development and are essential for growth and development.

### *Ac*CIB2 is a nuclear protein involved in photomorphogenesis

**Fig. 5:**
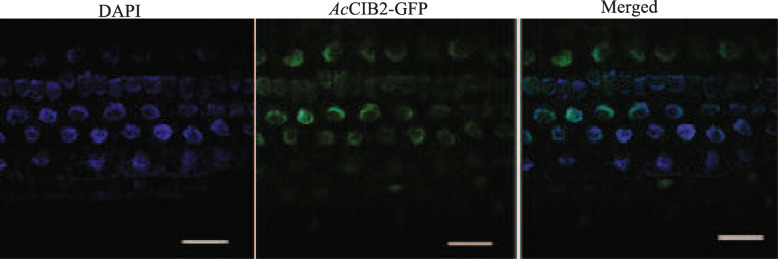
*Ac*CIB2-GFP localizes to the nucleus. *Ac*CIB2-GFP localization in the nucleus of seven-day-old roots of transgenic *Arabidopsis* plants. GFP fluorescence is represented in green and DAPI in blue channel, Scale bars = 20 μm

**Fig. 6:**
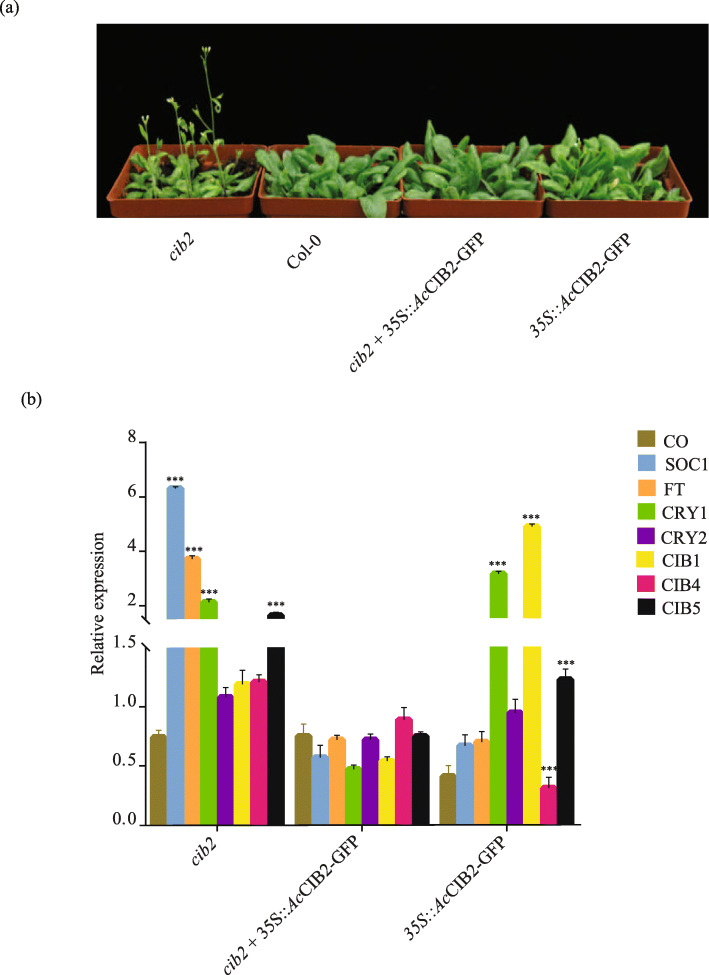
*Ac*CIB2 regulates the photomorphogenesis. **a** Photograph showing the early phenotype of *Arabidopsis cib2* mutant and complementation of early flowering phenotype of by *Ac*CIB2-GFP. Plants were grown on the media plates for ten days, followed by transferring to soil in plastic pots and kept in a walk-in chamber. Plants were then photographed after 20 days of the transfer. **b** Relative expression of critical flowering genes (*CO*, *FT* and *SOC1*) and *CRY-CIB* genes (*CRY1*, *CRY2*, *CIB1*, *CIB4* and *CIB5*) in Col-0, *cib2* mutant, complemented (*cib2* + 35S::*Ac*CIB2-GFP) and in *Ac*CIB2 overexpressing (35S::*Ac*CIB2-GFP) lines. Gene expression is represented in fold change of expression against *Arabidopsis* ef1α calculated by 2^−ΔΔ*C*^_*T*_. Vertical bars represent the mean ± SE of three biological replicate assays. Asterisks denote the statistical significance between control and treatment as judged by the Student’s t-test (*** *P* < 0.001)

To investigate the possible role of *Ac*CIB2, we generated transgenic plants of *Arabidopsis* that were ectopically expressing *Ac*CIB2. The observation of 7-day old roots of transgenic plants under a confocal microscope showed that *Ac*CIB2-GFP is a nuclear protein, and it localizes in the nucleus (Fig. 5), which is in agreement with the previous findings [23]. Generally, Col-0 plants start flowering in 23 to 26 days after transferring to the soil in a walk-in growth chamber, but the *Arabidopsis cib2* mutant begun to produce the flower in 14 to 16 days (Fig. 6a). We found that the *Ac*CIB2-GFP could complement the early flowering phenotype of *Atcib2*, and complemented plants produce flower between 23 to 25 days after transfer to soil. While the plants ectopically expressing *Ac*CIB2 do not show any significant differences in the flowering time and produced flower between 22 to 24 days, similar to wild-type plants (Fig. 6a).

To explore the reason behind the early flowering phenotype of *cib2*, we examined the expression of major flowering related genes (*CO*, *FT,* and *SOC1*) and *CRY-CIB* genes (*CRY1*, *CRY2*, *CIB1*, *CIB4,* and *CIB5*). We found that the expression level of *SOC1* and *FT* was significantly higher in the *cib2* mutant, whereas the expression of *CO* was reduced compared to Col-0 plants (Fig. 6b). The complemented plants of *cib2* mutant by *AcCIB*2 did not show any significant change in the expression of selected genes compared to wild type, suggesting that *Ac*CIB2 may have a conserved role in plants. However, the ectopic expression of the *AcCIB2* changed the expression of *CRY-CIB* genes, and the expression of *CRY1*, *CIB1* and *CIB5* were significantly altered, suggesting that the *CRY-CIB* genes may be working redundantly in the pathways (Fig. 6b). Taken together, these results indicate that the *AcCIB2* is involved in photomorphogenesis and may have a conserved function in plants.

### Ectopic expression of *Ac*CIB2 enhances abiotic stress resistance

**Fig. 7:**
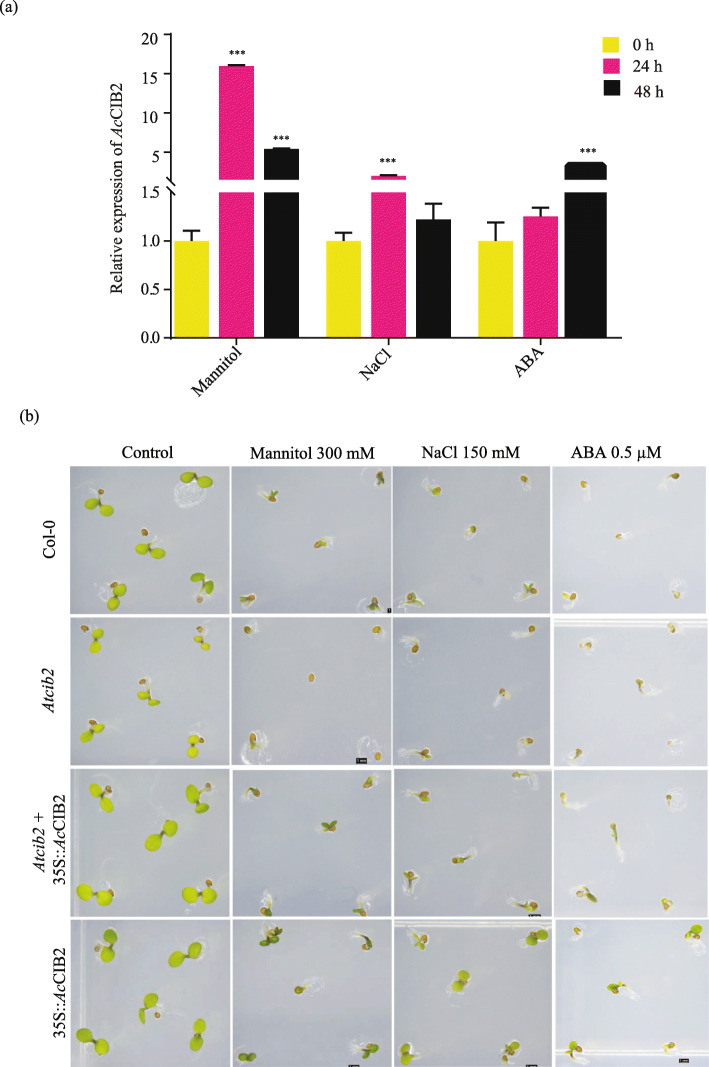
**a** Relative expression of *Ac*CIB2 in pineapple plants at different time points after osmotic (300 mM Mannitol), salt 150 (mM NaCl) and phytohormone ABA (0.5 μM) treatment. *Ac*CIB2 expression is represented in fold change of expression against pineapple ef1α calculated by 2^−ΔΔ*C*^_*T*_. Vertical bars represent the mean ± SE of three biological replicate assays. Asterisks denote the statistical significance between control and treatment as judged by the Student’s t-test (*** *P* < 0.001). **b** Overexpression of *Ac*CIB2 results in resistance to abiotic stress. The phenotype of 3 days old *cib2* mutant, complemented (*cib2* + 35S::*Ac*CIB2-GFP) and in *Ac*CIB2 overexpressing (35S::*Ac*CIB2-GFP) lines germinated under osmotic stress (300 mM Mannitol), salinity stress (150 mM NaCl), and ABA 0.5 μM

To better understand the role of *Ac*CIB2 in response to various abiotic stress, we checked the expression of *Ac*CIB2 at different time points under osmotic (350 mM Mannitol) stress and salt (150 mM NaCl) stress in pineapple plants. The quantitative RT-PCR shows that both the osmotic and salt stress increased the expression of *Ac*CIB2, suggesting its potential role during abiotic stress in pineapple (Fig. 7a). To further validate the involvement of *Ac*CIB2 in abiotic stress response, we grew different *Ac*CIB2 transgenic lines, including *Atcib2* and wild-type *Arabidopsis* plants on a media supplemented with Mannitol 300 mM, 150 mM NaCl and 0.5 μM abscisic acid (ABA). In the germination assay, we found that the *Atcib2* mutant displays susceptibility to salinity and osmotic stress compared to wild-type plants. However, the transgenic plants expressing *Ac*CIB2 resulted in better performance in terms of germination and growth phenotype during salinity, osmotic stress and ABA treatment (Fig. 7b). Overall, these findings approve the role of cryptochromes in abiotic stress response.

## Discussion

Transcription factors regulate the expression of downstream target genes, resulting in control of diverse biological processes. A group of transcription factors contains a highly conserved amino acid motif known as basic helix-loop-helix (bHLH) domain. These bHLH proteins perform a myriad of regulatory function in eukaryotic lineages and have been studied in several plants [6, 11]. The bHLH transcription factors have been previously studied in several plant species, including maize and potato [3, 4, 11, 26–28]. However, this group of crucial transcription factor family is still unexplored in pineapple. The availability of the sequenced genome of pineapple serves as a great genetic resource for studying the gene families [29]. Here, we identified 121 bHLH genes that form a large family in pineapple. Generally, angiosperms have more bHLH sequences in their genome and form a big family; for example, in *Arabidopsis,* approximately 170 bHLH proteins are found [3]. Similarly, pineapple also possesses a large number of bHLH proteins suggesting their dispensable role in the pineapple evolution and development.

Previous studies suggest that the two helices of the bHLH domain fulfill the DNA binding prerequisite by forming the homo or heterodimers between the bHLH proteins. In contrast, the basic region of most bHLH proteins interacts with the DNA sequences such as E-boxes and G-boxes [6]. Besides, approximately 77% of *Ac*bHLH possessed the conserved glutamic acid (E) at the 9th position in their basic region of the domain (Additional Figure S3). This glutamic acid (E) directly binds to CA nucleotide of the hexanucleotide sequence of the E-box and/or G-box [6, 30]. Pineapple bHLH proteins also share similar conserved domains and amino acid sequences with the *Arabidopsis* proteins in the same cluster.

The exon and intron structure of genes is an important feature to study evolutionary and functional divergence within the same or closely related gene families [3]. We found different exon-intron structures in *Ac*bHLH superfamily, some of the genes have no introns in their structure, and some are intron rich, in contrast, some have few numbers of introns (Additional Figure S2, Additional Table S1). Consistently, a phylogenetic tree reveals the functional relationship of proteins within a group and serves as an excellent tool to study evolution [27, 31]. Phylogenetic analysis classified *Ac*bHLHs into 18 subgroups (Fig. 1), the pineapple bHLH also formed a group with their *Arabidopsis* homologs. *Ac*CIB2 falls into group XII with pineapple CIBs, *Arabidopsis* CIBs, and phytochrome interacting factors (PIFs), suggesting that they might have a similar function and are closely related via a common ancestor. Members of group XII play a significant role in photomorphogenesis [27], indicating the functional and conserved evolutionary relationship of bHLH proteins between pineapple and *Arabidopsis*. Interestingly, bHLH transcription factors and cryptochromes are also encoded by *E. coli*, liverworts, and ferns, indicating their conserved nature [32, 33].

The gene expression patterns during different growth stages and conditions could also be an indicator of gene function [11].

One of the essential class of photo-receptors involved in flowering initiation is cryptochromes (CRYs) and their interacting proteins; cryptochrome-interacting bHLH proteins (CIBs) [18]. Most of the photoreceptors signaling mechanisms have been reported in *Arabidopsis* [15], and recently in tomato [17]. CRY2-CIB network in plants decodes an excellent pathway for modulating light signal during photomorphogenesis. In *Arabidopsis*, CRY2 interacts with CIB1, CIB2, CIB4, and CIB5 to mediate growth and development, especially flowering time regulation and response to environmental cues. The finding that *AcCIB2* expresses in the majority of developmental stages and could be involved in flower development led us further to study the function of *Ac*CIB2. Previous studies also suggest that CIBs interact with CRY2 and activate transcription of flowering related genes in response to blue light [9, 18, 25]. In agreement with these findings, we found that the null mutation of CIB2 results in an early flowering phenotype. To check whether pineapple CIB2 and *Arabidopsis* CIB2 have a similar function, we complemented the *Atcib2* mutant with *Ac*CIB2. Consistent with our hypothesis, *Ac*CIB2 could complement *Atcib2* (Fig. 6a), suggesting that *Ac*CIB2 has a similar biological function with *At*CIB2.

Generally, six different genetic pathways control the flowering, and they finally come together downstream at floral integrators FT and SOC1. The expression of FT and SOC1 induces the expression of floral identity genes resulting in flower formation [34]. Previous studies show that CIBs act redundantly to regulate flowering by promoting the transcription of some flowering genes, notably *FT* and *SOCI* [9, 18, 35]. We investigated the transcript level of *FT*, *SOC1* and other *CIBs* in the *Atcib2* and the transgenic *Arabidopsis* plants with pineapple CIB2. Consistently, the transcript level of *FT* and *SOC1* genes changed significantly in the mutant. The quantitative RT-PCR data indicate that the mutation in CIB2 triggers the *FT* transcription resulting in early flowering (Fig. 6b). Besides, the transcript levels of *CIBs* (*CIB1*, *CIB4* and *CIB5*) were also altered, indicating that CIBs act redundantly during photomorphogenesis in plants.

Increasing evidence indicates that cryptochromes are also involved in abiotic stress response through biosynthesis of ROS [36]. Several findings suggest that in addition to their established role in photomorphogenesis, cryptochromes also react to numerous abiotic stress responses [36, 37]. This indicates that CIBs might also be involved in abiotic stress response as they regulate cryptochromes. In agreement, the quantitative RT-PCR result showed a significant change of *Ac*CIB2 transcript in pineapple plants under osmotic and salt stress, validating the idea that CIB2 plays a role in abiotic stress response (Fig. 7a). Further, transgenic *Arabidopsis* plants also showed resistance to salinity and osmotic stress (Fig. 7b). The role of CRYs in stomatal development, opening and closure during stress conditions has been well documented [38–40]. We also found that the transgenic plant performed better under ABA treatment, supporting the notion that CIB2 could be a regulator of abiotic stress response in plants through CRY-CIB pathway.

Taken together, the present study provides a platform to study the pineapple *Ac*bHLH genes. Future studies with the specific *Ac*bHLHs that are involved in the particular pathway will further clarify how *Ac*bHLHs regulate the response to biotic and abiotic stresses in pineapple.

## Conclusion

In this study, a comprehensive investigation of bHLH genes was performed, and 121 *Ac*bHLH genes were identified in the pineapple genome. Pineapple bHLH genes were further classified into 18 subfamilies. The *Ac*bHLHs expression profiles suggest their diverse expression at different developmental and in different organs. Besides, the functional characterization of *Ac*bHLH8 (*Ac*CIB2) shows the conserved functional role of bHLH genes in photomorphogenesis and response abiotic stress. Overall, this study provides important information about the potential functions of the *Ac*bHLHs, especially the *Ac*CIB2 role in flowering, which is an essential trait for crop breeding.

## Methods

### Plant materials and growth conditions

#### Pineapple growth and treatments

Two-month-old tissue culture raised MD2 variety, a hybrid pineapple (*Ananas comosus*) variety from Pineapple Research Institute Hawaii, was used for the experiments. The pineapple breeding group provided the starting material for tissue culture in Fujian Agriculture and Forestry University. The plants were reared in pots containing soil supplements (peat moss: perlite = 2:1 *v*/*v*) kept in a chamber at 30 °C, 16 h light/8 h dark photoperiod under the light intensity of 60–70 μmol m^− 2^ s^− 1^ and 70% humidity. For osmotic (300 mM Mannitol), salt (150 mM NaCl) and abscisic acid (0.5 μM ABA) stress treatments, pineapple plants were treated for 24, 48, and 72 h. The leaf tissues from the treated and control plants were harvested and immediately frozen in liquid nitrogen and stored at − 80 °C for RNA isolation.

#### *Arabidopsis* growth and treatments

*Arabidopsis thaliana* L. ecotype (Col-0) was used as wild-type and for transgenic plant generation, and the T-DNA line (SALK_121700) of *Arabidopsis cib2* mutant was obtained from the Arabidopsis Biological Resource Center (Columbus, OH, USA). The seeds were surface sterilized and plated in circular 9 cm Petri dishes, as described previously [41]. The plated seeds were then kept for stratification in the dark at 4 °C for 48 h. After stratification, the plates were moved to a growth chamber and grown vertically at 22 °C in a 16 h light/8 h dark photoperiod. All the experiments were performed from the plant of T3 generation using three independent lines of 35S::*Ac*CIB2-GFP (*Ac*CIB2 ectopic expression) and *Atcib2 +* 35S::*Ac*CIB2-GFP (*Atcib2* complementation) plants. For abiotic stress treatment, wild-type, *Atcib2* mutant, *Atcib2* complemented line and *Ac*CIB2 ectopic expression lines were assayed for germination on Hoagland medium supplemented with or without salt (NaCl 150 mM), osmotic (Mannitol 300 mM), and phytohormone 0.5 μM abscisic acid (ABA). To observe the germination phenotype, photographs were taken after 3 d. All experiments were repeated at least three times.

#### Plasmid constructs and plant transformation

*Ac*CIB2-GFP construct was generated by amplifying 1.3 kb CDS sequence, excluding the stop codon, from pineapple cDNA and cloned to pENTR D-TOPO vector, followed by recombination to pGWB505 destination vector using LR clonase II (Invitrogen, Carlsbad, CA, USA). The construct was confirmed by sequencing before transforming *Agrobacterium tumifecians* GV3101. Finally, wild-type and mutant plants were transformed by the floral dip method [42].

#### Identification, phylogenetic analysis and characterization of bHLH in pineapple

The protein sequences of bHLH transcription factors from *Arabidopsis* and pineapple were downloaded from TAIR (http://www.arabidopsis.org) and Phytozome (https://phytozome.jgi.doe.gov/pz/portal.html). To identify the bHLH genes, we used the plant transcription factor database (http://planttfdb.cbi.pku.edu.cn). We also downloaded the HMM (Hidden Markov Model) profiles for bHLH (PF00010) from Pfam database (http://pfam.xfam.org). The pineapple genome was then searched using the HMM profiles by BLAST-P with an e-value set at 0.01. Using the SMART tool (smart.embl-heidelberg.de), the completeness and existence of the core domain in all the sequences were then verified [43]. For phylogenetic analysis, multiple sequence alignments of bHLH sequences from pineapple and *Arabidopsis* were generated using MUSCLE 3.7 [44] with default parameters. The phylogenetic tree was constructed in MEGA 7 using Neighbor-joining (NJ) method with default parameters and a bootstrap value of 1000. The isoelectric point (pI) and molecular weight (MW) of bHLH proteins were predicted using ExPASy (http://web.expasy.org/compute_pi/).

#### Chromosome location, gene structure and conserved motif analysis of *Ac*bHLH

The chromosome location information of *Ac*bHLH genes was collected from Phytozome and their location on the 25 chromosomes were visualized using MapChart software. Additionally, the *Ac*bHLH genes structure, number of exon and intron were then analyzed to study the evolutionary and structural diversity of bHLH genes, the exon-intron structure of *Ac*bHLHs was illustrated using gene structure and display server (GSDS) [45]. The conserved motifs of pineapple bHLHs were predicted using MEME program [46]. Parameters were set to any number of repetitions, motif width of 10–200 residues, and searching for ten motifs, with all other settings in default.

#### RNA-Seq analysis for different organs in pineapple

Total RNA isolated from various stages of development of gynoecium, ovule, stamen, petal, sepal, root, leaf and flower pineapple was used for library preparation followed by RNA-seq as described previously [47]. Briefly, using Plant RNeasy Mini kit (Qiagen, Strasse 1, Hilden, Germany) the total RNA was isolated. The cDNA library was prepared using NEBNext UltraTM RNA library preparation kit (NEB, Ipswich, MA, USA) following the manufacturer’s protocols. The quality of the libraries was determined on the Agilent Bioanalyzer system and sequenced on a HiSeq2500 sequencing instrument using 150 bp paired-end protocol. After sequencing, raw reads were filtered by removing the adapter sequences and low-quality sequences using TRIMMOMATIC v0.3. The published pineapple genome was used as the reference genome [29], and reads were aligned to the pineapple genome by using TopHat v2.1.1 [48]. Alignment results were processed using Cufflinks, and FPKM values were calculated by using Cuffdiff (FC ≥ 2, FDR ≤ 0.05) following the method described previously [49]. The abundance of pineapple bHLH transcripts was expressed in FPKM (Additional Table S6), and a heatmap was generated based on the log2 (FPKM+ 1) using pheatmap package of R software.

#### Quantitative real-time qRT-PCR

After the RNA isolation from the desired plant sample, TransGen cDNA preparation kit was used to prepare cDNA using one μg of total RNA. The qRT-PCR was carried out using 2X qPCR superMix (TransGen) in 20 μL reaction volumes using Bio-Rad CFX96 Touch™ real-time PCR machine (Bio-Rad, Singapore). The reaction conditions for qRT-PCR included the following steps: 2 min at 95 °C followed by 40 cycles of denaturation for 10 s at 95 °C and annealing for 15 s at 60 °C, and extension for 15 s at 72 °C. At least three biological replicates were used for each experiment with three technical replicates. The fold change in the expression of genes was determined using the Livak method (**2**^−ΔΔ**C**^_***T***_), and the pineapple *ef1*α gene was used as the internal control [50]. The primers used in this study are listed in additional Table S7.

#### Microscopy

For confocal microscopy, the roots of 7-day-old *Arabidopsis* seedlings were mounted on a slide and examined under a TCS SP8 microscope (Leica).

#### Statistical analysis

A two-tailed Student’s t-test was used to analyze statistical significance and results are represented as the mean values ± SE of three biological replicates.

## Supplementary information

**Additional file 1: Table S1.** The properties of bHLH genes in pineapple.

**Additional file 2: Figure S2.** Exon-intron structure of pineapple bHLH genes. Blue boxes indicate untranslated upstream/downstream regions, red boxes indicate exons; black lines indicate introns.

**Additional file 3: Figure S3**. Alignment of amino acid sequences from the bHLH domain region. Gene IDs in red represent pineapple CIB genes.

**Additional file 4: Table S4.** Amino acid sequences from the bHLH domain region. Gene IDs in red represent pineapple CIB genes.

**Additional file 5: Figure S5.** The motif composition of pineapple pHLH proteins. The motifs, numbers 1–10, are displayed in different colored boxes. The number in brackets at the end of each protein represent the domains present in it. The sequence information for each motif is provided in the bottom box. Gene IDs in red represent pineapple CIB genes.

**Additional file 6: Table S6**. The expression profile of pineapple bHLH genes in different tissue and developmental stages. Details of the samples are: sepal Se1–Se4, gynoecium Gy1- Gy7, ovule Ov1–Ov7, petal Pe1–Pe3, stamen St1–St6, fruit ‘Fr_S1–Fr_S7’, root ‘Ro’, leaf ‘Le’, and flower ‘Fl’ where ‘S’ is the abbreviation for ‘stage.

**Additional file 7: Table S7**. List of primers used in the present study.

**Additional file 8: Table S8**. Protein sequences analysed in the present study.

**Additional file 9: Table S9**. DNA sequences analysed in the present study.

## Data Availability

All the data and materials that are required to reproduce these findings can be shared by contacting the corresponding author. All the protein and DNA sequences analysed during this study are included in this published article as additional file S8 and S9. The datasets generated and/or analysed during the current study are available in the NCBI SRA repository under accession number PRJEB38680 [51].

## Abbreviations

bHLH: Basic helix-loop-helix proteins
UTR: Untranslated region
LG: Linkage groups
GSDS: Gene Structure Display Server
HMM: Hidden Markov Models
MEGA: Molecular Evolutionary Genetics Analysis
MEME: Multiple Em for Motif Elicitation
RT-qPCR: Real-time Quantitative PCR
SMART: Simple Modular Architecture Research Tool
*At*CIB2: *Arabidopsis thaliana* CIB2
*Ac*CIB2: Pineapple (*Ananas comosus*) CIB2
FPKM: Fragments Per Kilobase Million
