# Supplementary material for: Genome-wide study of pineapple (Ananas comosus L.) bHLH transcription factors indicates that cryptochrome-interacting bHLH2 (AcCIB2) participates in flowering time regulation and abiotic stress response
Source: BMC Genomics. 2020 Oct 22;21:735. doi: 10.1186/s12864-020-07152-2 (PMC7583237; doi:10.1186/s12864-020-07152-2)
Supplement: Supplementary file 1 — Additional file 1: Table S1. The properties of bHLH genes in pineapple. [file 12864_2020_7152_MOESM1_ESM.docx]

**Table S1.** The properties of bHLH genes in pineapple.

| Gene | Gene ID | MW (kDa) | pI | Amino Acid | Location | ORF | Exons | Introns | Chr |
| --- | --- | --- | --- | --- | --- | --- | --- | --- | --- |
| *Ac*bHLH1 | Aco006737 | 3.87 | 5.31 | 348 | 22317729..22328759 | 1044 | 9 | 8 | 01 |
| *Ac*bHLH2 | Aco009603 | 6.16 | 5.04 | 540 | 972985..982485 | 1623 | 14 | 13 | 01 |
| *Ac*bHLH3 | Aco009661 | 1.84 | 9.47 | 165 | 589341..590885 | 498 | 3 | 2 | 01 |
| *Ac*bHLH4 | Aco011419 | 2.50 | 8.66 | 225 | 11721923..11724076 | 225 | 5 | 4 | 01 |
| *Ac*bHLH5 | Aco012350 | 3.96 | 4.82 | 359 | 2776325..2778193 | 1088 | 5 | 4 | 01 |
| *Ac*bHLH6 | Aco015075 | 5.54 | 6.47 | 517 | 24608249..24611828 | 1554 | 4 | 3 | 01 |
| *Ac*bHLH7 | Aco015882 | 3.67 | 6.02 | 332 | 10968499..10974303 | 999 | 4 | 3 | 01 |
| *Ac*bHLH8 | Aco017355 | 4.83 | 6.12 | 447 | 23696099..23698751 | 1344 | 8 | 7 | 01 |
| *Ac*bHLH9 | Aco018208 | 4.40 | 6.90 | 409 | 13784959..13786424 | 1230 | 5 | 4 | 01 |
| *Ac*bHLH10 | Aco000773 | 5.03 | 9.06 | 458 | 16792284..16796112 | 1377 | 6 | 5 | 02 |
| *Ac*bHLH11 | Aco000904 | 5.34 | 8.58 | 489 | 15778462..15787108 | 1470 | 13 | 12 | 02 |
| *Ac*bHLH12 | Aco000913 | 5.32 | 5.95 | 487 | 15711010..15715504 | 1464 | 9 | 8 | 02 |
| *Ac*bHLH13 | Aco001074 | 3.43 | 7.21 | 319 | 14405087..14407404 | 960 | 2 | 1 | 02 |
| *Ac*bHLH14 | Aco001133 | 9.66 | 6.57 | 85 | 14019416..14020497 | 258 | 2 | 1 | 02 |
| *Ac*bHLH15 | Aco001136 | 1.04 | 6.90 | 95 | 13963856..13964791 | 288 | 3 | 2 | 02 |
| *Ac*bHLH16 | Aco001255 | 3.56 | 5.51 | 325 | 12975135..12977620 | 978 | 3 | 2 | 02 |
| *Ac*bHLH17 | Aco001282 | 345.97 | 8.90 | 3097 | 12705963..12741872 | 9294 | 50 | 49 | 02 |
| *Ac*bHLH18 | Aco001331 | 23.92 | 8.32 | 218 | 12272540..12273196 | 657 | 1 | 0 | 02 |
| *Ac*bHLH19 | Aco012193 | 26.50 | 5.91 | 237 | 456191..458055 | 714 | 5 | 4 | 02 |
| *Ac*bHLH20 | Aco016928 | 31.58 | 4.92 | 310 | 2158540..2164822 | 933 | 7 | 6 | 02 |
| *Ac*bHLH21 | Aco011934 | 51.20 | 6.09 | 469 | 13673164..13680248 | 1410 | 14 | 13 | 03 |
| *Ac*bHLH22 | Aco011985 | 29.91 | 6.98 | 279 | 14121682..14123052 | 840 | 4 | 3 | 03 |
| *Ac*bHLH23 | Aco012028 | 74.14 | 5.51 | 657 | 14470469..14476653 | 1974 | 9 | 8 | 03 |
| *Ac*bHLH24 | Aco012816 | 48.21 | 8.44 | 456 | 14911993..14915420 | 1371 | 5 | 4 | 03 |
| *Ac*bHLH25 | Aco012937 | 33.15 | 6.26 | 303 | 15891697..15896145 | 912 | 3 | 2 | 03 |
| *Ac*bHLH26 | Aco017264 | 55.77 | 5.26 | 502 | 16471638..16476465 | 1509 | 4 | 3 | 03 |
| *Ac*bHLH27 | Aco024469 | 39.14 | 7.66 | 356 | 8046225..8053689 | 1071 | 6 | 5 | 03 |
| *Ac*bHLH28 | Aco027896 | 49.91 | 5.67 | 453 | 13120434..13130415 | 1362 | 6 | 5 | 03 |
| *Ac*bHLH29 | Aco025839 | 18.92 | 9.85 | 169 | 5349241..5351713 | 510 | 2 | 1 | 03 |
| *Ac*bHLH30 | Aco002151 | 24.20 | 10.76 | 225 | 3948373..3950754 | 678 | 2 | 1 | 04 |
| *Ac*bHLH31 | Aco002225 | 62.25 | 5.50 | 559 | 3191272..3195402 | 1677 | 8 | 7 | 04 |
| *Ac*bHLH32 | Aco002246 | 32.60 | 6.72 | 293 | 2966466..2968822 | 879 | 3 | 2 | 04 |
| *Ac*bHLH33 | Aco002333 | 29.07 | 9.03 | 255 | 2351158..2352400 | 768 | 3 | 2 | 04 |
| *Ac*bHLH34 | Aco011121 | 16.44 | 5.21 | 150 | 13568317..13570623 | 453 | 4 | 3 | 04 |
| *Ac*bHLH35 | Aco021988 | 44.21 | 5.99 | 394 | 215914..217770 | 1185 | 3 | 2 | 04 |
| *Ac*bHLH36 | Aco022096 | 71.91 | 5.84 | 665 | 46726..50404 | 1995 | 9 | 8 | 04 |
| *Ac*bHLH37 | Aco023468 | 47.24 | 6.31 | 429 | 6052949..6055332 | 1290 | 7 | 6 | 04 |
| *Ac*bHLH38 | Aco023519 | 44.16 | 5.95 | 394 | 351345..353202 | 1185 | 3 | 2 | 04 |
| *Ac*bHLH39 | Aco004377 | 37.40 | 5.83 | 342 | 2756028..2758536 | 1029 | 3 | 2 | 05 |
| *Ac*bHLH40 | Aco004559 | 47.02 | 6.90 | 424 | 4014760..4017688 | 1275 | 8 | 7 | 05 |
| *Ac*bHLH41 | Aco004647 | 40.54 | 8.96 | 365 | 4755646..4760854 | 1098 | 9 | 8 | 05 |
| *Ac*bHLH42 | Aco004686 | 28.88 | 8.68 | 267 | 5035516..5039932 | 804 | 6 | 5 | 05 |
| *Ac*bHLH43 | Aco012714 | 35.17 | 6.13 | 315 | 14133542..14135066 | 948 | 5 | 4 | 05 |
| *Ac*bHLH44 | Aco015158 | 20.31 | 9.72 | 176 | 1725206..1726761 | 531 | 3 | 2 | 05 |
| *Ac*bHLH45 | Aco019347 | 28.44 | 5.20 | 257 | 6225018..6225791 | 774 | 1 | 0 | 05 |
| *Ac*bHLH46 | Aco027360 | 20.31 | 9.72 | 176 | 1872405..1873960 | 531 | 3 | 2 | 05 |
| *Ac*bHLH47 | Aco002862 | 9.75 | 7.93 | 86 | 12161918..12163605 | 261 | 2 | 1 | 06 |
| *Ac*bHLH48 | Aco003064 | 58.61 | 6.36 | 536 | 13569068..13579794 | 1611 | 16 | 15 | 06 |
| *Ac*bHLH49 | Aco003149 | 35.51 | 8.62 | 320 | 14122668..14127433 | 963 | 6 | 5 | 06 |
| *Ac*bHLH50 | Aco021068 | 53.77 | 5.50 | 494 | 7139845..7144410 | 1485 | 9 | 8 | 06 |
| *Ac*bHLH51 | Aco004914 | 36.61 | 4.77 | 333 | 756886..761996 | 1002 | 8 | 7 | 07 |
| *Ac*bHLH52 | Aco014442 | 43.48 | 5.95 | 399 | 14242956..14245451 | 1200 | 7 | 6 | 07 |
| *Ac*bHLH53 | Aco014447 | 26.30 | 4.73 | 238 | 14170604..14172572 | 717 | 4 | 3 | 07 |
| *Ac*bHLH54 | Aco014454 | 25.92 | 5.62 | 236 | 14137091..14140219 | 711 | 5 | 4 | 07 |
| *Ac*bHLH55 | Aco020380 | 27.86 | 7.79 | 263 | 4372857..4376577 | 792 | 6 | 5 | 07 |
| *Ac*bHLH56 | Aco020381 | 28.21 | 6.45 | 264 | 4386453..4387436 | 792 | 3 | 2 | 07 |
| *Ac*bHLH57 | Aco025287 | 28.02 | 6.92 | 266 | 4337968..4341688 | 801 | 6 | 5 | 07 |
| *Ac*bHLH58 | Aco011852 | 49.08 | 6.87 | 447 | 11047639..11051445 | 1344 | 7 | 6 | 08 |
| *Ac*bHLH59 | Aco011855 | 32.70 | 5.27 | 297 | 11008994..11010075 | 894 | 2 | 1 | 08 |
| *Ac*bHLH60 | Aco018875 | 61.50 | 5.93 | 570 | 13192430..13194167 | 1713 | 2 | 1 | 08 |
| *Ac*bHLH61 | Aco026878 | 27.83 | 8.17 | 249 | 13314138..13318011 | 750 | 3 | 2 | 08 |
| *Ac*bHLH62 | Aco008553 | 40.66 | 6.41 | 366 | 703789..705796 | 1101 | 5 | 4 | 09 |
| *Ac*bHLH63 | Aco008686 | 51.00 | 6.21 | 464 | 1560002..1562710 | 1395 | 8 | 7 | 09 |
| *Ac*bHLH64 | Aco009100 | 37.01 | 7.71 | 339 | 13323096..13324115 | 1020 | 1 | 0 | 09 |
| *Ac*bHLH65 | Aco015791 | 55.00 | 6.32 | 498 | 10539780..10544345 | 1497 | 7 | 6 | 09 |
| *Ac*bHLH66 | Aco017396 | 33.36 | 6.05 | 316 | 3000027..3006310 | 951 | 8 | 7 | 09 |
| *Ac*bHLH67 | Aco026239 | 29.97 | 8.57 | 278 | 4188849..4200182 | 834 | 7 | 6 | 09 |
| *Ac*bHLH68 | Aco009909 | 54.11 | 8.31 | 485 | 1315610..1318267 | 1458 | 8 | 7 | 10 |
| *Ac*bHLH69 | Aco010678 | 25.57 | 9.13 | 232 | 2657993..2661169 | 699 | 2 | 1 | 10 |
| *Ac*bHLH70 | Aco010845 | 65.09 | 6.12 | 600 | 4506728..4508530 | 1803 | 1 | 0 | 10 |
| *Ac*bHLH71 | Aco016398 | 47.72 | 6.73 | 422 | 12360619..12365063 | 1269 | 8 | 7 | 10 |
| *Ac*bHLH72 | Aco016415 | 24.04 | 9.77 | 216 | 12065451..12069454 | 651 | 3 | 2 | 10 |
| *Ac*bHLH73 | Aco016434 | 34.17 | 5.34 | 311 | 11827093..11828412 | 936 | 5 | 4 | 10 |
| *Ac*bHLH74 | Aco020569 | 41.69 | 6.21 | 375 | 12672656..12678034 | 1128 | 6 | 5 | 10 |
| *Ac*bHLH75 | Aco005342 | 31.16 | 4.98 | 274 | 9248662..9250987 | 825 | 4 | 3 | 11 |
| *Ac*bHLH76 | Aco005770 | 45.02 | 4.91 | 411 | 12796640..12797875 | 1236 | 1 | 0 | 11 |
| *Ac*bHLH77 | Aco021761 | 38.00 | 6.46 | 345 | 7770430..7778553 | 1038 | 7 | 6 | 11 |
| *Ac*bHLH78 | Aco000179 | 10.38 | 9.17 | 92 | 3934890..3935924 | 279 | 2 | 1 | 12 |
| *Ac*bHLH79 | Aco000186 | 29.78 | 6.37 | 267 | 3867571..3872017 | 804 | 5 | 4 | 12 |
| *Ac*bHLH80 | Aco000272 | 38.01 | 6.65 | 349 | 3160899..3163293 | 1050 | 4 | 3 | 12 |
| *Ac*bHLH81 | Aco000605 | 26.70 | 5.27 | 241 | 697592..698406 | 726 | 2 | 1 | 12 |
| *Ac*bHLH82 | Aco010917 | 37.69 | 7.68 | 343 | 6585876..6590217 | 1032 | 6 | 5 | 12 |
| *Ac*bHLH83 | Aco012482 | 18.86 | 6.07 | 169 | 1484021..1484530 | 510 | 1 | 0 | 13 |
| *Ac*bHLH84 | Aco013643 | 65.18 | 6.31 | 604 | 11518871..11523671 | 1815 | 10 | 9 | 13 |
| *Ac*bHLH85 | Aco014156 | 24.68 | 8.20 | 223 | 479199..482865 | 672 | 5 | 4 | 13 |
| *Ac*bHLH86 | Aco014880 | 25.56 | 5.69 | 230 | 307334..310208 | 693 | 5 | 4 | 14 |
| *Ac*bHLH87 | Aco014894 | 45.63 | 6.13 | 420 | 224858..227294 | 1263 | 7 | 6 | 14 |
| *Ac*bHLH88 | Aco025362 | 28.11 | 9.61 | 260 | 4738771..4741696 | 783 | 2 | 1 | 14 |
| *Ac*bHLH89 | Aco004054 | 35.34 | 5.09 | 316 | 1718242..1719902 | 951 | 3 | 2 | 15 |
| *Ac*bHLH90 | Aco004136 | 10.24 | 9.75 | 91 | 1142587..1143634 | 276 | 2 | 1 | 15 |
| *Ac*bHLH91 | Aco004138 | 8.17 | 5.74 | 71 | 1101495..1101997 | 216 | 2 | 1 | 15 |
| *Ac*bHLH92 | Aco013347 | 35.47 | 6.54 | 323 | 11030254..11032852 | 972 | 10 | 9 | 15 |
| *Ac*bHLH93 | Aco013365 | 31.84 | 5.78 | 293 | 10873670..10875096 | 882 | 2 | 1 | 15 |
| *Ac*bHLH94 | Aco019856 | 37.34 | 5.96 | 353 | 8908516..8910505 | 1062 | 2 | 1 | 15 |
| *Ac*bHLH95 | Aco005839 | 57.76 | 7.01 | 531 | 11017707..11019512 | 1596 | 2 | 1 | 16 |
| *Ac*bHLH96 | Aco005872 | 43.95 | 7.59 | 392 | 10755357..10759436 | 1179 | 6 | 5 | 16 |
| *Ac*bHLH97 | Aco003204 | 32.56 | 9.17 | 295 | 1091224..1094383 | 888 | 3 | 2 | 17 |
| *Ac*bHLH98 | Aco003613 | 38.18 | 6.50 | 342 | 4233721..4235597 | 1029 | 7 | 6 | 17 |
| *Ac*bHLH99 | Aco016776 | 92.57 | 5.89 | 843 | 789850..795799 | 2532 | 10 | 9 | 17 |
| *Ac*bHLH100 | Aco022880 | 63.28 | 5.45 | 596 | 9123810..9129170 | 1791 | 4 | 3 | 17 |
| *Ac*bHLH101 | Aco026511 | 39.89 | 7.69 | 357 | 2243843..2260402 | 1074 | 7 | 6 | 18 |
| *Ac*bHLH102 | Aco027966 | 33.57 | 5.24 | 305 | 3353113..3371838 | 918 | 8 | 7 | 19 |
| *Ac*bHLH103 | Aco029228 | 31.38 | 5.96 | 284 | 5570860..5577103 | 855 | 5 | 4 | 20 |
| *Ac*bHLH104 | Aco018903 | 33.32 | 5.83 | 305 | 7389483..7400366 | 918 | 8 | 7 | 20 |
| *Ac*bHLH105 | Aco008079 | 28.00 | 8.84 | 252 | 8083988..8091831 | 759 | 7 | 6 | 21 |
| *Ac*bHLH106 | Aco016081 | 33.57 | 4.75 | 293 | 1103574..1105015 | 882 | 3 | 2 | 21 |
| *Ac*bHLH107 | Aco016125 | 40.01 | 5.07 | 353 | 1588442..1592377 | 1062 | 8 | 7 | 21 |
| *Ac*bHLH108 | Aco023503 | 45.61 | 5.27 | 407 | 3242657..3244231 | 1224 | 3 | 2 | 21 |
| *Ac*bHLH109 | Aco007275 | 76.66 | 5.39 | 684 | 3001669..3007952 | 2055 | 9 | 8 | 23 |
| *Ac*bHLH110 | Aco010168 | 50.12 | 6.48 | 473 | 3131866..3137573 | 1422 | 8 | 7 | 25 |
| *Ac*bHLH111 | Aco010345 | 25.70 | 6.14 | 236 | 1897380..1900168 | 711 | 5 | 4 | 25 |
| *Ac*bHLH112 | Aco013073 | 35.46 | 5.02 | 322 | 866316..870164 | 969 | 4 | 3 | 25 |
| *Ac*bHLH113 | Aco013092 | 53.10 | 5.83 | 475 | 651741..655504 | 1428 | 8 | 7 | 25 |
| *Ac*bHLH114 | Aco021969 | 25.92 | 5.62 | 236 | 351:127479..130607 | 711 | 5 | 4 | scaffold |
| *Ac*bHLH115 | Aco021976 | 26.30 | 4.73 | 238 | 351:160947..162855 | 717 | 4 | 3 | scaffold |
| *Ac*bHLH116 | Aco027975 | 33.15 | 6.26 | 303 | 1258:29417..33687 | 912 | 3 | 2 | scaffold |
| *Ac*bHLH117 | Aco028213 | 65.92 | 5.55 | 591 | 1506:15099..19314 | 1776 | 8 | 7 | scaffold |
| *Ac*bHLH118 | Aco028872 | 29.07 | 9.03 | 255 | 1930:11904..13139 | 768 | 3 | 2 | scaffold |
| *Ac*bHLH119 | Aco030561 | 19.68 | 8.91 | 178 | 1436:13460..18068 | 537 | 5 | 4 | scaffold |
| *Ac*bHLH120 | Aco030901 | 69.07 | 6.60 | 617 | 1362:3232..8274 | 1851 | 9 | 8 | scaffold |
| *Ac*bHLH121 | Aco031432 | 26.66 | 6.67 | 240 | 2196:13835..15074 | 720 | 4 | 3 | scaffold |
