# Supplementary material for: Genome-wide study of pineapple (Ananas comosus L.) bHLH transcription factors indicates that cryptochrome-interacting bHLH2 (AcCIB2) participates in flowering time regulation and abiotic stress response
Source: BMC Genomics. 2020 Oct 22;21:735. doi: 10.1186/s12864-020-07152-2 (PMC7583237; doi:10.1186/s12864-020-07152-2)
Supplement: Supplementary file 8 — Additional file 8: Table S8. Protein sequences analysed in the present study. [file 12864_2020_7152_MOESM8_ESM.docx]

>Aco006737; AcbHLH1

MNRLRSQAQDSPQQPSSSSSSAPPPPPSSSFSSLSSSSSSPSSCSSSSMYTHYTNQSNLPNTTLYRQDNQIFPKSWSQLLLDGLVEEKEEEEDDDDDRMISSSFAPHFQPKLVENWKDQVLYPPPNTNIVGPKQEGYESGFMYQNKTEDIQITMRAPFWSHALHASSINSCATTSLSSSSSSMLDISKSKPDSNNLPLEISSDQCNSTTGSALKKARVQISSVSQPSIKVRKEKLGDRITALHQLVSPFGKTDTASVLQEAIGYIRFLHHQIEALCLPHLVDGSTNSRQLEKDERNDVSHEVQFQDQDACDEPKKDLRSKGLCLVPISLILYVDRDNKANYWVYLEPT

>Aco009603; AcbHLH2

MVVDKGKRCKVAERGEESDRVDGELVPYIEKLQEIQDELEKVNEEASDKVLEVEQKYNEIRRPVYMRRSDIIKSIPDFWLTAFLSHPVLGDLLNKEEQKIFRHLISLDVEDFKDLKSGYSITFNFSPNPYFEDTCLRKTYSFSDEGTTNITGTTIKWKEGMNCVNGEARENNGSKRPFGEESFFSWFSETHPKRMPEMFSDEIAEIIKEDLWPNPLKYFNHEAGDEFEGDEDDDEGRWDGRFCGEFLEARQMVHGVNGSDPDERESEEARIRDLGTAWGTQPNQKTGSGVRNLSMVSESEQSSSMANGEIVAERPAGRSVPSKKSQAQVPKKIHKAEREKLKRDQLNDLFSKLDKMLEPDRQNNGKACILSDTTRIVRDLIAQVESLRKENAALQTESHYVTTEKNELRDENAVLQAQISDLQCEVRARMGYNGVWTNPPNVNTINALHPTTNPMLQQPPVIERAFTTPPRELQLFPGAAGSPDHESPSPRVRRPHASYATPSNTWTERLLPGLRREAQEEEQQQSSSGITESSREDRVG

>Aco009661; AcbHLH3

MMRERQRREKLSQSYADLHSILSTRSKGDKNSIVQSAALYIRELKGVRNELQRRNEEFKARLVGDDANVEGVKVKFEVANPSSTIDSMIGALRCLKNMDVKARAMRSNFSGHVLLTVMSIETKMSVSEVEKAIEGALANAETNKNQFPFHGSGGWALNSHVENMT

>Aco011419; AcbHLH4

MGIKMLWLTGMTTTRGIGNYSQAGSDAVHAMANSRLRSQWSFSRQDSVLSQISEMSIPEIGESGNSSDEATGHAGQSYISSNLLGASWEDTNSIMFSSPGKRGKESNGDVITSLSNFDSQFGMPKTSAEFADMERYLQMHQDSVACKVRAKRGCATHPRSIAERERRTRISKKLRKLQDLVPNMDKQTSTSDMLDLAVHYIKELQSQVQKLNQEQENCTCTSKKI

>Aco012350; AcbHLH5

MENEGLALEATWSSFEESEIMAQLLGGAVATNYFPGDQEQEPTNGMHLMFWPNYDSDSNFSSPTDVNYNSFHWPQSISTPCISTSTGSFLLSNPCYGGGCYLGDTNLVPHNTTSSFDVDFNRRYCDLPSMTEEACDDNELGLSIANQMRPAVFADEPSHAAKRKFGLGDNEKPIEDVKDDDTSSLVPRKKARVTMGNVSIQAQERNKNADTKKPLKNEENGNADFHGQSSSSCSSEDDSSGSQEQMEGGGNTSSRSKKSETTSVTGKTRAGRGSATDPQSLYARKRRERINERLRILQNLVPNGTKVDISTMLEEAVQYVKFLQLQIKLLSSDELWMYAPLAYNGICISPDLKISPPQL

>Aco015075; AcbHLH6

RSEREPPRPSLPPRRLRAWPEPSPSSSLPPPPPQRLLRRGGGGVRILGRGQEGWTRGLGSHLQVLLEDDADAEAAAAAAAAGSSAPTLPLRRLRPPPHPTPPPPPPPRTPFLTLPFPLPLLPSSHHDAWPLCGAPNPFASPFDLPDPASAASLFPIPDPNPSPQLGNPALAFHQTGFAPFDHPAAAAAAPPFPNRPKALKPLEIVPPVGARPNLFQKRAAAALKHNLPVGLGLEKRRRGNGDEDDEFGDQSIDGSGLNYDSDEVNVENVKGEGDDEEEDEEDDEEGGVGNSGVGSEVNLNSVASGSGARGGSGKGKNKKGLPAKNLMAERRRRKKLNDRLYMLRSVVPKISKMDRASILGDAIDYLKELLQRINDLHNELESMPSSSSVPGSNTTTPTPSFHPLTPTPQTLPSRVKEELCPSSLPSPTSQPARVEVRVREGRAVNIHMFCSRRPGLLLSTMRALDNLGLDVQQAVISCFNGFAMDVFRAEQCRDGPGVLPEEIKAVLMHSAGFQDAM

>Aco015882; AcbHLH7

MATTQWFSQLEMDEGSFFQQWEETAAAAADSLHHFTEEQLSILGFGQQDQQQQLAQAICTDKPRKVAPKSCSWDSCITTTTTTTTTTTTTDQSSSPSFLSFSNSDVEQPSCHLHDHHHQQLKKEEVEISFPHHQQGGVKRKFEALVSEGLRKVNTTTTAATTRPVSQNQEHIIAERKRREKLSQRFIALSAIIPGLKKMDKASVLGDAVKYLKQLQEKVKTLEEQSRKRTVETAVLVKKSQLSPDDESSSCDGESSAIDGSSCGPEIEAKMSEKSVLVKIHCENRKGVLVRALSEIESHRLAIVSTSVVPFAGSSIDITPTVVPHAILVEDV

>Aco017355; AcbHLH8

MNCGPPPPPPPPPPPPGDLCFFNLNWDQSIDPNGEISSQPHYVAAAPLNSPPKLDLSMISHLHHTQQAQGSLPISEGLMPITHLDQFPTDSGFVERAARFSCFDGGGGGYAGFGRSLTANNVELGNAREESSVSDPASASGEVSLKGPPFESNARKRKAAPKGKGKETCSTISSNDPPKNSEAKDPNAKKCKLGESEGTKDDSIKPKVERNNASTTDNGNGVQKQGKNNTKPPEPPKDYIHVRARRGQATDSHSLAERVRREKISQRMKLLQDLVPGCNKVTGKALMLDEIINYVQSLQRQVEFLSMKLATVNPNLDFNNLPNLLAKDMHQTCGGPLVNSIFPLETSGVALSYASQPQHGNLLNCSIANVLENHCSINPLDSALCRTMNTHHPYFNGIGDAASQMGTFWEDDLQSVVQMDIGQTQEMVVSSQSFNGPSESVHMKMEL

>Aco018208; AcbHLH9

MEELISTPSSCSPPASQQHFLGAGPGGRTPSSGAAPSEHHHHHAHPPHLVLLSFGDGHFRGTRDGEHQRRRGVDQEGEGEGAGAGGDGDEAEWFYVVSLTRSFGVGDAAAPARAYVASAPLWLTGAHAMAASGCERAREAHLHGIETLVCVPVPGGVLELGSADLIPENWVLGERRRGAKKRGRRPGTGRETPVNHNWVLVQQANSILSSDAPHHHLAAADPSLRAASLDSEHSDSDAAGLLQGERRRGAKKRGRRPGTGRETPVNHVEAERQRREKLNHRFYALRSVVPNVSRMDKASLLADAVAYIKELRAKVDDLEADAKRARNDRILEVKVVGSDALIRAQSENAGHPTAVLMAALRDLELDVRHACVANVKEVVLQDVIVTVPYALQTEDSLRTALLNSLEKSC

>Aco000773; AcbHLH10

MYGSPQAARSKDLNLPVSAEPAFGHRKEEKELLQRHHHHHHHRDHQQHQHHNPPPPPPPPHHQQQQQQQQQMSSGLLRYRSAPTALLGEVVDDFLPVRPSSPETETMFARFLATSDLRDEIREKPPSAAAQRSSTFASAMDEAAASQQQQQQQQQQMMYHSHHQPMPSHGSVESLYRTASSAAIDLEPMKSDGATNNLIRQSSSPPGIFSHVNVDNGYSVMRGIGGFRNGNNSLGAVNAANTNSTSNRMKGQISFSSRQGSLMSQISGMGSEGGAIGGSSPEDGGNSGGTAAGGGRYIPGYPLGSWDDPPLLSDHFSGSLKRGREAEGKIISGLNQSEHQNGLMHQFSLPKTSSEMAAIEKFLQFHDAVPCKIRAKRGCATHPRSIAERVRRTRISERMRKLQELVPNMDKQTNTADMLDLAVDYIKDLQKQVKTLTENRASCTCSSSKQKQYQNPAA

>Aco000904; AcbHLH11

MELQGKKATHDFLSLYTKDSDPKPPPQGFYLKTHDFLQPLERAEEARKKKRGDESAAVGPAEHVLPGGIGTFSISHVSDPRARAAVKSERGTCAPAPGFGPESKPESVPFALWGACADHRGQWSSPFAAHVSGSASFGSVSSASSPLNFVTELGVKIDGNGRGGGNDQRPNTPRSKHSATEQRRRSKINDRQVQAEVFYYSKSLKSKSSICYSGSRIALDEFQILRELIPHSDQKRDKASFLLEVIEYIRFLQERVQKYESSCPEWNEDNVKMMPWVKVYFRSFWKNSRNNNRSPENFISDSSQINKSGSAHPQQTLLGKINDNHIQIAHTVTSDAPNPTELNQMAGVSFKATENPPHCPDNNTFQAQNQWLRSPLVADSAFSNEMLNQHEELAIDDGTINVSCHYSQELLTTLSQALQNSGLDLSQASISVQINLGKRAANKRSAAVSTSSPKDHVPAVNNQASEHARAGNTGQEFSEAPKRRRVDNS

>Aco000913; AcbHLH12

MDMNEKEKLGLEKNEDHMSYHASDISTAWQQFNSSSGAIGQEIQMGSATCPSNPMVDSFNPSLWNHHPSSQNLVGLSENNAHVGNPPMGLNPSSFFPKGGTFLPSLPKIPPPPILPHFSADTGFIERAARFSCFGSGNLSGMVNPFGASEILSPYANPSKFSGQKSEMSVDEASENAPLPVNDHLSSIRSPRERRDDANSLRESAEPEFSGGGEEEAPNLATAARDNSSSKGPNAKKRKRSNQGGAETEQVEGAPQVPIETTKENMDNKQKNEQNSSTVGTSKASGKQAKDNSDLPKEDYIHVRARRGQATNSHSLAERLRREKISERMKFLQDLVPGCSKVTGKAVMLDEIINYVQSLQRQVEFLSMKLAAVNPRLDFNIEGLLSKELFSRGGPSSAIGFSPDMIHPHLHPSQHGMVQAGMPSIANPSDVLRRVMNAQPTSINGYKEPTSQMPNTWDEELQNVMQMSYPPLNAQELNSKPRDGFPL

>Aco001074; AcbHLH13

MWEDRVHGLHYTATNTSSTSTTTGATILGGDTATGLLHNPTTILPWAVNPSAYSLPYHAPFNGDFYQPERNFPALGASTDQADLSGLHIGSSTERLLHGKAGGLASTMSIFGSLHAEFGKMSAKEIMDAKALAASKSHSEAERRRRQRINGHLAKLRSLLPNTTKTDKASLLAEVIEHVKELKRQTTAITAEGGTLLLPTEADELTVDAAGSDEDGRLVVRASLCCDDRSDLIPDLARALKSLKLRARRAEIATLGGRVKNVFIITAHDDACHSDCVASIQETLRAVMDKKTATSDTSSSSSGIKRQRTNRVDEQQGSI

>Aco001133; AcbHLH14

MSSRSRITEEEINELISKLQSLLPDQARRRGSSRASATKVLKETCNYIKSLHREVDDLSDRLSDLMASMDSDSPQAEILRNLLRS

>Aco001136; AcbHLH15

MSSRRPRSPRSSTSRITEDQINNLLSTLQHLLPTSLVGNTNRVSAARLLHDICNYIRSLHEEADHLSERLAELLAATDASSAQADILKPSSTSNL

>Aco001255; AcbHLH16

MALEAVVFPHGLLGYTMKELCSMGGGGGGGGGCSHGELGWFEEEEEKGVLLGYLEEGGGGDEQSGKRKWDVNWVDTSCSSMVGVFEEWDNVNSLSPPPEPAGGRRKRRRMKSIKNKEEVENQRMTHIAVERNRRKQMNEYLAVLRSIMPPSYVQRGDQASIIGGAINFVKELEQLLQSLEAQKRSNEQHSESAPFAEFFTFPQYSSYTARSPNATAAMVANESAAAAEEAKGSAAAADIEVTMVESHANLKVLAKRRPKQLLKLVVGLQNLRLTTLHLNVTTLDRMVLYSFSLKVEDDCQLSSVDDIAAAVHHIVGKVEEEAAML

>Aco001282; AcbHLH17

MAEPKNRWTWEVPGFEPRRSYEPQESSVAHTVVRRLSVSPSTLLPRAEAPPRPSVAAKLQRLKEQVKHARNDYLELRQEATDLREYSSAKLDRVTRYLGFLADRAHKLGNVKVYCRTRPLFEEEGSSVVEFPDDFTIRVNTGDDSLANPKKDYEFDRVYGPHVGQGELFRDVQPFIQSALDGYNVCLFAYGQTHSGKTHTMEGSIHERGLYLRSFEELFDLSNLDTTSTSQYSFYVTAFELYNDQVQDLLLESRSAMPRVRMGPEDSFVELVHERVQNPLDFSKVLSAALKKRGTDSSKAIVSHLIVTIHIHFTNCITGDRIYSKLSLVDLPGSECLLVEDASRDHVTDFLHVSKSLSALGDVLSSLTSKKEIVPYENSRMTQILADSLGGGSKTLLIAHLCPNALNLPRTLSTLNFSARARNTELSLGNRDTIKKWRDVANDSRKELQQKEREVQDLRNEVLQLKLALDGANDQCTLLFNEVQKAWKVSFTLQADLKSENLMLADKQKAEKEQNNQLKNQVAHLLQLEQEQKMQIHERDLTIKSLQAKLKTIESQLNEALNSSDSRSTLGSESGSTGVLSTPKMEASVDSSSVTKKLEEELSKRDALIEKLHEENEKLFDRLTEKSGLGGSPQVSSPSTKRTVNTQSRDLSRSDTSRGRSQDVLQLPRTQDKTVTTGALVKSSNEIAKTTPAGEYLTTALMDFDPDQFESFAAIADGANKLLMLVLAAVIKAGAAREHEILAEIRDAVFAFIRRMEPRKVMDTMLVSRVRILYIRSLLARSPELQSIKVSPVERFLEKANTGRSRSSSRGSSPGRSPVYHDSSTRSSLIDEHVHGFKVNIKQEKKSKFSNIVLKLRGIDQETWRQHITGGKLREITEEAKAFAIGNKALAALFVHTPAGELQRQIRLWLAENFEFLSVTGGDALGGTTGQLELLSTAIMDGWMAGLGTAQLPSTDALGQLLSDYSKRVYTSQLQHLKDIAGTLATEEADDLAHVNKLRSALESVDHKRRRILQQMRNDTALLTKEEGGSPIQNPSTAAEDARLASLISLDAILKQVKELMRQTSVSSLTKTKKKAMLASLDELMGRMPSLLDIDHPCAQKEIMGARRAVESIPEVEGQFDEAPRGLNSYSESTSTGESEVSQWNVLQFNTGSTTPFIIKCGANSNCELVIKADARVQEPKGGEIIRVLPRPTVLADMGFEDIKQAFEQLPESVSLLALARTADGTRARYSRLYRTLASKVPALKDLSTIGNPQNEAKMSPRKDRTSESYGGVISPRFPSTYFYLQMSSSLTCTRLTIWAFAAVLLQIAGLSLFLIGFFPVKPTLPGFSGPESYRMPTSDPVSDAEEVEELPPDRLRSLYREVSKIPPVYDRLILMVIDGLPAEFVLGRGNKPPTKNMMEAMPYTHSLLSSGKAAAYHAKAAPPTVTMPRLKAMVSGAIGGFLDVAFNFNTQAMLEDNLLEQFYRIGWKLVIHGDETWIKLFPRLFHRQDGVSSFYVKDTVEVDFNVSRHLEAEFASTDWNFLILHYLGLDHVGHIGGRQSILMAPKLKEMDDVIKRIHMNSILDQENSDSHTLLVVVSDHGMTDGGNHGGSSYEETDSLALFIGHGVEESNYSPYDHNEAFQVDIAPTLALLFGVPIPKNNIGVLLTKLFDSLTDDQRLRSLELNSWQLLRLLKAHLPGLHCGESSCYSCEKGLETNVPTSEAKQRLCYLFSKAISAHNSWQFRQHSDFISADINYFQVAAESYNDFLRNASEWLSHRATDKPVNVLMSAIIMMLVSSVLLMGIVFCLFRRVHFAQVGCCSQLEDPYKILHLDEVFVFIVIFLHVLSLGASSLVEEEQYTWHFLTSTLYLIFLSRTIESLLKRPNSVTLQNKDDKTVLPHSSPTDARNNSGHKFTKFRPSKINNHGTCRLFSVVVVLICGRLLRGWHQGGVNWIYLPDISKLLVHAGTSTIKVCQILSLFAIIILGSHAIFKLKSTTNFVYGVWSSLLLSAFLVMLHTMENHIYDLEPMNLSTTSIAQVFYVVASTSAVLTFLASPWIFPVYSMEKQMVYQVRSNSCSTKQRDSFLLSIRETTYLIGTTYTAFWCLLQLLIQQPINAIPILLIYLQILFSIIYFSADRSFHRQWVEVAAIYFLGLAGHFGLGNTNSLATIDVAGAFIGISSHSAVLSGILMFVITYASPSLSYLSMVMYIDMKDITFISSTHEYEWSIFLEKMIALPCLLPLLFNSLVLTSFTIILLLMRNHLFVWSVFSPKYLYVCAATVCVYIGVFIVAATGLYTYSVFCFRTRNPQEKCPQVLRDNVISHSQTNAHIISAFENSTYPVQEMCLESGCSKVKLETIMRRREVGNSESHPIVDQEHTKPLDNHQYRHRRRSGDITWIKISDHSWWPSQVVDEESVANKPKKKAKDEVRKRENISTRELLKKTLEQDISKMKSAGKSKRKLREPKDNDAAKASNHKKQKQDSRGGNQEETVSASPVITSGKSEAKKVYTRSEALRQREAKLEVTRESTKMENAKPEPRKENIMNQCTRREQNATKLSRKASVKNISGQDGLRRSSRINAKEQTSERVASETSPPGDAGGRQDKMGKANETKKAQNGMAGHNNQKRVGFGHPESEVSEEEIRAMVRDVIFRERTSKQSVNGKPNAKEEIGNGASEVGNAKGEGFGKKEREHAEKQVNIVSSAETLTGLVRKEGPEVGTVKKDSFRKQKIEDAEDRVTKKIVAKTSREKEVKQLDTEEKESNNATNCTLRTKMFDKDNSTTLKPNEIKEQETAKGRDKASKHESRRQGNAGIPTVTEDVGEACKAKKIGKVDPNKSKQLDFNGQVVENGSCRASKYKGSGEKETRKQGEAKSGACQGKPFNQNDSKQKENSNIKMHQSTVTTTPIKHAVTEERGHLSARQLRVMQRLGLVAPPEMLRRWKWTAEIRSRRTRDRPHRINGHLATLRSLLPSATRMDKAALLGEVVRHVRELREKVDDVAVGVVVPGEGDEIGVDQVGGPGDDEGKRVRAWVCCADRPGLMGDLNRAVGSVRAKAVRAEMATVGGRTRSVLEVEMLGVAGGAGPVRSALQMALRSVLLNRDAGPVEIYKRPRLSSRFSKA

>Aco001331; AcbHLH18

MDVDFFNSSPEAQMEFMDMMEKVTSLCDQPIRDFPELPATPLPHSPPPVNLCGPQTAPPPFRCGAAQEQFATQPMSSAAAMREMIFRIAAMQPIHIDPESVKPPKRRNVRISKDPQSVAARLRRERISERIRILQRLVPGGTKMDTASMLDEAIHYVKFLKTQVQSMERAAAVAAQSGGTARALSAAASSAPVAFSVDGSCYPQWVLDQGFVSTNRSC

>Aco012193; AcbHLH19

MDDMKGEYDLYWETKRFLESEELESMWGIEQAISGSYASSSSSVDVVAPSSAAKNIMVERDRRRKLNERLYALRSAVPNITKELQEQERRMQEEISKLESEKTSIRDVVPIIERDDLLVPQRKKRTTQSSSSLALGSSGSSSLEVTQLRVSKVGERASIVEITCNKRRGSMVKLCEVFHSLNLKIITANITCVPGSISHILLVESEGMENAQMREKVAAAVAEVDAPRSLMSNISLQ

>Aco016928; AcbHLH20

MQPCSREMQAIAASLSAIGIQPLLGGVSGGDEFLEQMLAAAPSAAWCDHMAEDPSPPPPPPVATRYTPYDPIGGAAAAATAAPASSAAELGLFAPAIGDGGCEIDPSSSFKSLNPTGGEGVYAGGFGGFLSVPAAQRTSMSFGITPTNAEATVTTTAPKQRVRARRGQATDPHSIAERLRRERIAERMKALQELVPNANKTDKASMLDEIIDYVKFLQLQVKVLSVSRLGGAAAAAPLVGDLCSEGSGAGGGGGNETLTAATEKKVAQMMEEDMGTAMQYLQGKGLCLMPISLASAISSATSSPSVAADQ

>Aco011934; AcbHLH21

MAQHEATSEASLSSSTSTSTSTTNWGDAHGNPLSSWTSMGQWLQAHYSPAAAAAAAASDDDYTNVHAGLTMDSSSSSSANADISGETNLWNHVLLSTGLRGPSTQHNHDIGESFPEVIRRKSFAPPETFDPAFDYLKKMDNNWEFASAPPLANSFGKQLNCYHRNTIEPERMTDLSDLVSNWSIAPPIPNIDDHHINAPSVRHFSLGPNISHVKHAIPNSSPSYTRVDLSHYYNDVKGESHYQDLISVDNSVIGLDNKLCGSGMMEEYSCSNARNISDLISFSGNINASEVEFRAANSCSKSSDPSAGNSTSLTRASSRSSGTSDAKKKRSEESSEALLKKSKRESSTASSNKLQITKAKLGDKITALQQIVSPFGKTDTASVLLEAVNYIRFLQEQVQLLSNPYMRSSSSKDHNAWGGGLERKDKVDAQLDLRSRGLCLVPVSCTSHVYRDITGPDYWNQPYRSCLYR

>Aco011985; AcbHLH22

LNCFGGPQFAPEPNFDCLSEVCSPFAAAGLCGSSAPPAAAAAAAAAAHAAIHEEMSCGGASGGGRNICKAEQKQSAGTPEPLMLAASSCLERKNARPKKADGLPSKNLMAERRRRKRLNDRLSMLRSVVPKISKMDRTSILGDTIDYIKELMARIKALQEEAEAESDPRNRPNQLGALKDQLNPSETTLMRSSTKFDVERREGDTRVEVCCAAKPGLLLSMVSTLDALGLEIHQCVASCFSDFGMQASCLEDKEQTELISSEEIKQALFRNAGYGGRSL

>Aco012028; AcbHLH23

MCREMAAAPPTKDESPEEHFRKQLAATVRQIQWSYAIFWSISTGQQGVLSWMDGYYNGDIKTRKTTQPMEFKADLLGLERSEQLRELYDSLSAGDSNQQNKRPSASLSPEDLTDTEWYYLVCMSFTFGQAQGLNPSYCTGNYNRLPGRALASNQHLWLSNAQYAESKIFSRSLLAKTVVCIPFMNGVLELGTTELVLEDPSLIQQVTTSFWDLPYPTCSEQSRSASPKGEKEEVNLCSNLHRSNINAIGEEPHLLEVGSPSFPFPLHNYENENEELHNKLDEVHVNLYEELNIDSTDDSLRVEVHNSTSQVHGRQLMDDEFSNGLHGSLNSSDCISQSFLTPQRIKNPTLFTVQEGELNKLTDLEDDASHYARTLVAILRNSKQSAPISCPMNGSHNSSFSIWMRGFNAHKTFSSTPQKLLKKVLIDTAWIPGAQPTKPIEENGPQNKVWKSQGDDAGVSHVLSERRRREKLNEKFLILRNLVPSISKVDKASILGDTIEYLRELEQRVEELESCRELLDHESRTRRKYPDIAERTSDNYCNKKIPNGQVSASKRKASDINETDVEHHWILSKDGPIDVNVTVIEKEVFLEIRCPWRECLLLEIVEVLSNLHLDPLSMQSSTADGVLALTIKAKFVGSVMASPGMIKQSLQRVASKC

>Aco012816; AcbHLH24

MSQCVPRWELDVDPPPPPPPLLNSPPTTTPPQQLPPLVPTAGTSERYEVAEITWENGHLSLQGHGLPRVAKPLPKFPTSAASPWNKHHHHGGGGGHGTLEAVVDQATRPQLQEVGPSVDLLAWISGSHPSPSAAAAADLLVPCGEAVAGGRKRGRGSAATASQGSAAPGRGESALAAFDTSSGGAGGDEVCFTPTTTSPTTTTTNTNNNTSASLGSPGTENTSFGRGGGCYDSVCHSRRPPQKDGVCNEEEKAAKREAGRSSSSAKKSRAAAVHNQSERKRRDRINQRMNTLQKLVPNSSKTDKASMLDEVIEYVKQLQAQAQMMNRMSGMLMPMTMPLPLQMSMIGNMANMAQMAQMGMGMGMMDLSSLGRTGPMGLPSLPPLLHPSPFLPIMSPSWDGSGDRLQQPSGPIVSPDPFSAFLACQTAQPMSMDAYNRMAALYQQLYQQNTQSNSKP

>Aco012937; AcbHLH25

MTLEALPSNELPAFLIYDTINAAAAAAAASSSRLLFNGNGGITEESPLIGGMAAMAEEVAVAAGEGRRKRRRRARSGKSKDDAESQRMTHIAVERNRRRLMNEHLAVLRSLTPDSYVQKGDQASIVGGAIDFVKELEQLLQSLEAQKRTLLQQQQQQQQKGNSDIFPTACYENDSPPFAQFFSYPQYAWCHPARDYPSLEIQHRPATVAPSVADIEVSLIETHANIRILTARQPGQLLKMVAGIQSLRLTILHLNVTVLDAIVLYSLSVKVEEGCDVASVDDIAAAVHHILSLIKAGTNLVDR

>Aco017264; AcbHLH26

MARCVKNLDSDLALHDKHGGWNSIKTAKMFDENGYTDPSHDPNLVPNSSFHSSSQEPLPDNGLGGVKECTKVPFFPIEEFSSQNHYPSLEGTSIAAGLDHLENQLGFDIEQELHSHIIHGTPSMESATWEPAMQDIQDPAIYLQQNYENQLPIPVEQNIQSSDGLLYPNSYAVTTDLLSFMKLPRCTTNPDYPPMSGFPFEAYNELSGATIQDGGASMLYDPALHSVVCPTTRSHLLKDLLHSLPQNYGMLYGDEGDAVIGVGEGIGGNIFQPIDGRKISSAILDCRREIGGLAKVDGKQNFAAEKQRREQLNEKYAALKSLVPNPTKSDRASIVGDAIEYINELNRTIKELKILVEQKRHGNERRKMLKMVNDVNSGDMESSSMSPFRGDQDNPLNGALRSSWIQRRSKDCFVDVRIIDDEVNIKITRKNKANCLLYVAKVLDELQLELIHSTGATIGDHNIFMFNTKIFEGSSVYAGAVAKKIIEAMEKQYPTITFPTSF

>Aco024469; AcbHLH27

MDQWKSTESLFPADHSSAAEIAGEVSRRRSGDAGPDRSQSPCPNPSQRVEYEARSSISARKVQKADREKLRRDRLNEQFQELGKALDPDRPKNDKATILTDTIQMLKDLTARVNRLKAECASLTEESRELTQEKNELRDEKATLKSEVDNLNTQYQQRIRVLYPWAAMDPSIMSLSPAYPFPISVPIPSAPLAIHSSLPPFPFFRNPSSGPIPNPCSTFMPYSQLCHAQVDQLSSQQSQFPHPSSSRSRPAGQQDSRNKSSTHQQPSCGGERSDNHSDVATELELKTPGSVGPSHSKMANDKDLSSEMRKGKQRQPEKKESGFAAEGSSSSRSSSSSGGVQQSSSNSVEDGSVADK

>Aco027896; AcbHLH28

MRATAGTSVHVVKANKVEPFYETAASGSCQFPALLPLFGAAAPMERLQGPINPFLGGEEMNSECLETGVANSNSAVEMSYLSLISSHHHRHHHHHRQTPFLQLLLRGAMGVETAGEDEEEEEEEEEEEEEEEERLHSQSLLPSYPSESQFQLLQLQSNCGKRDRTAEIDVFSAAAAPVPVEQLESCISHASESQSDVRRLGHDHNEKKVEIIMATAAAPRAKKKRKRARASGSAKKPEEVETQRLTHIAVERNRRRLMNDHLAALRSLIPPSFVQRGDQASIIGGAIDFVKELEQLLLSLRAQKRIRTSAASSFRCTASDEAHDPTQEGAVLDGFFFSPQYTTYSQQQRRRLQQEGEEKMAAAVFGVDVEAKVVQGHVSLKVAGPRRAGQLARAIAAVEELRLSVLHLNIASLDESSVLYSLNLKMEEDCRLGSADEVAAAVHQIFTCINAGC

>Aco025839; AcbHLH29

MSHIAVERNRRRQMNEHLKVLRSLTPSFYIKRGDQASIIGGAIEFIKELHLVLHSLEAKKKRKSLSPSPTPSPRSLLHFNPSPPITNNSSHDSENIVKQLGACCNSPVADVEAKISGSNVLLRTLSKRVPGHVVKIITLLEKLDFEILHLNISSMEDTVLYSFVIKVRT

>Aco002151; AcbHLH30

MLLHPRSAFSTYARVGSHDIGSLAAGRHVGSAAAVNVHRRLFGFIRRIGDGGVAAAGVRAAAEAEDGVAGVGRSRGFQHMMRERWRRERMSQGYADLYAMLSSSSKGDKISVAKAAAARVRELKYQKEQLQRRNAELAVVVAAQRPATAAVAINLTIANCSTPMAMDALTAALQCLKLMELKITAVRSSFSGKNLSVIVGAETKVRSHVIFVHVRLIGYVHACLK

>Aco002225; AcbHLH31

MEENSRSFSDLLAKSNQMKPIRPDNELVELLWHNGQIVLHNQTHRKSPPPCIDFRQSQNPKSVLKSEITNVNANNLAQEDETMSWFQYPIDDPLERDFYTEFFELPNGGFNDNSLGKEKCAETENESNAVNLGSGVQVGGGGECSSIMTIGSSICGSNQVPTQVEGSNLHHLNTAKLPIEGNNYNSSTHEATATSSSGGSGCSIGITQQQSISNQGTKRKERSTEESESQSEEAEQESIEANEPTKQSASRRSRAAEVHNLSERVRLTDDPEIKPACLIPQSPYSIANSFLMQRRRDRINKKMKALQELIPNCNKTDKASMLDEAIGYLKSLQLQVQMMWMGSGMAQMMFPGVHQFMSHATMGMNPASMPSMHYPVQMPTVPFCSSESFPNQMQNINFPGLNSMQVTNFCVNGACSLQSNQISVLPSYNLSHAIGEFPTENVRDDKSKYSEDPLKVKVFCWLLLKKRILTADVLVRRGWTGNTVYVLCGGEEETTDHLFSRCVYFRFLLVMTLDDTEIAGLGLGVHPLLDSLAARSSMPPKARLDVLVAAWWVTWEIRN

>Aco002246; AcbHLH32
MQSELKGMDDSNISHQWTMNSFNQVIPSHQIVAASGESSQPSLSQESFSSCPNSFYTTRNDVSSSNSILTHSPNSLFGWSNTFGRPSTQPKEEVEFVISYNNSEQNYEAIGSKQRSNMVKFGAASPQAQDHIVAERKRREKLNQRFIELSAAIPGLKKMDKASILEDAVKYVKELSEKVKTLEDQSPKTIESVVLRKKSCRSCDQDGSSSYNNHDSKRCLSEKPLPEIDARIHEKNILVRIHCENLKGVLVKVLSEIEELHLSVTNTSLMPFQGGSIIITVIAQASSSQLNST

>Aco002333; AcbHLH33
MLALSPPLFSSPLENNVMSNELRSDDRIFASNRIQHEDGMLQQSLLFPSPPHPELEFDDQSRASNSAKDSPRSHKKLSHNAYERDRRKKLNSFYSSLRTLLPESDRTQKKMSIPSTVSRIVKYIPELQSQVARLSRRKEEVLAEIAKRKEGSPTKEGIEFLINVSATCLNSEEVMVHITVLNKNISLPLSKFLKVLEGEGLQLMNASTLTSFGDKTFYNLHFQIKRCTGMEGQIFCEHLVKVIKEKGRDDYSSIQ

>Aco011121; AcbHLH34
MVSREHKKAGLHEKLQLLRSLTNSHALNKASIILDATKYIEELKEKVVKLNQEVACAQNIVIEDPLPMVTVETLERGFLINVFSDKSSPGLLVSILEAFEELGLEVMEARASCTDTFRLEAVGAENLAEGVDAHVVKQAVLQSIKNCSGN

>Aco021988; AcbHLH35
MYHHHPELYPPHEHIPMEGSSSEFIGPSHTATMNFSDLALMGSGQNSSSNSSNGTSAAAAELDFHRQFGLDVVGKPEMAPHLMHFPENLFFAEIPPETASVGSTVLYDPSVQLDLVHHQPCQVREIYTANNINNNDDVSLPQDYSGFIHGSSVNGHFVGGVMEEVYGLGQQLESPHLNTRRQKGGMRQKGLSFNGVEKKEKQRRERLSEKYELLKSLIPNRTKDDRATIISDTMDYIRELGRTVNELKLLVEKKRRKKERGKEVLIGEELVGDMESSSVKPFIDEGEHHASNGSLRSSWLQRKSKETFVDVRIVEDEVTIKITQRKRMISCLLTASRILDELQLELLHLSGGIIGDCHIYMFNTKIPEGSSVYASAVAKKLIEVMDVQFPPQTC

>Aco022096; AcbHLH36
MTNNSSSDRSSFMPDNDFVELLWEDGQIVMQGQSSRPKRSFVPTPFNPSYNNRFQDKDIKNEILPKQLNHFESGDPTVRHDFFTSDPLNDKDDDDDDSVPWINYPMVDDSLSNDFCSEFLAEFSGLNPNNVSISHNTDQIVRGSSSMENNHAPSKAVIGGSGGFTSRSGQLLESSQQHLNSGHAVKSKATDFGTASSIVTHSRMPQPSGTSPLLNFSHFSRPVSLAKANLESMERLRSNEKAFTAPTSSNPPESTLIQSTGGFKELRPSSANTPKEMVSLEHTQALCQQEMPRKNNNHTAVANSNCINQQGSGFAPQKGPETIVASSSVCSGNSAGAASDDPKHGLKRKDCEGEDSGNHSEDVEDESVGLKRPANVRATKAKRTRAAEVHNLSERRRRDRINEKMRALQELIPNCNKVDKASMLDEAIEYLKTLQLQVQMMSMGGSLCMPPMMLPPGMQQMHLPSMAPHYAQMGVGMGLNVRLGYGIGPLDMSSSPNCPLIPVPPIHCGTQFPCPTVPGAQVRPAMAGPGNLPMFAIPGLGAIPSAVPRIVPQLGSFSGLAVMANPVPVPASDTTTPLNCKEHREHSMNLERKKSSSDSQKTPFAPSALAQSNQTLHSSGSGGANSDCSLNFEWPYDLHEHDGLGFDFCAICAKAVQAEDFGMLG

>Aco023468; AcbHLH37
MADDLHTAGICSGRGSWWMNSARSSAGLDGQIAISSTSIADIDARGSTFSWSAGESKSRSCEESTGSASAGSSITFQDTHLNPSMNWNETLLGGKADTRIQAMHQDDLSKRPYWHDGLLIDSYRAHVGVEESSTNLFKEINHGKNFLLDKHLLNSSNEASASFGSPSRLLQTFITSEANPPLSIYGNPYLHHQSSTATASHESLSELLQPSWTKFSQLVKPSPLKNQLQFSNSTPFWNPSSTAMNEVSSGFYPSVPSQFISQQKPSYSNLIVKEKSLLNSEQVQDSCSSAKKKSSSGDDHQPTFKKPRIETPSPLPTFKVRKEKLGDRITALQQLVSPFGKTDTASVLHEAFEYIKFLHEQVSVLSTPYMKNEDLTQHQQGLERLNDGEGPKQDLRSRGLCLVPITSTYPVATETVAEFWHPTFGGSFR

>Aco023519; AcbHLH38
MYHHHPELYPPHEHIPMEGSSSEFIGPSHTATMNFSDLALMGSGQNSSSNSSNGTSAAAAELDFHRQFGLDVVGKPEMAPHLMHFPESLFFAEIPPETASVGSTVLYDPSVQLDLVHHQPCQVREIYTANNINNNDDVSLPQDYSGFINGSSVNGHFVGGVMEEVYGLGQQLESPHLNTRRQKGGMRQKGLSFNGVEKKEKQRRERLSEKYELLKSLIPNRTKDDRATIISDTMDYIRELGRTVNELKLLVEKKRRKKERGKEVLIGEELVGDMESSSVKPFIDEGEHHASNGSLRSSWLQRKSKETFVDVRIVEDEVTIKITQRKRMISCLLTASRILDELQLELLHLSGGIIGDCHIYMFNTKIPEGSSVYASAVAKKLIEVMDVQFPPQTC

>Aco004377; AcbHLH39
MRDNISDLFEDPPEFRSADFAGAASPDDLFSLLETLEDSRKEVPPFNPFEKAAASAVPQKPLLSSVRRHETTDDAEEANDPARKKHKASAAEAAAQESEAAVAAAQDGGQHKMSHITVERNRRKQMNEHLTVLRSLMPCFYVKRGDQASIIGGVVDYIKELQQVLQSLEAKKQRKVYSEVLSPRPAISSPRPSVSPRPLPPPLSPRVGLPISPRTPQPGSPYKPRIVQQGLYLPATMMLPSMESSPSSEHTLADLAANSKSPVAEVEVKFTGPNVLLKTVSHRIPGQAVKIIAALESLALEVLHVNISTIDDTMLNSFTIKIGIECELSAEELVQEIQQTFS

>Aco004559; AcbHLH40
MNCGDQLPQFFLNLNCDHDLLGQFGAEISSQHPYIGSISPNLNPSPPPPLTMTHLDQLFPDPGFAERAARLSSFDARNYRAFASRFGLPEIGKAPIDAEESSVSDPPSAECFHSPSESNPKKRKAAAAKSRGKEAPLVNSPKSSLNVLEEKGLERKRCRLGESDQKIEEEDSIKHKEAQNGAQKQGKEKNNAKPAEPPKDYIHVRARRGQATDSHSLAERVRREKISQRMKLLQDLVPGCNKVTGKAVMLDEIINYVQSLQRQVEFLSMKLATVNPQLDFTNLPNLLSKDMHQNTGPFLNPVFPLETSGAAFSYTDHPHQEGNILHSMNQLDPSLSQSMSPHQLLNGIGNASLQVGNMWEDDLQSVVQMDIAQNQDVVVSSQSFHAVELQCNKLVQLKKGGANITNTPFKMLKREKKSARDVEL

>Aco004647; AcbHLH41
MGFFSFQHQNPFLLDSPIYDYPSHDETFKMQFLPAEQAEDMGNNASSSIECGSSSIGASTPLIPSSPSSSLVAVPHPCERAAIIKPQDKKRKSRCGSSLSSSQSKESKESSKARKVRNEENKAKNGVKSEAKSSEEPPKGYIHVRARRGQATDSHSLAERVRRERISERMRMLQGLVPGCDKVTGKALMLDEIINYVQSLQNQVEFLSMKLASMSPILYGLGSDFDGLVDQAQGCSPNFEKYSESMSVTIPCVHPETSPLQPIAFEGALGDYPMTSNSTSLSLQNQGPISFSQVTLGEVSVTRTLEIFSCKWVTKDRAFSTKWCSMTCARFSRERKKRTSFNMQEALSIYVRTRAETVNVQWKTK

>Aco004686; AcbHLH42
MMNRFEPDGRKKPSPMGPPAGLARFGSAPGSLLAGIADSVIGGAGGGGRAALVRHSSSPAGLLSHLGVDHGPRGIGSYSQTGTEFVQAMMASQKLRPQWSFSRQDSLSQIAEMSIPDIGESSNDFSITSWDDTNSFMFSAPSSKRAKDNNAEIITSMSNLDSQIGLQGTSLEISGMENYLQIQEDSVACKLRAKRGCATHPRSIAERERRIRISKRLRKLQDLVPNMDKQTSTSDMLDLAVQYIKDLQSQVQKLNEEQANCTCSSKV

>Aco012714; AcbHLH43
MAFAKEQAPKESTRPHSIYDTISLELFGYKGHRHTSSLLNEVSYCEGLPPILADTSNSSSSASFALGVLNSPPQEAHSGLSSSKTKSDSCWAYSTSSVLSFDLGDQFPHSSYANHNDQEEECDVWINAMDQNHTMNQSSFEYSWIVHDHSYETRSAAKDDAYGEEQFALLHPSSISMNGAQEVSRQDKLSQKRPYAGDHEMPTPKKQRGINRNTKTKPTPPKDPQSVAAKVRRERISERLKILQDLVPNGTKVDLVTMLEKAINYVKFLQLQVKVLSTDEFWPAQGGKVPDISQVREAIDAILCSQRDMSSNSKK

>Aco015158; AcbHLH44
MESSHGTSTKLERKTVEKKRREQMKALYAKLDSLLPSSTSSREGATLPRPDRLSEAANYIKGMQEKLERMKERKRQLMTRNEAADNSKLPKIEVQNRGSNLYMIQIVSSPDDRAMFYEAVRAVEEEGGEVLNAQFSTTDRKAFHTIYALVGDFKYRFETGKLKERLKDLGWQGRRV

>Aco019347; AcbHLH45
MDNITTTNTNSSSSWDLEMSMGSHPFLLFDYHQPPFQLVHPSSEAILPLIHAHNIPSPCFSTPPPHIEAAPAAAAAAADHHRDQEEEEEEEEEEEEDREGGEGEREEEVYKIAAMQPVDIDPSTIKKPRRRNVRISDDPQSVAARHRRERISERIRILQRLVPGGTKMDTASMLDEAIRYVKFLKRQVQELQSNPPPPPPPPPPIASAPHRATFLGAVPLSAPVDWAVHGASSSSSSSHQGLGFGFIAPKQPWDALN

>Aco027360; AcbHLH46
MESSHGTSTKLERKTVEKKRREQMKALYAKLDSLLPSSTSSREGATLPRPDRLSEAANYIKGMQEKLERMKERKRQLMTRNEAADNSKLPKIEVQNRGSNLYMIQIVSSPDDRAMFYEAVRAVEEEGGEVLNAQFSTTDRKAFHTIYALVGDFKYRFETGKLKERLKDLGWQGRRV

>Aco002862; AcbHLH47
MSSRRSTRITEEEISELISKLQSLLPEGRRRGNSRASATKLLKETCNYIKSLHREVDDLSDRLSDLMAGMDNDSAQAEIIRSLLRS

>Aco003064; AcbHLH48
MELRGKKTTHDFLSLHTVDPSFKRHDTSPSSSQGFFIKTRDFLQPLDDREEESAGRDPAVGPTEEERTLPGGIGAFSISHVSGPGSRAPLKPDRGTCGAVRGFGVKSKPEPDIDSGVYTTQNVGVLGAPFSTWAHSTARDSGPSSRGPWPSPFAAARANGFVGSLSFASRDNAATEGKRSMEAASRSSRGAFDDDDDDDGDEFGRREASSSRKELSIKVDGKGSGSGSGTDQRPNTPRSKHSATEQRRRSKINDRRVYVGCITLLFWLLICFAMLRFQILRELIPQGDQKRDKASFLLEVIEYIRFLQEKVQKYEATNPEWNQESSNLLPWVNVYFRSFWKSAQSNNHGLSDPLQAIKNGSSPSGYMFSGKYTDNGTQVAPAVLTGVAQNRTETEMMTAGFSSKAMETPQNFINESMSGAQAPWLRPHGAADSAVNGEMLSEQEELAIDQATVNFSNAYSQGVLTTLTQTLQNSGIDLSQANLSVQINLARRSKRTNANVTACTTKDQEDPACSNQETGCREDVTQAPKRHKTDYS

>Aco003149; AcbHLH49
MYGSPQGAGTRKDLSGPFPPASASEDSDLLLLHRHHEQQQQMTSSGLLRYKSAPGALLGEIGDDFFNPAAESVLSDFWDNSSNRPGHSRGGGGGGCRTYNPVGFPNNNNSLDQNRSGPSPSDLQAYQLVFSLSLNQSSFVRDLNGWFLHYLILQDQNVGVQKHQDFELAHQLSLPLTSSELAAAVDKFGRQFQDAVPCRIRAKRGCATHPRSIAERVRRTRISERMRKLQELVPNMDKQTSTAEMLDLAVDYIKDLEEKVKYVTAQRFSPRFRFAITQTPKRTSDGGFGLEPSGSFYVDLEADWRGCRGVKSPLGFLGNK

>Aco021068; AcbHLH50
MDNFIDPFLSSSSWVGNATSLEAYAGNERNSSEFMTPSSHIVVNGSAEDLKIHEHNRTSSIFTNGNIKYELDKSLFSGGVESNSTPVSFGLELDMPHSGVAISSSIESNGSEFSAFPQSLTDAHSISSPSVMWPSSYVNISSLILQRKPQTFGLQELENDNILRNTCDENGKFSQLENLPLRFIHDKNEFHSSHLPSFTAGPQVQFTNGGSQTQEQEQNGVHKLHMPSFASGPQMTFSNTGLAQNSQQLTQSIEGNTSKLHINHSSNSQSQLAPVNENGCNGAVKPRVRARRGQATDPHSIAERLRREKIAERMKNLQELVPNSNKTDKASMLDEIIDYVKFLQLQVKVLSMSRLGATGAVVPLLTDSQAECSGNLLISSPSISATGPDFADSQDTSSLEQEVVKLMESNVTNAMQYLQNKGLCLMPIALAAAISNQKGSNSSAIPPERRKPSLVHPETNNAAEDRANGFDDGDMRKEEVAKSVNNGRELQSKA

>Aco004914; AcbHLH51
MSYEMEPTKVVSEINWSSYSPSMQSESEIMAQLLAPFPFQCEPDNPDLGFGAPSFFWSGHATESYYCSENSNPNVYYLSQGEVSLSGSTSNSSFILPSSVYESCYVNGSNVALGINTCSEPIDLNPLYGEEFIDLSEQTTQPKRKFPSVEEENFVDHGEFDSTAVSTKKKARDSEKVQSAKKGEVKRTANSIQSGDDEALHGQSCSSYGSDNDSNASQEMNGGGSTSSCSKGSSVLNLSGKTRANRGSATDPQSLYARKRRERINERLRILQNLVPNGTKVDISTMLEEAVNYVKFLQLQIKLLSSDELWMYAPIAYNGMNIGIDLKMSPSQQ

>Aco014442; AcbHLH52
MADDLDARICRGGSWWFNPGPSSSGGGGGFVDAPGSVSCSTAIADAPRGSALPSDHVGSSFADSFSLSAHFMDWGHDQLRSSGKAENSFHAMLQEDMSTRPYNFRQGSIEPELEPSMSQNFLLDHHQHHPSSTNDPAPDCSVITSYALAPTAYGCPPVMHLQTLLEADAKPQQSVYYDQPTETPMATAFHGTNWPVLKDQEGSSNPLHFSNNTPFWNASATAVNEAKSGFYPNSVPSQFVMPAFEPKLPSCSNFMVKSNRDASMPKKSGSETALKKPRVEAPSPLPTFKVRKEKLGDRITALQQLVSPFGKTDTASVLHEAIEYIKFLHNQVGLLSTPYLKNGQPMQQQQSAEKSKEGDESKKDLRSRGLCLVPVPSTYPMASEATVEFWHPTFGGTFR

>Aco014447; AcbHLH53
MPRRPRGAAASQAAEAEAEAEAELEAELQGDFVDSVLDMEGGIGGETPMEKWEMRFKSKNLEAERRRRGKLNTNILALRAIVPKITKMSKESTLTDAIDYITLLQKQVLDLQTELLETDGDLIHKNEANEDEEGEKQRSPSSETVAPSVTVQCQGQVELIPMGPNKYQLKIMCKNRMGQFTKVLEALSCFNAEVAEISSVAFFGFSKSVFSVEVKEGEEGEIIELRNLLLALVGASEN

>Aco014454; AcbHLH54
MGSSENANWVFDCPLLDDVAVAGDDFPASGSGFYWGSQGINGSSNGGVEISGSFLDSECVKERGSNKRVRSESCQPSSKACREKMRRDKLNDKFLELGSILDPGKPPKMDKAAILSDAVRMVTELRSEARKLKESNESLQEKIKELKAEKNELRDEKQRLKAEKESLEQQIKIINSRPSYMPHPPVMPAAFAAPGQSAGHKLVMPLIGYPGYPMWQFMPPADVDTSQDAESCPPVA

>Aco020380; AcbHLH55
MWQPLPMIGVPVAGPGAERGAGTLFAAAAGGVRNRDHSVDESSVTESSGGSRGAARRRRRDSAAAEGESPKLASATSGGGNGLTNSEGKRLKGVNSVDNNVNVKGAAEASSGMANKQMDQIAPPPEPPKQDYIHVRARRGQATDSHSLAERARREKISERMKTLQDLVPGCNKVIGKASVLDEIINYIQALQCQVEFLSMKLEAVNSSMDTGIEAFPPKDFGPQTFNASSSLPFSLETPREYGQGSATEWLHMQVGGALDRVT

>Aco020381; AcbHLH56
MSELLFCSEGTARLNCFGGPQFSPEPNFDCLSEVCSPFAAAGLCGSSAPPAAAAAAAAAAHAAIHEEMSCGGASGGGRNICKAEQKQSAGTPEPLMLAASSCLERKNARPKKADGLPSKNLMAERRRRKRLNDRLSMLRSVVPKISKMDRTSILGDTIDYIKELMARIKALQEEAEAESDPRNRPNQLGALKDQLNPSETTLMRSSTKFDVERREGDTHVEVCCAAKPGLLLSMVSTLDALGLEIHQCVASCFSDFGMQASCLE

>Aco025287; AcbHLH57
MWQPLPMVGVPVAGPGAERGAGTLFAAAAAAAGVVGNRDHSVDESSVTESSGGSRGAARRRRRDSAAAEGESPKLASATSGGGNGLTNSEGKRLKGVKSVDDNVNVKGAAEASSGMANKQMDQIAPPPEPPKQDYIHVRARRGQATDSHSLAERARREKISERMKTLQDLVPGCNKVIGKASVLDEIINYIQALQCQVEFLSMKLEAVNSSMDTGIEAFPPKDFGPQTFNASSSLPFSLETPREYGQGSATEWLHMQVGGALDRVT

>Aco011852; AcbHLH58
MWHVGRSNSYSSSSCDDVGVGASASTPTTTLVNAAPNGGRSSLSTDPLSAAAAAVDGLLSEPMEHRLWSQLLLSSGNGGTMHNNHGVGGENFLDLLNSKSLAPELFDPAYESYPKPSKLGNNTYYEFTDTTAAPALNHLEALELSHYNPSMVEPSEIKMTHPSMNLATNWCSTAASPNPRYSGSNLYETHSSLSQARERSGLVLQQHHPSYMEPFNAPIDLNNSLMELNNKLNYSGVAELPWTSNRNFSDFIAFNGCLKKQEVGVKASKHCMMNSSESNERKKQAYEVTSIRGDGRNSGAASVGKKRKSEESSEALLKKPKNESSSAAASLRVQVPKVKMAEKITALQQIVSPFGKTDTASVLQETIVYIKFLHEQVQLLSDPYIKSSSCKDQNLWGVDQRKEKEEAKLELRSRGLCLVPLSCTSQVYRDNNGPDYWTLPYRSCLYR

>Aco011855; AcbHLH59
MALSYQSSWETLAHLNPEIGGAFHEPQSEVADALLGFFCDPIDASTFPIDSLFDSSPESYFYAETETETVPLPSLSHSSLSAPSILALTPDLYPPCDKFDLYRCPKRPRSCGDLFRPSNLVFEQPGSYIRHIAAGAMMGGCRSSDFLSEFAAAPPLAVGMQERKAGSGCLSAQSAAARERRKRISEKTQELGKLIPGGNKMNTAEMFQSAYKYVSFLQAQVGILSLMGSIQERGKVPLLVEQQLQLLLESTTIQEKLYGEGNCLVPNKIVDTMAKDKEIKSNMLVSRDLDRFIESMR

>Aco018875; AcbHLH60
MMEAFMATSDLQGFPWGSSAAAFSAPPIPPAAAPPPSPPQPFFNQEKLQQRLQALIDGARESWTYAIFWQSSVDVTTGASLLGWGDGYYKGCDDDKRKRRGPTAASAAEQEHRKRVLRELNSLISGVGGGGGGGPDDVVEEEVTDTEWFFLVSMTQSFVNGAGLPGQALFSGAPMWIAGADRLAVAQCERARQSQVFGLHTMACVPVGSGVLELGSTDLIYHSSEVMNKIRILFNFSSSLDVPSAAASWIGATSAAAGAAPPQVAAAPDQGETDPSHQHQHQQQHQHPHQSFFTRELNFSEFALNGSSSQSFKPESGEVLNFGDNAPPSSSTAAGAAPVKSGDSDHSDLDASVREVESSHVAEPEKPRPRKRGRKPANGREEPLNHVEAERQRREKLNQRFYALRAVVPNVSKMDKASLLGDAISYINELRSKLQSLESDKDTLQSHVESLQKERDARPALTPASAPPPPPPPSNGVSDAHGRCHGAEIDVKILGHEAMIRVQCHKSNHPAARLMMALKDLDLEVYYASVSVVKDLMIQQATVKMSSRVYSQEQLNAALCARLTDPSPCR

>Aco026878; AcbHLH61
MSSTLGGLCSSELPSFFIYDTINATQLHYTNPSESAAVLGPAPPEAAAAAAVEQQQMRVQGGKRKRRRRPRSGGSKEEVESQRMTHIAVERNRRRLMNDHLAVLRSLMPDSYIQREEVESQRMTHIAVERNRRRLMNDHLAVLRSLMPDSYIQRGDQLNVGGAIDYVKEHGAYSSSLDALQKEDHYCCSTRSSKHPLTHNDGSCAISPPFRPSFFACTPSIPCCATGLAKDYPFPRESDPPWPDIEVTS

>Aco008553; AcbHLH62
MSREEMELTANWSSYNGSSTPAEESEIVAQFISTYPFQNEQDHRDLGFGAPPMYWPDHHNASNSYYCNGNANPNLHYWSQGDSNSSSVSTSTGTCCYFVPHSDYESYYVNTNSSSPMIFNLVEEQRKNRSLQVVPNPSLREQTGVNAETSSDDHGDSSMNIGLSDQITHPKRKFLSNRNDKVVDQSKFENPIESTKKKSKASTKVQKCAKKMQSKRAQKNVKSGDEEECNAAVNGQISSSCYSSDNESINSQEMNGGGSASSSSKGSPALNLNAKTRAGRGSATDPQSLYARKRRERINERLRILQNLVPNGTKVDISTMLEEAVQYVKFLQLQIKLLSSDELWMYAPIAYNGINIGLDLKISPPQ

>Aco008686; AcbHLH63
MEWNKYQMNQFVPNWNMDDESRSLRDLVAAPDRKEPIGADNELIELLWQNGHVVMQSQTHRKPPALAPEFKQLRKHDEPMLKSAATVAATSSLTRDETASWFQYPLEDSIDRDFYSEFFFDMPNADEGRPGAENDAVDFSHFAKQWKGGDAGASNPLPGEKRVESGGLGVAGASSSSMMTVASSICVSNQIQVAAKLRQIASGDAGASASRRSSKEEAHTRLASQEAAVASSSGRSGCSFGFGRTEQHNNKRKERGMEEFESLSEDAENESVEANNQAQRSTSTRRSRAAEVHNLSERRRRDRINEKMRALQELIPHCNKSDKASILDEAIEYLKSLQLQVQFLWMGTGMGPMMFRGFHQYMSRVGPASIPSLHSPVPMSRAPFMNNQFMASTSTANAANFSNQMPNVPLGAGLLGLNHLPLQSQAMNCDAYGPKTAQQHQSSVEPISGASPSESTPKDKAGKT

>Aco009100; AcbHLH64
MDRELPQLLISPSLSGPTDSAEWRSMYPVVAETLPLYALDCYGGLLPPPPQLGIAPAPYPPMEWLSSGNVVHDLDVFHGSNPIGSLPSAQDSRLKLKFPKIEPLAIDDRSAIALSAGSAAFHHLPGLRSLEPPPDLVAEAPDPPRFSGDLISLIGPSKKRLPERSFSDPPPPPPPQPPPPPPPPLRSTGFPRPPPSEISRRRRKSISDRTRILRGLMPWERKMDTATLLEEANKYVRFLEAQVTALQTMPERSARFAPPREPPGSLPCPAAALGRLNRQQLLQVLVNSPVVQDRLYARGVCVFSAEQVASLRCSAERHRPPPLLLPPAAAADDDDDVRN

>Aco015791; AcbHLH65
MAQENSEASSSVTLISSSRGTNNNISSSWWDMHGNSSSSSSSLSSCSNTTMNNNRSSSSSSSSSWQQLLHRPDHRHGNPCGHVGIDMASQGPLKLSSEAGESHLWNQVLLNAGSSMNMQNTHGGENFLENLSSKSLSSEMFDPAYDYLKKMDSTWEFNTNAPSLNTLQKELMRSYDGTMLEPERMTNLSNLVINWSIAPPDPQIDHRIAPPSCEVSMDNPSVHSQYLAPSISHIEHEVIPHSQLDSCNGMIDGSTSYKEDSHHQEFGYQIGLNNSILGLNNKLCSGLMTDIPWSNTRSLSDFISFSGCLSKPEVELRGPSNPYGKISDPFEKKKQGLETSSTRVNNRGSRTTSDGKKKRSEDSSETLAKKIKSESPTATSLKLQVPKVKLTDKITALQQIVSPFGKTDTASVLLEAIKYIKFLHEQVQLLSDPYMKPSPTKDNAWGGLDIRKEMAEIKIDSLRSRGLCLVPISCTPQVYKESSGPDYWTPPYRSCSYR

>Aco017396; AcbHLH66
MANHPPPPNGMGDDFFDQIFAMPPYGGGDDAAAALGGGGGGDGHHLAAGMVLHPRPGSAGDGGGGAAPPFPLALSLEQGKAGEGSGSGNPLREDADAKERDPVHLAGLFPPGFGHMQTHQIRPNPPPQMFHGQAKPGGVVAAPQAPAPRPKVRARRGQATDPHSIAERLRRERISERMRALQELVPNTNKTDRAAMLDEILDYVKFLRLQVKVLSMSRLGGAGAVAQLVADIPLSVEGEASESGSKQHIWEKWSTDGTERQVAKLMEEDIGAAMQFLQSKALCIMPISLAVAIYDTQQQHQPESGHSVKPEPNTPS

>Aco026239; AcbHLH67
MDPPSLVPEMWPPPQPHLPLAGMRLGGIMIGRVGPSSSSAAAAAAEASAAAAAGPRDLSVDESSLTEQSGAAGRGRRRRRDTAASEDESSKPVSTSSGNEMTDSEVKRLKVMKSGDENSKGEAALKKPTDQNPLPPEPPKQDYIHVRARRGQATDSHSLAERARRDKISERMKILQDLVPGCNKVIGKASVLDEIINYIQALQRQVEFLSMKLEAVNSRMNSGIEGFPPKDYPSQAYDAPTALAFNPQTPREYGQGSAAEWLHMQVGGAFERISKQSK

>Aco009909; AcbHLH68
MDSNHYTEILYYLSPHPHIAHMDSLFLLSTEARKRFLQSVSRILGCSYICLWSPRSTYLICVDCWFHEDDSAQPSSLSKILFDAYRSSPCSIVTGCVPGMAYKDGSPYIELIGSNLVNSASTQVQQQFYQEAGIKTAIFMGCENGEIELGMTTTTTSNSNMQMNIQQVFSEDFIQQSLLGDQPPPPPSSSSLRSLSMGSPPEFSSFKGSTSSFMPPEAYARLPFPTPAADDAAMAQAMLAVISSSPPPSLLYQPPQREQAPRQRAFKAYNAALRPKTDPVKPGVPGQKMIKMAVSMLRRVHMMRFEARMPEPRPTSNQLHHMISERRRREKINESFHALRMLLPPGSKKDKASVLAKTKEYVNTLKAQISELEEKNRMLESQLPPPTEQMKQVDSGDSSNRVEVQISSGSESTSETRQINLNVIVRVECDTIDVLLRILEFLKGNGNINLVSINARSTQPQSNTYASANLTFQDKVNPPTKYNSQ

>Aco010678; AcbHLH69
FVMGTVPVGVGGVRVTESSSASLVVDDGERVGKKGGVGRGAPEAKTAMALKNHSEAERRRRERINSHLATLRTMVPCSDKMDKAAILAQVINHVKELKSKAVEISKGYNIPSDTDEVRVEAEANAVNSGSFYIRATLCCEDRPELFAELRQTLDTLQLKLIRAEISTLSGRVKNILIMRCDDNANDIDRHIYTASVHQALKSVLDRVNSTVDFYPRAKRRRISMFESSCSSS

>Aco010845; AcbHLH70
MKTEMDDDAGAGAGDCGGGFWSAEDAAMGAAVLGPEAFDYLAAAHAAASAEGLITAASGDADLQNKLVDLVEGPADRPGLGWSSAIFWQISRAKTGDLVLVWGDGYCREPEPGESAPQSARNPNPESHQKLRKRVLQKLHEAFGGSDEDYYALRLDHVTDAEMFFLASMYFSFPCGKGAPGRAFASQKHLWIPDSELKISPPNYCFRGFLACSAGFKTIVIVPFETGVLELGSVRSIAESSDALQMIKAVFLGTVKVPVVVEKKKNEGNGFVGNYGPGERVQGSAKIFGKDLNLGRPASNVGISVSNQPNSKNSSEQHMLFPNVRKGLQGFNWNHARNLNPPQQFGNGIVVASNEVNHHANGIGDSPVLNQFQLQKQSRQIDFSTGATSAAGGNLVARVQGPLEGENADIDALCKEERGSGAIEERRPRKRGRKPANGREEPLNHVEAERQRREKLNQRFYALRAVVPNISKMDKASLLGDAIAYITELQKKLKEMESEKEQMLDSNMTDPREGVNHHQPQVDVQEIQDELIVRVSSPIETHPVGKVFRAFEEAQVNVADSKVAAANGKLVHTIVIKSAGFEQQMKEKLISALSRVMSST

>Aco016398; AcbHLH71
MAQCIPTRASTNPSVLERQRVRLNWQQQQSTDNTDCIGYNNIESFAPLSSFDQLPKNFVDGERSRNESFGHVHDPIESLGDGWPDFSMVNYPNFCAINVGEKANDKENSDSSKKRKFGNFSNSQVDANNATENSGSSKGMKEDYVLGERKGATGNQKPQNKKETSTETLKENGKASRSSTTTTPLKTDYIHVRARRGQATDSHSLAERVRRERISERMRYLQDLVPGCNKITGKAGMLDEIINYVQSLQRQVEFLSMKLAAVNPRLDFTVDSFFNEEMNIACNTGLIQGMNILLPHDQLDPSYIQSNALLHQAQVGPSSSGLDMAMSTTQQMTLQCPMSAPMQLVNTFMDSCYNNVHGSSSLWDQGTVYQTILRRKRERFKILLFEISKSLIAESEDSFRILYNLGLFHFTENGREWSYFRP

>Aco016415; AcbHLH72
MKRSCGSRGSNTTTTNNSSINNCDKVERKTIEKNRRMHMKNLSMNLLSLIPKDHFTTSKDALTQLDHLDVAASYIKKLRGRIEKLKQRRESGPSIEGVREDASEGTMTTATTAAATIGVRLPVVEVRYQDRHLEVVLIISSNSSSSSSSSSSNSQKRRFRFHEVISLLEEEGAEVVNANFSVVGDKVFHTIHSQAVCSRIGLEASRVSERLKKLVF

>Aco016434; AcbHLH73
MALTRERTIKELQYDSIYAPSLDLFGFKSHYSSFLEEGEVSESLSSYSAGLPPILSDPSNSPAPSFVFGMGNSPLQEAHLSTSFKVGSASDHWGYCSSSSVLSFEQGGRLPHDSYLNLDQEEECALWIDAADQNYSINQSHDQSFDISGVNDSQQQGEERFGLLYSSSSSADGVQESANVHNKVPQKRSYVGGDVQVSTSKKQCGANKKSKAKPSPSKDPQSIAAKNRRERISERLKILQDLVPNGTKVDLVTMLEKAISYVKFLQLQVKVLATDEFWPTQGGKAPDLSQVKDAIDAILSSQRDRDSNPRQ

>Aco020569; AcbHLH74
MMDSSNHLDHHHHHYHHHQEGLHGSSLEEAAAPLIHCGGTNHGWNNQPLIWNCGDFNVLNESEVVLSNQREFIRATYQDEPLTPPLSSIMVQDLGFQWCSNADTLMNQPNNQSHLARIKEELPTSDNFPKLNGLIKNPTYIDNYQLSEKLFLKALPQNGHNNALQPPTDDFHFGPSSLANLESGRGGFSMVLPSVNISLPSLPPLPFSGSLDMDLQALLASTKLSRSFFAPPFNSMAPLLKEDPIFYGPPHVQEAVHGPFNNNHKVRKEKLGDRIAALQQLVAPFGKTDTASVLMEAIGYIKFLQEQVKTLSVPYMRPSNNKKLRTTQGASNEEKNEPKLDLRSRGLCLVPLSCTSYVTNENGGMWSPSNYRGSA

>Aco005342; AcbHLH75
MHIEIDLKRYRTVEEVGEIIMNVRIIAYCQLMQVCQGDARILNGKGFLSHEYELGCTQNSALNDHYPYSVPFPPQFLSPLSGLEFQPLENCPKNFIIFDHTYDKGSVMFHPALVRKLGSPNLDLYPSHHAEKKGKISSRKNDICQDKFSSSFEEDSKDIDALLCSDEEGNDGKKDDDDDDDDDDVVSTGRTPDFSCSGKHEPSFSQDFTFDGSISKEKKREKMRKMVRVLREIIPGGDRLDAPAVLDEAVRYLKYLKVEVKKLGLQNFDDQSCD

>Aco005770; AcbHLH76
MDSLGWSNPTPIQHEISTSTCIWSTQYDNFSEPKEYYSNINDDVELHECIYSTGLELQGVRACDTISSSNIEGVCDDQMVGMLAPAPFDSMDGISSLDLLQPQPQEIIKLVADSSVLARSQQATTAWCADIILSSSDQLLCSSLVPISRPTFVDSQRALLENLSSGENFQNLSYSTGNISSGESENCASFDRNTLNRDEAASQSSSNPEPPQLEFFNPKAPNPHKRKLEEECEGILLGEKSHLCSLLRSSSPAMEGGFQIVFGGNSSNSSKGKRSKGEKNSGQSTSNNIEFSQESNYEPDTEAIAQVKEMIYRAAAMRPLNLGAEEAAERPKRKNVRISSDPQTVAARHRRERISERLRVLQRLVPGGSKMDTASMLDEAANYLKFLKSQVRALETLGGSKVDGSIINSMQ

>Aco021761; AcbHLH77
MNRAMFESSLVQQMMVGGNPSWWNNMDNIKPPHQEISPLLPCSTSSAGAVASSTSSSSSSSSSSSSSLNSFPMYPQYTQSSRFLPFTSWQDNQGLPDPWNQMLLGGVMEEEERCGFGLQTKKVENWEDHQLYPSGNSHVFGAKQENLERSAYLCKKYENDQQVLKTTSPWAQSLPASSTKSCVTTNSLSTNMLDFSHSKEGKRHLQSEQSSESNSTGTGSPFKKARVQASSAQSPLIKVRKEKLGDRISALHQLVSPFGKTDTASVLLEAIGYIRFLQGQIEALSLPYLDSGSRNPRHCDAAAYEEPKKDLRSRGLCLVPVSFTLHVGGDNGADYWAPAFGEEFR

>Aco000179; AcbHLH78
MSSRRSRSRQSSSSRITEEQINDLVSKLQSLLPEARIRSNDRVSAAKVLQETCNYIRGLHREVDDLSERLSELLATTDMSSAQAAIIRSLLM

>Aco000186; AcbHLH79
MLDSSQSTVSENPTSTANSVTEKSTDGSPPNRKCQGKVPKKIHKAEREKLKRDQLNDLFLELGNLLESDRQNSGKASILGDTTRILRDLLSQVECLRKENAALQTESRYVTTEKNELKDENTALQTQITELQNELQARMGYDHPVWTNRTDSLSRLATPHPAISIQPPHHHNRHHPQPLAQPPPAVIGLVCPTPPRELQLFPVSEPAREEGEAPSNVRRPHARYPASLDSWPGQLLHTSLVVTRTPDEQCSSSGLTLTTNTLEENPR

>Aco000272; AcbHLH80
MEASAARWFADLGMEDPMFFHQWETSQLDHFTTQQIAAEFDQDLQQSLSSESCTNSFPNSYHPPTTAAAAAAAVSNTFHSASAANSKPPERPKKALKTNSWDSCMTADRSSAAAAAAAAAAPDASSPTILSFGKPVSRKNEPSLYSEFLATVKPKEELEFSNVPHGLKRSYEAMSFHGVKKASANSRPASHNQEHIIAERKRREKLSQRFIALSAIVPGLKKMDKASVLGDAIKYLKQLQEKVDTLEEQAAKRTVESAVLVKKSRYSPDDDSSSCDESSGDHQHGQSLPEIEAKISEKAVLIKIHCENRKGVLVKALSEIERLHLSIVNTSVVPFANSSLDITVMAQAS

>Aco000605; AcbHLH81
MSATNSGGDIPVPAAYFSMPLETSPATDHPDISDRALTALQSHREAERRRRERIKSHLDRLRTVLACDPKVDKASLLAKAVERVRELKTRTADIPGSARLLMPTETDEVIVLPTATANPTASVFEASVCCNDRSDLYSELVATLRSLRLKTLRAEIATVGGRVRSVLVLSREAGDDEEEEEENRHVDGEEEEEEEEEDRDVECGKGGGGDFLRDALRGLVHRPAPAADRSKRRRVVDRRST

>Aco010917; AcbHLH82
MRQPTFKSRNAHTPLLSPPYPSCTILKESSFKLRSLPIPTFMGDHHFVDNLNSTTTLKPSPHFMEMDPSCMGLVITDHLAELHGNGAGAASMAFCDESYAYFPHHHDQFPLQLQQQQQPPFADQNSSPGLLPMLGDLAPITLPPPPPAAAAFVGVRKRKAAAAADAAAETSSANSSSQPMENSLRDHQYRAIKKICSDSTAAGKRGRGNSKSKKEVEKPKEVVHVRARRGHATDSHSLAERVRREKINERMRCLQELVPGCYKAMGMAGMLDEIINYVQSLQNQVEFLSMKLSAASSFYDFNVDIEPMTTLQMMSINANEAQVMEKVRELGGYGGGGCPSFFE

>Aco012482; AcbHLH83
MDTFGDHRNNTNLNQSISEKRRGVAGGGSRVGSSRTRVKLSTDPQSVAARARRHRISDRFRTLRSLVPGGTKLDTVSMLDEAIHYVKFLEAQLWLLRQVELSLPADFAAAQMSGDFDNNHEFEMTQQLFCDSVQLQPPPPSPSPSPSLPPLPTCFIQGEEEMILDLMYS

>Aco013643; AcbHLH84
MPLSEFLLKTTKSKPGTAQSKMPIPNSSSSDPFSISDHEFAELLWENGQIVLHGHASRSSKKSWAAASAADHPEKAPAKDNVDSVDAKNEQLGADLSVHQEDDMVPWINFRIDDESLDADPVHNDFYSEFLEDIGVLGQEAKSPPNAPQEIPSKGAPAASERGRGNKPNQLIQLSQNCQSAVTNNNSRVSELGNGGGKAQMGYCMELVKNTRLAPRPNTGVNFVNFPLFSRPHAVKINDRARVEEKAAAAPVNRNLVESSSGVKSVAGIKALPRKLELEKPDAPGRQHVKVPSERIEANRERGEDHETEKDDKNAAAIKDCSFAKPETEKDPVAVVASSSVCSVNSAGIESYEPKNNREKRKVSEREESSGEQSDDLEDESVGTKKPANGRGTGAKRSRAAEVHNLSERRRRDRINEKMRALQELIPNCNKTDKASMLEEAIEYLKTLQLQLQMMSMRNGMYMPTPMMLPPGMQHINGPAMAHFSPMGVGMGMGMGMGMGMGYGMHVNGSPGCSLIPVQPMHGQQFPCPAIAGQAALHRMMLGPANPPVFGAIAPHQLSSPPCHVVKGNTAPDVTMPAAAPAPPTSDPAAAPVSRDQKEEGKNL

>Aco014156; AcbHLH85
MSGGQSNGGGWLIDYGLVDEIQGSDFIWGSQINDDPMVSSVMFGFDVSHKQEGCADNSCPKKRTRPESCAAPGTKACREKLRRDRLNDRFTELCSILDPGKPPKADKVAILSDATRLLNQLHLEAQKLKKSNEALQDSIKNLKAEKSELRDEKMRLKAEKDRLEQMLKGVSAAPQFIPYPTAPVVAYNKAVPYPNYPPAGVWQWVPPAVLDTSKDSVLWPPVA

>Aco014880; AcbHLH86
MGSPENPNWYLDCALIDELPVADGGLCWSAQGFNSSSNVSLETDGPCVNSGGSNESGSRKRIRSESCRRPASKACREKMRRDKLNERFLELGSVLDPGKPLKVNKEAILSDATRMVIQLRDDAQKLKDSNESLQAKIEELKAEKNELRDEKQRLKAEKDGLEQQIKILNSRPSFIPQPPLIPTAFAARNHSEGQKLVMPIIGYPGFPMWQFMPPSDVDTSQDTENCSPVA

>Aco014894; AcbHLH87
MGEDVIYSRMYSGQEGWWNSNIPRTSCFNGSAVMSASISCSTEFTDVMGGGFNWAALAADVPLSQSQSQSQSSSGESPGSAVSNNSLSSFQDTYHAATAQMTDLAAVSSPAVDWNQKPLLSRESGYHGLFQEGNKSSSLRGDDLQLLNPNPNPNPNPNPNQKNNMCDENISSSVSLSHNINQGLPQYQHHYESLLRSLLEPEILEPYYTQPLPEPSPPKQQPLQFSTHINTSSAIRPATTPISKDLRLSSPRNDVVVSHHALKEINLNRSNRATKSVSEGAGGLSPDIAAQQPALKKPRIETPSPLPTFKVRKEKMGDRITALQQLVSPFGKTDTASVLTEAIEYIKFLHDQVGVLTAPYLNNGNQMLHLKSLEKLNGTQGPKRDLGSRGLCLMPVSSTHAVASEIPFDFWAPTFGGTFN

>Aco025362; AcbHLH88
MAKCYASTDEGKQVVVSFPSLYKGSLILPRPTTAATTTTTTTTPRPLLRSGSPSCSSASSSSSSVAAARASKIHSEAERRRRERINSHLSTLRCMIPDAHKMDKASLLSKVVDQVKDLKRKANDTSKTVNVPPEIDEVTVERHKGGRDHTFSGDETLRMRVSVTCDDRPDLFASLIQAFHKLRLRTIRADITCIGGRVQNVFILCGTGEGSENMCLGSLKECLKEALAKAASPEVLLSSSSSSSSSSSFSSKRQRLLGSH

>Aco004054; AcbHLH89
MALEAVVFPQDIFGYNCKELYTNMGGIWGNDLANMVVVEEMEMSECEMGGWVGENWNPSSSSLVQNLDEWEINSSSAEVWGVAVEAEGEEAMKAVDGRRKRRRTRSVKNMEEVESQRMTHIAVERNRRRQMNEYLAILRSLMPPSYVQRGDQASIVGGAINYVKELEQLLQSLEVQKRLKQVQTDSTSLASPFANFFAFPQYSSFSGRGGGGGSNSGTVTDNQSAVADIEVTVVESHVNLKVLSKRQPKQLLKMVAGLQNLRFIPLHLNVTTVDDMVLYSFSLKVADDCHFTSVDEIATTVYQMIGRIEEDAKFSK

>Aco004136; AcbHLH90
MSSRRSRSRPSSSSRISAEQINDLVSKLQALLPEARIRRNDRESAAKVLQETCNYIRSLHREVDDLSERLSELLATADSAQAAIIRSLLTQ

>Aco004138; AcbHLH91
MSSRGRSRITDDEINELISKLQSLLPESRRRNIDRSLHREVDDLSDRLSGLMATMDDNSPQAEIIRSLLRS

>Aco013347; AcbHLH92
MQSDQIVGASSVNTPCSSARFGDQYFGQAMDFNGPSGFSKYNNSATQQRLQFYNPSADFSTGSKRSNDKNFKGKKRKGAVDGVSIYSETPQDRIQQMEVKMESLSNAVEKDEIKLHSGNQAKESTFDGDASKDDYIHVRAKRGQATNSHSLAERVRREKISERMRLLQDLVPGCSKITGKAMMLDEIINYVQSLQRQVEFLSMKLAAVNPEMSFNLEQILTKDILQSRDMRPALLGSGLGLSNVQSSFHGSSPNGIVLPEMLFGIQNPGNTPATSIPQFPTISQIPGLWDDELQNIIHMGFIPSAVTGGTENSNVSGSMKVVE

>Aco013365; AcbHLH93
VIIISQESEEPTFMYQPHDMGSMDPLSLVDEENSPFPVENYAPDYCFDPSFSGSSSSPTSLRFNPSMNAVKTEGPPTAAASAASASSNILSFGGDDHLLRPILNSDHHSGAATMYSHGVVGKDETIDFQQRERSSKGSSRASYCTAQEHVIAERKRREKLAQQFIALSAMIPDLKKTDKASLLGGAIDHLKQLEEKVKVLEEQATRKTTESTILLKNSHAISNASSADHETLPKVEATLQGKTLLLRIHCEKRKGVLVMILSEIEKMSLTVVNTSVVPFEGSSLHITATAQAC

>Aco019856; AcbHLH94
GGGGGGGGEGEGFAWSIPSMHHHHHHHQISSFQNPLFHSLPPLPPPPPPPPPPPPPPPTFYGDLYARRASAALQFAYDGGGIGSSSSDPFGLAGLYMGPEALVRTGGLVSSSSSPFNIGLHRELGKMTAQEIMDAKALAASKSHSEAERRRRERINAHLAKLRSLLPSTTKTDKASLLAEVIQHVKELKRQMSEIAEESAPLPTESDELSVDAASDDEGKLVVRASLCCDDRSDLLPDLIKTLKALRLRTLKAEITTLGGRVKNVLVVTGDTDTDNDNDNDSAASASASASAADDPQPPPPPPQQQSLASIQDALRAVMEHRAASAEETTSAGGGVKRQRTTNLSSILEHRSI

>Aco005839; AcbHLH95
MVMGRLWSEEERAMAAAVLGPRAFEYLSGCRVSPEGVVAGDVDAELQTKLQEVVEGPSGRPWTYAIFWQATRARTGEALLVWGDGHCRDAEPRRRGRSIVARAGEAEREEERRQRMIKRVLQKLDAVYGCGDENAAIRLDRVTDAEAYFLISMYFSFPLGEGGPGRALASGKPFWVSDVATSSSSSSSEFCVRAFLARSAGFRTVAFVPLDGGGVVELGSLDRVPEGFEAMKMIRSVFSRGENRLKESIPRIFGMDFTSIGRPPQISASGPEERPPRPRPRPRPQQLPPPPPPLGTVDFGNGGGATSAAAAASPAAAQSGGALALDSDLPDVESSSKDDRRITAAAAAAAEERRPRKRGRKPANGREEPLNHVEAERQRREKLNQRFYALRAVVPNISKMDKASLLGDAIAYIQELQSRLKEFESDRARWNNPGLGPRPPELEPPSVEIQALHDEVIVRVTSPLDGHPLSRVIQAFNESNVSVVDSNVLATNGSVLHTFVVKSPPDSEQATRDKIVAAISCEMTTSQSQSQ

>Aco005872; AcbHLH96
METGWYYSSEGSDMDFSQTLSDGYWGSFCSIDNGLTLHCNENGGANSMNLGADQEQMQLYQAKLITPTPHSSITAMRSHNQNLNPKFPTLERDGDADDVAIMRAMVVVISSTSSSSSPSSPSVLSNPPSQDYFPTTQSQRRGNGKIGAFKRYSRSDLTAKSEAEDNCNYGQSMMKRSILMLRRLSSFEGRSRESKSTSNQLHHVISERRRREKLNASFEALRMQLPPGTKKDKTSVLINTKNYVNTLRSQISELKEKNRRLETCLMITDEGKEASHGLNGRAQVRLIKCSSPTTSELQQINLTITLRVECDIIELVLHILECLKVIRSINLISVDAYTYSPQMNLFAKASIKFGIKDCDWDEALFHEAMTRAVDDVILNTATPIISELSRLF

>Aco003204; AcbHLH97
MSQEGDGGGGQGGFPWENPQWAFSNSDNSGSGRNDPKTGTRPVESTTNSPTNQKDPGERKRNRPGPSKSSSSKGVGGVGDSDHELHIWTERERRKKMRNMFSNLHALLPQLPPKADKSTIVDEAVNYIKTLQQSLQKLRKKKLDRLRGVVLSDPSSSSIVGFNTANIADPTREGFMAEQGKKWAAMSSPTALSIPHFPFCFQTWSTPNVVLSVAGDDAHISICCPKKPGLFTAALHVMEKHKLEVVSAQVSSDYFRSMYMIHARATRVSDQFPETLMIEEIYKLAVGEMILWIST

>Aco003613; AcbHLH98
MELFSNQHHQASLLSPSNLPNSFMEKNFPPQQLGEMSNETSYCFPYCYLSEAIPEFSNNSDSTARAYESSSSLDTVRNASSAGTQMSHSAVITDPGSPSGKRRKNRDSTSLCLAPLSNDAMESKTKKQKRPNGGLKKVEEKKPKGDEIKHKEVCGEPHEGYIHVRARRGQATDSHSLAERVRREKINKRMKMLQSLVPGCDGVSGKALMLDEIINYVQSLQNQVEFLSMKLASMNPMFDEFGVDFECLMNHPEVARTKAKCQHTLLLIWPISLGFDIDLTLYSMPHEQVPTVDQTNHSQATPSEATTVNYQMVDNSTPILQHGQGPTYFPQEANSTRHKDGT

>Aco016776; AcbHLH99
MSGFEMFSLREVLRRLCAEIGWSYAVFWRAIGPSHRMLLVWEDGCCGHTSGILGSEASDLLLEEHGLVQKLHNQQAAKYGSQTEESVNALVRKTMMTQVHVIGDGIVGEAAFTGNHRWIVRDSLKDYGSLSKDADEMNQQFAAGIQTIVIIPILPRGVLQLGSTQMLMENVGFLIHTKNLLSQLNNRSGSSFSRLSLDTNPSFVEPILHCSRQLEGETVGARVYTKANLSLGKQGVTNDNGSKIQTQPSVMDSDFTSRLTSLEEQLLFMSHDGLLEPGNNAGSAVQGTGAVRLILNGRDNPNLLEDSNIVSPHLQNKLLNTVDSLGKFGSLLHGSNEITRVLCANSSGTGTSQMIDQRYNNAESSQQNQPSSPYRVTQAVPIEGNEGRHENDLVEAAGLRRSIQNDRQTRSIANSIANESNKIYATENGNGVKDENDCKLSHIRTEKTSLLPLDRSMESDLFDMLGPKFRQFHDGSDNSLGINAFIHSIKSDASPAFDSANDDFSQSGIFSVADTDQLLDAIVSSINPTAKQNSDDSISCKTSQTDIHNSACYGGYAPSSEVKQDELSYLPPVPIKNETAISSYVKPQCSHPQSTGVHKSQIHLWLESGQNMKCDSMPASNSKKLDITGKANRKRSRPGESPRPRPKDRQMIQDRIKELREIVPNGAKCSIDALLEKTIKHMLFLQSVTKYADKLKEKGECKIVDKDGGLLLKDCYEGGATWAFDVGSQSMICPIIVEDLNQPRQMLVEMLCEERGFFLEIADFVKGLGLTILKGVMEARKSKVWARFVVEANRDVTRMEIFLSLVRLLEQTAGGGIIPMNVNNVDATHTLLCQPSSIPATGL

>Aco022880; AcbHLH100
MLSRVDGMVWMGEEEERRASVGGGGGGGEEEGDSLSWTRANPNPNPNPNHNHNHNHPHGAKEDEMAGLSRFKPMLDDDDGDDDDWYLAPNPAHQPFDALPTHQHGIKGAAFATAPQHEPSLLLPPPQPGMGMGVGVGVGVXLLPLLLVLLLLGCGCGCGCGCLFNLDHAHHAFFPSKPSLSSLLSVVSSNPFDAGFDLGCDASGFAHVSASPVLLNKGGQGQELLGFGGFGPSGPMAGCPELSSLAQFPAGPSSVSPFGQLGFDCFENSPFLSRNKVLRPLEIFPPVGAQPTLFQKRAAAALRQSSATTEEKGGSLWLRSGIMDEESEKKRRGGTEEDEELDEGSIDGSGLIYDSDDAIAENAKGEENARNGTSGGGGNNSNATSTVTGSGDQKGKKKGLPAKNLMAERRRRKKLNDRLYMLRSVVPKISKMDRASILGDAIEYLKELLQKINDLHNELESTPSTSSLPPSSTATTPTSFHPLTPTLPTLPSRVKEELCPSSLPSPNSQSARVEVKLREGRAVNVHMFCARRPGLLLSAMRALDGLGLDIQQAVISCFNGFAMDIFRAEQCKDGPGVLPEEIKAVLLQSAGFHTVM

>Aco026511; AcbHLH101
MHAFGGTRRSSKAESDMPKRLETSSVEPVREPYRDVPVNLITAPRNDMPTLQNIWACQKSFLNVCGNDADMLFPGPFSSSTRILHANPQAINRLHDVRGFFELKDGHFDDSKFSSNPLRKVSGNSSLADIGASGVKSLGPIISSRITSTASGSSKAVDSVECRTHIEVYNLPMITHSMLDAEEQTGRMSSNGENQNFNQSYALMKPKSNEHYFNLAEADCKEIVRSSKSKNKSVANDKLRRARISEGIKALQNCLPYSKKRNKESAFDDLIDYVKFLKLQLKVLSESRLDGEETDHRFVHVEGYGHYLLHPQMSAEPLGEMMGQLMESNMQVANQLLESKGIALFPLEFAYTLLRSS

>Aco027966; AcbHLH102
MESASRSSEDEGDFGRRLSSSSHQELMVKVDGKSGEQKLGTPTTPRSKHSAMEQRRRCKINDRHTLRDLIPHSDQKRDKATFLLEVIEYVKFLQEKVQKYESYPEWNQDNVKLTPWSNNRVPADGISDLSHATRVGPIPGFTFSGKLVDNSMSPTPAMLSNSQNVMESDLTADNVASQPQSQSQWQRSSCAADCTITSDMAADQEELMIDEGTISVSSLYSQNLFAALTHAMESSGIDLSQATVSVQVNLGKRASCGRPTSGTTLSNTKDHIDAPPANQTMEDFPMATGGEEPEEPSKRHKVDNS

>Aco029228; AcbHLH103
MAVAFPPKEKRKQKQKQKQKQRKRESGCQNMEEMDPEYNFYWETKRFFENEELDSMWGLEEAMSSYYDSSSPEGSGSGNSGGGGGGASSSTAAALKSIEMERNRRRKLNERLYALRSVVPNISKMDKASIIKDAIDYIQELQEQERRLLEDLSHLQATADKKESAAGGADDFGLSRGRKKMKSTAPASPPVEIVDLGVAEVGDKIMVVSITCQKRKDTVAKVCEALESLNLKIITANITSVSGSLLHTLFVETGEMDSAQMKEKIECAIAELDALRSPTSSMSL

>Aco018903; AcbHLH104
MPFSSLDPYSGGDSDELGSRGHDFDESEEGAEEPVRRIQPRSSGSKRSRAAEVHNLSEKRRRSRINEKMKALQNLIPNSNKTDKASMLDEAIEYLKQLQLQVQMLTMRNSLHLHPMYLSGVLPPLQASEMCMGLVADNSTGMSIGMGILSLNQDSAAHHSLDQLNHCAPSNQPALLTSATSITIPECSFQIESSQSQIGPFQLPSVTAEALFTEDILPKHQLASGRVAASLQDEMKPKRVAATSRHFAAQASSPADIDHLQSCMGRGEKLVNINMIANDPQGHIFVQHLQRLESGRAPDADAKAE

>Aco008079; AcbHLH105
MSTGASRGAFFLQSSSRITEFVIRKLIFLHLASLSLTRDRESYSRLGSDSDHAVAGDNRRLNSQLSLSFSRQQSTLSQISEIGLSDVGESSQSDEGNAGHSLAPNGFSIGSWDETNSIVFSAPVSKRGKDNNGDVMASLSHIESQFGLPRSSLEMATMENFLQIQQDQVPFKVRAKRGCATHPRSIAERERRTRISEKLRKLQELVPNMDKQTNTADMLDLAIQHIKGLQSQVQILSQERDDCRCGSKREKN

>Aco016081; AcbHLH106
MEYQHEDYTNHYTNLDQTYNYWFVDDDLMFFDCGLIDHELRSMNKDLVPNTLNIDTVSVSQGLSLCLDDSGGGLLSEGLEGFPKENRDDGDSFVTKTRIRRDRSKTLVSERKRRGRMKEKLYELRSLVPNITKMDKASIIADAVEYVNDLKSQVKNMEKELLILETSLKSDQVPQNLLKNTMRNTQLEEAIASPHGAKILRLNTTEVGDQRFFFMIECENKDGVASSVYSAIESLMFFHLECSNFSLASDRFVLSLTINVEEFDEEIEASTMKLWVMGALLKEGFEFEGICIS

>Aco016125; AcbHLH107
MMENNDPFFSAINTAAAAAAASAANYFPWRRPQLLGSADLDAPRNRSARRKRKRKSSHGVRRRRRSSVKEEEEENNDNDDDDDEEEEDEEDENPLLSPNKLTDQDSNPKRCRADQSERTATINPKTAEIGANRDSNEKENSSSKQDYIHVRARRGQATDSHSLAERVRREKISERMKLLQNLVPGCNKVMGKALMLDEIINYVQSLQRQVELLSMKLAAVNPLDFCVNSLLRPKDMNNRAQQQQAPDLPAAQIYHRSENSSAGSSYSQRINGTPLESFENKFVFDELDPSFCSSISMEFPTLNGFADATQLGNFWEDDLQSVVQMGFFGQSRDAQANLLAELLRPCDDRQQED

>Aco023503; AcbHLH108
MLLHPALFGAFQADHRASMAPPLDCHLVHHHTQHGSTLQDESAEEGGIEMVDYVLSSPAPLPPPQAQISFDRLSFSDVVQFADFGPRLSLNQSKASEDENFFLKFHVLGDKLLQEEVLPPPPPPPPPPQPTAFEEDKEGGGEEGRLLENVSLSQQQLSFVGQAEKTGVGEVKNRRKRPRSMKTSEEVESQRMTHIAVERNRRRQMNEYLRVLRSLMPGSYVQRGDQASIIGGAIEFVRELEQLLQCLESQKRRRLYGSAEAPRTVMDAAALVPVQHQPLQQQQQQQQQQPFYPFPDDNVKILDLDPTGALREEMAENKSCLADIEVRLLGFDAMIKILSRRRPGQLLKIIAALEDLQFSILHTNITTIEQTVLYSFNVKIIGETGCAAEDIANSVQQILSFIEANTI

>Aco007275; AcbHLH109
MVALSPIRLQKALQSVAQSIQWTYSLFWNPCPQQGVLVWGDGYYNGAIKTRKTVQPAEVSAEEVTLQRSQQLRELYESLSAGEASQQQQQQLQLARRPSAALSPEDLTESEWFYLMCISFSFPPGSGLPGKAFARRRHVWLTRANEADSKVFSRAILAKTVVCIPLMDGVLELGTTEMVEEDTGLIKHAKSFFAEHHEIQSMPALSEQSTSNPAKYNEPPSFQVGVTMHRMHAEPTESDRQNDEDDDEEDDDHDDDDDEEDEIEGGSGSEVGTLKCSHMITPTLTRIEGATTPNAEHDELMQLGLSQNVRLGSPDDCSNNLNTQLQMVGVCCNTSGVSNHQREDETYHCWNFLHEDICSELQQQSSVQAQELSQEDAHYSETVTTILRHNSSRGDESKSNDYFVPSRQTAFAGWNSTRDHPQIVLSEGTSQWLLKTMLFRVPTLHCRYKDENSPPSIEGEGGGSRSRKGAAQEEPSANHVLAERRRREKLNERFIVLRSLVPFVTKMDKASILGDAIEYVKQLRKRMQDLESRNRQMESLTKTAELQRPGSSKDPNVPRGSGSPQGSGVDHVATSQQRAFSSDKRKIRVLEGTRVADAPSTNVQVSIIEADALLEMQCPYKEGLLLKIMQTIHELRLEIMSIQSSSVNGTLFAELRAKVKEMNGKRASILEVKKAIYRVFSNNR

>Aco010168; AcbHLH110
MNHLVPEFELDVDDPFLVSSFVPAPHRCKISESALAESELLELIWRNGPVAQPVHGDWIRGLSPPPPESPGSAALSGRADAGLVVDVASQLFMEEDEMASWLHYQIGGGGGGDLFFGSSPQDIGNDGRAAAAAAAAAAASTAAAERLVAAPKESAVGSNEVSVVEVCDPYGVGTGGGGGSPRACEQALSSSSEASSERRGMAHADERKRKAREGGGGSGCHSEKDTEFRTLEKRKDARRSAAGRRSRAAEVHNLSERRRRDRINEKMKALQELIPRCNKSDKASMLDEAIGYLKSLQSQIQMMSAGCSINPAMFPGVQPYMPPMGMGMGMGMGMGMGMHMGMNMAPGMTIGAEVGGGGLRPPMLPFGPLLPCASIASPARPLPIMPSIHPPNVVNVNQFRVQAASQQALASNMQSQYMVQIPHVGDPYHGFVPVNPFQVSSQNGVVEKPEANKQAGNNENIPHGRLKCRNLWK

>Aco010345; AcbHLH111
MASDAIDDYWIDGEGSDGELRCALESFCDVAAPAPSAHAGIGVEESYGGVCCLDQTSSRKRARDESCTGSKSKACREKMRRDRLNDRFLELSSILDPGRPPKSDKASILSDAARVVAQLRNEAKQLKESNEKFQETIKDLKVEKNELRDEKTRLKAEKERLEQMVKAVSAPPTGFVPHPMAFHPAAAAPIFAPHGQAPGNKAAAPVAAYPGVAMWQWLPPAVLDTTQDAKLWPPNA

>Aco013073; AcbHLH112
MELDENGFLEELLSMSMRRESTWDAFSAAAAAAAGIGEFFAAGEGGFDCFMHESQPCAAVLPGFAAAFDAGCSDRSHDVHRATTTEPNFDCLSEVWFPAPPLPPPPAEGALDVGLVHGGESSVCKVEMGDPMAFGTAVGGERKKKKADGVPSKNLMAERRRRKRLNDRLSMLRSVVPKISKMDRTSILGDTIDYMKELLERIKHLQEEMEVGSEKAKLVSLFKDQLNPNEILVRNSPKFDVERRESDTRIEIYCAMKPGLLLQMVDTLDALGLEIQQCVVSCFNEFGMQASCSEDMEQRALMTSEEVKQALFKNAGYGGRCL

>Aco013092; AcbHLH113
MGGGEMITEALRPLVGSNGWDFCITWKLSPDQRYLVWMSCCCSGVVTQSSSAAEIFPLLSTQKFSCRDNVYQHARTGACDALEELPLSIPLDSSLGYLKHIIYTNMVLSKSFVMGEDQQAVEFVMSQCTMDIGGADDGVQSWTEPPEPMAADGSTAFPWDMPHPVDQARLFGPSLGLFSGPMYRTKSGGKRHHSKNLVAERRRRKKLNDRLYSLRSLVPKITKMDRASILGDAIEYIMELQKQVKELQDELEENHNQDDEAAKGSNNNSNGHNMGVSVHNELMNHHNHGLLMENEGSPNCSLMVIDNNNNNNNNNNNNDATGKQGHDATCKEEKGHEMEPQVEVKQVEGNEFFLKVLCEQKQGGFARLMEAMTSLGLEVTNANVTAYKPLVLNVFRVAIDHLGNIFLQKRDNGAVQAEQVRDSLLEVTRDSTGGWMEAGQAKSTADNGGMDYHHHRHHHFHHQLNSHHNIHYLNH

>Aco021969; AcbHLH114
MGSSENANWVFDCPLLDDVAVAGDDFPASGSGFYWGSQGINGSSNGGVEISGSFLDSECVKERGSNKRVRSESCQPSSKACREKMRRDKLNDKFLELGSILDPGKPPKMDKAAILSDAVRMVTELRSEARKLKESNESLQEKIKELKAEKNELRDEKQRLKAEKESLEQQIKIINSRPSYMPHPPVMPAAFAAPGQSAGHKLVMPLIGYPGYPMWQFMPPADVDTSQDAESCPPVA

>Aco021976; AcbHLH115
MPRRPRGAAASQAAEAEAEAEAELEAELQGDFVDSVLDMEGGIGGETPMEKWEMRFKSKNLEAERRRRGKLNTNILALRAIVPKITKMSKESTLTDAIDYITLLQKQVLDLQTELLETDGDLIHKNEANEDEEGEKQRSPSSETVAPSVTVQCQGQVELIPMGPNKYQLKIMCKNRMGQFTKVLEALSCFNAEVAEISSVAFFGFSKSVFSVEVKEGEEGEIIELRNLLLALVGASEN

>Aco027975; AcbHLH116
MTLEALPSNELPAFLIYDTINAAAAAAAASSSRLLFNGNGGITEESPLIGGMAAMAEEVAVAAGEGRRKRRRRARSGKSKDDAESQRMTHIAVERNRRRLMNEHLAVLRSLTPDSYVQKGDQASIVGGAIDFVKELEQLLQSLEAQKRTLLQQQQQQQQKGNSDIFPTACYENDSPPFAQFFSYPQYAWCHPARDYPSLEIQHRPATVAPSVADIEVSLIETHANIRILTARQPGQLLKMVAGIQSLRLTILHLNVTVLDAIVLYSLSVKVEEGCDVASVDDIAAAVHHILSLIKAGTNLVDR

>Aco028213; AcbHLH117
MEENSRSFSDLLAKSNQMKPIRPDNELVELLWHNGQIVLHNQTHRKSPPPCIDFRQSQNPKSVLKSEITNVNANNLAQEDETMSWFQYPIDDPLERDFYTEFFELPNGGFNDNSLGKEKCAETENESNAVNLGSGVQVGGGGECSSIMTIGSSICGSNQVPTQVEGSNLHHLNTAKLPIEGNNYNSSTHEATATSSSGGSGCSIGITQQQSISNQGTKRKERSTEESESQSEEAEQESIEANEPTKQSASRRSRAAEVHNLSERVRLTDDPEIKPACLIPQSPYSIANSFLMQRRRDRINKKMKALQELIPNCNKTDKASMLDEAIGYLKSLQLQVQMMWMGSGMAQMMFPGVHQFMSHATMGMNPASMPSMHYPVQMPTVPFCSSESFPNQMQNINFPGLNSMQVTNFCVNGACSLQSNQISVLPSYNLSHAIGEFPTENVRDDKSKYSEDPLKVKVFCWLLLKKRILTADVLVRRGWTGNTVYVLCGGEEETTDHLFSRCVYFRFLLVMTLDDTEIAGLGLGVHPLLDSLAARSSMPPKARLDVLVAAWWVTWEIRNAVIFRHTQTDPILAVTKIVQLYNLWEMPFSAS

>Aco028872; AcbHLH118
MLALSPPLFSSPLENNVMSNELRSDDRIFASNRIQHEDGMLQQSLLFPSPPHPELEFDDQSRASNSAKDSPRSHKKLSHNAYERDRRKKLNSFYSSLRTLLPESDRTQKKMSIPSTVSRIVKYIPELQSQVARLSRRKEEVLAEIAKRKEGSPTKEGIEFLINVSATCLNSEEVMVHITVLNKNISLPLSKFLKVLEGEGLQLMNASTLTSFGDKTFYNLHFQIKRCTGMEGQIFCEHLVKVIKEKGRDDYSSIQ

>Aco030561; AcbHLH119
MGLHNNSPLTHENTSPSTVEISWGHIKKKGEAAAPDSSQSSATPRLENGYSQENVARKKSSLGGGGGGRRGRRCSKEEKKAKEVVHVEARRGQATDSHSVAERLRRERINERMRCLQELVPGCYKEMGMAGMLDEIINYVQSLKNQVEFLSMKLSAASSFYDFNLDCDQPMAAPQVLL

>Aco030901; AcbHLH120
MVALSPIRLQKALQSVAQSIQWTYSLFWNPCPQQGVLVWGDGYYNGAIKTRKTVQPAEVSAEEVTLQRSQQLRELYESLSAGEASQQQQQQLQLARRPSAALSPEDLTESEWFYLMCISFSFPPGSGLPGKAFARRRHVWLTRANEADSKVFSRAILAKTVVCIPLMDGVLELGTTEMVEEDTGLIKHAKSFFAEHHEIQSMPALSEQSTSNPAKYNEPPSFQGATTPNAEHDELMQLGLSQNVRLGSPDDCSNNLNTQLQMVGVCCNTSGVSNHQREDETYHCWNFLHEDICSELQQQSSVQAQELSQEDAHYSETVTTILRHNSSRGDESKSNDYFVPSRQTAFAGWNSTRDHPQIVLSEGTSQWLLKTMLFRVPTLHCRYKDENSPPSIEGEGGGSRSRKGAAQEEPSANHVLAERRRREKLNERFIVLRSLVPFVTKMDKASILGDAIEYVKQLRKRIQDLESRNRQMESLTKTAELQRPGSSKDPNVPRGSGSPQGSGVDHVATSQLKAFSSDKRKIRVLEGTRVVDAPSTNVQVSIIEADALLEMQCPYKEGLLLKIMQTIHELRLEIMSIQSSSVNGTLFAELRAKVKEMNGKRASILEVKKAIYRVFSN

>Aco031432; AcbHLH121
MAVVFPPKEKRKQKQKQKQRKRESGCQNMEEMDPEYNFYWETKRFFENEELDSMWGLEEAMSSYYDSSSPEGSGSGNSGGGGGGASSSTAAALKSIEMERNRRRKLNERLYALRSVVPNISKMDKASIIKDAIDYIQELQEQERRLLEDLSHLQATADKKESAAGGADDFGLSRGRKKMKSTAPTSPPVEIVDLGVAEVGDKIMVVSITCQKRKDTVAKVCEALESLDLKIITANITSVS
