# Supplementary material for: Genome-wide study of pineapple (Ananas comosus L.) bHLH transcription factors indicates that cryptochrome-interacting bHLH2 (AcCIB2) participates in flowering time regulation and abiotic stress response
Source: BMC Genomics. 2020 Oct 22;21:735. doi: 10.1186/s12864-020-07152-2 (PMC7583237; doi:10.1186/s12864-020-07152-2)
Supplement: Supplementary file 9 — Additional file 9: Table S9. DNA sequences analysed in the present study. [file 12864_2020_7152_MOESM9_ESM.docx]

>Aco006737; *Ac*bHLH1
GTTCTTCCAAACAAAGTATTGTGGCTCATCTCCAAGACTTAGTGGTGATGTAGGAGAATCAAAGCAAACATAGGAAGATTGATAGAGAGCTACATCATGAGTAATAATAAGGTCATGAAGAGCTAGGAATATTGTTTTGCTAGCTCTCTTGTGCAACAAATGATATGTAGGAGAAAAACCCTAGCTGGTGGAACATGAATAGGCTAAGATCACAAGCTCAAGACTCCCCACAACAACCTTCTTCCTCCTCCTCTTCAGCACCACCACCACCACCATCATCATCTTTTTCATCTTTGTCGTCGTCATCATCATCGCCGTCGTCATGTTCATCTTCTTCTATGTATACTCACTACACAAATCAATCCAATCTTCCTAATACCACCCTCTACAGGCAGGATAATCAAATTTTTCCAAAGTCATGGAGCCAACTACTGCTGTAATAATCTCTCTCTCTCTCTCTCTCTCTCTCTCTCTCTCTCTCTCTCTCTCTCTAGATCTCTCTCTCTCTCTCTAGACAGACTCTGATACCATGTTGTCCAAGAGATCAACATAGTAGTAACTAGCCAAAATTAGTCTTAATTAGCATATCTATCTCTCTCTCTTACTCTCTCTTTCTCTAGTCAGACTCTGATACCATGCATGTTATCCAAAAGATCAAGTTAGTAGTAACTAGTCAAGATTAGTCTTAACATCTCTATCTATCTATCTAACTATTAGCCTTATGAAAAGACCTGAACTTTCAATCTAAGTAGTTCAAGCATGTCTCTTATTTTTTAACTTATTTCTTAACTTGTATTTTGGTGATGAGCTACACTTTAACAACAGTTTTAGTTGGTGACTCACAGGGATGGATTGGTAGAAGAAAAAGAAGAAGAAGAAGATGATGATGATGATAGGATGATCAGCAGCAGCTTTGCTCCTCATTTCCAACCCAAGTTGGTGGAGAATTGGAAGGATCAGGTGCTGTACCCACCACCAAACACCAACATAGTTGGTCCAAAACAAGAGGGCTATGAAAGTGGCTTCATGTATCAAAATAAAACTGAGGATATTCAAATCACAATGAGGGCTCCATTTTGGAGCCATGCTCTACATGCTTCTTCTATTAATTCTTGTGCCACAACAAGTTTAAGCAGCAGTAGCAGCAGCATGTTGGATATCTCAAAGAGCAAGCCAGATAGCAACAATCTACCACTAGAAATTTCATCTGATCAGGTATACTAAGGGGATTAATATTCTACCATTAATTAGCAACATGCTATTTGATTCTACTGCTGCTTCTTCTTCTTCTTCTTCTTCTTCTTTTTTGGTAAAGATTTATGGAACTTACTTGAGCTGTGGATCATTTTGATTTGGCTATCCAATATTTCAAAAGTTTGATTTTACTGTCCAACCTTTCAATTTGATTGATTTGATCGGTCAACAACGTTTCAACTTCAAAATTTAAATGCATTAATTATTTATCTTTATAGATTTAGTTAGTATATTTTATATGATTCTCATAACCATAAATTTATTAAAAATAAGCAATTAGCTTAAATTTTGAAGTTAAAGTGTCGTTGATTTACCCAAATCAAATATATTAAAAGATTGGATAGTAAAATCAAAATTTTTGAAAGCTTGAGTAGAAGATTTAAAGTGGTCTATAGTCCATGTAAGGTTTGTAGAAGCTTTACTTTACAAACTTGAAATTCTACTTTAAGTGACTTAATTGTTAAAAATATGACAGCGGGGAGAAAATTGAGATCAAGCTAAACTTTTAGAGACCGAGTTATTTGTTCGGATTGGAATTAATGCATGCTAATTAAGGCTGAAATTAAAATTCAGGTCAAACTTCGCCATTAAATTTGCAATCAACCATTCGTTTGTTTTTTTTTGTTTTTTTTAATCCAAATAGTGCAACTACAAGTTAATTTGCATGTCCTTCCATATTTGCTTAGAACTTGTACATAGTATCTATTATTATGTACTCTTTCATTTTGTTTCTCGTTCATTTTCTTTTTCTTTTTTCTTTTAATTTTTTATAGCTTAAATTATATAAAATTTTTCTGTTAAAATTCGATTTTTCATTTTCTCCCCTGTTATTTAAAAATCTACACTTTGTCCCCTTGTAAAATAAAAAATGTTCACAAGTTAGTACGGTATGAACTGTGATGACAAAATGATCATTTTGTCCTTCATCATTTTATCTTCTCCTTCATCTTGCTCATCCGCCGTCTCGATCGGCCGCAGTTCGCGCCTCGATCGGCTGCGGTTACTCTCCATTTTTTTCTCCACTTGCTCCCCTTCTCCTCTTGCACCCTCGTCGCCCCTCCATTTCGGCGACAGTAGAGAGGAAATCGAGATGGGCTTGGATGGAGAGGTGGGCAAGAACAACGAGGACGAAGGCTAAGCGGCGGAGGGCGAGGGGGTGTTTGTAGGCGCAGACGGAGATGCGAGCGAGGGCGAGGCAGTGGAGGAGGGTGAGGCGGAGGAGAGCGAAACAACAGTAGAGAGGGTGGAGGCTTTTTTTGGCATAAAAAAATATTTTATAACGGAATCCTAACGGATCACTAACAGTAGGGGGTGAAGTGAATATTATTTATTTTACAAGAGAGCAAAATATAGATTTTTAAATGACAAAAAAGAACAGTAAAAAATTAAGTTTTGACATAAAATTTTCTGTAGTTTAGCTTTTTTTATATGTACGTGAGGAGTTCTGTTGATTTAATAGTGCCAATCTCATGTAAAAAGGTGAAGCTTTGGAGAGATTTGTACGAGTTCTTAACTTTTTTTTTTCCTTCTTTTTTTAATAATTTTTCTGCTTTTTGTAGTGCAACAGCACAACTGGTTCAGCTCTCAAAAAGGCAAGAGTCCAAATCTCCTCCGTGTCACAACCAAGTATCAAGGTTTTGTTTTGACCATTATTTTTGTACATATTCACACTCACACACACACACACACACACTCTCTCTCTCTCTCTCTCTCTAGTATCAATATATCACAAGATGTAAATATTAGAGAGTATAATAATAATAATAATAATAATAATAATAATAAAGTACTCTTTAATTTTTTATCAGGTGAGAAAGGAGAAGTTAGGAGATAGAATAACAGCTCTTCACCAACTTGTTTCACCATTTGGAAAGGTATTTATTGCTCATGACTAAGGCAAATTATAGGGGTTTTTTTTTGCAAATGGCCCCTGAAAAAACTTTATTTTAAAATTAGCTCTAATAAATTTATAATTGCAAAAATGACCCTACTACTGCCACGCAGGCGCCACGCAAGCGCCCCGTGCGCGGACTGGGTGTCTGGTTGGCAAGTTGAACACGGTGAATCATTCATCGTGTTCAAACACGGTGATCACCGTGTTTCATATTTATATTTATATTCCTTTTTTTTTCTTTTAGGGATGTCAACGGGTCGGATTTGGATCGGGTTTTCAAAAATCCGAATTCGAATCCGAAAATCAAATTAAAATCCGAACCCGAATCCGAAAATCAAATTAAAATCGGAACCCAAATCCGAAAATCAAATTAAAACCCGAACCCGAATATGAACCCCGCGGGTTTTAAAAATCCATACTCATATAACTTTAATATATAAATATGGGTATGGATTTTAAAACCCAGCGGGCTCAGATACATGTTCGGGTTTTTATTTGATTTTTGGATTCGATTTCGGATTTTTGAAAATCCGATCCAAATCTGACCCGTTTACATTCCTAAAAGAGAAGAAAAGGAATATAAATAGAAATATGAAACACGGTGAATGATTCACCGTGTTCAACTTGCCAACCAAGCTCTCAGCCCGCCTACGTGGTGCTTGCGTGACAGTAGTAGAGTCATTTTTGCACTTATAAATTTGTTCAGGCTAGTTTTAAAATAAAATTTTATCAGAGGACTATTTGCAAAAAAAATCCTAAATTATATCATAGAGTGCACAAATTACCTTAGTGCTTCATATATAATAATAAAAGCATAAAAATATAATAATTAATAACACCGAAAAGGTGAAAAATGTATGAAATTTATCTAACCTACGACCCATTTTGACGCGATTATCTAATTTGTTTTTTTAAAAAAAATCTGAGTTACACTAAACACATGGATCACATGAAACATTATAATTAAACTGATTGAAATAAGCAACACCCCAAAATCTGAAATGGTAACCTATCAAAATACTCACTCAAATAAATTAAAATTTTAAAGATTAAATACTCAGATTAAGCGGATGATAGCCCGGAAAAATTTTATATTTGTTGTCCATCTTTTTATTTTTTTTATAATATTCTAAAGTAATGATATATATAATTGATAATTGATAGTGGAGTTTTTTTTTTCAGACTGACACAGCATCTGTACTACAAGAAGCCATTGGCTACATCAGATTCCTCCATCATCAAATTGAGGTCTCTCTCTCTCTCTCTCTCTCTCTCCCTCTCTAACACCAGGAACACAAATTAAGTAGTGAGAAGATCCTTTTGGGACTAATTAAGAGCATGCACAAAATAAAGTGTATGTAGCACTGTTATTTGGTGTAGTAGTATATATTTTGGCTTACCGTCCCTAGCGTAAGTGACAAAGGATTTGGTGGTTGATATTCGAGATCTCAAGTTCGAATTCTAGTTGAGTCATATTTCTAGCTAAGTTTATTTCTAAATGAAATAAACAAAGCGGGTAGCATGCTACCTATTTCTCTCAAAAAAAACAAAAAAAAATNTATATATATATTGAATTAGGTTGGAATACTATTAATAGTAAAACGCTTTTTTTGCTATCAATTTTTTAACTCTTGGATGAAAATTTATAGGGTCGGGATAATATTAGTTGGTTAGAGTTGAGTGGTCTCCCTAGGGTTGAATGGTTTCCACTGGGTTATAGTATTTAATCCAATGGTTAGAAATAATAAAAAGAGTTGATCCAAAGGCTAAAAAATTGGTAGCAACAATGAAATTTTGCTACTAATAGTAGCCCAGTCCATCTCTCTCTCTCTCTCTCTCTCTCTCTCTCTCTCTCTATATATATATATATATAGAGTAGAGCTACTATACTCTTATGAGTATTAGGCCCTTCGTACTCATAAGTTGTTTTCGATAATGGGACTTCCAAATCGACGATCTACTCTGTTAAATATGATCTAGAATATTTGAAACTTCTAAAAAATAAATTTCGTAATTTTTCAAAATCATAATAAATTCCATCAAGCAAGTATAAAATGAACAGTCGAAATCAAACAACGTCCTAAAAATGGATGATCGGATTCTTCAATTTAAGATCGGAGTTATTGATCTTTATCTAGGTAGTGAATAGAATTTTTTATTAAAAATTCAAATGATTTCGATTCTTTTAGACCGTTAAACTAGCAAGTATCCCATACGGCCGTTAAAAACTGTCAATTTTGTAACCTTTTGATCGTAAGGTAAATGATGTCGAAAAATTATGAAATTTGATTTCTATAAGTTTTAAATGCTATAGATAACGTTTGACGGTATGGATCGTCGACTCGAAAACTCTATCATCGAAAACAACTTATGAGTACGAAAGCGATCGTACTCATAAAAGTATAGTAGCCGGACTCTATATATATATATTGGTTTTTATTAGTGCCTTATTATTATTTTCTCATGCTTTCTATTTATTGATTATACCCCCAATCATTTCACATCTCTTTTTTTTTTCTTTTTTTTTTTTTAATGCAGGCCCTGTGCTTACCACACTTGGTTGATGGATCAACAAATTCGAGGCAGCTTGTAAGTAGTTTTTTTTTTTTTTTTTTTTTTCTTTTGTGATGATATGCAGAAAACTATTTTGAACATACAAGATGAAAATTCATGATGCGTACTTCATCTTTTTTAGCAAGCAAAAATGCATATGGTTTTGAAGGTTTTCTTTTTGGTACGTTAACAGTTTATTATTCCTTTCGGAAATTGGTGAAGTATGGAGCACTTTTGTTACAAATCAAGGAATTACAAACCAGTTTTGATTATAAAGAGAGTAAAATTACCACTAAATTTTTTTTGTTAGGAATAGAAAAAGTAAAGATAACATTAACTGCTCTATCCCTTAAAAAGTGAATAATAATAGTCATTAACTTTCACCTTTCAAGAGGTAGAAATAGAAAGTTGTTCTGTCACCGACCGTCCCTAGAGCAAGTGGCAAAGAGCTTGGTGGTTGGTACCCGAAACCCAAATTCGAATCCTAGTTGATTCACATTTCTAGCTAAGCTCATTTCTGAATAAAATAAACGAAGCGGGTAGCGTGCTACCTATCTCTCTCTCAAAAAAAAAAAAAAAAGAAAGTTGTTCTGTCGCTATCAAAATTAGATACGTGAATATCAGCTCCCCGTGATTAGCCACGATCGCCAAAAGCATTCAATTAGATTACTATAAAAATCTAACTAATTAGAGTAGATTCGTCTCAGGAAAAAGATGAGAGGAATGACGTTTCTCATGAAGTCCAGTTCCAGGTAAATATTAATTCTTCGTTTCATTAAATTTATGGCCCCTTAAAAAAAGTTTCGCACCAAGATTCATCTTTTGCAGCCTCTTTTTTTTTAAAAAAAATTCAAATATGGAAAAATAAGCATGATTGGCTTTTGACAAGAAGATTTATCAGTGCTTTGAAGCCTGTGAAGTTCTTCATGCCCTCCTTTTCTGTGCTTCTTCTTCTTTTTTTTTAACAGCAATTGGAGAATAATTTCATGGGTATTAATGTGTGGATTGTGAAATGCTTTTTCTTTTTCTTTTCTTTTTCTTTTCCTTTTGTAAGTGGTAATGGTTGGCACCAGATAAGTTAAGAATTAACTTAGTGTTTGCTACATCAATCACAGGATCAGGATGCTTGTGATGAGCCAAAGAAGGATTTAAGGAGTAAAGGATTGTGCTTGGTTCCCATATCCTTAATCCTGTATGTCGACAGAGATAATAAAGCAAATTACTGGGGTTAAGGGAATTTGTATTTAAATCCAAGTTTTATGTACAAACATGTGGAAAAGATTAGTAGTAGCAACTTCAAACTTGGGACTTCGCTATTTAACTTTGCGATTCCATTGCATGTAATTGTAGTTTCAGTAGGCATCTTAAATGTTAATTCATGGTAGTAACATGTAGCTGGGTGTATAAATTTAGATTATTTTACTCTTAAAAGAAACGAGGAGTTTATTGGGTTCATTGAAAGATAACATTTCAACAATCATTTTTCTGAAGAACTATACAAGCTACTTTCTATGGTGATAATAATACCAAGCTTCGTCAAGCATTTCCACTTCTTAATTATTTTGGATAGATATATGGAGATGATCTGGTGCTTCTAGGTCTTTAGACATAAAATGATGTTCCAACCAGAACTTGGTATACGAGAGATAAGGAAAAGATACAACATAAATAGCTAGGTTCAATAGCTTAGTACGTGCATAGGTATTTCTACACTAACTTTGCTACAATATCAATAATTAGTTATCTGGTAGATGAGATGATGCTAGAGGAAAAAAAGAGAGAAAGTGGGAGGGCTTTATTCGAGCGCGACTAACCCAGTTTGAGGCCAACTGGGTGGGAGGATTGACCAGTTGTAAGCTACATCTTTTTAATAGCTGCATTTATGATTCCCCTGCCAATAATTTCTTCAGCTCTTTCTTGTTCACCTGTAAAGAGAAAGTCAGTAAGCCGCTTTATGAAATAAAAAGGAAAAATGAGGGGGGGTGGAAAGAAGCAAGTTTCGGCGACAATTATTGGAACCATATATCTACCTTTCCCATGGCATTTCGAGGAAGCGAGTCCCACAGAAACAATCGCGTTGGTATCTGACACACAAATAAGAACTAATCAGATATATGCGGTAAGCACAATGGAGCTTTTATTGGATTCGTGTACAGTATATTTGATGATGCACCTTCGAGCCAAAACGGCAAAGTAGAAAAGGTTACAATGGGTGAAACTAACCCTCTAAATGTTGTGATTTTCACTCGGTCACCTAAAAATATAGAAAGCCGCTATACTACCTCCTGAAACTATGATTTTGCATCATTTAGGCTCCTTACACTAGCGTTCGCTAGGCTATATATATATATCTCACTTTGCTCATAGGTCAATGGGGATGGGGTACTATGTATAAAATGAAGAATGTAGGTTTAAGATTTTCTGCGAGGAGGGGAAGCAATGTGAAAATCACAACTTCTATAGAACGGCTCATGCTTTATAGCCTTACTAGAGAGGAAATAATACAAGTGAGCAGTGCACTACAAAGGATCACGCAAGAGAGATCTGCAACTAAGATCATCAAACATCAGCTCACTTCAGGATTGCACTAAACAAAAGAAAACTTCTAAGTGAAATGTCAATAACGGAAGATTGCTTGTCTATTTTTGCAATTTTATCTACAAGAAAACCTAAAAGAGAATGGAATATGAAAGTTTTATGCAAATAAGATAAAAGAATTGTCTTAAAAAGAACCTTGTATGGCGCTAGTCTCTCCGTGGACCAGCTCCTTAATTCCTCCAAGCTAAGGGCCGGTTTAAGCTCTTGTTCAGCTTTTTTCTTTGCTTCTTCCTGAGGCACAATTATTGCGCAGACAACTTCACCGTAGTCCTTGTCGGGTAAGCCCAATACGCAGCACTCTGACACGGCCTCGTGCTGACAAAAACAAATTTAACCTATGTGAGCTCAGTCACAGAAGAATCAGAAAGTTTTTAAACTTCAATAGATAAATAGGTAAATACAAAATTTATAAACTTTACAAGTTTGTAAATGCCCGTAAAGGGGTATGCTCTGATAATCTTTTGTACGATTATGGGTTAAATCTCCAAATTGATTTTTCTTTTTCATTTCTTACCTTCTCGGCGTCATTGCTAACTAAAATGTGGCTCAAATTTATGTAATCTATGCTAACTACAATGGTAACATATTCATGACAGTGCACATTTTAAAACAAAGGAAATGGGAAGATTGGCTGGTAACCATTTATAAGAAAGAGTACTTTATGACATGTACCACTTGCTATGACATGTACCACTTGCCTCTAGAATTACTGCCTCAATTTCCAATGCAGAAAGTTTATATCCACCGACTTTCATGATATCAGCATTAGTACCTGTCCATTGGATTACCAGAATTAATGAGAAAAGGAGAGTGAAAAAAGCAAAAAAGAAATAATGATCTCATCGTGGCTCATAAATAGTGGCAAAATGTATAAATGTGAATTATCAACTAAAACATACTAATATCAACTAGAACATACTAGAATTAGTTTGATTATTTTGTAGAACCATCAAGATCTTAAAGAGAAATAATGATATGATTCAGTACAAATATTTGCGCAAGCATCAATCTACTTTTTGTTTAATGCCTTCCTTTCCTGTTTTGACTGGACTTTTGAGTATTATGTTTGCCAATTCTACTTGTCACTCTCAATTGGAATATCATTACAAATATTTAAAAAATTTAGAAAAGAATGTACATAAAGTAGGGAGATCAAAGAATCTAGAACACATATTCACATTTTTATAGACTAAAGTGCTCCACAGACTTATTTCATTGAACTATCATTCATTTTCAGCAGAACTAGAGGCAATAATAATATCGCCAGCAAGACCATTCACCACGGTGAAACAATTTTGAACACAAAAGCTAATTGTAGATATCTAGTATATTATGTAGTAGCGTCTGTAATACACAACATGATGCAAAAAGGAGTGCAAAACAGGAGTAGGAAACTAACAAAGCTCACTATAGTAGAAGCTTATATACTTTATACATAACCAGAAAACTGACAGTTACTCATGATAAATAAGTTCATTATGACTGAGGAAATATCAAATCAACGAAAAAGATGAACAATTTATGGAATCGATATAACTTACGTCCTAATATTATGTAGTAGCTGTCCTCATCCACTGTAACTGTATCACCAGTCTTGAAGAAACCACCGTCAATAAATGACGCCTTTGTTATCTGAGACCAAAATCGTGGGGGAAAAAAATGAAACATGAACACAACCTCAATAAATTTCATTAGTTAGAATGCCTGTTCACAAAAAATAGGACAAAAACAGCAGAACTGATCATACCTCTGGTTGTTTCCAATATTCTTTAAACAGAGATGGACTTCTCACACAAAGTTCTCCCACTCCAGTTGTAGTTTCACTGCCGTCCTCTGTAATTATCTTGACCTAAACTTCATTATATAAAGCTATCATCGGATCTTCATGGACAAGTAAAAATAAAGGAGAAGCATGTAGACGATTAACAAAAAATTAGAACAATGCAATAACTATTAAGATGTACGAATCTAGTAATGCCCTCAAAAGTTTTTCAACTCACCTCAACACGAGGAAGAGGTTTGCCAACTGTGCCTCCTTTTCGGATACCATGCAATGGATTAGACAGCGCCATGACAAACTGTTAACATCTACTCATCAGCATAAACTGTAAAACTACTATTCATATGATTTTATCAAGGCTATGAAGCAGGTTGCTTTAAGGTTAGAGGTTCAGTGGTATGAAAACTGACCTCTGTCATTCCATAGCGTTCTAAAAGGCGATGCCCTGTTATCTCTTCCCATTGTTTCATAATTGGGTATGGAAGTGCCGATGAACCACACATCTAGAGAAGAAAATTTTCAATCACATCAAAATTATGAATATATCTTCAAAAGACTGTTTGGTCTCTGTAACACCATTCAGAGCAGCTAGCGAGCATTAACAATCTACAGTAGGGTGAGCATTGGTTGGTTCAATTTGGTATTTTGGGTTTCTTTTTCTTTTCTTTTGGTTACAAACCGAGCCAAAAACCAAAATCATAGACATAAATAGACAGAACCATATTGAAAAAATTGTTCTGGCTAGGTTGGTAAAAATTTGTTCTGGCTAGGTTGGTTTATTCTGTCTGGATTTGATTGAATACATCCATTGTCCCTTCTATTTAAAGGGTATTTAATCCAATCCTATCCGTCAAGCCTTTTATACGGATGTAGCGATTAAGCACTACAGTTGTCCAATTATTAACATGGTATTAAATTATTATTATTTGGTTCAGTTTATTTGGAACCAACC

>Aco009603; *Ac*bHLH2
GGAAAAGCTTACCCTTTCCACAGGTTTAAAACGTTAATGCTCTCTTTCTTCTCCCTCCTCCTCGTCGCTTTGGGCGAAATCGGGGAGGGGTCGAGAATTAGGGTTTTAGCTTGGGGTTAGAGTTAGGGTTTCGAAGCGAAGGGGATCGGAGGAGCTCGGAGGTGGAGGATGGTGGTGGACAAGGGGAAGAGGTGCAAGGTTGCGGAGCGCGGAGAGGAGAGCGATCGCGTGGATGGTGAGCTTGTTCCTTACATAGAGAAGCTCCAGGAGATCCAGGACGAGCTCGAGAAGGTTGTAGATTTTGTTTTATTTGTAAAACTTTAAGCCCTTTTTTGTATGTTTAATTAGTTCTTAAAGCTAATTTTTGACATTTTATGCTGGGAAATAAGCATTAGTAAGATTGTATGCTTCCGTAGGTGTTTTCTTAGGTTTGCGACTTGATTATTACTAGGAAAAGTTCTTCTTACTATACAAATATCACTTTTTTGCCTATATCATAACTTTTACGTCCCTAATTTACATAATTCTTTGCAATGTAGCAAAAGGTATGGTTTTGTAAGTTGAGAGTTACTCGTAAACTCTTTAATGATGAGGCATTAGCTCAGAAGTCATTTTGCATTTCATTTTTGTTTCATTGAGGCGGTTTAAAAGCTATTTTGCCTCGAAAGTATAGAAATATACATAATATGTAGAAGGGTTAGGCTGCTTGGACCAAAATGCAAACAATTGAAATTTCAGAGACTGAAAATCTCCGTAGGCTCAATTTTAAACTGAGCCAAAACTGATAAAAAATTTAGAATCCGAACCTCTTCGCCCATGGCAACCAGCCGACCCTGCAGTCCCACCAGGAGCTGATAATCCTGCATCACAGTCTTTACACTCGAGAATATGCACAGTATGTAGAGGTGGGCATCATGTCGTGTATTTTTTAAAATACAGGTCCTGAGAGAAGTGGCTTGGTAGGGCATGAAAGACTTGATGTGTCGCTCTTCAAAAATGATACCATAATTAGGAGTTTAATTGGTTTCCAAATAATTTTGGCGTAGTCGTTTTGTCATGGCTGGATGGATTCAGATTGATTATGAAACCCCTTTTTATTTTCAGACAGTTTGCTGTCGTATCTTATGAACTCTATGTAGTTCATTGGCTCAGGTGGGTCTTATGTACAAATTTTCTCCGCCTCTATTGCTAGTTAAACATTTCAGATTTGCTGTTAGTTATTAATTTTTCTTGGTATTACCATATCTAAAAGAGATTAGGTTCTGAATATTCTGGTGGGAAATTCTATTTTTAGAAACTTCTAAATTCGTTAAACAGAAGGATGTGATATTTCATAATCTTATAGCTGATATTCTTGTCCTGCATGGCACTGTATGCTTTGCAGGTCAATGAAGAAGCAAGTGACAAAGTTCTAGAGGTGGAACAGAAGTATAATGAGATACGAAGACCTGTCTATATGAGAAGGAGCGATATAATCAAATCCATTCCAGACTTTTGGTTAACTGCAGTCTGTACTTCTCATTTGTCCCTATCTGTATTTTGCTAGAAATCTTATCTTTTATTGGCTATCTGATTTGAATGTTCCTGGGATTTCTTCTAATGACAGTTTCTAAGCCACCCCGTACTTGGTGATCTTTTGAACAAGGAAGAGCAAAAGGTCTGCCGTTGAATTTCGCTATCTTTGCATAGCTCAGTCAGTATTTACAGTTGGCAGAACTGTCACGTTACACATGAGACTGTTAGATTGAATCATTTTTTTGACATTACATGTCATATTTAGATTTAATCATAACCCTGCATGGCTGTTTCTGTAGTGTTTTAATAAATTTTGCATTCCTGTAGATATTTAGGCACCTCATTTCACTAGATGTTGAAGACTTTAAAGACCTTAAATCAGGCTACTCCATAACCTTTGTGAGTGTGTTTCCTTTTTCTGCAAGTATTCTTTCATTAGAAATAGTTTATGAGGTGGTTCTTGCGGTAAATAACATGGATTGTTTACATTTGTTTTCGAGCTATTCAGAATTTCTCCCCTAATCCATATTTCGAAGATACATGCCTAAGAAAGACGTATTCTTTCTCTGACGAAGGAACAACTAATATAACTGGCACAACTATTAAATGGAAGGAGGGGATGGTAAGGGCAAATTCTTCCTTATGCTCGTTTTTCTGTTACAATACATAGTTCTGTACTCTGTTTTAGCCTTTCCGGCCCTTATATCTGCAGAATTGTGTGAATGGTGAGGCTCGTGAGAACAATGGCAGCAAAAGACCTTTCGGTGAAGAAAGGTCAGATTGTGATGCATTTTTTATGTCATTTATATCAGCAACAAAGTGCATGCCAAGTAGTTCTCACTTTACTCTATATCAGATAAATGGTTCCATTATGACACTTTTTACATCTAAAAGAGGTGAATATATGAATCTTATAGTATGAATTAATTGCACTGGAGTCTTCAACGTTTTGAAGTACTCAAACATTTCAATTGTTACAATGGTGTTCCCAAGAATGGTACAAGTAGCTAATAATTGCAAGAAATATTGTTCACAAAAAGTGTTAGTTTGGAGTTGGGAATACGTTTGTAACATGTACGTACATTTACATAATGACATAATGCTATAAACATGTGCAGTAGATTTTCAACTCCCGGAAATTTGAAGTACTGAATCAAATACTTATACTAGTATTTAGTTTGAAGTGCTTCCTTTTTTATGCAGCTTCTTTAGCTGGTTCAGTGAAACTCATCCAAAACGTATGCCGGAAATGTTCTCTGATGAGGTTAGTCGGGGTTTATTTGCATGAAATTCACTGTTAATGTGCCTGTTTCCCCAGGCATTAGCTGCTTCTGTATTCCTGGAAGCAGTTGCTCAATGCCTGCTCGAAACATTTCCCAACTAACTGTTTTATGTCTTTAAGCATGCATAGACTTTGTATTTCAGCTGCAGAGTGAAGCAGTTAGGGGGGCTATAATGAAAAAGCTGTTGTGCTTCTTTTAATAGCTCTACTCAAAAGGGCATGGATCCCAAAGCTCCATTCGGCCCAAAGAAGCCCAGTTCATGATTACAGAGGAACTAATAATTTATGCTTCTGATGTCATGCAAGTCTCTATTATGGGAAAATAAGCATCTATTATCTTGCAATCTTCCATCATTTGCCTTTCTTATTAAATTTTCTCCAACATTGCATGATAATAGATGTAAATTTGTCAGGACAAGTCATGCACATTAGCAAATGTATTCCGCTAAGGGCTTGTTTGGTTCAAGGGTGGAATTGGAATGGATAATGGAATGAGAATGAATTGGAATGGTAATTGGAATGGCCCACTCCACCAAGACGTTTGGTTTATTTGTGGATTGGAAATTGGAATGAGTTATTTACATTTCATTATTTTGTTCGTATAAACTAAAAAAATTATAGAATACTGTAATACTAATTTTACTCTCAAATATAAATTTATATTATTTAAAATTTATGAAAAATATAATACTATTCTCTAATAAGTTTTCCAAAAAAAATATCAAATTTATTTTTAAAAACTTTTATTGATGTGGGTTAGGGATAGGGTTTTAAGAGAGGATAGGTTTTTTTTGTTTGATGAGAAAGGGGTGAAGAGGAGGGTGAGATGGTCACATGGGCTTCGAAAACTTAGTGAGCTTTCAGAATGAAATCTCAATCCGGGTTAGAAGACCGGAATCAAGTTGAAGGCTAATGGGCCATTTCCATTCCGGTCCGGAATAGAAATTCCAAACCGATTCATATGAACCAAACAAAATTAACCGGATTTGATCAAACCGATTCCCATTTCATACCCAAAATGGATGAACCAAACAAGCCCTAAATATAATCCGGCATTAAAAATCTCTCCCTCAATTCAAATATGCAGATAGCTGAGATAATAAAGGAAGATTTGTGGCCTAATCCTCTAAAGTACTTCAATCATGTAAGTTGTTCTTCTTTTGGCTTTATTTACATCATTCAGTGGACTCTCCTGTATGCAAGGTCCATCACACGTTCATATATTTGCGTACGCTTATTTTTATGCTGTAAAAGCATGATGTTACCTGTAACGTTGTATATCTATAGATATTCTCCGAACTGCACACATTTGACAATTTAACCCAATGTTCTTTACTTCTATATTTGTCCCATTAATACAGGAAGCTGGTGATGAGTTTGAAGGTGATGAAGATGATGATGAGGTAGTTAGCTCTCATTATCAAACTATAAAGTGTATACTAGTTAGTAACGCGCTGTTTCTCGCACTGAACTTATATTGTTAAGTTATTTCTCCTCTTTGAGTTGGCAATTTCTGTGATACGGTATAAATTTTGTTAGCTGCATCGTACCGGCAGCCAAAGTTGATAACTTTTCAGTGTCTATTTAAAGAATGATGGGCGAAATGTATTCGTTTGATGTTTTTTTTTTTTTCCTTCTATTATGCTCGTCATGCTAGAAAAGATTTACCTGACCGAACTTCACTCCTCAGAAGGGCACCGAATTCGATGATGAGGATGATGAGGACGGCGACAGCGACGATGAAGAGGAGGATGAAGGCGACGAGGAGGATGACGACTAGGCTTGTGTTAAATGAGCCTTCAGTTGCAGCAGGGCTAGATTATGTTGTTTGTTATGTTATATTCGAACCTATGCACTAGGTTTGTTGCTTGTCTCGGCGAACTTAATTTACATACATGTTAGATGGATTAAGCTAGTTTGAGGTGTGACAGTATGTATAGCCTCTTGGAGGCCTTCGTTCTCGGAAAAATGGTTTTATGAAGTGCATTTACTGGGGCTATTCTGCTAAGAACACTGCTCTTGGTGCTTAAATCATGTCCGGATCAAAGCCCCATTTATTAATTGCACCAGATTCTCATGCACCAAATCCAATATGGATTAGCTTATTCGATGATACAGTAATGTAATCTGCAACATCAAACGAGGTTGCGAGTAAATATTATTAGTTCTGCCTTTTGTGGTGAATTTTTTTTTTTTGAGAGATAGGTAGCACGCTATCTGCTTTGTTTATTTTATTTAGAAAAATAAATTTGGCTGAAAATATGAATCAACTAGGATTCGAACTTGGGTCTCGGGTACCAATTATCAAGCTCTTTGCCATTTGCTCCAGGGACGCTGGGACGGCCGGTTTTGTGGTGAATTTTTAGAGGCACGACAAATGGTACATGGTGTTAACGGGTCGGATCCTGATGAGCGCGAGTCAGAGGAGGCCCGGATCCGAGACCTAGGGACGGCGTGGGGCACGCAGCCGAATCAGAAAACGGGGTCGGGTGTGAGCCGCGCACGCGCTCGCGCCCTACGCGGCGTGGGGGTCCACGCTGGCCGCTTGCGGAGCCGCGTGGGGCCCACGAGCAGCGCGCGCCACGTGGCGCGGCACGCGTCGCCGGGGCACGGATAGGGTGGTGGCCACACGCGGGTGCGTGGTGCCTTCGCGCTCCGCCCGGAGTCGTCCTCATCGTTCCCACCCTCGATCCCTACCAAACTCCCTTTGCGCATCAAAATTAAATAGAAATGCGAAGCCGAAGATAAACTTAATAAATAAAAATTAATAATGGTAGTAACATGATAATATCACAAGGAAGGAGAAGAAGAACCTCAAAAATAAAAAAAAAAAAACCTATCTCCTGCTCGATCCCTGCTTCGCCGATCGGTTTATCGTAAGGTACTCTTCCTCGATCCGTAGTCATCTTCGATCTGCGCATCAACTAGATGCAGATCTGATTCTTGGTCTCTATTAAATCCTGTAAATTATACACCTATTTTTGATTGCCTGATCTGTTAGATCCCTCGAAGAGTTCTTTTTTTTTTTTTTTTTTTGCTGAAGAACGAGTAGTAATTAGTGTTGTGAGTTCGAATCAGATCTAAAAGTGGAGTTCGTGATGTTTAATTGATGCAGGGGTTCGCAATCTGAGCATGGTTTCAGAGTCTGAGCAATCTTCATCGATGGCTAATGGAGAAATTGTTGCTGAGAGACCGGCTGGGAGGTTGTTCTATTACTTAAGATTGCCATAAAAATTGCATAATTTATGGATCACATTTGTTGCATTGCTCCTAGAAAATTTGTCACGCACTAGTAGATGTGACTAATTATCCTATATCTATAATCCTTCAGCTTTGGCTGGCTAATAGTGAAAACTGATTATTTATGCTTCTCGTGCGATGCTATCTGTAAGCACCTACAAACTATAATATATGTGATTTATAATATTCCCGACTAGGAAGATCAGAAACAAATGAAAACATGCAGATTATGGCAGAGAGAAATGTAATACACCCTATACGCATTATTTAGAGTGCTTAATGATCAGCTGGACATGTCTGTAGTGATATGGATTTGTTTGAGCGGTAAGCACCTCGACGTGGACTTTGATATGTTTAATTGAATCAGACCTATAAACTATACAGGTTTCATCAGCATAAGTGGCGGAGAAGGACAATGCATCTTACATTCAAATTTCAAGTTACTTAATGTTTATATCTTGATCTTGATTGCTAATTTATAAGTCGATTCATCTATTGCGAATTCACGATAATATTAAGACTAGACTGTAATTTAGTTCTTGGGAACGAAATTTACAGGGTAAATGTTCAAATTTTTGCGAACTTCTGCCTCTAGAATTGCGGCATTTTTATCTTTCCAATGCTGATTTAGCATACCGATTGTTCTAGCAATATCTAAACCTTGAGATCTTTTGTCACTTTGGAGTTTCTTCAAGAACCCATAACAAGAGAACAGCATCAAGCAACAGAGTATATGTCTGGACTAATTAATCTTTCGTTTCCTCGATCTTTTATGTTCAAACGGATTTTGTCAGTTGTGAAAAATAATAGATGACTCAGTTATGCTAGTTATCACAGACATGCAGCACACTCTGGTTTCTTCATATCTTGATCTTTGTTAGTATATATTACTTCGTTCTTTTCCTAGTAACTAATTTTCTTAAACCTTTATTGCTGCTTTAGATTTTTGATGTTTGAGAGCTTTCATTATGCAGGTCTGTCCCCAGCAAAAAATCCCAGGCACAAGTTCCAAAGAAAATTCATAAAGCTGAAAGAGAGAAACTTAAGCGTGACCAGCTAAATGATCTCTTCTCTAAACTGGATAAAATGCTAGGTAAATATATATGGATCAGTTGCTAGTATACATTCTTGTACAAGCTTGGTCTTTCCTTATAGAAAGCAATTAGCACATTGAAATGGTTACATATTAATTATTGTGCCTGATTTATTTGTACCCCCTGAATTATTATGGGTAATCACAGCTTATCATTGAAGTTTGACCTGTATTTTCATTTTATCCTAAAGAGTTTTAATCCCAGGAGTTTCAAATTACTTCTTGAAGCTTGACATCAATATCATTTACCTCCACTTCAGGCCCTATTGTATTCCCCGATAATTTGTATGAGAAAATCGAAACTGCGCCAAATTTCAGGGATCAAATTTGTTGTTAGATTTTTACGTTTTACAAACTTGTCCTGCTCCGGGAGAAAATGTTAGGCTTTTCTCTTGCTGATCAGTATGAACTATTTATTTGCAGAACCTGATCGACAAAACAATGGCAAGGCATGCATATTGAGTGATACTACTCGAATAGTGCGTGACCTCATTGCTCAAGTAGAATCCCTCAGAAAAGAAAATGCTGCTCTGCAAACCGAGTCGCACTATGTAAGTTCCTTGTTCTGAGGTTTATGTTCATTCGTAATGGTAGTGATTCTATTCTTTGGATTTTCGTTTTCATATTTAATTCTACATCATTTTTTTAATAGAAAATATCTTTTTCTTAATGGTAAAAGAATTGGCATATGTTTTTAGGAGAATTTGAAACCAAAATTAGAATGCGGCATTAATGTAGTTTATATATTCTCTACAATCATGTTATTATTATTATTATTATTATTATTATTATTATTATTTTGTTTAATCATGACCATGTTTCAGCATGCCTTTTCCTTTGGTTATTCAACTATGTGCAGCTATATACTCGTGTAGAAACTATTTTGTTATTTAAGCATTCAATATTGAGAGATGGGGAGATTTTCTCTGATAACATTTTTTTGATGAATGGTAAGCTCCTTACATATTTTAAAAACAACATTGCATGAACGACGTCAAAATGTTCAGAAGCAAACAGAGCTTGACTAGTCTACTGAGGTACAAAGCATCCTATATTTTCTTCTCCCGCATTGTGGATTGTTAAGAATAGGTCTTGCCTAGAGCTTTCATAGACCCTTGTGAATCTCATCATAGCTAAGTAACTAGCATGCCAAATTTAGGTGACTGGAACAGAAAAGGTGAGGAAGTTCATGGAGATGGAAGAAAATGGTATACTTGAAAATGAATGTTACATTTGTCTTCCATAGCATACAAATTTTTAAACTACAATAGGAAAAGTCAGATGCTCTTAAACGCGCATTTGTGCCTGCATGAGTGAGGGTTTTAACAAGCAATTCTACGGTGAGGAAAAAGAAAAGAAAAACTCTAAAGAAACCATTAGGATTCATGGGAGGCCTAGCAGGGATGGACAGGGAGGAGAATTAGCTTTGCTGTTCCTCCCTGGGAAATTGCCCAATCATTTTACAAGCTCCTAAGTTTGAAGAATGTGCGTTTGTGTTTAGAATCCGAAATGTGATGCTGCGCCATTAGCACTACTTGAGCCAATTCATGAAACCGTTTTCAAGCTTGGTCAGAACATCTATCTATCTATTGATTATATCTGCGTTTTCACATTTCATAATTTTTAAGCCTATGTTTTCATTCGGCTGCACGGGTACTTATCTCGCCCTGGTCCTATTAATTTTACTTCGCTAATCTTCCTAAGCGCTTGACGCCTTTCTTATGTCGGACGTTGAACAGGTCACCACAGAAAAGAACGAGCTGAGGGACGAGAACGCCGTGCTCCAAGCCCAAATATCCGACCTCCAATGCGAGGTTAGGGCGAGGATGGGGTACAATGGAGTTTGGACCAATCCCCCAAATGTAAATACAATCAATGCGCTGCATCCGACGACAAACCCCATGCTTCAGCAACCGCCTGTCATCGAGCGAGCTTTTACCACACCACCACGAGAACTCCAGCTGTTCCCCGGGGCAGCGGGATCACCGGATCACGAATCACCGTCCCCGCGTGTTAGGAGACCGCATGCGAGCTACGCGACACCATCCAACACCTGGACGGAGCGGCTTCTCCCAGGCCTCAGAAGAGAAGCACAGGAAGAGGAACAGCAACAGAGTAGCAGCGGGATCACAGAGAGCAGTAGGGAAGATAGGGTTGGTTAGTAGTGCTTGAGCTAGGGGTGGTGGTGGTTGGCTTGTAAATGATTAGGTGTTCATGAGTAAATTGTTATTTTGCTGGTTCTGCTAACTAATGTTAACGTAGATATATGTTCAGTTACACATGCTCTTGTAATGCTTCACAATTATTTTAGTTGTTGCAACTAAATCCTTGAGAAAGGAACGATTAATATAACAGATGATGAAAAAAGGAT

>Aco009661; *Ac*bHLH3
ATGATGAGGGAGAGGCAGAGGAGGGAGAAGTTGAGTCAGAGCTACGCGGACTTACACTCTATTCTTTCGACGAGATCAAAGGTTGGACTTCTTTTTCTCTTTTTTCTTTTTTGAGGTCACTTAATTGGATTTATGGCTTTAAGAGCACTTAATTTTTAAATTTAAGATGGATAATTAAGGAATTAGAGGAAGAAAATGGGTAATTTGGAGAAATCCCCTTTAGCATTGTGCTATTATGAAGTAACATTTTTGTTTGAAAAGATGCAGTGGCCTGATTCTCTGTAATTCGTATAATTGGAGCAAGATAGAATTATTCCAAGCGTGTTAAAGTTTAATTAAAGGATAACTTCAAGATAATAAAGCTTCAATGTGGCTATCTAAAGAAAATTGTGGTCTTAATTACATTTCAGATCACTTTTTACCCAAATATATATACATAAAATTAGCCAAAATCTAGCTCGGTTAATCGATCTACTTATATTACAAAGTTAGTGCATTTGGCCACAAACAAGGATCCTGCAAGGCTAACATAATCAAATCATTCACCCAATTAATATCTTATTGACAACTTCGCCAATCTAAGCTTTGATTTTACTCACAAGAAGTTATTTTAACTTGTTATAGGGCGATAAGAACTCCATCGTCCAATCTGCTGCATTATACATTCGCGAACTCAAAGGAGTGAGGAATGAACTCCAAAGGCGCAACGAGGAGTTCAAGGCGAGGTTAGTAGGGGATGATGCGAACGTAGAAGGGGTAAAGGTGAAGTTCGAGGTGGCGAATCCATCATCCACTATTGACTCGATGATCGGAGCTCTTAGATGCTTGAAAAATATGGATGTAAAGGCCCGAGCTATGCGGTCTAATTTCTCTGGCCATGTTTTGTTGACGGTCATGAGTATCGAAACAAAGGTGCGCATTGATTGATAGCAGTTCCTCGATGAAAATTTTACATTCAAAACTACTATGTGGATTATATAAGCAAATATGAAAAAAGTAGTTTCAAAGAGAAGATTAATAGATCCGTACAAGTTGAACTTTCCTTCTCATTATGTCTAGAGTATGAACTAGTTGTAATAATTTTACCTTACTCAAATGTTTAAGGGCCATAATAACATTAAAAATGCAGATAATTCTCCTGAATAAATCACTATATGTATTTGATATTGAAAAATCTTGCACTTCACCCTCTCTCTCTCTATATATAACAGAAGGGATTAAGTGGTAAGCTTTATATGAATTAAACGTAGTGATTACGGATGCAAATTTATTTATCAAGGATAAAGTGCAAATGGTGATCTATCCAAAGAACTACGTTAACTATGAATGTTTCAATTTTATTAAAACTTATCTGATTTTTCATATAATTTTTGTGCGTAGATGTCGGTTTCGGAGGTGGAAAAAGCAATAGAGGGAGCTCTAGCTAATGCAGAGACTAACAAGAACCAATTCCCCTTCCATGGAAGCGGGGGATGGGCACTCAATTCTCATGTGGAGAACATGACCTAAATTTTACCCCCCCCTTTTTTTTTAGTTTCCATGTTT

>Aco011419; *Ac*bHLH4
ATGGGTATTAAAATGTTATGGCTAACAGGTATGACGACCACAAGAGGGATCGGAAATTACTCTCAGGCAGGTAGTGATGCCGTCCATGCAATGGCAAATAGCAGGTTAAGGTCTCAATGGAGCTTCTCGAGACAAGATTCAGTACTGTCGCAGATCTCTGAGATGAGCATTCCGGAGATAGGGGAGAGCGGCAACAGCTCAGACGAGGCGACCGGTCATGCGGGACAATCTTACATATCTAGCAACTTGCTAGGTGCCTCGTGGGAGGATACCAATTCAATCATGTTTTCGTCTCCAGGCAAGCGGGGCAAAGAGAGCAACGGGGATGTTATTACCTCTCTCAGTAACTTTGACTCTCAGGTAAATTCAGTGATTCGAAGTAGGAGATTTAGGAAAATGTTTCTAGTATGAAGATATGATATTGTTTTTCTAGTATCTGGTTCCATCAGAAGTATTAATGAAAACCATGTTTAGGGATCTGCTGATCATTTCTACACTGCTGCCACTGCACAAAATGCAAGTAAAAGGGACCCAGTTATAACAATTGATAGTTTGTGGACCAAGCTTAAATTATGCTGAATGCAAAATATGCCCTCTGGCCTGCTATATATTACTTTTTCTAATGGAGAACTAAATAGCAAGTCGTCTGCTTGTCTTTCGAAAGTAAACATTTCCCAACTAGGAACAACTATCAGACTGCTATTTGTATTTTCATTGTGTAGCTTATATTTTGTTCGTCATGGTTTTCCATTGCAAGACATCTCCTGTTTTATCGAGATTAAACTAATAGCATTTTCTTGATCAATCAGTTTGGCATGCCGAAGACATCAGCAGAGTTCGCCGATATGGAGAGGTACCTACAAATGCACCAAGATTCTGTCGCTTGCAAAGTCCGTGCTAAGCGCGGATGTGCAACACATCCCCGGAGCATCGCTGAGAGGGTTTGTTATAATCGATCTGCAGTTTCTTTCCTCAAGTTTTTCTATTGGGTCTCCTACAGGGAATACTTTAATAACAGATGAAAAACAGTAATAAAGACTCTATTGTCTGTGTGGTATGAGTCTGATAGACCTATATATTTGTGGACATTTAATCATGGTCTTGTGTTAACAGCAATTTTGCTTGGAGCTTTGCAACTATACGCCTTTTTCTTTTTCTTTAATAAAAAAACAGCAATTTTTGTTGCTTGTTTCTAATAGATATGTATTCTCTCCCTTTGTAATTTTTTTCATTTGACGATGTTGCTCCAATTAGAAGTTGGTAAACTTTGCATTCTTACTACCATCCAGGAGAGAAGAACAAGAATCAGCAAAAAATTGAGGAAGTTGCAAGATCTTGTTCCTAACATGGACAAGGTAAGTAATCCTGGTTATTATCTCGTTCGAGTACAAGTCGACATGGTTGTGTATGGTAAATCATGTTGCATGTAAATTTCTATTTGCATGGTTTGGTAGTTGTGGATCGTAACCCTTGCTTACAAAAGCATCATGGGTATGGTTATGTAATATGATCCTTATATTCTGTTTCTTGGCAATGTACAGCAAACAAGCACATCCGACATGTTGGATCTTGCGGTGCATTACATCAAAGAGCTGCAGAGCCAGGTCCAGGTGTGTTAACATGGAGTATTACACTATGTGCCCAAAGTTTGTATTTCGATGTAGAAACTCTATTTCTGAGTCTTAGTTCCTTGAGGATATGCATAGCCACCTTTCTGACACAAAATTTCCTCCTGCAGAAGCTAAATCAAGAACAGGAAAATTGCACATGCACGAGCAAGAAGATTTGATGTTGAGCCAATTAAGCTATTGCTGAAAAACTCTTCAGTACAGTTTTGCTCCAACAAAAAGCGGGTGGAAGAGGCCTGTGATATCTTGTATCTTCTCCTCCTCCTTTTAATTCCCCTTATTCCATCAGTTTGCTTGTTGACTATAGGCAATGTTGTTTGGCTATAGGCAATGTTGTTTGCTGACACTATAACAATAAAAGTAGTTTTGAATTTCTACTGCAGTTCGGGGAAGCATGTCAGACTGTAATAGGTTTCTGTTTGAATTACTCCTTGTTAATGTGAAGAAACCAAGGTGGTGTTCTCTTCCTTGTAATATTTTTATTTGTACATACTCTTTGCTTTTTGGCATTATGCTTCTTCCTATT

>Aco012350; *Ac*bHLH5
ATGGAGAACGAAGGACTGGCGTTGGAGGCGACCTGGAGCTCCTTCGAGGAGTCGGAGATAATGGCGCAGCTTCTCGGCGGAGCCGTCGCGACCAACTACTTCCCCGGTGATCAAGAACAAGAGCCCACCAATGGAATGCACCTCATGTTTTGGCCTAACTATGATTCTGATTCCAATTTCTCCTCTCCCACAGATGTTAACTACAACTCATTCCATTGGCCTCAATCCATTTCCACTCCTTGTATCAGCACAAGCACTGGTAGTTTCCTTCTTTCAAATCCATGCTATGGAGGAGGATGCTATTTAGGAGACACCAACTTAGTTCCACACAACACAACTTCATCTTTCGATGTAGATTTTAACCGTCGATACTGCGACCTTCCTTCGATGACTGAGGAAGCATGCGATGACAATGAGTTAGGGTTGTCGATCGCGAACCAAATGAGGCCCGCTGTTTTTGCTGATGAGCCCTCGCACGCCGCCAAGCGGAAGTTTGGTTTGGGTGATAATGAGAAGCCCATTGAGGATGTGAAGGATGATGATACTTCCTCTTTGGTTCCTAGAAAGAAGGCCAGGGTCACTATGGGGGTAAGTCTCAAGGATTATATTTATAAATGCGGGTCTTTTTCTGCATAAAAAATCCATCCTTTTTTGTAATTTTGCATTTTTTTTTGTTTAAAATCTTTCCTTTTATGGACTTGAATGCCTCCGACATGCAAGAACAGCAAATAAATTTTAGTCCAAAGTTGCAAAGACCTAGGAAAAAAAAAAGTGGATCGAAGTAACAAAATCTAAAAAAGGATGGGTGTTTTAATGCAAAATAGTCATGGATATATGTTTTTTCTTTCACAATTCTGATCAAAGAATGTATCAATACAGGCGCAGGAAAGGAATAAGAATGCAGATACCAAGAAGCCCCTGAAAAATGAGGAAAATGGCAATGCCGACTTTCATGGACAGAGCTCGAGTTCGTGCAGCTCGGAGGACGATTCGAGCGGATCGCAGGAACAGATGGAGGGAGGAGGCAACACGAGCTCAAGATCGAAAAAGTCTGAAACTACTAGCGTTACCGGAAAGACGAGAGCCGGTCGTGGGTCGGCAACCGATCCTCAAAGCCTTTATGCAAGGGTAAGTCATTCATAAAAATGCCGAAAGCATTCGAAATCACTCTTCTGGTGTTTCACATTGCTGACTTTTTTGCTGAACCTTGTATTGCTTCAACAGAAGAGGAGAGAGAGGATCAATGAGAGATTGAGGATCCTACAAAACCTGGTTCCTAATGGAACAAAAGTAAGGAAGATGAAAATATCTTTAGTTAGTCACTAATTTGATTGTCAATGAAGAGAAATTTGATAATTAACAATTCAACTATAATTTGAACAGGTTGATATTAGCACAATGCTTGAAGAAGCTGTTCAGTATGTCAAATTCTTGCAGCTTCAAATCAAGGTAAAAATTCAAATCAAACACTTCTTAGCTAATTCATAAACATGACAAAGGAAGGATTTCCCCATATATACATGCCCCTCAAATTACATGTATTTGCTTATGAAAGTGCAAATACATACTTTTTGAGACCCATACACACAAACAGATAAATTTGCAGGGTTATATATTCAAAAAAATTCCCTAAAAAACAAGATGAACACAAATGACTTGACTACCAACCATAAGCCTGTAAAATACAAAGATAGCATTTTCTATTTTTATGTATAACTTATTTAAAATATTTAATTTGTTTAGTAACTGTTTTTTTCTTATATACATATGCAGCTTTTGAGTTCAGATGAATTGTGGATGTATGCACCTCTTGCTTACAATGGAATATGCATAAGCCCTGATCTCAAGATCTCTCCCCCACAACTCTAA

>Aco015075; *Ac*bHLH6
AGGAGCGAAAGAGAGCCCCCTCGACCATCTCTCCCGCCTAGGCGGCTCCGCGCGTGGCCCGAACCCTCCCCCTCCTCCTCACTCCCGCCGCCGCCGCCGCAGCGCCTCCTCCGCCGCGGCGGCGGCGGCGTTCGGATTCTCGGACGCGGGCAAGAGGGGTGGACGCGAGGGCTCGGCTCACACCTTCAGGTCCTCCTCGAGGACGACGCCGACGCCGAAGCCGCCGCCGCCGCCGCCGCCGCTGGCTCCTCGGCTCCGACCCTCCCCCTCCGCCGACTTCGACCTCCACCCCACCCCACCCCACCACCACCACCACCACCCCGGACCCCCTTCCTCACCCTTCCCTTCCCCCTCCCCCTCCTCCCCTCCTCGCACCACGACGCGTGGCCCCTGTGCGGCGCCCCCAACCCCTTCGCCTCCCCCTTCGACCTCCCCGACCCCGCCTCCGCCGCCTCCCTCTTCCCCATCCCCGACCCAAACCCTAGCCCCCAGCTCGGAAACCCCGCTCTGGCATTTCATCAAACGGGTTTCGCCCCCTTCGACCACCCCGCCGCCGCCGCCGCCGCCCCCCCGTTCCCGAACCGGCCCAAGGCCCTCAAGCCCCTCGAGATCGTCCCCCCGGTCGGAGCCCGGCCCAATTTGTTCCAGAAACGGGCCGCGGCCGCTCTAAAGCACAATTTGCCCGTGGGCCTGGGACTCGAGAAGCGGCGGAGGGGGAATGGGGATGAGGATGATGAATTCGGCGATCAGAGTATTGATGGGTCGGGTTTGAATTATGATTCGGATGAGGTTAACGTTGAGAATGTGAAGGGGGAGGGGGACGACGAGGAGGAGGACGAGGAGGACGACGAGGAGGGGGGTGTAGGGAATAGTGGAGTGGGGAGTGAGGTGAATTTGAATAGTGTAGCGAGTGGGAGTGGTGCTAGGGGTGGGAGTGGGAAAGGGAAGAACAAGAAGGGGCTTCCTGCAAAAAATTTGATGGCCGAGAGGAGGAGGAGGAAGAAGCTGAATGATCGGCTTTACATGCTAAGATCGGTCGTTCCGAAGATAAGCAAGGTGAGAATTTTACTAGTTAATTTCCAATAATTCTTCATAGGGTTGATTGCAAATTTATAGTTCGAATTTCGGGAATCCGTCCTCAGATTGTCAGTTGTAATATTTTAGTCCTAGAAGTTTTAATGCCGATTTCAATTTTATACTCGAGTTTTAATCTGGTCCTTGAACTTTGATCTTTGTTTTGGTATCGTCTTCGGAGTTTTGATGTTAACAACGGAATGCCTGATGACGGTACACACGTGGCGCCTTGTTCGGATAGTTGATTATTACAAGTACTTAATTATTATATAGAACGATCTTCGGAGTTAATTATTATATATAACAATCTTCGGAGTGAATTGTTCCAGTTGAAGTCAAGATCAAAGTGGTTCAGGGACTAAATTTGTACTGACCCTTTCTGGTACTTCGTTCTAGGATTCAGTAAATATATGTGCGGGGCGTTCGAGGCTTTACTAATTTACTTTGGAAGTGCAGTGAATTTTCTAATGGTTTTGGCCTTTTTGATTTGGAGAATATCAGTTTAGATCTATAGGCTTTATGGTTTGCGTGTGGTCCCTCTTGTCAACTTGCTTAAGAATTTATAATTCTGTCAGTTCCTGTTGCATGGCCTTATTAATATTGTAAATTGTAATATCGCATTGGTGTTACGAGTAAGCTGTTGATTAAAGTACGTGGACACATTCCTTAGGCTTTTATTTTTTATTTTTTTTGACAAACATTTTATCATAATGGAGATGTGATAGATCCTTCTTTCTTATCATTATGGAGATGTGATAGATCCTTCTTTCTTATCATTATGGAGATGTGATAGATCCTTCTTTCTTGACAGATGGATAGAGCTTCGATTCTAGGCGATGCGATCGACTACTTGAAGGAACTGTTACAAAGGATCAATGACCTTCACAATGAGCTCGAGTCAATGCCTTCAAGCTCTTCAGTGCCTGGTTCTAATACTACGACTCCTACCCCAAGCTTTCACCCTTTGACTCCGACACCACAAACTCTCCCAAGTCGTGTCAAGGAGGAACTATGCCCGAGTTCATTACCGAGCCCAACTAGTCAACCAGCCAGGGTTAGTTTTAATTGACGTATTAATTGTAAAAATTTACTATTACAAATCTAATAATTTCTTTAGAAACTGTTAATCGTGCATATTTTTTCATTTCTATTTTTTGTGCTTGTTGAAATTTAATAATGAGAAAGACTTCAGGATATTTAATATTAACAAATGGTTTCTGATGTCTAGAGCATGTTTTGGTTCAATATAGTTTGTTAGTTGGTTTTGCATGGCCCTTTGTACTCTTACTTACCACAATGCATTAACTTAGGCATTTCCTCATATCTACCTCAAGGTGGTACACACTAATCTATTCCTAAATTTTCTCTTCTTGGTGTAGTTTTTACTGAAATGAATATTATTTGGTAAGTTTAAATATTGTCTCTGTAAGCTGCAATTTGGAAATGTTCCAACTTCTATCAACGTAACTCATTGAATTACATAGTTTTACAATTTAAATTCTGGTTATGTATAATTTACTTTTGTTAATCATGTTGTTTCCGACACAGGTTGAAGTCAGGGTAAGAGAAGGGCGAGCGGTTAATATCCACATGTTCTGCTCTCGCCGACCGGGCCTATTGCTCTCCACGATGAGGGCCCTTGACAACCTTGGCCTTGACGTCCAACAGGCCGTCATCAGCTGCTTTAATGGGTTCGCAATGGATGTTTTCCGTGCTGAGGTTTGTTATATCTCTCCATCTTATATTATGCCTCTATGGCCTAGCTTTTGAAATAACTTTTCTATAACTTCTCTAAGTTAGAAAAGGAGCATAAAAGCATTTGGATAGCATTAGCTGAATGAGCTTATATAGTGCCTGTATGGTCGAGGCTCTTGACCAACTTTTCTGCTCTGAGAAGCAACCGCTAGGCTTCTTGCGAACAAAAGTACTATAGAGTTTTGCTGTGGCGAAAAACTAATATGAGCTTTTGGGTCCCAAAGTAGAAACTCAAATTTAAAGTTTCTGCCTGTGCCTGGGCAGGAAGCCGTGGTCTCGACGAGAAAGGTGCTAATGCTTTTTATGGACAGCCGAACAGATTTAGTGCAGAACTAACACACCTTTCCTGTGTTATGAGCTATATTTTCTGATGCTTCCCTTCTTTTACTCAATCAGCAATGCAGAGACGGGCCTGGGGTTTTGCCCGAAGAAATCAAAGCAGTACTCATGCACTCTGCTGGCTTTCAAGATGCAATGTAAGGTAGAGGTTGGTCGAGGAAGAATTTCGAAAGAATCAACGGTGATGATCTACCAAGGAGTTTGTTTATCCGTCGGTCTCTAATCTCACAAATTGGTTTTTCAGTGACAGTGAAAATTTCTCCTTCCTAGTGCTAGCATAGATCTTTTCTGTTAAATCATGTTCTCTGTTGGTGTCCATACAAATTGTCGCCGCTTGTCATGTAATCAATTTCCTCAGCAAGTTTTTAGTTAGTTAACAGACCTAATTTAACTTGAAATTTCTTTGTTTGGC

>Aco015882; *Ac*bHLH7
ACAGAAGAAGGAAAGAAGCCAATCTCTTCTTCTCCATGGCAACAACACAGTGGTTCTCTCAATTGGTAAGCCAAAATTAAAAATTCACACCCTCCAGAAGCTTTTTTTGTTTTTGTTTTTGTTTTTTTTTTTTGTTAGAATTAGAAAGCAGCGAAAACTTACAAACTGGATAGCGAATGTATCCGACTCTCAGAAATGAGCTGGTGCTTTATTTTAATTAACAGTATGGAAAGTACTGCAGGAGATGGATGAGGGCAGCTTCTTCCAGCAGTGGGAGGAAACAGCAGCAGCAGCAGCAGACAGCCTGCATCACTTCACAGAAGAGCAGCTCTCCATTCTGGGCTTTGGGCAGCAGGACCAGCAGCAGCAGCTGGCACAAGCCATCTGCACAGACAAGCCCAGGAAGGTGGCCCCAAAGTCCTGCAGCTGGGACTCCTGCATCACCACCACCACCACCACCACCACCACCACCACCACCACTGACCAGAGCTCATCTCCCAGCTTCCTCTCTTTCAGCAACTCAGATGTGGAGCAGCCCAGCTGCCATCTGCATGACCACCACCACCAGCAGCTCAAGAAGGAGGAGGTGGAGATCTCCTTCCCCCACCATCAGCAGGGGGGGGTCAAGAGGAAGTTTGAAGCCTTGGTTAGTGAGGGGTTGAGGAAGGTGAATACTACTACCACTGCTGCTACTACTAGGCCTGTTTCTCAGAACCAGGAGCACATCATTGCAGAGAGGAAGAGAAGGGAGAAGCTTAGCCAGAGATTCATTGCTTTATCTGCCATCATCCCTGGGCTCAAGAAGGTGTGCCAGATTTTGTAGATTCTGGGATAATTAATTTCTCTATTAAGAATTTGTTCCTAGATTGGTTCAAAAGCTAAAAGGATTACAAGTTGATATAAGAAAAAACCTTCTAATTAATTAATTATGATCCTTTATTAATTGTGTGTTTCCATCACAAATTATGCAAGATGGTTTTTTTTTTTTTTTTGAAAAAAAAATTAAAATCAAGTGGTTGGTAATTAAAGGGGAATTTAATAAATAAAGCATAGAAATTGATAGAAGAAACTTCTCAACAAATATCGAAAAACGATTTGTGAAAGAAAAAATAAATGTCTGTGGTAGAAAGCGGGGCCGTGGTTTTTTCCTGCAAAGAATTTTCCACGTAAATTCTTGTATTCTCTTTACTGTTTGTGTGCTTGTTGTAGTTATGCTACGAGGCATGAAATCATAATACATAGAAAATGTAACACAAAACACTTTTCTTTCTATTTTCAATCAATAAGTTAGGAAAGTTAGCTAGGTTTAAGAGTATATATATTCTCTAACATTGATTCATGTACAAACTGATTGCATGAGATGTTGTGATTAATGAAACCACTGGGTGCATGCATGCATGCAGATGGACAAGGCTTCTGTTCTTGGGGATGCAGTCAAATACCTGAAACAGCTGCAGGAGAAGGTGAAGACTCTGGAAGAGCAATCCAGGAAGAGAACAGTCGAAACCGCGGTCCTCGTGAAGAAGTCGCAGCTCTCTCCCGACGACGAGAGCTCCTCCTGCGACGGCGAGAGCTCCGCCATCGACGGCTCCTCCTGCGGGCCCGAGATCGAAGCCAAGATGTCGGAGAAGAGCGTGCTGGTCAAAATCCACTGCGAGAACCGGAAAGGAGTCCTCGTGAGAGCGCTCTCGGAGATCGAAAGCCACCGCCTTGCGATCGTCAGCACGAGCGTCGTTCCCTTCGCGGGTTCTTCGATCGATATTACAGTGATGGCTCAGGCAAGATATAAATCATCATCATTATTGTTACTATTATTATTTGTTGCTGTTGTTGTTCATTGTCTAATGAGTTTAAATCCTTATTTCATGTGTTTTATATATGTATGTATATATATGCAGGTTGAAGAGGGATTTTCAATGACAGTGAAGGATCTTGTGAAGAATCTGAACTCATGTTTCAAGCAGTTCATGTGAAGAAGCTAATAAATATGATCTCTTTTTTGCATTAGTACTCTTGGACATATATATATCTATATATAGACATATATATATATATAGAGAGAGAGAGAGAGAAAGAGAGAGAGGCTAATGTTTAGTGGCATTTCATAATGCTTTTAATTAGGGCTCTCCTTTTTCTTCTACCAGAGGCCTGAAGTTCCCTCAGAAGCATTGAGATCACCATTTTTCACTTGTTTTCTCTCTCTCTCTCTCTCTCTCTCTCTCTCTCTATNTTTCTATCTTTCTCTCTCTCTCTCTCTCTCTCTCTCTCTCTCTCTCTCTCTCTATCTATACACCCTTTTTGCATGGTTGAGCTAAGTCAAGTAACCAATCATGTTCTTGAGAGAGAAAACTAGCACACTTGGGGTTTTTCAAGGGCTTGATTACGTGGGTAAGTTTTAGCTTTTCAAATAATCTTTTCCTTTAATTTCTACCCCCCTCCCCTTTTTGGTTCCTATGTGGCCCTTTTTTGTTCCTTTTAGGGGATTATTATTTTTCTTTTTTTTTTTTTTTCCCCTAAACCTACTATTTCCCTTCTTCTCAATATCCATGATAACTTTTTCGCAATAGCTTGAAATTGGCTTTTTTTCTTTTTTTTTTTTAAATGGTGTACTTGTTTGTTTTGAGGGAGTGGTAGGGCCTTGTTTTAGAAGTATGGAAGGGGCGCCTCTTTTTTAGGGGCCTACAAATGATTAATTCCTCTTTTTATGAGTCTCCTTGTGAAAAATACATTAATGTTACTTAGCAAGTTTAATGTTGTTGTTGTTGTGTTAATTTTAATTTATGTAGATGCAAAAATAAATGGTTAATTTCTTTTTTCTATAGCAAATTTTATGTTATGGTGGGACTTCCTTGCTTTGGTGGCAAAATGTAGTGTATGAAAGCATTAATGTTTTGCATTTTAAATTCATTTTGCTTACCTCATTCTACAAAATTGATAATTAAAGTAGTAGTGATTGAGTGATGAGAAGGAGGAAGATCCAATTCCTTGAGAATTATTATGGGTTGGAAAATCCAATAGAAAAGTACTACATTGATTTGCTTTTGATTTGCTTGTCTAGAAGCTTAGTGAAACAATCACTTTCAATCCCTATCAATGATTATGATATTGTCTCAAACAAATCCCCCTCACTCTCTCTTATAATTAACCTTGATTGAGTTGTCCCTTTTATAATTTTGCTTCTTGTGTCATAAATTTAGTGCTAGACTAATAATAGTTTTTTTAAAATTTTATTTACTCTACATACCCTTTTTGTTTAAACTTCAAATTAATCTACTAATGCACATCTTCTCTATGAAAGGAAAAATATGCTCGAAAATATGTTTAAGTCAGTAAAATGGCATGCATTTACCAACTGTGTATTTGTGTTTGTAATGAATTGGTCATTAGTGAGCAAGGTAGTTAAAAATCAGTATCTATCCCTATATTTTTCCAATTAATTAGCGTTCTATTTTGTAAGCTTTTTTTATAAGGTTTTAGAAAAAAAAAATTTTCTATTAAGCAGCGCTATAGTTAAAAGTTGGCATTTTACTCCCCTACTTTAATTTTTTTTTTAACATAAAAATTTATTATGAAATAGTATAGTACAAATTATAAAGTCATACTTTAATACACCATAGAAATCTTGACCATTTATTTTTCTTAATAGATGGTTTAGATTTAACATGATTCTGAAGGTGACCTACTATTGAAGCATAGATACCATTCACCAGGTACCTATTTAAAGGGCATTTTGGTCTTTGTAAAATATTTTGGTTTACAAAACCAAATTTATCCAACTGAGTATACTATGACCTTTTCAAATGACTACAACAATAGTTGGATTTTAAATTACAACCGAATTGTAAATTTAAATTCAAAACAACATTTAAAACTTAATTATAAATCAAATTCAATTTTTTTAAATATAAAACACAAATTTAAAATAAAACCTTCAGTTCCAATTCAAAATTTTAATTTAAATCAAAAATTAAAATTTGGCTTTAATATTAAAAGTTCAATTTAGATTCAAATTTAAAAGTTTAATATAAATTTCAAAATTAAAATTTTACATTATAATACCAACTTCAAAAATTAAACTTAAATTTAATATTTTTTTGAGCTGGATACTTCACAAATTGGATATTATCAAACTTGGTAAGTTACCAAAGTAAATAAGATATTTAATATTTATAGGATGCTGACAAATTTGGTATGTACTACTAATTTTTTTTTCGAATATTGTCATATTAACTAATTCAAATCGGTAATCTATTCAACATGTTACTTTATGCAAAACTCAATTATATCCTTTACTAATTAAGAGTTATATTTTAATATTTATGGTGTATTGAAGCATTCCTCTAAATTATAAACCATATATAGGATAGTAAGAACAATGACATAATGAGCTCAATGTAGAGTTTGCTTATTTCTCTTAAAATTCTATATTATATAGCTTCTTTTTATAATATAAATATTCTTTAAATTGCAATTTTAGCCTTTTAATTTTTTTCACAAATCCATGTCTAGTCACTAAAAACTCTAGGACATCATTCGGTTAAATTACTATGTATTAATTTTTAATTAGAGTTTTCATGCAGTAGAATGTGCATATGAATTAAGAATTTTTCACAATCTATACAATTTATTGTGTTTATAATTTTGACAACCAACATAATTGAAACAAAAAGAAACACAAAAACAGCTAGTAACATGTTATGGTGGGGATAAATTCAAAGAAAAGAGAAATTAAGACACTCAATTTTTTCTTAATTTTTTTTTCCTCTCATCCTAACCGTCCATCAAAATTGATGGATGGTTAGAAACTTAAAAGTACATGGGCTGCTGTGCTCTTAAGAGTACAGTAGCCCTGCTCTCTCTCTCTCTCTCTCTCTCTCTATATATATATATATAATATAGAGTTTTGCTAGAATACTATTAGTAGTAAACGAGCTGTTTTACCATCTATTTGTTTTCGATGATAAAGCTTCCAAATTGACGATCGGCTCCGTTGAACATGATCTATACCACTTAAAGTGTTTAGAAATCAAATTTTAAATCTTTTCGACATCATTTGCCTAATGATCAAAGGGTTTCAAAATTTGTAATTTTAATGGTCGATAAGAGGCGTTTTCTCGTTTAACGGCGTAAAGATATCCAAATCAATTGAATTTTTGTTAGAAAATTCTTTAAACTATTTAAAACAAGATCTATACTCTTGATCTTGATTACAAGACTCTTATCATCATTTTTTAAAGAATATTCATTTTCAGCCATTTATTTTTCTGTTCACTTGATGGATAAAAGAACAATATCGAAAGTGCGTGAAATTTGATTTCTAGGTAGTTTAAATGGTTTAGATCATATTTAACGGAGCCGATCGTCAATTTGGAAGCTCTATCATCGAAAACAAATCGGTAGTAAAACGGCTCGTTTACTATCGATAGTATTCTAGCTCATCTCTCTCTCTATATATATATATAATATAAACTTTCTTGTGATCGGTGGAGAAATGTATTTGTCACGCATGAGACAAAAGCGTGAGAACTTGTTGTCCTACAACCCAACATGATTAGGACAGAGTAATTAAAATGCCTGCAATCAGAATGCGAAGGAGATGAGACCTCCTCCAAGTAACAACAACAACAATAATAATAATAATAATAACAATCAGTAAAGTGACTCGCGCGATGCGACGTATAGATAATATAAAAAAATAAAAATTTATAGAAGAAATGAAAACATGAAAATATAATTATCTTACGATTGTTGAAAATGGGGCAGCCGACGGTGGTGCCACACGCCATCCTTGTCGAGGACGTATGA

>Aco017355; *Ac*bHLH8
CTCTCTCTCTCTCTCTCTCTCTCTCTCTCTCCACTTTTACCTTTTCCATATCCACAACCCCCCCAATTTCACCTTCCTCCACAATTCTCCACTAAAAAAGCCCCTCTTTTCCCCAACTACTAGTGCTTTAATCTTGTCAATCACCCACCATCTCAGTGCTCTCTCTCTTGAGTAAGAGATGAATTGCGGGCCGCCACCGCCGCCGCCGCCGCCACCGCCACCGCCGGGCGATCTGTGCTTCTTCAACCTCAATTGGGATCAATCCATTGACCCAAATGGTGAGATCTCCTCACAGCCTCACTATGTGGCCGCTGCTCCTCTAAATTCCCCTCCAAAATTGGATCTTTCCATGATAAGTCACCTCCACCACACTCAGCAAGCTCAGGGAAGCTTGCCCATTTCGGAGGGTTTGATGCCCATCACCCATTTGGATCAATTCCCCACTGACTCTGGGTTTGTCGAACGGGCCGCGAGGTTCTCCTGCTTCGATGGCGGCGGCGGTGGTTACGCCGGATTCGGGCGGAGTTTGACGGCGAACAATGTGGAATTGGGGAACGCCCGAGAGGAATCGTCGGTCTCGGATCCGGCCTCGGCGAGCGGCGAGGTGAGCTTGAAAGGTCCCCCCTTTGAGAGCAATGCACGAAAGCGGAAAGCGGCGCCGAAGGGCAAAGGAAAAGAGACTTGTTCTACCATTTCTTCCAATGATCCTCCCAAGGTATAACAAGTAATTGAAACTAAACCTTTGCTCTTACTTACGAGTTTTGTACCGTTGCGCTGAGAGTGAAATTTTGCAATTTCAAGAACTCTGAGGCGAAGGATCCGAACGCGAAGAAATGCAAATTGGGGGAGAGCGAGGGAACCAAAGACGATTCGATCAAGCCGAAGGTTGAGCGAAATAATGCGAGCACCACCGACAATGGTAATGGTGTTCAGAAGCAAGGCAAGAACAATACGAAGCCTCCCGAGCCTCCGAAAGATTATATTCATGTTAGAGCAAGAAGAGGCCAAGCAACTGATAGCCACAGCCTCGCAGAGAGGGTAAAATTTTGAATCTCCTCTTTTATTAACTTCATTTCACATTGTTATTCTCTTTCCTTTCTCCGGCTCATACGCAAATTCTCTTGTCAATTTCCTAATTCCAGGTCAGGAGAGAGAAAATCAGCCAGCGGATGAAGTTACTACAAGACCTTGTCCCTGGATGTAACAAGGTAAACTTTTAGAGTTCACTTTGAGAAAAAAAAAAAAAAAAAATCTTGATCACGAATTATGCGTTCTGCTTCATGAATTTAGATTCTCAATGAGATTATGTCTTGTTGTAGGTTACTGGCAAAGCACTCATGCTCGATGAAATCATAAACTATGTACAGTCGCTGCAGCGACAAGTTGAGGTATAAAATTATGGTGAATAAATAGAAAAGCTTGCAATTTTAACGACGTTACATGTGTATTCACATCTAAGACCCCTGCTCCTCATTCATCTTGTTGCAGTTTCTTTCAATGAAGTTGGCCACTGTGAATCCCAACTTGGACTTCAATAATCTGCCTAATCTCCTCGCAAAAGATGTGAGCTTACTAATTTCTGTAGCCGATATCTTTCTAGTAGTCCTTAGATAGCAATGCACCTTATATTGCATTCTTTCGTGAATTCAGATGCATCAAACGTGTGGTGGTCCATTAGTGAACTCGATCTTTCCTTTAGAAACATCCGGCGTGGCATTATCATACGCCAGCCAACCTCAGCATGGAAACCTTCTCAATTGTTCTATCGCCAATGTCTTGGAGAACCATTGCTCTATCAACCCATTAGATTCCGCTCTTTGTCGAACTATGAACACACACCATCCTTACTTCAATGGAATCGGAGATGCTGCTTCTCAGGTAAAAATAGTTTTAAATTTCACAATTTATTGGTTCAATCTTATGATGTCGATGAAAGAAAGTACTTAGGAATACAAATTTCTCAGATGGGAACGTTTTGGGAGGATGATCTCCAGAGTGTAGTTCAGATGGATATCGGACAAACTCAGGAGATGGTAGTCTCTTCTCAGAGCTTCAATGGTAAGTACAGCTGAGCTATACTATTCTATTTCTATAGCGTAATTCATGGCTTCTTACTAACACACATATTGTGGCTTAACTGAAACAGGTCCATCAGAATCAGTTCACATGAAAATGGAACTGTGATAACTCGAAGTGACTCGGGTGATGATGCCTTTGTGTATATGGAGATGGCGGCTTGCAGCGGTAGAAGCTTTAAACTAACAATTTATAGCCCTAAGTGGATTTCAATCTAGTCTATAATTTTTTTCTACGCTAAATGATTTTTCGGGCGAATCTCATGTCATATGTTGTAGTTTCTCGCTAGGCTAATTATGTCGTTTGGTGCATATAGTACAAGCACCATCTCTAAGGCGTAAGGGGCGAGCAAGAGAGCGGAGCTGTGATTGGACGAATGTTTCTTTTCATCATCAGATTTCTACCTTGAAATTATGGTGTTTCTTTCGATAACTTCGTCGCAATTTCCTGCCTACTGTTTCAAGATAATTCAAAGTTCTTCGATATATCACTAATTCTAGGGAGCAATATGTTTCTCCGCTGACTTGTTGGTGTTAATCGTTAATCGTAATGGCTAATCGGTTTTTTTTTTTTTTTT

>Aco018208; *Ac*bHLH9
ATGGAGGAGCTCATCTCCACGCCCTCGTCGTGTTCGCCGCCGGCGTCGCAGCAGCACTTCTTAGGGGCGGGGCCGTCGAGTTTGCAGAGCAAGCTGCAGTTCCTGCTCCACGCGCGGCCGGAGTGGTGGGCGTACGCCATCTTCTGGCGCCGCACCCTCGGAACACCACCACCACCACGCTCACCCTCCTCACCTGGTGCTGCTCTCCTTCGGCGACGGCCACTTCCGCGGGACCCGCGACGGCGAGCACCAGAGGCGCAGAGGCGTCGATCAGGAGGGGGAGGGGGAGGGGGCGGGGGCGGGCGGGGACGGGGACGAGGCGGAGTGGTTCTACGTGGTGTCGCTGACGCGGTCGTTCGGGGTGGGGGACGCGGCGGCGCCGGCGCGGGCGTACGTGGCGTCGGCGCCGCTGTGGCTGACGGGGGCGCACGCGATGGCGGCGAGCGGGTGCGAGCGCGCGCGCGAGGCCCACCTGCACGGGATCGAGACCCTGGTGTGCGTCCCCGTCCCCGGCGGCGTCCTCGAGCTGGGCTCCGCCGACCTCATCCCCGAGAACTGGGTCCTCGTCCAGCAGGCCAACTCCATCCTCTCCTCCGACGCCCCGCACCACCACCTCGCCGCCGCTGACCCCTCGCTGCGCGCCGCCCCGCTCCTGCCCAAGAAGGAGGGCGCCGCCGCCGCGGGGCTCTCGTAGGGCGAGCGGCGCCGCGGGGCCAAGAAGCGCGGGCGGCGCCCCGGGACGGGGCGGGAGACGCCCGTGAACCACGTCGAGGCCGAGCGGCAGCGCCGNGAGAACTGGGTCCTCGTCCAGCAGGCCAACTCCATCCTCTCCTCCGACGCCCCGCACCACCACCTCGCCGCCGCTGACCCCTCGCTGCGCGCCGCCTCGCTCGACTCCGAGCACTCCGACTCCGACGCCGCGGGGCTGCTGCAGGGCGAGCGGCGCCGCGGGGCCAAGAAGCGCGGGCGGCGCCCCGGGACGGGGCGGGAGACGCCCGTGAACCACGTCGAGGCCGAGCGGCAGCGCCGGGAGAAGCTCAACCACCGCTTCTACGCCCTGCGCTCCGTCGTCCCCAACGTCTCCCGCATGGACAAGGCCTCCCTCCTCGCCGACGCCGTCGCCTACATCAAGGAGCTCCGCGCCAAGGTCGACGACCTCGAGGCCGACGCCAAGCGCGCCCGCAACGACCGCATCGTCCCCCGGCGGCGGAACCGTTTCCATGGAGTTGGAGGTGAAGGTGGTCGGATCCGACGCGCTGATCCGGGCGCAGTCGGAGAACGCCGGCCACCCCACCGCGGTGCTCATGGCGGCGCTGCGCGACCTGGAGCTGGACGTGCGGCACGCCTGCGTCGCAAACGTCAAGGAGGTGGTGCTGCAGGACGTGATCGTGACCGTGCCCTACGCGCTCCAGACAGAGGACAGCCTCCGGACGGCGCTACTCAATAGTCTAGAGAAGAGCTGCTAG

>Aco000773; *Ac*bHLH10
GCTTTCTCCATCCCTTATTTATTTATATTTATTTATTAATTAGTTCCCCTCAATTCCGTATTAAAAAAAGGGCCGGAAACCACTTCCTTGGTTTCGGTGCTTCGTGGAGAGGAAAAGAAGGGAAATAGAAAAGGAAGGTAGAGAGGAGAGGATCCGTTCGATGGCGAGTGGAGTAAGCAATTCGTGAATCTCCGATTATTTTGGTTATATTTTGGATTATTGTTTTTTTTATTTTGAGGTTTTCGATCGAATCGTGGGGGTTTGAGCTGGGGAAATCGCACGGATCGGGGATTGGATTTGGAGTACGGGGGAAGCATAGCGATCTCTCCTCGTTAATCCAGCTCCCAGGGTTTGGCCTTTTCGTGATGGCGAAGAGGCGGAAGCGTGGGGATTCAGCTGAATTGGAGCTGCTGCTTCTTCTCGACCTCTCTCTTTGATCTGCTTCGAACGGGCGCTCCATAAATCTCAGAGAGAATCCGATACGGAACCTGAATCTACCATTAAGGAAGAGCGTGAGCTCGAGATCGAGATTGAGATCGAGGGAGGGAGGGGGGAGAGAGAGAGAGAGAGAGAGAGAGAGAGAGAGAGGGCCGAAGAGATCAGTCTAGTGATGTACGGGTCGCCACAAGCGGCGAGGTCAAAGGATCTGAACCTTCCGGTATCCGCAGAGCCGGCCTTCGGACACCGGAAAGAGGAAAAGGAGCTCCTCCAGCGTCACCACCACCACCACCACCACCGGGATCATCAACAACATCAACACCATAATCCTCCTCCTCCTCCTCCTCCTCCTCATCATCAGCAGCAGCAGCAGCAGCAGCAGCAGATGAGCTCGGGATTGCTTCGCTACCGATCGGCGCCGACTGCGCTCCTGGGCGAAGTCGTCGACGACTTCCTACCCGTGCGCCCGTCGAGCCCCGAGACGGAGACGATGTTCGCCCGCTTCCTCGCCACCTCCGATCTCCGCGACGAGATCCGGGAGAAGCCGCCCTCCGCCGCCGCCCAGAGGAGCTCCACGTTCGCTTCAGCGATGGACGAAGCCGCGGCGTCGCAGCAGCAGCAGCAGCAGCAGCAGCAGCAGATGATGTACCACTCGCATCATCAGCCGATGCCGAGCCACGGATCAGTGGAGAGCCTGTATCGCACCGCGAGCTCCGCGGCGATAGACCTGGAGCCGATGAAGAGCGACGGCGCCACCAATAATCTCATCCGCCAGAGTAGCTCTCCCCCGGGCATCTTCTCCCATGTAAACGTAGATAATGGTACGATCTCCAATCTCTCACTCTCTCACTCTCTCTCTCTCTCTCTCTCTCTCTCTCTCTCTCTCTGTCGCTTCATCTACAGTTCCCATCTCATATACTGTTGCTTAGTTAATGGGCGTGCTTTAAATATCGTTACGGATGGGAAATTTCCGACTTATTAATTGGTATTCATGAGCAGAAACCGTTAGGCTTTTGATCCAATCAGGAAATTTTTGAGGGATTTCCTATTTTAGATAAGTCCACGAGGAAAAATCTCGTCTCGTGTTTGTTTAGATGCATGATGAAGGGAAGTTTCTGTTTCTGGGGACACATCCAAGGTGGAATAAATTCTCGCTGTCATGCAGGTTATTCCGTGATGAGGGGAATTGGCGGGTTCAGGAACGGGAACAACTCCCTGGGAGCGGTCAATGCCGCGAATACTAATAGTACCAGCAACAGAATGAAGGGCCAGATAAGCTTCTCGTCGAGGCAGGGGTCTCTCATGTCTCAGATCTCCGGGATGGGGAGCGAGGGCGGCGCTATTGGCGGAAGCAGCCCCGAGGACGGTGGCAACAGCGGGGGCACCGCAGCGGGCGGAGGCCGCTACATCCCGGGGTACCCTCTCGGCTCGTGGGACGACCCTCCCCTGCTCTCCGACCACTTCTCCGGCAGTTTAAAAAGAGGGAGAGAAGCTGAAGGGAAGATAATCTCCGGCCTCAACCAATCGGAGCATCAGGTACTGCTGCTGCTTTCGTATTAGATTAGATGAGATTGAACCACATGCCACATGGTATCAAAGTTAGTCCTAAGTTCCGTCGCTTCTTCCAACTTTAAACTAATCTCCTCCACAGACAAGACCGGGGATGTTATGACTACTGACATACCCCTATGATCTTTGAGTAACAGCTGTTCGGTCAAAACCCTCTCTTAAGGTAATAAACTTTTGGATGCAGAATGGTCTGATGCATCAATTTAGCCTGCCCAAGACTTCGTCGGAGATGGCTGCGATTGAAAAGTTCTTGCAGTTCCATGATGCTGTCCCGTGTAAGATCCGAGCCAAGCGAGGCTGTGCCACCCACCCACGAAGTATCGCCGAAAGGGTAATTGATTGGTTTCTTAATAGATGTTCTGTAAAATGTATTTGGGTTTGGTCGATCCTTTTAAAGCTTTGAGATTGATTAATTGCTATACTGTTGAATTTGATCTGCCAGGTAAGGAGAACTCGAATTAGCGAACGGATGCGGAAGTTGCAAGAGCTCGTCCCCAACATGGACAAGGTAGTAATTAACACTCTCCAGTAGGACAGTAGTTGCCCTCTTTTAAACCTGGCGGGTGAATTATACATGATGTCACTAAACAGAAGTTAGGCTTTAATCAATTCATCTGATTTGTTCTCTTAAGTGATCTCATCAAATCTCTTATTAGATTCTATGTGCGAGCGTTGGGTGGAAATTAAATTAGTTGTGTCCTTCTCAGTTACCTGGAACTAGTATCTGCTGTCTTAGTTAGCATGATATAGCATCTACCTGTCTTCTAAAGAAAAGTTTGCATTTAATTGGGTTGCAAAGGTGAAAAGGTAACTGCCGTATTTGGGCATGGCCCATCTTAACCAAAATCCAATTAGTCAGCGATGAGGGAGGTGCTGCTCGTAAACTAGTTTCTGGTGATTGTGATGACACGTTAGTTCTCATTGGTGAATGTACCGGCATTGAAATCGTTAGTACGCTTGAATCATAATTGTTATGTTTTAAACTTGCATTTACAGAGGTAAAGTTATTATAGAATTTGTTCATTAAATTCAACGAACCTGCTTAATTATAACAATTCCAGCTCTATCACAGTACCATCTTGTTTATCCTCTAATTTGGTGTCCGTTGTTAACAGCAAACCAACACCGCAGACATGTTAGATTTAGCCGTTGACTACATCAAAGATCTTCAGAAGCAGGTCAAGGTATACATCAATTAATCCCTATATATGAGCGCACACTAATCTCCCACAATTACAGTACCATATCTATTAAGATTATGATCTCGACTCTTTTTCTCTTTGTCGTGATCTTCTTGTTTGAAGACATTAACAGAGAACCGGGCAAGCTGTACCTGCTCTTCTAGCAAGCAAAAGCAGTACCAAAATCCAGCGGCGTGAGTTCGAATCGCCGTATATGCTACAGATAGTAGTAACATTTGATTGCTGGATGAGATCTTGGAAACAGGCCTTTTATAAGTTAGTGATGAGCTTTAACTTCACCAGTGGAGGGGGGCCCCATTGAAGCAGAGAAGAAATGTATAGTTTTGGTGGTACTTTGGGAAGTTAAACTATATACATTTATGTATACCTACCACCATGTTGTATGTATTTTTCCTTTTTTTTTGTTTCTAGTTATCAATTGAGCTCATCATCAGTCACCAACCACTCGCTGACAGCGCAACGTTTTTGGTATTTGTGTAAATGGCGTACGTGGAGGTCATTCACACCGGATGGCTTAAATGGTTGGAGGCATCCTAGTTTTCTCACCAGAAAAAATAGCTACTGGTAAATGCACTAATAGACCTCTTACGAATGACTTACAACTAACTCCATGAATACCA

>Aco000904; *Ac*bHLH11

TCTCTCTCTCTCTCTCTCTCTCTCTCTCTCTCTCTCTCTTTTCCAGTATATTAATTCTCTCCATTTTTCTCCACCTCGAATTATCTAGACTCATCCAGATCTCTTAAGCTCTAGGGTTCTTGGATATATATATATATATATATGTATTGTTTTTTTTGGTTTTCTGTTTATTGATTAGAGTTTGAAATTTCGGAGTGGATTTTAAACTCCGTGGCCAGATCGAGAGATAAAGAGTTCACTTTCGCATGCCGCGCAGTGGAGATCGCGATCTTGTTTTTATCCTGAGGTATGGATTCGAAAGATAATTCATGTTCTATTTGCTTTGATTGATTCGTAATTCAATGATGGATGGATTTTAGCGGTCTTTTGATGCGAATTATGGTCAATTTTTGGCTCAGGAATTAAACTTCTGGATTCAGTGAAGAAGCTGCGTAGTGTGTGACGATCGATGGAACTCCAAGGTAGGCCACGAAGGATTTTATATATAGCATAGCAGCCGGACTCTATATATATATATATATATATATATATATATATATATATATATATATATATATATATATATATAAATTATTTCTTTGTAGTTTCTGTTGATCTCGTTTGCTAAATTTAATTATGATATTTTGTACAATTTGTATAAAAAAAATCTCAGGAAAAAAAGCAACTCACGATTTCCTCTCCCTTTACACCAAGGATTCGGATCCAAAACCTCCCCCTCAAGGTATATATTAACTTTTTTTCAAAAAAAATTATTTTACGCTAAATTTATAAATGCATCGCCAAAATAAATACGTGGAGCTCGATTATTTTTTTTAATTTTTTATCATCATCTTAATTTAGCAATAATTTTATGCGCACTACAACTGAAACTTCAATTCCCGAACTCAAAAGAGCTGTCAAATTCAGCGAGCGAAGGTCCGCATATTTATTTGAGCAGTTCATGTACTCTCTTGTATCCAATAATAATAATAATAATAATATCCAATGTTTTGCATTATTTACATTTTTCACCTTAATTATTATTATTATTATTATTATTATTATTATTATTTAAGATCATTTGCCCCCACCCGCACCGTGATGTTTTATGCGGGGGCGGTGTTTTTAATTAATATTTTATTTAAAAAGAAAATTCGACCGCAGGGTTTTATCTAAAGACGCACGACTTCTTACAGCCGCTGGAGCGAGCGGAGGAGGCGAGGAAGAAGAAGCGCGGCGATGAGTCAGCGGCGGTGGGCCCCGCCGAGCACGTGCTCCCGGGGGGCATCGGCACGTTCAGCATCAGCCACGTGTCCGACCCCCGCGCGAGGGCGGCGGTTAAGTCCGAGCGCGGCACGTGCGCCCCCGCTCCCGGGTTCGGGCCCGAGTCCAAACCCGAGTCCGTGCCCTTCGCGCTCTGGGGCGCCTGCGCGGACCACAGAGGTAACTAAACGTCGTTCCTTCCTCCTCTAACGGACGGTCGTGATTGCTTCGCTTTTCTTTTTCTCTTCTTCTTGCGTGCGATTGTGAGTTCATCAGCACCGTTGAAGGGGATGTGGTATCTCTTTTCGGGTACGTGGACCGAGTAGGTGTCGCGCAACCGGAGCCCACGTGATGGCCACGTGACATCACGGGGCTGTTTCATTCCGCAAACGTTTGTGTTTGTGTTTGTGTATCAGCGTGATAGGTACCTCTCTACCTACGTACCTTCGTGCGGGGGACCGGGGCGGTGAGTAAATTCAGGATCGTACACTACATTCAGTGATAAAAGTTCCAGGTTACCTTTTTCGAACGTAATTTTCAGTTATCATAATAAGTGTTTTTGGTATTTTTTTTACGGTTCTAAACGAGTGGTGTCAAATTTTGTTTTCCAAACAAAAAATAAAATAAAATAAAATAAAATAAAACACCACCATGGCTTTTTGCACAGATTTTCACTCTTGCCTTGTAATAATTTTAATTAAGATACTTAAGATTAAGATTGGTCATCTGATATAGTAGTAAGAGACAATTTCATCAGAATTTCGAAGAAAAATGAGCAACTTTTGGGGCATTTATTAATGTTTTTTTGGTACCGTTGAAGTCACATGGTACGTACGAATGGTAAATAGCATGTTCTGCGTCATTATCCAAAAAGCGTGCGATCCACAGGAGCGCCTTTGGGCTCGGCCCGGAACTCAAACCGTCCCGTCAAAACTAAAATCGAAATCCGCCGCGGAGTCCGGTTGGGTCCACCGCTCTCCCGTAATTATAATAATAATAATAACAATAATAATAATAATAATAATTATTATTATTATTATTATTATTATTATTAGTATTATTATTATTATTATTAATTAATAATAAAAAAGAAAAAATAAAAAGGAAAGTGAACCTCGGCCTTGTCCCGACATTAACCTCCTCCCACACGACGACGCTCCTTGGCACTACTCCTCGTCTCGTTTATTCTTTATATAATAATAATAATAATAATAATAATAATAGTAATAGTAGTAGAATATTTTATATATATAGTTTGTTAAAGAAGAGAGAGGCGATTTAGCGCTTCTGCAGAAGCTAAAACCGTCTCCTTTTTGGCTGCTTCTTCTTCGTCCTCCCCAGCAGGGCAGTGGTCATCGCCATTCGCGGCGCACGTTAGCGGCAGCGCCAGCTTCGGATCCGTCTCCTCCGCCTCCAGGTTCGCTTCTTCTCCATCGTCCTCTCCGATTCTCGCTTCGCCCGGGGGGGTTTCTAGGTTTCTGGGAGCTCAAAGCTTGCTTTTTCGGGGCGCAGGAACAAGCCGGGGCCGGAGAGGAAGCAGTTCATGGACGCGGTCTCGAGGTCCAGCAAGGGTTTCGAGGACGACGAGGAGGAGGAGGAGGAGGAGAAGTTTGCGAGGAGAGAGGCCTCTTCTTCGCATAAAGGTTCGATCTTTATTCTTTTCTTCCTCTTTTCCTCTTACTTCTGTGCTTTTTCCCCTGGTAAATTCGAATGGGGAATGATCTTGTGCGGGATGGGATGCTTCTGCATATAGGTTAACGTACGCTGATATCAATTTGAGGCTAAAATTTCTCTGTTCTAAACAATTTGCGACGAAAAGGTGTCTTTTTTTTTTTTTAGTGCCTTTCCGGTTGCATGAACCTTTTAGCTTGTCTACTCAATTGGAAATTTCGATCATATCTAAATTCCTTCCCTTTTTTTTTTTTGTTTTTTTCCCCTTTCCTTTCTTTTTGACCGAGCTACTGGTGTACCAAAGATGTAATCTAGTCCCCTTAATTTTGTTACAGAGTTAGGTGTGAAAATAGATGGGAACGGCCGCGGGGGCGGCAATGATCAGAGACCCAACACGCCAAGATCAAAGCACTCCGCTACGGAACAGCGGAGAAGAAGCAAAATTAATGACAGGCAAGTTCAAGCTGAAGTATTTTACTATTCAAAATCCTTGAAGTCAAAGTCCTCAATTTGTTATTCTGGAAGTAGAATAGCCCTTGATGAGTGAATAAGCAACACGTTCTGAACTATAATTAAGGGTTCTTTGATAAGTAGTTTATGAGTAAGGTCGCTTGCAATACAAGACCAAGATTGAGATAATTGTCTTGATCCATATTTGAAATTCCTCTTTTTGTTTTTTTAATCTGTTTTGACCTAGAGCCTTTTTTGTTAGCTTAGAGCTAAGTACTGCTATAAAGATTACTGAGAAGTGCAAAAGAGACAAATTTAATTTATTATAATTCATCATTGGTCTTGAACTATGCCGAACCTCGTCTTCCCATCAACTTCTATCATGGGTCTATGCAATTAGCTGCCTTTTTACATTCTTTTCTGGATATTTCGAAAGTCGCATCTGAAACAAATGGGAATAATATTTTGTTTCGCTGCCATAATTCTTTGAAATCTTGCTTGCAGATTTCAGATACTTAGGGAACTCATCCCTCATAGTGATCAGAAGAGAGACAAAGCTTCATTTCTTCTAGAGGTAACTGCACCTAATTTGATTCTCTCTTTTCCCCAACTATGTTAGGTTGAGTTTTATTAGAAGTGACAGGCCAAGCTTTAAATGACCTTTTTGCAGGTTATCGAATATATCCGTTTCTTGCAAGAGAGAGTGCAAAAGTACGAGTCCTCATGCCCAGAATGGAACGAGGATAATGTGAAGATGATGCCATGGGTAATGCGATTTTTAGGGCTGTGTATTCATGAGTTGGTTCATACTTGATGTGAAATTTTATTCTTTTTTGGTGTTCTTTGATGGCTTACCTTTAGGTATCGTTTGGTTCGAGAATAAGGAAAAAGTGGCTATTCTAGGGATAGGTATAAATTGAGGTATAAGCGGGGATTAGATCAATTTTGCGTTTGGATGAAAATTGGGTTATTCCTGGGAATAAAAAAAATAGCGTTTGGTTAGGTAAGATGGAATAAGAAAGATAGTGGGTTTTATAAATAAAAAACAATCATATTATCCTCTTATTATATATAAATTTAAATTTTTAAAATTTTAAATATATATTTTTAAATTTAAAATTTTAACTTTGAAATTTCAAAATTTGAAATTTAAAATCTCAAATTTTAAATTCAAAATTTTAAACTTTAAATTTCAAACTTTAAATTTAAGATCAAATTTAAATTGAAAAATATAAATATAAAATTTTAAATTTCAAATTAAAATATCAGAATTTAATTTTAAATTTAAAATTTTAATTATAACTTTAATTTTGAGATTATAAATTATAAATTTAACAATTTAAATTTTAAATTTTAAATTTTTAAATAAGAATAGGGATTGGGAACAATTAATAAAAATAATTTATTATAATAAATTTATAAATTTTTTTAAAATTCTACTTAAACTTAAATTTAATAAAATAATTTATTATAATATTTAAATATAATTTACTATAATTTTTTATAATATTAAATAAAATAATAATTATTAATATAATACAATTAATAATTTTATAATAAAAATAATGTATTATAATATATAATTTTAAAAATTAAATTTTTAATTTTTAAATTTTTAAAATAAGAATAGGGGTGGGAACAATTAATAAAACTATTTATGATAATAAATATTAAATTTATTAATTCTACTTAAACTTAAATTTAATAAAAAAATTTATTATAATATTTAATATATTATTATAAATTTATTTATATATTTAAATATAAATAATATGTATTAATATAGTACAATTAATAATTTTATAATAAAAATATTGTATTATAATATATAACATATTATATTTAACTTAATTTTGATTTAATATATTTATATTATACAATATATACATTGGAATAGTGCTATTCCACCAAAATGGTGGAATAGCAAATCCCTTGCTATTCCGGGATAACCGGGAATAGCAAGGTTTGACCCGGATAAAATATCCCGGTCAAATTTCATCCTGTTTGGCGGAATAGCTGGGATAACTTTCGTTATCCCTGCTTATTCCTCCAACCAAACGGGGCCTTAGTGTCACCAAGGGCCTCAATTGGTCTGAGGGGGATGGCATACCTGCTTGTGTGTGGTTGCACAAATTTTTAGGGCTATATGATCAGTAGTTTGGATGCGTACATCACACATCCCATAAAAATGTTACTTTGGATATATTTGTTTGGATGCATCCCTAATTCATTCCATGTGTATTGTTGGAACATACTTGTTTGGGTCGGAGGAGTATTATTTTGTGATGTATATATCTACATCCCACTTCTCATTTTCTGGCAAAATTTAAGTAAGCCCTTGAATTCGGAAGCTTAAAAAATATTTAGATGCAAACAGATAGTACAGTAGGTCTTGAAATCTTTCTTCTATGCACTAATAAATTTCAGCTTATGCTGTTGCTCTCTGAGATTAATTAGTTTCAACCACCATACCTCCATGCATATTTACGGCAGATTTGGGTTTATTTTTACTGTTGGAACTATTTTATAAGAATACAAGTATCAACTAGTCCATGTTGCACCAAGTGAAATTTTTAGATTGTACAGAGGTCTTTAGTTTTTCTTAGTGACACTTACAAACAAAAAAAGAGCCTCCTTTTTTTTTATTGATTTTCATTTTGGTTTCTTTGGCAGGTAAAAGTCTATTTTAGATCATTCTGGAAAAATTCACGGGTACCTGTCACTTAAAAAACTGAAAATAGAGCTAGAATTTGTAGGGCACGTTTATCTTTTAAGAGTTTCTTATATATTTCATCATTCTTTTAATCAGCTGTCTGAACTTTCTCTTTTTTTCTCTTCTTTTCCTTTTTTTTTGCAGAATAATAACCGAAGCCCTGAAAATTTTATATCTGATTCTTCACAAATCAACAAAAGTGGTTCTGCTCACCCTCAGCAAACTTTGCTTGGAAAGATCAATGACAATCACATTCAGATTGCACATACGGTTACCTCAGATGCACCCAATCCAACAGAACTGAACCAAATGGCTGGTGTCTCTTTCAAAGCGACTGAAAACCCCCCACATTGTCCTGGTAAGGCAGCTGATTTACACGCTTCTTTTGCTCATATACTATTGCTCTGTTGGATGGTTCAGTGATGTTAGTCTTAAAGGAGGTTTCTTTTAGATGCAGATAACAACACATTTCAGGCGCAAAACCAATGGTTAAGATCACCTCTTGTTGCCGATTCTGCATTTAGCAATGAAATGTTAAATCAACATGAGGAACTGGCGATAGATGACGGCACAATTAATGTCTCTTGTCATTACTCTCAAGAGTATGTATTTGATGACTCTCTTGCTTTTATTCTTTTAGTTGGTTATTTCTTTTTATTTTGTTAAGCTCACTTATCTTAATATCAATTGTTGATGCATATTCTTGTTACTTTGCTATTGAATGTGGGGGATTCTTGTGTATTATGTCTGAGAATAGTGGTTTCTTTTTCCCATTTTTTATTACTTTCCGGTCTCTGAACGAGCTTCCATCTTTGCTTATTCTCTGTTATACTATGGGATTAAATATCCTACTTGCTAGATTTCTGCTTGATAAATCTTTTATATATTGCATTATTTGAAAAAAAGATTTTTTTTTTTCTCAATCTTTCTTTCAACTATAGTCAATGATGCATGAACTTGTGTGTTTTGATATCCTCATGAATGTTCAGCAATTGTCATTAATTGTTCGTTTTTTCTCTTTTCTTTTCTTTTTCTTTTTTCTTTTGCATTCTTTTGACTGTTTACTTCACATTTCTGCATTTAGTGATCAAATATTTAATATTACGCCGATTAAGTGTATTGGTAAACTATTTAGTTTCATTTTGTGTGAAGAATTGAAGCTAATGCTGAACTGAACTACTTGATCTAACAGGTTGTTGACTACACTGAGTCAGGCTCTCCAAAACTCTGGTTTAGATTTGTCACAAGCCAGCATCTCTGTACAGATCAATCTGGGTAAACGTGCCGCTAATAAGAGAAGTGCAGCAGTCTCAACTTCCAGTCCGAAGGTAATAAGCTATTGGTGTATTTCTCCATTTGTTTGTATGCCTTCTTAAACAGTTAAACACAAAGAATAGATGTTCAAATCTTCTTTCAAAGTTCCGGTTTGGTTCGCTTTTATACCATTTCTAGTGTAGAATACTGCTGTCAGATAGGTTTAGACATTTAATATTGCCCTTCACCTGCTTTAGTCTTGCTCCTAGTTCTAGAGGGTGTGAATATTATTTTGTCCAACATTTTGAGAAACGCTAAGGTATGTTTGGTTGTCGGATATGTTTGTTGGTATTATTACATACTCTTTGTTTAGGTTCATACCTTGTTATTGTTGTGATGTAAGACATAAGGTGATTTCTTGTGCAATTCATTTTAAGGTGGTGGTGCCCTTGAAAGATTTTTTAGGCCATTAGACAGGATATAAAGATGGCACCATATTGAAGTCGCCAGATATCAAAAGCACAAAGCTATTATATGGCTGAGCATGATTTATCTAATTTTCAAAAATCTTGGATGTATACGATAGTTCGTAGATATACTTTTTAGTTCTCCGATTTCTGATATATGGCTGAGCATATGCTTTTTCTCTTATCTGCTGTAGATCAGATTGTAAAATTAAGAAAAATTCATTCCCTTCAGCAAAAGTTTTTAAGTTTTGTTTTTCAAAAATCTCATATCATTTTGAACCATTTTGCAAAATCAATCTGATCCCTTTCGCTCTACATCTTTTCCCTGTTTATAGGATCACGTTCCTGCCGTAAATAATCAAGCAAGTGAGCATGCAAGGGCGGGCAACACCGGACAAGAGTTTTCTGAAGCACCAAAGAGGCGCAGAGTCGATAACAGCTAACGCCAATTTCCTCAAACAGTTTTTTGGGATTTTTATTTTTATTTTTTAACATATTTTCCGGCTGTTGCTGAGCACCGCTGATCAGTGTATATTTGGGGTCCGCGATTCTGATTTTCTCTTGTTGCTGCGCACCGTCGACTAGTGTATATTATTCTATATCTATATCTATATATATATATTTGATCTTGATAGCCCCGACGCATGCTTGTGTTGGATATTCTTTCAATCTTCTGTAATTTTTTTTATCTTTTTTTCCTATTTCTTTTTCTTCTTGGAAGAGGCTTCCGCTGTGGATACGTGAAGCTTTTTATTTCATGCTTCGCATTGTTTAGTGTTGCAGATTAATTTGTATATATACTTATTCACAATGCTTGGTGTATTTGCGTAGTCATTCTTATGGCAGGTAG

>Aco000913; *Ac*bHLH12
TCTCTCTCTATCTCTATCTAAAAACAGAAGGGGACCTCAGTATATCTCCTGCCTTGCTCTCATTGGAGGTTGGGACATGGCAAGGAAAGGCTTCATGGAGGGCAGAGTGTTAAAAGTAGAGGGAATCTGAAGTGGGTATCTTTTCTTTTCCCCACTTTTTTCCTCAGTGTGGCTGTTTCCCTTTGCACTCCATCAACCTCTCAAACTCTCTTAAACACCAACTTCTAAGAGGCAAAAAAACAGGGAGCTGTGAAAGACTGGGTTCTGCTCTAATGATCTCTGAGGCCAATGTGCTGTTTAATTATTATTATCTTCTATCAATTTGGATTTGTCAGCTGTAGTTAGTTTCCATCTGGGGGTAAGAAAAAGATTTTTTTTCTTCTTCTTCTTCTTTTTGCTGCTTTTTGGGATTTTTTTTTTTTTTTTTTTTTTTCTGAGTCTAGACATTGGCTTCTTGTGCCAGCTGGGTTGTGCCTATTAAGGATTCTCTCTTTGGTTGATCAGGCAGTTTTGGTGAAAGAGCAGCTTCTGCAGTCTATCCTGTTTCTTCTATTTATATAGCTTTTCTCATTCTGCCTTCTTTTTTTTCTTTTTTTTAAAGAGAGATTAGGTGGTAAATTTATTCTGAAAAACTAGTGATCTCTGGAATGGGCAAAAGCCTTGTTATTTAGCTAACTTATTGCCTGGTGGTGTTGCTGGGAAGTGACCTGTTGATTCCATCTGTTTCCTCTTAATGGAATACTAAATATTCCCCACACCAAACACTCTCATCTTTTGGTTAAACCCTTTTCACCCCTCATTTGATCTCTCTCAATCTAAGATAACCCCCACCCCCCCCCCACCTTTACTGATCTCTGAGTTTGTGGGGTGTTGTTGAGGGTGCTGAGATTAGGGTTAGGGTTGTGTCAGCAAAGAGGATGGATATGAATGAGAAGGAGAAGCTGGGGTTGGAGAAAAATGAGGATCATATGAGCTACCATGCTTCTGACATCTCAACAGCTTGGCAGCAATTCAACAGTTCATCAGGAGCAATTGGTCAAGAGATTCAGATGGGCTCTGCTACATGCCCATCAAATCCCATGGTGGATTCCTTCAATCCAAGCCTCTGGAACCACCACCCATCCTCGCAAAATTTGGTGGGCTTGTCCGAAAACAATGCTCATGTAGGAAACCCCCCCATGGGCTTGAACCCTTCGAGTTTTTTCCCGAAAGGAGGGACTTTCTTGCCGTCGCTTCCGAAGATCCCTCCTCCCCCAATCCTTCCTCATTTCTCGGCCGACACCGGTTTCATCGAGCGCGCCGCGAGGTTCTCGTGCTTCGGCAGTGGCAATCTGAGCGGCATGGTGAATCCTTTCGGTGCTTCTGAAATTTTGAGCCCTTACGCCAATCCGTCGAAATTTTCGGGTCAAAAGAGCGAAATGAGTGTCGACGAAGCTTCCGAGAATGCCCCCTTGCCTGTGAATGATCATTTATCAAGCATTAGGAGCCCGAGGGAACGGAGAGATGATGCGAACAGCTTGCGCGAATCGGCCGAGCCTGAATTTAGTGGAGGTGGTGAAGAGGAGGCACCTAATTTGGCCACTGCAGCTCGAGATAATAGCTCTTCGAAAGGGCCTAATGCTAAGAAGAGGAAAAGGTCCAATCAGGTAAACCATAATTACATTTTCTTCAACTATATTCAGAGTTGTGTGTGTTGCTTTTTGCTTGTTCTTAAGCTATACATGTCACAAACATGGAGATCAAGAAATATTTATTGTTCACTAAAGGGCGGTGCGGAAACGGAACAAGTTGAAGGAGCCCCACAAGTGCCAATTGAAACCACAAAGGAGAATATGGACAATAAACAAAAGAATGAACAGAATAGCTCAACTGTGGGGACTAGCAAGGCTTCTGGGAAACAAGCCAAGGATAATTCTGATTTGCCCAAAGAAGATTACATTCATGTAAGAGCTCGCCGCGGCCAAGCAACTAATAGCCACAGCCTTGCAGAAAGGGTAGGGTTCCTTTTCAATTTGTTTCAGCATTTGGAGCTCTAGGGTTTTGCGTCTCTAGTTATTTGCAGTAAGAGATTTGGTTTTGATGGTTTTGATGCTTTCTTATGCAGTTGAGGAGGGAAAAAATTAGTGAAAGGATGAAGTTTCTGCAAGACCTTGTTCCTGGTTGCAGTAAAGTTAGTCTTTCTACATAATTACCTCTAGGTTTCTGATCCGTAAATTCTAATCAATATACATTCACTCCCTTTCTGAACTTGTGAGTTCAGGTTACCGGAAAGGCAGTTATGCTGGACGAAATAATCAACTATGTTCAATCTCTACAACGACAGGTCGAGGTAAGAAAACTCTTGTAAAGCAACTAATTCCAGATAGAGTCTCTGATTATTTATATGGACAATTCTCTAAGAGGAAATTTCTGTTTCTATTTCCTTTTTTCTGTTTCCAGTTTTTGTCCATGAAGCTTGCTGCTGTTAATCCAAGACTAGACTTCAACATAGAAGGACTACTTTCTAAGGAAGTACGTATCGCTGAGGGCTTTTATGCTTGTAATTTGGAAATTCTCAAGCTTCACCTAATCTTTTTTCTTATTTCTAGTTACGTCGATAATCATGATGACAAACGGATCGTATTTTACTTAGTTGGGTAATTTTCTCTTTCCTTTTTTCTTTTAACAGCTTTTCTCCCGCGGTGGTCCTTCATCGGCAATTGGCTTCTCACCAGATATGATCCATCCTCATCTACATCCATCTCAACATGGCATGGTTCAAGCTGGGATGCCAAGCATTGCAAACCCTTCAGATGTGCTTAGAAGAGTTATGAATGCTCAACCAACTTCCATCAATGGATATAAAGAACCTACATCTCAGGTTTTACAGCTCTCTATCTCTGTCTTTTCAGCACAAGTATATCATAATCTACTCAAAAAAAAAAAAATCATAATCTTGCGCATGTTATTATGAGCAACAATCAAGATATGTTGATTATATCTTAGGATGTGTTAATGTATCATTCGAGGTGCTATTATGAGATGCACTTTGACAGAATCCTGGTACTAAAGTTGGCAATTTTACTATTTAGATCTTTCCAAGCTAAAAATACTTAATCATTCAATCCAAAATAGTGGTATTGTAATAAAACTACTACCAAAAAATTTGTTCAGAAAATGTTCTTATTTTTCTGTTTCGAGAATTGATGCTCCACAAATAACCACATTATGTTTCTTTCAAATTCATCAAGTATTCACATTTATAATTAGCATTAACAAATTACAGCCTCTGGTTTTGAAATCATTGAACAGATGCCTAATACATGGGATGAAGAGCTCCAAAACGTCATGCAAATGAGTTATCCACCTCTCAACGCTCAAGAGTTAAACAGTAAACCTCGAGATGGATTTCCCTTATGAAGTTGCATCCATGTTCATGTTCCTGAAGATCATACATTTATATATATGGCAGTAAAGCGCACCCACCTGTTCATATGGAACTCAAGCTGTGACCGCTACATGGATGCATGGTAAGCTTATCGCAGTATGCATGTGTTTTTCTTCAAAGAAGTCAGTGAAGCTTATACATAGTCAAAGATGCAGGGTTCATAATGGAAGAGTCACTGTTTTCTCTATCCGCATTTATATCGAAAGGTTGGTTGTTGTTGCTTTCTTGTATAGCAACTTAAGGACTGCTTCCTCCTAAGACCAAGTTGCATTCTTTAATTGCTTTATTTCTTTGATTAGGCACTTTAATGCACCAAAAAAAAAAAGGGTGAGAATTAAGGGAGAATGAATTCTCTCTTTCTTCCCCTCTCTTGAATTACAGTTGTAGAGTCACTTGCATGCCTTTTTTTTATTCAGTACTATAAAGGGATTTCTCATAAAATGGATGTATAACCTGGACTGCTTTGGTTTTTAGTATGAAAATGTTCTTCGGGATAAAATCTTTTTGTATGTCTGTTGATATTAAAAGCAGATATCATATATCATTTTTTGTATTCTTTTAATGAAAAAGGGCCTCCACCCTTATATTTGATGTAGTATTTCCTCTTCTCATGACATCTGAAAATTGCTTTTGTTTTACCTTCTTCATTTTCCTTCTTCGGTGACAACACAGAGACTTATGGGGGAAGCAGTTGCTGTCTTGAATGCTTCTGACTAATCACATCTTATATATGATTAGCAGTATATAACAGGATGCCATTATAGATGATTCAATCCGCTTAACAAATCAGTATCTTAGGAATTGAGTTCTCCAAGCACATGTATGAAGTTGGATTGAGTTAACTGAGGAGTTCCTTTTCGCCCACTTCTTACTGATTGGATCTGAAAGGAATCGGTGATTGCTAGCAGACCAAAGTAGGAATATAGCAGAATGGAGATTCTGTAAGCTATGGCATGAATAAGAACTTTTTTTAAAATGCAAATATTGCTCAAAGAAATGCTTCTCGTAATTGTCAAATAGAAACATGGCTTGTGCTGTTTTAGTAGAAATCAAAGCGAAAAGGATAAC

>Aco001074; *Ac*bHLH13
GAGGGAGGGAGAGAGGGAGAAAGAAGCACAAACTTTGCTATGTGGGAAGATAGAGTTCACGGTCTCCACTACACCGCCACGAACACTTCTTCTACTAGTACTACTACTGGTGCGACGATACTCGGAGGCGACACCGCAACCGGTCTCTTACACAACCCCACCACTATTCTCCCATGGGCGGTAAACCCTAGCGCCTACTCTCTTCCTTATCATGCTCCCTTCAACGGCGACTTTTACCAGCCCGAGAGGAATTTTCCGGCGTTGGGCGCGTCGACGGATCAAGCAGATCTGTCGGGCCTGCACATCGGCTCGTCGACGGAGAGACTCCTGCACGGGAAAGCAGGGGGGTTGGCGTCGACGATGTCGATCTTCGGGAGCCTCCACGCGGAGTTCGGCAAGATGAGCGCCAAGGAGATCATGGACGCCAAGGCGCTCGCCGCCTCCAAGAGTCACAGCGAGGCCGAGCGCAGGCGCCGCCAGCGCATCAACGGCCATCTCGCCAAGCTGCGCAGCTTGCTTCCTAACACCACCAAGGTGAGATCAGTAACAATAATTAATTCCTCCCATTAATCCACTATAATTATTATTATTATTATCATTATTTCCGTCATCTGTAAATTATCTGAAGTCGATATATGCATATATATTCGGGGATCTCACATGGATTTTACATATATTCTAAATTAAAGATACGAACGAACGAGTGGTGCAATCACTCTTTGGGCGTGTTTGTTTGTTTTTTCCTGATCTTCCAGCAGGAAACTCGCTCTCTTGTCGGAGATTGAACATGAGGTGTGTTTGGAGGCGTATGCTATGTTTCCTACCAGCACCAAGTCAAAGATCACACATACAGGCGCTTAATTCTATCCTTCCACAAACTGATGATGATTTTTCTTGGGCTGCGAGAATTTAGAGAGAGAGAGAGAGAGAGAGAGGAGAAAAGAGAGGACAAAGAGGTGGAAAAGAGCCGCCGAGTTGAATGTTGTTTGACGCTCAAAAGGGAAACCGGAGGAGCAGAATTTGAACAGATCAGCTTTTCGTGGGAGCTGCTTCTGCCGGAAAACTAAAAAAAAGCTCCTAAGAAAGACAAACACGCGCACCCCCTTCTTGTCAGAACCCATTATTAAAGCTGAACCGGAAAAAAAAACAAAAAAAACAAAAAAAAAAAAAACGGGCACCACCACTAGTCGCACGGTTCTGCAGCTTTTTTTTCCCCACGCCCATAATCCCCCGTCCATATCCACACTCCCTAACCGAACCTATTATTCCCATTCAATTGTTCGTTATATATTTCAGAAACAAGTCTATTATGTAGATCTAATGGATAAGAATTATAAGCTCTTATGAATCGCTGTTGATTTGATATTAACAACAGCAGTCATTTTATTATTAAAAAAAATAATACCAGTTGTATTAGTATGTCAAGAATACATATCTATGATTCGAATGATGCATATTGAATGGTGAATCTGTTCAATTTGAATATATATATATAAAAGTTTTTTTTTTTCTTTTTTATTTCGTTAATGCGGAACCAATACAATTCAAATTGAGTTATAATTGTTTTGGTTCCTCTCTATATATAATGTGACAGACGGATAAGGCGTCGCTGCTGGCGGAGGTGATAGAGCACGTGAAGGAGCTGAAGCGGCAGACGACGGCGATCACGGCGGAGGGCGGCACGCTGCTGCTGCCGACGGAGGCGGACGAGCTGACGGTCGACGCGGCAGGCAGCGACGAGGACGGGCGGCTGGTGGTGCGGGCGTCGCTGTGCTGCGACGACCGCTCCGACCTCATCCCCGACCTCGCGCGGGCCCTCAAGTCGCTCAAGCTCCGCGCCCGCCGCGCCGAGATCGCCACCCTCGGCGGCCGCGTCAAGAACGTCTTCATCATCACGGCCCACGACGACGCCTGCCATAGCGACTGCGTCGCCTCCATCCAAGAAACGCTTCGCGCCGTCATGGACAAAAAAACTGCCACCAGCGACACGTCATCGTCCAGCAGCGGAATCAAGCGGCAGCGGACGAACCGCGTAGATGAGCAACAAGGTTCTATCTAATTAACTAATTTCTATATATTCTCTTACCATCTTGGGTACGTGCTTAATTAGATTTTTATCTGCATGGTGTTGGAGTGTTGTGGTGTATGTGTGTGCGTGGGCGGTGCATATATGTGTGCACTTTTAACAAGAGGGAGAATTTTATGTATCCCTTTGTTGTTGGGTGGTTTGCATGGGGATGAGCTAGGAAGGGCTTTGAGTGGTGTTTCTAGGGACAAGGTTTGGGTTGTGAAAAATAAGCGGATCTGATTATATCAAT

>Aco001133; *Ac*bHLH14
CCCAACCCAACCCAACCCAACTTTTCTTCTATATAAGGTTCCCTTTTCTCCACCTTTTCCTATCCCTTTCCCCCTCCTTACCTTACTTTTTCTTTGGGTACCCATCTTAAAGACAAACAAACATGTCCAGCAGGTCAAGGATCACTGAGGAGGAGATCAATGAGCTCATCTCCAAGCTACAGTCTCTTCTTCCTGACCAGGCTCGCCGCCGCGGCTCCAGCAGGGTAATTAAGCAACCTATTTGTTCCCACAAACCAATCGCATTTTCGCTGCGAGTGGTGTTACCCGCATATCAGAAGATTCATCAAATCGGATCACTGTTCATAGATTGATTTTGACCAGATTCAGTGAGCCTGATTTGCATATACATGCTGCATTATCATAATTGCCATCTTTTTGATTTTCGGTTTGGTTCTTGGTTTTGCAAACGTTTGGTTTAGGCTTCGGCGACGAAGGTGTTGAAGGAGACGTGCAACTACATCAAGAGCCTCCACCGCGAGGTCGACGACCTGAGCGACCGCCTCTCCGACCTGATGGCCAGCATGGACAGCGATAGCCCGCAGGCGGAGATCCTCCGCAACCTCCTTCGTTCTTAAGCTCAAGTTACGCCTCCTTGATTCCTCGCGCTTAGGGTGTCTTAGCTAGATCTCAATGTACTTATTTTATAAGCTTATTAATTAGGGCTTAATTAGGAACTCAGCTTATGGCTTATAGCTACAGCTACCTCTTAGTTTTCTTCTTCTTCTTCTTCTTCTTCTTCTTCTTTTTGGGTTTACTTCGTATATCGGGTGTGGTTCGAGTTGTTTCTCCCAAGACAACCGACCAACCCTATGGTTTTATAATATTATGTAATCCAGTTTCTACTTAGTTCCTGCATTATGTTAGACATATAATATAAGCTTAATTGGTCTACTTCTTGGTTATGGTTCCGGCTCCTGCTCCGGCTCTGGCTCTATCCATTCGCATGTGAAAGGGGCTGTTACTTAAATATAAATAAATTCCAATATTTTTTGCATTATTACATGCTTAAGTGTTACATTATTTCCTTTTTGGATCTTATGTATGACTCCATGTTCCAACTG

>Aco001136 *Ac*bHLH15
ATGTCAAGCAGGAGACCGCGATCACCGCGTTCAAGTACTTCAAGAATCACAGAAGACCAGATCAATAATCTGCTCTCCACGCTGCAACATTTGCTCCCCACGTCTCTTGTGGGAAACACGAATAGAGTAAGTCCAAGTTAATCATCCCACGTGTAATAGATTACGAGTATTTCACTAACACAACAGTGCATATTTTGACGACCTAATTAATTGAAATACTATGGTTAATATCCATATATATATATACACATAACTTACATCGGTACGTGTAGGTGTCAGCGGCTAGGTTACTACATGACATATGCAACTACATCAGAAGCTTGCATGAGGAGGCAGATCATTTAAGCGAGAGGCTCGCAGAGTTACTCGCGGCGACCGACGCAAGCAGTGCTCAAGCAGATATTGTTAGGAGTCTTCCAAATTAATTATTCCCCCCACCCTCCCTTTATGTATCTTCCTTATTTACTCCTAGTTGGTTCGCATTATGTTTGCTACATGTGTTGCTAAAAATGGCGTAATGAGGTAAAGTGTCATGTTAGCGTATCTGGATATGTATATGGTAGTGAAGGAGATGGTAGTGAATGAGAGTGTTAATTTGTCAATGAGCTAGCTAACTAATTAATGCTTGAAAGCTTCGTGGTTTGTTAGCTATTATTCTTGAGCGCAGATTAATGATTGCACGTGTTGAGTACGTGAGCCAAGAAAGACTCTCAGCGTGTTAGCTCAAAATGTACTTTTCCCAGTGAAGTAGATTAGTTATCTTTCAGTTGTAAATAACAATAGGACAATTTACGTAAAAAAGAATATTTACAAAAATTAAATACTGTCCCAGTTTTTACATATTTGATTTAAATTTTTAGTAAAATTTTCTAAATCTTTTTTTTTTTGAAGGTTTATTTTTTTCAGTTGAAACCCTCCTCAACTTCTAATCTTTGA

>Aco001255; *Ac*bHLH16
CCCCCCCCCCCCCCNCCCCCCCTTCTGAAGTGGGTGAGTATATATGGCTTTAGAAGCGGTGGTGTTCCCACATGGGCTCTTGGGTTACACCATGAAGGAGCTTTGCTCCATGGGGGGAGGAGGAGGAGGAGGAGGAGGATGTAGCCATGGCGAACTTGGTTGGTTTGAAGAGGAGGAGGAGAAAGGGGTGCTTTTGGGTTACTTAGAGGAGGGTGGTGGTGGTGATGAACAAAGTGGGAAAAGAAAGTGGGATGTGAATTGGGTGGACACTTCTTGCTCCTCAATGGTGGGGGTTTTTGAGGAGTGGGATAACGTGAATTCGCTGTCGCCGCCACCGGAGCCGGCTGGGGGGCGGCGGAAGAGGCGGCGCATGAAGAGTATCAAGAACAAGGAGGAGGTGGAGAACCAGCGTATGACCCACATTGCCGTCGAGCGCAACCGCCGCAAGCAGATGAACGAGTACCTCGCCGTCCTCCGCTCCATCATGCCCCCTTCCTACGTTCAAAGGGTACAATTTCTTCACCTATCAGCTTAAGCTTTTAGAATATTGGTCCTACATACGTACGTAGGAGCGTTCCTTTTCTCTTCATCTAATTGCTCTTTTTAGTCCCTTAAATACTTGAATAAGTTATTCAGTAATAACATATATTAAAGCAGTTTCTTCTTGAGTTAGTTAATTTTAATATATTTCACTCTTTTCATAAGGGAGATGCGCCAATCATGCCCTAAAAAGTTTCTAATGAGAGTCAAGGTGACAGCTCCTTTGGTTCGTAATTTTGGCAGAGAATCTAAAATACAAACTGTTGCAGTGCCTGAAAAGCTGAACCATTTTGTCCCACGTGTTTGTATATATGCTTCGTGCCTAAACTTATTGTCGGAACTAATCAAATCTCTTTTTCATCATCTAATTATTTTTAATTACTTCGTGTGATTAAACTATAACAGGGTGATCAAGCATCTATCATAGGGGGCGCCATAAATTTCGTGAAGGAGCTCGAACAGCTCCTCCAATCCCTCGAGGCCCAGAAGAGAAGTAACGAGCAACATTCCGAATCCGCGCCGTTCGCCGAGTTCTTCACGTTTCCCCAGTACTCGTCGTACACCGCGCGAAGCCCGAATGCCACTGCCGCGATGGTCGCGAACGAATCGGCGGCCGCGGCGGAGGAGGCGAAGGGGTCGGCGGCGGCGGCGGATATCGAAGTGACGATGGTGGAGAGCCACGCGAATTTGAAGGTGCTCGCGAAGCGGCGGCCGAAGCAGCTGCTGAAGCTGGTGGTGGGGCTGCAGAATCTGCGGCTCACCACGCTGCACCTCAACGTGACCACACTTGATCGAATGGTCCTCTACTCGTTTAGTCTCAAGGTAGTAGCAGTAGTAGTAATACTAATGAAGCTCACTTTCGTTAATCTCTTAGTTAAAAAGTAATTTCTATTTGATTGGTAATAATCGGGTAATTAATAATAGTGGTAATACAATCACTTGAGTGTGGTGTTCATTCACGTGCACTTGCACTGAAAGAAAAGATAATAATACAGATAAATGACGTGAATTTATAGCTCTTTTGTGGGCGATCAAAATTGTGAGCTGAAGAGTCCGTTTGATGAAGCACCAAACTCATAAGATCCTCCGATCCCGTACTCACATCCTTATAGATCTCAGGCTCTCAATTTTGGCCAGGTGACCGATCGTTTCTAGCACAGTTAGCTAAGAATTTGATGGCTGGTACTCGAGGTATTAAATTCAAAATATAATTATCTCACATTTTTAATTAAGTTTATAATTTTTAAGAAATAAACAAAGTAAATAATATGCTATTTTTTCCTCAGAAAAAAAAAAAAAAGGCCATCTAGATGCTTTCAAACAACCTCTTAAAACCATCAAATTTGGCCATCTAGATACTTAATTTAGTGAAATGGTTTAGGTGCTAATTTTGATACCATACTATCAAAAAGATCAATCAGATATTATATAGATCATAATTTATGAATAATATTCGCAAATTTTAAGTGCTGTTTATTAGTCATTATTTGCCATAAATGGCCATATATGTTAGTAAGGTGCTCTCTCATTGATATTTTGTGTCGACTTTTTTTGGGGTGGTGTAGGTGGAAGATGATTGCCAACTCTCCTCAGTCGACGACATCGCGGCCGCAGTTCACCACATTGTCGGGAAAGTTGAAGAGGAGGCGGCCATGTTGTAATTACCAAACTTATTAACTTGCTTGTAATCCATCCTAACCATAAATGCGTCGCAATCTCTCTTGGCTTTTAGTCAGCAAATGTTTGTTAATTTGTGCTTTACTTTTGCTGAGAATATGCATGGTTTGGATGCAAGTAATGTCTAAGCCTACGTGTATAACGAGTTTTTGTTCCTTTTGTTAATTAGTCTCTCTCTAAGATAATAATGAATGGGCATTCCAAAAGGGTAAAATTTCGATAAATTTATCATTGATTGGGAAAAGATTCTGGGAAGAGGGTCTTTTTCA

>Aco001282; *Ac*bHLH17
CGAAGGGGGAAATCTCGGGGAGCGGTAATAATAAACCTCTGGCCCCCATCTCTCCCCCCTCTCCCTATCTCTCTATCTCCCTCTCTCCTCCTCAAATCCTCTCTCTCCCCCTCTATCCCCAAACCCTAGACGAAGCCACATCCCTCTCTCTCTTTCTCTCTTTCTTCCCCTTTCCTCTGCGGCGCGATCTAGGGTTTGGATCGAGAACGGGGAGAGGAAGGGGGGTGGCGGGGCGCGGCAATGGCGGAGCCGAAGAACCGGTGGACGTGGGAGGTGCCGGGATTCGAGCCGAGGAGGTCGTACGAGCCGCAGGAATCATCGGTGGCGCACACGGTGGTGCGGCGCCTCTCCGTCTCGCCGTCGACGCTGCTCCCGCGCGCCGAGGCCCCGCCGCGGCCCTCCGTCGCGGCGAAGCTCCAGAGGTTGAAGGAGCAAGTGAAGGTTCGTTTGCTCGCTCGCTCCCCTCGCTTTAAAGATTAGTTTCTTGATGTGTTTTCGGGAATGTGGAGATCATTCGGGGGGAATTGCGTTCAAGAATTGGTTGAGAAAACCCGGAAAAAGTTTTCGGGTTTTGTTGAGCTGTTCCGTGTGTTGATCGTGTTAACGCTAGACGGCATGTCTGATTCGAGAACAACTTAATGCAAATATAGTTGATTATGCGGATAGCATTGTTGTGAGGCGTGCATTGGAGTAGAGTAATCGAATTGTTGAATCAAGAACAGCTCTGGGACATATATTGGCGTGCAGTAAGCCAATTTAGAGTAGTAAAAATGCGAATGATCATTCTGAATTATGTTTTTTCAGTTGAAACAGCAATCTTTCTAAATTTCTGTTAGTTATCATACGTGGAGCTCTAACTGTGCAGACAGTTTGGATGGGAGTTGTAGAGGTGGTTGGATGTCACTAACATGAAATTGAAATCTTTCCTCATTCTACAAGAACAATGAAGGGATGCAAATCTTGACAAGTTTAAAATAATAGGGAATTGGGTAAAAGATTGGTATCAGATCCAAACGGTATAAAAAACCGATCGGACAATCTAACATATTGAGTGAGTGCAGGATTTTAGCATAACTATATTTCTAATTTCACAAACTATGCTGTCACATCTAATGTCCTTATGAGAAATGATCTATGCTAAATTAATGGGTACTTCTTGTGAACATGTAAAAAAGTGTATATTCTTTATTGTAGCATGCTTCCCTTGAAATTAGAAGATTACACCGTTTAAGATTAAATAAAATAAAATTCAAAAGATGAAATTAAATGGACTGAGTCTTGTACTGTGGTAACTAGCTGATTTTCGTGTAAATTTATTTGCATGCATAATTTCATTTGATTTCTATATTCCGTTTACTTTATATTGACATTGTTTTTTCCTATCCCATGTCGTGATCTATTCATACTGGAGTTATAGCATGCAAGGAATGATTACTTGGAGTTGAGGCAGGAAGCCACTGACCTTCGAGAGTATTCTAGTGCAAAACTTGATCGAGTTACTCGCTATTTGGGTTTTCTTGCAGATAGAGCACACAAACTAGGTAAGCATGTCCTTGTTGCTTCAACTTAAAACGTTAAAATTTATTAGGTTCTCTCTATTGTTCATTTTTCCTAAGCTCATCCTAGATAAGCGGAAACATTTGTGTTGATTAAAATTTTCTATTAATCTTCTAGTTTGTAAACAACTTAATATTTATTAAATTTGTTTATTAATCTTCTAGTTTTTAAATTTTCTCCTTTTTTTCTAAGTTTTCTTTTTTTTTTTTTTTTTTTGACTGTCTCAATGATTCAGTTTTTATCAACCTGTAGAAATTCTTGCTTCTAGGGTTTAGATTGTCATCTACAATCCTGATCTTTGATTAATACTGCCTATTTTTAAGGATTTTGTTCATTCTAACGTTCAATGCCCCCTCGGGGTACTTCAGATAACATGTTTTTATTTTTCTTCATCTACTTCCTTTTTTGTTTGATAATAGAACTTGGACTTGATTCATAATTTATTTCTAACTACTTGTAGAGAATTATATTTCTTAGAGAAACAAGGACCCGGCACATCATCATCCAATCAAATTCAGCTGTCATCTGAAATTTTTGATAAGAGAGCAACATTAAGGGTGATGTGGCTTCAGTTAGAAATGTAGGGTTGCCACTGAAATAGGGAGAAAATGCAGAAGTATTTTTTTTTTCTTTTAAAAACTTAGCTTAGACTTCGTTATTGTATCTTTTTCCTGTATACTTACTATATGATACGAAGCTCATTAATTTTCAACATGCGTGCTACTCCAAAATTTCGTTTAAGCTTATGCTCCCTCTTGTCTGCAGACCAAGCTGCACTTGAATCGGAGGCCAGAATTACTCCATTGATCAATGAGAAAAAAAGGCTCTTCAATGAATTATTGACTTCGAAAGGCATGAATATGAGATCTTTTATTCACTAAAACTTGCAAGGGTCTCTTTTTTCTTTGGTATCTCTTGGGTTTGGTGTTTATGACCTGAAATATTAATTTCAGGCAATGTAAAAGTATATTGTCGTACACGGCCGCTATTTGAAGAAGAGGGCTCATCCGTAGTAGAATTTCCTGACGACTTTACTATTCGTGTAAATACTGGTGATGACTCTTTGGCCAATCCTAAGAAGGATTACGAGTTTGATAGGGTATATGGACCTCATGTGGGGCAGGGTGAGAACCGAATTCCTATTGTCTCTCCTTTATGATTATTTTATGCTTCGAATCTCGAAATAGTAAATGATTTTTTTCCTCTATAGGCAAAATGGCTGTTCTATCTGTTTTTGCTGCTTCTTTTCTAATTTGCTGATTCACTTAACTGTCAGGGGAACTTTTTCGCGATGTGCAACCATTTATACAGTCGGCCTTGGATGGATATAACGTTTGTCTGTTTGCATATGGTCAAACCCATTCTGGAAAGACACATACAATGGTTTTCCTCTTTCCCCCCTCCCCGAGATATTCCTCTGACAATAACATGTGTACTGTTTCATTATTTATAGCTCTAGTCTCTTCAATTGCCCTTCAAAATGGACTATTTTAATTGCCTGATTTCCCATAAATAGTCCGTTGTTGTCTCACCTTCAGTTATTTCTATTGTGTTTGTCATGTGTTTGAAGTATATGCCAATGACTGGTAGTTGTAAATTGTAGTTGTATTTAGTGTATCCTCTGTAGACTTTCCCTGTTATATTGAAGACTCGATTTGTCTTACACTATGGGGATAATGGTGTTTTCACATAAAAGTGAGTTGTCATAATGACAATCGTAGCGCGATGCTCTTTGAATATTGGATGCTTAAGGAATATGGGTCATGACTGAAATCCTGTGCTGACTCTGCACTAATATTTTCCCAGACATGCAAGTAATGCTAATTTATATCATTTGTTTGTTTGAGTTGATTTTTTTGCTTTTTTGCTTTTTGTTTTTTTTTCTGCATGAGAAATGTTCCTCTGGCAATTGAAACAGTCTTTTCCTACATCGTTGTGGAAATGAACAACTATACTTACTCAGTTCCCTTCAGGATGATGTTGCTTTTGATGGTGTGAAACTAATTTTCTCTATAAAGTTCAGAAAAAACGAACTTCATGGCTGTCCAATGTTTATTTTCTGTAATTCCTTTAGTACATGCGAAGAATGTTGAGGAAAATACATTGATTCTTCATATATCAATGTGCCTTTTAGGTACAAATGAAGTGCAAGTTGGTTTTTAGCATGAATAGTCAGTATTTCCTGCCAACATCGGGGCATGTTAACTTTACCATACGTCCATGTTTTGTATTATTTACACATTGGAAGAGTGTTTATAATATACCGCAATACCATGAAAAGACTATTGCTATATTTTGATCTACGAATCAATAATGATTTGAAGTTTCTATGCATCATTAGTCTGTTTTAATAGTTTGCGTACGCTGGTGCCTGTAGTAAGATTGACTTTTAATCCTGGATGGAGAAAATTGCTCATATTTTCTGTCTCAGTTTTTAATGAAAAAGATTTTATTCTGTTCTCAAGGTTGTAGTCCTCTTATAGCATATGATGCAAAAAAGAGGGGATCTATTGGCTGATGCATGGTGTTATTTACTTTGCATTAGAACTTAATGTTCATGACATGTTGTCATTAAGTTTCGCCGTACTTTGCTGTTTCAGGAAGGATCTATCCATGAACGTGGCTTGTACCTGCGGAGCTTTGAGGAGCTCTTTGATTTGTCCAATTTAGATACTACTTCGACATCTCAGTATAGTTTCTACGTTACTGCCTTTGAGCTATACAATGATCAGGTTTTTCCTCTTGATTGCTCCCTCTGTTTTCTGAGACATCTCTCTACTGTCTATTTTATTCACATTTCACGGCCAGTTTCATGTCATTCATGCTCTAACATCTCAACTTTTAATTCATATGGCGAAAGAAATTCTGTGCACATCTACTATCTTTCAATAATGTATATCTGATTAATTTATTTTCTTACATTGTCCCAACAATAGGTTCAAGACTTGCTGTTAGAATCTAGAAGTGCTATGCCAAGAGTTCGTATGGGTCCAGAAGATTCCTTTGTGGAACTTGTGCATGAAAGAGTTCAAAATCCACTGGATTTTTCTAAAGTATTAAGCGCAGCACTTAAAAAACGAGGGACAGACTCTTCGAAGGCCATTGTCTCTCACTTGTATGTCAATGAGTAACAAGTATATAGACCGAGTAGGCTCAATTGCTTGATATTATTGTTTGATTTTAATCATCAATACTATCAGTTGGAAATTATCATAGTGCCTGGAGACCTGCATTCTACTCTTGACATTAAGTGCTCTACATTGGACCTGTGGTTCTAGAGGAATGCACTTTTCGAGCTCTGTATTTTTCTTCTTGCTAAATTAGGTGGAATATTATTTGTCATTGCATTTTATTGATGCTGGCTTTGCTTTTCCTATTTCCAGGATTGTCACTATCCATATACACTTTACCAACTGTATCACCGGCGATCGTATATACAGCAAACTCTCTCTTGTAGATTTACCTGGAAGTGAGTGCCTGCTTGTAGAAGATGCTAGCAGGGATCATGTAACAGACTTTCTGCATGTGTCAAAATCACTTTCGGCGTAAGTATTTTGTTTGTGATGTTGTGTTCTTAACTTTTTGGTGTCTCCCTTAATATTGCTTACACAACTGTAACATAGAGGCAACAGTAAAGTAGAGGCAGGAAACTTTGTCTCGAGTGTTCTTGTGGCAATTTCAAAATGCTTGGTTTTCTATGGGTGTATCATTGTCTAATGATGTTCCCTTTATATTTCAAATGTTATTCTTTCTGTATCAGGTGCTCCATATTCATTATATTCAATCAACAATCATATTGTTACCCTATAAATTTTATTTTTTGAGTAGGTTGGGTGATGTTCTGTCCTCTTTGACTTCCAAGAAAGAAATTGTGCCATACGAAAATTCAAGAATGACGCAAATTCTCGCCGATTCACTTGGTAAGTAAATTTTTTTTTTACATCAAGTGAAAATTTGCTGTCCTTTGTTTGTGAGAGCATTAATCATTCTGTGCATTTTTAATTTCCATCGTGTATGACTAACTAAACAGTCGTGGCTGGAATTTACCTGGTTGTCTTTATGTTTTACACAAATGGGCAAATTTTCTCAGCATGCTTCTCTTTTTCAAGTTCCAGTGCATCACCTGCCCTACTTTTCTTTTATTTAGGTTGCATTTTTTCCTGTATCATCAACAGGACAAGGTTGCTATAAACCTTTAGAATTTCCATAGCTTGGAAACTATGCACATTCTTACATCTTCATCGGACATAATTATCAATGATCTTCTAACAAGACCTTGGGAAAACAGTCTGCTATTTTAACACAAAAATCCTTATATCCTATACAAAAAAAAGATGACAATCTGAATACGGTGAATGATTCACTGGTTTCTAAAGGTGGTAACGATTCACTGTGTTCCTCATTTTTGTCAAACTTGGCGCTGAGTTGGCAAATGGAGGGTTAGTTGGCCAAAAAAATTTGGAAAGGTTCATTTGGAAAATTAGAAGTTTTCGTAGGGTTATTGTAAAAAAAAAGAAGTCTGTATTTACATCTTTGTATTTTGTTTCTTTCTGTCATGTGATGAATTAGGCATTTTGTGATGCTGCAGGCGGTGGCTCGAAGACATTGTTGATTGCTCATTTATGTCCAAATGCTTTAAATTTGCCGAGGACACTGTCCACCCTTAATTTTTCTGCAAGGGCAAGAAATACTGAACTAAGCCTTGGAAATAGAGACACCATTAAGAAATGGAGAGATGTAGTAAGGCTTCTTCTATACCCTTCTGGCAAAGGAAATATCTTCATGTGTTTAACCTTTCTTTGATCAGGCTTTGATAATGACAGGTCTTAGTTCTGTATTTGATATTGACTTACAGGCAAATGATTCACGAAAAGAACTGCAACAGAAGGAAAGGGAGGTTCAAGATTTGAGGAATGAAGTTTTACAGCTAAAGCTTGCTCTTGATGGGGCCAATGATCAGTGTACTCTCTTGTTTAATGAAGTTCAGAAGGCTTGGAAAGTTTCTTTCACACTTCAGGCAGATCTGAAGGTCTCTACTCTTTTGTCTTTATGAATACTTAAGTGTGCTTGATTTTTGAATTTCAAGTTCTTCATGCTTGGACAAAAGCGGTATGTCCATTTATTGATACATTTGTGATGATGCACGTTTGAGAGGCTTTAGATATTAGATCGTTCCTTCTGGAAAACATTTTTTTTAATTAAAATCACACATTCTCCTTTACTAAAACCCTAGTAAAATGCGCCCACAAGGAACCAATAAAAGTACAAATTAAAGGGGGAGAAGGATGAGAAAGGTAGTATGTGCAAGTTTTGTTTGTCACCTATAATAGGAATGATCAGATTGAAATCTTTCCTGCATCACTTATGACAGCTTTAACTCTCTTTCACTTTGGAATCGCATGCCATTTGTACGAACTGTAATCTTTTGTTGTACATAATGCTGTGTGATATTGCTTTCTGTTAGCTTCTCTGCCAAACATATATCATATATAAATATCTGCTTTGAAAACATGAGATAGTGAGAATGTAATGTTCCTAATTGTCATGTTAAATTTTCTGCTATTTGTACATGCAAATTTCGCTAGCTTTCAGTTCGGGACTAACTATTCTTCTATGATATCTTCATTGCAGTCGGAGAATCTAATGCTTGCAGATAAACAAAAGGCCGAGAAGGAGCAGAATAATCAACTGAAAAATCAAGTTGCTCATCTTTTGCAACTTGAACAAGAGCAGAAAATGCAGATTCATGAACGTGATTTAACCATTAAATCACTGCAGGTTAAACTTGGTGTTTCAGTATATTTTAAATTTTGCCTTCTCTGATTTGTGTTATTGATGGTGTCTGTCTGCAAACATATATGCGTGTGAGCATGGATTTGCATGAGTGTGCATGTTTTGATATGTACACTAACCTGCAAAACGATCTCTTCTGTATCTATGTGTTGATGCTGTTGTTTTTGGTTGTCTTTTGTTTTGGCAGTTGATTTATCTTTTTCTGTTGCCACTTTGACTAACTTAACAATCACTTTTTTGATCGATTAACCTTTTTAATCTTTCTAAACTCTATATTATTAACACATCCGAGATGGGTCTCCCTGGTTGGTTAGCTATTGCCCTAGAAATACTATGAAAGCATTTGCTATACAATTTTCATTGGTGATGCCAAAACTAATGATACAAGTTTTAATTATGGCAGCCCTAGATGTGTCCCATTTAGTATCAACAAGCTATCATCATATTTTAGGTTCATCTCTTTCTATTTAATCTTATGATTTACATATCATATCAAGAGACATAAACCTACTTCCCTGTCATCTTTTTGCAACGAGTTGGTTGATTCATGTGATTACGACAATACCAATTCTTCTTAAGAGTGGCTCATAAAATGTGGTAGAGGAAAGTTTCAAATCCTTCAGAATGAACCATCACCCATTTTTTTTTCTGCTGCTTAATGTGATGCTTTAAGAAAAGCTTTGTACTCCTTATTTGTGTAAAATAGTTTAAGAAGCAGAACTAAGCTAGCAGTCTTATTTAGTCAAGAATTGTGACATTGAGCCACCATGAATTTCTGCCTATTTTACACAATTTTTTCCTCTAACATGCATAATTTTGTTGAATTAAATTTAAAATGCAAGGTTCAAGCACAAATTTTTCCTCTTACATGTTAAGTTGTATTGACTACACTTACATATTGAACTGGAGAGGTTCAAAGTTGTAGATTTTCAATATCATGTGTCTCTATTAACTGCAGAAGCTGATCGTTTTGCATTAAGAGCATGACGATATATAATTCTCAATTTCTTTCTATGTAGGCTAAACTTAAGACCATTGAATCTCAGCTCAATGAGGCCCTTAACTCTAGTGATTCTAGATCTACTCTTGGATCCGAGTCTGGTTCTACTGGGGTTTTGTCAACTCCAAAGATGGAGGCTAGTGTAGATTCTTCATCGGTAACCAAAAAGCTTGAGGAGGAACTCTCAAAGAGGGATGCACTTATTGAGGTACTATTTGGCTTGCTTGAGCTAATTAATTCTATTCATGTTTAGTTACTATAGATGTTTCTTCCCATATGCTCAGTTATGTGATGTTTTTTTTTCCTCCCATTTTTGATATGGGTTTTTAGCTAGTGACATTTTATCCTAGGCAGTTTGCATGCATGCATGCACATGTTCATGTGATTGTCTTTATCGTGGGATTTGTAGTTCCATTCAGTATCATCCCCTTGCTAATTCATTGTTTATTTGTACCACTGTAATGCGTGCAGAAATTACATGAAGAAAATGAGAAGCTCTTTGACAGGCTTACAGAGAAGTCAGGATTAGGAGGATCGCCTCAGGTTGCTTTCTGAACTTACGATAATTTATTCTCCCTAAAGTTGATATCAATGTTTACTAGGTATTAAGAGCTTCAGTATATTAATGAATAATGCCTGTCTAGTTTACTCCTATTGTGCTCCACCACTATGGATGAAAGGGACCACATTTTGTTGCGTGCTAACTATAGCTAAGTGCCAGCAAGCCATGCTACATCAGATGCAGCTTTCAGCACACTATTTGCAGTGTCATGTATAGTCTGGTTCAAAACTGATTAAGTCAAGCACAGTGCTTAAATCATCTTGAACATGTTGAACTAATGTTAACCATAAAATATGTTACTTCTGTTGAATGTGGAGGTGTTTTTTCATTGTTTCTTTACTTCGAAGGTGCTCAACAAAGTTTTAATATTTGTTGTTATGACTGCAGTGTGTATCATAGATTCATAGAGCTCGCTTCAAATTCTGATATGGGATTAGGCACTATTCAGCATATATGTGTCATCTTTTGGTCTTTTTTTCATTCCATGTAATACTACCTTGAGATCTACTTACCTGTTATGACTACTTTGACTCTTTTGGATAACTTTAATGGTGTTGCAGAACATATGTTTAAAATGTAATATGTTTACTTGTACAGTTATACCAAAATACCGTGTGAAAAAATAAAACTGTTTTATTGGTGAAAATGAAGGGCTGATGCATTAGTGTGGAGTTTATCCTGTTTACTTATCTCTGTATCTTTTTTTAGTCTTTTCTCTTTTACCGTTGCCTGATATTATTTATTCATGACCTTTTTACGAGGACAGGAAGGTTCCGGCTGGAAGACAGTATATCCTCTTTACCCTCACAATATATAATATTAAGAAGCAAGATTCCTTGTCAACAGCTATTTTTGCAAAGATTACAGTAGCAGTGAGCGATCTTACATTGGAGTTTCCATGTTAGCAGCTAGTTTATGATCATGTTATAGAGTGAAAACTTAGATATTTGTCAACTACAAACAATAGAAAGTTCTCTTAAATTTCTGGATTCTTTTATAGTTTGGTGCTTTATTACTGTACATTTTGCTCAGCCATGCCTCTGTTGTGCATTTATTTGCATATCTTAGCATGTGCTTTCTTATACTTGTGACATGTAAACAACTCATCAATTTGTGTTTTTTAAGTGGATTTGAGTGTATGCTGAAGTAAATTGTTCAACTACTTTGTAGGTTTCAAGCCCTTCTACCAAAAGGACGGTCAACACTCAGAGTCGGGATTTGAGCAGGTATAGTGTTTAAACATTTGTGCAGGTACATAGTTAAATTGTGCTTCACATTGGGTAAGGATGTACTTTCTTGTTTTCACTTGTTGGTAGGAGCGACACAAGCAGAGGGCGGTCGCAAGATGTCCTGCAATTGCCGAGGACCCAGGATAAAACTGTAACTACTGGAGCTCTTGTAAAATCAAGCAATGAGATTGCAAAGACTACTCCTGCAGGAGAGTATCTTACCACCGCACTGATGGACTTTGATCCGGATCAATTTGAAAGCTTTGCCGCAATAGCTGATGGTGCAAATAAGCTTTTAATGCTGGTATGATTATCTGAAAGTATACCTTGCTTGTTAAGTTTCACTTATTTCCTTTAAAAAACTGCAAGTTGATATGAATAAATAAATTATGATTCATTCTGGAAGATTTAAATTTGATATTGTCTAGCTTGAAGTAGATGATATTTGCAGGATGAGATACTTAGGCAGGTAAAGTCTTTTCTGAGAGACAGAGAGAGGTATTATTCTTTTGCGACCATTGGATGCAGTGTAGTGATTGTACAGACTTAGTTCTGACAGTATGTTGGGAGCGAATATCTTAATTTCTATACTAGATGTTTTATTCCTGTAGGGACCTGTCTGTTGGTTTTTATATATCTGTTCAAATGTTTGAAAGTAATATCATTGATACTTTTGGTTGACTCTGCTTGATGGGACAGTAAAACTTATAAGCTTATTCCAGCTTTTCATGCAGCTTTTGCTTTTCCTTGAATTAAGATATTATAATTATCTATAGAAAAAGTTTAAAGTGGTTTTGATACTGCTAGTTCTAGTAAAACTTTTCAGTACTGAACAAGACTAAGTTGAATAGGGAAGTAAGCTTTTCTGTGCTGGAAGGGATATGAGACTATTTTGTTGTAACTTCCTGTTGCATCTGTTCTACATCTGTTTCTTTCATACTAACTCGTCAGAAATCATTTCCCATGTCTGTTTCCCATGTCTTTTTACAAAGTATAAATTTGGAAACTCTGTACATTGAAGCATCTGTGCCCCTGCTGCAGGTTTTGGCTGCAGTTATCAAAGCTGGTGCAGCACGAGAACATGAAATACTTGCCGAAATCAGAGATGCTGTTTTTGCCTTTATTCGTAGGATGGAACCAAGGAAGGTTATGGATACAATGCTAGTTTCACGAGTCAGAATTTTGTACATAAGATCTCTACTTGCTAGATCGCCAGAGCTGCAATCTATTAAGGTATATAGTTTTAATTCAAATTTGTTCTGTTTTCATCAGATTCCTCATCCTCTTGCATTTACATAAGCTGTTGCTTTGCCACCTTCTGCAGGTTTCTCCAGTTGAACGCTTTCTAGAGAAAGCTAATACTGGCCGTAGTAGAAGTTCCAGCAGAGGCAGCAGTCCTGGTCGATCTCCTGTTTATCATGATTCCAGTACAAGAAGTTCGTTGATAGATGAGCATGTCCATGGATTTAAGGTCAATATTAAACAAGAAAAGAAATCCAAGTTTTCTAATATTGTCTTGAAGCTTCGTGGGATTGACCAGGTATTGATGTTAATGTCCTTGTATTATTTATTGTTTAACCCTTTTCCTTAATTCTTGTGGGACATTTTTCTTTGTTTGATTTTTAAGAATTTTTTTGTGTCATCAATTTTTATCGCTCAAATATTTTTTAGATTTTTTTCTTTTTTTCATTTCAGTAAAATTGCATATAGGTCCTCTTGAGTATAGTTCATATTAAAATAGGCTAAAATTTCCTTTTTCTGATTGGCCACCAAGCAACTTTTGCTCGCTATGATTTGAGTTATTTCAGACAGTATAAAAGTTAAGGTGATGCTTATACCCAAAAAAAAGGGTCTTGAGGCCTTGAGGGGATCTTTATACATTTTCACCTTTTATTTGACCACTTTTCTTTTATTACTTCTAATGTTCAAATGGACCTATATTTATTTGCAACTCAACAGGAGACTTGGAGACAGCATATTACCGGCGGGAAGCTCAGGGAGATAACAGAGGAAGCGAAAGCTTTTGCTATTGGAAACAAGGCTCTTGCTGCTCTCTTTGTTCATACACCAGCTGGTGAATTGCAACGTCAAATACGATTGTGGCTTGCAGAAAATTTTGAGTTCCTATCTGTTACTGGTGGAGATGCTCTAGGTGGAACTACTGGCCAACTTGAACTTCTGTCCACTGCAATTATGGATGGATGGATGGCTGGTCTCGGTACTGCACAGCTTCCTTCAACTGATGCTTTAGGGCAGCTTTTATCAGATTATTCTAAGCGGGTGTACACGTCACAGTTGCAACACCTGAAGGTTCTCTTTATCTTTCTCACAATTTATTTCTAACAAGATTTGCCTTATTTTAGTGAAACAGTTTATGTTGTTCTATCACTTGATTTCTTATGGCTCTCTGTTTACATTGCTATTGTATGTTTGGCCCTTTGTTTTTGTCCAGGAGATGCTGTATTTTTGCTTCATGATTAATAACTGTTTTCGTTGTATGGTATCCATTTTGTCCATTTAGGACCCACTAACCTTGCACATTTTTGTTATATCCGGCAGTTTAAATACTACAGATCATAATCTACATTGATATTCTAAAATCCAGGTTTGTTAGGTCACGTTTTCTGTGTTGTTTATATTTCCAATCAGATGTAGTATGTCTCTCTATTTGTAATCATGCATCGTGCTACCATTTTGGTATGACAGTTTTGGCTTGTTTCTATGATCTGTATAACATAAAACAATCCAGTTCTTGTTTCGTGTTAACACAATACACTTACAAGCTCTCTCTACATGCATGTATTCTGTGTATCCATATGTCAAATATGCAATATTTACATTTCAAATTTTGTTTGTGATGTCAAGTCAGCTGGTGATATGGATTTTCAAATTACTCTTCTTATTCCCATGTTACTTGTTTTTCTGTAGGATATAGCTGGTACATTGGCAACTGAAGAGGCTGATGACCTAGCTCATGTTAATAAGCTGCGCTCAGCCCTGGAGTCTGTTGACCACAAAAGGAGAAGGGTAACTGTCGATGCTTTTGAACTATCATTAATTCATTTATTTTGCATCATCATTCATTTCATTTTCATTGTTTTTTTTTTGGGCCAAGCTGTTGGGTGTTACTATCATGAATCCTGTGAAGACTCTGATTACCTTTTTGTCCAGTTATTTGTAGAAGGACATGGTTGAATGGAACTATGCATATTCTAGTAGTTATCCTGGACAATTGTGTGCTTTTAGGTATCGCTAATAGTTGCTATATCAACCGTCCTTTTACATGTTATCAGATTCTGCAACAAATGCGAAACGACACTGCCTTGTTAACAAAAGAAGAAGGTGGTTCACCAATACAAAATCCTTCTACAGCAGCTGAGGATGCACGGCTAGCATCTCTCATATCTTTAGATGCCATCTTAAAGCAAGTCAAGGTCTGATCCCATTGACTTCATCTAGTATACTGCCTTGATTCATGCCAGTAAAATGTTTTTGTTATGTTACAAGTTCAACGATAATTTCACAATGTTAGGAACTAATGAGGCAAACCTCAGTGAGCTCTCTGACCAAAACCAAGAAGAAAGCGATGCTGGCATCACTGGATGAACTTATGGGACGAATGCCTTCACTTCTGGACATTGATCATCCATGCGCTCAGAAAGAGATTATGGGAGCACGTAGAGCTGTAGAGGTAAACAAATAATTAGCTTGGAATTTGCTTTTAGCATATTTCTTGGCGATTTGCTCTTTCTTATTTTGAACTTCAAAAGCAACAAAGGAAACTATTTTATTCCTTTAGATTTTAGTGTCAAAAGAATGTGCCATAAGAATGTTAAGTCGAGGATGCCAATATGTTTCCTTTTTGGAGGAATGGCCATATGCTATGGCAGAGTTCTCGGTCATTATGGGAATCACTTTATCTGTCGTGTAAAAAATAGCAAGATTAGCCATGTTGTATATATGTGTCTTGATAATGCTCATCATGAACATCCTGTAATGCTTTTTATCACTTTCTAGCCATAATGCTACAATAGAAAGTGAGCATGCTTTTAATCAGAAATCATGCATGAAAGTGAAAACAATTCCTCCATTTTGCCTTTCGCCACTGCTGGTAATGCCAATGACCTACTTAACGTGACACAATAAATCTCTGCAGTCTATACCAGAAGTAGAAGGCCAATTTGATGAAGCACCTCGAGGTCTCAACTCTTATTCAGAGTCAACATCCACTGGGGAGAGCGAAGTTTCCCAGTGGAATGTCCTCCAATTCAACACAGGCTCCACAACCCCATTCATCATAAAATGTGGGGCGAACTCCAACTGCGAGCTGGTCATCAAAGCCGATGCGCGCGTCCAGGAGCCTAAAGGAGGCGAGATTATTAGGGTTTTGCCAAGACCTACGGTTCTCGCAGATATGGGCTTTGAGGATATCAAGCAAGCGTTCGAACAATTGCCCGAGTCGGTTAGCTTGCTTGCTCTAGCACGGACGGCTGATGGTACTAGAGCCAGGTATTCTAGATTGTATAGGACTTTAGCTAGCAAAGTCCCAGCCCTAAAAGATCTGGTAGCTGAGCTTGAGAAGGGTGGCATGCTCAAAGATGGGCGATCATAGATCCTTGTGTTTTTGTACTATGTATTTATATCATGTACAATATGTATATCATTGCGTCCTGAATTTACAGAAAATTTCAGCGAAGACTCTTAGAGGGGTTCTTTTTGTTAATTGACAAGCTTAACGTGGTATTATTTTTGGTAACAAGGCGTGTTGAAGTGTTTTCGCCTCTGGGAGTACTTCGCGCAGGATATTTACATATGAAGGCAAGCTTGTTCATCTGAGGTGAGTTGTAGTTCAAACTCCGTCCGCACTCCTATCTCTTTGAACTCTTCCATCAGTGTTACATTATTACCTGTATATTCCAGCATAAGAAAAAAAGGGAGAGATCGTCTGCTGCTTGGGGGATTATCTATGTAGACTTTCTTTTTATCGATTCTCTACATGTAGATCAAAACGAAATGCTTTTGCAATTCTTGCTGGTGCTATGAATGGCTTGAGGTAGATGAATTTAGGTGCTTAAAATAGGTTGCGGGGTTTGTACTCTGTAGTCGTCTACGATGGAATTGCTCAATCAACTTTAATGAACTTTTCTCTTTATTACTTGTCAGAGATTCTCAGGATCTTATTTCTTTTCACCAAGTGATGTTAAATTTCAAGATGCTGCTGGCAATCCTTGTAAAATGAGATCGTTTTCGCTACTCCCCATTTTTGTTGCTAATTGGTTGACTCGGGATAATCTTTTGTACCCTTCCCTGCGTACGCCTGGCGGCAGGTAATTAGAATATTATGCTATTAAATTAAATAACGACGTGTGGATAGATTTTTTTTTATTATTATTATAATTATTGTTCATGAGAAATTGACCAAATTATGTGTCTAACATCCATGCAAGCAGAGTACAATCGGGAATCCGCAGAATGAAGCAAAAATGAGTCCTCGCAAAGATCGGACTTCGGAGTCGTATGGTGGTGTAATTAGCCCAAGTAAGCTCGATTTTCACCACCCAAGTTACAAAAAAAAAAAATAATAATAATAATAAAAAACAGTTGTCTTTCGTTCGTTCGTGTAGCGTATGGTATAGCGTTGTACATATACTGCGACGAATACCCAAAGAATGCCGACTACTGTACATTACTGGTTCGGTGGTTCCATTGTTTATTTTCCGATCCCGCCGCATCTTCGTTTCTTCATTTTTCGATTCTTTACCTCTACGCCTGTTCCCTCGCGAGTCTCGACCGGACTTTTTTATAAAACAACCCTCTAAAGTTTTGAAATTTGTGAAATAACCCTCTCAAAATTTTATTTGGAAAACTAACCCTCCTTTCGCCACATCGGCGCCACGTCAGAATTTCTTTTAAAGGCGGTGAATCGTTCACCGCTTTCTCCCATTTCTTTTAAAGGCGGTGAATCGTTCACCGCTTTCTCCTATTTCTTTTAAAGACGGTGAATCATTCACCGCCTTTCACTTATTTATCCCATATATAATCCCAACAACCACATAAAAATTCTAACAAATCATATATAATCTTAACAATCTTTAAATCCTAATAATTTATCCATATAATTTCATATAATCTTAACAGCCTGAAAATCCTAATAATCTATCCATACAATCCTAATAATCAAATAAAAATTCTAATGATTCACATATAATCCTAACAACCTCACTTTTTTCTATTTCCTATATAATTCACGCACAATTTTAATGAATGCGGTGAACGATTTACCGTCTTTAAAAGAAATAGGAGAAAGCGGTGAACGATTCACCGTTTTTAAAAGAAATGGATAAAAGCGGGAAATGATTCACCGCCTTTAAAAGAAATGGGTGAAAACGGCGAACGATTCACCGTCTTTGTAAGGAAACCCTGACGTGGCACCCACGTGGCGAAAGGAGGGTTAGTTTTACAAATAAAATTTTTACAGGGTCACTTTGCCAATTTTAATTTTTTGGAGGGTCATTTTATAAAAAAGCCCCTCTCGACCACTCCCATCCTCTTCATTCTACACCCCCACCCTTGTCCCTCCTCTTCTTTTCCCTCGTGGAAACCTAAGTAGGGTTCCCTTCTACGTACTTTTATCTACAAATGTCCTCTTCCCTGACCTGCACCAGGCTCACGATCTGGGCTTTTGCGGCCGTCCTGCTTCAGATCGCGGGCCTCTCTCTCTTCTTGATCGGATTCTTCCCCGTGAAACCCACTCTCCCCGGATTCAGGTTGTTCTTTCTTGATCCAAGATCTGGTCCTGTTTCTTTTTGTTAGAACCCTAATTTGGAATTGGTTCTCATTTTCAGCGGGCCAGAGAGTTACCGGATGCCGACGAGTGATCCCGTTTCCGATGCCGAAGAGGTGGAGGAGTTGCCCCCCGATCGGCTCAGATCGCTGTATAGGGTTTGCGCATTCTCGTCTACTTTGCCCTTTTTTTCGTTCGTTCTGTTTTCTCTATAATTTGTTGTTAGAGAATAGGGATAATGCTTAATTTGGATGACTCAAACTGCATTATATCAATTGCAACGGGTTTATGTAATTGATGCCTCTGCTAAGATAAATTGTGAACAAAGGCTCAGGTTCTTGAGGGAAATTGAGGGTAAAATGCCCACAATTTTGATCAAAACAGGGAGGAAGAAACTGTTTAAAATGTTATTAAATGGATGTGTAGAGGCAATCCATTACACAGCCCCACTTGATCCGAAGCCAAGATATTGAGTCAGGTAACAGGTTCTTGAATTTATGCTGTGTTGTGTCTGATATGCATGATTAGGATTCTATCAGTTGAGCCAGAATTAGATTGTTTGGAGGGTTACTAGTTTCCGAGATTAAAACTTCAAGATTCACTGGGTCCACTACCTTCCTGAGCATCAATAACTACCAAAAGCAATGATCTGTAATTTGGAAGAAAGGCCATTCTTTTTATGCATGAGAAAAAGGTTTTGAGAACATAGCATCTTTTAATATACACATTTAGATGTCATAGATCACGTCATCACATACACATATGTTTACAAAATCTGACCTAACAGCATCATTTGGAGAGCTAAGCAGTCTTTGGATGGAACTCTTAATAATGATCTTTATCCAGATTTCACCTTAGAGTGCTCTTTTAGTAAGATTGTTTGGAATATGAATTACTCTTGCTGAAGTATATCTTGAAGTTCTTTCCTTGTATTGTTTAATTCTCCCTTTCCATTTTTTCTGTGTTTGGGATTAGTTTTTCGTGGCTTCATCAACTACTCTGAACGATAGGTTCATATGATTATGTGAAGTTTGATGAAGTCAAGACAAAGACAACCGCTTCCTACTAGGAAAAGCCTTAATATTCAAAATCTTTTAATAGTAAAGCTTGACTTGAATTTATTTGCTATTATATGGCATAGATTAAACTTATGATGGTAAAACTTAATTTAAATAATAATTTGTTTCATTACAGGAAGTCTCCAAGATACCTCCGGTCTATGATCGACTAATTTTGATGGTACATGCCTATCATTGTTTTTTTTTTAGTTATTTTTTATTATTTTATTATTTTTTATTTTTGTTAGTACAAAGGTGTAGAATCGAAATTTAATGAATCTATGTGCCATTTGATGATCATTTTTTCCCTCTGTTTTGGAATCTTTTTTGTATGTCAGTTTAGGTTATTGACGGACTACCTGCCGAGTTTGTGCTGGGAAGAGGTAACAAGCCTCCAACTAAGAATATGATGGAGGCTATGCCGTATACCCACTCTTTATTGTCGAGCGGCAAGGCAGCAGCTTATCATGCAAAAGCTGCACCTCCCACTGTTACCATGCCTAGGCTAAAAGTAAGATAATCTTATTGAAGAACAGATAACTAAGCTTTGCATAGTTTGATATATTATGCTAATTTCACATAGAGTTTTCAGGCTATGGTTTCTGGTGCAATTGGAGGTTTTCTGGACGTAGCATTTAATTTTAATACTCAGGCCATGTTAGAGGACAATCTTCTTGGTTAGTTCTGATACCTAATTTGTCATTTCTTGAAAAAGAATTTATTTATTGTGGCATTGTGGCAAGCTGGTCTGCCGCGGCAGTAGGCACAGCATGTGGTGGCCTTGGCCTTCCTGATTGGTTCAGCAATTTAGAAATCTTGTGTAGAAATGATAGTTACTTTTGAGTCAGACTAGTCAGTGCATGATATTGGAATGATAGTTGCTTCCATGTCAGTGATATTCTCAATCCATCTTGTTTGCAGAACAGTTTTATCGTATTGGTTGGAAATTAGTGATCCATGGGGATGAGACATGGATTAAATTGTTTCCAAGATTATTTCACAGACAAGATGGAGTTAGCAGTTTTTATGTAAGTTTTCTCTTTCTTTACTCACATGCTTGTGTTCTAATTTTGCTGTTTTTATAGTTTACTGCTTAGTTCTAGATGAAATCACAAGCTGTTGCCTCGATAACTTGCGATCCAGCATAATAGCTTCTTTTTCTATTTGGTCATAATTCTACATCAAGAGTTTAAAACTTTAACACAACCACGTTTTAAAATTTCATGCGATTACAGGTCAAATTACTAAGGACAATTATTCGCAGTATTAAACATGAATACTCAGCGAGGAATATATTATTTTGACCAGCATAATAATAACTATTTTAGGATTCACTAGTAATCAAACGACCATGATAAGATAGATGTGTGAGTGAGGCATTTATAATTCTTTTTGATCATTAACCATCTGCATTTGCCAAAATATGGTATATCTATTTCAACTATAATGCACAAACCCAAAACTCTATAGAGGTTGGGATTCTATCTCTGAATACAATATTGACGTACTGCTCTATGGTATGCAGATAGGGCAAATGAATCAGTACTTGTGTAGATGTAGTATCCTGGTAATTTATGCAATTAGAGATAGTTTTTAACTTTCAGACTTATAAATTGCTTATTACCGCTTAACATTACGTGAATGATGCTTGCGGTTTTTTCTTTTTACTGTGATAACGGTAGATTACGTGGGGGGATGCTTGGATGTTTGATGATCAGTGTATGTAACTAACTTTCTACACAAGTCAAACTACTCGTTATCAAGTTAATTCTCCTGTTTTCTAGATTTTTAGATCTCCTTTGAGTAATTGTCTTGGAGTCTTTTTTTTCTTGATTCTTTTCCTAACTAGGTCAAGGACACAGTGGAGGTTGATTTCAATGTTTCTCGTCATCTAGAGGCTGAGTTTGCTTCTACCGACTGGAACTTCCTGGTAATTTATGATTCTTTTGAATGTTCCTCGTTTTACGCAGTCATCTACCATGTGAAGATTTTATTATATACTCTTTTTTACTGTTATAGCTAGTTAAACTAGTCTTATGTAGTTTTGTCCATAAAGCCCCGACATTGTTTTAGTAAAAATGGAAAAATGGATGCATAGAACTTCCCAACAAAAATACTTTTAAATATGAGTATATTCCTTGCATGTGCTCTCTTTTGTTTGTCATTTTGTTCTATCCATGACCTTCATTATGGAGACGTTTCTTCCGTGAGCTTTCATGTCCTGTCACCCTGCTGGCATTCTTCTTTATGTTTTTCTTGTTGATCATGGATCTTTTGCTTATGGTAGCTTGGTAGGAATGTAAATTGTAATTCTTGTTGAAAATGATTGTACTAGGCACTGCTGATGGCTCTATCGGGAAACATGTGATCTAACCAGCCAGAGAAGAAACTAATAAATGCATTTTAATGACTCATTAATGCAACAACTTGAGGAGGTTGTTAATTGAATCAAGATCACTGCAAAGTGTTGTAATGACAGGATGGAACTAGTTACCTAGTGACAGTAGGCTGATTTATGTTTATTTTCCAAATTTCAATTTTTTTTAATGCTTAGTACTCCCTCATGGGGCTTGGCAATTGGTTATTTACCGTTAGTGGGTGGACCTGTGGCATTAGAGGTGTGGGGATTTGCCGACTTTTGGCTGCACAGTAGATGTTGCACTGTGCCAAATTAAAATTTTAGTTTGTGTGCATATCCTAGATTCAGCAAGTTAAAAAATATTAATAAATCCAGAATCGTCTTCAAAATCTCAGTGACAAGACAAAAATGCTAAAAGTGATAGAAGTTATCTTTTATAAGTTGCTTTAGTTCTGCCTGTCATAGTAATTCTGTTTAGTCTGCAGTAGTGTGAGGTTTACCTCTGATCAGGCTTCTGACTCCAGAATATATAAGATGTATTTAACTATTTATTGATAAAAGCCACTATTAGTTCTAGTGGTTTTATTAAACTATTTTTCCAAGTGTGAATTATCATTTATTTTTTCCTGCAGATTCTTCATTATCTTGGCTTAGATCATGTTGGACATATTGGTGGGCGTCAAAGGTGTTTTAATTTCTTGAACTGAACTCGTAATAGTACTTTGGCTGTCCACTGCTGTATTTGAGTTCAAGCTTAAAACTACAGTATTTTGATGGCTCCAAAGCTGAAGGAGATGGACGATGTTATCAAGAGGATTCATATGAACAGCATTTTGGATCAGGAGAATTCTGATAGTCACACACTTTTGGTTTGTACTTGTTATACTAATTATTCATGTTTATTCTCAAGTTTCAACTGATTAGATGTATTAGTGAGTATTATAAAACAAGTGCATTTAACCAAAATTTTCAATTCTTAATGGGTGCTTTTTTTGTGTTTAGTTTCATAATTTTCCCTCTTTATTGTTTTCTTAGTAAGTTTTTCTAGTTATCCCTTTGATCTATTTTTTTGTGTTAGAAAATTGGTATTAATAAATTTGTATTTAATACCACTGAATAGTGAAATAGCTGAACCTGTCAAGAACCAAAATGTTTCATTTTGGTCAAGCATTGCAACTGAAACAAAAAACATTTTTTTAATAACTGTGATTACTGAACTTTGGTGCTTATCCACTGTCCCCTCCTTTGTTTTGATATGACTATTGACACGTCGCTCTGCATTCTTCTTCAGGTTGTAGTTAGTGATCATGGCATGACAGATGGTGGTAATCATGGTGGATCCTCCTATGAGGAAACCGATTCCCTAGCTCTCTTTATAGGCCATGGTGTTGAGGAATCAAATTATTCTCCCTACGACCATAATGAAGCCTTTCAAGTAATTCCAAAGCTTCTAGTTCACCAATAATGTAGCTATCACATGACATATTTTACTTTTTATTTGCCTTTTATTTATCACATGATAATTCAACTTGCAACCAATGCTTAAAATGTTGAGCCTATATTGTTGCCTTGTTGGTTTGTACGACTTAATTTTGGCTACTTTATACCGGGTGTAGCAAATTAACAACTTACAGATATTTTATAAAACTAATTATTTTAAAATAGCTGGATATTTCTAAAATTGAGTAATAATTTTATCCCAGTGGACGCTCATCTGTTTACCTTGTCAGATTATTAATCAGTGGTAGTAATGTGTTGGTCAATCAGATGTTTTTCTTGTCATAGTGAACAGTGATAGTCAAATATATGGCCTTAAAAACTTAAAATTTGTTATCTAAGTTCGTAAGTCATAGTCAGTACTGAGTGTCATAAGTGACTCTTTAATCGGATTAGGTAACCGTGTATAATGCAATAATGAGTGGTTGCTGTCTGAGGACATATAATCACGCATGTGGCCACATTGGAATCCAATGTAATATGGAATAGTACCGCGAAGCATATGTGTATGGCTGTCATAGAATCATTTGAAGTACCATGAGTTTACTTGAGGTAGTAGAAGAAGAAAAAAAAAATCCTCTCCGAATTTAAGAAGGTACATGAAGAGAGAAATAGTTAAATAGCAGTTAATGAGACGGTTAAATAGTTTGATAAAATTTGGCCTATTCGTTATTATAAGCATAATGTATCAGATTTGAGTTGATAGGTTTGGACAGAAGTGGCAGACTATTTAGTATTTTGTCGCAGTGGTGACATGGAATTTGAAGACACTTTCTTGCATATAACTCTATTTCAATTCAGGTGCATTATTAATAATTCAAGTTTTGGGCTTCTTTTTTTATCACATTATCAAGTTGAGCTCTTTAATCTTAAGGAAAGTTTGCTTCCAGCCAAAAAATGAATTTATTTTTTTATGAAGCTATGAATTTTCATTGAATTATTCTTTTTTGCCCTCTAACCTATTGACTTGTTTCCCAGGTAGACATTGCTCCAACTTTAGCTCTTCTCTTTGGCGTACCAATTCCCAAGAACAATATAGGAGTCTTGCTCACAAAGCTCTTTGATTCCTTGACAGGTCATTTTCACTTTCTTCTAAGTTCATCTGTATTTTAGTATCTAAGGAGAGAAGAATGCAGCTAGCTGGTCGATCCTTTAGAGAAGAATGCAGCTAGCTAGTCGATCTGTTAATTTATGGTCCACATTAATTACATATACTAGAAAAGACTGAAATCAGCTCTAACTTCAGCAGATGTTGTTGAGCAGCTAACTTTCATCCTAGCAACAGTGAAGAACCAAAGAATTGTTTTGTTGTTTTTAATTATTAGCTATGTAGTTCTCTAATGGGGTAGCTAATATTGATAAGCATGATGGCCATAACTTCTCAAATTTTGCCTAGAAACTGTGCGAGTGACGAACCAGGCAAACATGGAATTTGATGATGATGTTCATCGCTTGCGTAGTGTCCATTGTCTCTTTGGATGAACCGGTGGATCAAACTGGTATATAAGGTTACCAAAGCTAGTAGTTGATTCAGACATCTGAGCTGACAGCCTGGCTGTTCGACAGTCCAACTGCGGAACCGGGCGGTCCGCCACTGTAAAGGGCAACATGTTTGCACTTGATGTTGATTTGTAACTCTTATAGTAAATCCATGGTTGAGGCTAGAAAATTTTTTAGTGTTATGATCATGAATCCTAGCTGTTGCTCTCACATTTTTCTCCTTCTCTTGGTTCATGTTGTTCATGGATTACAGATGATCAAAGGCTCCGATCCTTAGAGCTTAATTCCTGGCAACTACTGAGATTACTGAAAGCACATTTACCTGGTTTACATTGTGGAGAATCCAGTTGTTACAGTTGTGAAAAAGGTTTGGAAACCAATGTACCTACTAGCGAAGCTAAACAAAGGCTGTGCTATTTATTTTCTAAAGCTATATCTGCTCATAACTCTTGGCAATTTCGTCAGCATTCCGACTTCATGTAAGATTTCATATGGTTTCAACAAGCAGAGAGTTATAAATAACTACCTCTATCATTTGATATTGCTTTCTATTCCTTCTACAGATCTGCCGACATAAACTACTTCCAGGTTGCTGCTGAATCTTATAATGATTTTCTAAGAAATGCGAGCGAGTGGTTGTCTCACAGAGCCACGGATGTAAATTCACTCTCCTTTACTTGTATATTTCTCTGTTGGTGAGGTGGCAGTTAGTAGTTTCTGACTTTTTAAACTAGGGATAGTGCGTTCTATCTTATGATTTTATACTTGTGCAGAAACCTGTAAATGTACTTATGTCTGCAATTATCATGATGCTCGTTTCGTCTGTTCTTCTCATGGGCATTGTATTCTGCTTGTTTAGAAGAGTGCATTTTGCTCAAGTTGGATGTTGTTCTCAGCTGGAGGATCCTTATAAGATTTTGCATTTGGATGAAGTTTTTGTCTTTATTGTAATCTTTCTCCATGTGCTCAGTCTTGGTGCAAGCTCCTTGGTAGAGGAAGAGCAATACACATGGCATTTTCTGACGTCTACCCTGTATCTGATTTTTCTTTCCAGAACAATCGAGTCTCTACTCAAAAGACCAAACTCAGTGACACTACAGAATAAAGATGATAAGACTGTTCTCCCTCATTCTTCACCAACAGATGCACGCAATAATTCTGGCCACAAGTTCACCAAATTCAGGCCATCCAAAATCAATAATCATGGCACTTGTCGGTTATTCTCTGTCGTTGTTGTTCTCATATGTGGAAGACTTTTAAGAGGTTGGCACCAAGGTGGTGTCAATTGGATTTATCTCCCTGATATTTCCAAGTTACTTGTCCATGCTGGAACTTCTACTATAAAAGTTTGTCAGATTTTATCGCTGTTTGCGATTATAATCCTTGGTTCACATGCAATTTTCAAATTGAAATCGACAACAAATTTTGTTTATGGAGTATGGTCGAGCCTACTTCTATCAGCATTTCTGGTCATGCTACATACCATGGAGAATCATATCTATGATTTGGAGCCAATGAATCTCAGCACTACATCAATTGCTCAAGTTTTTTATGTTGTTGCCAGTACTTCAGCAGTTCTAACGTTTTTGGCATCACCATGGATTTTTCCTGTTTATTCTATGGAAAAGCAAATGGTGTACCAAGTTCGCTCTAACTCTTGTTCTACCAAACAACGTGATTCCTTTTTGCTTAGTATTCGTGAAACTACTTATCTGATTGGAACAACATATACTGCTTTCTGGTGTCTTCTACAGCTGCTGATACAACAACCGATAAATGCCATTCCCATTTTATTGATTTACTTGCAGATCCTCTTCAGTATCATCTATTTTTCAGCTGACAGATCATTCCATAGGCAGTGGGTTGAGGTTAGAAATGTCAAACGTTCAAATTATTTCTGCTAATTGTTCCAATTTCCATATTAGAATGAATTCCTTAAAGAGTGTGAAGCACATATTATCTTGATTGGGATATCATGGTATGCCCGTGGAGAACTTTCTTTTATAGGCTTAGTATGTTTGGCCATTTTATACGCTTACTATGTTTTATTATTTCAGCCTGATTGATGGAGGTGGTTTCAAGATCTGTACAATGTTTTGCTACAAAAAGATTTGTGTTAACAATGTTTGAAAAATATGGATAAATTAACAATAAAAAGTAAGGTACATTCAATCTTGACATATTAAGGAGATGTTAGACTTGTTCAACTGAAGGTGTGTGTGGTTGTTGGTAACTAGATTACTGGAAGTGCTCTTCTCGATAAATTGTCTACATTATAAAGTCTATGTAATGCTATTCTAAACGAATATTTTGTTGCTCATAGATCATGAAGTATTGGTTCTTGTAACCATTATACGTTCTAACTACAGGCTCTGAAATTTATAGGTTGCTGCCATATACTTCTTAGGGCTGGCTGGTCATTTTGGTCTTGGGAACACTAATAGCCTTGCTACTATTGATGTTGCGGGAGCATTCATAGTAAACTCGTCTTCTCAAACTTGACTAACTGAAAGTTTTACTCATTTCACATCAAATATATATATATATATATATATATATATATATATATATATATATATTTGCTCTAACTATGTTGCATTGAAATGTAACAGGGTATCTCAAGTCACTCTGCAGTACTTTCTGGCATATTGATGTTTGTTATTACCTATGCGTCACCTTCATTATCCTATCTCAGCATGGTGATGTACATAGATATGAAGGATATTACTTTTATCTCATCTACACACGAGTATGAATGGAGCATTTTTCTAGAAAAGATGATTGCATTGCCTTGCTTGCTCCCGTTGCTATTCAACTCACTTGTGTTGACTTCATTCACCATAATATTGTTGCTCATGAGGAATCATTTGTTCGTTTGGAGTGTATTTTCACCCAAGTAAGTACCACGTAATGTTTGTAAAATATGTTTCTTGCGAAATTGACATGCATGGAGCTCAATCTAGTATGATCGGTTTCTGAACATCAACTTGAGTTATAATGAAACAGTGCTCAAACTGATCTATTATCTAGCTGATGATGAGCTGTTCGTGAACAGCTCATCAGCTAGATTTTATGTGATAAATGATCCGTATTGATTTTATGTGCTCAAAGTCATAAATCATTATGGTGGTTGCTCAGGAAATGCTGTCGTCTTAGATGTCATTACTAAGTTATAGTTCTACATACTATGATATTCCCCTGATTTGGAACGCCTTCATGTGAAGCGTTAACAAAAAAGAAATAGATATGTCAATCCAGATTGGAGAAAATATTTGTTCTTACCTCATGTATTTCGATCCATAATTGATCTATTTATCAAGCTGTCATACATTGGCTAGATATAGTGCTAGAACATAGCTACATCTATAAACATTCATGGATATAACGTAAATTGCTTATTAGATTCAACTTTTGTAATGATGGCTCAACCTGTTTCTGTTATGCTATCTTGAAAGTGATTTTCGAGATAATAATATAGCTGTCTGTTTGCACCACATGTCAGTTCACTTAATGTCTATCATTGCTGTGGCTGCTTGTGTCTGCTGTGCTTTCCTGGCAAGGCTGGTGACAAATATCATGCACATTGAGATATCTAGTCATTAAGTTGGAGAGAGCTTTGAATCTTTTTACTATGCTCCTGTTTTCTTTGACTTCTTTGTTACTCAATTCTTTCTAATGCAATTGGCAACAGGTATCTGTATGTTTGTGCTGCAACAGTGTGCGTGTACATTGGGGTGTTTATTGTAGCTGCAACGGGACTTTATACATACAGTGTTTTTTGCTTTAGAACAAGAAACCCTCAGGAGAAATGCCCGCAAGTGTTACGAGATAACGTGATATCTCATTCTCAGACGAATGTTATTTCTCAGTAACTAAAGGTGCTAAATTAGCTAGAGAGCACTTTTTTGTTGTTGTTGTTGTTGTTGTTGCTGCTGCTGCTGTTGTTGTTGCAGTGTTACCTATTTTTTTGTAGGCACACATCATTAGCGCATTCGAGAATTCAACATATCCTGTTCAAGAAATGTTGGCATTCCTCTGACAACTTTTCCTCAGGTAGTCAAATGATCATTTTGGGTTTTTGTGTGTAGTGGGTTGGCGGTAGAGGGGAATATTCAAGTAGTTGGAGAACTCTGTTAAATGAGATCCAAATTGTAAGGCCATGGGCTGTTTGCTAGAATTAGTATGATGAAGTTGTAATATGTAGAGGATGATCTAAGGTGCTCTGCTGCATTTGGTCTGCTGACGGCGTTATCTTGGTAGGGCATATCTTTTTTAAAAAGAAAAAAAAGGGCAAAGAAAATTCAGAAAACTTTTACTGGCTTAAGACGTTGTATTATCCGTTTGTGCATTGACATGAACCGCTAAACGTAATTAGGTGTTCGGGATGCTCAATTTAGATGCTTTCAGATTCAAATATAAAACTCTTTTACTGTGTGACAAATCGTATCCTCTCTTTTTTTTTTTTTTTTTTTTTTTTTTTTTTTTTTTTTCTCGCACTCTGCGCTCAGAAAATAAGTATCTAAAATTTTGTCATAAAATCTCTAGTTGTTAATTTATGGGGCAGAGTTTTATATGTGATGCTATAAATTCCGATTTTTGGGGTCGGTAAACAGAGTTCGAGCGTGTGCTTCTGAGTTGACATGGTGGGCCAGGTCCATGCATGTCATCTCAGAACAATTCCGATTTTACAGGAGTTAACCCGGCGTGGGTGGTCCACGGTACAATCCCTGGCTCGGTCGGAACAAATCTCGGCGAGCCGAGGAGTAGCGCTCAAGTGCAACGGTTACTCATCGCGACAAGCTGGCGGGTCCCACATAAGCAGCCCCCGTCCGCCTCGGGCAACGCGCTCGAGCGAACAGCTAGCGAGCGAGCGAGCGAGCGATTCAGATTTTCAGCGAGCGAGCGAGAGAGAGAGGGAAGCAGAGAAGGAGGGAGAAGAGAGCTACTACCATCGGTACGTACTCTTTTTCTCCTCCTCCCCCTTCTCCCTCCCTCCCTTTCTTTCTTTCTTTCTTTCTTTCTCTATTGGGTCTCCTCCCCTGTCCCTTTCTACCTCTTCTTCGTTAGTTTGAGATGATGCTGCTGCGTGCGCCATCGTGCTCCGCTGCGTTCGTTGTTCGTGTTTTGGGCGTTCACATTGAAATGTTGTTGCGAAAATGTTGAATCTAATGGTATGATCGGTTGCAATCTAGTTCCCCCCCTATTTTCTTGGCTATTGAGCTGTACAAAACTTACGCAGATTTTTTTTTCTTTTTTTTCCTAAAAGCATATTTTAAGCCATTTGTATCTGTTGATTATTTCTATACGCCTAGGGTCCCCCTTTTTTTTTTTTTTTTTTTTTCCAAGGTTTGTAATATTCCAGAGATATTGTATATTTCATATTGGGAAAGACAAGCTTGCTACTAATAACTTATCAACCCACTTGGTGGGTTGGAACTTGGAAGGTTGTCAGGAATTAATATATTTTTATTAATGGACTACTACTAGTGATTCTCATCATGTATGGAAATAAGAATATTATTTAATCCATGATAATCTCGTATTCATACCACATAAGATTTTACTACTTCACCGACCTATTTTACAACTATCAAACTTGATGAGTGGAGGACAATGATTTGAATTTTTAATAAAAAATATGATGGATTACCGGAATACTACAAACTATGAACCAAACCAAACGGGCCCTTATAATTATTCGATTTATATACAACATAATGAAATTCAAGCTATATTAAATGCAGGTGCCTAGAATCCGGTTGCTCAAAGGTAAAGTTGGAAACTATAATGAGAAGGAGAGAAGTGGGAAACAGTGAATCTCATCCAATTGTTGATCAGGAACACACTAAACCTTTGGATAATCATCAATACAGGCATCGGCGTAGATCAGGGGATATAACATGGATCAAGATCAGTGATCACTCATGGTGGCCATCTCAGGTATATCTAATTTTTTCTGGTCTGTAGATATTGTTCTTTACAAAAAAAAAGTATCTAGGCAATTTCTTTTACCATCAGTTTGATATGTTATACTATCGGTAGTAAACAGTTTGGTTTGCTACCGATTTATTTTTGATGATAGAGTTTTCAAATCGACGATTGACACTATTGAACATGATCTAAATCATTTAAACTATATGAAAATCAAATTTCATGCTTTTTCCAATATTATTCTTTTGTCCTTCAGGTGTACATAAAATGAATGATTGAAAATAAATCTTTTTTAAAAAGTGATGATACAATTTTTGTACTTAAGATCTAGAGTCTAGATCTTTTTTTAAATAGTTAAAGAATTTTCTAACAACAATTCAGCTGATTTGGACTCTTTTATATCGTTAAACGAGCAAACGCACCATACCGGACATTAAAATTATAATTTTTTTTCACCTTTTGATCATTCAGTTAGTGATGCCGAAAAAGTATGAAATTTGATTTTTACATAGTTTAAATAGTATAGATCATATTCAACGGTGTTGATCATTAATTTGGAAGCTCTATCATCGAAACAAATCAGTAGTAAATTGAGCCGTTTACTACTGATAGTAGCCCAGTAAAGTTATATATATGAGATTTACTAGAATACTATCGATAGTAAACGGCTCAAATGAGTAAGCTAATGTTTACCAATTTATAGACAATAGGACAAGAATAGGTTGTTGTAAATAGGAAAACGAACCACAAGGTTCTCTCACAGGTTTTTTGAATAACAGCGATATAGGTCACACAGATGGACTACATTATTATATGAAGAATAAACTTTCAATAATTAATATTGTGGTGCTTGTCAACATCTAACTATGTTGTTGATCATGGTAGCATGCAAATGCGCATTTTTTCCTTGTGTCCCTGTTTGAAAGGTTGTTGATGAAGAAAGTGTGGCCAATAAACCAAAAAAGAAAGCAAAGGATGAAGTACTTGTTCGGATATATGGAACTTGCAAGTAGTGAGTGGCTCTATCAAAGTTTCTATTTTTTTTTTTTTCCTTATTTCTCTTTCTAGTCTGAATAATTGACAATTTTGAGATATTTTGCAGCTTCTACGTGGACCCATTAAAATTCATTTCTGAGTTTGAGAGTGTATGTTCAAATTCTGCAGCTTTCTAAGCATAGGATAACCCTTGATTTTACTGTTGTTGGAAATGATATTCTTACTCATGTTTCTGATCCCTGGAACTCGATCTCTCTAAGTTAATTTATTTTCTGCAGGTGAGAAAGCGAGAAAATATTAGTACCAGAGAATTATTAAAGAAAACTTTGGAGCAGGTATTATATTCTTATACGGGTTACAATTTTTTTTCACTTATTAAGAGAGACATAGTTTAGGATTTAAAGGCAATAAGAGTGATTCGTATAAATTTGAACTGTGTGGCTACAGGATATATCTAAAATGAAATCAGCTGGCAAGTCTAAGAGAAAATTACGTGAACCAAAAGGTTTTTTTCTGCCTGTGATCTGACTTTTTTCTGCATATTATCCGCATGGAAGATGCTCGTTGACTTATTTTTAATGATCTTGCCTCAGAAAACTTGTTAATTCGCTGCAGTAAGTAAATTGTAGTAATGAAATGCTTACATGTGGCATAGCGAGGCATTTGAACTATTGAGGCGGGGTGGATCCTGATATTTATACTGTGCATTTAATATTGTCTATTATCATTATATTGTCTATTATCATTATTTATAATCTAATTTTCTTTCTCTTTGATACTCCAAAGGAATAAATTGATGTTCTTGAGTTAGAATATTTACTGCTGGGACATGTAAACTGGCCTTGTATTGTTCTTTTTTGTAAAAAAAAAAAAAATGTTAATATTAGACTCGAAATTGAAGATTGCATGATTGATTGCGCAGAAACCTAAGTAACCTGCGTTCATCCATTGATTGTTCCTATTGTAAGATGATAAATTTTGTTATTTTCCCACATAGTTTTCTTTACCTATAGATGATTTTGCAGATAATGATGCTGCTAAAGCTTCTAATCACAAGAAGCAGAAGCAAGACTCTAGGGGGGGGAATCAAGAAGAAACTGTATCTGCATCTGTGAGTTTACATATCATATGGATCGTCCCAATTGTTGAATACTGCATAGAGATTCTCTTCAATTTAATATATATATGTTTTCCCTTTCAGCCAGTGATTACTTCAGGTAAATCAGAAGCGAAGAAAGTTTATACAAGGAGTGAAGCTTTAAGACAGAGAGAAGCAAAGTTAGAAGTCACTAGAGAATCAACAAAAATGGAAAATGCTAAACCTGAACCTCGTAAAGAGAACATTATGAATCAATGCACGCGTAGAGAGCAAAATGCTACAAAATTGTCAAGAAAAGCATCCGTGAAAAATATTTCTGGGCAAGATGGTTTAAGAAGATCAAGCCGTATTAATGCCAAGGAACAGACATCTGAGAGAGTTGCAAGTGAAACTTCCCCTCCAGGAGATGCTGGTGGGAGACAAGATAAGATGGGAAAGGCAAATGAAACTAAGAAGGCTCAAAATGGAATGGCGGGACATAACAATCAAAAGCGAGTTGGTTTCGGGCATCCTGAGAGTGAAGTCAGTGAGGAAGAAATAAGAGCTATGGTTAGAGATGTAATTTTTAGAGAGAGAACTTCTAAGCAAAGTGTAAATGGAAAGCCCAATGCAAAAGAGGAGATAGGGAATGGAGCCTCTGAAGTTGGAAATGCAAAGGGAGAAGGCTTTGGAAAGAAGGAGCGCGAACATGCTGAGAAACAAGTGAATATAGTGAGCAGTGCAGAAACATTAACAGGGTTGGTGAGGAAAGAAGGCCCTGAAGTTGGAACCGTGAAGAAAGATAGTTTCAGGAAACAGAAGATTGAGGATGCCGAGGATCGGGTTACCAAAAAAATTGTAGCTAAAACTTCGAGAGAGAAAGAAGTGAAACAATTGGATACTGAAGAGAAAGAGAGCAACAACGCAACAAATTGTACATTGAGAACTAAAATGTTCGATAAAGATAATTCTACAACTCTAAAACCAAACGAAATCAAGGAGCAGGAGACTGCAAAGGGGAGAGATAAAGCTTCAAAACATGAAAGCAGAAGACAAGGTAATGCAGGCATACCAACTGTTACAGAGGATGTAGGTGAAGCTTGCAAGGCTAAAAAAATTGGAAAAGTTGATCCAAACAAATCGAAGCAGTTAGATTTTAATGGACAGGTGGTAGAAAATGGTAGTTGTAGGGCTTCAAAATACAAAGGGAGTGGGGAAAAGGAGACAAGAAAACAGGGAGAGGCTAAATCTGGAGCTTGCCAAGGCAAACCATTCAATCAAAATGATTCAAAGCAGAAGGAGAATAGCAATATCAAGATGCATCAAAGTACAGTGACCACCACACCTGTAAGCATATCTTTCTCTGTTCACTTTACCCTTTTCTTTTCCTATCTTTTCCGTTCGAAATTATCTCTATCTGTTTGGTAATTTACATTAATGCCAGATAAAACATGCGGTTACGGAAGAAAGAGGCCATCTTAGTGCACGGCAGCTTAGGGTAATGCAACGTCTGGGTCTCGTTGCTCCTCCTGGTTCTCCATTTGCGAAAAAATAGATTGGTCACAGCTGCACCAATGGGACATTGATAGGATATATGCACACATATTTAGTCATATTGATCACCTCTTCATAGCATAGACGGCTCTTTTCGTAGACTAACTTCAGCTATTATTTCATTTTACTTTTAGTCTCTGATCCCCTATGAGTATTTTTGATCTAGAGCTGCATTGGATGAAAAGGATGTGGCAACCTTTAATACAAGTAGATTGTGAGTTAGCAGATGCTATTTTACTAAGTCAGATCACTAATATTAAAGACTATTTTTGAACTGTTTCATATACGGCTGGTCTTTTCGGCAATCTGGTATCAGAGCTAAATTTAAGCTTTCTCGGTTCCCAGAGCTAATTTTAGTTTTGTTATCTTCTGTGCCGCTGTGTGGGGTATGATTTTACATGATCTTCGCCGAATATGAGAGCGAAAACACTAGGACTAATTTTGAATCATGATCTTTTGGCTTGTTGGATCACATTAGGTGGGACAGGAGCAAGCCCTAAAAATTTGACCACCTTAAAATGTTGACCGGGGAAAAATTGTCGGTAGCAAAGTGGCATTGCATGAACCTCTGGATCTTCTCTTTACTTTTGCTTTTACTTTTGGCCTGCATGTAGCCTAAAGTGGTTTGCATTTCCTGCTGGTAAATTGCTCTTTGTTTCCGTGCTTTTGAAGCACGACGAAGGCATGCTTAGTAAAAGGGTTGTAAAGAATCTGCCTATTAGGCTATTATTACTAGTAAGCAGTAGCTCTGCTCTTGGCATTTAGTTCTCCAGCACACGAAAGGAATTAAGAGAAATTTACAGCAAATTATGAGTCATTTTGTGCTCCAGTGCATCCAACATAACAACAAGAACAGCATTCGGGATGCTTCTTATTATTATTGTTTTGTGTGTTCAGAAAATAATTTTATGTACTAAAAATTTTGAACTTACTGTATATGATAATGATTGCTATCCGTTTCGTTTATTTCATTTTAGAAATAAACTTAGCCGGAAATGTGAATCAACTAGGATTCGAACTTGGGTCTCGAGTATCAACTACCAAGTTCTTTGCCACTTGATCTAGGAACAGTCGGTTGTATATGATAATGATTAAAAGTAGTAATGGGACAAATAGGGGATAAGATGATCAGTGTGCGGCTCAGCGCGCTAATAAATTACTATGCGATTGAGCGGAGCCAAACTTGATAAAGAATCCACCATGCATTTGCGAGCTTCGACGGGATGCTACTGTGTTGTGACAAAAAAAAAAAAGAAAGTAGAAATGAAATATGAACTAATTAGTAAACATATAGATATATAGATAGAAGAGTAATATTAAGTTTCCAACTCAAAGGTGAATGTAATCCTACTACTTGGTTATGTAAAAAGACCTAAAGTTTTATAAGCTAGTCTTGTCAATGTTAAGTTGGAGGCCTTTTTTTTTCACAGAGAGAGAGAGAGAGAGAGGAATGTCGCTATCCTTTTGTTCATTTTCATTTTTAATCTAAATTTAGCTGAAAATGTGAAATGATTAGATTTTTAAGTTGAAACTTTGAGTATCAACCATCAAATTTTTTAACACTTGTATTACGGATGATCACGGAGCTTAATTTTAAAATGGTAAATTATTTTTTAAGCTGAAATACGAAATAAGTAGATAACCAACGATCACGGGGGATTGTTGGATATTGTTGGATTCTAAAACCTGGATGCGTTGTAGACTTTATAATTTTCCTCCTGCAGATGGAAAAGTATGTCGGAGTCTGATGGGCGCGCGATGCTGTGTGTCCCCCCGAAGGCACCCCAGCTCCGCGGCTTCGTCTCATCTCGTCCTCGGGAACATCCATCGTACCCCTCTCCTTTCTTGTTACCACCACGTCCACGCCACCGTAAAAGTAATTGAACATCTTTTTCTTTTTGTTCTTGTTCCTTTTCTCTTCGATCAGAGGAGAAAAGCCAAGGAAAAAGAAAAAGAGTAGATTGAACGTGAACTTCCCTAGCTACTATATAAACTCCCTACCCAGCCACTTCTCTTTTTAAACTGCATGCGACGCCGAGGACGAGGCAATGCACCGCTTCGACTTATTAATTAATAGCGGATTCGCCTCAGATTTGGGTTTTAATTACTCCCACTTTCTTTTTTTTTTCTTTCTGGACTCTTCCTCATCAGTTGCTTCTCTTATTATTATTATTATTATTATTATTATTATTATTATTATTATTATTATTTTACCTTGTTTTCTGATTCGGTATTAATATTCTTTTTGCATGCGCGAATTATAGTAGCAGAAGAGATGGTAACTCTTGGTGCCAGAGATGCTGCGGAGGTGGAAATGGACGGCGGAGATCCGATCACGGCGAACTCGAGATCGGCCACATCGGATCAACGGCCACCTCGCCACCCTCCGCAGCCTCCTGCCCTCCGCCACCAGGGTGAGCTTAATTTAACCGCTCTCTATTTCTCTCTTTCTCTATATACTTTGCACTGAAAAGAAACACTTTTTGGTGCAACAACGTATATTTATCATCATTATATAGAGCAGTTTCATTAGCTTCTTATATGATCAACTAATAAAAGGTTTGATGCACGTAGATGGACAAGGCGGCGCTCCTGGGTGAGGTGGTGAGGCACGTGCGAGAATTGAGAGAGAAGGTTGATGACGTGGCGGTGGGAGTGGTGGTCCCGGGGGAAGGCGACGAGATAGGAGTAGACCAGGTGGGTGGACCGGGAGACGACGAGGGAAAGAGGGTCCGAGCGTGGGTCTGCTGCGCGGACCGGCCCGGCTTGATGGGGGACCTGAACCGGGCCGTCGGGTCGGTCCGGGCGAAGGCGGTTCGGGCCGAGATGGCGACGGTCGGAGGAAGAACCAGAAGCGTGCTCGAGGTGGAGATGCTCGGGGTCGCGGGCGGGGCCGGTCCGGTCCGGTCGGCTCTGCAAATGGCCCTCCGGTCGGTTCTCCTGAACCGGGATGCTGGCCCGGTCGAGATCTATAAGCGGCCGCGTTTGTCCTCGCGGTTCAGCAAGGCGTAG

>Aco001331; *Ac*bHLH18
ATGGACGTAGACTTCTTTAATTCCTCCCCTGAGGCCCAAATGGAGTTCATGGACATGATGGAGAAAGTGACTTCCCTCTGTGATCAACCCATACGCGACTTCCCTGAGCTCCCCGCAACTCCACTTCCCCACTCTCCCCCACCAGTTAATCTATGCGGTCCCCAAACTGCTCCTCCACCCTTCCGCTGTGGTGCTGCGCAGGAGCAGTTTGCGACCCAGCCCATGTCGTCGGCGGCCGCGATGCGGGAGATGATATTCCGCATCGCGGCGATGCAGCCGATCCACATCGACCCGGAGTCGGTCAAGCCGCCGAAGCGGCGCAACGTGAGGATATCCAAGGACCCGCAGAGCGTGGCCGCGAGGCTGCGGAGGGAGAGGATAAGCGAGCGGATTCGGATACTGCAGCGGCTCGTCCCCGGCGGCACCAAGATGGACACCGCCTCGATGCTCGACGAGGCGATACACTACGTCAAGTTCTTGAAGACCCAGGTGCAGTCCATGGAGCGGGCCGCGGCCGTGGCGGCGCAGAGCGGGGGGACCGCCCGGGCGTTGTCGGCGGCGGCATCTTCGGCACCCGTCGCCTTCTCCGTCGACGGGAGTTGTTACCCTCAGTGGGTGTTGGACCAGGGATTTGTTAGTACTAACCGTTCGTGCTGA

>Aco012193; *Ac*bHLH19
ATGGATGATATGAAGGGAGAATACGACTTGTACTGGGAGACGAAGCGCTTCCTCGAGAGTGAAGAGCTCGAAAGGTTTAATGATCCTTAATCCTTCCCTAATTCCCCTTCTACTTCTATCTGATCATTTTGATACGTTTTGTTTCATCACAAGCTGTATGTAACACTTCAGTGTTGTTCCCTCTCTTTTCAAATTTGAATCTTATTACTGATGCTGCTGCTGCTGTTGTTTGGTGTATTGAAGCATGTGGGGAATAGAGCAGGCCATTTCGGGCTCCTACGCTTCGAGCTCTTCCTCCGTCGATGTCGTCGCGCCCTCCTCGGCCGCCAAGAACATCATGGTGGAGAGGGACCGGCGGAGGAAGCTCAACGAAAGGCTCTACGCCTTGCGAAGTGCGGTCCCGAACATTACAAAGGTACATTCGCAGCTAGTATGGACCTCATAGAATAACTTACTCGGTACATCTCTTTTGTTTAACTGAAAAGGTACTCGAAGTACGAAAAAATATTGAGTATAACAACGCAGAAATTTGTAACTTATCAATTCTGACATCCAAACAAGCCCTTGTACATATTTGTGATCGATAATTTGACTAACGGATGGCCTACCGCTGAGTGGATTAGTTGGACAAGGCATCAACCATGATGGATGCTATCAACTACATTCAGGAGCTTCAGGAGCAAGAGAGAAGGATGCAAGAAGAGATATCCAAACTCGAGTCGGAGAAAACATCGATCCGTGACGTCGTACCGATCATCGAGCGAGACGATCTCCTCGTTCCGCAAAGGAAGAAGAGAACTACGCAGAGCTCATCGTCGCTAGCTCTAGGGTCTTCGGGCTCGTCGTCGCTCGAAGTTACGCAAGTAATCATCTCCCTTTGTCTTTCTAAACTATATGAACATGCATTCATGTGTTAAGAGTGAGAGGGAAAAAGATTTTTGAGTGCATGTATTATTTGTTTATTTATTTTCTCTTCTATTTTTGGTTTTTTTTGGGGGGGGCTTGTGCAGCTTAGGGTTTCTAAAGTTGGTGAGAGGGCTTCAATTGTTGAAATCACATGCAACAAGAGGAGGGGTAGCATGGTGAAGCTGTGTGAGGTTTTCCATTCTCTCAACCTCAAAATAATCACAGCAAACATCACCTGTGTTCCTGGAAGCATCTCACACATCCTTCTTGTTGAGGTATCTCTCTCTCTCTTTCTCTCTCTCTCTCTCTCTCTCTCTCTCTATAACATCAGCATGCTGAGGTAAGTGAGCTGACAATTGAGCTGACCAGTTGACTCAGCTTTCGGCCACATCCAAGTGGCACTTGGATTACCTTACAAATGTGGTAGAACATAATTCCCCTTTTTCCATACAACTATATTCTCAAAAGTGAATATTCTTGTTGATTGGTCCATTTGATTGTCACAGAATCCTTATTATGGACAGGTGAACATACATAATAGAAGTACTAATGGTCAATAAAAACTAAAACATGCCAAACGAATCCTTATTATGTGCCTGTTTGTCACCACTTCTACTTCTACTTTTATGTCACATCCAAAAGTTTGGTGTCTGTGCCGGAATGGATATTAGGAATCGATATTTCGAACAAAATAGTAAAAAAAGACTAAATTAAAACTTCTATTTCTATGGCAGAAAATCTGTCGATCCTATCTTAGTAGAATATATTTCTTCTGAAAGTACTGGTACTGATTAGAATTAGAATGTTACTACCATACCATTTGATGAAGTAACATCTTTTTCTAATCTTTGTTTCAGAGTGAGGGAATGGAAAATGCCCAGATGAGGGAAAAGGTCGCGGCCGCCGTCGCCGAAGTGGATGCTCCAAGAAGTCTTATGAGTAATATTAGTCTGCAGTAG

>Aco016928; *Ac*bHLH20
CTCCCTCCTCCTCCTCCTCCTCCTCCAACTCCTCCGCACAAGCTCCCTCCCTCCACAAACCCTAACCCTAACCCTAACCCTAGAAATTGGAAATCCTGCCCCACGATGCAGCCCTGTAGCAGGGAGATGCAGGCGATCGCGGCGTCCCTGAGCGCGATCGGGATCCAGCCCCTCCTCGGCGGCGTCAGCGGCGGCGACGAGTTCCTCGAGCAGATGCTCGCCGCCGCGCCCTCCGCCGCGTGGTGCGACCACATGGCGGAGGATCCGTCGCCGCCGCCCCCGCCGCCGGTGGCGACGCGCTACACGCCGTACGATCCCATCGGCGGCGCCGCCGCCGCCGCCACCGCCGCCCCGGCGAGCTCCGCCGCCGAGCTCGGCCTCTTCGCCCCAGCGATCGGCGACGGGGGCTGCGAGATCGACCCATCCTCCTCCTTCAAATCACTCAATCCCACCGTACGAGCCTCGAATCCCCAATTTCGACCAAAATTAAACCTTATTCGCATCAATTGTTTAGTTTTTTTTTGATAATTTGGTGCTCAATTTAGGGCGGTGAAGGGGTCTATGCTGGTGGATTCGGTGGGTTTCTTTCCGTTCCAGCCGCACAGGTTTTTTTCTTTTTTTTCTCCCATTAATCCCCCTATTTTTTTCCCTTTTTCTTCAAAAAAAAAAAAAAAAAACACAATATTTGAACTAAAATTTCAATCTAAAATAACAACAGAGAACGTCAATGAGCTTCGGAATCACACCTACGAATGCAGAGGCGACGGTGACAACGACGGCGCCAAAGCAGCGAGTGAGGGCGAGGAGAGGCCAAGCAACGGATCCCCACAGCATCGCTGAGAGAGTAAGCTCTAAAATTCCAGAACAAAATTCCTAAAATTTAAAAATTTTAAGTTTTAGAAATTCTATTATAGTCCCTAAATTTTAAATTTAAAATTTTAATTTAAAATATACGTAATTTGATTTATGCGGAGCAGCCCTTTTTTTTATTTTTTTCAATTTTGTACGAATTTTAATATGATATACTTTTTTAAAAAAATTTAAGAGGTTTTTTTTTTTTCTTTCTTTTGATCTCTTTTGTGTTGCAGCTTCGGAGGGAGAGAATAGCAGAGCGGATGAAAGCGCTCCAAGAATTAGTACCGAATGCGAATAAGGTAAAATAATAATTTCTCCCATAAATAAAAAAAATTAATTTTATACATATCCCTACAAATATAATAAATTATAAATATATTCTTACAAAGTTTAACTTTTATATTTTATTTCTGTAAAAATTATGGTATTTTCAAATATGTCTTCGCCGTTAAGATCAGTTAGAAAAATTAGTTAATTATAGATAAAATACTTAATCCTAATTTGTTAATGAAATGAATTGATCTTTTTACTTCTTTTCGATTAACAATTGAGATTTTTGGACGGACATTTGCGAAAACAAAAATATCATTTTATTTTAAAAATAAATAGTACTTTAACGGTAACAAATCAGAGGGATATATTCAAAAATATTAAAAATTTTTTTTGCAGTGACAAAATATAAAAATTAAACTTTATGAAAATATATTTACAATTTGTAATATTTATAAGGACCTGTATAAAATTAATTTAATAAAAAATAAAATTTGGAGTGCATATTTAGCTCAGAGGAACTCAAGGATGATCAATCTTAAACAAAACAAAAAAGTAAATTTTGTAAACTCTAAAACGATAATTCATGATTAATGCACGAATTTTAAAGCAACAGCTTCAGGAGCAAACTCTTCCGGTTCTATTACTGTTTAATTTTAATTTAATTAAATTGATAACTAATGATGTATTGGTGTTGGAATTATACACAGACGGATAAAGCGTCGATGCTGGACGAGATCATCGACTACGTGAAATTCCTCCAGCTCCAGGTCAAGGTATAAAACCATCCCCACTAAAATGTAATATCCCCCTAATTTAATCTTTACGCTAATTAATTATTATCTACATATATTTTTCACACATTTGATCCAAAGGTAAATTTCAATTAATTCTTTTAAATGACTTAGCATTTTTTTTTTTATTTTGTTGTCATTACCGTTTGAAAAGTCTTTGGTCACGTACCTTTAACATTATTTCATCTTAAAAAAAATAGTTTTTAAATAAAAACAAATTTTAAGTATCATTCGTATGGTTTTGTATTTTTTTATTTTAGTATTTTATGATTTAGAGTGTATCAATTTAGTATTCCATAATTTTTTTTAATTATCCCACTCACTAATATTTTTTTTCATTAAAACAATAAAAAAATAAAAACTGAAGAGTACTAAAATAAATATTTGATAAATCTAAATAAGGTATATGAAATTTTTTATATATAATTTAACGAAATGGTAACGAAGAAACTAACAAAAAAAAAAAAAAATCATAGGATATTAAATTAATATACTTTAAATAACAAAGTACTAAAGTAAAAAAATATAAAATTGCATGAACGGTATTTAAAATTTATTCTTAAATAAAATAGCAAAGTTGAAGTAAAAGGGCGAGTTATAGACTATTAAACTATTATATTTTGAAATTAATTACTTTACTATGCTGGTACCCCATCTATTGCCAATAATAATGTGGTTTTTTCTTTTTTTTTCCTTTTTTTTGGTCGAAATTTTTCCCCAGGTGCTTAGCGTGAGCCGCTTAGGCGGAGCCGCAGCGGCTGCTCCTCTTGTGGGCGATCTCTGCTCCGAGGTATCTTTCATATCTTTTTCTCTCTTCTTATCTTTTATTTGTTATATTTATATATATAATAACTGGAGTAGGTGTTCTAAGTCTTTTTGGACTATGAAGCTCCGGAATGGACGATCGATTTTGTTAAATTTGATTTAGCATATTTAAAATATTTAAAAAATAAATTTTATGATTTTTTAATATCATTTACTTAGCGATCAAAGGAGTTCAAAATCAACGGCTAAAAATAAAAATTTCATAAAAAGTGGTGATATAGAACGAAGATCTCGCTGTGAGTAGTATAAAAGATTTTCTATCAAAAATTTATCTGATTTGAATGCTTCTAAATCGTTGAACTTGCAAACGATATATGTCAATCATTAAAAATTGTTGATTTCGGTCCTTCTCGATCGCTAGGTAAATGGTATCAAAAAATCACAAAATTTACCTTTTAGATGTTTCAAATATGCTAGATCAAATCTAACCGAGCCGACAATTCAAAAGCTCCGTCATCGAAAACGACTTAGAACCACGGAGGCCTCCGTACTCCTAATAAGCACGTAAGACCTACTCATAATAACTGTAATAACATATTAAGAATTATCAACGACCCAAGCGCAAGTGGCAAAAGATTTAGTGATTGATACTTGAGGGTCCCAAGTTCGAATTCTAAGTTCGAATTCTAATTAATTTATATTTTCGATTAAATTTATTTCTAAATAAAATAAATAAAGTAAACGAAAGAAGTAGCATGATAATTATCTCTCAAAATATATATATATATATATATATATATATATATATATATATAATATGTAAAAAAAATAAAAAAGATTATTGGGTTGTTGGGTAATTGTGGGGCCCGATTCGGCTCTACAGGTGGCATAATTATTAGCTATCTGAACATTAGTTATGGATCCGGCTGACAGCGACTTGTCGGGACGGGAAACAACAGGGGGCACGCATGCCCCCATGTGAAATTTCCGGATTACCCCTCACCTTCTAACTAAGTTTTAACATGTTTATAAGCAATTCTATCGAATACACTCTAAAGCGGGTGTATATCTTACATCCATTTCAGACTAAAATTATTTTTATCACATCAAATTTTTAAAATTTTACTAAAAATTAATTCGATTTTTATCAACTCTACACCCTTTTTTGTGGGGTGTACAAGAAGCATTTTTTAATATTTATAATCGCAGACTGTGCCTACCGCACCGTACCGTATCGGTAAAATATCGACACGATACCACTCTTGTGCCAATAATATAGCTCAACATTTTTTTTCTTTAAAATTAATTAGTGATCATTATATGATGGTAGCAAAAAAAAAAAAAAAAAAAAAAAATTTTTTTACGTTTTTATCGTTTACGTATATTTATTTAACCAATGACCCACTCAACCCTAGAAGACCGATCATTAATGATTTGGTTTTTGTAAAAGTATAGAAGCTAATTCCTACATAAATAAATCAGTAGGGCTTATTAATTAAAGCGCAAACTTCAAATTACTACCCTATGAGGGGGGGAAGTTTTTACCGTTTCTCATTTAGTATCTTATTGGGCCTTGCCGGTTGTTTTTGGTTCGAGGTTATAAGTAAGAACTGCTAGTTCTTAGGGATAGGTTACAATTCAGTGATAATCACAGGAATTAGGAACAATATTATGCGTTTGGATGCAAAATTTGAGTAGTTCCTTAGGATATAAGACAAGATAGTGTTTGGCATGTTAGATTGGAACAAGAGGAATAAGTTCATATTTGAAATTAAGATTATATTATGTAATTAATAGTAATAAAAAATATTACTTAAAATAAATAGAAATTAATTATTAATAATTAATTATAATAATAATAATCTATTAAATAATTAATTATCAATTAAAAATTAATAATATGGATGATCTAATTAATAATAATAATTCATTATATTAATTATTAATAAATAATAAAATCATTAATTATTCTTATTAATTATTATAAATAATAATTAATATGAGATATTGATTATCTGAATTATAAAATATTGATTATAATATTTATTATTAAATAATAATTAATCAATTATTATTTAATTATAAATAAATAAAATTAATATTAATATCCATTGTTATTGATTAAGATAACAAATGAATAATTAATTAAAATAATTATTATATTAATTATATAAATAAATAATTAAATATATATATCAATTATCTAATTACTTAGAATAAAATTATTAATAATATTAATAATAATATAATTATTAATTATAAATATGTAAATTATAATAATAAGATTAATATATATATCATTTGATCTAATATTAAGAATACAAAATTATTAATAATAATATAATATAATAATATCATTTAATTAGTAGAATAATATAAATAATAAATTAATATTAATAGTCATTTTCATTAAGAATAAATAATAATTATTATAAAAATACAGTGAATATTATTAATTATTATATAATTATAAATAATAAATTAATAATAATATTATCTAATTTTTAATATTTAATTATATAATAATTATAAATTATTAAAATTATTTAATTATTAAAGTAATAAATAATGATTTAAATATATTAATTAGATTAATTAATAATTTACTGTCATTAAAATTAAATTTAGATTTTAATTAATCTTGTTCTCATCCAGGAACAAGCTTGTTCCAGGAAACAGGTGGAACAGCAGTTCCAGCCTTATACCAGCTAGTTCCAGATTCTCGGGAACTACTGTTCCCGGGAACCAAACGTTGCGTTTGGGAACAAAAGGGGGAACAAGGGCTTATTCCCCCCCTTGTTCCCGAACCAAACACTACCTTAGAGATTTAAAAAGTGTATCAATTTTAATACCTATGATTTTTTTTTTTTCGTCAAATTTTTTTCATTAAACCAATGGTAAAGTTAAAACTAAAAAGTATCTAAGTGAATATTTGGTAAATCTAGATGGGGTGTCTGAAGTTTTTTTGTATATAATTTAACGAAATGAAAAGAGAAAAGTATGGTTACGACAGGATACTAAAGTAAGAAATTAGGAAACGATGGTATTTGAAGTTTATCTTAAATTTAAAAAAAAGAAATTTATTATTATAATTATTATTAAGAAGGCGTTTTTGGACCTCAGGGGAGCGGCGCGGGGGGTGGGGGTGGTAACGAAACCCTAACGGCGGCGACGGAGAAGAAGGTGGCTCAGATGATGGAGGAGGACATGGGGACGGCGATGCAGTACCTGCAGGGGAAGGGCCTCTGCCTCATGCCCATCTCCCTCGCCTCCGCCATCTCCTCCGCCACCTCCTCCCCCTCCGTCGCCGCCGACCAGTAACGCCGTCAGGATCTGACGGCGCCGGAATTCCCGCTCGCGGGAGGGGAATGGTTAACGGCGAGGTGACGGAGATTGACGGCCCCATTATTTGCCCTATAAAATGGGGGACGTCGACGATCCCGTGGCTCATCCCGCTCTTCCTCTCTTACCTTTTTTTCTTCTTTTTTTTTTTATAATTATTATAATTATTATTATTATTATATTATGTATTTTGTTAAATAGTTGTTATGATATGCGCGCTCTCTCCGCGCTTGTAAGTTGCTGTATCATTTTCTTCG

>Aco011934; *Ac*bHLH21
CACAACTGGCATAAACTGAGGCCAAGTTTTAAGAGCTCTCTTCTTTCTTAGAACCTCCTCGCACCCACTCTTCCCACTCCTCTCCCCACCCTCTCCTCGATCGCGCACCAACTCTTTCTCAAACGCACGCAATATACATGCATGGCCCAACACGAAGCGACCTCGGAAGCATCACTCTCCTCCTCCACCTCCACCTCCACCTCCACCACCAACTGGGGCGATGCCCACGGGAACCCGCTCTCTTCGTGGACCTCCATGGGCCAATGGCTGCAGGCCCACTACTCCCCCGCCGCCGCCGCTGCCGCCGCCGCTTCCGACGACGATTACACCAACGTGCACGCAGGCCTCACCATGGACTCGTCTTCATCGTCGTCCGCCAACGCCGACATCTCCGGCGAGACCAACTTGTGGAACCACGTTCTACTGTAAGTATGGCACCGTCGCCGGGAGTTGAACTATCTAGAATATTTCTAAAAATGCCAATCTCTTAATACTACTGTCGCGCGTTGTGTCACATCATGGAGCATTCGAATTTATGACCAATATCGTTAAATATTATCTATTATTTTAATAATTCTAAAATTAACATATTTTATCAATATACATAAAGTTTTTATGGGATCACTTATTTACTAGATAAAAAGCGTTATAACGTAATAAAATTTGCATTACAAAATACTTAACAATATTGATCATCGATTCAGAAATACTAATATCACTGAGAAATGACATAGAAGTATCAAATTAAGGATAAATATTTTTAATAGTATTCTAGATCAATTCCTATAGTATATGTTAGAGATTAGTTGGAGAATAATATGATCTTACTCGATCGTATTATTATATAATATTAATCTGGAATAAAGTTAATGATAAATATACATATGTTTCGTGGGAAAAGCTTGAGATTCTTGTGCTGGGGAGCTCCTTAAAACATTAGTTAATGGAATGCCCAGTTGTTAAATTATGCTATGATATAATGTTAAATTATGCCATACAATTTTTATTCTCTAAAAACTTCAGCTATCCGATAATGGTGCTTTTAATTTATTTATTCAACAAAAACAACACGTAATCACGAAGAACAAATCAACAGTTCAGTTAGAATTAATCTAATGTGAATTTTTCAGGAGCACTGGTTTAAGAGGCCCAAGCACACAACACAATCACGACATTGGAGAAAGCTTCCCCGAAGTGATTCGTCGCAAAAGCTTCGCGCCACCGGAGACCTTCGACCCCGCCTTCGACTACCTAAAGAAGATGGACAACAACTGGGAGTTCGCAAGTGCACCTCCCCTCGCAAACAGCTTCGGAAAACAGCTGAATTGCTACCACCGGAACACGATCGAGCCCGAGAGAATGACCGACTTATCAGATTTGGTCAGCAACTGGTCTATAGCTCCGCCCATCCCGAACATCGATGATCATCACATTAATGCGCCATCGGTGCGCCATTTTTCTTTAGGGCCGAATATTTCACACGTCAAGCATGCAATCCCTAACTCATCTCCTTCGTATACTCGCGTCGACTTGTCGCACTACTATAATGACGTAAAAGGAGAGAGCCATTACCAAGATTTGATCAGCGTCGATAACTCGGTGATAGGACTCGACAACAAGCTTTGCGGTAGTGGAATGATGGAGGAGTATTCGTGCTCGAACGCAAGGAATATATCTGATCTAATATCTTTTAGTGGCAACATAAACGCATCGGAGGTCGAATTCAGAGCTGCAAACTCTTGTTCGAAAAGTTCAGATCCATCAGCTGGTAACTCCACTTCATTGGTAAGTTGTGTTCTTTCTAGTAAATAAATTAGGACGATGTGTTAGTGGTTAAGATTGAATAAGAGCAAGATCATTGTGTTCTTGTAGACGCGGGCAAGTAGTAGAAGCAGTGGAACAAGCGATGCGAAAAAGAAAAGATCTGAAGAGAGTTCAGAAGCGCTTCTTAAGAAGTCGAAACGTGAAAGCTCGACGGCGTCATCTAACAAGGTAGTAATGAATTGAACAGATCTTAATATATATATAGTAATATGTTAGCATGTGATTGATGAGACTTCTCAATCACATTGGTTGGATTCGTAGTGGTGTCTAAGTTTGGTCTAAGAAATTAAGCTCTAATTTGTGAAAACATGCAGTTGCAGATAACTAAAGCAAAGTTGGGAGATAAGATCACTGCGTTACAGCAAATTGTATCACCTTTTGGAAAGGTATATATATAAAATAATTTTCTTTTTTCAATTATAACAAATTTTATTTGCCTTTTAATCGTTAATTTCCTTCTAATTTCTTCTATGATATTCGACAGACGGATACTGCATCAGTACTACTCGAAGCAGTAAACTATATCAGATTTTTGCAAGAACAAGTGCAGGTAACACTTTTTACTAAGAACTTTTAGATCACTTTGATCTGTGATTGTTTCTGGTATTGCTAAAACCGCCTCAAATACGCCGCTTTTCCAACAGTAGTGGTTAGGAGAAAAAAAAAAATCTTAGAAAAGTTTATTATATTCACTTTTGTTGTTTTTGTGGTGCTTAATGCAGTTACTCAGCAACCCATACATGCGATCAAGTTCAAGTAAGGTAGTGCAACTCTACTACTCTTTTGACCTAAAACTAATGGTGCTTTTTTGGTTCTATTATTGGTCTCTAAAATAGAGCAGAAGCGAGATATTTGGCAAACAAAAAAACTAGAACTTCGTGTTGTGCTAAAGCTCCAGCTTTTAGCTAGCTTCAGCTGGAAATTGTTTGGACAATTTTAATAGTAGAGTTAATTTTATATAGTTCTCTATAAATATAGTGAATTGTAAATATATTTCTACAAAGTTTAACTTTCATATGTTGTCTCTATAAAAGTTCTAATATTTTCAAATATGTCTCTACTGTTAGAATCCGTTAGAAAACTTTAGTTAGTTATAAGTTAAATACATAATCTTAGTTAGTTTATGAGATAACTCGATATTTTTGCCACTTTTATATTATACTATTGTCGTTTTTTGAGGGACATATTTGTGATGGTAAAAATGATATTTCACTAAAAATATAAACAGTTCAATAACAGTAACAAAGCAGAGAAACATTTATGAAAATACTAGAACTTTTATAGAGACAACATATGAAAATTGAACTTTATAGAAATGTATTTGCAATTCGTTATATTTATAGGGACTTGTATGCATTTAACCCTTAATAATATATATACACACTAAGATTCTCGAACAACAACTTTAATAGTTTTGCCGTAAAAGATTCAGTTGCACCAACGGCCCACCGTAAAATATGAAAAGTAACTCTTTTTTTCTTGTTTTGAAATTTAAAAAAAAAATAATTTATAGGATCATAATGCATGGGGAGGAGGGTTGGAGAGGAAGGACAAGGTGGATGCACAGCTTGATTTGAGGAGTAGGGGGCTTTGCTTGGTTCCTGTTTCTTGCACTTCTCATGTGTATCGAGATATCACTGGGCCGGATTATTGGAACCAACCATATAGAAGCTGTTTGTATAGATGAATCAAAGCTCTAAGAACCTTGCCTTTTTGACTTAGCTTTATTTATGCTAATAGCTTTTATAGGATTTAATTAAGACATATAAGTGAACATCCATTTTTCTAGTCATTTTACCTAATTTGCATTACTATTGATCCTTTTTACCATTCGCTATAATTTACTTTCTCTTTCTTATTTTATAAGTACCGAGTAATTCAAACTGATCACCTAAATTTATTTTGTTTGTACGTCGTTTTACCTTTCTCTATTGAGTTGTTGGTATATTAATATAGTTGACAAAAAGGATCCTATTAGTTCACACTAGGGATGCAAATGGAGCTGGTCCGGGAGCGAAAGGAGTCCCTACCTTCCGCCCCATTGTTTAGCTTTTGTTCCCAACTTACCTTTACCTTCAGATAGGGACGGATCGGGAGCGCATGTATTGGCAGATCGGGACAGATAAAGAGAAGAAAAGGTCTTTGGGCTCCTGCTCCGCGGATCGAGCGGATTTGAAGCGAGTTAATTTTTTTTTCTGATTTTTTGTCCCTACTTACCGCCCCATTTATGGCGGATCAGAGCAGAAGCTCCGTCAACCCGGATCCTTTTGCATCCTTAGTGTCCATAATACGCCCAATTCTAAGCCCAATACTACAATCAACTGGGCTTTATAGTGTTAGGCCCCAAAAATTGTAAAAACAGAGATATTCGGGATATATTTTGGGTGTGGGCTTAATACGCCATTTGGGCCGGGCCAAATCTTTGTGAGCAACCAACTAAAGCAGTGTATGGCGGGGACATCTCGTCCTTTCCTTACACCTTATGAAAACGATCATCATTAGGGAGAACGACTTAATAAATTAATTTAATTCGTAACGTTAACTAATAAGCGTTCCTTCAAAGCCCCGTCATCGTCTTTGTCCCCTCTCTCTCCTTCTCTCCCTTCCACGCAAACCAAAACCCTAGGAGGGAGAATCGAAACCCATCGACCGATCAATTGATCGAAAGCATAATATCTCTCTCGCTCCTTCAACTCCCATGGCGACGATCGGCAACACCAACCTCAATGCCAAGCTCGTAAGCCTTCTAGTCCCCTCTCTTTCTCTCTCCGATCTCCCCTAGTTTCGATCTCTTGATCTTTTGGTTGATCTCAGGTGTTATTGGGCGACATGGGGGCCGGGAAATCGAGCCTCGTTCTCCGATTCGTCAAAGGCCAATTCCTCGAATTCCAGGTGCTCTTTTGATCCCATCTCTTCCACACTCAGGGTTTTTTGATTTGGGGATTAGGGTTTCGGTGTTGTTTGTGCTCAAATTGGGGGGTGAATCCGCAGGAATCTACGATCGGCGCTGCGTTCTTCTCGCAAACCGTGGCGGTGAGCGACGCCACGGTGAAGTTTGAGATTTGGGACACGGCGGGGCAGGAGAGGTACCACAGCTTGGCTCCTATGTACTACAGGGGAGCTGCTGCGGCTATCATCGTCTACGACATCACACGAACGGTACGATCACGGCAAATACGATTCAATTCCTTGTCACATTGCATGTAGCATTTTACTGCAATTATTAGTTATTTTTTCTGATTTTAGTTTCTCTATGAACTGTTAGGAATCATTGGAGAGAGCTAAGAAGTGGGTGCTGGAGCTGCAGAAGCAAGGTGATCGTCTAGCTTTGCTCATTGGTTTTGTTTCTTTCTGTTGTTTCTTAGGAGTTTGTAGTTGAAGCATGCTAAGTTGGAATTTGCTTCTCCTTTCGTTTCTAAGATAATGTGGATGCTGTGTGTAGGTAATCCAAACATGGTGACAGCTCTTGCAGGAAATAAGTGTGATCTCGAAGAGAAAAGACAGGTTGCAACTGAGGTAAGTATACTAATTTAATGTGTCGCATGTAGCTAATTCCTTCTGTATGTTTAGAACATACACACGGTTTCTTATGATATCTGACATTATGAGTATCCATATCCATATTGAGAATGTTTGGCACTCTATGTTGTGTAGCAAAACTGTGATAGTTTGTCCGATTGTAATGCATTGTTATCAAGTTCATGTATCATTTTGTTGAATTACATATGCAACTGCTTTGGTAATATGAGAGCATGGTTCTGGAATGAGGTTAACTCCATGTATAATATATTACCCTGATCATGCTAACTGTCCCATCATCATTTTCATGAAATATGCGTGTATTATACATATGATTATGTGTTTATGGTGTATATTTGAGGTCGTAACCAACATGTTAAACATGCTCTAATGTGTTTACCATGGTAATAGGTCAGACTCTAGTTAAAGTTCAAACCAATTTAGGATCTAAATTGCAACCTATGAGCTAGTAGGTGACTAGGTTCGAGTGCCTACATGCATTTGCTTGTGTGTCAGTATGCACTATCTGTGATCCTAAATGCAAATCTGAACCTGCTTAAAAGAACCGTTGAGTATAGTTTAAAGGACTCAGACCTGATAAACTGGTAGGATTTGAAATTTGAGTTGGTTAACAAGTCCCCTCCACCTCTAATTAATTTCGTGTGATACATTATTCAGTTCCTTGTAATTTATGATGTCTGTTGTTGCAGGAGGCGCGCGCTTATGCTGAGGAGAATGGTCTTCTCTTCTTAGAAACCTCTGCCAAGACAGCTATAAATGTGAATGATATATTTCATGAGATAGGTATGCAAGCCAATTTCATTTGTTGATAATCAGGATAGCTTTTGTTGCTTGTGTACTTTGTCATCCTTTATGCTATAATTGATTGGATACCTTAACTTTTGTTTCTTCCTTCTTGCGTCTATGAGAGTAACTTCAGATTTTCTGCATATTTTATTATGTCAAATATTAATTGATAATAGCTCTTCTATTCAACCATATGCAGCAAAAAGATTGCCCCGTGCTCAGCCAGCTCAGCAGCCAGCGGGGATGGTTCTTGCTGATAGACCAGCAGAGCAATCACGGGCTTCTGCATGCTGTTCTTAGGTGCTGTTTTTTTGAAAATAAAGCAGAGAGACACGTGCCTGTGGTCTCAACATTTAAGGCTGTTTGATAGGCGGCAACCGGCATATTCAACTACATCGCAGGTTCTAACTGCAAGAGGGAAATCTTGTTCCTGTAGTTGTATCTGGAAACTGCAGTTGGATATGCTGATGATTTTCGGGCTGTTGCCGTAATTTATATATCTTTCCTTGTAAGAACTGCATTCATTGAACGCCCCTTGATTTGGACAGGTTCTCGGAAAAGCCAGAGGAACTCTCGACGTTATGAGGTGAATGACGATAACAAAGCAATGTGATGAACCAAGAAGATGAAAATATAAGCACTTGTAAATTTGGCCCACCTCCTTTATAAATTTCTGTTAGTTGTGTTTCTTCATTCATCAGTTAATATCGAAGTAGCATTATTATCCGGCAATTATCGAGTTCTTGTCATCGTGTGTAGTATGTGTTTTAACATTATTAGCGCTCTTGTCTCTTGTGACTCTGCACTCAGTAAAGAACTGTACAGATGTAAATCATATTTGAGTTGGAGATTATGTTTCTCTTAGAATTAATGATGTTCAGATTTTTTTTCCTCTCTTCAAACCACTCAAGAGCCAAATAAGACTAGTACCGTAG

>Aco011985; *Ac*bHLH22
CTGAACTGCTTCGGCGGACCGCAGTTTGCGCCGGAGCCCAACTTCGATTGCCTCAGCGAAGTCTGCAGCCCGTTTGCTGCCGCCGGCCTGTGCGGCTCGTCGGCACCGCCGGCGGCGGCGGCGGCGGCGGCGGCGGCGGCGCATGCTGCCATCCACGAGGAGATGAGCTGCGGCGGCGCCTCCGGAGGCGGTCGGAATATCTGCAAGGCCGAGCAGAAGCAGTCGGCCGGCACGCCGGAGCCGCTGATGCTCGCCGCCAGCTCTTGCCTCGAGAGAAAGAACGCCAGGCCGAAGAAGGCGGACGGCCTGCCTTCCAAGAACCTGATGGCGGAGAGGCGGCGGAGAAAGCGGCTCAACGATCGGCTCTCCATGCTCCGATCCGTCGTGCCCAAGATCAGCAAGGTAATGTTAATATGACTAAAAGTGCCTGTTCGGACGTGGCCTTATATATATATATATATATATGCTCACATACTCGTCCGGCCTATCAATTTGTAGATGGATAGAACGTCCATCCTCGGAGACACAATCGATTACATCAAAGAGCTCATGGCACGGATCAAAGCCCTGCAAGAGGAGGCCGAGGCCGAGTCCGATCCGCGGAACCGGCCGAATCAGTTAGGTGCCTTGAAGGATCAGCTGAATCCGAGTGAGACGACGCTCATGAGGAGCTCCACCAAGGTAAATAAATTAATCTGCCGGCCGTGTTGCTCTCGATCGATCGTCAGTTTAGTAAATCCTTTTGTTCCTGTAACGATCGAGTGAAGGCCGTCGCATGCAGTTCGATGTCGAGAGGAGAGAGGGGGACACCCGCGTAGAGGTTTGCTGCGCGGCGAAGCCCGGGTTGTTGCTTTCGATGGTGAGCACGCTGGATGCTCTCGGTTTGGAGATCCATCAGTGTGTTGCGAGCTGCTTCAGTGATTTTGGGATGCAAGCTTCTTGCTTGGAAGTAAGATTAGCTGCAACATCGATCTCTCTATTTCAGGAGCTGTTTGTTTATTTATTTCTTTTGTTTTTGTTAATGCTTATGGTGGTTATTAGGACAAGGAGCAAACAGAGCTCATCAGCTCTGAAGAGATAAAGCAAGCTCTCTTCAGGAATGCAGGTTACGGCGGGAGGTCTCTTTAGTTAGGAGTTCGGAGCTAGAAGAAGAGAAGCCGCAGTGGGGGAGAACTTGAAGAAGCTCTTGTTTTGGGTGCGAGAATTGAGAGATGGGCTTGAATTAATGACTAGAGCTGACTCATTTGTTGTAAGCATCGTGCTAAACAACGGATGCTTAATTTGTTCCTTTTTCGCGGATTGTAGTATCTTTTGGTACCGTTTGCATTGCTCTTCCTGCAACGGAATTTACAAATTAGAAGTGTTTTTTCG

>Aco012028; *Ac*bHLH23
TTGTTTTTTTAATAATCTTTCTTGTAGTGTTCCAAAACCATATATGCGCCTTTTTTTGTTTGAGTTGAGTGATGATAGGTTTTTGCGCTCTACCATTGAGGAAAGAGAATACTAATAATTGAAGATTCTTGGGTTAGTTTGCGCCTAGCTTCTATGAGTATATTATATTTATATTCAACCTCTCTCTTTGCTCTCCCTTATTTACTAGAAAATTGTGTGTCTCCGGGCCTTTGTTTCCTCTTCTTGCTGCTGCAAAACTAAGAGACAGAGACACTTTAAATGGTGATCTATGGTGCCGAGTGTTTTGGTAAACTAAGCACAGTTGCTTCAGCATTGCTTCGTTCTGTTTGGCTTCAGAAACCCATAGAATACTTCAGCTCCTTCTAATGGTAATTATGCTTCCAATCTCCATCCACATTTGAAGCTCTTCTTTATTTGCAGAGAAATTTGCAGTTGGTTGAGCTGAGCTATTCCCATTTCGGAGAAAGATACAGTACAAAAATTAATTGTGTGCTAAATTTTTAAATTAGGTGGAATAGCGGGGATAACTTTAGTCGTATTTGTTATACTGAAAGAAGAAGTGTGATATTGTGATTTGGAACTTTCAATTTCTGTATTTTGCTTCTTTTTGTATCTTTAAGAACCTGCTACTGCTTTCTCCTCTTACAGCAGGTTATTTAGTTTTCTAATTGCCTATAACAGAGATCTTGGGCTAATTTCTATTGGGTAGGAGTGGCCATGACTAAATTAACAATGCAAGAGAAGAGGTTCAGCTCTGTTCAATTTTCTGAGCTGAAAGAAGAAGCACGGCAGGAAAGACGAAAGGCTGTCATCTGAATGCTTCTGCTTATGCTTCTAAGCTGAAGGTTTTCGCTGATTATGTGTCGAGAAATGGCGGCAGCTCCTCCGACCAAGGACGAATCGCCGGAGGAGCACTTCAGGAAGCAGCTTGCTGCTACTGTGAGACAGATCCAGTGGAGCTATGCGATCTTTTGGTCGATCTCGACCGGGCAACAAGGGTTTGGAAAATCCATACATGATTTTCTTTCTAACATCTTCTTTGTAGTGTATTGATTGGCCCAACTTTCTGATTTTATGTGTCAATTGTTACTGCATTGATTGCTTTAGAAGTTAATTGAGCTTCCAATCTTTTTTGTTGAGTGCATTACAATATCTTTGTCTTTTAAAGAAGAATTTAAGAGGTGGGTCCTGAGCAAATACTGTTGAGAGCACATGTTTTATGGAGCTGTGGTCCTAGTCACATGAGAATCTGCCTTTATTTTTAGTTTAATGCAAGTTCAAGTAGGTGTTGCCTAACATTCAGCTTCTGTTTAGAATATTGATAAAGGTTATGTTAGCCTTGGCACATCTAATTAATGTCCTTTTTTTTGCCCCTCCTTTTTGAGAGTAATAATTGTTTGGATAGTGCATATTGAAGCTATTATTTTTTTTTTTTTATTTTTCTGCATGTTCTTGTGAAATTGAGCACACAATTTCCGTTAAAACAATCTGCAAGCCAACCTTGTTGTTGCATGAAATATTTTTCCTTTTCAATTAGTTCCTCTGTTTTCGGTATTAAGAAACAAACATATCTCATCCGACAGCCCCAATTAAGCAACAAAGTATCTGAGTTGTTGTTATTCCCTTCTTCATGTGTTTTAAAGTAAATAGATAATACAAGATTTTCGGTAATGCATGCAGCAGTTAGCATCATTTCAGATTCCTCGGTGTAGCTTCTTCTTAATGGTTCTGAAACTTCTCTGATGTACTATTCCTTAAGCTTGATTATCTAAACCTTGCACTATGTATTTCATAACCTCTTTTTGCATTCAATTGGACGGTCTGTAGGGTTTTATCTTGGATGGATGGATACTATAATGGTGATATTAAGACGAGGAAGACTACGCAGCCGATGGAGTTTAAAGCTGATCTGCTGGGTCTGGAGAGAAGCGAACAACTGAGAGAGCTATATGATTCTCTGTCGGCAGGTGACAGCAATCAACAGAACAAAAGGCCTTCTGCTTCATTATCTCCCGAAGATCTAACAGATACCGAGTGGTATTACTTGGTTTGCATGTCCTTCACATTTGGGCAGGCCCAGGGGTAACTCCTCATTCATACTAACCTGCTGGTTTGGTTGTTATGCTTTTAATTCCTCATTTGGTATCAGGTTAAATCCATCTTACTGTACTGGAAACTA

>Aco012816; *Ac*bHLH24
ATGAGTCAGTGTGTGCCGAGGTGGGAGTTGGACGTCGACCCTCCTCCTCCTCCTCCTCCTCTGCTGAACTCGCCGCCAACAACAACACCACCCCAGCAGCTCCCTCCGCTCGTCCCCACGGCGGGCACGTCGGAGCGCTACGAGGTGGCCGAGATCACGTGGGAGAACGGCCACCTCTCCTTGCAGGGCCACGGCCTCCCCCGCGTCGCCAAGCCCCTCCCCAAATTCCCCACCTCCGCCGCCTCGCCGTGGAACAAGCACCACCACCACGGCGGCGGCGGCGGCCACGGCACTTTGGAGGCGGTCGTGGACCAGGCCACGCGCCCCCAGCTCCAGGAGGTGGGCCCCAGCGTCGACCTGCTCGCTTGGATCAGCGGGTCCCACCCCTCGCCGTCGGCGGCGGCGGCGGCGGACTTGCTCGTGCCGTGCGGGGAGGCGGTGGCGGGGGGCCGGAAGCGGGGGAGGGGGAGTGCTGCTACGGCGAGCCAGGGGAGCGCGGCGCCGGGGCGCGGGGAGAGCGCGTTGGCGGCGTTCGACACGAGCAGCGGGGGCGCGGGGGGCGACGAGGTGTGCTTCACCCCCACCACCACCAGCCCCACCACCACCACCACCAACACCAACAACAACACCTCGGCCTCGCTGGGCTCCCCCGGGACGGAGAACACCAGCTTCGGCCGCGGCGGAGGATGCTACGACTCCGTTTGCCACAGCCGACGACCGCCCCAAGTAAAACCATACATGCATGCTTTCCATTACAATTCTTGTTATTGTTTTTCTCAAAAAAAAAAAAAAAAAAAAACAAATGGAGCTTAATTATGTACGTTATTATATATATAAACAAATAGAAAGATGGCGTGTGCAACGAGGAGGAGAAGGCGGCGAAGAGGGAGGCCGGGAGGTCTTCTAGCTCCGCCAAGAAGAGCAGAGCGGCCGCCGTCCACAACCAATCTGAACGTGTAAGAAACAATTTTACTGGCATGAAATAAATTTTTTTTCGCTCTCTCGGTTAAAAAGTTGACAGCAATCTATAAGTACAGGTCATCTGAAGACGACGACCTGTGCTTATGGGTTGCTGTCAACTTACTTACTGATCTTTCAAATAATAATCCCATGTGATTTCAGTGTTATTTCATTGTCTCGTAAAATTATCGCTTGCGCTCAGGTGTGTATCTATTTTGTCGTCGGTTCTTAGCCTGTGAATTTTAGTACTTTACTACAGTTTTTAAGAACTGGATGATTCTGGCGCGATGCTCGGATGTGCCCGTGCATCTCCAATTGAAGGCAATAATTCTTATGATATAAATCAAGCGTATTGAAAATTTATTACTAAAATGAAAGTTTTCTAAGATTAGATAACAAATTCCAATTGATCCAAAATGAAGTCGAGTTCCTATACAGGATTTTTTTTTTACACCCCCAGTTTTCTTTTACATCACGGGTTTAATTAGTTATTAAACGCGATTTGATTGAGAAAGCAACTTTCATAGTTTAACGTGGGTTAGAAATTTGGTTGCTGAACCAGTGACAAGGCTGCGAGGAAAAAGTTTATATTTGTGGCCCCGCCGCATAACATGCTTATTGTCCCTTGTTACTGGTTCAGCAACTTTCATTTTAAGCATTTTAAGATTACTATCACTATTGGCGTAGCAAAATATTGTTCGTATGATCTATGTTTGTTCATCGTCATAATATGAAGAGCTCTGTTTGATTTATGTACCGTCAAGATATTATATAAATTTGTGTATATCAACGATTTACGGTGAGAAAGAGGTATGCATAATGTCTCGATCGATATACTCAAACAACAATTGAAGCCACTTTTCTTTGCAGTTTTGGTGTATACTAAAAACCTTAGCAAAAGAGCATTCCTTAAGCTTCAAACTAGTCTCGCTCCTGGAAATTAAGTACCATGTAAGATTTGGGCTCACACGGTTTGTTCACTTTTGTTTGTTGACTAGTGAGTTTTGTGCCTTCATGCGAAGAGTCAACACGGCAACTCACTGCGATCGGTTCGACCGTGACCTATACAGAACTTAATAATTATGGTACAGAACTTTTTAAATGTGGTTTAGTTAAATATATTGTGCTTGGATAATAGCTTACCAAGGTTTAACTGTTCTTTGTTGTACTCTACCGAGATTCTGACAAGCTATACACATCTTATCCTTTTAGTATATAATGCGCTTGTATAAAATTGATTTGTTGAGTTATTCAAAATTTTAAGGGTCGTACGTCAGTTAAAATTAGACGAAAAGAATAATGTATTTTAGCTATAACTACATGTATTTGCTTGCAGAAAAGAAGGGACAGAATCAACCAGAGGATGAACACCTTGCAGAAACTAGTCCCCAACTCAAGCAAGGTAATCATTAATAGAAATTAATTATTTCTTGTAATTTATGATTTATATTACCGAATAAAGAAAGCGAGCAGCGATCAAACATTATTATTTTTTTGGGTAATGAAAAAGACGGATAAAGCATCGATGCTTGACGAGGTGATCGAGTACGTGAAGCAGCTACAAGCTCAGGCGCAGATGATGAACCGGATGAGCGGCATGCTGATGCCGATGACGATGCCGTTGCCGCTGCAGATGTCTATGATTGGTAACATGGCCAACATGGCCCAAATGGCCCAGATGGGGATGGGCATGGGCATGATGGATCTTAGCTCGCTCGGTAGGACCGGGCCGATGGGCTTACCTTCTCTGCCTCCCCTTCTTCATCCTTCGCCCTTCCTCCCGATTATGTCCCCTTCCTGGGACGGCTCCGGCGATCGGTTGCAGCAGCCCAGCGGCCCCATCGTGTCGCCTGATCCCTTCTCGGCATTCCTTGCATGCCAAACTGCGCAGGTACACTCGGCGGTCGGACATATCTTACATAAACTCTGGTGCATTTTGAAATGCGTAGCTAAACTAAAAAGAAGTTCAAATTAATTTTAGAATCAGCTGATATTGTGGTGGATTTGATATACTTGGCTTTCATCTCCATGACATTGGTAATCTTTTTGACGGAATAATATATATGTAACCTACTTAACTTTAATAAATATTTCTGAATTTAAAAGAAATAAAAGATTGGGTAATAAAGACCATTGTCAATATTTAGGTGAAGTTTTCACATTTTGCATACAATTTATCAGTTAATCTAATGCGTAGTTTCTATTGAAATAAAATAGTAAAATACTATTTTTTTCGATAACTTTAGTTTTTATAGAATAATAATTGTTTTATATACCTTAATATAAGCAAATCTTCACTATATGCTATTTCACTTTGTCCTTAAAAACAATTTTTGCTTCATCTTTTTTTAATATTTTTTATTCCTTTTATCTCTTTGCAGCCAATGAGCATGGATGCATACAACAGGATGGCCGCTTTATATCAACAACTATACCAACAGAACACACAATCCAATTCCAAGCCATAG

>Aco012937; *Ac*bHLH25
ATGACGCTCGAAGCGTTGCCATCAAACGAGCTCCCGGCGTTTCTCATCTACGACACAATAAACGCGGCCGCCGCTGCCGCCGCCGCCTCGAGCTCGCGCTTACTCTTCAATGGCAACGGCGGTATCACAGAGGAGAGTCCGTTGATAGGTGGCATGGCTGCGATGGCGGAGGAGGTGGCAGTGGCGGCGGGGGAGGGGAGGAGGAAGCGGCGGCGGCGTGCGAGGAGCGGGAAGAGCAAGGATGACGCGGAGAGCCAGCGGATGACGCACATTGCCGTCGAGCGCAACCGCCGCCGCCTCATGAACGAGCACCTCGCCGTCCTCCGCTCGCTCACACCAGACTCCTACGTTCAGAAAGTAAGTGTCTGTTTGACTCCACTTTTATCAGTACGGTGCACGTCGGATGTTGAGGGGTGGTCAATAATTAATGTATCTCCAACCACATCTAGTTATATTTCACTAAATTTGGTGTTGTTCCATTTGTAGGGTGACCAAGCTTCAATTGTGGGCGGAGCAATTGACTTTGTGAAGGAGCTGGAGCAACTCCTCCAATCTCTAGAAGCCCAAAAGCGAACACTACTGCAGCAGCAGCAGCAGCAGCAACAAAAGGGCAACAGTGACATTTTCCCCACAGCTTGTTATGAGAATGACTCCCCTCCCTTCGCCCAGTTCTTCTCCTACCCTCAATACGCATGGTGCCATCCTGCTCGCGATTACCCCTCGCTAGAGATCCAGCACCGGCCCGCGACGGTCGCACCGTCGGTGGCCGATATCGAGGTGAGCCTAATCGAGACGCACGCGAATATCCGCATACTAACCGCGAGGCAGCCCGGGCAACTCCTCAAGATGGTGGCCGGAATTCAAAGCCTCAGGCTCACCATCCTGCACCTCAATGTGACCGTTCTAGACGCCATTGTGCTCTACTCTCTGAGTGTCAAGGTATGCATCATATATATCTTATATATGCATATCTATATTTTGATTATTGAACTTTTAATCTGCGCGGCAACTAGATAGAGAACAAAATGTAGATTAAAAAAAAAACCTAATTGATATTTTTTTTTGAGAGATAAGTAGCACGCTATCCGCTTCATTTATTTTAGTTAAAAATAAATATAATTGAAAATGTGAATCAATTAGGATTTGATCTTGGAACCTCGGACACTAACCATCAAGTCCTTTTGCCACTTGCGCTAAATATGGTCGGTCAAAAAAAAAAAAACTTATTGATTATTGAACTCATAATCTGTGCGGAATGTGTTGTTGTGTGATTTTTAATTCGAAAGCGGAATCAAAGTTAGCCTGATAGTTGTGGAGTGTCAGATATTCTCTTGCAGCAATCTTATGTCTTTAAGTAGGCTTTAAGTAGGCAAAGACTACGGCTGAAAATGTTCTTAAGTAACCGATCGTTTCTAGAGCAAGTGGCAAATTAAGAGCTTGGTGGTTGATATTTGAGACCCAAGTTTGAATTCTAAATGATTCACATTTTCAGCTAAGTTTATTTTTAAATGAAATAAACGAAGCGGATAGCGTGCTAACTATCTCTCTCTAAAAAAAAATAATAATAAAAGAAGAAGAAAATGTTCTTGAGTACCAACAAAAGTAGATCTGGCATGTACTACATACTATTCCAAAACACTCTTATTGGTGTTTGATGCAGGCTTTATGAGTGTCCTAGGTTTTGTTGTTTTGCACCAGCAGGGGGACCCCCGAAAAAAAAGAAAAGAAAAAAAAAGTGACCTTTGGTGGTGACCTTTGGTGGTGCTGTCAACTTCCACATATCTTATTAATCCATAGTCAGCAGAGTGATCTTAAAAGATACTACATAGATAAATTTTAAATTTTCATTTTAGAGCTTTAGCAGTCACAGGCTCACAGCTGATATATTTCATGTGTCTGCTGGGTTGCTGAGAGAGAATCTCTGGGGAGAGAGAGAGAGAGAGAGAGAGAGAGAGAGAGAGAGAACAATTGCGATGACACCCTTCCCTGTTGCGTTTCACAGAGCAGTGGCTTTTGCTCTGCTACAGGGTGGGTCCATTTAACAATAGATGCTGTGCTGCTTTTGACCAGAAAAGGTTGATTGAGATGCTATTCTATATTTGGGAGATATTCATCCTTGAGTACGCTATTGTTGATTGGCATCGGTTTCCTTTGATATTACAGGTCTGAATTTCTAGGAATTACTTTATAGCAATGAAAATTTTTAGAAGGCAAATGCTTGTTGTACAGTAATATATATATATATATATATATAGAGAGAGAGAGAGAGAGAGAGAGAGAGAGAGAGAGTATCTAATTGCCTTTTCTCTCTACAGGTTTCATTTATTAAAATTATGAACTGAGCTACATGGGTGAGGTAATAGACCTTAGTCAGGACGAAAAAAACAAAAAAACAAAAAAAACAAAAAACAANGTACAGTAATATATATATATATATATATATAGAGAGAGAGAGAGAGAGAGATGGTTGAAGTATTTTTTTTAGAAAAAGGTAGTTTGCTACCCGCTTCATTTATTAAAATTATGAACTGAGCTACATGGGTGAGGTAATAGACCTTAGTCAGGACGAAAAAAACAAAAAAACAAAAAAAACAAAAAACAAAAAACAAAAAACAAAAACAAAACTAAACAAAACTAAGCGGCTACTTTGCCGAGGTGAATTTCTCCCACAAGTCCTTCAAGTACCTGATCTTATTGACCGCTAGAAAAGGACGAGCAGGCTCATTTCGTTTATCTTTCTAAATATCATATCGTTTCTGACCTTCTAAATAATCCACCAACAGGCTATCAGTCCGATCAGCTTAGGACGAGCAGGCTCATTTCTAAATCTTGAAATCCACTTATCCCAGATGTGGGACACTTCGTCACCCAAGTTTATCAATTGAATCCCCTCCAAGACTATTACTATTAGGAATTTACTAAATACGCATTGCGTGAATAGATGATCCACAGTTTTCTCTTCCACCCCACATAGTACACAGGCTATAGACTTCGTCCATCCTCGTTTAAGTAGGTTGTCCGCTGTAAGATGTCTCTTCTTAATGACAAGCCAGGTAAAGATTTTAACTTTCAACGATATTCTGAGATTCCAGATTTTACTTGCATGTGCATCCCTTGTACCCCCATCACTAAGGGTGGCATATGTCGACTTAACGGTGAAAGTTCCATCAGAACTCCACCGCCAAACCACTTCGTCCGGTCTCTGTTCTGTCTTGAACTTTGAAATTCTATCCTTGAGCTGCAGTATTATTAATACTATTTGAGCTTTTTTATTATTCAGTTTTTGACCGTTAAATTTATTTTTTTGCTAATTTTTATCATTAAATCAATATTTTTTTTTACTAACTACTACCAAATTTTGGGCGACCATTATCATTCTAATAATTTAATTTTTTATCGAAAAGTTAAAAACTGAATAGTAAAAAAGCACAACTACTATTAATAATATTTCAGCTCATCTCTCTCTCTCTCTCTCTCTCTCTCTCTCTCTCTCTCTCTATATATATATATAATATATCTCTTAAATTTCTTATATTTTCTAATATTTCTAAAACTTTTAAATGCTTGGTAAGAAATATTAACAAGAAAAACAAGTCCTACATGGAGTGTAAGATGTCCTATGTCGAGGTGTACAAATAAAATTACTCATTCTAGAATACTTTAACACCACCATCCAATCAAATATTTTTCTTTCTATTTCTACTTATTATTATTATTATTATTATTTTGTAACTTATTTTTACAACACAATTGTTTTTAGAATACAAAGTAATTTACTATCCATTGACAACAAGTTGGGGTTTAATTCCTAATTCTACTCAGCACGAGCAATTAATGTTTTTTGGTAGCCATTACTTTGTAAAAAGCATAATAATTAACCACTGGACTAGATGTTCAGTTCTTACAGTGAAACAACTGTTCAAAGAATCTTTTGTTGGTGACCACTTGCTATCATCACTCAAATGTCTTAGACCCAGACCAACCCTTTCAGTTTTGCTTATTTACAATTGTCAATAAAATACTTAACTACATCTTTCTGGCTACAGAATCTGTGTTACCGATATAAAATTCTAGGCAGCTGCTGGACTCCAATTTTCTCTGAAACAACAGTCTCCTCTGACCCGATTGGACCCTCCAGACCCTGTAAAGATTCTTCTGTTTGTCCAGTTTTCTGCGCCCTCAAAATTTTGTTTTCATTTTACTTTTTGTGGACCCTCATGTCGCGGACAATTTTAACATGTTAGCTAGATTATAATGATGATCAGATTCAATATATCTAATTTATTATACAACATGGTGATTGCAGGTAGAAGAAGGGTGCGATGTGGCTTCAGTGGACGACATTGCGGCGGCGGTTCACCACATCCTCTCTCTGATTAAAGCAGGGACGAATCTCGTGGACCGGTAGAACGCAACCGAGTTAGTAACATCTTATTAAGCTTGTTTAAG

>Aco017264; *Ac*bHLH26
ATGGCTAGGTGTGTGAAGAATTTGGATTCAGGTAGGTTTCTTAGAGGATTGAACTCCCAAAATTTGTACATATTGGTCGCATTTAAGATATTGGTTGCATTTAAGGTTAATTAACTACATCTGAGTCTAAAACATGCCTCCAAGTTCGATTTGTGGAGTAAATGATGTTTTTGAATTCTTAAGCTAGAATGACTTCGCATACTCTCCAAAACTGCACTTGTAACTAAAAAGGCTTCAGTTATGTTTAGTCTGCTCGAAGCTTCTGGAGAGGTGTTTTTGAGTAAAGTTGTTTGAGAAAACATTATTAATTTTTATTTTTACTCATGTTTAATTATAATTTTTTCGACAATTTCTGACAACAAGAGAAGTGAAGAAAATCCAAACAAATACTTCCGTTAGTAAGGTTTGTAAGCTCAATTAATTTCCTCCGCTGCAACAAAAGTTTAGATTTGTTTTTTAAGAGATAGGTAGCATGCCAACCGCTTGAACGTTTGTTGTTTGAATGTTTTGTCTATTTGATTCTTGAAATAGAGAAAATTTTGCAGACTAAGGCTAAACAGACATTTAAGCTATCAAGTAACAAAACATGGCACGTATGTGATAGCAATCATCTCGACTAACTAAATTCTTTAGGAATCCTTGTACGCCATTATCGCAAGGAGATCCCATAAATGACTTCTCAACTCCTTGCCAATTTGCCATCATGATTATTGTAAAATAATTTCACTCCGAAAAAAATGATTATTGTACAATATTAGCGCACTCTTATGTGGTACATTTACTTTTGGGACGTAGTGTATAAGTAAATACACCATATGAATATAGTAAAGGGGGAAGTTAGGAGGCATGGCTTGCATTTTTCAACTTCCTAAGGGATAAATAGAATCAGAGAGGCCAAAGCAAAAAGAGTGTGGATTTGGAGTCTCTTCTCCCCATGTTTGGTGGCAAACATGTGATATTCCCTCACCCAAACCGTGTTTAAATGGTAAAATGAATGAATATAGAATTCATATTAACATAAATTAGTCCAATTGATTAGAAATCCTATGAATTAGTTAATTGGTTAGACTATAGTTCTTATTTCTTGGCATAATTTACCATCAATCAAGCTAACAAGATTAGTTGCAATTGCCGTTTTTCATGCGTAGATCATCATTCATGGTCCCCACATTAATGTTAAACTTCTTTCGAAATCACAATTCTATCATAGAATGCTTTCGTTTTAACACAAAACTCTAGCCTTCCAAGTCTTAGGGGTAAAATTCAACTGCTATTTGCTTAGAATATAACTGCTGTACTGTTTCATTATATTTTGGTTTCAGCTGCATCCATCAGATACTCGAGAAATTAAATACTCAAATCACATATACCATTGTAAGCAACTATCGAATATTTGCATTACTTATCGAAAAATTGCCTAATAAATAGTTTGGAGGAAATATTGGAGGAATTTTGTGCATAGAATCTTTTTCCATAATACTGTTGATACCTTTCCCTTGTAATTGTACTTCAATTAATAACAAAATAAGAGTATATAATTGCATAACTCGTTGGTATCTTGCACTGAAGGTCTTTTTGTATGAAGGTAGCTTGATGGCACTCCAGTGATTATTTAGCGAAGTTTAGGTATTTGAAAACTTATTTTTCGGAAGAGTAATGATGAGGGATTAGGAGAAGGAAAGAAGATATTAGGAAGTTTGTTGGGGGCTAAACGGAGCACAATAGATTATCATTTCAACCTACTCTCCGCAACAGCTTCTCTACACGAAAAAAAAGAGATTGGGGGATAGCCTTACTTTGAACTCCGAACCTTTCGATCACTTCAGACCAATAAAAGAAACCAAGCAGCAAAGGAAAGCTCAACATCATCAACACTGTTATGTCCCTCTAAACATTCCTCTCCTTTTCCTCCTATTGAGTTACCTCCTTGTATTACCCTTTTCCTTTCCTTTTCTTTAAATTCTCGATGTTGTCTTCTAATTTTGCGACAACAAAGTTCTCTTAGGGACCTTTTCTTTTCTTGTAGCTTTTCTAAGTTCATCGGCCTCCTCTTTCGATATTGATGTTTAGATTTAGCTTTGCATGACAAACATGGAGGGTGGAATTCTATAAAAACAGCAAAAATGTTCGACGAGAATGGCTACACCGATCCCTCTCATGATCCTAACCTCGTTCCAAATTCTTCATTTCATAGCTCCTCTCAAGAACCCCTACCTGATAACGGCCTAGGCGGCGTAAAAGAATGCACTAAGGTCCCCTTCTTTCCGATCGAAGAGTTCTCGAGCCAAAACCATTATCCGTCGTTAGAGGGGACGTCGATAGCCGCGGGGCTCGATCACCTCGAGAATCAACTTGGCTTTGATATAGAGCAAGAGCTTCATAGTCACATCATCCACGGCACGCCTTCGATGGAAAGTGCTACTTGGGAGCCAGCCATGCAAGACATCCAAGATCCGGCTATCTACCTCCAGCAGAACTACGAAAATCAACTGCCTATTCCTGTGGAACAAAATATCCAAAGCAGTGATGGTCTCCTCTATCCAAATTCATACGCGGTCACGACCGACCTTCTAAGCTTCATGAAACTTCCAAGGTGCACTACCAATCCGGATTATCCTCCGATGAGCGGCTTTCCGTTCGAGGCATACAACGAGCTTTCAGGAGCAACAATTCAAGACGGTGGTGCGTCGATGCTTTATGATCCGGCACTTCATTCGGTGGTCTGCCCTACTACTCGATCTCACCTGTTAAAGGATCTTCTCCATTCATTGCCACAAAATTACGGGATGCTCTACGGTGATGAGGGGGATGCCGTGATCGGAGTTGGAGAAGGAATCGGAGGGAATATTTTCCAACCAATCGACGGGAGGAAAATTAGTAGTGCGATACTAGATTGTAGGAGGGAGATTGGAGGACTGGCTAAGGTGGACGGGAAACAAAACTTCGCGGCCGAGAAGCAGAGAAGAGAGCAGTTGAATGAAAAGTATGCAGCTTTGAAATCATTAGTCCCGAACCCCACAAAGGTACTGCTGTATATCATTTTGCAATTATTATCTATCTACTCAAACTTACATCTTTGTGCATTGGTATAAATTGCTTGCATATTGCTCGAGTTGCGACAAATCTTCGTATATTATATTTCCATAATTAACCTTAGTTTTCATTAGAATTAAATAGTTTTGTGTAATTAATTACTTACCCTTCTAAATTGATATGTCAAACCTTTAAAACGCAACATTTTGTATCTGGATATAGCACCCATATCAGCATTCTCAGAAAAAGTCTCATTGACTTGAAGCAAGAAAACCATTTCTTTTCTTTATAGCTTGTTATATAGTGGAGGTTAAAGCATGAGTTTCTTTAATTTATAGCTTCTTAATGTGGACTAGCTGTATATACAATTTGGAGCTAGACCTCCTTAATTAGACTTGTCTCTCTCTCTCTCTCTCTCTCTCTCTATCCCTCTCTCTCTCATCCATAAATGGATTGATGCTTTATCACAAGCTTTTTTGCAATGTAAAAGAGGACAATGTTGTGATACTATTCTTTCATAGTACGAATTCAATATCCGTGATAAATTAGTGTCTCTTGCTGTCAATGTTGTCAATTATTGCTCCCTTTGATAGAAACCCTTGTTTTCAATTTACACATGGCAACTGATATTACTAAAGCAGTTTTCTACTATTGAAAGAGCACAATGGTTCAAAACATGACTAGATACCATGTATGTTCTCACAATAGCATATATTGCTTTCGCAATATCAGTTCCCTTTACGCAATCTTGAGAAAGAAAATTCTCTTAATCTATATACTCATAAGGAGTTAAGAAGACAATTGGTGCTTCCAAATCACAATTTTTTTTTTTTTTTGCGATCACTAATGTTCTCGGTACAAATGTATTCTAATCCCTTGCAATTCTGTCTCATAATCAAGTCTGATAGAGCATCCATTGTGGGAGACGCAATTGAATACATCAACGAGCTAAATCGAACAATCAAGGAATTAAAGATCCTCGTAGAGCAAAAGAGACATGGAAATGAGAGGAGAAAGATGCTGAAGATGGTTAATGACGTGAATAGTGGCGACATGGAGAGCTCATCAATGAGCCCTTTTAGGGGGGATCAAGATAATCCACTAAATGGAGCCTTGAGGAGCTCGTGGATACAAAGGAGATCCAAGGATTGTTTCGTAGATGTTCGTATCATCGATGACGAAGTGAACATTAAGATCACTCGGAAAAATAAGGCCAATTGCTTGTTGTATGTCGCAAAGGTCCTAGATGAGCTCCAACTCGAGCTAATCCATAGTACCGGTGCGACCATCGGGGACCACAACATCTTCATGTTCAACACAAAGGTCCTTTTTAATTTCTCATTTCCACACAGCATATATTATGATATTTATGCATTTTTATAGCATTGTTTGTAAGGTAACAAATTTTTTTTTTTAATCGGTACTCTGTAGATATTCGAAGGCTCGTCAGTGTATGCGGGTGCAGTAGCTAAGAAGATTATTGAAGCTATGGAGAAACAATATCCAACCATAACATTTCCGACTAGCTTTTAAGGCCTTTTACGCGGTGCTTAGAGAAGGCCCTAATTGCTCTGATTCTAGTTAGATGAGACGTTTAAAGGCGTGTTACACGTGAGATATAACCATCTCCAGTAGCACTCAATCGATATCATATCGTCCTTGTTATCACATACTATTAGCTATTATCATACTAAACAATTTTATTTCCTTGCCTTTAAGATTGCTAACAAATATGCGTGTATTCTCATATACTTTCTAAAAGTAACAGTGAACAAGTTGAAAGATTACTACATGC

>Aco024469; *Ac*bHLH27
CACGAGTTCCTCCCTTTCTCTCTCTCCAACTCCTCTCCGGGATACAAAACCCTAGATTCCCGGCGAGAGGAGAGGAGTGGGCGTGGATGGATCAGTGGAAGTCGACGGAGAGCCTCTTCCCCGCCGACCATTCGTCGGCGGCGGAGATCGCCGGCGAGGTCTCCCGTCGCCGCTCCGGCGACGCAGGCCCCGATCGGTCGCAGAGCCCGTGCCCGAATCCCAGGTTTGGTTCAATTCGCTCGATCTTTATTTCTTGTTGAATCTTTAGGAACAAATTTGGTTAAAGTTGTGCTGAATTGGATAAAATTATTAATTTGGAGGACAAATCATGTGTTTTTTGTCATTGGAAGGTGTAATTTTGGCATCTGGATTGATTCGAACTTAGATTGGATTGCTTCTACTTAAAGCTTTAGATGCTGTGAGAGAAGCAAATGTTCTTCTCACATGGTCTCTGCCTTACTTATCTCACCTGGAGTAGCTATTACTACTATGTAAAATGTTAGAAAAGTGTCCTCACTTTTGCAGGAGCTGCAACATGGCCAAATGAACCCTTGGTCTAATTTTTTTTTTTTTTTTTACTTGTAGTTTGAATAGCATTCGAGTCACCTAATCCTGGTAAAGTCCTGAACATGGTTGGTAGTTGGTAACATTAACGAGGAAATGGTAATTGATAATCTCGTCAACCATTTTTTCCTTTCTTTGCTTGCTCATTAAGTTGGTGATATAACTTTTGAGCAAAATTTATTTGAGTTGTTACCTGTTATATTTTCAAACCAACAAACTATGCAAGAATGGCATATAGACTACATTAGAGTTATTTGACGCTCAGCATTATTATCTCCTTGAAAATCTTCTTACATGTTTATACCATAGATACTTGCGCTTACGTCAATATTTGTAGGTACTTTTACTACATGTACTTGCACGCATGAACCGAAAAAAAAAAATGCTTACACATATGTATTCATATTTTGTAATTCTATTGAAGTTTATTGATAACTTAAGTTCCTACCTGCTCCTTGTGTATGGCTATTAACATAAGTAGTTTTCAATTGAGATGCATAAGCACCTGCATGTATACCTGCTTACTATACAATTGTTCAGTAGTTTCTTGATAACCTGCTGTAGCCATCTTTAAACGTTTTTATATGCAAGGGAAGCTTAAGAAGGGTTGTCTTTAGCTAAGAGCAACTTAGACTATAGGATGCAAACGCTCGGAGGAGGACAAAGTTCCTAGATGCTAGAGGTGGCTAGGCAGATTTAGCAATCAAGGCATCTCAAAATTGAGAAATATTAGAAAATAATCTTACTTATTATGATGAAATAGCTTTTCAGTTTAGAACTTTGAGAATTGGATTGTCGAGTTAGCAACATTTAATTCTGGGTTTAATTCAATATTATCTTCTGCGCTGCCAAAGTAAAGTGGCTGGATTGCTAATGCAGCTGTTTTTTCTTTTTTGACAACTCCTAAATAATGAAAATTAAAGGATTTCTTAAGAGTTATCTGTTATGAATGCATATGATTTTATGTCAACAAGACTTATTCTCTATTGCTATTCACTTTCTGAACATTGATGTACTAATTAGCTGCTTTAACATTTTTCAGCCAAAGGGTAGAATATGAAGCCAGAAGTTCTATTTCTGCACGCAAAGTTCAGAAAGCTGACCGTGAAAAATTGAGGAGGGACAGACTAAATGAACAATTCCAGGAATTGGGAAAAGCTCTAGGTACAATGCGAGTACACTTTTACATTTACATATGCTGTGGGAAAATTGACCTGCACACACAAATAGTAATTAATGCAGCCAGAAATATAATAACTCGAATAAAAGCTATATCATTCTGAAAGTAAATGTTCTGATGCAATACTAATAAGTGCCAAGATGAAAATAAATAAATCGACGAATGAAAGAACATATCGGGGTGCTCAGAGCCCGTAGGACTTTTTTTATCTTCAGAAAGTCATCTCGAAATAATATTTTTAATTCACAACAAATTATGGTTCAGCCCGTCTCAATTCTGACCCAAAACCACCATCAATGAGCCTATAATTTGAAAGGACAACAGCACAAGACGCTAGCGATAAACGGCAGCGAGAGGAAGGTGTAGAGGAGGAGAAAACCCAATATAGGGCAACAGGAAAAGAGAAGGGAAAGTGCCCAATGCAGAAACAGCAAGAAGGCAATGGGAGAGTAACAGTTCGTCCATGGCACGGACAACACAAGAGGAGGAGCAGAATAACCAGCAATAGGAAGACAATATCAATTGTCAATCCCAAAAAGTCAGCAGAAGTTCTCAATTTATATGATAGATCGACTTAGATCCGACCAGGAATAACATGGGTAAAGTGGCTCAGCAGTCTGGCTATAAGTTAGACCAGCTGGGGTTTAATAGATTTTAAAGTTTAAATCGAACCCAAAACGCAAACATAAAGCTCCATTGATGAAATCTGAACGAAAAGTCTAAGGCCCCGTTTGGATACCCTTAGAAACGTAGCATAGTTAAACTATGCTACACCGGTAGGAAATTTATCCTATGAAGCGTTTGGGAGAAAAAAAAAATATCGTAATTAACTATACTCCAAAAAAGGGAGTAAAATTATAATCCACTCTAACCAAAGGAAAAGAATTCCACCATTCTTGGGCAAAGATGCATCAAGTTCTAACTTTTAAATTTCAACATAGATTTCAAATTTTAAATTTTAAACTTTACAATTTAATTTATATTCAAATTTTTAATTTCAAATTAAAATTTGAATTTTAAATTTGAAATATCAAAAATTAAATTTTATAATTTCATAATTTAAATGTCAAATTTTAAATTTTAAATTTTTTAATTTTATAAATTCAAATTTTAATTTTCGAATATCAAATTTTAAATTTTAGTTTACAAATAATAAATTTCAAATTTTAAATTTTTTAATTAATAATTTCAATTTTTAATTCTAAATTTTTTACTTTCAAATTTAATTTTTAAATTTCAAATTTCAAATTTCAAATTTCAAATTTAGAATTTCAAATTTCATTTTAAATTTTAGTTTTCAAAATTCAAATTTTAAATTTTAAATTTCAAATATCAAATTTTAAACTTCAAATTTTAAATTTATTTTTTAATTTTAAATTTAAAATTCTAAATTTAAAATTTAAAATTTTTATTTCAAAATTTTAAATTTCAAAATTTTAAAATTAAATTTAGATTTTTATATTTTTAATTTTTAATTTTCAAATTTTAAAATTTAGAATTTAAAATTTCACATTTCACATATCAAATTTGAAATTTTAAATTTCATTGAAAGTTGAAATTTGAAATTTTAACTTTTAATTTTAAAATTTAGAGAAATCCCGTGAAATTGCACAATGCGCGTCCAAACAATTTAAAAATTTAATCCGTGGAATTTTTTTAGAGTTACAGGATTCTCTGTTTCATTTGGACCAAAACTATAGTTTATAAATTCCGTGAAGATTTTTTACTGTGCATCCAAACAGGCCCTAAATGGGTAAGAATTAATCCCATGTAGGAAAAAACTGAGTGGGCAATTAATTAAGGTTACACCGAACTTGAACTGATCCTACAACGCACTAAAAACTAGCGAATACGTCAAAGTAATTTTTTCTAAGATAGAAATCTTTTCAAAACATCATGTATCATCGGAATGCAAACCAAAAATCATCTAAAAACAACTAAACAATCAAAATTAAAATAAAAATTATTAAGCAAAAATTTAAACTAAAAGAAACAAATAAATAGGACAAGGCTCATCCTTGAACTGCATCATTAATCAACTCGACTGCCCCATTTTAACTAGCTAATCACATATCTTAGCCATTTTGTCACGTACCTATTTTTATACTGTCAAACATTTGGATAGAGATTGTATATTTGAATTGTATTTGAACTCTCACCAAAACCTTTGATTTCATTCACTTGTCTTGTTAATAGACGCGGTTGAACCATTTTAAATGATATCTTGTAGCTTATTGTCAATTAGTGCCTTCCCTGTGATTGGCATAATATATTTTATTGTGACACAATCATCTTTTTTTATGTTACTAGGCTGTTTTTCAATCATGTTGGTCTCTAACACCGAAGATAAAAGTAGTAAAATACCATATACTGACAACTTAAGATTTCAGAGACAAGTGGTTATTTCACAAGATATTATAGCAGGAGGTTTTGAGTTCAAGTCTGTCCAATCGTCTATTCTTATTTGATTTCCTTCCATTACATATCTAGTCTACTTCCTGGACCTATCAAATCATCTTGAGCGCGAAAATAAGGTAGGGCATTGAGTATAAAATATTAAAACACTGCTCTCTAGCAGCTCAAGCTTTTAGAGACAAACAAAACTTTCACACTAAGTAGTTGAAGCTTTGAACCACCTTGTTTATTAGTTAACCGTCGTTTTTAAACTTTGTTTTTACCATGTGGCCAACAACAACCATGAAATTAGATGGATCTAGATTTATCTAGATAGTAAAATATAACAACTAATAAGAGATTTATCAAAGGTCATTAAGTTAGGAATTCAAATATTAGTGGCATCCCAGAGTTTCTTCTTTCAGATAATTCTGAGTAATGTTGATTTTTTTCTTTATTAAAGTGGAGAAGCAACAGTAATGTTTTTGGAATTGGATTGGTGTGAGCTCAGAAACCTTTAAGCTACACACTTTTTACACGAAAATAAACATAAACTGTAGAGGCCTGAAAATCAAAATCAAGACTGTTGGACATTCTGCGTGATCTAAAATATCTATAGACTTGTATACAAAAAAATCACTAGGTTTTCAAAACATGTATCCCCTGCATTGAATGATGGTCCTTTACTGATCTGGTCCAAAATTTTAGACCTTTGATTTTGATAGTTCTTGTTTGATGGTCATTTGAAATATGGTCCAAAATTTGCGGCTTTCTGCATTAGCGTTTTATATATTATATATAACTTCTATTAGTATGAATTGCAATTTTTGAACCTCTAAGGTTGGGTTCCTTACCAGCCTGTAAGTAGTTCTGTGTCATTTGGTCCCTCTTTGAAATATGTATTTCCCATATTAGATTCTATTGTAGTACAAAAATAATTTTGTATTACAAGCTACTTATAGTTAGCCTATGTGGTTGAAATGAAATACAGATCCAGATCGACCCAAGAATGATAAGGCTACCATTCTCACTGATACCATCCAGATGCTTAAAGACTTGACTGCTCGAGTGAACAGATTAAAAGCAGAATGTGCTTCTCTCACCGAAGAATCACGCGAGGTAACATCATCTCAATTTTGAAAAGTTAGAACTTTTGGTGAGAAGATTATGGTCCTTCACTGATCTGCAGCACGTTTTGAAGATGTGTACTTGAACTTTAAAATTGTGATTCATTACTTAACCTTACAATAGTATCTCTTAAGTTAGAACCTAATTGTATTTGTAACCTTTTGATGGATTTTCTGTAATGTCATACGATATTTTGGTACTTGACGGAATTGTCTGTAACTGTTTTTTTGGAATAAAATAGGAATGAAAGGTCAGGTAATAAAATCAAAATCGGAGAAAGTTAAGAAATCCATTCAAAATGTACTATAGTATAGGTAAGGTCTGTTAGTTTTTACCTAAACTTTATCTTTTCCTTTGTATAGTTGACACAAGAGAAGAATGAGCTTAGAGACGAGAAAGCCACTCTAAAAAGTGAGGTCGACAATTTAAACACTCAATATCAGCAAAGGATTAGGGTTTTATATCCCTGGGCTGCAATGGACCCTTCAATTATGAGTTTATCTCCTGCTTATCCATTTCCAATCTCAGTTCCAATTCCCTCAGCTCCACTTGCTATACACTCATCACTTCCACCATTTCCCTTTTTCCGAAATCCTAGTTCGGGACCCATTCCCAATCCCTGTTCTACTTTTATGCCATATTCTCAGCTTTGCCATGCTCAAGTTGATCAGCTATCAAGTCAACAGTCTCAATTTCCACACCCAAGTAGCAGTAGATCTCGTCCCGCTGGCCAACAAGATTCAAGAAACAAGTCTTCTACTCACCAACAACCAAGTTGCGGTGGAGAGAGAAGTGATAATCATAGTGATGTGGCAACGGAGTTGGAACTAAAGACTCCTGGATCTGTGGGCCCTTCTCATTCAAAAATGGCAAACGATAAGGTAAAAGATTATCTGATTAGAACATAACTTGTAATGAATTCATGCCATTATATTTGAGCAAATGTGCCTACATAGCAAGTGCCTATCTTGCCTGGTTTCTTGGACAAGCTTCCTACTCCGAAAAGCAGCAGTGTGGTATAAAGCAAACAAATAGTCCAAGTGGGAAGCTGGTAAGTAACTTCTGGTTTGCAAAAGCAGGAGCTCCAATCTAAAGCTTTAGTTCATTGCAGGAGAAGCTATTAAACAGATTCTTAATGAAAAGGAAAAAGCTATTACTGCATTTTCATGGATCTTTACTTAAATAACATTTTTGTGGAAGATCCAGGCAGGACAAATGGGCCCTGAATGTGATGCAGTAGTCCGGCCATGAATGTGATGTAGTAGTTTGGATTTTACTGCGCAGTTTGCTTCATTGCAGAACAAACAGCACACTAGAGTAAAAGTTCCTAACGTAGTGACTGCTATTTTTTGTAGGATTTGTCTTCTGAAATGAGGAAAGGGAAACAGAGGCAGCCGGAGAAGAAAGAGAGCGGTTTTGCTGCAGAGGGCAGCAGTTCAAGCCGGTCTTCTTCTTCCAGTGGTGGTGTGCAGCAGAGCTCTTCTAACAGTGTTGAGGATGGCTCAGTTGCAGATAAATAGCTAGACCTGCGCAAATGGCTTGAAGGGATGCGTTCCGGCCAAGCCTGGTTTCAGGCCTAGACGAGACTAGGTCGGGTCGGATAAAATAGAAGAGAATCTTGGGCGAGGGGGTAACAACTGAGGAAAGAAACAATGTGTGCTCCGCTGGATTTTATCAATCCTCTAAATATCAGACCTGGGCCAGCCGTGCGAGTTTGGGCTGGTCAAATGTGTCAGGCCGGATTTGCTCAAGGCTATCAACAATTATCAAAGAGCTCAGCTTTGACAACTGTGAACAACCATTCTTGCTGAAACGAGACCGAGTTCGATGATTCAAGCATGGTTCTTCTAGCATGAAACCGTCAAACATGCTAAAAAACATGAAGCGATACCAAGTTCGATGATTCAAGCATGGTTCTTCTAGCATGAAACCGTCAAACACGCTAAAAAACGTGAGCTCATCATAATGAGAAAATCCGCTCTGTTAGTAATTCCACATCGGCTAGGAACCAATAATCTGATATCATAGCAGGTGTCTCATAAAGAATGCTATAATATTTTCATATAGAGGACGGCAAGATGAAAATAGTGTTGCTTCATTGGCAGTACTTTGATTTGAGTTGTTGTATACGGTTCGTACATGTATAACCAGCCTAAAGAAAACTCTGTGACAACTTGCTTTGTATAATTATGAAATTTG

>Aco027896; *Ac*bHLH28
ATGAAGTTGAATTCTTCTTCCACTGAATAATGCCTTCTATTGAGAGGGTTGTTGTGAGATAGGCAATAGAACGAGCCCGTGTTATCACGGTGCGGGCCTGGAACACAATTGCAGGCCTCAAACGCAAGTGCTGGCCTCGAACACAATGCAAGTGAGGTACTTCCCTGAATCCTGCATATCATTTAATAATGCAAATGCCTCTCTATCTTGCTTGCTCCGAAGAATGCCTCATCTAGTGTACATCTGAACTATCACAATCTTTCATTTTTCTTTTTTCTTTTTTTCTTTCATCCAAGGACTCGAAATAGTGCTACATTAGAGAGTGACCAAACCTTATCATATAGGGACCCGACTGCATCAAAAAGTTTTTTTTTTTTTTGAGAGAGAAATTAAGGTAGCATGTTATCCGTTTCGTTTATTTTTTTTAGAAATAAGCTTAACTGAAAATATAAATCAATTAAGATTCGAATTTGAAACATTAGATATTAAACATTAAGTTCTTTGTCATTTGCACTACAGACGGTCGGTGACTGCATCATAGAGTATTAACTATCTAATAGGTAAGTTTTATATTACCGGAATTGTCAGAAGGTAAGAAAATCCTAATGTTCCTATGGCTTTGGCACTTTGCTGGCTCCATGTAAGTGTCATCTCGTTACTTCTTTAAATTCTGTGATCTAAAATTTTCAGTCGAGTCGTAGAATTTTTAAGTTAAAATCTGGACTTCCTATATGCTTAACAGCACTGTTGCATGTATATCATAATGCTATCTTTATATATTATTAGTATATTAATAAATTTTTATCATATTTCGCAATGCATGCATAAGTTAATCATGAGCCTCCACATTTAATATATTAATTAATAATATAGTGAGAGATATATATAGTGGAATGCTCACATCATTATTTTATGCGAAGTGACATGGCATGCTCATTCGAATATACTGATTTAATTTTGAAGGAAAAAATATAAAACGTGATTTTTCCTAAACAACCTGTTAGAAATGGATACATCTATTTCATATCTCGAAACATCCATCCTAAAATATAATTTAAAAAAAAATTAAATTATGTCAAATAAATCAGCAGAATACAGGCATATAAAAAAATTAAAAACATAAATAAAAAGTTTTAAAATATTAAGATGCAATTTTTTCTATTAGAAATGACTTAAAAAGTACAGTACCTGACGGTGAGCTAAATGGCTGAGTGCTGGTAACTATGATTTATTTGGTAAGGCATGGGAAAAAGTGAAAAGTTCTGTCCAGAAATCTGCAAGCCTCGGGAAAATGCCTCCCCTGGTCAAAAAGCTGGTTAATTTTGACTGAATCATATACTCTATATTATTATCTGAGCTAGACTAACGTGCTTTAAGAAGTATGATAATTTCCATACTATCAAATAATTGTCCGTACTAAGACTTTTGAATATATGATCGTTTTTGTTAGACTTAATTTAAAATATTTAAAAATTAAATTTTATAATTTTTTTTGATATTATTTATTAAGTAATTATAAAATTAAAATTAATAATTTTAGTAATCGTAGTGAGTCGTTTGTAAGTTTAACAGTACGTAAAATTTTGATAGAAATTTTTTTATACTATATAAAATAAGGTTAATATATTTTTTTTTTTTGAGAGATAAGTAACACGCTATCCGTTTCGTTTATTTTATTTAGAAATAAACTTAGCAAGAAATGTGAATTAACTAGGATTTGAACTTGGGTCTCGGGTACCAACCACCAAGCCCTTTGCCATTTGCTCTAGGGACGGTCGGTAAAAAGCAATATTGTTGATCTAAAATTTTAGTGTTATATTATCACTTTTTGTTAGATTTGTATTTTTAGCTGTTGATTTTGAGTCATTTTGATGATGAAAGAGGCGATATTAAAAAATTATAAAATTTATTTTTTAGGTACTTTTAATATTATATAAAGTCTAACAAAATGGGTTGATTGAAAAGTCTCAATATCAAAAACTATTTTGGAGAGCATTGTATTTCTGAAAGCATACAAGTCAGGCTCTTATTACCCTTACACTTCCATAGTAATTAAAAACGCTTAATCGCTTGTTTCCGCTTCAACCTGAAATTGGAGAGGTTGTTCTAACATATATGCTTTTTACTAGTTTAATAATAATAATAATAATAATAATAATAATAATAATAATAAGTAGATTCCATAGATTTATTGGAACACAAAAATTAGCTCTTTCGAAATCAATATCCTGGAACAAGGGTCTCGTCAGAGAAAAGTGCTTCTGTCAATTGCTGCCCTTGTCTTTGCACATACTTTTTACTCCCAACTCTGTATCATTTGGAAATGCTGTTCCCTCTTGAGATTCAGACCCAGACAGTATATATACAGCATTACAGCGTGGAAAGGGTAGAGGTTGACGGAATAATGACGAGTTAAATATGTTTGCCTCACATCGTCACTTTCATTGCAGTGATGAAGTATTGCTGGTGGTAACGTGTTATCCTATCTCTCTTTGCCATTTTAATATTTTGTTAGGAGTATTTTATCTAAACTTTTTTTGATGTGTAGTGTTGCTCCCAAAATAAATTTTATTTTTAGAATCCATTTGTTTCATGATTTATAATATTTTGATAATTTTTTTATATTTTTTATTAAAAATATTTAAATTATTATTATTCAACTTAAGTCGGGTAGTTTTAAAAAAGATGTTGGTAAATTAGTGAAATCATTATAGATTCTTAGTCGGTTGAATACTAAATTATTATGGATTACAAGTATTCTCATTTTTTAATTTAATGAAAATTATTAGTAGGCCGTTGATAAGAATATACTAGTTTTTAATAACTTCTCAATTCAAATAGTTTGATAAGTTACTAACAAAGTTGCAATTACAAATAATAAGAATATACTAGTTTTTAATAGGGTTAATTTCATTTGTACCCTCACAAAGTTATGAAATATCATAAATATCCTTTGAATTTTTAAAATTTCACAAATACCCCTCTAAATACAATATTTTTTTCATAAATACCCTCACCATTAATTTTTGGCTAACTTCTGTCGACATAGCATTTTTTTATTTTATTTAAAATTATGAAATTACCATTTTACCCTTATTTTTTGTTTAAAAATCAAAAAAATTTTCACTTATTTTTGATATTTATACTAAAACTATCAACTATAGGTCATTTAAGTAAATCCTTGAAGTTTTTATTATTATTATTTTTTTGAACAGAAACTTATTTAATTTTATATACTACGAAGTTATTAAAATTATTAAATTTATTTGAATCATTAATGGATTAGATAAAATCTCTAATTTAATAATAAAAAATCATTCAATTTAAGAAAATAAAATTTAAGAAGACTTTAATTAAAAAGTTAAATTTAGAAGGGTATTTCACAATGGGTAGTAATTGAGGGGGTCTTCATATATTCAAAAAAAAATTGTGAGAGTGGTGATTAAATTAAAATAGATGGTAAAATTGGTATATTGAAGCTAATATAACAGATTGCTAGCGGTGAGGGTATTTTTGAAACTTTTCCATGCTTTTGAAGGGCAAATATGAAATTTTAAAATTTTGACGTGTATTCATGAAATTAGAACTACTTGGAAGGGTATTGAAGAAAATTTTCCTTTTTAATAACTTCTCAATTCAAATAGTTTGATAAGTTAGTAACAAAATTACAATTACAAATAAAAATTATATAATATCTTCGAAATATTACAAATTCTTAACCAATTGTCCTTCTATTGATCGCTAATAATAATATTTTGTGAGACAATATTTAACCGAACAATTTTTGATGCGATAGATGCAATAGATTAAAATGGAACAAAAATTTTATGGGAACTGTCTGATCCTCCGTCCATCATGTGGACTATACGACTTTAAAGCATGATGAACGGTGGATGAGATCGCTTATCATGGGAACTATTCCCATAAAACTTTTGTCCCTTTAACCTTGTTAGGTTGCTGCTTATGTTCGTACCTGCCGTCGTTCTAGTAGTGTTGTTTGTAGAAAGAAGCTATTGGGGGAGAGCATACTGTCTTCTCCTATTACTTCTTGTTTCTAAGAAAACTCCATTATATAATAAGGCCAGAATATGAGGCAAATTTGTCTTGTTTATGGTAAATGCTATTTGTACATCCATTTTTGTATGTACCACTAGCATAGCTGTTTATAAACAGTGATCAAGATTGAGGTATTTATTATATTATTTGTTTTTTTTTTTTTAAAGAGATAGATAGCATGCTACCCGTTTCGTTTATTTCATTTAGAAATAAACTTAGCTGGAAATACGAATCAACTAGGATTCGAACTTAGGTCTCGGGTACCAACCATCAAGTCTTTTGCCACTTGCTCTAGGAACGGTCGGTATTATATCATTTATTAAAACTGCTTATTGCTATTCATATATCACGTACTGACTTTCCTCTTATCTCTTCCAACTAGAGCTTAACAACTATGTAACTATTATACCATTTGTTAAAATTGCTTCTTGCTATCTATATATCACATACTAATTTCTCGTATCTTTCCCAACCAAAGCTTAACTACCATGCTGATGCGTGATCTTTACTTTTTGTATCGAAATTATAGGTTTCTTACTTCACATTACGACAAAGAGTTCACATTTTTCATATGCCCGTTAAATTTTAGCCCGGGCCGTATGTGGGAGTCTTGTTCAATAGAAAATTCTTTGGGGAGATTCCAGGGATAATTTAGCCTAGTTCTAGGCCAACTAGAACCAAGAGGTGTTTCTTTACACTGGTTTGTGTGCGATGCGTGCGACTGCTGGAACGTCTGTACATGTCGTCAAGGCCAACAAAGTCGAGCCATTTTACGAGGTTTGACCGCAAATCTCGGAGTCCTTTATACTTTCTATTTTAAGCATGCTTCTCTATCATCTTATAAGTGCCTTTTGCATAGCCTTCGATTTGCAAAGATTTTTTACGAAACAGTTTTTTTTTTTTTTTTTTTTTTTTTGAGAGATAGGTAGCACGCTACCCGTCTCGTTTATTTTATTTAAACATAAACTTAGCTGGAAATGTGAATCAACTAGGATTCGAATTTGGAACATCGGTTATCAACCATCAATTTTTTTACCACTTGGTTTAGGAACGATCGGTTATTACGAAACAACTTGAGCATGTTTTATTTCTTACTAGTATGATAGAATAGATCAATGTGCCAAGCTACGGAAAACGAGCTGAAACTCTTTGTAACATAATAAGAACCAAAAAAAGATAAATAGAGCTAAAACACCCCATTCCTGGGTGTGGTGGATCAGAAGGATCAGAGACTCAGCACTAATGATCGATAAAAATTTAACCTATTATTAAATAATGATTTTGGATAATAAGGCCAGGATTCTTAGAATTGCAAGGGTTTTAGATAGGTTGTGACAAATTATTATCCAGAAGACCGATTCTCTAGATAATAGCCCGCAAATGGTCTTAATAATTAGTAATGATCTTTTACCAAATGGGGATTAGCAAATTTTCTTAAAATGTTAATCCAAAAATTTCAATGCGCACTGCATCTAAATTTTTTCTTATAGGAAATTTGGACCTCTTTGTCCAATATGTATTAGGGTGGAATTACTTTCAATCTGGGCTGACGTCTTATTAAGACCTACTATCTAGCCTCACTCACCTCTAACTGACTTCTCTCAGTCCGATCAATTATTATTCCGGTTATAAAGGTGAGGTCTCGATTTGAGCCATTGATTAGGCATAGTCAATTGAATTTTTAGTTAGGAATAAATGGCCAAAATCATGCTTTCACTTTTCTTTGTATTTCCCGTGATCTTTGATGCACGGCGGATGACAGAGTCCTTTGGGACTTTACTACACAGTGGGCGGGAAGCTGTTAGTGTCGTTTGCATATTTGGTCTATTGCCGAAACTTAGCGTTCTAGCAGATCCAGAAGGACGAGACCCCATCTAGCATTAGCGGCTTCTTTTGAGGGAATGAAATTGAGTTTACCCCTGATCTGGAGGTTTGAGGTAATCATAATCCTAAACCATGTGTAGAGGGTGTTGTTTTCCTCTTTTACGTCTTATCATCAAATAGACGATTAGCTGCAGGAGAGAGAGAGAGAGAGAGAGAGAGAGAGAGGATGGCAGAGTGGAGTGGGTAAGGGAAGGCCCCAGGACTGTCTTCGCAGACGGACAAACCTGCCAACCAAAACAATAATGCTGGTCTTGCAAAAAAGACAGCGCCACAATCCCCAGATCTATGTAGCCTGCGCCAACCTTTCTAAGCTTTTCCGCCCCACATCCACCACAACTACAATTACATTTTTAACCTCCATCCTCCCACACGGTTAATTGCTTGTGTATTTTGTTTATTGCATATCAAACAAAAACTGTTTATTTCGAAGATAAGTATAAGTTTAGATATAAGTCTGAAATATATCAATTTTGAATTTGGATAAAAATTGAGTTGTTTTCGAAAATAAGAAAAATAACATTTGATAGATAAATGAAATAAGAAAAATAATTAGCGCTAAATAGAAAAAAAATAATATTACTCTTTATATTATAAATTTTTAAAATTTAAATTTTTAAAAGAATTAAATAGGGGTGTGAACACCACCCATATTTTCACACAAAGAGTGGGAGCTATCCCACCCCTTAACCGCAGGGGCGGAGCTATTGAGGGGCCCACAGTGGTTCACGGCCTTCTCTAACTTTTTTAAAATTACATAAAAAATTTTTGTAATAAAAATTAAAATATTATAAATTTTAATTGTAGCGGGCCAAATTAATTTTTTTAATAAAATTTGTGTCTTAATTTTATTTTTTCAAATTTCTTGATCCTTTTTCTTCAACTTTTACAGTTTTATTCTTCTTTGAGTATATTATCATTATTGTTGCTAGTAATAACTAATCAGTAAACCTGACAATTTTTTTTTTCTTATTATCTGAGGTTGAATTTTTTTCCTATCAACTCTAAATCTTGGCCCCCCCAAATTTTAATTTCTGGCTTCGTCCCTGCTTAGGATTATTCCGAAATAATTCGTTAATAAGGAATCAGATAGATATCCCACTCTTATTCCCTAACCAAACGAGGTGCGTTAAGATTTTAATATTGAAAAATTGAATAAGTATATTCAAAATAGTTTGTATTAAGATAAATTTTGTATATTTTTATTATTTATTTATTATTTTTTTCAACAGCTTAATGTGGTATATATAAATTATTTGTGCAAATTAATTAAAATATTTTATATGAATTCGTTGTATATAAAATAATATTTTTGGGAGATGCTTCCGCTGTCAGAATCCCCACAATTGCCTTTTGTCACTACTCAATTACCTCATGGTTTATTACCGTGTATTAGTTTACTATTTATCATCATCCCTGCATGAGGAGGCTTTTTTACTCGCCGCCCCCACATCTTTTCTCACCCACAACCCTTCTACCATATATTGTTCTGCAGGACCGCTCCTCTCTCTTCTTCTCTCTTCCATTCCCTCTCATTCCATCTCCTTTGTTTTATTCCCCTCCCTAGTCCGTCCCCAGCTCTCCTCCGTAGTCGCACTCGCTCCTTTCTCTTCGGACCACTAGCCCTTTGAAACAGAGGAAGCAAGAGAGTAATGATTATTGTGGGCTTTGTTCCTTGTGTTGTCTCGCGTTTCTGCCTCTGCATTTAATGCTGCGGCTGGGTGCGTCGAGGATGGAATTAAAGGAGCACCTTTCGGTTCGATTCATGAAGAGAAGTGGTGGTTGGGGGATTGTTTAAGGAAGAAGCAGAGAGTCAGAAGGGGGGTGGGGGGGTGGGGTGCTACTTGCACACAGACTGCTGCGAGTGGGAGTTGTCAATTCCCGGCACTGCTTCCTCTGTTTGGTGCTGCTGCTCCTATGGAGAGGCTTCAAGGGCCCATTAATCCCTTCGTAAGTGTCCTTAAATACCCCACCCCTTTCCTGTTTAATTTCTCTCTTGCGCATCATCTGAATACCCCTTTCCCCCACACCCCAACCCCCCACAAACCTCCTTTTTGTGGATGGAAGAAAGGTGGGTACCGGTTCCCCTTTATGGACATTTCATTCAATGCAGCTGCATTAGAAGGAAGAGGATAGAGGTGGGATTATTTTGCCCTCCTTTCTTTGTCCGTGGCTTCCTTTTTCGGGTCGGAAAAGTTTCCATTCAATTTCCTTGACCGTTAATGGGTCCGCGTCATTTGCCTGTTTCGCGATAACCCCCCTGTGTAAAGTTTCGATTTTTAAAAGAGAACGCACTTGCTAATCTCTCCCATTTTACTTGGAAAATAAAGCAGTTGGGCGGAGAGGAGATGAACTCGGAGTGCTTGGAGACAGGGGTGGCGAACAGTAACTCTGCGGTGGAGATGAGCTACTTAAGCCTGATCTCGTCCCATCATCATCGTCATCATCATCATCATCGCCAAACTCCTTTCCTCCAGTTGCTACTCCGAGGAGCAATGGGAGTAGAGACAGCGGGGGAGGATGAAGAAGAAGAAGAAGAAGAAGAAGAAGAAGAAGAAGAAGAAGAAGAACGGCTCCATTCCCAATCTCTCTTACCCTCCTATCCTTCCGAATCCCAATTCCAGCTCCTCCAACTACAAAGCAACTGCGGCAAGCGGGATCGGACGGCTGAGATTGATGTCTTTTCTGCCGCCGCAGCCCCCGTGCCGGTGGAGCAGCTCGAGAGCTGCATCTCCCACGCGTCGGAGTCGCAATCCGATGTCAGAAGGCTCGGCCACGACCATAACGAGAAAAAAGTGGAAATAATTATGGCGACGGCGGCAGCGCCGCGGGCCAAGAAGAAGAGGAAGAGGGCGCGGGCTTCGGGGTCTGCGAAGAAGCCGGAGGAGGTGGAGACCCAGCGGTTGACCCACATTGCGGTGGAGCGCAACCGCCGCCGTCTCATGAACGACCACCTCGCCGCCCTGCGCTCTCTCATCCCGCCTTCCTTCGTCCAACGGGTACGTACGTTCCAGCCCTCACTCATATTCAAATTTCATAGGCGCTCGAAATGGCAGCCGTCCAATCTCTCCTATCCAACGGCTGTGATCTGAAGATTCCCGTGTTACGCGCAGGGGGACCAGGCCTCCATTATTGGTGGGGCCATCGACTTCGTGAAAGAGCTGGAGCAGCTCCTCCTCTCACTCCGAGCCCAGAAGCGGATCCGTACCTCGGCCGCGAGCTCTTTCCGGTGCACCGCATCCGACGAGGCTCACGACCCAACCCAGGAAGGCGCCGTGCTCGATGGGTTCTTCTTCTCGCCGCAGTATACGACCTACTCGCAGCAGCAGCGGCGGCGGCTACAGCAGGAGGGGGAGGAGAAAATGGCGGCGGCGGTGTTTGGGGTGGACGTGGAGGCGAAGGTGGTGCAAGGGCACGTGAGCCTGAAGGTGGCTGGGCCGCGGCGGGCGGGGCAGTTGGCGAGGGCCATCGCCGCCGTGGAGGAGCTCCGCCTCTCCGTCCTGCACCTCAACATCGCCTCCCTCGACGAATCCTCCGTCCTCTACTCCCTCAACCTCAAGGTCTTTCACCCTCATCTTCATTGCTCTCTCTCTCTCTCTCTCTCTCTCTCTCTCTCTCTCTCTTCCTCGACTTTTCTATTTTTATGCATGACTCTGGAGGATACTCCTGAAACAAGATAAAATCTACATCCATGTGATGGCCGCGGGATTGCACCTGCCGACCATGGAATTGCTACCGCAGAGTTACTCCGCGCGTAGCGCATAGCATTCATTGTAGGCAATGCGCCACGGATAATCCATCCGAATAGTCCGTACAAACGTAATCCCCGGACGCTAATTAGTGGAAGCTAATCCAAATGCAGATGGAAGAGGACTGCAGGCTCGGCTCCGCCGACGAGGTGGCAGCCGCTGTGCACCAGATCTTCACCTGCATCAACGCTGGCTGCTGAACGATTCTTTCTTATCCTGTCATCTCACGTCTCTTTCCTTCTTTCCCTCCCCGACACGTGGAGCAGAGAGAGACAGAGAGAGAGAGAGAGAGAGAG

>Aco025839; *Ac*bHLH29
ATGTCTCATATTGCTGTTGAGAGGAACCGGCGCAGGCAGATGAACGAGCATCTCAAAGTGCTTCGCTCTCTGACGCCATCTTTCTATATCAAAAGGGTATGCTATTCTCCATTCCGACCTTAATCCTAGTTATACAATAATACTGTTTTGTTCCATGTATATATCACGCGCACGTATCCACGTCCGATGTCGACGATTCTTGGACGCGCGTTCAAGACCGGTCCATAATGCAATTAAAAAGATCTATATATTTTCTTATTTTACATTTTTTATGCATATGTAATGTGAGATAATAAAAATAAATATTTTATCTTTCTCATTAATGTGTACAAATTATTTTTTGGACGGTATAAATAAATTATATACAGTAGAGAATATATTTTCTTAAAATATATGAACTATTAATGAAAATTTTTCTAATTTTCATTCGTATAAGAAATAAGATAATATTTTATCATAATTAATATTTTATACATAATAAATCTATATTATTATATTAATAATATTATAATTTATTAACGGTGTGCATGTCGTATTCATATCTTAATACTTTTGAAACTGTTGATTTGTTCATCCAAGTCATGTCGTGTCCCGTATCTCATATCAGTGCCCGTGATGTCTAGGTTTGATTAGATGTAGCAATATATAATAACAAACAGATTGTATAAAACAGGGAATTTGAAATGTGGCTATTTTTCAGTTTTTTTGTCCCTGTATACATGTGGTTCATTAATTTAAAACTCTTTAATTGATTTACGGACAATTGTTAAGGCATAGAATTAATTATCTCACCTTCTTTCTATGGATTATGTTTATTTACTTGCTTTAGATCATGATTATATCATTACCTTAATTATTATAATTCACTAGCCATGCACAAATTAACTACCATATATGCTGAACAGTGGAGGTCTAGTGCTGAATCAATCAAATTTTCTAATTTGGTTAATTTGTAGGCAATTTGAAGTTGCATATATATATATATATATATATATATATAGTCCGGCTACTATAGATTCTTATGAGTACGATCGTCCTCGTACTCATAAGTTGTTTTCGATGATTGTGCTTCCGAATCGACGATCCACACCGTTAAATGTTATCTAGAGCATTTAAAATGTCTAGAAATCAAATTTTATAATTTTTCGACATCATTTACCTTACGATCAAAAAGTCACAAAATTGACAATTTTTAACGACCGATATGACATATTTGCTAGTTTAACGGTGTACAAGAATCGAAATCAGTTGAATTTTGGATAGAAAATTCTAATTCTATGCACTACATAGATAAAGATCAATAACTCCGATCTTAAATTGAAAGATCTGATCGTCCATTTTTAAGACGTCGTTCGATTTTGACCGTTCATTTTATACCCGCTTGATGAATCTTCTTATGATTTCAAAAAATTACGAAATTTATTTTCTAGAAGTTTCAAATATTCTAAATCATATTTAACGGGGTAGATCGTCAATTCGGAAGCTCCATCATTAAAAACAACTTATGAGTGCGGCAACCCTAGCCTATATATATATATATATATATATATATATATATATATATATATATATATATANATATATATATATATATATATATATATATAGTGATTTTTGTTCAAAGGCAAAGACAATAAATAAGATTTTTCTAATGCATCTAGCTTTAGAATTATGTGTCCACCAAGCAGCTGCCGCACTTTCACCACTGTCTGGTTTGGCTCTTGATCAGTATATATCAACAATATATATATATATATATATATATATATATATATTGGGCCTAAATTAATCATTTGCATGATACAGACTCTAAATACAATATGAATTTAAAGCAAGAAGAAATTCTAGACTTCCATGCTAGCCCATATTTTGGGTAATTCAGTGCTTTGATATTGCTAGTTTTTGATGGACAAATATTAAACTACGGCAAGATCAAATTAAAACCACGGCCGGCAGGCATAATCTGATTTTGGACGTCCTTAAATAATAATTAATATGCTTGCGGACGTCATTTGATATATTGCGGCCTTGTTAATGCATGTTAATTTACATTGTAAAGGGCGACCAAGCATCTATAATTGGAGGAGCCATAGAGTTCATCAAGGAGTTGCATCTCGTTCTGCACTCGCTGGAGGCCAAGAAGAAAAGAAAGAGCTTAAGTCCGAGCCCTACACCAAGCCCGAGATCGCTGCTGCACTTCAATCCATCTCCGCCCATCACTAACAACTCATCACACGATTCAGAGAATATTGTGAAGCAACTCGGAGCGTGCTGCAACTCGCCGGTCGCCGATGTGGAGGCCAAGATCTCGGGCTCAAACGTGCTTTTGCGAACGCTATCAAAGCGCGTCCCTGGTCACGTCGTCAAAATAATAACTTTGCTAGAGAAGCTTGATTTCGAAATCCTTCACCTCAACATCAGTAGCATGGAGGATACTGTGCTTTACTCCTTCGTCATTAAGGTACGTACGTAA

>Aco002151; *Ac*bHLH30
ATGCTGCTGCATCCGCGGAGCGCGTTTTCGACGTACGCGAGAGTCGGATCTCACGATATTGGCAGTCTTGCGGCGGGGCGGCACGTCGGGTCGGCGGCGGCGGTTAACGTTCATCGGAGATTGTTTGGGTTTATTAGAAGGATTGGTGATGGGGGGGTGGCCGCAGCGGGGGTACGGGCGGCTGCGGAGGCAGAGGACGGGGTGGCGGGCGTGGGGCGGAGCCGGGGTTTCCAGCATATGATGAGGGAGAGGTGGCGGAGGGAGCGGATGAGCCAGGGCTACGCCGATCTCTACGCCATGTTGTCTTCTTCGTCCAAGGTGATTTTATATGCGTCACGGCAGTTGCGAATTTAGAAAATTTTATTAGAGAAATCAAATATGAAATGAAAAATAAATATGAAAGATGTAATAATAATTATTTTTGATGTACTTTTATATTTTGAAATTAATGCATAATGAAATTATTAGATTAGGATTTTTTTTTCACTAAAATCACTAATGTATTGAATGAAATTAATGTAAAATATTTTTTTATACTTTAATAAGTGCTTTTTTAAATTGGACTCCAAGTTTATAGCAAGACAGTTTTTACTTATTTATTCAAATATAATGGCTCAATGAGTCAATAAAATACGGTTACTTATAAAATGGACCCCACTGTTGAAAATTAAAATACGGTTACTTATAAAATGGACCCCACTTTTGAAAATGTGGGGACTTTTATTTCACTTTTGAAAATATGGGGAATTTTTCTTGGGAAATAAAGGTAATTTTTAAAAAGTTAGGATACGACCGTAGCAAAGATTATCGATAACTAGTGGAGTTCTCTTGTATACTCAATGACCCATCCGATGTGGCTCAGTTAAGATTCATTTCACACTTTCGACTGATAACCATTAACAATAATAACTAGTTAAGCCCAAATCACCAATACAAAATACATAAACCTAATTATTATTGTTAGTGTAGAATTCTCTAATATATTCAATGATAATTTTTTTCTCATCTGATGTAGCACTATTAAGGTGTCACAAATAATATATATTTATTTATATAAATATTAATTTTGCTTGAATATTATTGTATTTGTTATATTAATGAAACTCAATAAAATTCACGTTGTTAGTTTATATATATATATATATATATATATATANATATATATATATATTTGCACCGTACATGATTTATTTATACCTCTCAAAATTCCGAAAATATCTCATTTACCTTAATTTTTTTTTTCTTTCCAATATACCCCTCATACGTTTCTATGTTATTCCAAAATACCCCTACAGTTATTATCTGTTAAATTAATTTGGATTAATCGTGAGTTAAATATCTACCAGAGTTAAAAAAAAATCAAAATGCTTCTTTTCTTCTTAACTTAAGGACAAATAAGAAACGTGAGTTAAATATCTACCAGAGTTAAAAAAAAAAAATCAAAATGCTTCTTTTTTCCTTAACTTAAGGACAAATAAGAAATGTTGGTGATGGCAGAATGATATATTTGAAAAGACCAAACAGCAAAAGACTTTTGTGCCCTTGACTTAAGAGTAAATAAGAAAAGTTTGTGATAGTATAAGGGCATATTCGAAAGAATAAAATAGTAATTTTATAAAGATAATAGTTATTACTAACCGTTAACAGAATTTAACTCTAGAAATACATTTGAAATAGGTCAGAACGTCAAAAAGATAATTTCAATATTACCCTTCTAAAAGAGATAAATTAAAAAGTCGAAAACTTTTTAGAGGTATATAAATAATTATCCCTATATTTTTAATAGGACGCGTTATGAATCTGCATTCAAACGCATGTTCATATTATGTCCACGCTTATAGAAGTGTGTGCGATATATAATTTTTTTCTCTTTTAGCTATACAATAATTTTTAATAAAATACTTAATAAATAAAAGAATTATATACATGTACATGAGACAAATTCTAGTAAATCCTGCGTGAACTTCCATTTTTTTTTTTTTTAAGGGGTCAAATTTAGTGTTCTGAAAATGGTGCAAACAGGGGGATAAGATCTCGGTCGCGAAGGCGGCCGCTGCGCGGGTAAGAGAGCTCAAATACCAGAAAGAGCAGCTGCAGCGGCGAAACGCCGAGCTCGCGGTCGTGGTGGCGGCGCAGCGACCGGCGACAGCAGCAGTGGCGATCAACCTGACGATCGCGAATTGCTCGACGCCGATGGCAATGGATGCTTTGACCGCCGCGCTCCAGTGCTTGAAGCTCATGGAGTTGAAGATCACCGCCGTCCGATCGAGCTTCTCCGGCAAGAATCTGTCGGTGATTGTGGGTGCGGAGACGAAGGTACGCTCGCATGTAATTTTTGTGCATGTGAGATTGATTGGTTACGTGCATGCATGTCTTAAGTGA

>Aco002225; *Ac*bHLH31
ATAACATGTCTTTGCGCCATATTCACACTTGCTGTTGGTGTTGGATTTAGAGAGAGAGAGAGAGAGAGAGAGCGAGGAAACACGGGCAAAGTAGTGAAGATAAAGTGAATTTAGAACTCTTGGAAAAAGGTTGGTTAATTTAATTTCCTTGTGTCCTCTCTTTTTTTTTTTTTTCCCCCTTGTTTTCTGGGTCTTCTAAATTTATGATTAGACTCATTTTTGTCTCCTACTTTTATACATAGTTTTATTAAAAGATGGTGTTTTTTTTTTTTCCTTAATTTTCGGCATCTTTTTCTGCTTGTATTTATAAGTTTTCATCAGTGGTTTTTTGAATATATTTTCTTTTCTTTTCTTTTTGTGTGCCTTTTTTCTGCTAGATACATGAATTTTCTGAAATGGGTTTCAGTTTTTCTTTTTTTCTTTTTTCTTTTTTTCAATCATTGTTGGGTTCCATGTTGAGTGCAACAAACCAAATATGTTCTCACCATTTAAGTGGGAATGAAATTCTAATCCTCCATTTCTTTGCAGTTGTTGGCTTGTATCTGCTGATTCACCTGTAAATTTTAAGCAGAAACTTCAAATTAGATTGTGATGCAGTAAAATTAATCTCCATTTCATTCTATGAATTGAAGTTGTTTCAATTTTTTTTTTTTTTTTGATCTTTTCCTTTCATTCTTCATAAAAATGCAAAAAAAAAAGTGGTAAATTGTTTTCAAAATTGTGCTTTTTAATCTATTAGGTTTCTTCTCCTTATCATTCATTGTATTGTATGATTATGTGACCCTTTTTCACTCTCTTCTTTTTTGCTCTGCTTTTTTTTGCCTTTTCTCTTTTTCTTCTTTGGTAAGTTTTATTGATTGGTTTTCAATGCAATTTTGGCAATTTTCTTTTGCTTTTTTCAGTTGTGTTAGATCTGTTCCTTTTGTCTTTTGCTTATTTCTGTTTTCTTCTAATTCTTCTTTTTCCAAAGTGCAAGTCTTTTAAGCAAAATTTATCTAGAAAAGCAAGCACATAAAATGAGAGAGAATTTTTTTTAGAAAAAAAAAAAAAACCTGCTTTTGATATTGAACAAAGTGATGTTAACACTATTTTCTAGTTTCTTAGAATTTCCCCTGTTATCTTTTCTTAAAAGTAAATCTTTTTATAGGAATCAATTTAGTTCCAATCACAATAGTGATTCTCATCCCAGTTTCTGATTCATATTTACATGATAGGCAGATAATTTATTACAAACCGATTCATTTCCGATTCAAGTATGGAGGAGAACTCAAGATCTTTTAGTGATCTTCTCGCCAAATCGAACCAAATGAAACCTATCAGGTTAGTGTACTTGCTTTTCTATCCAATTTCTTGTTCATATCTCTAATTTCAGAAAATAAAAGATGCTACATGTTCACTTAATTTCATCATTTGTTACCTTACTCAATTTATGATTCGATTTTACAGACCAGATAATGAGCTTGTCGAACTTCTATGGCATAACGGACAAATTGTTTTGCATAATCAAACTCATCGGAAATCGCCGCCGCCGTGCATCGATTTCAGGCAATCTCAAAATCCCAAATCGGTACTAAAATCGGAGATAACGAATGTAAATGCGAACAACTTAGCGCAAGAAGATGAAACAATGTCATGGTTCCAATACCCGATCGATGATCCGTTAGAAAGAGATTTCTACACCGAGTTCTTCGAATTGCCAAACGGCGGTTTCAACGATAATTCGTTAGGTAAAGAAAAATGTGCAGAAACAGAGAATGAATCAAATGCAGTTAATTTAGGAAGCGGAGTTCAAGTAGGAGGAGGAGGAGAGTGCTCTTCGATTATGACAATAGGCTCGAGTATTTGTGGGAGCAACCAAGTTCCAACGCAAGTCGAGGGAAGCAATTTGCATCATCTTAATACGGCTAAGTTACCTATTGAGGGGAATAATTATAATTCAAGCACACATGAAGCTACTGCTACTTCATCTTCAGGAGGATCCGGTTGTAGTATCGGAATAACTCAACAACAAAGCATAAGCAATCAGGGAACGAAAAGAAAGGAAAGAAGTACCGAGGAATCGGAAAGCCAGAGTGAGGTATATACACTTTACCTTAATATTTCTCCTAATTTTCAAAAGGAAAGATGTAATTAAGAGGTTCAAACAGTTCCTTTCGCTTGAAACTCGGAGAAAATAGGAAAAAGTTTAAGAATGTGCCCAATTTTGGGTCTTTAGGAATCAAGCCCCTGAGCTCTAGGTTGTTTGAGTTTGGAAAATTTCATCAAAACCTCAATCTTTGTTGCTGGGATTTAAAGTTAAGCGATGAACTTTAAACGGCTTAAAGTGTGGGGGTCTCCCTAATCTTCTGCCGAGATAATAACATGGAAGTTGTTAACCTGTAGGAGGCCGAGCAAGAGTCTATCGAAGCGAATGAGCCAACGAAGCAGTCTGCGTCTCGTAGAAGCCGCGCGGCAGAAGTTCATAACCTCTCAGAGAGGGTAAGACTTACCGACGATCCCGAAATTAAGCCAGCCTGCCTAATTCCTCAATCCCCGTATTCTATTGCTAACTCTTTTTTGATGCAGAGGAGAAGGGACCGAATTAACAAGAAAATGAAGGCACTGCAGGAGCTCATACCTAATTGTAACAAGGTAAGTATCTCTTAAACATACTCTTAACCAAAATCTTATTGATGTTTGCATAATTCGTCACATCAAATGCTTATTATTTGCTAACTTTCCTACATCTCAGACCGACAAAGCATCAATGTTAGATGAGGCTATTGGATACTTGAAATCTCTTCAACTTCAGGTTCAGGTAGGAAATCGCGAATACCGAAACTACGCCTTTTATTTACATATATGTATCCTATAATCTTATTCTAATGCGAAGCCACTTCACTCTGTGATTTGGATATGACTCAGCTCGAAATAAGGATGTTATTGGAAGAAACTGCGCAAATGATTTCACTGGGATTAATGTATAAAAAATGCCCTTGTGTCTGTGCAACAGATGATGTGGATGGGTAGCGGAATGGCGCAAATGATGTTCCCCGGTGTCCATCAATTCATGTCGCACGCCACCATGGGTATGAACCCTGCTTCCATGCCTTCAATGCATTACCCAGTTCAGATGCCAACTGTACCGTTTTGTAGTTCCGAAAGTTTTCCGAATCAAATGCAAAATATTAACTTCCCAGGTCTCAATAGCATGCAGGTACTACCATCCCAGGTTTATCTCTAACTTGTTATCGTTGTTTAAACCCTTTCTTTATATTTTTCCGACAAATTATCGAACTCTATGTGGCTTTTACATGCAGGTGACAAATTTTTGTGTTAATGGAGCTTGCTCGTTGCAGTCGAATCAGATATCGGTGCTACCTAGTTATAACCTCTCACATGCTATTGGAGAATTTCCTACTGAAAATGTTCGAGATGATAAATCTAGTGAGAGGAAAAACTAATGGTTTTATATGATGTTTTTTGCTGATTTTTTTTTTTTAAAAGTAAAGTGTAAGCCTGTATTTATGTAAGTAGAACGAATTGTCGGAATAAATGAGCATTATTATCTATGTATGAGTAACATGTATTTTTCCAAATTTTCATATTCCTTCGGGAAATATTAGAATCTACATTTCAAACGATCTATGTATAAGCAAAATGATATCTATCACCTAAGGAACAATAAATGGAAGTTGTTAAGGTGATGGAAGATAATATTTTTAAGTTAGTTTATGTGGTTTTGATATTTTAGAGTGACAAAGTGAAAATATGGTATAGAGAATTTTCTGCATTTTAGCTTAATATAAATTACAAGAGTATTCTGAGGATCCCCTCAAAGTGAAAGTCTTCTGCTGGCTCCTTCTTAAGAAGAGAATCCTCACCGCCGACGTCTTAGTTAGAAGAGGATGGACGGGTAATACCGTCTACGTACTTTGTGGCGGGGAGGAGGAAACAACGGACCATCTTTTCTCCCGTTGTGTCTACTTTAGATTTCTCTTAGTGATGACACTAGATGACACCGAAATTGCAGGCTTGGGACTTGGCGTCCACCCGCTCTTGGATAGTCTGGCGGCTAGGTCTAGCATGCCTCCGAAGGCAAGGCTCGACGTTCTAGTAGCTGCCTGGTGGGTCACTTGGGAAATCAGGAAC

>Aco002246; *Ac*bHLH32
ATGCAATCTGAGCTAAAAGGTTTGCGTAATTTCTTCTCTATATATTCTGTGCATCTAATAGGCTCTTTTGTAGATATATATATCACATAATCTGGACTTCTCAAAAGAAATTTTATTAGAGCAACTATACATATGTTGGTATTTTTGTTAATTCCAAAAGATTGGTTTTTGGCTTTTGAAGATCAATGGAATCAACCCATAATTTCCCTTTTCTTTTGTCTTTTCATGAACTTTTGCCAAAAATATTGTGGAAACAAAATTTCTTTTTCTTGCTTGATCTACTGTTCATGTGGTCCTTAATTGATAGCTTAGGATTGCTATTGCAAGAAATATGATATTCCTAATTCAAACAATAAACCCAAATAGCCAAAAGGATTTACCTCACATCAATCTTTTTCATGAACATATATAAAAAGCTGTTTGATCTATAGCTTAGCAATAATCATCTTAGTTCATAAAAGTTGACTTTGCACTGTTTAGTGCATTATTGGCCCTAACTTTTGTATTATATTGCAATTTTTTTTTTTTTCTCAAAGCCTCTCTTACACACTGAGGATACATATAGGTCATGTTACGGGTTCTAATTCGTCATAAACTTGCAAGATCTCATTACCATGCATGAATTTCTTGAAGTTTATGATCATCTACATGAAAACCCTAAAGAATTTCATCATCCTTTTGTTTTCTTTTATTTATTATTTTTTTGGAGCTCTTATTGTTCATGTGATTTTATTTGATAATTACAAGTTTTCTTAATTAGCTTTTCCATCATAACAGTACTGATCATGGCTATGCATCTTAATTTGTAGAAATCTTTTTATTTTATTTTTTACTTTTTTCTTGGGGGGGCTTGATTGATTTGTAGGAATGGATGATTCTAATATCAGCCACCAATGGACTATGAACTCATTCAACCAAGTTATTCCCTCACACCAAATAGTAGCTGCAAGTGGTGAAAGCTCCCAACCATCTCTCTCTCAAGAGAGCTTTTCCTCTTGCCCGAATTCCTTCTACACAACAAGAAATGATGTTTCATCTTCAAACTCTATCTTAACTCACAGTCCAAATTCATTATTTGGCTGGAGTAATACTTTTGGAAGGCCCAGTACTCAGCCAAAGGAAGAGGTGGAGTTCGTAATATCTTATAACAATTCGGAACAAAATTACGAAGCAATCGGTAGCAAACAAAGATCGAACATGGTCAAGTTTGGAGCGGCTTCGCCCCAAGCGCAAGATCACATTGTCGCGGAGAGAAAACGGAGAGAGAAGCTCAACCAAAGATTCATAGAGCTTTCAGCAGCTATCCCAGGTCTTAAGAAGGTTGAGATATGCATATGTTGTCTAATTGTTCTTTTTCTCTTTGTACAATATATGATTGCATTGAGGTTAATTGGTTCACAGAAATATTATTTCTATTACATTAGTAATAGATGCATGCTTTTATGATATTGGGATTAATTACAAATGCTTAAGAGATATACCAATCTAGGATCTTAACTTGAAGTAAAGAAAATAAAATAGTGTTAGGTTTTTCGCTGTCGATCCATTTTAAAGAGGAAGCAATTAGCTAGATGTATAATTTGATGGGAAAAAAAAAGCACTAGTCTGTATCAAAAAGATCACTATTATTTTTTGTTATGTGAAGTTTCTAGCTTTTAATGCTATATATCCACTCCTTTCAACTAATAACTAGCTAGATTTGTTATATATGAAGGAAAATGAAAAAAAAAAAAATTTCTTTCTTTTTTACTGGAATCAAGTGTAGTAGTTTGCATGAAATAATAACCATCAAAAGTTTAACATATTCCTGCTTTTTGACATATTCGATTCGTATGTATTAACCTTCATTTTATACTTCTTGTTAGATTTTCTTCTTTCCCCAGTTTAACAATAAAAGAATTAATTATTAATTATCTCCATCTAGTTATTAATTGTTTTATTTACATTTTCAGATGGATAAAGCTTCTATTCTTGAAGATGCAGTAAAGTATGTAAAGGAACTCAGTGAGAAAGTGAAGACACTCGAGGATCAAAGTCCGAAGACCATCGAGTCCGTAGTCCTCAGGAAGAAGTCTTGCCGCTCTTGCGATCAAGATGGTTCAAGCTCTTATAATAATCATGACTCCAAAAGGTGCTTATCGGAGAAGCCCCTCCCGGAGATCGATGCGAGGATTCATGAGAAGAACATCCTTGTGAGGATACATTGTGAGAACCTCAAAGGAGTGTTGGTGAAAGTGCTTTCTGAGATAGAGGAGCTCCACCTGAGTGTCACTAATACTAGTCTCATGCCCTTCCAGGGTGGCTCTATCATAATAACTGTGATAGCACAGGCAAGTTCCTCACAATTAAATTCTACT

>Aco002333; *Ac*bHLH33
AGTATATAGATTTGAGCCTAGCAAGTTGCACCAACATTTTCTAGCCTCATACCATCATGTTAGCCCTCTCGCCGCCGTTGTTCTCTTCGCCGTTAGAGAACAACGTGATGAGCAACGAACTCCGCTCCGATGATCGGATCTTCGCAAGCAACCGCATCCAACATGAAGATGGCATGCTCCAGCAGTCTCTCCTTTTCCCTTCTCCGCCGCATCCCGAGCTCGAGTTCGACGACCAGAGTCGTGCCAGCAACAGCGCAAAGGACTCCCCAAGAAGCCACAAGAAGTTGAGCCACAACGCCTACGAGCGCGACCGCAGAAAGAAGCTCAACAGCTTCTACTCGTCTCTCCGCACATTGCTCCCTGAATCTGATCGAACCGTAACGTCCCGCGAACTTTAGCTACACACTTGCTCACGTTGCACTTACAATCAAACTCCGTAGTACTCACTTCGATATCTTGTGTACGTAGCAGAAGAAAATGAGCATTCCATCGACGGTTTCCCGCATCGTGAAGTACATACCCGAGCTGCAAAGCCAAGTCGCAAGGCTGTCGCGAAGAAAGGAAGAGGTTTTAGCAGAAATCGCGAAGAGAAAAGAAGGATCACCAACAAAAGAGGGCATAGAGTTCCTTATCAATGTCTCTGCTACTTGTCTAAATAGTGAGGAGGTAATGGTTCACATCACTGTTTTGAACAAGAACATTAGTCTCCCTCTCTCTAAGTTTTTGAAGGTTTTGGAGGGAGAGGGGCTTCAACTAATGAATGCCTCAACTTTAACAAGCTTTGGAGATAAAACATTTTACAACCTCCATTTTCAGGTATGTGCTCCAAATTGTTGTAATTTTCGCTATTTGTACTATGTGTGATTCATTTTTAAATACTTAGTAGGTGTTATTTGGACAGATTAAGAGATGCACAGGAATGGAGGGTCAGATCTTCTGTGAGCATCTTGTGAAAGTTATTAAAGAGAAAGGAAGGGATGATTACTCCAGCATACAGTGATTAATGTTTGGGCTAATTTTCGAAGGCGAAAAAGTTGTTTATAGGAGAATTTCCGGATTAACGATATGATTAACAAAATTATCGTACATTATGGGCTTGGAAAGATCTTAGTTTTTCTACTTTTTTGAGGAATTCAATTCTGGAATTTTGGTGTGTTGATTCATGCGGACTCCTGAAAAACATTATCTGGATAATTAATCCACCACAATGTTTGTAATTAAGATAAATGCATTTGATGACAAG

>Aco011121; *Ac*bHLH34
AGAGAGAGAGAGAGAGAGAGAGAGAGAGAGAGATGGTCTCTAGGGAGCACAAGAAAGCTGGTCTACATGAGAAGCTACAGTTGCTTCGTTCACTTACTAATTCTCATGCAGTAATTTGCTAAATTTCTCATGCTTTTTTGTGGTCCAGTTCAATGCTCTGGGTTTAACTTTATATATATATATATATAGATCATTTGCTATCTAATTAATTTATTGGTGATGTTCCCTAACTTTTTTGTTTTCAATTTTTTTTTTTTTCTTTCACAAATTTTCTCCATTATAAACATATATGACTTTTCATTTAACTTCAATACATGACTTCTATTTTGATGGTAGGAAACATTGTGATTCTCTGTAAATTCATTAGATAAGGCCTTAGTGTGCTATCGATATTCATGATATAATTTTTTCCACACAATTTCCAGTGAAATCGCATAAAATATTCTACATTGATCACCCAAGACATTATTTTAGTAATTTATTATGATCAAATGGCAGTGTATATACAATGGATACTTAATCTTTGACTGGTTGACAAGTTCTAGAATTTTTATCCCCTTTAATCAGTCCATTCCAACTGATATTAGTGTGACAAAAAGAATCATCCAGCTTTGTATGTCCTCGATTAATTAATATACACATATTAATTAGAGTTAAGCCCATGGCTTTTCTTGAACAATTGTATATTAGTGTGTGTATATATCTCACACATCTAAATTTTTATCTTCATTTGCGACAGCTAAATAAAGCTTCGATCATTCTAGATGCGACAAAGTACATTGAAGAGTTGAAGGAGAAGGTTGTGAAGCTGAATCAAGAGGTCGCATGCGCACAAAACATCGTCATAGAGGATCCCTTGCCTATGGTACATATATATTTGTTTTATTGGGTATTGATCATCTTTTTGTTGAAGTTTTGCTAGCTTAATTATGTTCTCGCACCTTTTGCTTAGATCTAAATCATGATTAATTAAGCAGGTCACAGTTGAAACCCTGGAGAGGGGATTCCTCATCAATGTATTCTCAGATAAGAGCTCCCCAGGATTGCTTGTCTCTATACTAGAGGCTTTTGAAGAGCTCGGACTCGAAGTAATGGAAGCTAGGGCTTCTTGTACCGACACATTTCGCCTAGAAGCAGTTGGAGCAGAAGTAAGCAAAAAATCCACCATCCCTTCATATGTTTGATTCTCTCAATTTATGCGACAATAATAATAGGGCAAAAAATTGCAACCTGGTTAATTATTCCTATATATATATCAACCAATTGATCTTCTCCAAAAAACCAAAGAAGAAGAAGAAGTTAAACAAAACAAAACAACAGTAATTCTACTAAAATTAATTACAAGGGAAAAAGTCATGTCAATTCAATTTGAACAAGCTAAAATGCAACTCTATTTTCTTAATCCTGTCAAATAAAAAAAAAAAACAAAGAAGAAGTTATTTTACTCAAACGAGTTACATTAATACAAACTAAGATATTTCACTAAAAATAAAAGGGGAAAAAATGCAAATATAACAATTAATTTGTAAAGATTATCTAGTCAATAATTAATATATATGCTTAGATTAACTTAAAGTTTACACACGTACATAGAATGCACTGCATTGTTTTTGTTTAGAAAATTAATTTAACCATAGGGTGGATGTACATGTGATTAATTTCTATGCGAATAATGATATATTGTTACAAATATATTTGTGCATGTATTGCAAATATTGCATATATATATTGATATCAATTAATGTTCGATCTCCACGTATCAATTTATAATAATGGCTAAAAAAGATCATACTACTATGGCATATATTTCTGATTCTCGAGTTAATTTATTGTAATTATTATCAACACTATTTTGCATATATGTTAATTATGCTGTACCCTTAATTATCCTCGCTCCTTAATTATCACATATACTTGCTTTGAATATAATTATGCTGTACCCTTAATTTGTTGTAGTGACCAAACATTTCACTTAACTTGTATTTTCATTAAAGTATTTTTTGATTGACTGATAATTAACTACACCATACAAGCAATTGTTGAGAACTTTAAAATTCAACAATTTGCCTCATTCAACCTCGATCACACGATCCGCACTAATATACGTCTACTAAATTATGTAGACACATACCATTCCTCTTAAAATCATGATATATTAAATCTTACTAATCTCAACTTTACTTTATGTTGCAGAACCTAGCTGAAGGGGTCGATGCACACGTCGTGAAGCAAGCGGTGTTGCAGTCGATTAAAAATTGCTCTGGAAATTAAGTTAGTTGTAGTACTAGAATAAATATTTGTTAATTA

>Aco021988; *Ac*bHLH35
ATGTATCATCACCATCCCGAGTTATATCCACCCCATGAGCACATCCCCATGGAAGGATCATCGTCAGAATTCATTGGCCCTTCTCACACCGCCACGATGAATTTCTCCGACTTGGCGCTCATGGGGAGTGGCCAAAACAGCAGCAGCAACAGCAGCAACGGCACCTCCGCCGCTGCGGCCGAACTCGACTTCCACCGCCAGTTCGGTCTCGATGTTGTCGGGAAGCCGGAAATGGCTCCCCATCTCATGCACTTCCCCGAAAACCTCTTCTTTGCCGAGATCCCGCCGGAAACTGCTTCTGTCGGCTCGACCGTCTTGTACGACCCGTCGGTGCAGTTAGATTTGGTTCATCACCAGCCATGTCAAGTGAGAGAGATATATACCGCTAATAATATTAACAACAACGATGATGTTTCATTACCACAAGATTATTCTGGGTTTATACATGGGTCTAGTGTTAATGGCCACTTTGTTGGTGGTGTAATGGAGGAAGTTTATGGGCTAGGGCAGCAGCTTGAGAGCCCACACCTTAACACAAGAAGGCAAAAAGGTGGGATGAGGCAAAAGGGGTTAAGCTTTAATGGGGTGGAAAAGAAGGAGAAGCAAAGGAGAGAAAGGCTTAGTGAGAAGTATGAACTGCTCAAATCACTCATCCCTAACAGAACCAAGGTATAAATAATTAATTTACCCTATTATTCACCACAGCAAATATTTTATTTATTAATTAATTTGTTCATGGCAATACATCTATGAAGCATATATTGATTAGTAGTATTTAGTGTCAAACTATTTATCTCCTACCTTCAACCACTTTTTCAAACCTAAATGCCTCCTTAATTATTGGTTGTTAATAAATTGGACACTGCTTTAAGTTTCTGGTTGAGCTTAATTGGTTTCATTTTTATTTGTTATCTGTTAGCTGTTAGAAGATGCTTAATTTATAGAATTATTTTTGTGTGTGCAGTTTCTTGGTTTTTGCAGTGTTTGTTTTACTTTTTGCTGGTACCTAGGGTTCTTGTTTACATTGTTTTTGCACATATCAGCATAATTTTTTACATCTTTTGGGTAGTGTACATGCGGAGTTTTAATTTTGACAGTGTATTTTGGGCTAGTTACACCCACTTTGGCATAAAAAATATGTTCAAAAAATTGCATAACTTTGGTATTAATTGCTAGTACATGAGTACATCTGTACATGTGAGTTTGTCTATATGTTTCTTCGCGCGGAAAAGGATGATCGAGCGACGATCATCTCCGACACGATGGACTACATCAGAGAATTAGGTCGGACGGTGAACGAGCTCAAGCTACTGGTCGAGAAGAAGAGGCGCAAAAAGGAGAGGGGAAAGGAGGTCTTAATTGGAGAGGAGTTAGTAGGTGACATGGAGAGCTCTTCAGTGAAGCCTTTTATAGATGAGGGAGAACATCATGCATCCAACGGGTCTCTAAGGAGTTCATGGCTCCAACGGAAGTCGAAAGAGACCTTCGTCGACGTTCGGATCGTAGAGGATGAAGTGACTATCAAGATCACCCAAAGGAAGAGGATGATCAGTTGCTTGCTAACCGCTTCGAGAATTCTCGACGAGCTTCAGCTCGAGCTTCTGCACTTGTCTGGGGGAATCATAGGAGACTGCCACATTTACATGTTCAACACTAAGGTCAGTGTACCGTATATCATCTTTCTCAATTATCATAATTTTTATTTTTTTTTAATTTATTTTGGTTTAATTAATGGAGAGTGCTTTAAATTGACGTTGTTATAAACAGATCCCCGAAGGATCTTCAGTGTATGCAAGTGCAGTTGCAAAGAAGCTGATTGAAGTCATGGATGTGCAATTTCCACCTCAGACCTGTTAA

>Aco022096; *Ac*bHLH36
GATGATGATGATGATGATGATGATGATGATGATAATTTGGGCGGTGAGGTTTGATAGGGTGCATACTCTGTACGGTTCATGTTGCTTTTCCTGATGTTCGGCTGCGGAAGTTGTTGTCCGACGCGTTTGGGAGAAGTAGAAGCCCCCAAGTGGCCTGTTTCTCTCTGTTTTTCGACTCCTCTTGCGATTTTTTGGTTCTTTTGGTGATGGGTTTCGGAGATTGTCCCTAAAGGAGGTCGCTTTATAACGCTGTTCCCTTCATTCATCCGGTTCAGAAGCTTTATGGTTCCGGATGAAGCGTTCCTGTAAGCGGTGTTTATGTTTGGAGCCCATTTGCCATACCTGGATATCATTTTGTTGATCTTATTAGTGATTTCTCTGTACCTATGAGTCACTATATATAAGCTGCAACTTTTTTAGTGATAAGAATAAGGTGCTACTAGTACTCCACTCCCCATTTGTTTTTAAAGAATCTAACTTTTTGTTTCTGTTAAAATGGTTTAGATTGAACGAAAAGGGGCAGCTTTGCGAAAAGGAATGCCCACTTTCTACTCTGTTTCAGAAATTTTTGCTGAGGTCAAGCACACTGGTGTGGATAGAACGCGGCCAGTTTTAAGGATTTTTGCGAATTAAACATTCAAAATTTTCCTTTCAGGTCCCTGTCTATTTCCGGCTCTTTTCGGGTAAAAATTTTTATCTCCGTTAAGCTGTGCCACTTTCTGACTGAATTCCATCGGGCAGCAGCAGCTAAAGTAAAGCTCGATCCTACTCGTCAATACAAAAGATGACCAACAATAGCTCGTCCGATCGTTCATCCTTCATGTGAGTACAATTCGCGCATTTGTGCCGTCTTTCCATTTATCTTGTTCTTCCGTAATCCTATTCTTAGATCGTATAAATATATATATATATATATATATTATTGTTTTCTGCGGAGTTACAGGCCTGATAATGACTTTGTCGAGCTGTTGTGGGAGGACGGTCAGATCGTGATGCAGGGGCAGTCCAGCAGGCCCAAAAGAAGCTTTGTTCCCACTCCCTTCAACCCCTCCTACAACAACAGATTCCAAGACAAGGATATCAAAAATGAGATCTTGCCGAAGCAGCTCAATCATTTCGAATCAGGCGATCCTACGGTCCGTCATGACTTCTTCACTTCTGATCCTCTCAATGATAAAGATGACGACGATGATGATTCTGTCCCGTGGATCAACTACCCCATGGTTGATGACTCTCTGTCTAACGATTTCTGCTCGGAGTTTCTAGCTGAATTCTCAGGGCTTAATCCGAACAATGTATCTATCTCTCACAATACCGACCAGATTGTTAGAGGTTCGAGCAGCATGGAGAACAATCATGCTCCTTCGAAAGCTGTTATAGGAGGGTCAGGTGGTTTTACAAGTAGGAGCGGCCAGCTACTCGAGTCGTCTCAGCAACACCTGAACTCAGGTCATGCTGTGAAGTCCAAAGCAACTGATTTTGGCACCGCAAGTAGCATTGTGACCCACTCTAGAATGCCGCAACCGAGCGGCACTTCGCCTCTGCTGAACTTCTCCCATTTCTCAAGGCCCGTTTCTCTCGCCAAAGCCAATCTAGAGAGCATGGAGAGATTGAGAAGCAATGAGAAGGCATTCACTGCACCAACTAGCTCCAATCCTCCAGAGTCTACATTAATCCAATCTACAGGTGGCTTTAAAGAGTTGAGACCATCATCAGCAAATACTCCAAAAGAAATGGTCTCACTCGAGCATACGCAGGCTCTTTGTCAGCAAGAGATGCCCAGAAAAAATAATAATCACACTGCAGTGGCGAATAGTAATTGCATTAATCAGCAGGGTTCAGGTTTTGCACCTCAAAAGGGTCCTGAAACAATTGTTGCATCTTCCTCTGTATGTTCTGGCAATAGTGCTGGGGCGGCATCTGATGATCCTAAACATGGATTGAAGAGGAAGGATTGCGAGGGAGAGGATTCTGGTAATCATAGCGAAGTAAGTGACAAGAAATTAGAAAATATACTGTATTTAATTGATCAACTTTTTCTTTATCGACTATGTGCTTCAAAATTCTTTGATGTCTATATAGGATGTTGAAGATGAATCTGTGGGGCTAAAAAGACCTGCTAATGTTCGAGCCACAAAGGCTAAGAGGACTCGTGCTGCTGAAGTGCATAATTTGTCAGAAAGGGTGAGTGCATAATGCAATTAACTACGTTCCTACTAATTCTTCTTTCTCTTAGTCTGGTGTGGAAAGCTCTAACAACTGGTTTCTTCGCTGACACGCAGCGGCGTAGGGACAGAATCAATGAGAAGATGCGTGCATTGCAAGAACTAATACCAAACTGTAACAAGGTATCCTTATGTTTCAGCAGCCTCTTACTTGTCAAAGTTTCTTGTGCAGCTTGGGATATGGTAAAGATTTTGCCTAACATCAAATACACTTTATAAGGTGGATAAAGCTTCAATGCTCGATGAGGCGATTGAGTATCTCAAAACCCTTCAGCTTCAAGTTCAGGTAATATCTTTGCCTTCCATTAAGAAATGTCTTGCCTAGCTTGTTTATAAATCTATCTTCCCTAACTAAAATTTTGTATTCTTCTTACACAGATGATGTCAATGGGCGGTAGTCTATGCATGCCTCCGATGATGCTACCTCCTGGGATGCAACAGATGCACTTGCCATCCATGGCTCCTCATTATGCCCAAATGGGTGTTGGCATGGGACTGAACGTTAGATTGGGATACGGAATAGGTCCGCTTGACATGAGTAGTTCTCCAAATTGCCCTTTAATTCCAGTCCCACCGATACACTGTGGGACACAGTTCCCTTGCCCCACAGTTCCAGGAGCTCAAGTTCGGCCTGCAATGGCTGGACCTGGCAATCTCCCAATGTTTGCAATACCTGGACTGGGGGCAATTCCATCCGCGGTGCCCCGTATTGTGCCACAATTAGGTTCTTTTTCTGGCCTTGCTGTAATGGCTAATCCAGTACCTGTACCTGCATCAGATACAACTACTCCATTGAACTGTAAAGAACATCGGGAGCATAGCATGAACTTGGAGCGTAAAAAGAGTTCCAGTGATTCGCAGGTTCAGACTCCAGCTCGTTGCTTGCTCATTTTGCTTCAACTTGATTTACTTATACTGAAACCAATCTCTTAAATTAATTTGTTTTTCATCATCTGTGGACCATCTTCAGTGTTTCTGTACAAATGCTTGTATGATCTTGTGTTCTGTTTTCCCATATTAATAATCAAATAATGGTTTTCATACTGCTGGGGACAGAAAACACCTTTTGCACCATCTGCCTTGGCTCAAAGTAATCAGACGCTGCACAGTAGTGGAAGTGGAGGTGCTAATTCCGGTAACCAAAATGGAACTTGACTAACCAGGGTAACAAAGTAAGCGGCATTCAAATTGTGCTATTGGGTTATACTTAACTAAATCATGCCATGCCCCGTCAATATATTTCCTCTGTTGCAGGTTATGGTTGAGGCATCCTCCTTTTGATGGTTTTTGTGCAGCTTGTCAAAGTTGCACACTATTTGGGAGAATTTTGTATACTAGACTGCTCTCTGAACTTTGAATGGCCATATGATCTCCACGAACACGATGGCCTGGGCTTTGACTTCTGCGCCATTTGCGCCAAAGCTGTGCAAGCAGAGGATTTTGGGATGCTTGGA

>Aco023468; *Ac*bHLH37
GAGAGAGAGAGAGAGAGAGAGAGAGAGAGGATTATGGCTGATGATCTCCATACAGCAGGGATATGCAGTGGAAGAGGGAGCTGGTGGATGAACTCAGCGAGGAGCAGCGCCGGACTCGATGGTCAGATTGCGATCTCCTCGACGTCGATAGCCGACATCGATGCAAGAGGGTCGACCTTCAGTTGGTCGGCGGGCGAGAGCAAGTCGCGGTCCTGCGAAGAGTCCACTGGCTCGGCCTCTGCCGGAAGCTCAATAACCTTCCAAGACACCCACTTAAATCCCTCCATGAACTGGAATGAAACCCTACTGTAAGTGATCTTCTTTCTTTGTATATATGCGTTTAGAGAGATATATACATATACATATATATAGAGAGAGAGATATATAGGGTTTTTCTTTTTTTTTCCTTTGAGAAAATTATCTATATATTTTGATTGATGGGTTCGGCAGATTCCTACTTCACCTTTACTGAAAATAACAGGTAAGGTGTATGCGAAATTTAACAAAAAGATTTAAGTGTAAGATTCAATTTTGTAGAATTGCACTTGCCAAATCTACATGATATGTTGCATGCTTAACATATATATATGAACTCTTATTTCCGTGAATTTTATATATGATGGAGAAGCAGCGGCGGAAAAGCTGATACAAGAATTCAAGCCATGCACCAAGATGATCTAAGCAAAAGGCCCTATTGGCATGACGGACTGTTGATAGACTCGTATCGAGCTCACGTTGGTGTCGAAGAATCCTCCACAAATTTATTCAAGGAGATTAACCATGGAAAAAATTTCCTCCTGGACAAACATCTGTTGAATTCAAGTAACGAAGCTTCCGCTTCCTTTGGAAGCCCTTCTAGATTGCTCCAAACCTTTATCACTAGCGAGGCGAATCCTCCACTTTCGATTTATGGAAACCCATATCTACATCATCAGAGTTCGACGGCTACGGCTTCTCATGAGAGCTTAAGTGAGCTTTTGCAGCCCTCATGGACAAAATTCTCTCAGTTGGTTAAGCCTTCACCATTAAAGAATCAGTTGCAGTTCTCCAATAGCACTCCCTTTTGGAACCCTTCTTCTACTGCTATGAATGAAGTGAGTTCAGGGTTTTACCCCTCCGTGCCTTCGCAGTTCATCTCACAGCAGAAACCAAGTTACAGCAATCTCATAGTGAAGGAGAAATCTTTGGTACCGTATTCTTTCTTTTTTTTTTTCATGATTTTTAGGGTTTTACTTGTGTTCAACTCAATTTAGTGATGCAACTCAGCAACTCACAATCATGTATGTAGTTAAATTCGGAACAAGTTCAAGACTCATGCTCATCTGCAAAAAAGAAAAGCAGCAGTGGTGATGATCATCAACCCACGTTTAAAAAGCCTCGAATCGAGACCCCGTCGCCATTACCGACCTTTAAAGTACGACACTATCTGCTTGAGATCTTTATAATCTATAGCAGTTTGATATCTCACTTTCTTCTTTGCTTCTTTCTGTTTAAGCTTTCATGATAGCTTTTTTGAGCTCATTGTTTGGATATGTAGGTGAGGAAAGAGAAATTAGGGGACAGAATCACTGCGCTCCAACAGCTGGTTTCTCCTTTTGGAAAGGTATCTCATATATCCTTTAAATGATCACTCTCACTCTCTCTCTCTCTCTCTCTCTCTCTCTCTCTCTCTCTCTATATATATATATATATAAGAGATAATTTGTTGGCTTATGTTCTAGATCCTTTTGTGTTTGCAATTAAATGTTTATTAAGCTTCCAATATGTATAGAACTGAATCTTTCCTTCCAACTTTATTATTTCAGACTGATACAGCATCAGTTCTTCATGAAGCCTTTGAGTATATTAAGTTCCTTCATGAACAAGTGAGTGTAAGTATCTCTACTAGTTTTATATATATATATATATATATATATATAGAAAAAATAGCCAATATCGAATTGTATTTAATAATATCTGTTTCTTAACTGCAGGTTTTGAGCACACCATATATGAAAAATGAGGATCTCACGCAGCACCAGCAGGTACAAAGCTCTTCAACCTGCACTCACTTAACTACTTACTAATATAAATGTTAGTTTCATACACAATGAAGAACTTGGCTATAAAAGTTAATGTTTTAGGGTTTGGAGAGGTTGAACGATGGCGAGGGACCGAAACAAGACCTTAGAAGCCGAGGTTTGTGTTTGGTTCCGATTACGAGCACTTATCCGGTCGCTACCGAGACTGTCGCAGAATTCTGGCACCCTACCTTTGGAGGAAGCTTCAGGTAGTGGGGTAAGGGCTCTGAGGAAAAAGATTGGATTACAACTTTTTTATTATAAAGAAGCAAAATTCAATGGTTGTTGTTAAAGAAGTGGGAGATCAATCAAGAGTGACAGAAA

>Aco023519; *Ac*bHLH38
ATGTATCATCACCATCCCGAGTTATATCCACCCCATGAGCACATCCCCATGGAAGGATCATCGTCAGAATTCATTGGCCCTTCTCACACCGCCACGATGAATTTCTCCGACTTGGCGCTCATGGGGAGTGGCCAAAACAGCAGCAGCAACAGCAGCAACGGCACCTCCGCCGCTGCGGCCGAACTCGACTTCCACCGCCAGTTCGGTCTCGATGTTGTCGGGAAGCCGGAAATGGCTCCCCATCTCATGCACTTCCCCGAAAGCCTCTTCTTTGCCGAGATCCCGCCGGAAACTGCTTCTGTCGGCTCGACCGTCTTGTACGACCCGTCGGTGCAGTTAGATTTGGTTCATCACCAGCCATGTCAAGTGAGAGAGATATATACCGCTAATAATATTAACAACAACGATGATGTTTCATTACCACAAGATTATTCTGGGTTTATAAATGGGTCTAGTGTTAATGGCCACTTTGTTGGTGGTGTAATGGAGGAAGTTTATGGGCTAGGGCAGCAGCTTGAGAGCCCACACCTTAACACAAGAAGGCAAAAAGGTGGAATGAGGCAAAAGGGGTTAAGCTTTAATGGGGTGGAAAAGAAGGAGAAGCAAAGGAGAGAAAGGCTTAGTGAGAAGTATGAACTGCTCAAATCACTCATCCCTAACAGAACCAAGGTATAAATAATTAATTTACCCTATTATTCACCACAGCAAATATTTTATTTATTAATTAATTTGTTCATGGCAATACATCTATGAAGCATATATTGATTAGTAGTATTTAGTGTCAAACTATTTATCTCCTACCTTCAACCACTTTTTCAAACCTAAATGCCTCCTTAATTATTGGTTGTTAATAAATTGGACACTGCTTTAAGTTTCTGGTTGAGCTTAATTGGTTTCATTTTTATTTGTTATCTGTTAGCTGTTAGAAGATGCTTAATTTATAGAATTATTTTTGTGTGTGCGGTTTCTTGGTTTTTGCGGTGTTTGTTTTACTTTTTGCTGGTACCTAGGGTTCTTGTTTACATTGTTTTTGCACATATCAGCATAATTTTTTACATCTTTTGGGTAGTGTACATGCGGAGTTTTAATTTTGACAGTGTATTTTGGGCTAGTTACACCCACTTTGGCATAAAAAATATGTTCAAAAAATAGCATAACTTTGGTATTAATTGCTAGTACATGAGTACATCTGTACATGTGAGTTTGTCTATATGTTTCTTCGCGCGGAAAAGGATGATCGAGCGACGATCATCTCCGACACGATGGACTACATCAGAGAATTAGGTCGGACGGTGAACGAGCTCAAGCTACTGGTCGAGAAGAAGAGGCGCAAAAAGGAGAGGGGAAAGGAGGTCTTAATTGGAGAGGAGTTAGTAGGTGACATGGAGAGCTCTTCAGTGAAGCCTTTTATAGATGAGGGAGAACATCATGCATCCAACGGGTCTCTAAGGAGTTCATGGCTCCAACGGAAGTCGAAAGAGACCTTCGTCGACGTTCGGATCGTAGAGGATGAAGTGACTATCAAGATCACCCAAAGGAAGAGGATGATCAGTTGCTTGCTAACCGCTTCGAGAATTCTCGACGAGCTTCAGCTCGAGCTTCTGCACTTGTCTGGGGGAATCATAGGAGACTGCCACATTTACATGTTCAACACTAAGGTTAGTGTACCGTATATCATCTTTCTCAATTATCATAATTTTTATTTTTTTTTTAATTTATTTTGGGTTAATTAATGGAGAGTGCTTTAAATTGACGTTGTTATAAACAGATCCCCGAAGGATCTTCAGTGTATGCAAGTGCAGTTGCAAAGAAGCTGATTGAAGTCATGGATGTGCAATTTCCACCTCAGACCTGTTAA

>Aco004377; *Ac*bHLH39
ATGAGAGACAACATTTCCGACCTTTTCGAGGATCCCCCGGAGTTCAGATCCGCAGACTTCGCTGGAGCGGCATCTCCGGACGACCTCTTCAGCTTGCTGGAAACTTTGGAAGACAGTAGAAAAGAAGTCCCCCCATTCAACCCGTTCGAGAAAGCGGCAGCTTCTGCGGTGCCTCAGAAACCTCTTCTCTCGAGTGTTCGGCGCCACGAGACCACGGACGACGCCGAAGAAGCGAATGACCCGGCTCGGAAGAAGCACAAGGCATCGGCCGCTGAAGCGGCTGCCCAGGAATCCGAGGCTGCAGTGGCCGCTGCACAAGACGGCGGGCAGCACAAGATGTCGCACATCACGGTGGAGCGCAACCGGCGGAAGCAGATGAACGAGCACCTCACGGTGCTGCGCTCACTGATGCCGTGCTTCTACGTCAAGCGAGTTAAGATCGAATCCGACCAATTCGCGATACGAAGATCAATAAAGTATATTTTTGCATGGTACTATTAACTCCGTTTACTATTTCGTCGTCTCAGGGAGATCAAGCATCCATCATAGGAGGAGTAGTCGACTACATCAAGGAGCTGCAACAAGTGTTGCAATCCTTAGAAGCCAAGAAGCAGAGGAAAGTATACAGCGAAGTGCTCAGCCCGCGACCCGCCATTTCGAGCCCTCGCCCCTCGGTGAGCCCACGGCCACTTCCGCCGCCGCTAAGCCCGAGAGTGGGCTTGCCGATAAGCCCGCGAACGCCCCAGCCGGGAAGCCCATACAAGCCCAGAATTGTGCAGCAGGGTCTGTACTTACCAGCCACTATGATGCTACCATCTATGGAATCCTCTCCTTCGTCGGAGCACACTTTGGCCGACCTCGCAGCGAACTCGAAGTCACCTGTCGCAGAGGTGGAGGTGAAGTTCACGGGCCCCAACGTTCTCCTCAAGACGGTGTCGCATAGGATTCCCGGGCAGGCTGTTAAGATAATCGCGGCGCTCGAGAGCCTCGCTCTAGAGGTGCTCCATGTGAACATCAGCACCATTGATGATACGATGCTCAACTCATTCACCATCAAGGTAATTAAGTGTTACTCAATGAACTATTGTATATACATTCCATATATCTTCTTTCTTCTTTTGCAATATATAAAGTTTGTCGCTTAACTTCCTTGTTAGTTAGGTTAAGTACTTAAAAAGAATTTTCACACAATATATACAAACTACTTCTATATGCCTTTAACAATCAAGGTAGTACTCCTCAAGTAATGAGCCATACCACATGACTCCTCATCTAACTCTAAAATATAACTTCTTTTCTACAATGTAAGATTTATCATTTTTTTTGAACTTTCTTGTTATATAGTTGGGTTACTTCTTTTAAGTAATTTTCACAAAATACATTCTAGTTCTATCATTCTTCGTCTTTTTAAAATCTTTTTTTCCTTTCTATTAATTTTTCTTCCTTCCTGGTCAAGAGATGAATAATAACATGACCTTTTTCCTTTTTTCAAAAAAAAAACATGACCTTATAATTTCCACATTCAAGAAAACATTCCTGCCAAAGCCCAGGAGACAATCACACAGTTATGGTTCAAATAAATAGCTCTAGTGTGCATTAATTTAACACTTGCCATGTACACTTGAGGGTCCAAAGCATATATACATATCTCTACGTCCTTTTCAGAGAGAGAGAGAAAGAGAGAGAGAGAGAGCACTAGGCATGTGGTGGGTTTAATTTATATACTGTTTAAGCTACTCAACTTACTGTTTTCGTCTACTTTCTCAGTTTATGTACTTCTCAGTCCTGAACAACCCTAGTAAATCTAAGATAAGTAGATGTGCAGTGTAGTAGTAGTAGTGTATAATATATAAATGATAACTTGGTTGCCCAATGGAGATAATAGTCCAGATGTACAGTATACTGATAACTTGGCTACTTCAATGATTACTAGGCTGTAAATTTACAGAACAGCTAGCAATAAGTTAGTTTTTTGTAGACTTTATGCAACCTAGAAGTTTTAGATAACTCATTTACTGGAATGATACTTGAATGTTTATAAATACACATATAGGTCATGACATGTTGTTCTGTAGCTCTAGCATGCAAGTTGCCACTGCCAAAATTAAATGATTAACTAATGAAGATCACAGAGGTGTGTATATTGTTTCCGGGCTTTCAAAGTTAAATGATTAACATAATGTTGGTTCTTGAAGTGTCCAACAAAACAGTTTAGCATGTATGTATCTATGTATTATGCATCCGACTCTTTCTCTTCCAATAACATGACATTTTTTTTTAACATGCATGGCACTGCACAAGTCTTTTTGTTACATTTCATGTTGCTTTCTTTTTTCTTTTTCTTTTTTTCCTTAGTTTTTGTTGTAAATCTACTTAATTTGTTGGAATCATTAGAGTTGCATGTTACTAATGTCGTTAATTTCCTTTCAACTATCTTTCACAGATAGGAATAGAGTGTGAGCTTAGTGCGGAAGAACTTGTGCAAGAAATCCAGCAGACGTTTTCTTAG

>Aco004559; *Ac*bHLH40
CTCCCTCTCTTTCCCCCAATTTTTCCCCTAAAAAAGGCTCCTCTTTTCCCCCTCAATTCTCACTCTAAACAGTGGATCATCATCTGGGTATTTGACATCTTTGCTAAATTTAGCTCTTTTGCTCCCAATTTGTGCCCAAATCTCATAAAAAAAGAGAGATCTTTCAACACCCCCAGCCAATTTCAGCAGCTATTTCTTGTCAGAAAATACTAAAGGAGAGATCTTTCCCCCCATTTCTCCCAAAAATTCCTCTTTTCCCCTTCAAATTCTCACTCCTCAAACTGAATCATCATCTGGGTATTTAGCTCTCTTTTTTCGAAAAAAGAAGAGATCTTTGAACACCCTCTACCAATTTTAGGACCTGAAATTCTCTGATCCACGGAGATGAACTGCGGCGACCAATTGCCCCAATTCTTCCTCAACCTCAACTGTGATCATGATCTTCTCGGCCAATTCGGCGCCGAGATCTCGTCGCAGCACCCCTACATTGGATCAATTTCTCCCAACCTGAATCCATCGCCGCCGCCGCCGCTGACGATGACCCATTTGGATCAGCTTTTCCCCGACCCGGGCTTCGCCGAGAGGGCGGCGAGGCTTTCGAGCTTCGATGCCCGGAACTACCGCGCATTTGCGAGCCGGTTCGGGCTCCCGGAGATCGGCAAGGCGCCGATCGATGCGGAGGAGTCGTCGGTATCCGATCCACCCTCGGCCGAGTGCTTCCATTCTCCCTCGGAGAGCAATCCCAAGAAGCGCAAAGCAGCCGCAGCAAAAAGCAGAGGAAAAGAGGCACCTTTGGTGAACTCACCCAAGAGCTCTCTCAATGTATTAAAAAGTTTCCTCCACATGTTTCTTTTTCATTTTAACTAGTTTGCAAGAGCCATTCATTCTATTATGCGGAATTGAGGAGTGCGAATCGCCGATTTGCAGGTGTTGGAGGAGAAGGGCTTAGAGAGAAAGAGATGTAGACTGGGGGAGAGTGATCAAAAGATAGAAGAAGAGGATTCGATTAAACATAAAGAGGCGCAAAATGGGGCTCAGAAACAGGGCAAGGAGAAGAATAATGCGAAGCCCGCGGAACCGCCTAAAGATTACATCCATGTGAGAGCAAGAAGGGGCCAAGCAACAGATAGCCACAGCCTCGCGGAGAGGGTACAATTTTGATCTCCCTTATGTTTGTCTCAGATTCCGCATTTTTCCTATGTTTATTCGGCTGGTTGCTGAACTTATTATGAAGTTTCATATTTGGTCATCGAATCTCAATTCGTTTCAAATTACCACATGAACTTTTAAGTTTCTATCACATGGCATGATTCTATCAAACTTCACTAAAATTCTCTCTCGGATGGATGATGTGACAGCTGATTGTATGCGCCGTAGCCCTTTTTATTATGTAAACTGAACGGAGTTTAACGAAGCAACCCAAATTGGAACAAAATATTAAAGTTTAGTGCCTTAATTACGACAAAATAAACTTTGGTCTTTTTCCTCCTCCCCCCCGCCCCCTTTGTATCTTTACTTAGGAATGTTCTACTTGTGTTACCAGGTTAGAAGAGAAAAGATAAGCCAGAGAATGAAATTACTCCAGGATTTGGTGCCAGGATGCAATAAGGTAACATCTAGAATTCAAGAAGAGAAACTAAACCTTGCTCGAAAATTGCTACTTATATCTACTTGAGGAATTGAATTTGACTTTGGATTTTCCCTTTTTGTTTTCCGGGCATGTAGGTGACGGGAAAAGCCGTTATGCTTGATGAGATCATAAATTATGTGCAATCTCTCCAACGGCAAGTCGAGGTATACACATTGTTGCAGAGCCGAAGTCGGCGTTTTATGATGTGAGAAATTTTATTTTGGATTCTTATGAATTTAAGGTTTTATTTTATTATTGCAGTTTCTTTCGATGAAGCTGGCCACTGTGAATCCCCAGCTAGACTTCACCAACTTGCCGAATCTTCTCTCCAAAGATGTGAGCTTGAAACACTATTTCAACATTCTCTTTGTTGTATTTTTTTTTTTCTTTTGTTTAAATGTAGTGCGCCTTATATTCAGATCCTTTTGTTGAATCCAGATGCATCAAAATACCGGTCCTTTTCTGAATCCAGTTTTCCCGTTGGAAACTTCGGGTGCGGCGTTTTCCTATACCGACCATCCTCATCAGGAGGGGAACATTCTCCACTCCATGAACCAGTTAGACCCTTCT
CTTTCTCAAAGTATGAGCCCACACCAACTCCTTAACGGAATCGGCAATGCTTCTTTACAGGTAATAAATTATGGTCGCCGACATTTAGTGTAATCCAATTTGATTCTTGTAGAAGAGAATACTCAATTGAAAAGCAATTTTAAATTTTCAGGTAGGGAATATGTGGGAGGATGATCTCCAAAGTGTTGTTCAAATGGACATTGCGCAAAATCAGGATGTAGTAGTCTCCTCTCAGAGCTTTCATGCTGTTGGTAGGTACATAATTTTGTGTGCATTTTTGAATCTTATATTCTTTTAAATGAGTATGCTGAAACATGATTTACGCGGTGCAGGTCAATTACAATCATCTCACATGAAAATTGAGCTCTAGTTATCTCAATTGATTTGGAGCTTGATGATTCCTTTGTATATAGCCAGAAGTTGGTCGGCGCCGGAGCGAGAAGCTTTTGAACTAACAATCTATAGTTTCTAAGACAATTTTAATTTCTCGTATGATTCTTTTGATATTTTTCAGAGTTGCAGTGTAACAAGTTAGTACAGTTGAAAAAAGGTGGTGCAAATATTACAAACACCCCTTTTAAGATGTTGAAGAGGGAGAAGAAGAGTGCGAGAGACGTGGAATTATGATGCTGTTTAAATTATATATCTATTTCATCTGATAAAACTCGTCCTATGTTTTCATATCAGAGCTAAGAGATGT
TGTCGGATAAAGATATATATATAGGGTGC

>Aco004647; *Ac*bHLH41
TCTTTGAACATTACATCTCTCTCTCTCTCTCTCTCTCTCTCTCTCTCTCTCATACAAAGAATGGGATTCTTTTCATTCCAACACCAAAACCCTTTCCTCCTAGACTCACCAATTTATGACTACCCAAGTCATGATGAGACCTTCAAGATGCAATTCCTACCAGCTGAACAAGCAGAAGACATGGGGAACAATGCCTCCTCCTCAATTGAGTGTGGCAGCTCCAGTATTGGTGCATCTACCCCATTGATCCCTTCCTCTCCTTCTTCTTCTCTGGTTGCTGTTCCCCATCCTTGTGAAAGAGCAGCTATTATTAAGCCACAGGATAAGAAGAGGAAGAGCAGATGTGGAAGTAGCTTGAGCTCTTCTCAGTCCAAGGTATACTGGTACTAGATTAATCAATTAGGTTCCAATCTAGCTACTCATTTCACTTCATGATATTATTTTGTTCCTGCAGGAATCCAAGGAAAGCAGCAAGGCCAGAAAAGTGAGGAATGAGGAGAATAAGGCCAAGAATGGGGTGAAGAGTGAGGCCAAGTCAAGTGAGGAGCCTCCAAAAGGCTACATTCATGTCAGAGCAAGGAGAGGCCAAGCAACAGATAGCCACAGCCTTGCTGAGAGGGTAAAAGAATGCTACTCACACTTCAATAATCACACAAGTGTCCCTTCTGATCAGTTAATCTTTTTTTTTTTTTTTGAAAAAAAGATATAAATCTCATATATCAGGCTCATAATTAATTAATTATGTACTTCATTGCTCTTTGTAATCAATTATATATTACCATTGTTCTCCTCTACATGACACTGTGTGCAGGTGAGGAGAGAGAGAATAAGTGAGAGGATGAGGATGTTGCAAGGCCTTGTCCCTGGCTGTGACAAGGTATACAATATATTGTAGACTCTTCTTAATTATCTCATCTCTCCCATAATGGGAAAAAAGCACACACATATATACAACAAGTCCCTTGCTATTATTAATTATTTGCTTTTGAAACTATATATTTCTTGCTCATCATGTATCTCTATTTTCACTGTTTTCTGATGTTAGGTCACAGGAAAGGCTCTCATGTTGGATGAGATCATCAACTATGTGCAGTCCCTCCAAAACCAAGTTGAGGTACAAATTAAATCTACTATAATCTTTTATATATATATATATATATATATATATATATATATATATATATATATATATATATATATATATANTATATATATATATATATATACCAATTACATTAAAATTGGATTTTGATCAATGATTAATGGGAATAATTATTTTTGCTCTTCTATGTGACAGTTCCTCTCCATGAAGCTTGCTTCCATGAGTCCCATATTATATGGCCTTGGCTCGGACTTTGATGGCCTTGTGGATCAAGCACAAGTATGACATATATAGGACCCAAAACTCCATTATCTATTTATTTATTGATCTTTCCATATACATATATAATTAATTATTCCTATAGTGTACTAAGAAGTTCATATTCTGAACAGGGTTGCAGCCCAAATTTTGAGAAATACTCAGAGAGCATGTCAGTGACAATACCATGTGTACACCCAGAAACAAGTCCTCTTCAACCTATTGCTTTTGAAGGGGCACTTGGAGACTACCCCATGACAAGCAATTCCACATCCCTTTCACTACAAAACCAAGGGCCCATTTCCTTTTCCCAGGTACTATATACATGTATAACTTATCTTTTAAAACCAAAAAAAAAACAAAAGGTTTTTTTTTTTTTTTCCCCTCCTCAAGATCAAGCAATAAATAAATATTTCAAGATCTTTAAGTGTAAAGTAATTGTATTATTATTTGGGTAAATTGCATGTTTGGTCCTTAATTAAAACTGTAAGGCTAGTGACACTTTAGTTCTATAATTTTAATTTGTTATAATATGTAACTCGAACTTTTCGGACTTGTTGCAATCGAGAGTCTACAACCTAATTTAGTTCACTAAATATTGACGCATCATAGATGTAAGGGAGCGTTTGGTTCGCACTATGAAAGATTACTAGCAATAAAAAGATGTCCGATAATTTTATTCCTATTCATTCTATTACTGAGAATGTGACATTCCTCTGTTTGGTTCGCACGGTTATATTATTTAGTCATGTTATTTAAGTTTACAAAAAATATACAATTAATTTATTTATTTTCTTTAATTAATTTTAGGTAATGCCACAAAAAATTTCAACATCTTTTTTTTAAAAAAAAGAGAGAACATAGGTTAGAGAGAGAGGGTATAATTAAAGAAATAGAAAGAGAAATAGAATCGAGATTAGAGGGAGTGAGAGAGTTGAGATTTTAGAAAAAGAGAGGGAGAGAGAGCTTAGTTTAGAGAGAGAAAAATAAGTTTAGAGAGAGATATGAGGTTAGAGTGTAAAAAAAAATAATACCAACTAGCCGGTGGGTAGGGAGAAAGAAAGAGATTAGAAATATTATGATTACCCCAATCCATTAATATGAGGATACCCACCCTCCCTCAAGGGGATGAGATCAGTACTTTGGGATGAATATCATTCTCAAGAAAATATAATATTACTAACTAGATTACTTAGTGCGTTTAGCCAAACACCGTCATATTTTTTTATTCCTATCGGATTACTAGAAATCTTAAAAAAATAGCACAAACCAAACGCGCCCTAAATGTAATGTGACTTTTCAATTACACAACACTGGTGGTATATTATTTCTACGTCAGAAATATATCAATGTTTAGTCAATTAAATTGTATTATAAAATTTGATTGTAATAATTTTAAAAATTAGAATAAGAAATTACAGTGAGTCAAAATTTGAAGCCCAAAATTATAATTTATCCTTACTATTTGGACACCATTGCAAATATAAGAGTAATACTTCTATACTTTTTTTTTTTAATTACCGTTCATTCACGCGTATTCAACGGCTCAGATTTAGCTTTTTTTAAATGGTGATTTGCAATAGCAGGTCACTTTGGGAGAGGTATCGGTTACCAGGTGGCAGGTACCTCAAAAAAGGGTATTTTTGTCTTTTAATACATGGGCAATTTAGTCATTTTACATCTACTATATTTTCAAAATTTTTATTTTCTCTTTATTTTCTTTTTCTCTCTCTATCTTTCCTTTCATAATTTGGCATTTTATATAAGAAAAATTTCAAATGATTTGACAATATAATCTTTTATTTTTTCTTTTCTCTTTGTTTATTTGTTTAATATTGCAAGAGAGTTTAATTTTAAGAAACTATGCATTAAAATTTCTTTCCATACCTATTGCTTTTTAAATCTTTTTTTAATAAAATAAGAATGAAATTTAGAACATATTGTCTAAAGTAGAACAATTCTATTTTTTTTTTAACTTATTAAATACATTTAAATTATAAATATTTTAACCTACATTCAAAATTTTTTAAATAAGTGATACAAAATTAATGTCTAAACTATATGAATTTAAAAGTTTCTAAAACATATTAAACATGTTGAAAAAAAATTTACCAACATTTCAATTTTTTAAATAGTAATATGAAATTCCTCTAATTTAAATTTCATTTTTAAATTTTAAGTATTTAATTTTAAAATTTAAATTCATCGTATTTAGAAATAAAACTATTTCTAAAATTCCTATAATTTAAATTAATGCATAATTTTAGAAACACTAAAACATTTAAAGTAGATGCCTAAATTAATGCCTAATTAATGCATAAATTTGGTTAATCAATTAATTAATTTCCTAAATTAGTGCAAATTAATGCATAAATTTAGGACCTCAATTACTGGTTTCTTTAAATGTTGTATTTTTTAATTACCAAACTAATCTTGAATAAAATAATTTTTTTACCTGCTAAATATTAAAATTATATTTTTATCCTTTATTAATCAAAGGTTAAGATCTTTTTTAAATTTTTTTTTTTAAAAATACCGAATCATTTCTATAATATCACTACCAGTACCCTAAAAAGTTGTTATCGAAAAACCTATCCATGTACACTGCTGATGATGTAACGAGCAATATACTAGTATCATGCCACGTACAAATTAGCTTGTGTGTACGTTATTATTTATGCACAAGGGTACCAAAGTATTTCTCTTAATAATAAATATGCGGAGCCCATCTCCTTCTCTCATGAGGTTTGCTCATAAATTTACGCAAAAAGTTAACGTTACTAACCTTCGTGCAATTGAGATTAAGGAATTAAGGATGTTTCGAGTGCCTTTATGAGTATAAAATGGCATTTTATCATATATATTAATATGTGGAGATTATTTACAGGACACTGGAAATTTTCTCATGCAAGTGGGTGACCAAAGACAGGGCCTTCTCAACCAAGTGGTGTTCAATGACATGTGCTCGTTTCAGTAGAGAGAGAAAGAAAAGGACCTCTTTTAACATGCAAGAGGTAACTTCAAATGAGTACCATTAATGAAATTAACTTCACCAAATTGATCTTACCATTCATCTTCTTTTTCTCTAATTTTTCTTCTTTTTTTTTTTGTTTTGTTTTTGATGTATAGGCACTCTCCATATATGTAAGAACAAGAGCTGAGACAGTAAATGTGCAGTGGAAGACCAAATAAGACTAGATCACCTCTCACTGCTCAGATCACAAGATTAAGTAGTTGGGTTCAACTTTCCTCTACTAATTTGGTGCAGTATACACCAATGTTCATGTGAAGCAAAACCCTGGAGTAGCTAGATGAGGGAGAGGGAGAAAATGGAATTGAAGAGGAGAGAGCATATGCATGGAAACATATAAACCTCTAATGGATCTTCATTGCTGCTTTATTAGATTTGTCTCATGCACAGATGTAGGGATCCTATATATTCAGGATGTTGCTATTAATTGTTGGCCGCAGTGCCATGATGATGTTAGACTTTTCTTGATGAAGTTATATAATGTTAAAATGACTTATTGTTTTCATAGACCTTCCTGTTGCAATCATCATTTACATTATGAGAGAGAGAGAGAGAGAGAGAGAGAGAGAGAGGAGAGAGATGATATGACTAGCCAATGAAGATATGCATTCTCTGATATCTTTTCTCCCTTTCCAAATTCAACATTTAATTAATCTCTGATGCATGTACTATGCACCCTGAGCTAGGTTTCTATCCAGGTCCTTTGCAGCAGCTATGTGACCATAGCATGTACCTCAGTTTGAGAACATATATATTTCCTATTGTTTTCACTCTTTTTAAGGATTATATATATATTTGGCTAATTTGACCATAGCAGC

>Aco004686; *Ac*bHLH42
GAGCTCGAGCTCGAGCTCGATCGGGATGATGAACCGGTTCGAACCGGACGGGAGGAAAAAACCGAGCCCGATGGGGCCGCCGGCCGGGCTGGCCCGGTTCGGATCGGCCCCCGGGTCGCTCCTCGCGGGGATCGCGGACTCGGTGATCGGCGGCGCGGGGGGAGGGGGTAGGGCGGCGCTCGTCCGCCACAGCAGCTCCCCCGCGGGGCTCCTCTCCCATCTCGGGGTCGATCACGGTGAGTTTTGAGGATCTCAGCTTATTTCCCTGCACCCTGTGCGCACTTGTATCTGTGCACCGTTTAAATATTAGCCGTTCATTTTGCCACTTCTGTCAATCTATATCGTTCACTGAGCTATAGAGTAGGAAGTAATAATCAAAATGGGGAAAAAATTCTACTTTTAAATGGCCTATAGTTGGGAAGATCCCAGTGGGGCCTACTGATTGGGTCATACCTACGTGGCAATCTCCAATTTGCTATTTTAGTCTTGTTTTTCCTTTTGGTAAAAGTATATAGGATTCCCTTTGGGGCATGTTTGTTTGGGTGGAAGTGGGGTGAAAGTGGAGAGGAGGGAAGTGGTTTTCGCTTTAAACGATCACTTTCGTTGTTTTGTTCGTCGTAAAAAAGTTACGGTCAAAACTCGAGTTAACCTCAAACGGTATAATTGACTTCGCTCCATCATAGGCCGAAGTCAATTATGGTGAAGAGTGGTGAAAAAAATAAAATAAAATAAAATAATAAATAAAGCTAGTTATATTTACATATATTTAATTATATTACTAATTATTTTTTTATTCCGTTAATATATCTAAAATATGATAAAAATTGATTAGTTAAGTTTTAATATTTTTTTATTTAGTTTGTACATTTAAAATATGATAAAAATTGATTAATGAAGTATTAATATTTTTTATTTATAATTTTATTATTGTATTACTATAATTTATGTATAAAAAAATAATTTAGTTGTTTTAAATTAAATTTTAATTATATAAAAATATAATTATATTATTTTTATTTATTAAAATATTTATTATTTATATATAAATTATAGCTTTATATTATCCTACCAAACAAATAGCAGAACTGATGAACTTCACTTCCATAACTACGAAACAAACAGCGGGGAAGAAGTAGTTTTTACCGAACTCCAGTTCAACCCAAAGTCCACTTCAACCCAAAGTTCAGTTTCGGTGAAACAAACATGCCCTTAAGTGCGGATAATTTTAAAATTTTGATTTTACTCTACCTTTTAAATTTATTTGATTTAACTCGGTCGATATTACTTTGACCTTACAATTTAATTTAATTACTTATTTAATGAATTTATCGGCGTGTGAATTGCATAAAGTATACTAACTAAATGTGTAAAATTAGACGACGCATTCAAATTCTTAAGTAAAAATGTTGTTGACTAACTCAAATCAAATAAATTGAAAGGTTGGATGCCAAAATCAAAATTTGGAAGGTTAATAATTTTTTTTCAAACTGTGCTAAAATTTTCAATTGGGCCAAATTAAATTCTCGATTTGATTTTGATAAAAACAAAAAAGTATTGTATGAATAAATTCAAATTTTTGCGCGTACCAGATTAAAATAACTCGTAAAAGCAATCAGTCAAAATGATTAATTTGAATGTGAATTAATTAGTCCTTATCAATCCTTTTTCCGATGATTGAAGAAAAAAATGAAACGTTTCTTTTTTATTTCATTTCAGGTTCTTACTGTTTCAGTGTCAAATTTTGATACTGTGGATTAAAAATTCTATGTATGAGGGAAGGTTTTTAGCTCAATTGTATGATTTGCGAAAAACCAATTTGCAAACACACAATGCTAATCTTGCTATATTGAAGCACAAGATGTGATTTTGTTCTTCAAAAATCACTATCATTCCTTGGACTAGCACTATTTCAACTACTTTAACTAACTATTGATATGGTTTGTAGCAGGTCCAAGGGGGATAGGGAGCTACTCTCAAACAGGCACCGAGTTTGTCCAGGCAATGATGGCGAGTCAGAAGTTAAGGCCTCAATGGAGCTTCTCGCGACAGGACTCGCTGTCGCAGATTGCAGAGATGAGCATCCCCGACATAGGAGAGAGTTCCAATGATTTCTCCATCACCTCGTGGGATGATACGAATTCATTCATGTTCTCCGCTCCTTCGAGCAAGCGAGCCAAGGATAACAATGCGGAGATTATCACTAGTATGAGTAACTTGGACTCGCAGGTAACCAACCATTGGTGTTTAATTTCTTTCTGAGAGCAGTAAAATGCACATTAGGAATTATGTTTCATGAATCCTTATATGAATGTGCCTTAATGTTAGAATATCTTGTCCAATACATCTGTTCTTTTTTTTTCCTGACGATGCATTTGGTATTTATAGTCTGTTGATTAGGGTGCAAGAAGAAGTTTAGAAACGAGTTAACAAATGTTAAATCATCTGTAGCTATTCTCCTAGGATACAATAAATCGTCTTGTAGGTGGAATCTACAATACTAAGAAGTCAGAATCACTAGGACTTCACAACAGCTATAAGTCAAACCCCAAATGTTGGTAATCATCAGAAGGAACTCGATCGAAAGTTAAATTTGCATTATTTTGAATAACAAATATTTTGATGACATATTTGATATGTCAAAACAGCACAACACATATGCAACAGTGAAACTTCTGAGTGACAAATATGTCAAAAACCCTTCAATACTTTTTCTACATTAACACTTGAATCAATTGCTTCAGATTGGCCTGCAAGGCACCTCACTGGAGATCTCCGGGATGGAGAACTACCTGCAGATCCAAGAAGATTCCGTCGCTTGCAAACTCCGTGCCAAGCGCGGGTGTGCGACACACCCGCGGAGCATTGCTGAGAGGGTACTTTTTTCTGTTATGGACCAAAAATAGTCATTTAAGTAGGTGTTTCGTGTGCTTCTTTGTATACTTGAATCTGTTGCAAACTTTTCTTGATTGAATATTGTTTCTTTGATGATATTCATATGCTAAGTTTACTTTTTGTGATCTTACTCCAATTAGATAATTTTCTATTGTTTATAGGAAAGAAGAATAAGGATCAGCAAAAGGTTGAGGAAGTTACAGGATCTTGTTCCAAACATGGACAAGGTAATTAACAAATTACTCTTTAACCTTATTTAAACAAAGGTCAACGTGGTTTCAGATACAGTATTCTGCCTAATCGCTATCTCCTAAAATTTCTAGCATTAGTAAAGATTTTAGAAGCTATCTAAGTTAACACTGATTAAATTCCAGCAAAAAAAGCAATTCGGTGACACTAGTTTATTGTTATAATTTCTTGTTCGTCTTGAGGTACAGCAAACAAGCACATCAGATATGTTGGATTTGGCCGTCCAATACATCAAAGATCTGCAGAGCCAAGTTCAGGTGTGTACCAAATTAAGCACAGATGAACACAGTTCTACTCCTTTTTATTTTGAGAAAGTCATTTTAACAAATATAAAATGTGAGATATGGCCAATTGAATGCATGAGCTGCAGAAAACCTTGAAGTAGGCATAAAGTAAAAATACGCATACTCCCTAGTAATATGAATGTTTACCTGCAACTCACACTTGGGTAAAATTTAGTTACAGGATCATATGCTATCACATTTCAACTAATTACACACTTTGTTGTCATCTACTCCAGAGTCCAAATATCAGTGCATTTACTATGGCCTCATTTTATTTACACTTTTATTTTTCTTTTTTAAAGCTCAAATTCTGAGGGATGGAACATACCAGCAGATCAACTTATTATTGTTGGTTAGCATCCAAACTTCTTTTCTTTAAAGAGAAAAAGAAACCAGAGAAATGTACTGCTAACTTTGTTTTCGCCAAACAATCCCCAAAGATGTAAATTGCAAACAATTTGTCACCTGCCAGTGCACTCAATCGCATATACTTGTTGAAAAGGGGAAAAAAGGAAAGCTTCAAAGATGTTTATCTTGTTTTTCCATATGACATTTCAAGTCTTTTCAAATTTACAGTGCAAACCTCTTCTAATTCTAAATTACTCTCGCAGAAGCTAAACGAAGAGCAAGCAAATTGCACTTGCTCAAGCAAGGTTTGAGCTTGGAAACTTCATGTGCATTAGGGTGTTTTTACGCCATTTAGTGGCTAATTTGGAAAGAGAGGACATTTTATTCATTGGGAAGTCATGTATAAGAGTTGCACAATGCTAATATCTTAGGTTCTCTTTCACTTGGGTTTGTTTTCTAAATGTAGCATAGGCAATAAAACTAGTGTTAAGCACTGGTTTTTTGCTAGTGTAAAGCTAGTGAACTTCTTTGAGTTTGTAATTTAGATACTCCCTTGATCCTTTCATCTTATTGTCTTTGTGAGGAGAAATTAATCCTTTTCGGTTATAAATCAAAAGCTTGCGTCTTGTTTGTTGTTGTTTCTG

>Aco012714; *Ac*bHLH43
ATGGCTTTTGCAAAGGAGCAAGCACCGAAAGAGTCGACGAGGCCGCATTCGATCTACGACACCATTTCCCTCGAGCTTTTCGGCTATAAAGGCCATCGCCACACTTCTTCATTGCTCAATGAGGTGAGCTACTGCGAGGGGCTCCCTCCGATCCTCGCCGACACTAGCAACTCGTCTTCTAGTGCATCGTTCGCGCTCGGAGTGCTCAACTCTCCACCGCAAGAAGCACACTCCGGCTTGAGCAGCTCGAAGACGAAGTCTGATAGTTGTTGGGCCTACTCGACATCTTCGGTGCTCAGCTTCGACCTAGGGGATCAATTTCCGCATAGTAGTTACGCGAATCACAACGACCAAGAGGAGGAATGTGATGTGTGGATCAATGCAATGGATCAAAATCACACCATGAATCAATCTAGCTTTGAGTATTCTTGGATTGTTCACGATCATAGTTACGAGACGAGAAGCGCCGCAAAGGATGACGCGTACGGAGAAGAGCAATTTGCGTTGCTACATCCGAGTTCGATCTCCATGAACGGCGCGCAAGAGGTTTCGCGGCAAGACAAGCTCTCTCAAAAGCGCCCTTATGCGGTTATTATCAGCTCAATTTGTTAAAGAATGTTGTTAAATAACTGATTTTCTCTAAAACCTTAAGCTATCAGAGAACGGTGCTTTTTATATATATATATTTTATGTTCAACGTGATTCTTCTATTGTATATATTGAAGAATTAAGTGATTCCATATATGGTTTACCATTTAGGGATCATATGTTAATGTGCACTAGTTACTATTCGCATAGGGTGATCACGAGATGCCGACTCCGAAGAAGCAACGCGGAATCAACAGGAACACAAAGACGAAGCCTACGCCACCGAAGGATCCACAAAGCGTTGCAGCAAAGGTGTGCGAATTAACTTATACATATAACGTACAATCAATCTATTGTTGCATAATTGATTTATCTAAACATTACAATCTCTTATAAAGTTTAACTAGTTAGTAAGATTATTGAATTATGCAAGCAATTGATGAACTTCAACTTTTATTACTTGTTATATTTTGTAACAGGTTCGGAGAGAGCGCATTAGCGAGCGCCTCAAGATTTTGCAAGATCTTGTGCCTAATGGAACAAAAGTGAGCCTCATCTGTAAAACAACCCCTTCATACATATATTTTTCTTATATATGTTCTTAAATCCTTTCGCCACGAACTCTTTCGTAGGTTGATTTGGTTACAATGCTCGAGAAAGCCATAAATTATGTGAAGTTCCTCCAATTGCAAGTGAAGGTATAGATGCATACATACATATTTATTTCTTTCCTCTCGACTAGCTCGATCTCCTTGTTTAATTTGGCGAAAGAGATTCGTGAACGATTGCATTCGATCTTAAATTTGTGCAGGTGTTGTCGACCGACGAGTTTTGGCCTGCGCAAGGGGGAAAAGTGCCGGATATTTCACAAGTTAGGGAGGCCATTGATGCCATTTTGTGCTCTCAAAGAGACATGAGCTCAAACTCTAAGAAGTGA

>Aco015158; *Ac*bHLH44
GAGGAGTTAAAAGAATCCAAAGAACATTAACAGCCATGGAGAGCTCTCATGGAACATCGACGAAGCTGGAGAGGAAAACAGTGGAGAAAAAAAGGAGGGAACAAATGAAAGCGCTTTACGCGAAGCTCGACTCTCTACTTCCGAGCTCAACTAGTTCAAGGGTATGTATATATATATATATATATAAAGAATTATATATGTTTCGATGTAATAAGGTGTAAAATATTCTAGAGTAATTCACATAACGGATATGTAGATCGCTCGTAGAAAAATGTTTATTTATATTTTTCCACAAAATCCAAAATTTTTGTATGCACCCTTGAGAAGCTACCAAATTAAGTACGTGTCTCAAATATATTTTTTTATTAAAGCCCAAATTATCATGGATTGGATGGCTCTTGCGATCGACCTAGCTACGTAACATTATATTCGAAGAGTAATATAAAAAATTGGGGCTTTGCTTATTTTTAGAGGTAAACATATGTATTTTCAATGAATACTATATAAAAACAATTTGGCACCCTATATATCGTTTTTAAGACTACGTACCTTTAGAAATATTTCGGTTTTATTGCTTTTTTTTCTCAAATGAATGTTAGAGTAGTCGATTTGACATTTGATACAACTAATAAGGCATCTAAATTACTTGTTCGAAGTTCTCGAAAGATATGTATGTGTGGTAATCCGTACTTTGTTTCGGTCCACAGGAAGGGGCAACACTGCCGCGACCTGATCGACTAAGCGAGGCGGCGAATTATATAAAGGGGATGCAGGAGAAGCTCGAGAGGATGAAGGAAAGGAAGAGGCAACTGATGACTCGCAATGAGGCGGCGGATAATTCGAAGCTTCCGAAGATAGAGGTGCAGAATCGAGGTTCAAATCTATATATGATACAAATCGTTAGCAGCCCCGATGACCGCGCCATGTTCTATGAGGCGGTTCGAGCTGTGGAGGAAGAAGGTGGCGAGGTTTTGAATGCCCAGTTCTCGACTACGGACCGAAAAGCCTTCCACACCATCTATGCCTTGGTGGGGTGAATCATATATATGATAGCTAGGTTTTACCATTAATGTTGTACTAATCTTTTCGCAGTTACTAGCTAACAGTTCTTATACTTACAAGGGCAATGCTAATCGAATATTCGATCGCCTTGTGGTATACTATTTAGATAGTTAAAGTGGGCACATAACAATCATATGATGATATACAAGAAATGCTCAAACGAAGTAGGTTAATTTGCTCAGTTCATTGATTCTTCTTGTTTGTAGTGTTTTAACAAGTCCCAAAACTATATTTACCAATAACTTCAATGGTTAATATGATTTGAAAATTAGGATAGCAAAATGTTGTAATTCGTCGTACAGGATAGTTAGTTAATATATACATACCATGCATATATATATATATAATTCATTTGATTTTGCTTAACTTTGGAATTGTGTTCTTGTAGGTTGGAGATTTTAAATATCGGTTTGAAACTGGAAAACTAAAGGAGAGATTGAAGGATCTAGGTTGGCAAGGGAGGCGTGTGTAAAAAGGATTCTTGTTTGTTTCGG

>Aco019347; *Ac*bHLH45
ATGGATAACATCACCACCACCAACACCAACAGTAGCAGCTCTTGGGACCTCGAGATGAGTATGGGGAGCCACCCATTCCTGCTCTTTGATTATCACCAACCCCCCTTTCAACTTGTTCACCCCTCATCAGAAGCAATATTACCATTAATCCATGCCCATAACATCCCTTCCCCGTGCTTTTCCACCCCTCCTCCTCACATAGAAGCTGCTCCTGCTGCTGCTGCTGCTGCTGCTGATCACCACCGTGATCAAGAAGAGGAGGAGGAGGAGGAGGAGGAGGAGGAGGAGGATCGCGAAGGGGGGGAGGGGGAGAGGGAGGAGGAGGTGTACAAGATCGCGGCGATGCAGCCGGTCGACATTGACCCCTCGACGATCAAGAAGCCGAGGCGGAGGAACGTCCGGATAAGCGACGACCCGCAGAGCGTCGCCGCGCGCCACCGCAGGGAGCGCATCAGCGAGAGGATCCGCATCCTCCAGCGCCTCGTCCCCGGCGGCACCAAGATGGACACGGCCTCCATGCTCGACGAGGCCATTCGCTACGTCAAGTTCCTGAAGCGCCAGGTGCAGGAGCTCCAATCCAACCCCCCGCCCCCTCCGCCGCCGCCGCCGCCAATTGCGTCGGCCCCCCACCGCGCGACGTTCCTCGGAGCGGTCCCCCTCTCCGCGCCCGTAGATTGGGCCGTGCATGGCGCGTCGTCGTCCTCGTCCTCGTCGCATCAAGGGCTAGGGTTTGGATTCATTGCGCCGAAGCAACCATGGGATGCACTGAATTAG

>Aco027360; *Ac*bHLH46
GAGGAGTTAAACGAATCCAAAGAACATTAACAGCCATGGAGAGCTCTCATGGAACATCGACGAAGCTGGAGAGGAAAACGGTGGAGAAAAAAAGGAGGGAACAAATGAAAGCGCTTTACGCGAAGCTCGACTCTCTACTTCCGAGCTCAACTAGTTCAAGGGTATGTATATATATATATATATATAAAGAATTATATATGTTTCGATGTAATAAGGTGTAAAATATTCTAGAGTAATTCACATAACGGATATGTAGATCGCTCGTAGAAAAATGTTTATTTATATTTTTCCACAAAATCCAAAATTTTTGTATGCACCCTTGAGAAGCTACCAAATTAAGTACGTGTCTCAAATATATTTTTTTATTTAAGCCCAAATTCTCATGGATTGGATGGCTCTTGCGATCGACCTAGCTACGTAACATTATATTCGAAGAGTAATATAAAAAATTGGGGCTTTGCTTATTTTTAGAGGTAAACATATGTATTTTCAATGAATACTATATAAAAACAATTAAGCACCCTATATATCGTTTTTAAGACTACGTACCTTTAGAAATATTTGGGTTTTATTGCTTTTTTTTCTCAAATGTATGTTAGAGTAGTCGATTTGACATTTGATACAACTAATAAGGCATCTAAATTACTTGTTCGAAGTTCTCGAAAGATATGTATGTGTGGTAATCCGTACTTTGTTTCGGTCCACAGGAAGGGGCAACACTGCCGCGACCTGATCGACTAAGCGAGGCGGCGAATTATATAAAGGGGATGCAGGAGAAGCTCGAGAGGATGAAGGAAAGGAAGAGGCAACTGATGACTCGCAATGAGGCGGCGGATAATTCGAAGCTTCCGAAGATAGAGGTGCAGAATCGAGGTTCAAATCTATATATGATACAAATCGTTAGCAGCCCCGATGACCGCGCCATGTTCTATGAGGCGGTTCGAGCTGTGGAGGAAGAAGGTGGCGAGGTTTTGAATGCCCAGTTCTCGACTACGGACCGAAAAGCCTTCCACACCATCTATGCCTTGGTGGGGTGAATCATATATATGATAGCTAGGTTTTACCATTAATGTTGTACTAATCTTTTCGCAGTTACTAGCTAACAGTTCTTATCCTTACAAGGGCAATGCTAATCGAATATTCGATCGCCTTGTGGTATACTATTTAGATAGTTAAAGTGGGCACATAACAATCATATGATGATATACAAGAAATGCTCAAACGAAGTAGGTTAATTTGCTCAGTTCATTGATTCTTCTTGTTTGTAGTGTTTTAACAAGTCCCAAAACTATATTTACCAATAACTTCAATGGTTAATATGATTTGAAAATTAGGATAGCAAAATGTTGTAATTCGTCGTACAGGATAGTTAGTTAATATATACATACCATGCATATATATATATATAATTCATTTGATTTTGCTTAACTTTGGAATTGTGTTCTTGTAGGTTGGAGATTTTAAATATCGGTTTGAAACTGGAAAACTAAAGGAGAGATTGAAGGATCTAGGTTGGCAAGGGAGGCGTGTGTAAAAAGGATTCTTGTTTGTTTCGG

>Aco002862; *Ac*bHLH47
AAACTCTTTTCTCCTCCGGTCCCTTCCCATTAACCTCTCCCTCCTCTTGTCTCGAGTGCTCCTCCCCTATAAACTTCCCCTCCCTCGCTCTCCCTTTCTGTCTCTCTCACATCGATCTCCTTCGCTCCGCGACGATGTCGAGCCGGAGGTCGACAAGGATCACCGAAGAGGAGATCAGCGAGCTCATCTCCAAGCTCCAGTCGCTGCTTCCGGAGGGCCGCCGCAGAGGCAACAGCCGGGTATTAACAATCTCAAGTCTCTCGAAGTAAAGCACCATACAAATATAAATATATATTTTAGTTAGAACATTTTGCTTGTAACTTTACTTTTATATTATGATGATGATGATGATGATGATGATGATGATAATGATGATGATATAAAATAATAAGATGCAGGCTTCGGCGACGAAGCTGCTGAAGGAGACGTGCAACTACATCAAGAGCCTGCACCGCGAGGTCGACGACCTCAGCGACCGCCTCTCCGACCTGATGGCCGGCATGGACAACGACAGCGCCCAGGCCGAGATCATCCGGAGCCTCCTCCGGTCCTGAGCGCCGTGCCGTGCCGTGCTGTGCTGTGCTGCGCCGCCAAGTTAGCCCCTCCAACGTACCACTCCTAATAGATAAATCTATATATATATTTATAAGCTTCTAGGGTAACTAGATCTCATTAATGTACGTACTTATATATTAGATCCTAGCTAGGCTTTATAATGCCGTAGCATTAGCTCTCCATTCCCTACCTCTTCTTCTCCTTTTCCTCTTTTTTTTTTTTTTTTTTTTTTTTCAATTTTATGCACATCTTCTTTGAGGTGTGGCTCGAGTTGCTTCTACTATGGCAACCGACCAACCACTACTTAGGAAAATTATCATGTAATCCAGCTCTTACTCAGTTTCCATTATATATATATAAATATATATATAAATATAAAACCTTATTTTATATATACATATATATATATGCTCATTTTCATGGCTCCTTCTTAGACTCCCTAGTTCAATCCGTACACATGTGAAAGGGGAGGTACTTAATAAAATATATAACTACCACATAAGTTTTTTTTTTTTTTTTTCCTGAAAAATTCCTTTTACTCCTTAGTTCCTGTTTAAGACCCAACACCGGCTCTGTTTTTATTGTGTTCCGATGATTCTAACACCCCGGCGGGCAAAAAACCCACCAATAGGGTTTGCACACGTGGACGGTACGGAATAGTGGGTGGAGCGCGTACTGGGATTAGGCTGTACCTGTGGAGCAACGGAGGGGCGAGGATGTCGACCGAGGGAACTGATCTAACGGTTGGTGGCCTCCGAGGACGCCAAAATTGAAGGACGCGGGTCCCGAGAGTCAGGGCCTGCTGGGCTCAGAGCCCCAGTGCGGGGCCCACCACTGATTGGGGCTCGGTCCCCACCGTCCAATGAATAGTATCCTACGGTTGTAGCTAGTTAGTTGAACACGACAGTTAAAAAAGGCAAAAAAAAAAAAAAATCATTAAACAAAAAATATTAGAAAATCCTCGACACCGTAATATACAAATATATTGAGATGATAATGGCATAATATGCCCCTACAAAAGGCTGTGCTGTACATGTAACGGGAGGGGTAAAAGAGTAAATAGAACAGAGAAAGGGCCAAAAAGAGGAAAAATAAAAAAATAGGGGGTTAAATGGCGAATAAGATAAGGTGCG

>Aco003064; *Ac*bHLH48
GAAAAAGGCGCCGTCTAAAAGGGTCTGGCGAGAGGCGCGCCGACCCGTGTCCACCTCCGCCACGGTGCGCCTGCGAGATCCCAAAGTTTCAAACACCTCTCTCTCTCTCTCTCTCTCTCTCTCAGTTATTAACGCATCAAAATTTGGCCCCATTACAATTTTCCCCAGATCTATATCTCCCTCTCCCACGCCCACTCCCTCTCCCTCTCTCTCTCTCTCTGAATTTTAGAGTTTCGATGTATCACGTTCGTTCCCTTTTCTAGATATATCCCCGCGAAGGATCGGAGAGTACACTCTGGGGTGAATCTCTCTTCTCTCTCGCTCTCTAAGGTACTTGATCGAAGGTTTTGTTTATCGATCTATACGTGAATAGTTCTCTAATTTTGAAGTTGTATTGATCTATATGTGGATAATTCTAACGAATTTCACGATCTATAATGCAGAAAACATGAGATAAGGTTTTTTTTTTTTTTCTTTTTTTGAATGTGATATTTCGAGATCAGAGGTGAATTCTCTTGGAAACTCGAAGTAGCGAGCATCAGAGATTACGAGAATGGAGCTACGAGGTAAGTGGCTGAATAATTTTACGCAGTAATTCGATGTTTTCGTTGAAACATTTTCAGATATAAGTTCTAATAATACTTCTACAGGAAAGAAAACAACTCACGATTTCCTTTCGCTCCACACCGTAGATCCATCGTTCAAGCGCCACGATACATCACCTTCCTCCTCCCAAGGTAATCGATCGGTGTCTCTCAGTTTTTTTCCCCCTCTAATTTCCTCTCGTTTTTTGAATTTTAGCTTCAAATTAATTTATCCAGCATCCAAAATTTTAAATCGTTCTTCATATATTCTCAATCGGTTAATACTGATAGGGATATTTTTGGAATTAAAAGACACTTTACGCAATGCTTGAATGTCCTTACTAATCACGTGTCTGCAATCCATCACATCCGTTGAAGTTGTTACTGACCCGGTGATCACGCCGTTGGAGCGACTCCACCACTCGGTGAGCCCGGCTCACCGTCGTTCGCCAATCAGAGAACGCTAATCGCCCTTTTTAATGTTTTTCTTAAATGCAGGATTTTTTATTAAGACGCGCGATTTTTTACAGCCGTTGGATGACCGGGAGGAGGAATCGGCGGGCCGGGACCCCGCGGTGGGCCCCACAGAGGAGGAGCGCACGCTCCCCGGGGGAATCGGCGCGTTCAGCATCAGCCACGTGTCCGGCCCTGGCTCGAGGGCGCCGCTTAAGCCCGACCGGGGCACGTGCGGGGCGGTTCGCGGGTTCGGTGTGAAGAGTAAACCCGAACCCGACATCGACAGTGGTGTTTATACAACTCAAAACGTCGGTGTTCTTGGTGCACCTTTTTCTACGTGGGCCCATTCCACAGCGCGGGATTCCGGTCCATCATCAAGAGGTAAGTGACATTAACATAACTGGTTGTTTGAGCTGCTCTGCTTACAGTCGGTTCCAATCAGTAGTTTCTCCTAAACGGTAGTAGTTGCTGCGCACCTTTCGGCTTTCGCACCTGCTGTGCACTGCATGCGCACTGCGTTCTCGGTTATTAGTTAGTTCAGTGATGGCATTTTCTTAATTTCTACACAATTGCAGAGAGTATGCTTCTACTTAGTTCTAATTAGTACCACCGTATGATTGCAAAATTTGGTAGTTTAGTCCAGCCACCTCAACAAAGTAGCCTAACTTGTGATAATTGTTTTTGCGACCATACCTAGAGTCATTGGCTAATAACTTGATAATTGCATAGTCAAAATCTCAAATTCAACATTTTAATAACTTCATACTTTTAACTGAGTTTATTTATATAAAAAATAAAGTCGATAACTTAGTTACCTTTCTCTCTCAAAATAAACAAGTGTGATTTTGTTATGGGCCCCTATTTCAAGAGCTAGACAAGAAGGGATTATTCCAATGTCTGTGTTTTATACTCTCAGCTGCTTCCCACAATATATATATATAATTGAGCTCCTATGTATCAAGTTATTGGTGATTTTAGATATTTAGCTATTGGATTAAAGAATGTGCGGTTAGGATGATATGAGATTCCTAGGGGTGAGCGTGTGGTTGATTGAATAGTATGATTTAACGGGCGAAAATGATCAAAGGAATAGATCTAACGGTAAAAAATTTACAAGCACAAAATATTTGGTGATTTTACAAGCAGCATAGCCGGACTTATATAGAATTGAGCTTTTATGCTTTTAAAAATATCAAATTATTGATGCTTGTAGAGTTTTTGCCATTGGATTAAGAGATGTGCTGTTAGGATGATGCGGGACCTCTAGGGTTGAGTGAGTGGTTGGTTGAATAGTATGATCTAACGGGTGAAACGATCACAGGATTAGATCTAACGGTAAAAAGTTTATAAGCATCAAGCGCTTGGTACTTATACAAGCACCATAGCCAGACTTTCTCTCTCTCTCTATAGAGAGAGAGAGAACGAGTTGAGCTTGAATACCAATAATAGTATAGCACCATAGCCGGACTCTCTCTCTCTCTCTATATATATATAGAGAGAGAGAGAGAGAAAGGGTTGAGCTGGAATACTAATAATAGTATTTAGATTCTTTTGCTATCAATTTTTTAGCCGTTAAATCTACATTTTAATCAATTCTATCTTTTAGATCATACTATTTAACCAATCACCCACTAAATCCTAGGAAGACCACTACATAATCCTAGGGGGACTACTATCATTCTAACCCTACAATTTTTTATCAAAAGATTAAAAACTCAATAGCAAAAGAGCCAAAATACTATTAATAGTATTACAGCACAATTCTCTGTGTGTATTATTAGTACCAAGTATTTGGTATTATTGAATTTTCTGTCGTTAGATTTACTCATTATTATTTTTACTTGTTAGATCATACTATTCAATTAACTACTCACTCAACTCAAAGGGAGTACTATTATTCTAGCTGCACAACCCTTTATCCAACAGCCAAAAATTTGAGAGGAATGCTTCAATACACCAGGTAAAATAAATCTAACAATTCATATTTTAAGGGCGAGATTGACTTTTGCATAGTAGTTACCTGCTCAACAGGTTACCATTTGTAAGGATAATATGAAAAAAAAAAATGCAAACTAATTATCAAATTTGATTAACCTTATACAAATATTTATTTCATTGTTTAATTTTGTTACTTACCAAATTAGATAGTGCTAATTTGATAAGTAATAAACCTTTATAAATTGAATTTAAAATTCAATTTATATTCAAATTTAATTTTAAACTTTATAATTGTGTACATTAAATTCAAATTTAATTTTAATTTTTATTTTCAGAATGAAAAAATACATGTTTAAATGAATAGTTTATTTCAATTCAAATTGTTAATTTGATATATTAAAATGATACGAAATTTCAAATATATTAAATTAATTTAAATTATAAATTATGCTGTATTTATAAACATAACTTTTATTTTTTATTTAAATTAAAGGCTTTTATTTCTAAAATTAAATTCAAATTGTTCATTCGATAGTAAAAACAACATTAAAATTTAAAAACATTGATTAAAATTAAAATTAAAATTTAGATTTAGTTTATATTATAGTTAAAAAAATAAAATTTGTTAACTATTTAAACTAAATAATTCTAAAGCAAAAAAGGAAAAGAGGAGCGGCGAGGAAAAAGAAATCGCGGCCTACGTGTAGGAAAATATCACGGTCCACGAGGCTGCACTCAGCCTCGTAGATTGTGCGGAGTGAGAGGTCCACGGTGGACCTCTCGCTCCACTCCTCCTTTTTGTGTGCGTGTGTGTGCGTGTGTTGTGTGTGTGTATATATATATCCAATTTTATTTTTATATTTTATAAGAATTATAATTATTTATGCATGATAATAGCATAAATCTAAAAAAATTTTAATTAAATAAATGAATTTTTATTCTGCATTTTTAAATGATAAACTTAAACTTTTCGTATAAATCGCGTATACGAATTATTGGCGAATCTAATTTTTAAACCGTATTTTTTAACGAACTTAAAAATTAAGATACAAATTTTATTCGTATTTTATCCGTATCGAATTTTTATCGTATTCGAATTAACTAACTATGGTTTAAAGACATGAGGTTTAATTTAGGAAAGATAATAATACAAAGATGGAATATTGGAGGATAAGAATGAAGGAAGATGGAATATTTTAAATTTACAAAAATAACATGTTATGAGGATGAAAATTTACCAAAATGCTCTCATTGCAAACTCCATGTAATAAAAAAAAAAAACCCTTATTAAAGTGGTGACCGGTACCTATACCCCCAAGTGCCGGTCACCCTCTTTTAATAGGTCTATCGTTACCGTTCATTTAAAGAATTTTAATGGTTGAGATTTGTTCGGTGCATAGGAGCATTGCTCTATTTGATAGTACAAAGTGCTTGGTACTATCGATAGTATAATAGTATAATTCCTATGTATATATAGATATAGACTAGGCTGCTATACTTTTATAATATAGACTCTTTTATAATTATAAGTTTTAGATTCTTAGATCAATGAATATGCAGTTAGTATAATAGTAATTTTTGATTAGAATGATAGTAGCCGTAAATTTTACTAATCAATTTTTGTTCTGGTGGCAAAGTATGATTTTTCGTTCAAGCCTCTCTCTCTTTTATTGGTTGAGCAAGTAGTTGTCACACGAGACCCACGTGATCGTGTTGTGACGCCACGCGCCCACAGAGGCGTTATGTAGTGGCCCGAACAATCCTCTTGGCGTGATCCGCGAGCACGTCTTTGTCACCGCACCACCACGAGAAAAATAAAATAAAAAAAAATAAAAATAAGAAATAAAAACCCGGACGGAACTCGAGCCGGAACTCAAACTGTCCCCAGAACCAAATTGAAATCGCCGTCGGATCCGGTGAGAGGGGGTACGTTGAGAAGAGGGGGTACGTTGAGAAGAGGGAGCCGAAACAAAAGGATTTGAAAAGGTGAAAATGTACGTAGAACCCTCACCACTAGTGGATTTTGAGCGGAGCCCTCAAATAATGCAGGCGTTCCATATTTGCGATTTCCATTCTAATCACAGATAGAGAATATGAATGCACATTTAGTAGTATACGGGTATTTTATAAAAAATAGCTAATCTATGTTTCATCCAAGTGATGATGCTCTATTAGCTTTTAATTTCTTAATTTAATTATTTTAATAAAATTATAAATTTCTTAATATTGATAGTTCTACTTGCTAGTTCTTAATAATTAATAAAATTTATAAATTTATCAGAAAATATAAAATTTGAATGCGATTAAACTGGGAAAAGAAAAATTGAGGGTCTATTTCAAAATTATCCATTAGTTGGGACGGTGATTGTACATATTTGCCTTTAAATAAAGGCCGGTGCCGTCGCCTATGAACCTCCACTCCACGCCTCGCTCTGTAGCACGAATCTTCCAGTTTTTAAAATTATAAATCGCTTTTTTATTAATTTAATTTAATTAAAATTAGTATTTTAAAATAATTGGGAAGAAGAAGAAGAAGGAGGAGGAGGCTTAGGCTTGGGCTGTGTTTTGTATTCTTCGGCGCAGGGCCGTGGCCGTCGCCATTCGCCGCCGCTCGCGCCAACGGCTTCGTCGGATCCCTCTCCTTCGCCTCCAGGTTCGCATCCCCTGTCTCTCTCACCCCCCTTTTCGCCTCCTAGGGTTTCCCCTTTTATAGGGTTTTGGAGGGTTTCTGACCCGTGGGAAGGGTGTGTTGGGCCACAGGGACAACGCGGCGACGGAGGGGAAGCGATCCATGGAGGCCGCTTCGAGGTCTAGCAGGGGGGCCTTCGACGACGACGACGACGACGACGGCGACGAGTTTGGTAGGCGAGAGGCCTCCTCCTCGCGTAAAGGTCCGTCCTTTCTCAATCGCCTTTCGTGTTTTTGCGCCTTTCTCTACTTGGGTTTTGGTTTCGGGGAAAATTGTTGGCCTTTTCTTGTGGCTTTTGGTAGTATAGTGGAAGTTTTGTGCGAAGGTTTTCCTTTTCGTGTATATGAATGAAAAAGGAATCAATTTTGTTGTCTTCTGTGGCAGAGTTGAGCATTAAGGTAGACGGGAAGGGAAGTGGTAGTGGCAGCGGCACTGATCAGCGGCCCAACACGCCGAGATCAAAGCATTCTGCCACGGAGCAGCGGAGAAGAAGCAAAATTAATGATAGGCGAGTTTATGTTGGATGCATCACTCTTCTAGTGTGTTGTACTCTCATTGTTTTCCTTGTATTCTATGTTTATTCAATAGTTGAATTGCGCTTATATGTGAAATAGCTAGTTTTGGTTGCTGATTTGCTTTGCGATGCTCAGGTTGTTTTGTGATCATTGGGTCATTTTGGAATTATGTTGTGCTACAAAATGCTACATTTTTGAAGTTCAAAAGCAGTTTTCAGTGTTAGTATGATTAGTGGACTGTAGTCAGAGTTTACACTTTCCTCATCTGGACAATTCTATACCATTTAGTACAGATCTCAGCTGATGTACCAACATCTGAGAATGTTTTTTTTTCGTGTTTTGATTTATGCTAATCATTGTAGAGTTAACCGTGTCCTGATAATAGCAACAGTTACTGATAAGCTCGGCATTTTTTTTGTGGGTACAGTATTCAATTACGTCAATTGTGCTCTTTTGCCGCTTTGGAGTTTTCAATGGTGATGGATATTTAAAAAGTTTACAACAGCATATTTATTCTCTTTGTCCGTATCTTTTAGGTGTCATTATCGGTGTTAGCTGATTGCAATTAAGTTCCAAATCCTGAGGGTAGATGTGTCCGATAGGTGCTGGTTCAATTCTCCAGTTTTGTTAGACACTAAAATTTACATCAGATTCAAGTTCATGCAGTACCAATGAGTGGCAAGTGCTTATTTGAAACTTAGTGAGCCATGAAGAAGGGCCTGATATTAGCATTTAGCAAAACCTTGTGAAATATTCCAATTAAATTTCTTGCATGTTTTTAGGCTTCGGCTTAAACATTGAAACCTGATCAATGTGTTTCCGTTGGTCATGTAGACCAAAATTGTGGTCTAAAATTATAACATATCTATTGTTGAGAATCAGCCAAAGGAAGGCTGAATTTAACATTGTTGACCAAACTGATTACCTATGGATGTTTGATGCTATTTATTCATCATTTTACATGTACTCTGCTACATTTTTTTTATTGAAGTATGAAACATGATAGTTATATGTATCAAAATATTGCAGAATTGGGTTTTAGTGTGATAATTCTAAGAATCTAATCTTTGTGTTGGCCTACGGGCGTATTCTTAAAAAAAATGTTTTTGCTATAATCTATTTTGAAATTTGGGAGATATCATTAAACACTGATACATCCTGTATACCTCCTCTTTCATATGCATCACTGTTATTTTTAAGATCATGAGCTGATGCATTCATGCACATGTTAATTATGTTAAGTAGCTTCGGTAGAGTTTAATGAAACAAGGACAATGGCAATTATTGACTCATGGCATGTTCTTGACCTTTTTTTTTTTTTCAACTGTCTTATTTGCTCAAAATTAAAACAGATCCTTCAATTTTTTTTTTCATGCAGATTTCAGATTCTTAGGGAACTCATTCCACAAGGTGATCAGAAGAGAGACAAGGCTTCATTTCTTTTAGAGGTATCCTTATAAGAGACAATGTTTCTATCATGTTTGTTGCCATAGACGATTCCGAAATCTTTTTTTTTCCCTTCATGAAGGTTATTGAGTACATCCGATTCTTGCAAGAGAAGGTACAGAAGTATGAGGCAACAAATCCAGAATGGAATCAAGAAAGTTCCAATTTATTGCCGTGGGTGAGAATTTTTGAGCCTTTAGCACTAGAATGAGAATATATGCATTTATGTATAAATATTTTGCCCTGATTGGTGCTTTATGGTTTTGTTGTTAATGCCTGTGGGAACTTCAGTTGGTGAAAGTATTGCATGAACAGAGATCTTTGGGTCTATAAGTTTGGCCATGGCATATGTGTGCTTTGTGTTATATGTATAAGCATATTTGCTCGACATATAAATTTTTTAAATAAATGTTGCATGTCATATTCGTATTTAGACAAACCTTTCCTTTACTCTAGTAAGTATTTAGAATTATATTCAACAAGGTTTAACTAAGTATTTAGAATTATATTCAACAAGGTTTAACTAAGTAAAGCAAACCTATTCTGCATTTGTATCATATTAACTCCATTGGCTAATCGGGATGCGTGCGGAAGACCATTCATTAATATTGTATAGTAATTTCTGTCTGTGAATGCTGTTTTTTTCTTTCTGTACATGCAAAAATGTTAATTTCGGCAATTCTTGGGGCCTTTTTAGTCACCAAGAAAATATTGCGGTCCTTGTTAATTTTCACTTTGGCTTTCTATGGCAGGTTAACGTCTATTTTAGATCTTTCTGGAAAAGTGCACAGGTATCTGTTTGTGTAGAATATTTCAACTGGGATTACATAGTACTTATAATTTCTCCTTTGATGAGTGTCTATTCTACTAGCCTTCTAAAATTGTCTTTCCTTCTTTTTTTCTCTTTTTTCTCCAATAGAGTAACAACCATGGTCTATCCGACCCTTTACAAGCCATTAAAAATGGTTCCTCTCCATCCGGTTACATGTTCTCTGGCAAATACACTGACAATGGTACTCAGGTGGCGCCTGCAGTTTTGACAGGTGTGGCACAGAATCGAACTGAAACAGAGATGATGACAGCTGGCTTCTCTAGCAAAGCTATGGAAACTCCTCAAAATTTCATAAGTAAGCCCCAGAAATATCTTAAATTCTTTTGTTGGGCGGAGAATTAGTGTTACTCAATTGCAATTGTTTCTTTTCAGATGAAAGCATGAGTGGCGCTCAAGCCCCATGGTTAAGACCACATGGTGCAGCCGACTCTGCTGTTAATGGTGAAATGTTAAGTGAACAAGAAGAGCTAGCAATTGATCAGGCAACTGTTAATTTTTCGAATGCATATTCCCAAGGGTCTGTCTTCTAGTCGATTGGCTCTATTTTTACTTCTGGTTATCTTTTGTTACTACGACCTTGTCTCTTTTGGCTATATGTTTCTTGGTTCTTTTGATGATTTTATTTCTTAAATTGCGTGATTACTATGGAGTCATAGGTGTCCTTATACTATATTTGTGCCTTCCTATGTAGTGAGTTTGGTTTTCGAAAGTTAACTTAACTAGCTTATCTCGTAAAAAAGTTTTTTCCAATCATTCTTCTTTTCAGGGTGCTGACAACACTTACCCAGACTCTTCAGAACTCTGGAATAGATCTATCACAGGCCAATTTGTCTGTGCAGATCAATTTGGCAAGGCGGTCAAAAAGAACTAATGCCAATGTGACGGCTTGCACGACTAAGGTACTATATTTTATTGTCATTTATTATCTGTTTTCAAAACTAGGTGGTAAAGAGTTCTGATCCTGTTGTAAAATGGTGCCTTTGTTTCTTTCTTTTCCTTGGTTAGATATGATTCCTAATGATGCTTGCTTAGCTGCTCTAAGTTTCTACTTTATTGTTAAATTTGGTAACTTTGATGTCCTTGTTGATAATCAAATCCTTTGCATAACAACACTGGTCCTCTGAGAGATCAAGTAGTGAGGCGACAAGTCCTGTTGATCATTTTGCACTCATATTTTAGCCCTAGAAAATAGCTGTAGGCAATGTTAGCCTCTGTTTGAATGAGAAAGTACTTGTAGGGAACATGGGCCTCACGGCCCTCACCTTGTTGTGCAAAAAAGCTATATTTGTGAAATGTTTACTCTTGGGAAATCAGCAGCCTCTCAGAATTTGTTTAAATTGCATTATTTTGTACTTGTGATTGTTGTGCTTCTTTCTGACCATAAAACACAATTAACATTGTAGTAGTTCATCAACATTGTTATCTGCTTGATGTATTTCTTTTAATATGTGGATTCTTTTTCCCTATTTGCCTATCTCACCATTTTTCCATGGAAAATAAGGTTGAAAATGACTTGATGAATGTCTTATTATATTCAGTCACTAATCCTACACTCCCGCTTGCTTTGTTTGCTCGATTACTTACCCAATAGTCCTATGTAAGAAGTCCAGCTTCTGGCTACAGCTAAATACTTAAGATCATTTATTTCTTGATCTATGGCTGATGCTTTATTTTTGGTAGTAGGATCAGGAAGATCCTGCATGTAGCAATCAAGAAACTGGGTGTCGCGAGGATGTTACGCAAGCACCTAAACGGCACAAAACTGATTACAGCTAGTGCAGCTACTACAGTACCCCCTTGTGCTTCTCTGTTCAAATATGCTAACTCGTCCTCTTTTAAAGCTCAGTTCCTCGGACACTGTACATTGTATGTTTCAACTGTGATTGGCGCCAAAAAGCCAATCGATTGGCGCCAAAAAGCCAATCAGGTATTCTTTCATTTGTCTCTTTGCACCTCTAGAAGAGGCTTCCATTATAAATTGCGAAGCCGCTTTTCTTTACAGGCTTCCATTGATATGAGAAGTTGCTCTTTTCTTTTGGCAAAGGGACTGTGGTTACTAGATTGTTTAATACTCTGTGTTCTTTGTAGTAAATATCATTGTATTGGTTGAGCAGTGCATCTCTGACATTGTTTATGCATTGGTTTCATTATTGGTTCAAAAAGTTAGTAGTTTGAGTAATGGCCGCTGCTCAGTTAGTCGGACCTCAGATGTTAGAAAAACTCTATATAATAATTTTTGCTGATTGTCTTGTGCTTTTCTTTGTTTT

>Aco003149; *Ac*bHLH49
ATGTACGGATCTCCTCAGGGAGCGGGCACGCGGAAGGATCTGAGCGGTCCGTTCCCACCGGCGTCGGCATCGGAGGACTCCGACCTCCTCCTGCTCCACCGCCATCACGAGCAGCAGCAGCAGATGACGAGCTCCGGGTTGCTCCGCTACAAGTCGGCGCCGGGCGCCCTGCTCGGCGAAATCGGCGACGACTTCTTCAACCCCGCAGCGGAGAGCGTGTTGAGCGACTTCTGGGACAATTCGTCGAACAGTTTCCGGGCGCCGGAGCCGTCTCCGCAGCAGGATCTCATCCGCCAAACCAGCTTTCCTGCCCCACCCTTGCACTTCAACGTAGAAAAAGGTATTCTCTCTCTCTCTCTCTCTCTCTCTCTCTCTCTCTCGCCCTCGCCCTCTCCCTCTCCCTCTCCCTCTCCCTCTCTCTCCGAAAATTGTAAAAGGTTTACCCGAGATTACATATACGACTCCGGCTCATCAGATGCAGTAACAAAGTATAGATTGATGCCTACTGTTCATCTCCTATGTTATTTTGATATCCATGCATAAATTAACTTTTTAGCGACTTACTTTGGCTGCTTAGGACCCGGTCACAGCAGAGGCGGTGGCGGCGGTGGCTGCAGGACTTATAATCCAGTGGGTTTTCCCAATAACAATAACTCGTTGGATCAAAACCGCTCGGGCCCCAGCCCTTCAGATCTCCAGGCATATCAATTAGTTTTCTCTCTCTCTCTCAATCAATCGTCTTTCGTAAGAGATTTAAATGGTTGGTTCTTACACTATCTGATTCTTCAGGATCAGAATGTAGGGGTGCAGAAGCACCAGGATTTTGAGCTGGCCCACCAGCTCAGCCTCCCGCTGACCTCATCGGAGTTGGCCGCCGCGGTCGACAAGTTCGGTCGGCAGTTCCAGGACGCAGTCCCTTGCAGGATCCGCGCCAAGAGAGGCTGCGCCACCCACCCGCGGAGCATCGCGGAGAGAGTACGTAATTATGCGTCGTTGGTTTCCCGCCAATCATTTCAAATTCTACCTGCGGATCGATTGACAGGCTTATAATTTTGTGCTTCGTTCAATATTCATCTGCGAACTTGCAGGTGCGGCGAACTCGGATCAGCGAGAGAATGAGGAAGCTACAGGAGCTCGTTCCCAACATGGACAAGGTTGGTTCTTGGTTCTTATATAGATATATATTTGTATACATATATATCCAATGGAACAGTAACTTGTTGCAAATTTGATCAAGTAGAATTAACATTACGTACTCTGACTTTTTTTTTTCCTTTCCTTTTTTGGTGATTGGATGAATAGCAGCAAACGAGCACTGCCGAAATGCTAGATTTGGCCGTTGACTACATTAAAGACCTGGAGGAGAAAGTCAAGGTAAGTGTATACGTTGAAGATGTAATCCCTTTCCATCGTGTGGTAGTGCTGTGCGTTTGACAATGTCTCTGGTTCTTGAAGACGTTGAAGGAGAGCATGTTTGGATGCACGTGTTCTTCTAGCAAGAGCTAAAGCCCTAAAGCCCTGCTCCAAAACAAACACCATGGATACGGAAGTCAACGCAATAGCAAGCGCCAAGCAGTGCCGACACAATTATATATCAACTGCTGAAGGGAGATATTCTTAAACTTGGTCGCCATTCGCTGTTGAATTTGACTAGATTTGATGAATCGGATTTGTGGTAGCAATAACTCTCTTAACGATGTTATCCCGTAAAGATGGCACTTATCTTGAACTTCTCGTATGTATATCAATCCAGACTAGCTATCGACCGATATTTATCGTTGGTTCAAAACTGAAATGTCAAGATAAAAATTGAAGAATGGAATAGAACGGACACTACACGAAGTTTTCTTAGGGCTATCTGTAGATAGTACAGAAGTGTTTTGAAAGGATAGAGATTGGTGAGCTGAACAAATGTATAGTGTTTCTTGCTTTGGTAAGTTGAATGCATCTGCCACCATGTTGTGTGTATATATGTACCTGTTTCAAGTATTAAAGTTCGTTGAACACTAGCTTCTTTTGTACATCACCGCACGTTTCTTTTTTCCTTTTTTTTTTTTTTTTTTTTTTTTTTTTTTTTTTTGGCTCTATATGTCCGGTGTCTGAACGTTGAGAATTCTCTTGCCGTGGATGGCCTAACATGATCTAATACTCCAAGTATTAGTTCACCACAATGTCTTGGTTGGACGGCAATTTACCTTTTGCCTTGCAAAAAAAGCTATGTATGTACAAGCTTACACATCAAATGTCTACTTCATTTTGGTTGGGGATAAGACATACATTTATTAACAACGACTATCATCTTGGAAACAATTTTCAGACTCTTGAAAATTAAGACATTTAGACGCGCCTTCCGGAGCACTTAATGTATTACGCAACAGAACTCAATTTTGTTTTGAAATTAACAAAGAATTATCCAGTTGTCCCATACGGGGACCTAATGAGTTTCTTTTCCTCGTGAAAATGGCGTCAAATTTTGAACCAAATATGTCTATAATCTTTCTCAAAAAAAAAAAATGTCTATAACGAAGAATAACTCTGTAAAAGCCACCGCAAATACCCATCTCAAAGAATACTTTGCATAAGATCATGACGGGTCAAAAAAGTAATTAACATTGGAATTGCGGTTGTGACTTGTGAACGGCAGTGGAAGGAGGTCAAGATCAGAACAGCCGTATTTCGCAATCTCTCTGCCCTCTTCGAAGGGGAAATACATGAAAAGGCCACAAAAAAAAAAAAAAGGGAAAAAAAAGATGACCTTTCCTTTTTTCTTGTTCCCTATTGGTATCGGCAGCAGCAACGTAGTACCAAAGACGACTGCCGTACATACACATAAAACAGACTCTAAATCTTCGATTTTCTCATTTAAAAATCTAAACCTTTTTTTAAGTTTTCAATTTTAATATAAATAAAAATTGATTATATTGGTGTTAAAATGTAAATCATTGGATGAATAAACAGTAAGATTTATATTTAGTTCTTATGTGTACATATAGCATTGCTCAGTGTGACATACAAAAAAACAAAAAACATGCTGGAAGTACTTTCAATTGCTAACACCCCTTGTATTATGTTTTCTACTAATGAAAACTTAATACTTTTTAAAACATAATTTAAAAATTGAGATTGAAAGTACTAATCAATGAAGGTTTAATCCAATCGACAGATGAAGCAATTATGGTGATAGAGAGAAGGAGACAGAGACTGGGCGGAAGTGCTCGGTAAAAGATAAAGGTAGAGGGCAATGAACAAAGGAAACACACGATGGAAGCGGCATCAATACCTTTCATCATACGCGCATACATCAAACTTTTCTATATATTGAAGTAAAAAAAACCAGATACATGATAAATAAAGTCTAAAGCTTCTTAGTAATGCAACAAAATAAAAAAGAATAGAAATGAACATAAGATGCTAAAGAATACAAGCCCTAATTTATAGCTTTAGTAAAATGAACTTCATAGTTCTACTTTCTGCAGTATGTCACGGCACAAAGATTTTCTCCGCGATTTCGCTTTGCAATCACCCAAACTCCAAAGCGGACATCTGACGGAGGTTTTGGCTTGGAACCTTCTGGAAGCTTCTACGTGGATCTCGAGGCTGATTGGCGCGGATGCCGAGGAGTCAAGAGTCCCTTGGGGTTTCTAGGCAATAAATGTATGACTCTTAGAATTCCTATGCATATTTATTATGACTCTTGGAAGACCGTAGCGTATTTATTGGCCTCCCCTTTTGGCTTGTAGTTTCATACCATTTGGCATCTTTTCATTGGCTTTATGGGTGGCGAGTGGCCTATATATAGTGTGGCCTCCACCCCCTCTCATTTCAGAATCTACTTTGTATTCTCAAGTTGAAGTAATAGAATAGCTTTCTTCTTATCTCTCTCTTGTACTTAGTCTAGGGCGAGTGAGGTCCTAACTTGTCGAGTAGAGAGGCAGGCTTGCTCACTTCGCGAAGGAAGGCGAGCGCCGCACGGGCTTTTGCGAGCAAATTCGTTAAGTCCGTGACAAGTAGCTCCTTTTGAAGATTCAAAAATCAAAAAGCACGCTAGGAGATGAAAACCTACGGCGTAGAAGTATCACGCATGATGCTTTTCCAGTATAAAGATTCCAACAATTATTTTTTATGATTGGACGAGCGCAAATTAAAGTTAGTTCCAATGGTTTTTGTGAATATCTAAAGCAGCAACATTTTACTCCTTGGAAAAGGCATTCAAAAGCAGAACCATTTGATATAAGGACTGAAGGGCAAAGAACAGAAAAATGAAAGAAAATGATAATACAAAAAAACACACCTACATCATAATACAAATGCAAAACCATTTGGTTATGCAGGTCCGAGAACATGAAAGCGCAAGTCTCTCTATTTGTTTGTCCATGTCAAAGCAGGTTAATACTTTTATAAGTAATAAGCACTCAGAAGGCCCTTTCTTGGCCAATGACTGAGAAGGGATTTAAAAATAATATAATAATAATAATAAAGAAGAGGAAGAAGAAGATAGAGAAAGTAGAGGGAAGAATTTTTTTTTTAGCCCGAAGGGTATTTCCTTCCACTGTTAAGTGGTAAAGAGCTTTTATCCTGCTGTGATTTAACAAAACTGAAAAATATAAATCTCAACAGAGTCGACTGATGTAATACCCGACACTGAAAACCCCAAAATTCCCAGCCAAAACAAGTCTTGGCCTGCAATGAAGAATCAATCGAACTTAGCCATCGACGCCAACATAAAGTTCTACTTTGTTCAAACAAGAATTGGATTTGTCGCATAG

>Aco021068; *Ac*bHLH50
TCATCTTTGAAAGGTTCTCTATTCTCTCTCTACTTCCAACCTTTCTCAGACTACGGAGACGGTGGATTTTCTGGGGAGATGTGATTATTTATCCGAAGATTTGCTCATCTTTAATTGCTAAAAGGAGTTTCTACTTTGCTATATTCTCAAACTGAAGTAGCCAAGATAAGGACACCAGGATTTTAGATGGACAATTTCATTGATCCATTCCTTTCTAGTTCATCCTGGGTTGGCAATGCTACTTCATTGGAAGCATATGCAGGAAATGAAAGAAACTCATCTGAATTCATGACACCTTCGAGCCATATCGTAGTGAATGGGTCTGCTGAAGACCTAAAAATTCATGAGCACAACAGAACCTCATCAATATTTACAAATGGAAATATAAAGTACGAGCTTGATAAGAGCCTATTTTCTGGTGGGGTCGAGTCTAATTCAACACCTGTCTCATTTGGCCTTGAATTGGATATGCCTCATAGTGGAGTTGCCATTTCCAGTTCAATTGAGAGCAATGGTAGCGAATTCTCTGCCTTTCCGCAATCTCTCACCGATGCACACTCTATTTCTTCGCCTTCGGTTATGTGGCCTTCTTCATATGTGAACATTTCCTCACTCATCTTACAAAGAAAACCACAAACTTTTGGTCTTCAAGAGCTGGAAAATGATAATATATTGAGGAACACATGCGATGAGAATGGGAAGTTCTCTCAGCTGGAAAATTTGCCGTTGCGATTCATACATGTAAATTTCACAAACTGGACTAGTATTTACAATCTCTCTTGCAACTGAAATGATTGACATTTGGCTAAATAATGATGCAGGATAAAAATGAGTTCCATAGCAGTCATCTGCCTTCTTTCACTGCTGGGCCACAGGTGCAATTTACTAATGGTGGTTCGCAGACTCAAGAGCAGGTTCTAAATTGATAGAAATGTCTTTTTTTCCCTTTTTTGTTTAAGAATCAAGTTTTCATTTTTAAGGTGTATGGATCGTTTAATTCTGCAGGAACAAAATGGAGTGCATAAACTTCATATGCCCTCTTTTGCTTCTGGGCCTCAAATGACTTTTAGCAATACAGGGTTGGCTCAGAATTCTCAACAGGTTTTATTCTCTCTTGATGTTCAGGAGCTCTTACATAACCTTGCAAAATCATCTATCTGTATTAAAATCAGAATTTTCAAATATAGCCCTCGAAGTTCACCATAAAATAATATTTCTAATTTGAATAGTCGTTCCATTGATGTGGGAGGTTATGTATTGGAAGTTCTTTCTAAGAAGGGATTGTTAGTACTGTGTTATATATATGTAAGAAATTTGGTTTCTGGTGGGCATTTGTGATTATTCAGGTTTTGCAGTTTGTAAATAGATAATTTTGCAATTTGCAGGGATGTGTATGTAAATATTTTTTAGTTTATTTCCGTTATCCTTTTACTTGTACATTTGATAAGCTGCAATTTTTCTGCCTTCGTTCAGTTGACGCAATCAATTGAAGGAAATACTAGCAAACTTCATATAAATCATTCATCAAATAGTCAATCCCAATTAGCTCCAGTGAATGAAAATGGTTGCAATGGAGCTGTCAAACCACGCGTTAGAGCTCGCCGTGGTCAGGCCACTGACCCTCATAGCATTGCAGAGAGGGTGAGTTGATGATTTTTTTTTTTTTCCCATCATCTTATTATGTACACCTCTTTACTTGATGATGATGATACATATTCTTCTGCAAGGTTTGCTTACTGTAGTTTCTTTTATTGGTCATATTTATGTTTCTTTATTAATTTCCTATACTGTTTGGAAAGATAAGGCTTCTTATTTGGAAGTTACTCACAAAATGCAGCTTCGAAGAGAAAAAATTGCTGAAAGAATGAAGAATCTCCAAGAACTTGTTCCAAATTCGAACAAGGTAGGTTCATGCTTCCTTTCACATCATGTTAGTGCCGCAGTGTATTGACTTCCTTGAATGTTAACACTTCTCCTCGTAATGTGCCTAGAGCTATTTTTCAATTTTTGAGGGCCAAATATCTTGGTGTGTGTTATCTCAGAAGTGTAACTTTTACTCTCCAATCGTTGGTCTCAAGAGAATTTCAATTTGCTGCTATTTAAGGTTGTGTTTGACCGGAAAGAGAAAGGATTTGTACTTGAGCCAATTACAAATTTTTCTTTGGTCTTCGTATATACTAGTTTGGTTTTCTCCTAGATATAAACTGCTGCAGGGGATATCTAACCAATCTTTACAATTTGTTCTCTGGTTGATTTGTTCGATTTCAGACAAAAAAAAAGAAATTTTCTCTGTTTTGATAGATTTGAAGAAAGTCATATCGGTCATCTGTAAGTTGCTCTCATCATCACCATCATCATCATCTAAGTCTTTTTCAAGTGTCATTTCACCGATGAGAAGGCTCTCGAGGAACCCAGAGTTTTAGTCAATGATTAGGTACCAAGTAGACATCAACTATGTCGGCATTTGTGTCAAATTCTTGGTTGAGGACTTGAATTAAAATTAGAAAAGAATATTTAATACAGAAGGTTAGTAAGCTGATAAATAGCTATTTTCCAGATTGTTTTGCCTAGTCAAGTTATCGACTTATCATCTAGATGAAGGAGAAACATGGCAATTTTAGGTTTAGTGATATTTCTGGACTGCTGGCAAAACTTCAGGCAATATGTTAAATGTAAAAGTCCGGTTCTTTATTTGCATCTTCACTTAATTTTTTGCCCTTAAATTTCTGGTGAATGATGACTTCTCTGCATGCCTTCTGAATTTCTTTCGGTTTCTTGAGCAGACGGATAAGGCATCAATGCTGGATGAGATAATTGACTATGTGAAATTTCTTCAGCTGCAAGTGAAGGTTTGCATGCACAGACAATTTTAAGAGGAAAGTAACAAATATTTTTCTCAAAAACTGACATTCTTTTTAATGGCTTGACTCAAGGTCTTAAGCATGAGCCGCTTGGGTGCAACTGGAGCAGTTGTTCCACTTCTTACAGACTCTCAAGCTGAGGTATATCATCTTATTATTTTTCTTCTTTTCTATGTATATTACCAGAGAAACATCAGCTAGTTGTTAGTTGCTTATCTGATGTTATTTTCATGTAACTGGATCTTACTACTGTACTTCATTAGTTGTTAGTTGCTTATCTGATGTTATTTTCATGTAACTGGATCTTACTACCGTACTTCATACCTTGATTACTTTTCGTAGAAAATATTGTGCTACTATACATCTGCTCTTTCAAAACTATTTTTGGCTGCGCTGTTGATTGGCTATGTACATCTGCTCTCGAGATGCTTTCGTAGTGCAAAGGACATTGTAAATGAGCAAGGTAATGAGCGCTGAAATGAATGCTGCCGAAACTCTTGTAAAATTGTGATTGGAGTTTTAACAGATAATCCTAATCGTTCAGCTATGTACTTGTTTTATTTTGCACCATGAACTCATAATTTTCTTCGATGTCTTCATATCATATTACTATAGTGAAAATATCATTCTCTAAATTTTATTTTTTAACAACAAACAGTTGGGAAATGTTTTTTTTGTATTTGCGGATTGTAAAACATTTTATTTATATCATATGCTCCTTTTTTTTGTGTGGCAGTGCTCCGGTAATTTGCTCATTTCTTCTCCATCTATTAGCGCCACTGGGCCCGATTTTGCTGATTCGCAGGACACCTCGTCCCTTGAACAAGAAGTGGTGAAGCTGATGGAGTCAAATGTCACAAATGCGATGCAGTACCTTCAGAATAAAGGACTCTGCTTGATGCCCATTGCTCTCGCTGCTGCGATATCCAATCAGAAGGGATCGAATTCCTCAGCCATTCCCCCCGAAAGAAGGAAACCAAGCTTAGTTCATCCCGAGACTAATAATGCTGCAGAAGACAGAGCCAATGGTTTCGACGATGGCGACATGAGAAAAGAAGAAGTCGCAAAATCTGTGAACAATGGAAGAGAGTTGCAATCCAAGGCTTAGGGTCCGAACTGACTACATAATCTTCTGGTGAAAATGTATGCACCTTACCTAACTATAGCGCATTTTGAAACGGGTACCTGAATTTTAGAAAATTTGATTTTACTACCTGTTCCTGTGTATTGACTATCCGTAATCTAACAGGAAATTTCTGTTAATTGACAGAATATTCTTCAACTTTTCTTGACCAAATCTGCCAGTTCTAACGGGACTTAAATGGAATTTGTCTGAAATCAAATTTAGCCAAAGTTAGGTAGTAGAATCAACAATTTCAACTTCAGGTACCCAGTTCAAAATACGCTAAAGCTGAGGTAAGGTCGTTTACTTTTTTTGTTTGATCTTTTCCTTCTTTGTACTAATTTGTTTGCTGATTGAGGAGACTTTTTCTTTAGCTGTTGTAATGTAAGTATGTAAGGCTGTAGATGTTGCGCTTTAAAGGTTGGTGAGTTTGAGTTCATTTTATCTGAGAATTAGGTGCTAATTGACTGATGATGATGAGCTCATTCAGATCTCTACGTCGGTATTCTTCTACGGACTTTATTTGTTGTGTGTAGAATATAAATAAATAATCATGGTATTGTCAAAATGCATGAGCTCGGT

>Aco004914; *Ac*bHLH51
ATTTTAGTAAGCTTGAATTCATTAATTTACAAGTGAAGTTAATTTTCTTCACCTAAAGCTTATAATGATTTATCTTATTACTTTAATATATAACTATTATATAAACAAAGAAATATGTGCTCAACCTTTAAAGAAAATCAAGTGGGCCCGACAATTTAGCAAAAGGAGAGAAACAGAGTTTTGTCAAAGGTGTGTTTGTAGCATATATTATTCTTCTCCATGTTGTTCGCACTAATTGTACTTCCAGTAATACAATAATATTGTCGGCATAAGTTCGCACTAATTGTACTTTCAGTAATACAATAATATTGTAGGTGTAAGCATTTTACTATTAAAATAAAATGCAAAGGAATTTTGTTTCAGATTTTTGGAATTGCAACTTCCTTTCATATTCATTTCATAGCTAGGGATATACACCTTGGAGGTTGAAGTTTGAAGTACTTTCACATTTATTTATTGCCTCAACTTCATTTCTCTCTGTGTATCTCTAGTGCTTTTGCTTCCCTAGATTTTTATTGTAAACACTCAACTTATGTCAGCTTATTCCAAACAAACCATGATTATTTTATTATTATTTATTTTCACTTTGGTTGTGACTTGTGAATTTATGGATTAACAAATGGTAGATATGATAAACCTTTGCCTCTTCTGTTCTGTTTAAGTTTTTGAGGTGGTGTAATTTTCCTTACAGTTGATTATGCTGTCCATTCTTTTGTACATGTCATGCTACATTTTCCATCTGTTTTATTTTACAAAATTTTATTGAAATTTTTCTTTTTGGTAGCTAAATTTGGTGTTAAGACAGCTTTTTTTAGCGTCTCAAAAAAAAAAATCCTAGTTTTTATTTCATAACAATTATGATTTCTGATTGACTTGCTGTGCACTTACCTTCCCCTGTATTCTGTATTTTGACTGCTCAGACAGATGGGTTTAGTATATTGATGCATATTAATTACTCATGGTTGATTATATTATGGTTTTATTGTTATGTTTTCTTCTTATAAGACATGCCTGGGATTCAGCTAACATACATATTGCTCTTCATCAGCAAAGAAATAATTATTCAAGCACACTAAACTGTTGTCATTTGCCTTAAAGTCAACGAATGAACTCCGTATATGATACTAATTAACAACTAATGTCAATGAGAAGTGGCATAATATATTCAATATTTACAGAAGTTGGAACATCTAAGTTTTCTTCCATGTGAAGTGAAAATTTAAAGCCCACCAGCAATGCAAATCTGGTCATAAAAAAGAAAAGAAAAGAAAAAAGAATAAGTCCTTTCTGAAAGTCCAGGAAATTTGTGAAAAACCACCACTGCGTCTTAAGATATTGATAATCTCCAACATCGGATACCTAATCAGGTAACCCACGTGTGGCTTAACAATTGGCATACATATTATAATAATTAGCAGAACTTGATGTTTAATAGCATAGTTTGTTAGAATAATTTGATACACTAATTATTTGCTGAGTTTATTAGAGGATTTTGGTTTATTAATTATTTGAAAGAATTTTTAGTCCTCAGAAGTTGTCCTTTGGGATATTATTTAGGGGTTAACAGCTTTTACAAGCCAGCCGGCGGATCAGAGCCAATAAGGATGGACTTTTGAAAAACCCAGAATCAAGGGGAAAAAAAAATGGTAAAAGGAATAGTCTCAGTTCAATCCCCTCTCTTGTATTCTTTTTTGTGGTAGTAGATTAGAGGTTCATGTATTCTCAACAACCAATGTGGAATTGCTGATAAAAAACTCTCTGGCTTAGTTTATGTTATATCATATATACAGCTAAAAGGTAAGAAAATATAAGGAATCTTATCTTAAGACCTTTACATCATGCAGTGAACCGGCGAAGATATTGCCGACATCTTAGTTCATCTCCTTGGTTATTTTATTTGTTCTTGAGTCCTTTCGGAAGAATGGGAGAAGAAGAGGTCCTAAATCTTTATCACATTGTTGTAATCTAAATCATAGTCGTTTGTAGTCTGTTTGTTTTAAAAGCTCCGTATTTAGGGAATTTGTCTGTCACTTTCTACTAATATTGTGATCATCATCATTGAAGAGAGTGGTAACCAGTTTCAAGATCCGAGAACTTATTTAACGCAAATCTTACATTCCAATGCTTTGGAATCCAAAAGTTATAAAAAAGTTCTCATTAATTTTAATGAGCATTACGACAGATTGGTTTTCATTTTCTTGATTCTTCTTGCTGGATTAGTTACATCATTCCTTTAAAAGGTTGCATTAGTTACATCTTATTATGTCAAGGAGAAATGGACTAAACCAATAGTTTCTCGCTTTTGCAATAATTACATTTTTTTTTTCATGTTTTAATTGTAAAACACGGTAATTCCCTCTGCAGTTCATCGAAGGGATAAGAACATCTTATTTTTTCAAAAATGGGACCATGAATTTGTACTCTTTTAAGTTTTAATAAGAATTATTATTCCTTTCTTCATTCATAGTAACCCACAATAGGCATCACCATTGCTAATAGCTTCTTGCAACTACCAAGTATGTGATGATAACTTTTGAAGGCCAACTCAGCAAATTTTGTTTCTGCTACTAATGATTGTACTCAAAAACAGGAGAAGAAGCCTAATTGAGGCTGTGAGCAGAAGCAATGGAACTATGGGTAGGTGAATAGACCCTCCCTATGGGGAGACAAGCACCGTGATCACCGGATATGCTACCCAATTTTTTCTCAGGTACTGTAAGGAATTGCAGAACCTCTCCCCACGAACATGCAGACCTCTCTCTCTGTGCTCGTTTTCTTTATTCCCACCAACAGAATTCCTCTACAAATTCATTCTTTTTTCTTGATCGCCAGATCATTGACTTCTCACTCTTTCTATCCAATCCTCTCTTTAAAGTTATTCTGGGTTCACACTAATCATGAATGAGATCATATTCCAATCATTTAGCCAACATCTAGGTAAGTGGGTTTTGGGAATTAGTCATCATGTTATCTACCCACAAGAAGTTCTAAATTTAATTATTTCTGAATATTTTATTCATATCATTGCAGATACTCAGAGCCCAATGAGTTGTATCGCCAATTCAAACCTGCCGTGGACTCGAAATCTTTAATAGTGATGAATAAAGAATTCACAACTATTGTTGAGGTGAGTGCAAGTCAAACTTGCTAACTTTTGCAACATTGTCCAAGGAGATGCATATAAATAACCAATGAAGAGCTTGCCTATTGAATTATCAAGCTAATACTTGACAACAACACTTCTCGAACCAAAGTTACCTCCCTCCAATCCAATGTCTTATGAAATGGAGCCTACAAAAGTGGTTTCAGAGATCAATTGGAGCTCATACAGTCCGTCAATGCAATCTGAGTCTGAAATAATGGCCCAACTGCTTGCCCCATTCCCTTTTCAGTGTGAGCCAGATAACCCAGATTTGGGCTTCGGAGCCCCATCATTCTTCTGGTCTGGTCATGCTACCGAGTCATACTATTGTTCTGAGAATTCCAACCCTAATGTGTACTATCTGTCTCAAGGAGAAGTTAGTCTTAGTGGTAGTACTAGTAATAGTAGTTTCATTCTACCCTCTTCTGTTTATGAGAGCTGTTATGTAAATGGCTCGAATGTGGCCCTCGGGATCAATACTTGCTCGGAACCAATAGATTTAAACCCTTTATATGGTGAAGAATTCATTGACCTCTCTGAACAGACGACTCAACCCAAAAGAAAGTTTCCTAGTGTTGAGGAAGAGAACTTTGTGGATCATGGCGAATTTGACAGCACTGCTGTGAGTACTAAGAAGAAGGCTCGGGATTCTGAGAAAGTAAGTATAAGGATATTCAACCTTATGTCTAAGTGCTTTTAGACCATTTAATACATTTTATGTCAATATTTAATGGTATGGTGAAAATGCAGGTTCAAAGTGCAAAGAAGGGAGAAGTGAAGAGAACTGCCAATAGCATTCAAAGCGGAGATGATGAGGCTTTGCATGGGCAAAGCTGCAGTAGCTACGGCTCTGATAATGATTCAAATGCTTCTCAAGAGATGAATGGAGGTGGGAGCACAAGTTCCTGCTCTAAAGGGTCTTCTGTCCTTAACTTGAGCGGCAAAACTAGGGCTAATCGTGGGTCGGCAACCGACCCTCAAAGCCTCTATGCAAGGGTACTGGTTTTTATCTCTATCACTATAAAGTTAGATTTGCTAGCTCTTCAATTATCATGTTCTCAAGCTGTTATTATTTGAATGCAGAAAAGAAGGGAAAGAATCAATGAGAGGCTGAGAATATTACAAAATCTTGTTCCTAATGGAACCAAAGTAAGCGTTTAAGGTGACCTTTTGGTGCAGTCAAAATTGTTTAACAAGAAATGTGGATGGCTAAAGGATGGTTTTGTCTTTACAGGTTGATATCAGCACAATGCTCGAAGAAGCTGTCAATTATGTCAAGTTCTTGCAACTACAAATTAAGGTAAGAGTTTATTTCATACTCCTATGTGAAATTCTATTGCATCATAAGTTCGCGAAGAGAGTGCAATACTGATGTGAATCTAATCTTTGTTTATTTTTAATACAGCTCTTGAGCTCGGATGAACTATGGATGTATGCTCCTATAGCTTACAATGGGATGAACATTGGGATTGATTTAAAAATGTCTCCATCTCAGCAATGAAGGTGAAATCTGTGTACTGAATGGAGAAAAAGGCGGCGGGAGGAGCTCCATTGTAAATAAGTCTTCTTACACATCACCTTAAGGAACAACTGCTTGAATGTAATGGGGTTATCATCAATAAGCCACTCTGGGTGGTAATCCTTAAGAATACATGATTCGGATCAAATTCTTTTCCTTTTACTTTTCCCAAAAAAGTTACAGTAGAGAACTGGTTGATCACCTGTATAGATTTGTATCAGATGAAGTCCTCAAATTTACACATGAAATGTGTGTAACAAGGAAAGGCAGTTTGTAATAGGCCTCATATATACTAGATTACTGTGATTTCCTTCTCCAGTAAAATTTAGCATTTTGACTCACATGCTTTTGTTTAGCTACTTGTTTCACATGAAGGCAATGTGGCATACAATATATATATACGGTCCAATCTACAAGTTGGCATTACATGTCTTTGGAGATCAATAAAGC

>Aco014442; *Ac*bHLH52
AAGCAGTCACCAATTAATAAGCCAAGAGCAATTTTGTTCATTGTAAAAAAGAGATAACAAGGGAATAAAGAAGAGGAGAAGAACATGGCAGATGATCTTGACGCAAGGATATGCAGAGGAGGGAGCTGGTGGTTCAATCCCGGCCCGAGCAGCAGCGGCGGTGGTGGCGGCTTCGTCGACGCGCCAGGCTCCGTCTCGTGCTCAACAGCTATCGCAGATGCCCCCCGAGGATCTGCTCTTCCTTCTGATCATGTCGGCTCGTCGTTCGCCGACTCCTTCTCCCTCTCCGCTCACTTCATGGATTGGGGGCATGATCAATTGTAAGTCCCTCGATCTCTCTTCTATCATCAAATGAACACACACATGTATATATTAATTTATGCTTGATTCAACTTAATTGGTTGATTTTGATATGTGCAGAAGAAGCAGTGGAAAAGCTGAGAACAGCTTCCATGCCATGCTCCAAGAAGATATGAGCACAAGGCCCTACAATTTCCGGCAGGGTTCGATCGAGCCCGAGCTCGAACCAAGCATGAGCCAGAATTTCCTACTAGATCACCACCAACACCACCCGAGCTCGACCAACGATCCCGCGCCCGACTGTAGTGTCATCACTAGCTATGCCCTAGCTCCGACGGCCTACGGTTGCCCGCCGGTCATGCATCTGCAAACCCTACTCGAAGCTGATGCAAAGCCCCAGCAGTCGGTTTACTATGACCAGCCCACCGAAACTCCGATGGCCACGGCGTTCCACGGCACTAATTGGCCAGTATTGAAGGATCAAGAAGGTAGTAGTAATCCGCTGCACTTCTCCAATAACACTCCCTTTTGGAATGCATCGGCGACCGCTGTCAACGAAGCCAAGTCCGGGTTCTACCCTAATTCGGTGCCGTCGCAGTTCGTCATGCCGGCGTTTGAGCCGAAACTGCCCAGCTGCAGTAATTTCATGGTGAAGGTAAAGCCTATATATATATACTGCGGATTAAGATGCTACATATAGTTTGAGTTCTTGCTCAATGATTTTTTTTTTTTCCCTTTGGTTCTTAATTGTTCAGTCAAATCGAGATGCATCGATGCCAAAGAAGAGCGGAAGCGAAACTGCATTAAAAAAGCCCCGAGTGGAAGCTCCATCCCCGTTACCGACCTTCAAAGTACTTCATGAACTCATTTACAATTTAATCCATTTGAGTTCTAATTTTGTTTTCTTTGTTTGGTTCTAACTCATTTTAAGCATTAATCATGGTAGAGTTAATTTTGATCTTCTCTATAATTTTAGGTGAGGAAGGAAAAATTAGGGGACCGCATTACTGCACTTCAACAGCTAGTCTCACCTTTTGGAAAGGTAACATTTTTTTCTCTTTCTTCTTCTTCTTCTTCTTCTCTTTTATTTTCCTTTTGCCACACTCTCCATGAAAAATCTCTTCTTAAAGCATTAGCTACTAATGTTAGTTCTTCATCACTAAATTGCGCGATCTTCCATTTTTACAGACCGACACAGCATCGGTTCTACACGAAGCGATCGAATACATTAAGTTCCTCCACAATCAAGTCGGGGTAAGCATCTTCGATCTCTTTTCCCTAGCTAGATTCTTGTCATAAATTTTCAATTCACACTAAATGTCGCATATAAATGATTAGAGTACGTACCCTTCTCACGAGTCTTTTTCAGTTTAACCAACTAACTAGAGAACGGATCAGTGTTAATCATTTATCCTGAATACCAATTATTTAATGGTCTCCTGGTTATAAATTAACACATGTTCTTGATCCTCAGCTCCTAAGCACACCATATTTGAAGAATGGGCAACCCATGCAACAACAACAGGTATCATACACGCAAGTTCTCAAATCATCTTTAAAAATTAAATAGAGAAACTTTTTACATGCGTCATTCATTAATGTTTTTTATTTGCTTATTAACTCAGAGCGCGGAGAAGTCTAAAGAAGGCGATGAATCGAAGAAGGACCTCCGGAGCCGAGGATTGTGTCTTGTTCCAGTCCCGAGCACTTATCCGATGGCGAGCGAGGCCACGGTCGAGTTCTGGCACCCTACCTTTGGAGGAACATTCAGGTAGAAGAGAGTTAGGATAATTTGCGCCTTTTTTTTTCTTTTCTTTTTTTTCTCCAGTTAAGACTGAGAGGTACTAATTTATTAATGTATCTTCATAGCCATTATTGTTGAAGCTAAAGCAAGGTAGGAATGATAGGTTAGGAGGAAAGAGAAATATAAAAGAAGTTAACAACAGTGTAAGTGATTAACCCAGCTGAAGAAAAAGAAAGGGAGATGATCAATCCTTCAAAGACAGGCTATGTTGGGGCAGCTACAAGGGGGAATATATCAATATCTATATCTATATCTATATCTATATATATATATAGATACATGCATGATGTACTGAGGTGGCCATGGAGAATTAATAGAACTTTAGATGGAATTATTTATTATTAGTACCATGATTGCACTAGTAGTGTAGTAACTATTGTTAAGAGGTGGTG

>Aco014447; *Ac*bHLH53
ATGCCGCGGCGGCCGCGGGGCGCTGCTGCATCGCAGGCCGCCGAGGCGGAGGCGGAGGCGGAGGCGGAGTTAGAGGCGGAGCTGCAGGGCGACTTCGTCGACTCCGTGCTCGACATGGAGGGTGGCATCGGCGGGGAGACGCCGATGGAGAAGTGGGAGATGAGGTTCAAGTCGAAGAATCTGGAGGCCGAGAGGAGGAGGAGGGGGAAGCTCAACACCAACATCTTGGCCCTCCGTGCTATCGTGCCCAAAATCACCAAGGTTCAATTTTTTTTTTCTCTTTATTTCCCCTTTTTTCCAATTTTTTTTTTTAGTTATCTTTTTTTTTAATTATTATTAATGTAGTAAGGACCACCCCGCTTAATTATTATTAGGCTATTAATTTTGGAAATTATTTGTGTAACTTTGTTGTTTTTTTCTTTAATAAAAAAATATTTAATCGGAGTTGTTATAGATAGTTTAATTAAGGACTATATCAGACTTATTAATGGTTTTTTTATCTGTTAGTTGTGGATTATTGGTGGAACTTGTAGTCTTATTGTTTGCTAAAAAATGATTAAAAATGTTAAATTTAACAGCATTTTTATTAAATTTATTAAAATTTTTATGTAACTGATTTGAAATTGCTAAAATAAAAAAAAAAGTTGGGATGGGATGGTCTCTATATATTTTACAATTTTACAATAAACAATCTGGTAAAAGGAAAAAAAAAAAAAAAAAAAAAAAAAAAAACAACATGACAATCAACAGTTCCTAATGTAAGTGACAAAAAATTTAATGATTGGAACAAAAAGTTTTAAGTTCAAAGTTTAANAAAACTCTTCTTTGCTGTTGTTAAGGAATTGTTTTTGGTGATTTTAGATGAGCAAGGAATCGACCCTCACCGACGCGATCGATTACATTACGCTGCTGCAGAAGCAAGTGCTTGATCTACAAACAGAGCTCTTAGAAACAGATGGAGATCTTATTCATAAAAATGAAGCAAATGAAGATGAGGAGGGAGAGAAGCAGAGGAGTCCAAGTTCTGAAACAGTTGCTCCCTCTGTGACAGTGCAATGTCAGGTAATTTAGTTTTTTTATTTTTCAAGAAAATTAAAATGATAGATAAATTAAATATTTTGAGTTTCCAAAGTAGAAGTTTAAAAGTAAATTATTATGAAAAGCAATTGATATATTGGTTTTTTCTTTAAGAAGAGATTTTAATAGAAACATCCAATAAAAGGAGAAAAGAATAGGAGGTGATAGAGATTAAGCTAAAAAAATTTGTTAAACTTAAAATACTAAAAAAAAACACTTGGCGAGGATGGTCTCTAATGTGAGACCGTCACATTTGTCATTCATGTGGCTTTGAAATTCGTGTCATTCACGTGATTAATAGCAGATAGGATAGTCTTGAAATTGAGGACTGTTCCCATCAAATTTGTGTTGTTCTGTGTTTTGCTGTACATTTAGTATAATAGTGAAAGTATACAATGAAATGCATGGGGAGGTGGTTTGGATGTGGAGGATAAACATTTCAATGCTCAAAAAGAGGAAGAAGAAAAAAAAAGACAAAATAATTTTTTTGGTGTTGTAGCATTTTGTCTCCTAACCACTTTAGAAATGCTTGATACTCTTTGCCAACAGGGGCAGGTTGAGCTAATACCTATGGGCCCAAACAAGTACCAGCTGAAGATAATGTGCAAGAACAGGATGGGGCAGTTCACCAAAGTTCTAGAAGCTCTGAGCTGCTTCAATGCAGAGGTTGCTGAAATAAGCTCTGTGGCCTTCTTCGGCTTCTCGAAGAGCGTGTTCTCCGTTGAGGTGAGTCCACACAAACTAAAATATCCTTCTACTAGTGAAGTAAGTGATTAATTGTTTATGTTTTTCTTTTTTTTTTCTCTTTGTATAGGTGAAGGAAGGAGAAGAGGGTGAAATCATAGAATTAAGAAATCTTTTGTTAGCACTTGTTGGAGCCTCGGAAAATTGA

>Aco014454; *Ac*bHLH54
TATTTTTCACGCTTTTACCTCCACCAACCCATTACGAGTTCGATCCCCGACTCTCCTCTCCTCTCTCCCTTTACCCGATAAGAGCGCAACCCTCGTTCTCCGACGATCACCGGAGATTGGCCGGACATCACCGGCAGGGGATATAGGGCTCCGGCGAGGCACCGGGGATGGGTTCCTCCGAGAACGCTAATTGGGTCTTCGATTGCCCCCTCCTAGACGACGTGGCCGTCGCTGGCGACGATTTCCCGGCGTCGGGGAGCGGATTCTACTGGGGATCGCAGGGAATCAACGGATCCTCCAATGGCGGGTACGATCGGATCCGAGATCCCTTTCTGAATCGCTTTGATTGATGTCGTCGATTAGGGCTAGGGTTTCGCTGCTCCTCATCAGTAATTTCCTGTAGATTATTGCTCATGCTATTCCGGATTGCATGTATACGTTGATTTGTGTTAGGTTTCGTCTATTGCTTGCTTTTATTTGTGTTACAAATTAGGATTTGTATTTAGTAAGTTACTGGATGTTGCAACTTCTCCAATTGGTTAAGGTACTTCATAGAATAATCTTGAGTAATATAGGGGAAAATTGTATTGGAGATTTGAGGATGCTTTGGAGGTGCATAGATAATGGAAGAATCTCATAATTGTGTATTATTAGAGTCTTAAGTAATCATATGTTACAAGATGTTTGGTTCTGAAGATTCTACTTTTATGCTTTACCTTAACACTTTTGTTGATTATAACAGAGAAAAAAAGTGATGTTGAGATTATTTTTGCCGCTTGATTCTGCATGTGTAATGGTTTTATAGTGTTTGGAACTTATATCAGTTTTGGTATTCCCCATCAATTATATATACTCGTCTTTATTTGCTCTCTTTTTAGTCCAGAAGAACGCTTACAAAGAGTGAGTTAGCCAGTACAATTTCGTCTAAGTACTCCAATTGAATGCCATTTATTTGTAGGCATATGGGATTCCATTTGATTTTATAAATAAATTTTCATAATTATGGGTGAAACTTCGTCCACAATTTCTCAAAGTTAGGAAAGCTGACTTAGATCTAACTAAGCTAAATTACATAGATCAATTAAGGTTTACTAGCTTTACTATAATGATCCTATGCTCCTAAATCCACTCATTGCAAATTCAAGACTCTATTGGCTCACATAGGGCAGATTCAGACCCGAATTAATGAATCAACTAAACTATACTCAACTGGAGAAAAATACATACCAGACTAGTTTGATAATGGATTCAATCTGACCGATTACATCTAGTCCTGGTCCTCCTAAACGGGCGATGTTTTGACAGTTGCAGCCGGCCAGAGAGGGAATAAGAAGAGAGGAGTAGGCAACTAAGAGAGGAGGATAGAGGAGGGTGGAAATTGCAACATGTGGCGAAATGATGAGTTCCCTCTTCTGTGTATTTAGAATTTATATATGATTGTTTATAAATGTAGAAATAGGATAGGAATGATACACATATATGGATAAGATGGACTTGTAGTCGTGCATGCTATAATAGGTTTTATCACATTTTATGACATTTACTTAGCTGACCTATATGTGTATAGGAATGATATACATATATGGTTAAGATGGACTTGTAGTCTTGTTCAACCCTATTTGGAAATCAAAGGGATACCCTTTATGCTGATTCTTATATTACACTTTAGTTTTTTGATCGTTGCTCTTTTGTATTAGTTAATTGGAATTACCAGATTATTGAAGCATGTTCTTATTCTTTTCAGGGTTGAAATAAGTGGCTCCTTTTTGGACTCTGAATGTGTCAAAGAACGAGGCTCCAATAAACGGTTTGGTTTTAAAAATTGATATGATTTTAAGCTGACTTTTTTTTTGTCTGTTACTAATTTGTATCTTTGTTGCAGTGTTAGGTCTGAAAGCTGTCAGCCAAGTTCCAAAGCTTGTAGAGAGAAAATGAGAAGGGATAAGCTGAACGATAAGTATGTGCTCCACTTTTACATCTCTTTTGCTGACCAATGTTGTGCTGGCTCTTTTTTCTTTTTACATTTTTTTTTTTGGGTTAACTTTCACTTTAAGGTTCTTGGAGTTGGGCTCCATTTTGGACCCAGGTAAGCCACCAAAAATGGATAAAGCAGCTATTTTAAGTGATGCAGTCCGTATGGTGACCGAGTTACGTAGTGAAGCACGAAAGCTGAAAGAGTCAAATGAGAGTCTCCAAGAAAAGATCAAAGAGCTAAAGGTTTATCTTTAATGCTTGTGTCATTTATTTTCTAAAGTGTCATTTTCTTAGGAAAGATTAGATTGAACTTCTTGTTTTGATCCTTGACTGCCATGCATTGAAGTTTAACATTTGCCATACTCTGTTTTCTGTGGTTGTATATTTCTTCACATGGTATGCAATTGCTATATTGAGTTTGGGTGATTCATTTATTTGATTTCCAAAGCAGTCAACTTCAGGTTTTCACTCATGTTTCCCTCCATGTCAATTCCTTGCTGCAATTTTTTAAAGTTCTTATCTTACCTGCCAGTGTATCGATTAGCTATGATTGTTCTTTTTAACACTGCACGCCGATTCCAACTTTCTCACAGGCCGAGAAGAATGAGCTTCGCGACGAGAAGCAGAGGCTGAAGGCTGAGAAAGAGAGCCTGGAGCAGCAAATAAAGATCATAAACTCTCGTCCGAGCTACATGCCGCACCCTCCTGTCATGCCGGCCGCATTTGCTGCCCCGGGTCAATCGGCAGGTCACAAGCTGGTGATGCCTCTCATCGGCTACCCGGGATATCCTATGTGGCAGTTCATGCCCCCTGCAGATGTCGACACCTCGCAGGACGCCGAGTCCTGCCCTCCAGTGGCTTAAGATCATCATTGTTCAATCCATTTCTCATGCTAAATTTCTCTCGAACTAATTTCCTCATGTTGAGGAAAAAAGAACTCTGAATTTCTCTCTCCAAGCTTAAGCTGTGTCATTTTTATAACTTTGAATTTAAGTTGTCTGGATTGTCTTGTAATATCTTTATGAATAAATTCAGTGTATGCCAGACGTAGATTTGTCCAGTGAATAACTTTAGATCACTGGGATTATTATTATTTTTTATTTTTTTATTTTTATTTTAATTTTTCCCTGTGGCTAGTATGGTAGGAGATGTCAA

>Aco020380; *Ac*bHLH55
CTCTCTCTCTCTCTCTCTCTTTCTCAGTAACAGTGTCAGTCCCAAGCTCACGCTTCTTCTTTCAGATCTACTCTCACGCTCTCTTATTCGAAGCTTCGAATCCTCGCAATAGAGAGATCAAACCCTAGCTATGTGAAGTGGGATCGAGCTCCGAGATGTGGCAACCGCTCCCTATGATCGGGGTCCCGGTCGCCGGGCCCGGGGCGGAGAGGGGCGCGGGAACTCTTTTTGCGGCCGCCGCCGGGGGCGTCCGGAACCGGGACCATTCGGTGGACGAGTCGAGCGTCACGGAGAGTAGTGGCGGGAGCCGCGGGGCCGCGCGGCGGCGGCGGAGGGACTCCGCGGCGGCGGAGGGCGAGTCGCCGAAGCTCGCCTCGGCGACGAGCGGCGGCGGCAATGGCTTGGTACCCTCTTCCTCCCCTCGCTCCTGGTTTGGCTAGGGTTTGGTTTCTTGTAGTGATTTGTATGTGTGATTGGAGAGGGTTGGGGTTAGGGTAAGAAATTAAGCCTCTTGTTGATTTGTTAAAATTGAGGTGAAAAACAGTTGTTTTGGAGGGTTTCTTGGGGTAATACTCTGGTGGAGTAGGGAAAAATGTGAACTTTACAATTGGCTAAAGGTGTTAATTTGTCCAAATGTTACTTTTGTAGTTTCGAAGTTCTTGAACTATACTCAGAAGCTGTTTGGTTGTGCTTAGTTGCACTTGAACCGCTGTCGTCGCCGCATGCAGATCCAAATCTAGGGTTTGGTATGATGCATTTAAATTCAACTACTGGAGATTTGGAGAGGGCAACTTTAAACAAAAGAGAAAACCTTACTTTCTAATAACTTTTTTAATTTAAATTACCTTTTTCTAATAAAATAGGGAGTAATCGAACCATCCCGTATATAAATAAAAGTTTGTGAACGTACTTTTTCAGCTGCTTACAACCGAACAGCTTATTTATAGTTGTAGTGCTTTCTGTTACATCCAACCGAACAAGATGCATGTAGTTATAACTGTTGTATTTCCATCTGTACAGATTTCTAACTACACTATATTTACAATTACATTCAACCAACTGGCTCCTTAGTGTGTTTGATTGCTTGTGTTAGTTTTTAGGGTAAATTACAAAGAAACCCTCTCAAGCTTTAGCCCGCGTTTCAATTACCTTTACATGAAAAATTGCATTAATTTCAACCTTGTACTTTATTAATGTCGTAAGTAGGTCTAACAACTCGAATAATTGTAGAATATGGATGCAAAATATCACAGAAATGAGTTGATGGATGATGCTGGTTATACTTAATAGAGTACAATCATGGAAATTTTATAAATCGGTTAATAAACTGCTGATTTCGGTATCTCTTGTAAAGAAAGAGATCTTTTTTCGCTAGTTTTAGATCCATTATTTATTTGCAACTTAGATAGATGTCCAAATATAAATTCGAGCGATATACTGCATTCTAGCGAAGTTTTGCATCCAAATTCAATAGTCTTTCCAAATTTTTGGCTGATTTGAGTTGACTACCTCAAACAAGGTTTGAAATTTTATCTACTGTATCTGTTTTGATGATGGCTCTCAGTGCTTCAGTTTCAGTAGTTTTGGCTGTTTCCAGTGTCATCTTCGGATCTGGACAAAAAAGTTTCTTAAGTTTTTCAGCAAAGAGAAAATAATCTGGAAAATTGAAATAACAAAAATAAGGCATACTTTCTCAACGTTGTAATACATTATCAGCGTGCTTGATCTATCATAATTGATTCCTCAACATTTTCAAAAGCTTGGCTTGTTTCTATTGGTGTGCCCCATATTGGTTTAGTTTAATCAGTGGTTTCAATCTGCTGAAATAGTGTATCTCTAAGTTGTAGCTTAATTGATATACTGTTGGGTTTTTATGTTTAATAGAAGGCAATTTGGTGCTTTCTTTAGGGATATGATAGTATGCTAAACAATTTTCTATTTTTTGGAGCCCTTGCATAAAAGTAAAATCATTAAAAAAAAAGTGCGATGATTCATGTAAGAATTGCTTCTAATGTTGAAAAATGGTTGAACTGAAAGCTCGTTGAAATACTTTGCAGACTAATTCTGAAGGAAAACGGCTCAAGGGCGTAAACTCTGTTGATAATAATGTCAATGTGAAAGGTGCCGCTGAAGCAAGTTCTGGAATGGCTAATAAGCAGATGGATCAAATCGCTCCACCGCCTGAGCCACCCAAGCAAGATTACATCCACGTCAGGGCGAGAAGGGGGCAAGCAACAGATAGTCATAGTCTTGCAGAAAGAGTAAGGTTTTCTTTGAAGTTACAACTTAAACTCCGTTATTCCCTACCTCCTTGGTATCCATGATTTGTAGCTTACGAGCTGTTAAAATAAAATTAATCTTCTTTTTCCATTGAAACCCCAGAAGCAGAGGTTTCAAATAGAAGCTTTTGCTTTCTGAGCTCACAAGCTCACTTCAGCTTCTAGCCACAGCAAAAGCTCTGGCTGTTGCTTCCCCATGAAGCTAGACAAAAGAGGCACGAAATTTTTTTTTTGAAATGTGTTCCTATTTACTTGGTAGAAAGTTGGTCACAAAATTTGCAGCATTCTGAGGTTTCTTCGCTCCATAATTTTAATTATATTACTGGAATTTCTTTATTGGCAGGCAAGGAGAGAAAAAATAAGTGAACGGATGAAAACTCTCCAAGATCTAGTCCCTGGATGCAATAAGGTGCATATTCTATATTGAAATTTCCACATTATTTTTTGTAAAAAAATTTCAGATATCATGTAGTATTGCATCGGATCAACTATTTTCAACTCATGCTGAAGGTCGCAATTTAATGTCTCTTCTAGTTTCTGGAGCTACTTTTCGTTGAATGTTGCGCTCTGACGTTGTGCTTTCATGCGTGTTCTCACAGGTGATTGGTAAAGCATCAGTTCTGGATGAAATAATCAATTACATTCAGGCCTTACAATGCCAGGTTGAGGTAGATACCTTTCCGGCAGTTGGCATTCTTTGGCATTGCCTTCAAAAGTAGTTCAAAAATAATTCCTTTTTGCTGCACATTTCAGTTTTTGTCGATGAAGCTAGAAGCAGTCAATTCAAGCATGGACACCGGCATTGAAGCATTCCCGCCAAAAGATGTAAGTTTTTGTGGAAAGTTTCACCATGGGTATAACATAATTATAGTTCCTACCATGATTACGTATCCTGGGTTAATCGATGATGGATTATTGTACTTTCACGGCAAAATCGTCGTGAAACATTTTGCAGTTCGGCCCTCAAACATTCAATGCTTCTTCTAGTTTGCCATTTAGCCTAGAGACACCAAGGGAGTATGGACAGGGTTCGGCAACGGAATGGCTTCATATGCAGGTTGGTGGTGCTCTTGATAGAGTGACATAACCGTAGAAGAAAATCCCCATTCATGTGAAATAAGAATTGCATACCAGGATAACTCAACTCCATGGTTGTAGGGTCTTGTATTTCAAAATCACTCCAAAGTTCTTTTGTTCAGAAGGTGATTTTATTTCTATTTTGGAGCTTTTCTGTTCATTTTGCCACGCCTTTTAAGGAATTAGCTGCTTACTTTCCTCTTGCCATTTGGAGTGTCTAATATATTCGCTCAAACCGGATTATGCGACAACTTTTAGGTTAAATATCGAAAACCATCCCTGCACTTTCCTTCCTATTTCATTTACCCTCTTTCATTTTCAATCTATTTGGGAGTCAATCGTGCATTTTACTCTCACAGTTCAAG

>Aco020381; *Ac*bHLH56
ATGAGCGAGCTACTCTTCTGCAGCGAAGGGACCGCCAGACTGAACTGCTTCGGCGGACCGCAGTTTTCGCCGGAGCCCAACTTCGATTGCCTCAGCGAAGTCTGCAGCCCGTTTGCTGCCGCCGGCCTGTGCGGCTCGTCGGCACCGCCGGCGGCGGCGGCGGCGGCGGCGGCGGCGGCGCATGCTGCCATCCACGAGGAGATGAGCTGCGGCGGCGCCTCCGGAGGCGGTCGGAATATCTGCAAGGCCGAGCAGAAGCAGTCGGCCGGCACGCCGGAGCCGCTGATGCTCGCCGCCAGCTCTTGCCTCGAGAGAAAGAACGCCAGGCCGAAGAAGGCGGACGGCCTGCCTTCCAAGAACCTGATGGCGGAGAGGCGGCGGAGAAAGCGGCTCAACGATCGGCTCTCCATGCTCCGATCCGTCGTGCCCAAGATCAGCAAGGTAATGTTAATATGACTAAAAGTGCCTGTTCGGACGTGGCCTTATATATATATATATATGCTCACATACTCGTCCGGCCTATCAATTTGTAGATGGATAGAACGTCCATCCTCGGAGACACAATCGATTACATCAAAGAGCTCATGGCACGGATCAAAGCCCTGCAAGAGGAGGCCGAGGCCGAGTCCGATCCGCGGAACCGGCCGAATCAGTTAGGTGCCTTGAAGGATCAGCTGAATCCGAGTGAGACGACGCTCATGAGGAGCTCCACCAAGGTAAATAAATTAATCTGCCGGCCGTGTTGCTCTCGATCGATCGTCAGTTTAGTAAATCCTTTTGTTCCTGTAACGATCGAGTGAAGGCCGTCGCATGCAGTTCGATGTCGAGAGGAGAGAGGGGGACACCCACGTAGAGGTTTGCTGCGCGGCGAAGCCCGGGTTGTTGCTTTCGATGGTGAGCACGCTGGATGCTCTCGGTTTGGAGATCCATCAGTGTGTTGCGAGCTGCTTCAGTGATTTTGGGATGCAAGCTTCTTGCTTGGAA

>Aco025287; *Ac*bHLH57
CTCTCTCTCTCTCTCTCTCTCTCTCAGTAACAGTGTCAGTCCCAAGCTCACGCTTCTTCTTTCAGATCTACTCTCACGCTCTCTTATTCGAAGCTTCGAATCATCGCAATAGAGAGATCAAACCCTAGCTATGTGAGTGGGATCGAGCTCCGAGATGTGGCAACCGCTCCCTATGGTCGGGGTCCCGGTCGCCGGGCCCGGGGCGGAGAGGGGCGCGGGAACTCTATTCGCCGCCGCCGCCGCCGCCGCCGGGGTCGTCGGGAACCGGGACCATTCGGTGGACGAGTCGAGCGTCACGGAGAGTAGTGGCGGGAGCCGCGGGGCCGCGCGGCGGCGGCGGAGGGACTCCGCGGCGGCGGAGGGCGAGTCGCCGAAGCTCGCCTCGGCGACGAGCGGCGGCGGCAATGGCTTGGTACCCCCTTCCTCCCCTCGCTCCTGGTTTGGCTAGGGTTTGGTTTCTTGTAGTGATTTGTTTGTGTGATTGGAGAGGGTTGGGGTTAGGGTAAGAAATTAAGCCTCTTGTTGATTTGTTAAAATTGAGGTGAAAAACAGTTGTTTTGGAGGGTTTCTTGGGGTAATACTCTGGTGGAGTAGGGAAAAATGTGAACTTTACAATTGGCTAAAGGTGTTAATTTGTCCAAATGTTACTTTTGTAGTTTCGAAGTTCTTGAACTATACTCAGAAGCTGTTTGGTTGTGCTTAGTTGCAGCTGAACCGCCGTCGTCGCCGCATGCAGATCCAAATCTAGGGTTTGGTATGCTGCATTTAAATTCAACTGCTGGAGATTTGGACAGGGCAACTTTAAACAAAAGAGAAAACCTTACTTTCTAATAACTTTTTTAATATAAAATTACCTTGTTCTAATAAAATAGGGAGTAATCGAACCATCCCGTATATAATTAAAAGTTTGTAAACGTACTTTTTCAGCTGCTTACAACCGAACAGCTTATTTATAGTTGTAGTGCTTCCAAACGAGATGCATGTAGTTATAACTGTTGTATTTCCATCTGTACAGATTTCTAACTACACTATATTTACAATTATATTCAACCAACTGGCTCCTTAGTGTGTTTGATTGCTTGTGTTAGTTTTTAGGGTAAATTACAAAGAAACCCTCTCGAGCTTTAGCCTGCGCTTCAATTACCTTTGCATGAAAAATTGCATTAATTTCAACCTTGTACTTTATTAATGTCGTAAGTACGTCTAACAACTCGAATAATTGTAGAATATGGATGCAAAATTTCACAGAAATGAGTTGATGGATGATGGTGGTTATACTTAATAGAGTACAATCATGGAAATTTTATAAATCGGTTAATAAACTGCTGATTTCGGTATCTCTTGTAAAGAAAGAGATCTCTTTTCGCTAGTTTTAGATCCATTATTTATTTGCAACTTAGATAGATGTCCATATATAAATTCGAGCGATATACTGCATTCTAGCGAAGTTTTGCATCCAAATTCAATAGTCATTCCAATTTTTTGGCTGATTTGAGTTGACTACCTCAAACAAGGTTTGAAATTTTAGTTACTGTATCTGTTTTGATGATGGCTCTCAGTGCTTCAGTTTCAGTTTCAGTAGTTTTGGCTGTTTCCAGTGTCATCTTCGGATCTGGACAAAAAAGTTTCTTAAGTTTTTCAGCAAAGAGAAAATAATCTGGAAAATGGAAGAAATAAAAATAAGGCCTACTTTCTCAACATTATAATACATTATCAGCGTGCTTGATCTATCATAATTGATTCCTCAACATTTTCGAAAGCTTGCCTTGTTTTTATTGGTGTGCCGCATATTGGTTTAGTTTAATCAGTGGTTTCAATCTGCTGAAATAGTGTATCTCTAAGTTGTAACTCAATTGATATACCATTGGTTTTTTATGTTTAACAGAAGGCAATTTGGTGCTTTCTTTAGGGATATGATAGTATGCTAAACAATTTTCTATTTTTTGGAGCCCTTGCATAAAAGTAAAATCATTAAAAAAAAAGTGCAATGATTCATGTAAGAATTGCTTCTAATGTTGAAAAATGGTTGAACCGAAAGCTCGTTGAAATACTTTGCAGACTAATTCTGAAGGAAAACGGCTCAAGGGCGTAAAATCTGTTGATGATAATGTCAATGTGAAAGGTGCCGCTGAAGCAAGTTCCGGAATGGCTAATAAGCAGATGGATCAAATTGCTCCACCGCCTGAGCCACCCAAGCAAGATTACATCCACGTCAGGGCGAGAAGGGGGCAAGCAACAGATAGTCATAGTCTTGCAGAAAGAGTAAGGTTTTCTTTGAAGTTACGACTTAAGCTCCGTTATTCCCTACCTCCTTGGTATCCATGATTTGTAGCTTACGAGCTGTTAAAATAAAAATAATCATCTTTTTCCATTGAAATCCCAGAAGCAGAGGTTTCAAATAGAACCTTTTGCTTTCTGAGCTCACAAGCTCACTTCAGCTTCTAGCCACAGCAAAAGCTCTGGCTGTTGCTTCTCCATGAAGCTAGACAAAAGAGGCACGGAATTTTTTTTTTTGAAATGTGTTCCTATTAACTTGGTAGAAAGTTGGTCACAAAATTTGCAGCATTCTGAGGTTTCTTCGCTCCACAATTTTAATTATATTACTGGAATTTCTTTATTGGCAGGCAAGGAGAGAAAAAATAAGTGAACGGATGAAAACTCTCCAAGATCTAGTCCCTGGATGCAATAAGGTGCATATTCTATATTGAAATTTCCACATTATTTTTTGTAAAAAAATTTCAGATATCATGTAGTATTGCATCGGATCAACTATTTTCAACTCATGCTGAAGGTCGCAATTTAATGCCTCTTCTAGTTCTGGAGCTACTTTTCGTTGAATGTTGCGCTCTGACGTTGTGCTTTCATGCGTGTTCTCACAGGTGATTGGTAAAGCATCAGTTCTGGATGAAATAATCAATTACATTCAGGCCTTACAATGCCAGGTTGAGGTAGATACCTTTCCGGCAGTTGGCATTCTTTGGCATTGCCTTCAAAAGTAGTTCAAAAATAATTCCTTTTTGCTGCACATTTCAGTTTTTGTCGATGAAGCTAGAAGCAGTCAATTCAAGCATGGACACTGGCATTGAAGCATTCCCGCCAAAAGATGTAAGTTTTTGTGGAAAGTTTCACCATGGGTATAACATAATTATAGTTCCTACCATGATTACTTATCCTGGGTTAATCGATGATGGATTATTGTACTTTCACGGCAAAATCGTCGTGAAACATTTTGCAGTTCGGCCCTCAAACATTCAATGCTTCTTCTAGTTTGCCATTTAGCCTAGAGACACCAAGGGAGTATGGACAGGGTTCGGCAACGGAATGGCTTCATATGCAGGTTGGTGGTGCTCTTGATAGAGTGACATAACCGTAGAAGAAAATCCCCATTCATGTGAAATAAGAATTGCATACCAGGATAACTCAACTCCATGGTTGTAGGGTCTTGTATTTCGAAATCACTCAAAGTTCTTTTGTTCAGAAGGTGATTTTATTTCTATTTTGGAGCTTTTCTGTTCATTTTGCCACGCCTTTTAAGGAATTAGCTGCTTACTTTCCTCTTGCCATTTGGAGTGTCTAATATATTCGCTCAAACCGGATTATGCGACAACTTTTAGGTTAAATATCGAAAACCATCCCTGCACTTTCCTTCCTATTTCATTTACCCTCTTTCATTTTCAATCTATTTGGGAGTCAATCGTGCATTTTACTCTCACAGTTCAAG

>Aco011852; *Ac*bHLH58
ATGTGGCACGTCGGGCGATCGAACAGCTACTCATCGTCGTCGTGCGACGACGTCGGCGTCGGCGCCTCCGCCTCGACGCCTACGACGACGCTCGTCAACGCCGCGCCAAACGGTGGCCGCTCCAGCCTCAGCACCGACCCTCTGTCGGCGGCTGCAGCTGCGGTCGACGGCCTCCTTTCGGAGCCCATGGAGCACCGCTTGTGGAGCCAGCTTCTGCTGTGAGTATAAGCTGCTGCTTCTGATCATATAGAAAATTATAGCACCAAATAAATATGCATAATAGGAATAATAATAGAGGAGTGGTGCAAATGGAGCATTAATTAATTGGTCCTTAGTGCATGTTGCTCTTGTAGTACTGCATGTGGTGTTAGTGTTCACATGGAAAAGGGGGGAAAAAAGGACAAAGTTTCTTTTTTTTATTATTATTTTTATGATTTAAAGACAAATCACAAAGTTCAATTATCGAAAATATATAGATAGAAGTTAAGTTTATGTACTCGTACGGTCTAAATATTTCATGCATGGCTTTTAATTTCCATTTCTCAACACTATGTATCATCTTTTATGAACAATATATAGTACAGTAGCTTTGTTTTAATTAAATTGGGATACATATAATGATTCTAAAAATTATTGAAGAAGATCGATTATAAGAGATCATATATATATATATATATATATATATGTATATATCATATTGCACCTTCAACAAATTTTTTATCTTTCTCGAAAGTTTTACTTAAAACTTTTATTAATTATGCGCGTTCAATTATCAGGAGCTCTGGAAATGGAGGAACCATGCACAACAACCATGGAGTAGGAGGAGAAAACTTCCTTGATCTACTAAATTCCAAAAGCTTAGCACCCGAGCTTTTCGACCCGGCCTATGAAAGTTACCCGAAGCCGTCGAAGCTGGGCAACAACACTTACTATGAGTTCACCGACACTACCGCCGCGCCCGCGCTGAACCACTTAGAAGCTCTCGAGTTAAGCCATTACAATCCGAGCATGGTCGAGCCCTCGGAGATCAAAATGACACATCCATCAATGAATTTGGCCACCAACTGGTGCAGTACAGCTGCTTCTCCGAACCCGCGGTATTCGGGTTCGAATCTATACGAGACTCATAGTTCTCTATCGCAAGCTCGCGAAAGATCGGGACTAGTGCTACAACAACATCACCCTTCGTATATGGAGCCATTCAATGCTCCTATTGATCTCAACAACTCACTCATGGAGCTCAACAACAAACTCAACTACAGTGGAGTAGCAGAGCTACCGTGGACTAGTAATCGAAATTTTTCGGATTTTATAGCTTTTAATGGCTGCTTGAAGAAACAAGAAGTGGGAGTGAAAGCTTCTAAGCATTGTATGATGAACTCCTCAGAATCAAATGAGAGAAAGAAGCAAGCATATGAAGTTACATCAGTAAGATACACCTTTTTTTGGTTTTCTTTTTTTCTTTTTCTTTTTCTTTTTGGAAAAACCAATTTTAATTTTCATGTATAAAATTTGTTTGCTGCAGATAAGGGGAGATGGAAGAAACAGTGGAGCAGCAAGTGTGGGAAAGAAAAGAAAATCTGAAGAGAGTTCAGAAGCACTTCTGAAGAAGCCAAAAAATGAGAGCTCAAGTGCTGCTGCATCACTCAGGGTATGTTAAATATGGTGCAAAACATTACTACTTACTAATGCAAAATTAATTAAGAGGGTATGATTAGATACTAGTTTCATTTTTATTTTTCCCCCAAAGGTGTTAATTAACCTTTTTTGCCAAAAAAAAAAGACAAAGAAGAAGAACAAGAAAAGGAAAATAATTTATTGTTCTTATCCTGACACAAAAGTTTTTTTTTTTTTTTTTTTTGGTTTGGTGTGTGTGCATTGAATAGGTGCAAGTACCAAAAGTCAAAATGGCAGAGAAGATCACAGCACTACAACAAATTGTGTCACCTTTTGGAAAGGTAATTAAGTGCATGTGAAATTACATAATATATGTTTAATTTCTTAATTGCATTTTTTAACTTTATTTCCTAACATTTTCAAATTTTTGTAATCTGGTGCTTCATATTTATTAGTCAACACCATGAATCAAAATTTATTGCTAACATGGCAAACATGTTAATTTTGCCACTGATGTGATAATTTTCATCTGAATTAACAAAGTTTTGAACTGAAAAAATGAGATTAAAACACGTTAGGAATTTAATTGCGAAATTAAAAGATTTGAGTACCAAAGTGAATTAAAGTTTTGTAAAGATTAATTGGAGAATGATGGTGCAACTAATTCACTCACTGGATTATTAATATTGAATATTTTTCTAATTTCTCTCTTTTGTTTTAATCAGACTGATACGGCGTCCGTGCTACAGGAAACAATCGTTTATATCAAATTTTTGCACGAGCAAGTACAGGTAATTAATTAACAATTGTTTATTCTATTAATTACATCAATTTAATTATATTAAAAATTAAATTATTATAAATGCTGTGATAATGCAAATCGTATTACTTAGTCATTTATTATTACAAAGTTTATAACAATAATTTACAAACTATTCTTGTTTATTGTAAAAAATTATTACTGCAACTAGTAGTTATCCTCTTTTTTTTTTCCCCTTTAAGTATCTTTGTTGTATGTAATCTTTTATTATTAGTCATTCAAAGTTTGCAATTAAAATTTAACTTAGTTGCTAATTACCACCCGAGCTTTGAGCATTTATTCTATTTTGAAATTTTCAAAAATATTTTGTAATTACGTGACAGCCATATAATATAAAGGGGTCTGAAGCTTATTTCTCTTATTAGTAAAATATATATAAAGAAGAATAAGTTTATATATGATATAATAAGTTATAACTAAGTGCCAAGGTAATTCCACTAGAAGATTTTTTTTTCTTTTCATTTTTTTGTTCCCTCTAGAATACAAGCCTTTAACATGTCCGAAATTAACATCTACGTACCATTAATTGTTTTTTTTTGTGGTATATATTCATGCAGTTACTAAGTGATCCATACATAAAATCAAGTTCCTGCAAGGTACGTACTTCAAATTCTACAACACTTTTTTTCGATTCGTTTATAGCATATTTATTTCACCGAAAGTGAAGAGAGTGAAGTTATTTTCGATCATAAATTTTTATTTCTGTTGTTTGGTTTATCATAAGTTGAAATCATCTATAATTCAACTTTCTTAACACGCGCCTTGAGTGATGCCAAAGACAATTTGGAAGCAAAAACAAAAAAAAACCAAAAAAAAAAGAGACTATTATACCTTTTTTTTCATCTTCTTCTGTATTAGTGTGAATGTTTTCAGTTTAATGGATAAAAAAAAAAGAAGATTAATTACAGTTTTAAAGCCATTGACTTACATGTTATCAACCAAACAGCAGTGGAAAATCACTTTTGGGCAAACAAACAATTAGGGATTAACTAATGAAATTTTTTTTCCTTAATTACTTTTCATCACATATATTACACCCATCTTTTTTATTTTTCAGGATCAAAACTTATGGGGAGTGGATCAGAGAAAAGAAAAGGAAGAGGCAAAGCTTGAGTTGAGGAGTAGAGGGCTTTGCTTGGTTCCACTCTCATGCACTTCTCAAGTATACAGGGATAATAATGGGCCCGATTATTGGACGTTACCTTATCGTAGTTGTCTCTATAGATGAATTAATGAAGTAGAGAGATTCATAGAAATGTACGGTGTATCCTTTAAGGTTGTGTTGGGAGATAAGAGATGTGGAAACTAACTTATTTATGCACTAAATGCAATATTTTCAATAGAAGATGTGATCTGTACGTATCTTTACA

>Aco011855; *Ac*bHLH59

ATGGCATTGAGTTACCAGTCCAGTTGGGAAACCCTGGCTCACCTCAATCCGGAGATCGGCGGCGCCTTCCACGAGCCGCAGTCGGAGGTAGCCGACGCTCTCCTCGGCTTCTTCTGCGACCCGATCGACGCTTCGACGTTCCCCATCGATTCCCTCTTCGACTCGTCGCCGGAAAGCTACTTCTATGCCGAAACCGAAACCGAAACCGTTCCCTTACCCTCCCTCAGCCACTCGTCGCTCTCTGCGCCGTCGATACTCGCTCTGACCCCCGACCTCTACCCGCCATGCGACAAATTCGACCTCTACCGATGCCCGAAGCGACCGAGGAGCTGCGGCGATCTTTTTCGCCCGTCCAACCTCGTCTTCGAGCAGCCCGGTTCGTACATCCGCCACATCGCTGCGGGGGCAATGATGGGCGGTTGTCGCTCGTCGGACTTCCTATCGGAGTTTGCGGCCGCACCGCCGTTAGCTGTGGGGATGCAGGAGAGGAAGGCGGGCAGCGGGTGCTTGTCCGCGCAGAGCGCGGCGGCGAGGGAGAGGAGGAAGAGGATCAGTGAGAAGACCCAGGAGCTTGGGAAGCTAATACCTGGGGGTAATAAGATGAACACTGCTGAGATGTTCCAATCTGCCTACAAGTATGTCAGCTTTTTGCAGGCCCAAGTTGGTATTTTGAGCCTCATGGGCTCCATCCAGGTAATTTTAATAGATCAGACAAATCATAAACTATCTTAATTGCATCTTTGGCTTTGTATCTTCAGCAATTATCATGGTTTTTTTTTCAGTTTCCTCTACTTCCGTGTAATTTTTTGTGCTTATCCGAATAGTTATATTCAAACAGTACTTCAGCTAACAAAGTCGTGATCTTTTGATTTTATGTGCAGGAGAGAGGTAAGGTTCCGTTGCTGGTGGAGCAGCAACTTCAACTCCTGCTTGAATCCACCACAATCCAGGAGAAGTTGTATGGGGAAGGGAATTGCCTGGTTCCCAACAAGATAGTGGACACCATGGCTAAGGACAAGGAGATCAAATCCAACATGTTGGTCTCGAGAGATCTCGATCGCTTCATCGAGTCGATGAGATGATAATAGGTATGTGGTATGTTTGACTACTATTTTTCATCCTTGTTTTTTCCGATGTCTCATAAGCTGATAATCTGAAGAAACAACAGATTTTTTTCTGGTTGAAATAGAAACATAAGAAGATTAGAACACAAAACAAACACCAGTCCAATACTAGTCTTGACAATGATATACAGCATGTGCTGAACGATCCGTTCATTGATCCAAACACGGTGTTTTCATTGGTTTAACATGTTTTATGTCCTTTACAGGTTGCCAGGTAGAGCTTTAGCAAGCAATCAGCATCTATGGTTGAGCAATGCTCAATATGCAGAAAGCAAGATATTTTCCCGCTCTCTTTTGGCAAAGGTTATATTTGTTAACAATGCCTTCATCCACAGATACAACTAGTATGATAATGTAGTACTAATTATTTGCTCTCTTGTATGTGACATGCAATTTGTCATCGGGTTGACGACTTTCTACCAATCATCACCTCAGAGTGCATCAATGCAGGTATTGTCATTACCTGCACTTGAATCATGTAATATGTATGAAACTGTTGAGAAGTAAATAGTGTTATGCTATTTTGCCTTGTAACTTAAAGAAGGGAAAAGTTCTTTATCATTACTATGTATAGAGTGGTAGCTACATGAAGAGAAAGACTTCTAATCGGCTACTCTGTTTAGTCCTTTTCTGTCCCTACCTGGATGTATTTCTGCCTATAACTGTGGTACTAAACTAGATTTATGATCCTTCCACCTATAACCGTGGTACTAAAATAGAGTTATGATCCTTCCACGCAACGCCCCAATGATCCCACATTATATGGGAAAAGGATTCTGATTGAAAATATAAGAGGCCATACATCTTAGTAATAAGGCCCAATGAGTTATTATTGCTAGTGATTCGAGTCGTTACAGTTAGTATCAGAGTCGAATACTAGTGTGAAAGTTAAGTGCTACACCACTGGTTTGATGGGAGCCACCTCTAAACCCACAGGAGCCACCTCTAGAATCTCACATCACAGTGTGAGAGCTAAGTACTATGTGGGGCAAAATGCTACACTATGGGTTAGTGAGAACTACCTCTAGAATTTCAGATTGCCTGGTAATTCTGATCTATAAATTTGTGATTGGATTTGGACCCGACAAAGACGTCAAGGTCTAAAACGGAGGAGTCTATAATGCACCCTAGTAATAATAACTAGATTTAAGTACTTTGGGCCGATGGTTTGGGCCCAACAAGTTATTATTACCAGTGGGTCAAGTGTTTACATTCCACCTATAACCCTCGTACTAAAATAGATTTATGATGCTTCATGCATGTGCCTTAATTTTTATTTTTTTTTCTGCAGACAGTGGTATGCATTCCCTTCATGAATGGTGTGCTCGAGTTGGGTACAACTGAATTGGTTTGTTCTCCATATAAATTCAGATATCCTCATTATTACCTTAACAAGAATCCTCATTTATCTTTTTCTGCTTTCAATTTTATAGGTTTTGGAAGATCCCTCTCTCATACAACAAGTCACAACTTCCTTTTGGGACCTGCCATACCCTACTTGCTCTGAGCAATCTAGATCTGCTTCTCCAAAAGGTGAAAAAGAGGAAGTCAATTTATGCTCAAATCTTCATCGTAGCAATATAAATGCAATTGGTGAGGAGCCTCATTTATTAGAAGTTGGATCCCCATCTTTTCCTTTTCCTCTTCATAATTACGAAAACGAGAACGAAGAACTCCATAACAAGCTTGATGAAGTGCATGTTAATTTGTACGAAGAGCTAAATATAGACTCTACAGATGACTCTTTAAGAGTTGAGGTGCACAACAGCACATCTCAAGTTCATGGTAGGCAATTGATGGACGATGAATTCAGTAATGGTCTACATGGTTCTTTAAACTCAAGCGATTGTATATCACAGTCCTTTCTTACTCCCCAAAGAATAAAGAATCCTACTCTTTTTACCGTTCAAGAGGGAGAACTGAACAAGCTTACTGACCTTGAAGACGACGCTTCTCACTACGCAAGGACTCTTGTGGCCATTTTAAGGAACTCGAAACAATCTGCACCAATCTCTTGCCCTATGAATGGTTCTCATAACTCGAGTTTTAGTATTTGGATGAGAGGCTTCAATGCACATAAAACGTTCTCTTCCACGCCTCAGAAGTTGCTCAAGAAAGTTTTGATTGATACTGCATGGATTCCTGGTGCTCAACCAACCAAGCCCATAGAGGAAAATGGCCCACAAAACAAAGTCTGGAAATCACAAGGAGATGATGCTGGTGTTAGTCACGTGCTGTCGGAGAGAAGGAGAAGAGAAAAACTAAACGAGAAGTTTCTGATACTAAGAAATCTTGTTCCTTCTATTAGCAAGGTATATTCTTCTTATCTGCTTATGGCAATATTGCTAAATCGTTTGAAATTAAAAACTGTGATTGAATTTTCATCTTTAAAACACAAGACCTCACGGAAAAGATCTTGTGCAGATAAGTCTATCTCCATCTTATAAAAAAATTGATAGTCAGTCTTTGTAAATGTAATGGTGCTAAATATTGCAAAGCAAGAGCTAGACCATTTTTGCCTTTTTGGAGCCACTGACTTGTAGTTGAACATTCTGTCATTAATCAGTTCCTCTTTATTAGTTTGTTCCAAGCCTTCAACAAAATGTATTTCTGTCTCAGGTTGACAAAGCATCCATCCTTGGCGACACAATTGAGTATCTAAGGGAGCTAGAGCAAAGAGTAGAGGAGCTAGAATCCTGCAGAGAGCTATTGGACCACGAATCTAGAACTAGAAGGAAGTACCCTGACATAGCCGAAAGAACTTCAGACAACTACTGCAACAAAAAGATACCAAACGGCCAAGTTTCTGCAAGCAAGAGGAAGGCGTCTGATATAAATGAAACGGATGTGGAGCATCATTGGATCTTGTCGAAGGATGGTCCAATTGACGTCAATGTTACCGTGATCGAGAAAGAGGTTTTCCTTGAGATACGTTGCCCATGGAGGGAGTGCTTGCTTCTTGAGATAGTTGAGGTATTAAGCAACCTCCATTTGGATCCCCTCTCAATGCAATCATCCACAGCTGATGGTGTTTTAGCATTAACAATAAAAGCTAAGGTATTGCCATAGGAAGTGCTTGTCTTTTATTTCTCCTTTTTGGGGTCAATTGCAATTTCAGTTGCTCAAGATTGATCCCTAATTCAATTATGCCCTCAGAGTTCCAGTTGTAACAATTTAGTTCCTAGAGTTGTCCTGCTTGACAATTAAGTCCTGCTGCTGGTCTATTTACTGAAAAAAAAAAAGAAGAAAAATTCTACGTCAGCATTGTTTGCTGCCAGGGCCAACTTCAATAAATTGCCATGTCAGCAAACTCTAATGAGGATGCTGACATTGCATCTTGTTTCAGAAAGTAGAGTAACAGAAAACTTTAACTGTTATGCAGGACAAATTTGAGGACTGAATAAGTAAGATTAAAACCTGAAGGGTGAAACAATATCAGGGTCAAATTGTGAGGGCTAATTTTGTAATTAATAAGCTTTTCAAAAAGAATCAAGCTTTTTCTTTATGTTCCTGATTTTTCTGCTTTTGATCACTGATGTTGTTTTGAATTTTTTACTTGCAGTTTGTTGGTTCAGTCATGGCATCGCCAGGAATGATCAAGCAATCCCTTCAGAGAGTTGCAAGTAAATGTTGAGGTTAGTTTGTTCCAGCTGCGAATGGTAACTTTTGCAACTCCCGAACTCTTTGAATCTGTCCATTCTGGTGAGGCCTGCATCATGTATTTATTTATTCTAAGGAACTTCAATGCAATCTTGCTAAGTATTTAGTTAATGATGGATCATTCCTTTCTCCTGTTCCTTAATCATATTCTGTATGTATTGAATAATTGGTATTGCGCATCATATTTGTCGCAGAAAATGGTAATTGATAAGCTTTAATCCCAATCCTTTTGAAGCTTTTAGTAACAGTTCTTAACAAATAGTTGGGAAGATACATATACTAG

>Aco018875; *Ac*bHLH60
ATGATGGAGGCCTTCATGGCGACCAGCGACCTCCAGGGGTTCCCCTGGGGCTCCTCCGCCGCCGCCTTCTCCGCCCCGCCGATCCCCCCGGCGGCGGCCCCGCCGCCGTCGCCGCCGCAGCCGTTCTTCAACCAGGAGAAGCTGCAGCAGCGGCTCCAGGCGCTCATCGACGGCGCCCGGGAGAGCTGGACCTACGCCATCTTCTGGCAGTCCTCGGTGGACGTGACCACGGGCGCGTCGCTGCTCGGGTGGGGCGACGGCTACTACAAGGGCTGCGACGACGACAAGCGGAAGCGGCGCGGGCCGACCGCGGCCTCCGCGGCGGAGCAGGAGCACCGCAAGCGCGTGCTCCGCGAGCTCAACTCCCTCATCTCCGGCGTCGGCGGCGGGGGCGGGGGCGGGCCGGACGACGTGGTGGAGGAGGAGGTCACCGACACGGAGTGGTTCTTCCTCGTCTCCATGACCCAGTCCTTCGTCAACGGCGCCGGGCTCCCGGGGCAGGCCCTCTTCTCCGGGGCCCCGATGTGGATCGCCGGCGCCGACCGGCTCGCCGTGGCGCAGTGCGAGCGCGCGCGCCAGTCGCAGGTGTTCGGCCTCCACACCATGGCGTGCGTCCCCGTGGGGAGCGGCGTGCTCGAGCTCGGATCCACCGACCTCATCTACCACAGCTCCGAGGTCATGAACAAGATCCGCATCCTCTTCAACTTCAGCAGCTCCCTCGACGTCCCCTCCGCCGCCGCATCCTGGATCGGAGCCACCTCCGCCGCAGCGGGGGCGGCGCCGCCTCAGGTGGCTGCCGCGCCGGATCAGGGGGAGACGGATCCCTCGGTGCTCTGGCTCTCGGATCCGTCAGCATCAGCATCAGCATCAGCAGCAGCACCAGCATCCCCACCAATCCTTCTTCACGAGAGAGCTCAACTTCTCCGAATTTGCCCTCAACGGCTCCTCCTCCCAATCGTTCAAGCCCGAATCGGGCGAGGTCCTCAACTTCGGCGACAACGCCCCTCCGTCCTCGTCCACCGCCGCGGGCGCCGCTCCCGTGAAATCCGGCGACTCCGACCACTCCGATCTCGACGCCTCGGTGCGCGAGGTGGAGAGCAGCCACGTGGCCGAGCCCGAGAAGCCGCGGCCGCGGAAGCGCGGGCGCAAGCCCGCCAACGGCCGCGAGGAGCCCCTCAACCACGTCGAGGCCGAGCGGCAGCGGCGCGAGAAGCTGAACCAGCGCTTCTACGCTCTCCGCGCCGTCGTCCCCAACGTCTCCAAGATGGACAAGGCCTCCCTCCTCGGCGACGCCATCTCCTACATCAACGAGCTCCGCTCCAAGCTCCAATCCCTCGAGTCCGACAAAGACACCCTTCAGTCCCATGTCGAGTCCCTCCAGAAGGAGCGCGACGCCCGCCCTGCCCTGACCCCCGCATCGGCCCCGCCGCCACCTCCCCCGCCCTCCAATGGTGTCAGCGACGCCCACGGGCGGTGCCACGGGGCGGAGATTGACGTGAAGATCCTCGGGCACGAGGCGATGATCCGCGTGCAGTGCCACAAAAGCAACCACCCCGCTGCGAGGTTGATGATGGCGCTCAAAGATCTCGATTTGGAGGTCTATTATGCTAGCGTTTCGGTTGTTAAAGATCTAATGATCCAGCAGGCAACAGTCAAGATGTCGAGCCGCGTCTACTCGCAGGAGCAGCTCAACGCCGCGCTCTGTGCGAGGCTCACCGATCCCTCTCCTTGCAGGTAG

>Aco026878; *Ac*bHLH61
AGGGAGAGAGAGAGAGAGAGAGAGAGAGAGAGTGTGTGTGTTTGTGCGGGCTGCATACATAAAGGTAGTATGAAGAAGCTTAATTAGAGAGAGAGAGAGAGAGAGAAGGGGGGAGGGAGAGAGAGAGCGCATACATACAATGAGTAGTACACTCGGAGGACTATGTTCGAGTGAGCTCCCCAGCTTCTTCATCTACGACACCATCAACGCCACGCAGCTCCACTACACCAACCCCTCGGAAAGCGCGGCGGTGCTCGGCCCCGCGCCGCCCGAGGCAGCGGCGGCGGCGGCGGTGGAGCAGCAGCAGATGAGGGTGCAGGGAGGGAAGAGGAAGCGGCGGCGGCGGCCGAGGAGCGGCGGCAGCAAGGAGGAGGTGGAGAGCCAGCGGATGACGCACATCGCCGTCGAGCGCAACCGCCGCCGCCTCATGAACGACCACCTCGCCGTCCTCCGCTCCCTCATGCCCGACTCCTACATCCAAAGAGTACTATATACTAATTAATAATTAATGCATTCATTCGTTAATTAAGATTTAAGTTTCTTCGTCTCGAACTCGAACTCGTGACTTTTTTTCTTTTTTTTGTTGTAAAATCTCGTCACTCAAACTACCACTAGAATTTTAATAGTTTTGACATTAAAGTGTACATTGTGCAATATTATCAGTGCATTTCAAACAAGCTCGGAATTTTCCGGTTTGAGTGAGTTTTTGGAAATTCGTACAGTATGCATGTTTATATTTGTACTGTGATACTAGTCAGATATTAATTATTACCCTCATGGAGTTTCTTTACCTCATCTCATCTCATCTCTTCTCCTCAGATTTTGTCAAATTTAATGGAAAAGAAAAAAAGAAAATTAAGGAGAAATATATACATTTTGATATTTATTTATTTATTTATTTTATTTTAACGGCAACGTGCCATTGTTATAAGTACATGGTCAATTTCCGTAGTAATTAATGCGGGTACACAGTTTGATCGAAGCGTTCATACTCTAGTTTGGCAGAATATTATTCCCTTCACCGTGGAACCACATGCATTTATGCGTCCTGCACTTTCTTAATTGTACTATATTAATACATATTATTTATATTTAAATATTATAATAACATTTATAATAAATTATATTTAAATATTATAATAAATTCTTTTTATTAAATTTAAGTTTAAGTAGAATTTTAAATTTTTTTTTAAATTTATATAATAAATAGTTTTTATTAATTGGTCCCACCCCTATTCTTATTTTAAAAATTTAAAAATTAAAAATTTAATTTTTTAAAATTTGATCTTAAATTTAAAGTTTGAAATTTAAAATTTAAAATTTTGAATTTAAAATTTGAGATTTTAAATTTCAAATTTTGAAATTTCAAAGTTAAAATTTTAAATTTAAAAATATATATTTAAAATTTTAAAAATTTAAATTCAAATATAATAAGAGGATAATATTATTATTTTTTATTTATAAAACCCATTATCTTTCTTATTCCATCTTACCTAACCAAACGCTATTTTTTTTTTATTCCCAGGAATAATCTAATTTTCATCCAAACACAAAATTGATCTAATTCCCGCTTATATCTCAATTTATATTTATTTTTGAAATAATTATTTTTTTCTTATCCTCGAACCAAACGATACCTTAAAGTACAGGTTGGTTATGCGAGAGTATATGCTGGGCTTCTCTTGTATTAAATACGTCCACTGGACCATGTGCTTGTTTTCGCTCTCCTCCTCACCTGATTCTCGCTCTGGCTATTATATATATATGTAATCACCATGGTTTTGTTGTCTCTATTCCGATGATGCTAATGGGTCCCCCACCTCATTTCGTGACATGGGTTGCAGCCTCTTTGTAGAGTATTTGTATTTGGAGAGAGAGAGAGAGAGAGAGAGAGAGAGAGAGAGCTCTTTTTAAAATTTTTTGAAGCAAATAAAAAAGCTATTCTCCTTTCCTGACCCCTCACGTGTAATCTTCTTCACAACCCCCTTCCTCTCTTTTGAACTTCACCTCATATCACACACACACACACACACACACACACACACACACACTCAACACTCACGAACCTTGCGAAAGAAAAAGTGGGTTTTGGTGGTAATGGTTTGTAGTCTTATGTACACGCGACCTCATGCAGTGAGTGAGAGAGAGAGAGGGAGAGAGAGAGAGAGAGAGAGAGAGAGAGAGAGAGAGTGTGTGTTTGTGCGGGCTGCATACATAAAGGTAGTATGAAGAAGCTTAATTAGAGAGAGAGAGAGAGAGAGAAGGGGGGAGGGAGAGAGAGAGCGCATACATACAATGAGTAGTACACTCGGAGGACTATGTTCGAGTGAGCTCCCCAGCTTCTTCATCTACGACACCATCAACGCCACGCAGCTCCACTACACCAACCCCTCGGAAAGCGCGGCGGTGCTCGGCCCCGCGCCGCCCGAGGCAGCGGCGGCGGCGGCGGTGGAGCAGCAGCAGATGAGGGTGCAGGGAGGGAAGAGGAAGCGGCGGCGGCGGCCGAGGAGCGGCGGCAGCAAGGAGGAGGTGGAGAGCCAGCGGATGACGCACATCGCCGTCGAGCGCAACCGCCGCCGCCTCATGAACGACCACCTCGCCGTCCTCCGCTCCCTCATGCCCGACTCCTACATCCAAAGAGTACTATATACTAATTAATAATTAATGCATTCATTCGTTAATTAAGATTTAAGTTTCTTCGTCTCGAACTCGAACTCGTGACTTTTTTTCTTTTTTTTGTTGTAAAATCTCGTCACTCAAACTACCACTAGAATTTTAATAGTTTTGACATTAAAGTGTACATTGTGCAATATTATCAGTGCATTTCAAACAAGCTCGGAATTTTCCGGTTTGAGTGAGTTTTTGGAAATTCGTACAGTATGCATGTTTATATTTGTACTGTGATACTAGCAGAGCATATGTCCCTCATGGGAGTTTGCTTTACCCTCATCTCATCTCATCGTCATCTCATTCTCCCATCAGCTTTGAGTCAATGGTTAATGAGAAAGAAACAAAAGAAAATCAGGAAAAGGGTATAAAGCATTGATTTGATAATTTATTGCTATTATTTATTTTATTTTATACGGGCAACGGGTAGCGCATTGTTATAAGTACTTTGGTCAATTTCCGTGAGGTAATTTAATGCGTGGTACAGCGTTTGGATCGCGAAGGGGCGTTCATACTCTGTTGGGCAGCAATCTTATTGCCCCTCACCGTGGAACCGACATGCATTTATGCGGTCTGCACTTTCAACAGCATTCCCCATCATTAAATAATAATAAAGGGAATGATGACCGGAGTTTGGTCGGCGGTTTTTTCCCTATTTAAATTAGGATTGGATGACATTCGCATGTATTGACGTATAAAGTTGAGCCTTACTGCTCTTCAGTTAAACAGATGAAATCATTTCCTAATAACGTACCATGCTCGGTAGACTGCACTGTCCTTTATATTCTATATAATTATTCTAGCATCTGTTTCTATATTCTAATTTATTAGTTCAACTTAATTTTTTTTATTTTTTAATTTTTTTTTTAATTATTATTATTATTATGATTATTTTGGGGGGGTGGGGTGCATGAGTAAGGGTGACCAGCTTAATGTGGGAGGAGCAATAGATTACGTGAAGGAGCATGGAGCATACTCCTCCTCCCTCGACGCGCTCCAAAAAGAGGACCACTACTGTTGCAGCACAAGATCATCAAAGCACCCACTCACGCACAATGACGGGTCATGCGCTATCTCCCCGCCCTTCCGCCCCAGTTTCTTCGCATGTACTCCGTCAATACCGTGTTGCGCCACGGGTTTGGCGAAGGACTACCCGTTCCCGCGAGAGTCCGACCCGCCGTGGCCCGACATCGAGGTGACCTCATAG

>Aco008553; *Ac*bHLH62
ATGTCGCGGGAAGAGATGGAGCTTACAGCGAATTGGAGCTCATACAACGGGTCGTCGACCCCCGCAGAAGAGTCGGAAATTGTGGCGCAGTTCATTAGCACTTACCCATTTCAAAATGAGCAAGATCATAGAGACTTGGGCTTTGGAGCTCCACCCATGTATTGGCCTGATCATCACAATGCATCAAACTCATACTATTGTAATGGCAATGCTAACCCTAATTTGCACTACTGGTCTCAAGGGGATAGTAATAGTAGTAGTGTTAGTACAAGCACTGGCACTTGTTGTTACTTTGTGCCGCATTCTGATTACGAAAGCTATTATGTCAATACAAACTCATCCTCGCCGATGATTTTCAACTTGGTCGAAGAGCAACGAAAGAATCGGTCCCTTCAAGTAGTCCCGAACCCTAGTTTAAGAGAACAAACTGGCGTGAACGCGGAAACAAGTAGCGATGATCATGGAGACTCTAGCATGAATATTGGCCTTTCGGATCAGATAACACATCCCAAGAGAAAGTTCCTTAGTAATAGAAATGACAAGGTTGTGGATCAAAGCAAGTTTGAGAACCCAATTGAAAGCACCAAAAAGAAGTCCAAGGCATCAACTAAAGTAAGTGATTTATAATTTTTATCCCCCACTCAAATGTGCTTTTGAATCACTATCTTGCATGTAATGACCCTAACTGGTTTTTTTATTACTATCCAGGTGCAAAAGTGTGCGAAGAAGATGCAATCAAAGAGGGCGCAAAAGAATGTGAAAAGTGGTGATGAGGAAGAGTGCAATGCCGCCGTGAACGGGCAGATCAGCTCGAGCTGTTACAGCTCCGATAACGAATCGATTAATTCACAAGAAATGAATGGAGGGGGGAGTGCGAGTTCTAGTTCAAAAGGGTCTCCTGCTCTCAATCTGAATGCGAAAACTCGAGCAGGTCGCGGCTCAGCAACAGACCCCCAAAGCCTCTATGCCAGGGTACTGTACATTTATAAACCAGAATAATGTGCATATATTGTTGTTATATGCTTAGTTTACATATAGGAGCAAGATTAATCATGTTCTCATTTTCTTCTTCTTCTTTTTTTCTTTTTCTTTTTTTTTTTTTAACAGAAAAGAAGGGAAAGGATCAATGAGAGGCTGAGGATACTGCAGAATCTTGTTCCAAATGGAACAAAAGTAAGTTTTCAGACGAAGATCTCTGCGTCTAATTATCCACTACATTACATAGTTTAAAGATGAAATCTAAATAGATGGTGTTTTCTTATAGGTGGACATTAGCACTATGCTTGAAGAAGCAGTCCAATATGTAAAGTTCTTGCAGCTTCAGATTAAGGTAAAACTCCGATTCGAAATTCTTTGCATAACCAAGTAGGTGCAACATGGTTCAACGATTAAGACTATTTCTAGGTTATTTTCTATCGGATAGATTTTATATGTTACACAATATACAATCCTAAACCTCCATAATTTTACGAGACTGTGGATGCAATTTTCCATTTCTATATGGATTAAATCACCACTGTTTTCTCTTAAATTATCTCTTATGTCAGTACATATAACAGTTCATCCTCGTACATTATTTAGTGGATTGAATCACTGTTATTATTTTTTAAATTATATCTTCTATCAATATACACGTGGCAGTTCATCTGATTTGCACCATACACGTGAGGCAGAGTGTTCAAAAATATTTGCGTTATTTACTATCATATTTGTCTTTTAAGTAATATGAAGCACAACATTAGAATTGTATGATAAGTAATTAATCAATAATTGTGTCATCAAATTTTCCAGCTGCTGAGCTCGGACGAACTATGGATGTACGCCCCAATTGCCTACAATGGGATTAATATTGGCCTTGATCTAAAGATCTCTCCACCTCAGTGAAAAACACAAGTTGATATTAACCATGCATGATCAAAGAGAGAGAGCATGCATGGAGATATGCTTCTGAGATTTATACATTTCTCCTTTTTTTCTTCGTTTTCTCAGAAAGACTATACATTATTCT

>Aco008686; *Ac*bHLH63
ATGGAGTGGAACAAATGTAAGAAGAGTTGAAGCTAAGCTCTCTTGCAAAAAGGTCATCTTTTTTGTACCCATTATTATTTTTATTTATCTCCTATTCCTTTTTTTTTTCTTTCTTTTTTTTTCTTTTTTTTTGTTTATATGGATTTCACCACTTTTGGCTTCATGAAAAAGCTGCAATTTTTTGCTCATCTGAGTAGCTAATTTTTTTGTGCTTTAAACTTGTCATAATTCATCATTCATTTTAATAAATGGGTCCTTTTTTATTTTATGTTTACAGGTGTGTATTTTCACTATTTTGATTTGTTTTAAACTATACAAAAAGTTCATTTCCTTTCCCTTTACTGCATGATTTTTCCTCTTTTTGCATTAAAAAAAAACACTTCCTCTTTTGTGCTGTATCTTTTTGATCTTTACATCTATGATCCATCTCCAATATTCATTGTAGAATCCTTATTAATTTTTTTTTTTTTTTGTTTCCCCTTCAAATTCCACTTATTATTGTGCATTTTATGCCTTGGCCATGTTACATATATGATCCATGTTCTAATAATGTTCAACTATATTGTGCATGCATGTAACTACACAACTTGCTAATGTGTTATGTATTATATATATGATCTCCTAATAAGATCAATTCATTTGCTTTTCAATAGTTGCAGTGTTGTGTGAAGTAGATCTCAATAAGCAGATCAAATGAACCAATTTGTTCCGAATTGGAACATGGACGACGAATCGCGATCTCTTCGAGATCTCGTCGCCGCGCCGGATCGAAAGGAACCTATTGGGTCAGTCCCTTGTCGCGAAAATTTTCTCCGATTTTAGCTCAATTTTAAGTTTTACTAGTCTCAACATTTGTTTTCATGTTCTGATGATGATGATGCAGAGCAGACAATGAGCTCATAGAACTGCTGTGGCAGAATGGGCATGTTGTCATGCAGAGCCAAACTCATCGAAAACCCCCGGCCTTAGCCCCCGAATTCAAGCAATTGCGTAAGCACGATGAACCGATGCTGAAATCAGCGGCGACCGTTGCGGCCACGAGCAGTCTTACTCGAGACGAGACCGCGTCGTGGTTCCAATACCCGCTCGAGGATTCGATCGACAGGGATTTCTACTCTGAGTTCTTTTTCGACATGCCGAACGCCGACGAAGGGCGACCGGGCGCGGAGAATGATGCGGTTGACTTCTCGCACTTTGCGAAACAATGGAAGGGTGGTGATGCAGGGGCGTCGAATCCGTTACCGGGGGAGAAGAGGGTGGAGAGTGGGGGTTTGGGAGTGGCGGGAGCGTCGTCGTCGTCGATGATGACGGTCGCGTCAAGCATTTGCGTGAGCAACCAAATTCAAGTGGCTGCTAAGTTGAGGCAGATTGCGAGCGGCGACGCCGGAGCTTCCGCTTCGAGAAGAAGCTCGAAAGAAGAAGCTCACACTAGATTGGCTTCGCAAGAAGCCGCTGTGGCTTCGTCGTCGGGCCGTTCCGGTTGTAGTTTCGGTTTCGGACGAACGGAACAACACAACAACAAGAGAAAGGAAAGAGGAATGGAGGAATTCGAAAGCCTCAGCGAGGTAAAGTAGAAGCTCTATTTTGGAGTCTTGATTTGTAGTAATTTCTCATCTTTCAAAACGGTGCTTGAAATTATGTAGGACGCGGAGAATGAATCCGTCGAGGCGAATAACCAGGCTCAGCGGTCTACATCTACGCGCAGAAGCCGTGCTGCTGAAGTTCACAACCTGTCAGAGAGGGTGAGAATTGTCAAAGATCCTTTTTTTTTTTTCGTTCTTTTTTTTAACTTAGTTGCAGCTCATAGTCTTATTATATCTTTTATTCGATAGAGAAGAAGAGATAGAATTAATGAGAAAATGAGAGCACTGCAAGAGCTCATACCTCACTGCAACAAGGTAAGATGTCTTTGTTGCGGCAATTTCGTATCATACACAAAGTTTCAAGTTTTTAGTCAATTCGGAGAGTTTGGTGTTTAAGCAATCTTATACATGGTTACAGCAAAATAATTGTGTGCAACTGCACATAGGGAAAAAAAAAAAAAATACAAATTTCAATATGCTAGTGCTGATAATTTCAGAGTGACAAAGCATCTATATTAGATGAGGCGATCGAGTACTTGAAGTCGCTTCAACTGCAAGTTCAGGTCAGAAACAATTTTCTATTTTCTTTCGTTCTTACTTCGAATCATCGATTTGCTTAGAGAAAGAGAAAAAAAAAATTGTTCTTACATGTCTCGCAGTTTCTGTGGATGGGAACTGGTATGGGGCCGATGATGTTTCGCGGGTTCCACCAATACATGTCGCGCGTGGGTCCTGCCTCCATCCCTTCGCTGCATAGTCCGGTTCCGATGTCGAGAGCGCCATTCATGAATAATCAGTTTATGGCCTCAACTTCTACAGCAAACGCGGCAAATTTCTCCAACCAAATGCCGAATGTACCTCTCGGAGCTGGTTTACTTGGTCTTAATCATCTTCCGCTACAATCTCAGGTTCGTCGCTCTTGTACGAATTTACATTCTGTTTTTTATATGTTGTTCTTTTTTCCTATTTTTTCTAAAAGATTTTCACATGAATTTTTTTTTTGCAGGCGATGAATTGTGATGCATACGGACCTAAAACAGCGCAGCAGCATCAGAGTTCAGTGGAGCCAATTTCCGGAGCTTCTCCCTCCGAAAGTACTCCGAAGGACAAAGCTGGTAAGACATAG

>Aco009100; *Ac*bHLH64
ATGGATAGAGAGCTCCCTCAACTTCTCATCTCGCCCTCCCTCTCCGGCCCCACAGACTCCGCGGAGTGGCGGAGCATGTACCCCGTCGTCGCCGAAACCCTACCCCTCTACGCCTTGGACTGCTACGGCGGTCTCCTTCCTCCTCCTCCTCAGCTCGGAATCGCTCCGGCGCCGTATCCGCCGATGGAGTGGCTCAGCTCCGGCAACGTCGTCCACGACCTTGATGTATTTCATGGGAGCAACCCCATCGGGAGCCTCCCCTCCGCCCAGGATTCACGCCTCAAGCTCAAGTTCCCGAAGATCGAGCCCCTCGCGATCGACGACCGAAGCGCGATCGCTTTGTCCGCGGGATCCGCAGCGTTCCACCACCTCCCCGGCCTCCGATCGCTCGAACCGCCGCCGGATCTGGTGGCGGAGGCCCCGGATCCGCCGCGCTTCTCCGGGGATCTGATCTCACTGATTGGCCCCTCCAAGAAGCGCCTCCCGGAGCGTTCCTTCTCCGATCCTCCCCCTCCTCCTCCGCCACAGCCGCCGCCGCCGCCGCCTCCTCCTCTGCGATCGACCGGCTTCCCGAGGCCACCGCCGAGCGAGATCTCGCGGCGGAGGAGGAAGAGCATCAGCGACCGCACGAGGATCCTGCGGGGGCTGATGCCGTGGGAGCGGAAGATGGACACGGCGACGCTGCTGGAGGAGGCGAACAAGTACGTGCGCTTCCTCGAGGCGCAGGTCACGGCGCTCCAGACCATGCCGGAGCGCTCGGCCCGCTTCGCCCCGCCGCGCGAGCCGCCCGGCTCGCTGCCCTGTCCAGCGGCGGCGCTGGGCCGGCTGAACCGGCAACAGCTGCTCCAGGTGCTCGTCAACTCGCCCGTCGTCCAGGACAGGCTCTACGCCCGCGGCGTCTGCGTCTTCTCGGCGGAGCAGGTGGCGTCGCTGCGCTGCTCGGCGGAGCGCCACCGCCCGCCTCCGCTACTCCTCCCCCCCGCCGCCGCCGCCGACGACGACGACGACGTTCGCAACTGA

>Aco015791; *Ac*bHLH65
TGAGAAACCAAAGCAATATACTCACTCCCACTACGTACACTCATACACGTGCGACACGTACGCATGCAAATAAAACGATTCGCTACTACTACTGCAACTATTACTACATGGCTCAAGAGAACTCAGAGGCTTCTTCCTCAGTTACATTAATCTCATCTAGTAGAGGAACCAACAACAACATTAGTAGTAGTTGGTGGGATATGCATGGGAACTCCTCCTCCTCCTCCTCCTCCCTCTCCTCATGTAGCAACACCACCATGAATAACAATAGGTCGTCGTCGTCATCATCATCATCATCATGGCAACAACTACTGCACCGCCCGGATCATCGCCACGGCAACCCCTGCGGACACGTCGGCATCGACATGGCATCGCAGGGGCCTCTCAAGTTGTCCAGCGAGGCCGGAGAGTCCCACTTGTGGAACCAAGTTCTCTTGTGAGTATAATCCTCAAAAGGAAAAACAAAAAAAAGAAGAAGATCTCTTGCGAGTATATTATAATTAAGTTTACAGATCATGGTTATGAGTTATATATATATGTTTTTTTTTGAGAGAGAGATAAGCAGTACGTTGCCCGCTTCATTTATTTTATTTATAAATAAATATAGTTAGAAATGTGAAGCAATTAGAATTCGAACTTGGGATCTCGGGTACCAACCATCAAGTTCTTAGCCACTTGTGCTAGAGACGGTCGGTATGAGTTATATATATGTTAGGTTGGCTAGGGTTTTGGTGTATATCTAAGGATATTGCAGCACTAGATCATATATAGTTTTTAGCAAACTAATGGAAAGAGATTGAAAAAGAGAAAAAGTTATATATATCTAATAGCTTTAATGTTAGTGCTTCAAATTAATACTTAATTTGGAGGACTTAATTGTGCTAGAAATTTCAAAAAGGGAGATTATTAATTCGTGAAACTTTGGTTTTTAAATGCACCATAAACACTTGAGTGCATGGTCCCTATTGAAACCATAATAAGCAGTGATGCAACTAGGTTTTTATTGATATATTGAATGCTCATAAAGATATAGGTTAACTGGCTAGCCATAAATTTTTCTCCTTTGTTTGTTTATTATCATTAAAAATATTGTTAGATGATGGAACATTTCATAGATTGTATTTGGATATAAATTTGAATCACTTTTAGTTGATGATAAAGTTAAATTTATATTTTAAAAAAGTAGCTTTTAAGCATGTTTCTTTTTTTTTTTTTCTTTTTTCAGAAATGCTGGAAGTAGTATGAACATGCAGAACACCCATGGTGGAGAGAATTTCCTTGAAAACCTTAGTTCCAAAAGCTTATCATCGGAGATGTTCGACCCGGCCTACGATTACTTGAAGAAAATGGACAGCACCTGGGAGTTCAATACAAATGCACCATCTCTCAACACCTTGCAAAAAGAGCTTATGAGAAGCTACGACGGAACCATGCTTGAGCCCGAGCGGATGACCAACTTATCGAACTTGGTTATCAATTGGTCTATAGCTCCTCCCGACCCTCAAATCGATCATCGAATCGCGCCACCTTCGTGCGAAGTTTCTATGGATAATCCTTCAGTTCATTCTCAATACTTGGCACCAAGTATTTCTCACATCGAGCACGAGGTTATTCCTCATTCGCAATTGGATTCTTGCAATGGAATGATCGACGGAAGCACGAGTTACAAAGAAGATAGCCATCATCAAGAGTTTGGATACCAAATTGGTCTCAACAATTCAATTCTAGGACTCAATAACAAGCTTTGTAGTGGATTAATGACCGATATCCCGTGGTCGAACACGAGGAGTTTGTCGGATTTTATATCTTTTAGTGGGTGTTTAAGCAAACCGGAGGTCGAACTAAGAGGCCCGAGTAATCCCTATGGGAAAATATCAGATCCATTTGAGAAGAAGAAACAAGGATTAGAAACTTCATCGGTGAGCAAATATAACTCTACCAAATTAAGGAAAATTGTACCTCTTGCATATATACTATCTCATACACCGCATTCGCTATATTCTTCGTAGTAAGGATAATGTAATGGCTAAATGAATAAAGCATTGTTGTGTTCGTGTACGTAGACAAGGGTGAATAATAGGGGCAGCAGGACGACGAGTGACGGGAAGAAGAAACGGTCAGAAGATAGTTCAGAAACACTTGCCAAGAAGATTAAAAGCGAGAGCCCGACCGCAACATCTCTCAAGGTAATTGGTTTAACCTATATAGGAACATATCTATATATATATATATCTATATATAGCTTCTATTAACTTTTGTTTCTAACTTGCTTAATTCACTGGTGATTCGAAAAGTACTAGCTAGTCAGATTTTTATTTTCAAATAGGTTGGAAAATTTAGTTTAAGTGCATTTTGAATCAAAATATGTACATGCGTTCAACTTTGATGGAACTCTCTCTCTCTCTCTCTCTCTCTCTCTCTCTCTCTCTCTCTCTCTTGAGGAAACAGTTGCAGGTGCCCAAAGTCAAGCTAACAGATAAAATTACTGCATTACAGCAAATTGTGTCACCTTTTGGAAAGGTAAGCACTATACTTAGTTGTAATTAAGCAAACATTATGTTTTCATTTCGAATTAAAAAGGATATATAGCAATGTTTTCAAGTTAAACTTCTCTACTTTCTCTTATGTTTAAACAATCATATTATTACAGACTGATACAGCATCAGTACTACTTGAGGCAATAAAGTATATCAAATTCTTGCATGAGCAAGTACAGGTATGTATATATAAATATAGCACCAATATATTAAGGGCTTCCACTTCTAGCTATACATATTATTTTCCATGTAATTAATGACCATGATTATTATTCTCATATACTTTTGCCAATTATTGGTGTGATATTATCCCTTGAAATATTAGTATTATGCACTATAAGTTCTTGAAATAAAATAATCTTTCATGCATGCATGGATGGAACAAGCTTTGAGTGAGGTGCCACTAATTAGAAAAGAAAGTTGTGTTGTGGGAATATATAATAATTAAGTTTTGTCTCAATGGAGAGCCTGATTCAGCTCAGCTGATCATTATCTCTAATTTAATGATCAACTAATTGGTGCTTTTTCTTGTGAGATATATATGACTAATTTGAACTCTTGTGGTTAATTATATCCATGCAGTTATTAAGTGACCCATACATGAAGCCAAGCCCTACCAAGGTACACTACTCTTGACCCTGCAGCATCAATTATTTTGACCTCTCTCCGAAAACAGGCATTAAAAAAGTAAAATAAAACAAAATTAAGCAGCAAATTAGAGCTTATGCAGAAGTTTCAATGAAGTAAAATTACTAAAAAGGTACCAATGAAGTTGTTTTATCCAAATCCTAGTTTAATTTCTTCTAGTAATGTAAACAGAAGCTTTAAATTCAACCTCCTACTTTTACATACCTAAAGTTCGTAGCAACTTATGGAGAAGGTAAAGGGATTGTTTAGCCAATAATTTTTGAAGTAGAAAAGTTGTTTTAAAAACTAGCTAGGCCAGAAACTCTTTGGATACTTAAGTGTTGAATATAACAAAAATCTTATTTTCTAGTAACTTAAGCTTTCCGAGAATAGCAATTACTTCATATAATTTAAAATGGTACGTACAAAATGTCTTCACTCAAATGTTATCAAGTTTCTAGTATTATTTAGTTTCCTCCCCACTTAATTCAAGCCCACGTTTATTTAATTTTTTCCTATTTAATTCAAGCCCATGTTTTGGGCTTATCGAAAAATGCCGACTCCAGATGTGATGGACCGTGTTGATTATAAATATTAAAAACACGATTTTCTGTTTGTTTAAGTTTTTAAAAAACAACGATTATTTCATATAATTTAATCGGTACTTCTATCATTCCCTTCTAGGTAATTAATGGTATAAACGTACATAACATATATGAACTATGGACAATTTTGAATCGGCTATCCAACATTTCAATCTTTGATTTTACTTGCTATCTAAAATTTCAATTTGTTTGATTTTAGTCGGTCAACAGTCCTTTAACTTTAAAACTTAAGTTAATTACTTAGTTCGTTGAATTTATAGTTGCAAAAATTAATGGATTCTACAATATAAACCTATAAAAAATAAACGATACATTCAAATGTTGAAGTCAAGCTGCGGTTGACTAACTCAAATTAAACAAGTTAAAAGGTCGGATAGTAGAATCAAAATTTTGAGATATTGGATAGCAAAATCAAAATAGTCCATAGTCCAAATAAGTTCTGGTACTTAATTAGTTAATGCACTAATGTTTTTTTTTTTCCNTTTTTTTGTCCCTTTTCAGGATAATGCATGGGGAGGGTTGGATATAAGGAAGGAAATGGCAGAAATAAAGATTGACAGCCTAAGGAGTAGAGGGCTCTGCTTAGTTCCCATTTCTTGCACTCCTCAAGTCTACAAAGAGAGCAGTGGCCCAGATTACTGGACTCCACCTTACAGGAGCTGTTCATATAGATGATCTTTAAATCGCATATAGTTAATTGGCCTGGGTTGTAATAGTAAAGTATTAGAACTTGATATTGAAAAATAGATTCATTAAATCTGATTATAATTGACTGATTGAATTTGACTAGATTCGAC

>Aco017396; *Ac*bHLH66

CTCTCTCTCTCTCTCTCTCTCCCTCTCATACTCTCTCCTCTCTCTATGCCCTTTGCGAATCCCGAGGCCCATCGTCGTCTTCCTCCTCAGCTAGGGCACGCTCCGCCTCGTACATCTCCCGCCCCCTCCTCCTCCGCGGCCGCCGCCGCCGCTGCTGCTGCCGAGGGAGAGCCATGGCGAACCACCCGCCGCCGCCGAACGGGATGGGCGACGACTTCTTCGACCAGATCTTCGCCATGCCGCCCTACGGCGGCGGCGACGACGCCGCCGCCGCGCTCGGCGGCGGCGGAGGCGGCGACGGCCACCACCTCGCCGCCGGGATGGTGCTCCATCCCCGGCCGGGCTCCGCCGGCGACGGAGGGGGCGGCGCCGCGCCGCCGTTCCCGCTCGCGCTGAGCCTGGAGCAGGGGAAGGCCGGGGAGGGCTCCGGGAGCGGGAACCCCCTCCGCGAGGACGCCGATGCCAAGGTCGGTACTCTTTTTTACCCTTTTGCCCCTTCTCTCTCGCCCGAAGAGCGTTGCGTGTCGGAAAATTCCTGCAGGACATCGATGCCATTTATAGAGTGTCGAGTGGTATTTTCGTCTTTTCGCACTCACTCGTTGTTCCTCCCCCCCTTCTCTCTTTTGTAGCAGGAAAGGGATCCGGTCCACTTGGCTGGGTTGTTTCCTCCGGGATTCGGACACATGCAGACGCATCAAATCCGGCCTAATCCTCCTCCTCAGGTCCGGTTGCTTCTAATTCCCCTTTCAAATTGTCACTTTAATTGTCTTTTTTCAGGAATTTTAACTTGATTTTTGCAAAACATCAAATTTGTTTCTTGTTTTGTGAATGTGTATTTATTAACGAAATCGAACTCTGTGAATGCGAAAGAACGACTCTTACTCAACACTTGCAATCAATTACAAAAGTTGCATGTTCAATTTTCCGCATCTTGATAAATTCTTAACTATCTCCTGTAAATTTCTGATTTTGTGTGTTTTTTTGGGTATTTTGTTGTGCTAATTCCTCTGATTCTATAATCAGATGTTTCATGGGCAAGCAAAGCCTGGCGGGGTTGTGGCGGCGCCGCAAGCACCAGCGCCGCGACCGAAGGTGCGGGCAAGGCGTGGTCAAGCTACCGATCCTCATAGTATAGCCGAGCGGGTAATGTTGAACATTTTTATCAGAGCAACACACTATGTACATATACATCTATCTTGGTTTAGTTGTGAAAACTCTGTACCAATAGTTTGCAACAGAATGAGATCGTAGTTTCAAAAATTTGTTAATTTCACTGCCTTTGGAAAGTCATTACTTCCTATGAGATTGTGATGAGTTACTGATGTTTTGAATGTTTGCTAGTTGCAACACAGCATTTTTCTTATGCTTGTTAGCTAATTATAATATCGTCTGTAGAGTTTCAATGAATACACTTGTATAGTCTTTAACATAGTTTTTTGCCGCTGAAGAAGATATGGGCCTTTTTGCTCTTTCGTCTCATCTTATTTTTGGTGGTTGTTTGTTGGTGCAGCTACGAAGAGAGAGAATATCTGAAAGGATGAGAGCCCTGCAGGAACTGGTCCCAAACACCAATAAGGTCCGCATATCAAAACATTTTTTTTTTACTTTCTTCTCTGAATTTTTTTTTTTCACTCTTGAGAGATAGATAAGCATACTATATGCGGAGTGTTGTCAAGGACAAAAGAGATGGGTTGTTCTGAATTTTTTGCAGTTGTCATCATTTTGAAAACCTGGATTGTGTTGGCAATAATGAGTAGAATGTTGATTTCCACAGTTGTATGGATGACTTCCATTTATTTTTGCAGACAGATAGAGCAGCGATGCTTGATGAGATTTTAGACTATGTGAAGTTTTTGAGGCTTCAAGTAAAGGTAAATCACAACTTTCTTGTCTATAAAATAATGTCTTCTTGTTTATCATGATGAAACCTAGTAACAGCATTAATGCAGTGATATTCTCATATTCATATGTAGAAATAAGTGTTTATGGCATGACTTCTAATTTTCATCCTTATTACTTAACTACCAGGTTCTAAGTATGAGCAGGCTAGGTGGTGCTGGTGCTGTTGCACAACTGGTAGCTGATATCCCACTGTCAGTTGAGGTAAACCTAGAAATAAGATGTCACAACATACTATAATCTTTCCTAACACTGCTTATCTTTATGCGATTCTATATGCATGCATTTTACCTTTGTTTATGGGGCATGGCTTGTTTTTAATACGTTTCATGCGAATCTTCCTATTTTCTCTTATCCACTGTATAGAGCACTATAATGTAAAATATATAGATGCAAGTAGTGTTATAATGATAGTGATTCACGGAATTGGTTATGACCTTCATGCTTATGTTCACATGTTTTCCTTATGGTCGGACCTTATCCATTTATGCTTGCTAGTCCTATCAAAGTTGGCCTGGGGACACCTAGGCTACTGTGGTAACAAAATAACCAAGGGATAAAGCATTTTATTCTATGTATTATGCCAAACACAACTAAATTTGACAGGGGTAATTTATTCTAGTCAAAACAGGGTTATTCCAGGTTATTTTGGAATAACCTGGCCCGAGGTAGGGTTATTCCCCAATTTCTCCTTCACCTCGTTGTCCGTACTGCACTTTACTCCACTCCCACCTACCCTTTTGCCTCGTTCTCTGTGACTGTTTTCTACTATTCTTAAGGAAATATCCACCGGTGAATGTGGTTATCCTACTAGGGAATAAGGAAATGTCAAACCAAACACAAAATTCCTTTTATTCCTTTCCACCTTAGAATATTGAGTTATTGCAAGAATAAAAATATTTATCCCTTCAAACATTATCCCATTACTAAATGCACCCTTAAGAGTTCTTATTGCCATTATAATTCGTCGTATTAGACTATTTTTATATGCTTCTTCATACTTGTGCATGTATGCTCGGATTAATGTTACCTTATAAACTTAGGTATTGTCCTTTCACTGTCACCTGATAAGCACAGTTATTATCTAATGACCAATGTTTTGTCTTACATGTTTGTAAGCATATTATGGTGATGTAAAATGCCTATATGTTATTGCAGTTTTCTTGCACTTCTCTCTCAGAAACATCATCTGAGTTCCCATTTTTTTTTTTAACTCTGTGTGGTTTCTTACTAACCTCAGCTAGTCTCTCATTGGTAGTTCTATGCTCAGAAGTGAAGCTGAATAATCACAATTATTTTCCCTCACATGCACATCAACATTCTCTCTTTTGATGCATGTGTCGGAAATCTGCTTATATAGAATGATTCTAGTCCATACGGAGAATTGTGATCATTCTATTCTTTAAACCCAGAGTCTTGAAAGCTTGCAATGTAATCACTAATGCCAGAATATCTCAAGCATCTCTATTATTTCTCTACTATTATAGCTGATTCAACAGAGTACATTTTGGAACACATATCTTTTGGCCGTCAAAGGACTTTGTAGTTATCTATAAACGTCGCTTGCGTGTACTATTGCCTTTCTTGTTAAGCAACCCCACCATTAACTGGTATTGGCTGTCTTATTTTGTCCTCTCCTTTCAACTCTATCTTGTTGGTCTACTCCATCAATAAAGAGCTGCCATTTCAACTCTCGTTGCTACCGAATCCTTTGTTGTGGTTGCTGACCCTCTGTGCAAGTCCTTGCATTTACTATTGTCATTACTTGTAGTATTCCTCGCTGCCTAATTGGGTTTGGCTGTATTAAATATTTTTCCCTTTGCTCAACTCTATCTTGATGATGTCCTCCATCAATAAAAGGGCAGCGAGTTGATGTCCTCCGTCAATTTGGCCTTCCTATCCCCTTTGGCTCGAGTTGAAATCACTTCTCTTAGTGGTGTACTATGGTGGGCATACCTGAATTTTCGTAAATGATCATCTCACATCTTTTTCTAGCATTGCTTTCACATTGACATGATAAAAACGTTTCTTATGTTGCTATCACCACTTGTGCATCCCAACATTGTGGATACATTTTTAAAGGTGACCCAACATTGAGAACCATAATATATTGCTCATCTTGTGGGTCAAAAAGTTTCCTCTTTAAATAAATTCTATGCAGAGATTGCGTAAGATTTTAAAAGCATTATCTTCCTTTATGCTGGACAATTTATTGAAGGTGACAAAAACAATCATTATCTTCTTGCAGAAGTTCTAATCCAGCTGTCAAACTATGTAAAAGTCGCATCACTAAGCGTGCACTGGCTGTGTTTATTGTCACTATTATTTTAAACTTTATATTTATGACTGTTGAATCTAGAAACAAACTGATCTTTTGCTCATCGAAAGTATTGAAGGCTTGCCAGTTGCATTAGAAGATTTTCTGGTTATAAGTGACCTTATCTTATTTTCTGCAGCATATTTGCATGAGTGTTTCATAATTAAGGGCATTCTAAGACATTAACAGCTGCAAGCCACCTATGAATGCAATATTCCCTAGGAAGCATGCTGATATTTCGAAGCATAAGTGGGAGCTTTTATAATGAGATGGACTAGACCATTTTTATGCTAGCTTCTATTATCATTATAATAGCTTTTATTAATCTGCTGATTGTCTTATCATTTTGATTAATAAATAGCTTTGCTTTTTGACATCTGCGATCAAACTCGCTACATAGATTGAATTTTTGAACTTCTGTATGGTTTTCATTCCATCTCCCTTGCAAGCTGTATGATTGTTCTGATAAGTTATTTTGCAGTGGCCGCTATACAGCAGCAATATAGTCACTTGGCCTAATCACTCATGATCTATTGCTTGAATTTTGAATCCATTTGCTAGTTTCAAGTTACTTCCTTTGTTGAAGAAATAGTGTATCTTTTCTCTTCCTGAATTTTCTTTGGTTTTCTTCTCTCTGTTGGAGCTACTGAAGATATTCATTCTATGATATTTCCTACTTCAATCTTTCTACAATCAAATTTAAACTTGCAAAAAATAATTTCAACATTTAATAATAAACTTTGTTTAAATTCCATTTGAAGGATTAAAATTGTTTATCTGTTCTTTTGATGAAATAATTTATAAAATTTCTCTATCTTTTTCGATCTCTTGTTCATCGCTTCAAAATCATTTCTCCCTCTCCTTTCATAGACGTACCTTGGTGTCTATTTTTCCTGTTTATTGCTTTAGAATGTTTTTCTCCCCATAATCAGTACGTAGCTTGTGATAGAGTTTATCATCTGCCGCCTCCTAATGACCAACTACTTTCTTAGCTTAACCTTTTAATTACTCAATCTTAAAACTCTCCAGGTTTTTAAACTTTTTTTATAGTTTTAGATCTTCCCTGACGCTAACTGTTTTTAGGACATTACTATTCTACCATATGTTTTATATCCTTTTTAGGTCTTTTAAGGACATCCTTTGCTGGTCTTTCATATGCTTAACTAACTCGCCTCATAATTTTCTCTCTTATTTTTGTTTTTGATTCTGTTAGCTCGTTTTCTTTTGACCTGAATTCTATTAGCTTTCTTCTTTCATGTTGAATTATATGGTGTTACATTCTTTTCTGCTCAACCATTTTTTTTTTAATGCATATATCTACTACTATTTATGTTTAGCGGAGAATACTGGTCTTCCTGTCTAAATACTTAACAGATACCTTTTACTATTTCGTGCATCTATTCCTGTTAATTAGTGCCTCGATTTCTTAATGTGTTGATGCTCTCCTCTTCGCTACCTAATTATAGAACAGTTGTTTGGTATATTTCCTTTTGTGCAAACATAGTCAACACGCATTCTACAATGTTAAAATCATGCCACTTATTCGATCTCTATCCAAATGTCGCTAGGGGGAGGCAAGCGAGAGTGGGAGCAAGCAACACATCTGGGAGAAGTGGTCGACAGATGGCACGGAAAGGCAAGTGGCGAAGCTGATGGAAGAGGACATCGGGGCGGCGATGCAGTTCCTCCAGTCCAAAGCCCTCTGCATCATGCCGATCTCTCTCGCCGTGGCCATCTACGACACGCAACAACAACACCAGCCCGAGTCGGGCCACTCGGTCAAACCAGAACCGAACACCCCTTCATAGCATAACTCACCAAAAGAGGGGAAAGGAAAAAAAAAAAGAAAAAGAAGCGCCTAATGAGAAAAAATCGCCCAATGGACCAACTTGCTACGAGTTTGGCCCCCACGCAAGTGTAAGAAATCTGCACAGCTTGATCCTCCTAACATATCATGTCACCTTACTTTCCTTTTCCTCCCATTTTCTTTTTTGTCAAAGGTGGTTTGATTGCGTAGGATAATGGTAGAGAGAGAGAGAGAGAGAGAGAGAGAGAGAGAGAGAGAGAG

>Aco026239; *Ac*bHLH67
CAATCCTCTTCTTTCTCGCTCTCTCTCTACGCTCGCTCTCTCTCTCTCTCTCTTTGCGCTCTCTCTCTCTCTCTCTCTCTCGCTCACTCAGAGCTCCAACCTCGCCTTAGATCTACGCCCTCTCTCCGCCCCGCAACCTCCTCCGCGGCGAATTTAAGGCGATGTGAAGTGGCGGGATCGAGCTAGGGTTTTCGCCGGATGGATCCGCCGTCACTGGTGCCGGAGATGTGGCCGCCGCCGCAGCCGCATCTGCCCTTGGCGGGGATGCGCCTCGGCGGGATCATGATCGGGAGAGTCGGGCCCTCCTCCTCGTCCGCCGCCGCCGCCGCCGCCGAGGCGTCGGCGGCGGCGGCGGCGGGGCCCCGGGACTTGTCGGTGGACGAGTCGAGCCTCACGGAGCAGAGCGGCGCCGCGGGCCGCGGGAGGAGGAGGCGGAGGGACACCGCGGCGTCGGAGGACGAGTCGTCGAAGCCCGTCTCTACGAGCAGCGGCAACGAAATGGTAATTTTCTCTATCTTCTTCTCTTTTTTTTTTGTTTTTGTAGTTGTTTGAGTGGTTTTTAATGTTAAGATTGGTTGGTACTTCATTATGTTTCTTGATCCGTGTGTTTTTTGCCTCACTTATGGTAAAAATTGTCCTAGAATTATAGATTTCAGTGCTCTTTACAAAAAAAAAAAAGGCTCCTGTGAAATCGGGCAATGGTAGTATGTTAATTGTTATTAATATGCTTATCTGTTTCCAATTATATAATTTGGATAGCATGTTGTTTGGCCACATTTTTGGCAAGGTTGAGCCCCGATTTGAGTTAGTTTGTGGGCAATTTGGTAGAGCATGAAATAATTCTTCTTTTTTTTTTTTTTCCTTTTTTCCCCTCATTTCTATGAGGTTTAACCATGTTATAACTCTATTTAGTATCATTTCTTGCCCGTCGAACAATTCCAAGGAAACACTGCATATGCACTAAGTTAGTGCATTTCATACGTTCTGACGTTCGGTTGTTTCATTAATCCTTTATCTGGAACAATATATTCGGGAGAAACAACAACATGGATTATTAGAACTAATGAATTTGAAATAGGCAGGAGGGGTTGCAAGTGAATTAGTGGAAAATTGATGACTTCTTTTGTTATGGCAGGGAAAAGACGTGATCTTTGCTGGTGAATATTGAATTCAATGTCATTGCTAGGAAATTTCCTAGAGAAATTTGATTGACTAGACCGAGTTTATTTGTCCAATAGTTATCAGCTTTAGATGACTTTTAGTATCTCTTGGAGAAATTTCTCTACTTCTTGTCGGTAAAGCTACAGTGTATAGAATAAGGATGGTGAAATGAGTCAGCTGGCTGGAAAATCTTTGGCTTAATAATTATTAAGGACAGTGAAATGAGTCAGTTTGACCGGAAAATCTTTTGCTTAATAATATTATGATATGGAGCTTTGTTCAATGTGGCTTCTTGGGTTATTATGACATGGAACTCTAGTAAGATTCAAGTTTTATTCTCGCAATGAGAGTTATCCAAGGTGTACAAAACATGAGTTGAACTTGCATTTGATAGATGAAAATGTGCTCATATCTTGCTATTGGTTGTTAATGTCGGATCGTTGGCGCTAACCATTAATCCCAAAGGGTCGAGCTTTTAAAAGATGCAACCAATTCACTTAAAGTGTTCAGACACAGCCCCCATTGTGAGAATGGATGACCCTCGGGAAGTTGGGGTGTCACAGTTGGTATTAGGGCTAATTACCAGCAAAAAGTGTGAGATGAGTCTCGGCTGAGCCAAGTTATACAGCGGGGAGAGATAGGCTCTCAGGCTGTTGACTATTGTGGGTGGAGCGCGGATCCGCCACAAGGTCAGTTGAGATTAGCGAGATGAGTCTTACATCGCCTGATACTTGTGAGTTTAAGCCTGACGAGGACGTTAGGGCTTAAAACGGGGAGAATGTGTGACCCGGTAGTCCTATATATAAAATATAATAGGGTTAAACTCTTAATCTGGTTATAGCATTTTGGGCGGTGGCTAAATCCAAGAGGTTAAAAGGATTAAGCGTACTAGAACTGGAGTAGTTCTAGGATGGGTAACCCCAGGGAAGTTGGGGCGTCACACCCACGGGTCTTGATTGCGACATGGCCCAACCAGCCTCATGCCATTTCGAATATTACTTGGCCTAACCCGACTTTCTGCTATTTCGAACGTGGCTCAACCCAACTCGGCCTCCTGCCATAGGGAAGGATGCTGGCCCATTTAAGCCAACAATTTCCCTCCCCCCACCCCAGTACCTGCACAAATTCATAGGATGCGGGAACCGAATCCGTGACCTCTAACTCTGATACCACTTGTTAGATCGTTGTCGCTAACCATTAATCCCAAAAGCTCGAGCTTTTAGAAAGATGCAACAAATTCATTTAAAGCATACATACACAACTTCCACGGGTTTTGATTGTGACTCGGCCCAACCAGCTTCACGCCACTTCGAACATGACTCTATCCAACCCGACCTTCTGCCATTTCGAACGTGACTTGGCCCAACCCGACCTCCCGCTGCAGGGCAGGATGCTGGCCCATTTAAGCCAACAGTTAAGTCTTATGGTCTTTTTGTATTTAATAATGTTGGGTGTTACTAATTGAGGAAATCAAATTTCCTCACATTATGCTTGTCAAATTTTATTAACTAAATGAATGAGTTATGGAATTAATGAGCCAATGGTTAAGGTTGTGAGTCTAATTTTAAGATGGTCAAGCTATTTAAATGAAGTTTCATGAGTGAGGTTATAAGTAGTCATAATTGGGTGTCATGAGTCAAATTCTCTATTTTTTGGTTAATAGAGATCCTATTAGAGTTCCATGAGGCTATTTAATGAAGTTTCATGAGTGAGGTTATAAGTAGTCATAATTGGGTGTAATGAGTCAAATTCTCTATTTTTTGGTTAACAGAGATCCTTTAGAAAAGCTTGTTTTCTATGGGTTTCTTGTTTCCTTAATAAGAGTCTTAAAGATTTAGAATTAGTTTTAGTCCATATTATCAAAAGATTTTATAGTGGTAGTAGATGGGTTTGGAAAGGTCTTGAAAGATAAGGATTTGGTTTTAGGCTTGAGTAGGCCTTTTTATCTATAGGTCAAACGAAGATTATTAATAGAGTCGCTATAAGTTGTTCCCTGAGGCCCTGTGCCTTCTTTATCCTATGATTCTTCCTTTATGCTATATTTTGTCCTTGCTTCTTAATTCCTCTCTTCTTGTTCCTTTCGTTCTCTTCTATTTTTAGTGCAGAATCAATTGTTTCCAATACTAGCAAATTTATGTGTTATATTTTAACTTGTTGGATCAGCTTTAGTGCTTTTTTCACATTTATTGGTTGGTGCTACAGATCTACTTAGCTTGACACCTGTGGACCCACATTGTCTCACTTTACTTTTCTTAGTGTGTTATAACTCATAAACTTTATTATTCTAGTTACACTTTTTTCTTCACAAATAGTTTATACTAGTTCTAAGTAAGTTAGATCACTTCACTTTCTCTTTAATTGGTTTATGATGTTGAAAGACAGTGATGCATTCTATTGCAATTTCATCCTATCACTAGCGAAGATCCTGCGCTTCGCAGTGGGTCAATTATTCTTTCAACCAAAAAATAAGCAAATATAATAGAAAAATTCTTGTGACCACAAAATGAATGAATATTATATTGAAAAAAGTCCAAATACACTTTGAAGACATGGAATAAAATGAAGAGCAAACTACAAAGTTTGATGCACATGTAATGAAGAATAAGATGCACCAGGTTCAGCTGGGCAAGTAATATACAGTTAATAGGTTCATTCAAGTCCCTTTTTAAATAACATGCAATGGGCAGTATAGGTGCCTATATTCATCAAACATTTAGAAACCCACATGTCAATCTGAATGTTTCCAATAAAAAGTTGTTCTGCCCCTAGCTCCTATGCATATACCTGACATACTGCGGATGGCATTATAGATGCCTTGTGCATCTTGTGGAAGATGGATCGGAATTAGTGTACACGTAGATTTTTTTTTTTTTATGCACTTAATTACAGGGAAACCAGGCTCTTTGAAAATTTTATTACAAAGTTCTTACATTTTTATTTATAATTTAATTAATAAATGTTGTAATATATATATAAAAAAATTATATTAGTAAAAATCAAAAAATTTCCCACATATTATTTTTGCCCACATATTATTTATGCACTAAAATGATATCCTTAGTTATACCATTATTTTTCAAAACTACTTGATGCCAACGTGAAATATTTTTTAAATTTATAGTTTTTGTACTTGGATTCTAAAGATGTTATAGATAATGTCTATTGGACTAGATTTTACTTTAAATTTTCATACAAATATATTAATTATATTAAGTATGATATTAAAGATGAATAAATATGTTCAACAATAGTTTCTCCGATTCCCAATTCCATTTCATACAAATCTATATAGAATATTTCCAAAATTTAAATCAAGAGGAGGAAATTTAGGAATTAACAAGGAAATTAAAAGAATTTCTCCGAAAGCTCATCTGTCGAGGCATAATGTGGCTTCTAGGAGGAACAACTCCATATCAAGACCCAAGCCCAATTGACTTTCTTTGGAATCTCCTCCTGCGTAGGGGCACCATCAAAGGAAAGAGTGAAAAAAACAAAGAAAAAAAAAGAAGAAAAGTAGAAGAATGTTAGTAGTAGAAAAAAGGGAGAAGGAAAGAAAAAAAAAGGTAAAAGGAAAAGGTGGCAATGGAGTTACCTGTGGGTTCAATGTGGAGGAGGAAAAAAGAGGGAGGGGAGGAAAAAAGAGGAGAGGAGCAAGGTTAGGCGAAGCAGTCATTTGCCACATGCAAAGAAAAGGAAAAAGAAAAAATAGGAGAAGAGTGTTGTTGCGGACATATTATGGGTCAAGGATTTAGGTGCCCATCGGCACGGATCGTATCTATCGTCCCGTATTGTGCCAACAAGATATCGGCACGATACAGCTCCCGTGCCGATGGCACAGCTTAAAGCCCTTTTTTCTTTAAATTAGTAAGTAATTTTCTCAATAAAGTTCAAAAGATTTGATGAAGATACATAATAAGTAATTAGAAAATTTTATTGCTAAAAAAAATAAATATTTTATATCATTAGGTGTCGGCATACATGTATTTTTTTGACCGGCATGCATTGGCACGCTCCGGCACGCACCGTGCTGTATCGTGCCAGCAAGTTTTCAGTACGATCTCGTACCATGGCACTTAAATCCTTGTTGTGGATTCATTTCATGGAAAAGGCAGATGGTGATGTTTTAGAGGAGGTCCGCTGCCATTCTTCCCCTGAGGCACTATAATTCGTTCCCAGCAGCACCGTCATGCCACGCGATAGGGAGTGAGAGCTTATAACACCCAACAGAGAAGGGAAAGATGGAAAGCGGTAGGGTTGGGACTAAGCGACACATGGGTGGGGAAGAGAACCATGTCGATGTGTGTAATGGGACAAAAAAATGAGTTGTCCTTATAATAGCTAAGGTCCCGGGTGTTTAGGACCACGGTGTAGGTCCACGAAGTTTAGTGTATATAGATAGATAAACTATAGATACTATAGAATTTAGTGGTCTTGCATGCACAGTGTGCAATGAAAAAACTATTAGTGACACATGTCACTATAATAAGAAATAGGTACTTTAATGTAATATTACATAAAACTCTTTTACAAAAAGTAAAAGTCCTCGCATAAAATTTACCAACTTTTCAAACCACAAAGTAGCATAGTTTACTCAAAACTTCTCTATGTGACAATATGCATAAGTTGCAGTTTACTCAAAACTTCTTCAAAACTTTTTTTAGAAGTAAAATTCTTGCATACAATTTACCAACTTTCCAAACTATAGAGCAGCAACATGCATTATTTACAGTTTACCCAAAGCTTCTTTAAAAGGCAACATGCATTGCATGTAATTTTTACAAATAAGTTATTTAGAGGAACCTATATGCAGAAGTATGTTTCACAAATGCTATGTTGAGAAATGGAATAATCCCTACCTTTCTCCTTCATATATATCATATGATAATACATAAGGATTTAAGTGCTCATCGGCACCAACCGTATCCACCGTGCCGTATCGTGCCAGTAAGATATCGGCACGATAAAGCCCCCCGTCCCGATGACACAACTCAAAACCCTCTATACTTTAAATTAGTAAGTAATTTTCTCAATAAAGTTCCAAAGATTTGATAAAAATACATAATAAATAATCAGCATAGTTTGTTGCTAAAGAAAATAAATATTTCATGTCATTATGTGTCGGCACACATATATTTTTTGTGACCGGCACGGATCGACATGGCCTGATACGCACCGTGTCATATCGTGCCGGCAACTTTCCGGCACGACTCCGTGCCACAGTACTTAAATCCTTGATAATACATATTATCATTTTGAAAAACAAAAATTCAAAAATTTGAGAAAAATCCGTCCTTTTCTCCTTTCATATATGTTGTATGATTTTGCGAATTGGTTAGCGTCATTCTACTCTCTTAATACAGCCAGATCTCCTTTCATATATATTGTATGATTTTGCGAATTGGTTAGCGTCATTCTACTCTCTTAATACAGTCAGATCTCTGAAACTTCTTGATGTGATCATCCGGTCATCTTTTTCATCTCATTTAACTTTCAACTCTGCGGCAGCTCAGAGGAAGTTCAAGGTTGGTGTTTTTCTGAATAATAGCCTGCATCCTGGTGTCAAGTTCTGTTTGCTTTGCTGCCTGATAATTTTATGGAATTATATATATGTAATGCATAATGGAAATTTTTTGGGTCTAACCTTCTGATGCTGTGATTTTTTCATTCTAATGCATAATTTCTGAATTTTTGTTTCCTATCTGGTGCTCTAGAGTATTGATTTGATACAGTATTTACTAGAAGATAGCTAATAACATTTTTTAAGATATCTACTAGTTGATGTTCACTTTTTCTGGAGAATTAAGAAATTTGAAAGATGTAGATTGTTTGCTTTAGTTTTCATGCTTGGGATGTTTAATTCAGATAATGGATGATGTAATGTATCTTTTTTAGTTCTGAGTTTGTCTTTAAGCAATGAAACTAATTATGACAGACTGATTCTGAAGTGAAACGTCTAAAGGTCATGAAATCTGGTGATGAAAACAGTAAAGGTGAGGCTGCGCTGAAGAAGCCTACAGATCAAAATCCTCTACCACCTGAGCCTCCCAAGCAAGATTATATCCACGTGAGAGCAAGAAGGGGTCAAGCAACCGACAGCCACAGTCTTGCAGAAAGAGTAAGTTCTTTTCTCAATATTACATGCTTATCAGTCATTTTTTTTCATTTCCCTAGATTCTCGTATTTTATATCTAATCTATATTGAAAGGGGAATTCCCATTCCCTTTATTATAAGGGGATTAAGATGAATATGATAACACCTAGCATCAGGAGGGCCTTAGGTGTAGTTATGTTAAATCATTACTAGATCTCTCTGATTGATATCTCTTTACTGACAGGCAAGAAGAGATAAAATAAGCGAGAGGATGAAGATTCTCCAAGATCTGGTTCCTGGATGCAATAAGGTGCATTTATTGTAATAAAATTCTGCATTGTGTTCTTCAAAGTGCTTCTTGGTATCGTGCAGTTTTTACTATCAATGCAGTTCCAGTTTTTTAAACCATCCAACTCTCTTAGCCTATCTGGAATCTACTTTCGTCAAAGCCTGCATCTGTAAACTAGTTGTTTCTTCAAGCAGGTAATTGGCAAAGCATCGGTACTTGATGAGATTATCAACTATATTCAGGCTTTACAGCGTCAAGTTGAGGTACATCTTCTTTTTCCTGGCATATAACATTATTTAATCCTATATTGCCTAGTTTTATTTAGGATTGATTAACAATTAATGTATCTCCACAGTTCTTATCTATGAAGCTTGAAGCAGTCAATTCACGCATGAACTCTGGCATTGAAGGGTTCCCGCCTAAAGATGTAAGTTGAGTTATGCATGTAAATAACTAGTTAGTTATTAATTTGTTCATGTAAATAAATGATTCGTCAATAATATTCAGCTATTTAGATGATTTTCAAATTATATCAGAAGCATACATGCATTTCATTCCATATTGCCATTGTTTGTAAATGTCGATAGTTATACACTTTGTTTTTGAGCATGAATATCCTAGAAGAAGATATCCTAGAAGAAGACTTGGAAGTGACCTGCAATATCTAGCACGCCTACGTGGAGAAAACCAGATGTTGTAAATCTATCTCGCAGATATATAGCAATATCACTTTATGGAACAATTTGGAGAATTCACGACTTGGGGATAAGGATTAAAAAACATTGTTTTCTGTCTAGGCCTTTTATATCGTTGTAGTTCACATCCAACAGTTTGCAATTCATCCTGCAGTCTTTGCGCTTGGATTTACTACAAGTGTATAACTTGTTAATTTCGTAATAAACAGCATCTCTATACCTATATATCTAGCTGTCAACCTCTCTGTTTATCTACATATTCGGGATTTGAATACATACTGAACGTCAATATTTCAATTACAGATTTAATAAGCTGGAGATTACTCAATTTCAAATCCACTCCATTAATTTTAACTCTGCATAGCGAAAAGTGTCTAGGAGAAAAGAACCGCGTCTTTTCCCATGCATAAAGCTTTAAAATACTGGATTCCATATAAGGCGCTAGTGTTGCAAAATTTGCAAGAGATATACACATAAATAACCCTGCAGAATTATCTAATAATATATATATCCTGCAAATCTTAAATTTATGTTCTCTGAAAACTCCCCCCCCCTATATTACTTGTACTAACTCATGAAATTTCTCTTTAAAAACGATAACAATGCAAAATCAACTTCCTGTCTTGAATTGAATGACTTCCCAACCTTGGAATAAGGGTAATTTATAAGCACTTTTGAGAGTTAATATATGAAAATTGATGGTTTTGTTATGCTATAATATAAAAGTGGCATGCAATTCAAACATATACATGGATAATCATGGCTATGCTTATTCAAAATCCTTAAGGTCTTGTTTGGATATCAGAATAAGTTATCGAGTAGTCTTTGGATAATTTTTTCGGTATGGATATTTTATTGTATGATTGGAGGTAAGGAGTTTATTTTTTGCTTCTTGAAAGTTCTTGTCATCTATTATTTAGTAGGATAGCATATTTTACCTACGAGATAGTTTATCTTATTCCGAAAAAATAGCTTGAAGGCCGAATTTGCTATCTCATTCCAGACATTTGATCCTCCTTATCTACCAAATACACAAGTTTTTTGAATAAATCAAGAAGATATACCGAACAAGATATTTATGCATCCAAATAGGGTCTAAAGTCTTAAATAAAGAATTTTGTTGGACAGTGATACTTATAGAGCTAAATATAGTAGCCCTTTTTTTGGGTGGAAAGAGCACAAGCCAAAGAAACCTTGAAACCAAATGACTTCTTATTTGTCAGTAGGTTAAATTTTTTTAGATCTGCTTGTTTAGTTCTTGTTAGATGGGATTTTGTTGATGACTTAAATGGCTGTGTTCAGTTTGTTGAGTCACAGGAACTTCTGAAAAATCCATCAAAAATTTTCATGTCTCTCTCTGTTTTGCAGTATCCTTCTCAGGCATACGATGCACCTACTGCCCTGGCATTCAACCCGCAGACGCCAAGAGAGTATGGGCAAGGCTCCGCTGCCGAATGGCTTCACATGCAAGTTGGCGGTGCTTTTGAAAGGGTGACATAACATAACTGCGTGCCATTTCCTCATTCGTGGGCGATAGGATTACCAGTTTAATCCGATCCTTCTTGTTGTAGGGTTTTTCGTTTCTAAATCAGCTAGTTATTGTTGCGTTCCAAATGTGATTTTGCGTCCAGTTTGGAGCTTAATGTTGATAAGGCAATGCATTTTAGGGAAGTAGCTTAGTGGACCCTTTTTCTTTTTTTTGATGTCCTATGTTGTGTTTCCACGGCTCAGTGTTAGAAATTAGTAAAACTGTAATATTAGACTCTCTGACATTATCTAAATAGATTTATTTATGAGTAAAATGAACTTATTACTGTTGCTGTTGGCTAGAAAAAGGACCAAAATTAGTTAATTTTGAACTCTCTTATCTGCATGAGCGTTTTTCACTAACAGCACCTTCTGTATAGCTAAACAGTTTTAATTATTGATGCCTGTTTTGCTATTTTCATTATGTGTAGACGTATATAGTTTAGTTTGTGCTGTAGAGACATGTTTGTGCGGTTGAAGTGATCACGTACAACATCTTTTTCTTCTTGTATCAATCGTAGATAGTTTAAACTTGTATATACACCTCCTAAACTTTAACCCATTATAGTCGGTGTTTGCCCTGGCTTTTGAGAAGTTTTTATGCCCGAAAGTGATTTTCGGCTCAACAGTATTTGGCAATCTCGTAACCTGATTCTGATTTTTAGAGTAAGTTTTCTTATTTTAGATGCTCAAATTAGAAACGGCTAAAGCCTGTTTTTTGAAATTTGATTTTGATTTTAGTCTCAAACCTCAAATTTTTATTTTAATTTTATATATAAATTTTAAATTTTTATTCAAATTTTAATTGCAAATTTTAAATTTTATATTTGATTTTTTAAATTTCAAATGTTATAAATTTTAAATTTTAAATTAGTATTAAATTTGTGAATTTTAAATTTGAAATTTCAAATTTAAATTTGAAATTTTAATTTTGCATTTTGAATTTTAAATATTCAAATATTCGAATTATAATTTTAAATTTTAAATTCAACACTTTAATTTATATTTTTAAATTAAAATATCAAAATTTGAATTTTATGCAAATTTAAAATTAAAATGAAAATTCAAAATTTTAAATTATTTTAAAATAAAATTCAGTAAAATAATTATTATTTGCAAATATCAAAAATATTTTTTCTAAGCGGCTTTACAAAATTATTTTAGAAAAATTTCTTTCTATCTAAACTGAACTAAACGTTTTACAACTTTTAACCAAAATCAATTATTCAAACCAAAATCAAATCCGAAGATATTTGATTTTTTTTTTTTGAAAAATCAATAAAGGTATTTTGGTCAATTAGAAATATATTAAATATTTTCGAAGTTTTGAAAGATACATTAGATATTATTCGAAAATATTAAAAATAAAGAGACACAAAAAGTCATTTTAGATAGAGCATGGTAATGATACACGTAAAAGTGAGTGTAAATCTTACACTCCACTCTCCACCCTCCACCCACCTACAGTGTGGATGTCTTCACTAGTCATATATATGGGTGGAATGTAAGATGTATAACAAAAAGTGAGTGTACAAATAGTTTTTATTTTTTAGATATCTAAACAGAGCAAG

>Aco009909; *Ac*bHLH68
ATGGATAGCAACCACTACACAGAGATATTATACTACCTCTCCCCCCACCCCCACATCGCACACATGGATTCCCTCTTTCTCCTCAGCACAGAAGCTCGAAAGCGCTTCCTACAAAGCGTGTCCCGCATCCTCGGTTGCTCCTATATTTGCCTCTGGTCTCCTCGCTCCACGTACGCATCTATATATCCATATCATCATCCTGTAAAATTAAGCACTTAATTTTTGCTTATTTTCTTTCTTTCTTTCTTTCTTTTTTTTTGGTCCTTTGGGGTCTTCGATCGAAGTTACTTGATATGCGTAGATTGCTGGTTCCACGAAGATGATAGCGCGCAGCCGAGTTCCCTATCGAAGATATTGTTCGACGCGTATCGGAGTTCGCCTTGTAGCATCGTAACTGGGTACGTTGCAACAATAATGAGCTTATTACTATTATTATTATCTTTTTTTTGCTTTTATTGATGGGTCATGTTGAGCGTTTTTTTTTTTTCCCCCCTTTTTTCAAGGTGTGTTCCTGGAATGGCATACAAGGATGGGTCACCATATATCGAGCTAATCGGTTCTAACCTCGTGAACTCGGCTTCGACGCAAGTGCAACAACAGTTCTATCAGGTAAGCGCTTCATGTTTAAACTAGCTTATGTTCATTGCCATGTAAGTCATTTTTTTAAAATTTGGAGCAGGTGCTAAATTTCTATTTGTTCTTATTGTTTCAGGAAGCAGGGATTAAGGTTTGTACTTAATTATCTTATAAGTGCAAACCTTCTATTCATAATTTCTTTAATTAAATTACCTTGACGAACAAAATTTGATGCAAAATGGATGATTTCTACAAACAGACGGCTATTTTTATGGGGTGCGAGAATGGAGAGATCGAATTAGGGATGACGACGACGACCACGAGCAATGTAATCAATCATATATACTCTCCACATTTTCTGTTAGTATTTTCATAATTACGTATATATAAATATTTGTTTTTATTATGCTTGCATTAATGTAATTTTTCTTTATAAATTGATAGAGCAACATGCAAATGAACATCCAGCAAGTTTTCTCCGAGGATTTCATCCAACAATCGTTGTTAGGAGACCAACCCCCCCCGCCGCCATCGTCCTCTTCGTTGCGGTCTCTCTCGATGGGAAGCCCACCCGAATTCTCCTCTTTCAAGGGAAGCACTTCTTCTTTCATGCCGCCCGAGGCCTACGCTCGGCTCCCATTCCCGACCCCTGCCGCCGACGATGCGGCGATGGCACAAGCCATGCTCGCAGTTATCTCTTCTTCACCTCCTCCTTCGTTGCTTTACCAACCCCCACAACGAGAGCAAGCACCCCGCCAAAGGGCGTTCAAGGCCTACAATGCGGCACTCCGGCCCAAGACTGACCCCGTTAAGCCGGGCGTTCCCGGGCAGAAGATGATTAAAATGGCGGTGTCGATGTTGAGAAGAGTGCATATGATGAGGTTCGAGGCTCGGATGCCGGAGCCACGGCCGACGAGCAACCAGTTGCACCATATGATATCGGAGCGCAGGAGGCGAGAGAAGATCAACGAGAGCTTTCATGCACTGAGGATGCTACTTCCACCAGGGTCTAAGGTACATATGATATATCGATCGATCATATGCATAAGAAAATAACTGGATTATGATCCTTAACGTAACTTAATTTCTCCACTAATAGCTAGCTTGATTTCATGCTTATTGAATTACCTTGGCTTTTATACAAGATGGACCAGTTAATTAATTAGAAGCCCAACATATGATCATAACATAAATAGCTGTGATTTTTTTTTTGCGTAAATTAACCTAAACTGCCACAAAATAACACTTGGAAATTAAGGAGAATTTTTTCTTTAGGTTTTAAATGCTGCATGTTTAATCAAAACTTAATAAATGGTTTTGCTTCTGTAATGGTTAAATATTACAGAAGGATAAAGCTTCAGTACTTGCCAAGACAAAGGAGTATGTGAACACTCTAAAAGCTCAGATATCCGAACTCGAGGAGAAGAACCGAATGCTCGAATCGCAACTCCCTCCGCCAACTGAGCAAATGAAACAAGTCGACAGCGGTGATTCCAGCAACAGAGTGGAAGTTCAGATCAGCAGTGGATCTGAATCAACGTCAGAAACTCGACAGATCAATTTGAATGTGATAGTCAGGGTAGAGTGCGACACGATCGACGTCCTGCTTCGTATACTTGAATTCCTAAAAGGGAATGGGAATATTAACTTGGTATCAATTAACGCAAGGAGCACTCAGCCACAATCAAATACATATGCATCGGCCAATCTAACATTCCAAGTGAAGGTACAAAGATTATCTACTGCATATCTACTCATTTCGTACTTTCGAATAATGTTTGTGCACATACAGTAGACGATAATATAAATATATATATATATATATATATAGCAGTTCCTTCGTTCGAGTATCCGTAACTCTATAAGAACAAATTTAAGGAGTTTTTGTTTCTTTGGCTAATGTTTTGTTTTTCAATCTAGAAAAAAAGGAAGAAACTAGTAGATGTGTTATTTTTGGGGGCACCAAATAAGTAGGAAATGTTTGAGTAGGTATTAATGATAATGAGCCTTGTTGTACTAAACTGCTCCTAGCTTAATTTGCAGGACAAAGTTAATCCTCCCACCAAATACAATAGCCAATGA

>Aco010678; *Ac*bHLH69
TTTGTCATGGGCACGGTTCCTGTGGGTGTAGGGGGGGTGAGGGTTACGGAGAGCTCGTCGGCGTCGCTGGTGGTGGATGACGGGGAGAGGGTGGGGAAGAAGGGGGGAGTTGGGCGGGGAGCGCCGGAGGCGAAGACGGCGATGGCGTTGAAGAACCACAGCGAGGCGGAGAGGCGGCGGAGGGAGCGCATCAATTCGCACCTCGCCACGCTCCGGACCATGGTGCCTTGCTCCGACAAGGTTGATCCTCCTTCTTCTTCTTCTTCTTCTTCACCTTTCTATTTACTTTAACTCTTTTGAGTGAACAATCAATTTGTTTCAATCAACCATCCCGTACTAACTTCCGTGGATTGTTGATTTCGGTAACAAAAATTAGTACGTGAGTGTCACAAGACTCTTGAATATCAGATGACCTCTCTGATGATTGATGGTTCGAAGAAAGAATAGTCTAAATAACAGATAGAAAACTGTTCTGTGTAGGCTAATTCTTCCGTTTTTAAGATTCGGTTGATCATATCTTGTTGGGACGAGTTTTGAGCGCCCGGTACATTTTTCCTTGAAAAGACTAGTATCTTTGGATTCTTGTCATCGTTGGTTGCGTGTGAATATATAATTTTGAGCAATACTAATTGATTGTTCGCTTCGAACAGGCTACCCTTATTTAGCCTATCTCTTTGATTTACTCGAGAAGCTTGCGGTATTTTGATAGCTGAGCTGTGTGATCAGATGTCTAGAATGAAGTATCATATTCAGCCTTCAAATCCTTTTTTTCACCTCAAAGGTGAAATATGCTACTCTATCAAATTATAGATGTTAAGATCTTTCGAGGAGTAAAAATTAAACACCTTACTTCCAATCACACGAAAAGATCCTCATATGTTCGGACATCCAAACTGAGCCTAAATGTCTCTGTGCGTTCCCTGTAATTGCAATTCTTGTGTATTGTTCTTAAAAGTTGGCCTTAAGCCGTAACTGCAATTCCTTTCTTAGTTGTCCTTGCATCTGCGCAATAGTTAAGATCTTATTGATATCTATAAGTGAGATAGATTTTATTGAAAAACTTATGGATAATTCCGGTTCTATGGTCAGTTTGGGAGAGCTAACTTTTTTTTGGGTCAATTGATAACTCTTGCTGCTCAGTTGTTGCTACTTCTTCCTCAGTGAGACAAACAATGGTTTCCTTAGTGCTCCTAAACTTGGTTATAGTTGTACTTAAAATTGACTACCTCTCTCCTGCTTTGCTACTTATTTCATTTGTTATCTTGTTTGTATACTGTATCTTGTATTCTTATTCTCAGCTGAAAAGCAGTAAAAATTGTTGTTGTTACTTGCAGATGCAATTCACTAGATAAAAGGCTGTCAGTGGCTTTTGCTGTCATGATGTTAGTATATTCCATATGAAATAGCTAGTTGTTATTACTTTCCCTTTTAAGATACCTGACTTAGATGAGAGCAACCTTCATAAAACTTTGTCGGTTAGATTATTGCTGCTAGTTGTGAGATAGTCTTTTCTTTTGGTTTTCTTATCATATCTCCTGCCGAACAATACTTTACACGTGATTTATTTATTTATACCTCGAGCTTCCTGCCTTAGTAGGTATTTGAACTTCAAAATGCTTGCCTAGAAAGATGAACTTGGATTACCAACTGTTTTTGTGCTTCTTTTAATCCTCTCATGGCAGGATATAAATAAGTGTTTAGATTAATTCCTTCTTCTCTTTCGTGCTCTTTTATATTGTGCTTAAGTTTTCATGTATTACCCTTGCATTCTCTGCAATGCGGCATAACTTCACATCTGAATTCATGTGTGACTTGGGAAATGACTATTCTAAAACTATTGCCTGAGTACTGCACATTCTTTTCTTTTTTTTTAATCTACAGGGATTGCTTTTTAAGTTATATTAAGTGGTTTCTTTCCCTAACTTCTGTTCTCCACTTAATTTGCATAATCTGGCTTTCACATTAAGGTCAAATACCTGTTTCTTATACATACATCTGAGAGCAGTGAAACTGTGATGATTCATGTTATAAGAATTACTATCCTATATCTAATCATTTGTTTTCGATTCCACTTTTTTATTTAATTCAGCAACCAATTCTTTCTTATCATATTTCTTTTTTCCCTATATGCTAATACTAGCGTCTTAATTTTTGAATTTGCGGGAAATAAATTTGTTTTGCTTCCACTCTGTGAAACTAAATTTTTTGTTGTGTTTCTTCTAATTAAATGAAACTTCATAGCATGTAGTAAGAAGGGAAAGAAAAAAGAAAAACTGAACTGATAATGTTTTCACTTGCAGATGGACAAAGCTGCGATACTCGCTCAAGTAATCAATCACGTGAAGGAACTAAAGAGCAAAGCAGTAGAAATCAGCAAAGGCTACAATATCCCCTCCGACACAGACGAAGTGCGAGTTGAAGCTGAAGCCAATGCAGTAAACAGCGGGAGCTTCTATATCAGGGCAACACTTTGTTGCGAGGACCGCCCCGAACTCTTTGCCGAGTTAAGACAAACATTAGATACTTTACAGCTAAAGCTAATCAGGGCAGAGATTTCTACTTTGAGCGGTCGAGTCAAAAACATACTAATAATGAGGTGTGATGATAATGCCAACGACATTGACAGGCACATCTACACGGCCTCCGTTCACCAGGCCCTAAAGTCCGTTCTTGATAGGGTTAATTCAACAGTTGACTTTTATCCACGGGCAAAGAGGCGAAGGATCTCAATGTTTGAATCTTCATGTTCCTCGTCATGAGGCTCTAAAAGCTTTTTAAGTGGCACTAATTGTACATCATTCCACACTTCTATCATGGGGCTTCGGTTTGTTTGCGTTCATACAATGTTGTAGTTACAGCAGTGTTCTTTGTATACAAATGCACAATGAGGAAAGGCTCTGTGATATATCATTTTATTACTATGGTGGTCGTCGATATTCGGAGGAAAAGATGCTGCAGCGGATTAAGTGAGCCTTTAGCTTATTAACAGTGTTGCTTGTCTGTGTAGAAAGGTTGATTCCAGTTTTTAGGGATCATGTATTTGGTTTCTATTGATGTAAATAAACAAAATGGATGTAATGTGAATGGATATTTGTAAGTGTACTTGTTTCTCGTACAACGAAGGCATTTTTTGCTTCCATTTTCTCTCGCTTCTTCAGATTTGAGGTTTCATCCTTA

>Aco010845; *Ac*bHLH70
ATGAAGACGGAGATGGACGACGACGCCGGCGCCGGCGCCGGCGACTGCGGCGGAGGGTTTTGGTCGGCCGAGGACGCGGCGATGGGAGCGGCGGTGCTGGGGCCGGAGGCCTTCGATTATCTGGCGGCCGCGCACGCCGCCGCCTCCGCCGAGGGCCTCATCACCGCGGCGAGCGGCGACGCCGATCTCCAGAACAAGCTCGTCGACCTCGTCGAGGGCCCCGCCGATCGCCCCGGCCTCGGCTGGAGCTCCGCCATCTTCTGGCAGATCTCGCGGGCCAAGACCGGCGACCTCGTCCTCGTCTGGGGCGACGGCTACTGCCGCGAGCCCGAGCCCGGCGAGTCGGCCCCCCAATCCGCCAGAAACCCTAACCCCGAATCCCACCAGAAGCTGCGCAAGAGGGTGCTGCAGAAGCTCCACGAGGCCTTCGGCGGATCCGACGAGGACTATTACGCCCTCCGGCTCGACCATGTCACCGACGCCGAGATGTTCTTCCTCGCCTCCATGTACTTCTCCTTCCCCTGCGGGAAGGGCGCCCCCGGGCGGGCCTTCGCTTCGCAGAAGCACTTGTGGATCCCGGATTCGGAGCTTAAGATCTCGCCCCCCAATTACTGCTTCCGGGGATTCCTAGCATGCTCCGCGGGGTTTAAGACGATTGTCATTGTGCCGTTCGAGACCGGCGTGCTCGAATTGGGTTCGGTGAGATCGATCGCGGAGAGCTCCGATGCGTTGCAGATGATAAAAGCTGTGTTCTTGGGGACAGTGAAGGTGCCTGTTGTTGTGGAGAAGAAGAAGAATGAGGGGAATGGTTTTGTTGGCAATTATGGGCCGGGCGAGAGAGTGCAAGGGAGTGCGAAGATTTTTGGGAAGGACCTAAATCTCGGTCGGCCTGCGAGCAATGTTGGGATTTCCGTGTCGAATCAGCCGAATAGTAAGAACAGCAGTGAGCAGCATATGCTGTTTCCGAACGTAAGGAAGGGACTTCAGGGCTTCAATTGGAATCATGCTCGCAATTTGAACCCTCCTCAGCAGTTCGGTAACGGGATAGTGGTGGCGAGCAATGAAGTGAACCATCACGCCAATGGTATTGGGGATAGCCCTGTTTTGAACCAGTTTCAGCTACAAAAGCAGTCGAGGCAGATCGATTTCAGCACGGGAGCCACTTCGGCGGCCGGTGGTAATTTGGTCGCCCGTGTTCAGGGTCCCCTAGAAGGGGAAAACGCAGATATCGATGCTTTGTGCAAGGAAGAGAGGGGTAGCGGTGCAATAGAGGAGCGGCGGCCAAGGAAAAGGGGGAGGAAACCCGCGAATGGGAGAGAAGAGCCCCTTAATCACGTGGAAGCCGAACGCCAGAGAAGAGAAAAGCTCAACCAGCGGTTTTATGCCCTAAGGGCAGTCGTACCCAACATATCAAAAATGGACAAGGCGTCCCTCCTAGGGGACGCCATCGCGTACATTACCGAGCTGCAGAAGAAGCTCAAAGAGATGGAGTCGGAGAAAGAGCAGATGTTGGATTCTAATATGACGGACCCAAGGGAGGGAGTGAATCATCATCAGCCGCAGGTGGATGTACAGGAAATTCAAGACGAGTTGATTGTTCGAGTGAGTTCTCCTATTGAGACTCATCCTGTGGGCAAAGTGTTTCGAGCCTTCGAGGAGGCGCAAGTGAATGTGGCCGATTCAAAGGTTGCGGCTGCTAACGGAAAGCTGGTGCATACGATCGTAATCAAGTCTGCGGGCTTCGAGCAGCAGATGAAGGAGAAGCTGATCTCCGCCTTATCCCGTGTGATGAGCTCGACGTAA

>Aco016398; *Ac*bHLH71
CTAAAAACTAGTAGAGAGAGAGAGAGAGAGAGAGATAAATTGAGAAGAGGCAAAAGATACAACAAGGAGCTCAATTTCTCTCCCTAGAGCTTAGAAATGGCACAATGCATCCCTACTCGAGCGTCGACGAATCCCTCCGTGTTAGAGAGACAAAGAGTGCGCCTTAATTGGCAGCAGCAGCAATCGACTGACAACACTGACTGCATTGGTTACAACAATATTGAGAGTTTCGCTCCCCTCTCGTCTTTTGATCAATTGCCCAAGAATTTTGTCGATGGCGAGAGATCTAGAAATGAAAGTTTTGGTCATGTTCATGACCCGATCGAAAGCCTCGGCGACGGGTGGCCGGATTTTTCTATGGTGAATTATCCAAACTTTTGCGCGATCAATGTCGGAGAAAAGGCAAATGATAAAGAGAATAGCGATAGCTCAAAGAAGAGAAAGTTCGGGAATTTTTCGAATTCACAGGTAATTAGCATTTTCTCCAAAACTACTTGATCACAATTTACATGCTATTTGTGTTTTCGCTTTGATATTCAACTTATATAAGGCTAAAAAGCTCATCACATGTAAACCTAGCTAGAGAAGCTTGGAAAAATATTTAAAGGCAATAATTTGTTAAAATTTGTTGATAGCAAAGGCACCTTCTTTAATTTATAAGCGTAAAATCATACAAAATGTCGATAATTGAAACTAGCGTGTTAAATTTAGAAATTTTTCGAAATTTTCTCATGAACATTGAAGAATTTGAAGTTGCTACACTTTGTTTACATTTGAATCAAGAGATGAAAATTTTCTTTCTTTTACTAGCAGGTGGATGCTAACAATGCAACAGAGAACAGTGGCAGCTCAAAGGGAATGAAAGAAGATTATGTATTAGGAGAGAGAAAGGGCGCAACCGGAAACCAAAAGCCTCAAAACAAGAAGGAAACATCAACAGAAACCTTGAAGGAGAATGGGAAGGCATCCAGATCGTCGACTACGACTACGCCGCTGAAGACCGACTACATTCATGTTAGGGCTCGCCGTGGCCAAGCCACTGATAGCCATAGCCTTGCTGAAAGAGTATGCGATCTCTCTTCCTCTCTCTCGCGTTTCCTCTTGAAATTTAAGCTGCTCCACAAGTGTATGTTGTCTTAAATGGGCCAGCATCCTACTCTATGACGGAAGATCATGTTGGGCCGAGTCATGTTTGAAATTGCGTGAGACTGAGGTGGGCCAAGCCATGTTCGAAGTGGCGCGAGACTGGTTGGGCCGAGTCACAATCGAGACCCGTAGGCGCTGTATCTATACGCTTTAAGTGAATTAGTTGCATATTTCTAACAGCTCGAGCTTTTGGGATTAATGGTTAGCGCCAACGATCCGACAGTGTAACACGCCGGTTTATATAAATAGAAGAAAATTAAATGACAGCAAAAGCTCGCATAAACTTAACTGAAATCAAGATCTCGTTTTGATACCATACGATCAGCCAATTTTTTATACAATTTTAAATAACTAGAAAATGGTGTTTTAGATTTCACTCTCTCTTATATACACACACACACACTCTCAAAACAAACGTTTTGCTCTAGCTAGTAGCTTAAGCTTCTCCACAGGAGTGGTACATGATTTGAATAAATGGGGCCAGGGAAAATAAATTGAGAGTGAAAGCTTAAAAAAACTTCAATTGATGATCTCATGCTCTGACACCATATGAAACAATCAATTTTCTCTAAAAGCTTAGATGTTAAAGAATGTGTTTTAATGTTCCATGCTCAAAACATCTCTCTCTCTCTCTCTCTCTCTCTCTCTCTCTCTCTCTAGCTCTAAAAGTTTTAATACTTGATGTAGGTGAGGAGAGAAAGGATAAGCGAGAGGATGAGGTACCTGCAAGATTTGGTTCCAGGGTGCAACAAGATCACTGGAAAAGCAGGCATGCTTGATGAGATCATCAACTATGTTCAGTCTCTTCAAAGACAAGTTGAGGTAGTTTATCATATATATATATAACCGATCGTCGAATCATATGTCCTATCATCGAGAGCAACTTACAAATACGGAGACCTCCGTACTTTCGAAAGCATAGGGGCCTAGCTCTATATATATACATGTATATGTTATTTTCTAGGTTTAATGTGTTGATTGATCTTTTCTTTTTTTCTTCTTTTCTTTCTTTGTTTTGTTTTCAGTTCTTGTCAATGAAGTTAGCTGCTGTTAATCCAAGGCTGGACTTCACTGTTGATAGCTTCTTCAATGAAGAGGTTAATTTCCTCTCTCTCTCTCTCTCTCTCTCTCTCTCTCAAAACAAATAATTATGTGAAATTATGTTCCTATTAAATGTGGTAGGGTCCATACTGTGGTTCTTGTTACTTGTTAGGCTAAACACAAGCTGGTAGTTTTCATGTTTAACAACAGTAATAGGCCCACTACTATAACAACACACCAAACCAATGCAACCATGCATATGGAGACCACTAGTCAATGATTAATGGTGATCAGGTTGGGTTTCACAATCACGAAAAAGCAGAAACCAAAATGAGCATTCAAGTCGCAAGCATAAAGAAACTCTAATTTAAAGCTTCTGCTAGGGCTTTAATGTATGAATAAGTGAATAGTATCACTTCAAGTAGCTCTACTCAAAAAAAATTTCGACTGATCCACACATACGTACCTTAATTATATACTTTTGATACCTAACCATTACAAAACTCTACAAGTAGAAATGTTAAGAAATGAATTATAGTACGGCTTTTTGAGTAGTTGAACTCCAACAACACTTCTGCGTGCACGCATGCATGCGAGCTTCATTGGTCTATTTATGATTTTGAAAAACTAAATATCTAGTCATGATTTTGAATGCAGATGAATATTGCTTGCAACACTGGCCTCATTCAAGGGATGAACATATTATTACCTCATGACCAATTGGATCCATCCTACATACAATCCAACGCTCTACTACATCAAGCACAAGTTGGTCCCTCATCAAGTGGACTAGACATGGCCATGAGCACTACCCAACAAATGACCCTTCAATGTCCCATGAGTGCACCCATGCAACTGGTTAACACATTCATGGATTCATGCTACAACAATGTAAGAGACATTAATTTAATTTCTATACTATGCTTTAATGTTTCCTATAAGAAGATATTTTATGTCATTTTCAAGATATAGAAGCAACTTGGTATTAATTTTATATAATATATATTTTTAATGAAATTTTCAGGTACATGGGTCTTCTTCTCTTTGGGACCAAGTTAGGTTGAACAACACCTTTGGAGCTCATTTTTCTTTTCAATCACTACAAGGTATCAAAAAAAAAGAAAGGAATTAAAGGAAAAAAAAAACCTTGGTTTAAAACTTAGGTTTAAAACAAGAGATTTTCAAAACTTAGGTTCGAATCCAATCGAAGGCCTTAGGGTAGTCGGTTTGATCGATTCTCGTACAACACATTTCTCATTAGAATTGTTGTTGTTTTTTTTTTTTTTTTTTTTTTTTTTTTTTTTTTTTTTTTGATAAGTTGATTGAATGATTTTGAAAGCTTCTAGTAGCAATTTTACTTAATTGTGTTGCTATTTGTCTAAAATAAGCTAATAAATTTCATCTTTATATTTTTGCAGGGAACTGTTTACCAAACAATCTTAAGACGGAAACGCGAGAGATTTAAGATTCTCCTATTCGAGATTTCTAAATCATTAATCGCCGAGTCCGAAGATTCTTTTAGAGTACATAAATTAGTGCTTCAATTCATTTTTCGATATCATGTTGTACCATAATTGACCATTTTTTCCATTATTCTAAGTACATTTTGTAGAGAAGAGAGTGTAATTTGACTTCAATTCTTTTCTAAAATTAAGCATGTTTACTACTTTTTGAGCCAAACCATTTTGTGGATTTGTATTAATGTTTATTTGGTTCCAAAAGTTTAAAAAAATAAGAAAAAAAATGACAAAAATTTTTTGCAATGAAACCATATGTTGATTGCAACTTAAATGCAGGAATTTATTTGTTCACTTTTTTTTTTTTCTATTTTATCTCAAGTGAGTAATTCAATGGAATATAAAGCACATTAACAACTGAAGTGATTATTGAAAAAACAACTTTTCACTCAAAAAATGTACTTTTCTTTTTTACAACAAATGCATAAAATCCAACTATTTCTTTAATTTTGGTCCAAAAGCACTTTTTTACAACTTTTTCACGCAGTGATTCTCATGACCATCCTTTTTGAGTTTGTGTTTTGGACCTTATCTCTCCTATCCTTTTTCTCAAGGACAAGGATGGACAAAATTAAATGTGGGATAATTGTGGCCATGAACCATATTTATTTAGAAAGAGAAAGAACATAACATGTTTTTTGCCCCCAATTTTGGACCTATATATTTATTTAGATCCTCTACAATCTTGGCTTGTTTCATTTCACCGAAAATGGAAGGGAATGGAGTTATTTTCGACCGTAA

>Aco016415; *Ac*bHLH72
AGAGATCTAGAGAGAGAGAGAGATGAAGAGGAGTTGTGGGAGTAGGGGATCTAACACTACTACTACCAATAATAGTAGTATTAATAATTGTGATAAGGTGGAGAGGAAAACTATAGAGAAGAATAGGAGAATGCATATGAAGAACCTAAGCATGAACCTCCTCTCTCTCATCCCAAAAGATCACTTCACCACATCAAAGGTACCCTTTTTTTTCATTTATTTAATTTATTCCTTCTCTTTTTTTTTAAAAAAAATTATTTATTACCCTTTTTTTGGATTCCTCCTATTTTTTTCCTCTTTCCCCTTTTTTTTTTCCTATTTTTGGGGATTACTATAAAAAACTTTTGTTCTTGTTTGTCATTTTGCTAAAAAAAAATATATTAGAAAATAGTTTTCTGGCTGAAAAAATATATGAAAAAATAGTGTTAATTTTTGTTTTTTCGTATTTTTTTAAATTGAAAAACGAAAACTATGGAAAAGATGTTTACATTAAAAAAAATTAGAAAAAAAACTTTTTTATGGCTTATGAGAATGTGTTGGAATTGATCACTATAATGCCTCAAATGTACAATTTTTTTTAATCTTTTCTTTTTTGTTTTTTAGAGTTGGATTACAAAAGGTGCATCTATTTCCNAAAAAAATGATTAATTTTGATATTATTACTCATTTTGTTTCCTTAGTTTAGGGTAATTAAAGCACTATATTCAAGGTTTTTTCCCCCATGTGTTATCCTCATTTCATGAATTATTTTAAGGCCAAATGAGTTGTTTTTTTTTTGTTTTTTTGTTTTTTTGTGTGAATCCAATAGCCCTAATTGAAAGATTTTCTGAGTAGTATTAAAAAAAAAAAGAAAAAGGACTAGTATTTATATTTTAGTCCTTTTTATCTAATTAATTATTTTGCTCTCAATTCCCACCTAATTAAATTATTTTAAAAAAACCCAATAGTAATATTCTTGGGCATATCCCAGGTAGGTTGTGTTAACTTTGTAACTTGTAATATATTATTCAATTAGGCCCCTTGCTTCTCTTTTAATTATTACCAGGTCCTTAGAATCATACCAACTTGTGTAAATATAAGAAAATTAAAAGGCCCATGTGCGTGTTCTTCGGTGTTGATAGAGACTTTTATTCATGAAACTTTTTTAATACGAGGGAAAAGCATGAAATATTATTTTTTCGAAAAAAAAAAGAAGAATTAAGAACACCATTTTTAATTATTTTTATTTCCTAGATAAAAAAAAAAATGGAAAGTGAAATGAGATTTATTTTCCCTCCCATGAAATCTATAAAAAAAAAAAGAAAAAATTTCAAAAACCCCCCTGTGGTTTCGCACGTTCTCACTTTAATACCTTATGGTTTAAAGTGTATCAATTTGCCCCTTGTTGTTTCGTTTTTCTCTTTTTATTATCATTTTTTTTTTCTTAAATCAGTGACAAAGTTAAAATTAAAGGATACTAAAGTAAATATTTAATAAATCTAGGTGGGTATCTGAAATTTTTTGTATATAATTTAACGGAATATTAAGGAAAAAGGTGGCGAAAAGATAAAAATAAAACCACATGGGGGCAAATTGATACACTTTAAACTATAGAGTACTAAAGTAAAGAGAGAAAGTACGAAATCACGGATTTTTTTTTTTAAGTTTTTCCAAAAAAAAAACAAGTTTTTCAGAAAAACTACTTTTTGTGGGAAACAAGGGACGAAAACTGGGCAAACTGTTTTCTAAGATATGTTTTTATAAGATAATAAATTTATTTATGCATTATACTTGAAGAGATCATCAAATTATAAGACATTCCTTTTATTAATTTTATGCATTTATTATCTTGTGATATGATTCGCGAAAACGGCTTTTTTCGAATCGGTTTAGTGTCATAATCACATTTAATGTCTTTTTTCGACTCAGTTTAGTGTCATAATCACATTTAAATAGTATTTGTGTATTGTATAAACTATTTTAGGGCATAATGGTTATGTAACCAGAATAGGTGTTTATCATATTTATTTCCAATTTTCATACAATTTAAGGTACTCAAAATCGAAAATTTTCATATAATCTTTCACATTAACCTGTCCATGTCGATTAATATCGCAAATTGAGCCTTCCCAATATTCGTACGAAGAACTAAGTAAACAATTTTGTGGGACATATATGTATATAGAGTTGAGCTATCGTGCTTTTAAGCATTTGTGCTTATGATTTTTTAAGCTGTCGGATCGCTTCAAAATCCGTACAACATTATTTGTATGTCCCAGTAAGTTTTTTTTACTAGCGCGGCGCAGATTTTGAGACAATTAGACAGCTAAAAAGTCATAATCACGCTCTCTATATATATTTCCTAGAAGTTTCAAATATTCTACATCATATTTAACGGAGTGGATCGTCTATTCGAAAGTTTCATCATTGAAAACAATTTATGAGTATGTAGGGCTTTATATTCATAAGAGTATAGTAGCTCTTCTCTCTCTCTGTGTGTGTGTATATATATATATATATATATATAGTTTTCGACGTCTGATGTTATTTGCGGTGTTGTGTTTAGGATGCATTGACGCAGCTCGATCACTTGGACGTAGCCGCGTCGTACATAAAGAAGCTAAGAGGAAGAATCGAGAAGCTGAAGCAGCGGAGAGAGTCGGGACCGAGCATTGAAGGTGTTAGGGAAGATGCGAGTGAGGGGACGATGACGACGGCGACGACGGCAGCGGCGACGATCGGTGTTAGGTTACCGGTGGTCGAAGTGCGGTACCAAGACAGGCATTTGGAGGTGGTTTTGATCATCAGCAGCAACAGCAGCAGCAGCAGCAGCAGCAGCAGCAGCAACAGCCAGAAAAGGCGGTTTCGGTTCCACGAAGTGATTAGCCTTCTCGAAGAAGAAGGCGCGGAGGTCGTCAACGCGAATTTCTCCGTCGTCGGCGACAAAGTGTTTCACACAATCCACTCCCAGGTATATATATATATACATATATTATTACTGTATTTTCATCTTAAGTTCGTAATTTACGCACTTCGTTTTTCACTCTTTTGTGGCTCTGCTCATCGTAAATTTAAGTTAGCTGAAAATGAGAAAACATAACTAGGGATGATTCTACATGATTCTACATGTATGATGATTTTGCATACGCTCTCGAAAAGTCAAAAACATTGTCGATATAACTATATTTTAAAGGGGATTTTGCTTATTTATCTTACTATTTAAAATTTTGAAATTTATTTCAAAATTACTAAAATACCCTTCTAGAATCCAATCTTATTAACAAACCTTAGGAGAAATGAGAATATTTGAAGGAATTTTTTAAACTCAATAAGGGTATTTTGGTTGTTCGGGCGCAAATAAAATATTTTTGAAGTTTTGAAACGCTTGTTATATATCATCCCTTTTTTTTAACACGTAAAATATATTATCCGATAGAAATGGTTGTCAATGTAAAGTCATTTCATGATCGAGTGCGAATCTTAAAGTGGCGTGAATGACAAAGCGAGCAGTAATAGTCTCGTATTGAGAACCATCCCCGCCAAATCTCTTTTTCTTTTTTTTTCTTTTTTTTTTCCCTAGAAGAGCTTAATTTTACCAATATTTTCATGTGTATATACCATATTATCTCTAAAAAGTATTTGTTTTACGAATCTCCAATATCCTTTTGCCTCAAATTTTGCGGTACGAAACCGATAAACTAGAAAAAGATGAATTGTTTTGCGAAAGAAACTCTAATAAATGCACGAATATGCGTAGATCGATCGATCGATCGATCAATCGATATTGATTCGAAAAATTCGTATGTATGTTAACTATTCCTTTCGGTGCATGTATTTTCAGGCCGTTTGTTCGCGAATCGGATTGGAGGCTTCGAGGGTTTCAGAGAGACTGAAGAAGTTGGTGTTTTGAATGTTCGTCGACTGTAGTGCAGTTGTAATCTATTGAGGAGAAATAGAAGGAGACTTAAAAGTTTTTTTTTTTTCAGTTTACTTTTTTTTTCTCTTTCTCTCTCTCTTTAATAAGA

>Aco016434; *Ac*bHLH73
ATGGCTCTCACAAGGGAGAGGACGATAAAAGAATTGCAATACGATTCGATATACGCTCCTTCTCTCGATCTTTTCGGATTTAAAAGTCACTACTCTTCGTTTCTTGAGGAGGGAGAAGTCTCGGAGTCTCTCTCGAGCTACTCCGCGGGCCTTCCCCCTATCCTTTCTGACCCGAGCAACTCTCCTGCCCCTAGTTTCGTGTTCGGGATGGGGAATTCTCCACTTCAAGAAGCTCACCTGAGTACAAGCTTCAAGGTCGGTTCGGCTTCAGATCACTGGGGTTACTGCTCGAGCTCTTCGGTTCTGAGCTTCGAGCAAGGCGGTCGGTTGCCTCATGATAGCTATTTGAACCTCGATCAAGAAGAGGAGTGCGCGTTATGGATCGATGCGGCGGATCAGAATTATAGTATAAATCAATCTCATGATCAAAGTTTCGACATTAGTGGCGTGAATGATAGCCAACAACAAGGAGAGGAGAGGTTTGGATTGCTCTACTCGAGTTCGAGCTCCGCGGATGGTGTTCAAGAGAGTGCTAATGTACACAATAAAGTCCCCCAAAAGCGGTCGTATGTGGTAATTTTATGTTTCAATATTGAATTATGATGATAACATACATAGCAATTCTTGTTCCTTAACAAATAGAGTTCATCACTTCATATTTGTTTTTGTTGTAGGGTGGTGATGTGCAAGTAAGCACTTCTAAGAAGCAGTGTGGAGCAAATAAGAAGAGCAAGGCCAAACCTAGTCCATCCAAGGATCCTCAAAGTATTGCAGCAAAGGTGTGCTAATTTAACATGCCTAGTATAGTTAGGGCAGGCCATTTTTGTGATCACTATGATAAATAGAAGTACTAAAAGGTTCTTTCAAACTGCAGAATCGGAGGGAACGGATCAGCGAGAGGTTAAAGATTCTGCAAGATCTCGTGCCGAATGGCACCAAGGTGCACTACATTGCTCATCTTTGGTGCATGTTGATTTCTTATTCAACTTGGCATTAATTCTTCTTAATTACTCAATTTATATTTGTAGGTTGATTTGGTTACCATGCTAGAGAAAGCTATTAGCTATGTGAAGTTTCTCCAATTGCAAGTAAAGGTAATATATATATGATCATTGAGTTATGAATCTTATATGAGACTACTTAATTTAACATATAAGCTAAGACTAATCCTTTTAGGCTTGAATAAATATGTAGGTGTTAGCAACTGATGAGTTTTGGCCTACACAAGGAGGGAAAGCACCTGATCTCTCTCAAGTTAAGGACGCAATCGACGCGATCTTGTCATCTCAAAGAGATAGGGACTCTAATCCAAGGCAATAA

>Aco020569; *Ac*bHLH74
ATGATGGACTCTTCAAATCACCTTGATCACCACCACCACCACTACCACCACCACCAAGAAGGGCTTCATGGGTCCTCTTTAGAAGAAGCAGCTGCACCTTTAATCCATTGCGGGGGAACCAACCATGGATGGAACAACCAACCTCTCATATGGTAACCTTGGAAAACATATTTACTCCATGCCTTTAATAGCTATATATATATATATATAAAATATCTTTTGTTTAATTAGTTCCATGCATGTTTTTGAAATGGGTAGAGGATGTTTCTTATTTGCTGTTTTTTTTTCTTGTTTGGGTCAGAGCACATTGTGGTAGCATCTGGTTAATTCCTAGGATTTGGAATTTTCCTGCATGTATTTTTGTTTGTTACTTGTGTTGTCATTTTGCATATCCTAGGAATTTGTAGCCAATTAATTGAGTCATGCCAACATTAGAAAATGCAAAATAGTAGAGACCTATATATATATATATATATATATAGACTCTATCTTTAGGGTTAGACTCCAATAATTTTCACCCATATATGCTACAATTAAATAACTTTTTACCCTCCATGAAAAAGAATCCTCTCAGGTTCAATAATTGTAATCAAATTACACTAAAGGCATAGGTTTTAAATTCTTTCAAACATATTCATTTTTTGGCATGCAAGTTGCAATAACCTTCTTAAATTTTCTCTCCTTTTCTACAATAACTTTCTTTTTTTTTTTTATTTCTTTGAAAGGCCTCTTCTTATACATATTCTTAGGCTCTAAAACTTTTCTTTCATTTAGATACCGTATTCTTAAATTTTTCTCTCTTGTGGTGTGTCATTCAGGAATTGTGGTGACTTCAATGTACTAAATGAGAGTGAAGTAGTTCTCTCAAACCAAAGAGAGTTCATAAGAGCAACTTATCAAGATGAGCCCCTAACCCCTCCTCTAAGCTCAATAATGGTTCAAGATCTAGGGTTTCAGTGGTGTAGCAATGCAGATACCTTAATGAACCAACCAAACAACCAATCACACTTAGCAAGGATCAAAGAGGAGCTACCAACATCAGATAACTTTCCAAAGTTAAATGGGCTCATTAAAAACCCCACATACATTGACAACTACCAACTAAGTGAGAAGCTGTTCCTAAAAGCACTTCCTCAAAATGGCCACAACAATGCCCTACAGCCTCCGACCGATGATTTTCATTTCGGCCCGTCGAGTTTGGCGAATTTGGAAAGCGGTAGAGGAGGCTTCAGCATGGTCCTTCCGAGCGTTAACATCTCGCTACCGAGTTTGCCGCCGTTGCCGTTCTCAGGCTCCTTAGACATGGATCTTCAAGCTTTATTGGCTTCTACTAAACTTAGCAGGAGCTTTTTCGCACCTCCTTTCAATTCAATGGCACCCTTGTTAAAAGAAGATCCTATTTTTTATGGACCACCCCATGTTCAAGAAGCTGTTCATGGACCATTCAACAACAATCATAAGGTTGGTATATATAGATACACATAAACTTTATATGTATATCACTTGCTTCTCCAATAATAAACAGTTTAACTAGCTAGTTCCTAGCGATAGCTAGGCGTGATTGATAAGCATGGTAACATTACCAAACCTATAGCATTTAGTCCCATCATTCCTCTTCGTATGTACAAATATGTTGTGTTCTTTTGGTGTCGTTTCTAATTAATAATGAGATCAGTTCAACAACTGCTACCATTATTTTCTACGTTTTGGTCGGTGTACTAAATGATTGCTAAAGGTATTATTGTTGTTGCGATAATCATGAAGTTCTTGAACCTCATTTAGACTCATGTATAGTTACCCTTAATAAAAAATGCTTCTATATATACACACACACACTAAGTTATACGTATGATGGTAATATTTGTAGTAGTTATTGGATTATGCATATAAATATGATTTCATTTAGAAGTACCATGCACAAATATGAACATTTGCGCATGTACTTGTATATTCATGTCACCTTCTTTATCTCTTTGACGTGTACTTGTATACACTTTGCAAGGTACAGCTTGATGGGCTTTATATATGATGTATGAAAATGTAGGGACCTTTAAGGATTTTTGAAATAATTTCTTTAGCTACTCATTGCACATGATGTGCCTTATTTTTCTCTTTCTTTCAAATTAGACCAATGGAACCATAAAGTACAAGAGGCTCTTTTTTCAGCTTTTAGTGTGACATGCATCATCTTTTACACAAAGGATAAATGGAGTTGCGAAAGAAAAGATAAACAAATGAGAAAAGCAAATGAAACTGTGCTTTTTTTTGGAGTTCTCTACTAGCATTAGTTGAAGTTAAAAACCAAAAAAGAAAGAAAGAAAAGCTAAGAACTTTTTCCTTTAATTGATTATTACTATTGCTTCTTTGAAGGTGCCATGTATAGTGAGTGGGGTTTCAGAAGCAGAAGCAAATTGCATTGTGAACCGCAGAGCATCTCAAACAGTTGTGGCTAGGAAGCCTCGAATAGAGTCACGTTCCTCATTCTCATCTTTCAAGGTATATATATATATATATATAGTTTGTTGCTCGCTCAAGCGTAGTTTGATAATGTTGTGGGTAAAAGTATTATTTTTATACTATAGATTAGGGTCTAAAAGATTCAGAAGTACTTTTACTCTTACTTTCTCTTAAGTTTATAAAATTACGGTTGCAATTGGCGCCAAAGAAGCATGCGACTACTAACTCCTCCGCCATGATAGTTTAATAATGTGGTGGCTAAAAGTATCATGTTTATACTATAAAGCATCTAAAAGATTTTGAGACACCTTTAATCTTACCTTCTTTTAAATTTATAAAATTGCGATTGCAACTTATTATATAAAACAAGAAAAGATTGTCAGCACTTTTAAATTTTTTGACCTTTTATATATAGTATAAAAATGGTATTTTTAGTCACCACATTATCAAACTATAGCTAGGTCTTAGAATAATTCAAGAATATTAAGTGTTTTTTTTAAATTAATATGCCCTCCTAGCTTTATTTGTTATTGTTACTAATATGTTTAGGTGTGGGTGAAATTAATTAATAGGTGAGGAAGGAGAAATTAGGAGATAGGATTGCAGCTCTACAGCAGTTGGTAGCACCCTTTGGAAAGGTAACCTTTAATTCATCCTCTCATGGCTTAGGAATTTAAAGGTTAAAAAAAAAAAAAATTATTGCCACATGCATATGAACATTGATCCTCTTAATCACTACACATTACCATTTGAGCTTTAGAAAATTAAAATAAATTTTCTGAAATTTATAATTACTTTAGTTAATTATCATCCTGCTCGATTTGAACTTTGTTATTTTTTCTTAACAATTAATCTTTACTCACATGAAAACCAAGTAAACCCATATGATTCCCTTCCATTGAATTGCTTAGATAAGTTTTATTCTACATATGTGTATTAAAACTATATAAAGTGGAATCAATTTATGATCACTATGAAATATAACTACTTAATTAACACTAGTTACAATTGAAAGTCCTATACTATTTCTTACATAACAGGTAATTAATAATGTGCAAATACAATAAAAGGTTTTTATTATAATTATTAACTGCATAATCCAATATTAAGAAACACATGCAACACCTGTGCCTCTACTTATTTAGTTTTTCCCTTGTTTTTACTTTCTCTACCTTAATATAATTATTAATTTCCTTAATCTTGCGTATGCAGACCGATACAGCATCGGTTTTGATGGAAGCGATTGGATACATTAAATTTCTTCAAGAACAAGTCAAGGTAACTAATTATATATAAATCATGTTTATGCGGTTCGATCATTTTAAAGGAGTTTATTATAGCTAATTAGTTAAGTATCATCAAACATGAAAAACAAAAGCATTAATTTCTAAATATATTAATTTAAACAATGGCACAATATAGAGCTCACTCCTTTTACTGTATATGTGCAAATTAACATCTAATCTTTATTGATTTCTCAATTAGAACTAGATAAATAAAAATATTTTGTGTAAACCATATATATGTAATTTCAATTTATTATTTATTTCTGAAAAGTTTATAATGTCATCATGGTTTTCTTTCTTCTTTCTTTTTTTTTCTTTTTTTTCTTTTTGAAGGTGACATGTTATCTATGCTATTCTTAATTGGATTGATAACTTAATTTAAATTTTTTTCTCTTAATTTGTTGCAGACACTTAGTGTACCATATATGAGGCCCTCCAACAACAAGAAATTAAGGACAACACAAGGGGTATGTAACAATTCCCTACATATATTAATTCCTAATCATCAAAAAGCTATACATGTAGAGTACTATATATTCAACTCTCTTCTTAATTTGTTTCTTCATTGGCTTGCTATGGTACAAAATTTTCTTTTCATCTAATTTACTCATCTGAAGTTACAGGATAATTTGTGAAAACTATTTCCATGTTGACATTCTAGTGCATTGATATCAACATATATTCAGTGATTTATCCTACATTCATATATTTTTGAATTTTCAAAAGCGAGTGAGTTTCAACTCATGAATTGTTTATTATGTATAGCATGTCAAAATTAACCTGCAATGAGAAGTGTTCATCTCAAAAAAGATCAATTTGTTGTTGTACTATATTGTCATCGAAGTGACCAAACGTTTGCAACGAGGTTATGCGAAAGGTGGTAAATCAATTTAGTAGATCTTCGATCTCTCTTTTCTTTTTTTGTTAAAGTAAAAAAGAAACAAACTAAGAAGCTATCGTAGAGAAAACAAATTTTAGATAGAGACATATCTTGAATCGTTGAATAGCTAGTTTTGCTATCACAAAACTTTTGATGCATTTCCAACATACATTATTCTTTACTACGCGACTTTAAGTTCGAACTTACTATCTACAAGAATCATTGTTCAATAAGATTTGAAATTCGATAATCATATATGGCACTATTTGAACAATTTTGCCTGCAAGGGTATTTAAAAAAGAAAAACAAGTTTCTATTCATTGTTGGCTTATTATTATAATAATGAATCGGTTAAGAGAAAACAAATATCTCAATTATTACTATAGCATCTTTAGAACATCTCGAAGTCTATCCAAAACTGAATTGCTTGGCCAACTGGGGACTCACTAGCAAAAAGCTTTTTTTTTGGTTTTCTGTAGATGAGTTCTTTCAATCTGTACCACTCGATTAATTGAATATATAATTAACTTTGCAATTATTTTCTCTCACATGGTTTCTCTCTCTTTGTATCTAGGCCTCAAATGAAGAGAAAAATGAGCCAAAGTTAGATCTAAGGAGCAGGGGACTTTGCTTGGTGCCATTGTCATGCACATCATATGTCACAAATGAGAATGGAGGGATGTGGTCACCATCCAATTACAGGGGAAGTGCTTGATAAGTAGACTCATGTAGTAAAATTAATGGTATACACAAGGC

>Aco005342; *Ac*bHLH75
CGACGACACTCTATATATCGCCCCGTGTTTCATCTACGACTCTACACAAAAGCGCTCCCTTTTTCCCCCAATCCTCTCGATCGAAGAGCCCCTCTTCGAAGTCTCCCGTAAATCCGCTCCAAGTTCCGATTTTGATCTCGTCCTCGTCCAATTCGATTCGTCCCCGTAGAAACCCTAATCTCGGATTCCTCGATCTCCTCTAATTTTCGGTAATTTTCTGTTGGTTTTTTCTATTGAATTATGGAAGGCTTTTGTTTTTTGGATTCAATTAGAGCTTCTCTTTTGGTTAAATTCTTGTTGGTTGATCGATTCAAATCGTTATTAATAGCGTTTAATTTGATAAATTTTATCTATTGATTGATAGATTCCGGATCAAAAGAAGAAGAACATGCCGTGGGCACCATTGTATTCGAATTACTACACGAGCAAGAAATCCGTTTGCTTTGTCGTATTTTTCCGGGTGATTGTGTATGATCTCCGTGCGCTTTCGAATTCATCAGATAAAACTTTTTCTTTTTGGTTGCAAATTAATGTTTTTGGTGGGTTTTTGGTGCAGGTGAGGTTGCTACAAATTTAGGTGTTTTCTTTTCAAATTTTTTTGTTCTTCATATCGGTATTGGATTTGTGAGGATGCACATTGAGATCGATCTAAAGAGGTATCGAACGGTGGAGGAGGTCGGCGAAATAATAATGAATGTTAGAATCATTGCTTACTGCCAATTGATGCAAGTTTGTCAGGTGAATCATATTCTTCTATGTGTTTTTTTCTCCGATTTTATCTCGCTTTCTCTAAATGTTTCTTCATTAGATCAATTTTTATTTGACGCACCTTATTAATCACGGAGCGTTGATTAAGTAGTTTCAATCTGAGCTGATTCTTTTAGTTCGTAAAAATAGTAATTTTACTCGTAGTTTCTGAATCATCAAGTTAATTAAGATATGAGTTTGCAAAGCTAACTTGGGGAAGCAAAATATTGAGCAATATAGAACTGTGGTATGCTTCTGTACAACTCTGCTCACCGCTGTACGATCACAATCCGCAAATTCTTATGATGTGCCCTGGTTACGGCTTACTGCGATTGTGATATTGTTACCCCCTTTATTGCGGCGTGTTTCGTGGCGGTTGGATGATGGAAATGGATGGCCTGCATGCTTTCGTGTACAGAAGCATCACTTGGGAAAAAAAAGAAAAAAAGAAAAAACGTTGATCGTTTATATGTACATATTCGTGAGCCCTAGTAGGGCTAGCTTTTTCATAGTTACCTCAGGATTTAGCATTGTATCTTCGATTAAACTTCGTGCTTTCTTTGCCTTATTATTTGATTTACATGTTTTCTTTGGCTTGTTATTTGTGAATTTCTGATTAATTATTCCCAAAATGGCAGCCTGAGTACTTCCGCCAGTTGCTTAAGCCTGTCACGTAGTTCCAAGTTCTCGGCACGACGCGATTTTAGCTTCGATCTGTCCTAAACTACATAAGGCAATTGAAATAATAGGATGGCTAACTTGGGGTAGAAGGTATTTGTCTCCACCTTTGTCGCTGTACGGAAAATAACACAGTTTCTGTGAAAAGGCATATTCCCTGTTTCAGTACTATAGACCCATGCAGGGGGACGCGAGAATTTTAAACGGGAAGGGCTTCCTCTCTCACGAATATGAACTGGGTTGTACCCAAAACTCAGCATTGAACGATCACTATCCATATAGCGTCCCTTTTCCGCCGCAGTTTCTATCTCCACTCAGTGGATTAGAGTTCCAGCCTTTGGAAAACTGCCCCAAAAACTTCATCATCTTTGACCACACTTATGACAAAGGTTCTGTCATGTTCCATCCTGCCTTGGTCCGTAAGCTCGGTTCGCCCAATTTGGACCTTTATCCATCGCATCACGCGGAGAAAAAGGGAAAAATTTCAAGCAGAAAGAATGATATTTGTCAGGACAAGTTCTCTTCCTCTTTCGAGGAGGACTCGAAAGATATTGACGCATTGCTTTGCTCGGACGAGGAGGGCAATGATGGAAAAAAAGATGATGATGATGATGATGATGATGATGATGTGGTCAGTACGGGGCGAACTCCTGATTTTTCTTGCTCAGGAAAACATGAACCGAGCTTTTCTCAAGATTTCACGTTTGATGGATCAATTAGTAAGGAGAAAAAGAGAGAAAAAATGAGAAAGATGGTCAGAGTTCTTAGGGAAATAATCCCTGGCGGCGACCGGTTGGACGCTCCCGCTGTTCTCGACGAAGCTGTTAGATATCTGAAGTATCTCAAGGTGGAAGTAAAGAAGCTCGGGTTACAAAACTTTGATGACCAAAGCTGCGATTGA

>Aco005770; *Ac*bHLH76
ATGGATAGTTTGGGTTGGAGCAATCCGACACCCATCCAACATGAGATTTCCACTTCGACATGCATTTGGAGCACCCAGTACGATAACTTCTCCGAACCGAAGGAGTACTACAGCAACATAAATGATGACGTTGAGCTCCACGAGTGTATCTACAGCACAGGTTTGGAGCTTCAAGGCGTTCGCGCATGCGACACGATTAGTTCGAGTAACATCGAAGGAGTTTGTGATGATCAGATGGTGGGAATGCTCGCTCCAGCGCCTTTCGACTCCATGGATGGCATTTCAAGTCTCGATCTCTTGCAGCCCCAGCCCCAGGAAATTATCAAGTTGGTGGCCGATTCATCAGTTCTCGCGAGATCACAACAAGCGACGACCGCGTGGTGCGCCGATATTATTCTTTCTAGTTCTGATCAGTTGTTATGCTCTTCTCTCGTGCCGATCAGTAGACCCACGTTCGTCGATTCACAGCGCGCTCTACTCGAAAACCTTTCGAGCGGTGAAAATTTCCAAAACTTGAGTTACAGTACCGGCAACATCTCTTCGGGGGAGTCCGAGAACTGTGCTTCCTTCGACCGCAACACGCTGAATCGGGACGAGGCGGCATCGCAGAGCTCTTCGAACCCGGAGCCTCCCCAGCTTGAGTTCTTTAACCCGAAGGCACCGAACCCTCATAAGAGAAAGCTGGAGGAGGAGTGTGAAGGAATATTGTTGGGAGAGAAGAGCCATTTATGCAGCCTTCTTCGATCCAGCTCTCCGGCTATGGAAGGAGGCTTCCAGATAGTCTTCGGCGGAAATAGCAGCAACTCGTCGAAAGGCAAGAGATCGAAAGGCGAGAAGAATTCGGGGCAGTCGACGAGCAACAACATCGAATTTTCTCAGGAGAGCAACTACGAGCCCGACACGGAGGCGATCGCGCAGGTGAAGGAGATGATATACAGGGCTGCGGCCATGAGGCCGTTGAATCTTGGGGCGGAAGAGGCGGCGGAGAGGCCGAAGAGGAAGAATGTGAGGATCTCAAGCGACCCGCAAACTGTTGCAGCAAGGCATAGGAGAGAGAGGATAAGCGAAAGGCTTAGGGTTCTTCAGAGGCTGGTCCCAGGAGGGAGCAAGATGGACACCGCATCCATGCTTGATGAGGCTGCAAACTATCTCAAGTTCCTCAAGTCCCAGGTCAGAGCATTGGAAACCCTAGGAGGAAGCAAGGTTGATGGTTCCATCATCAACAGCATGCAGTAG

>Aco021761; *Ac*bHLH77
CTTTAGGAAAAGAGGAGTAGAAAGAAAGGTTTACAAAGCAATACTAACTATACCCATCCCTTTAATTTGAGACCTCAAAAAGCTTCATCACCAAGGAAGTGAAGCTAAGCTAAGCAACACATATTATTGATTCAAGAGGAGATAGAAATAATAAAGAAGAGTTATCAGTTATTAGTAGAGTAATCTTTAAGGCATAAGGAATGAATAGAGCCATGTTTGAGAGCTCTCTTGTGCAGCAAATGATGGTAGGGGGGAACCCTAGTTGGTGGAACAACATGGATAACATAAAGCCACCACATCAAGAGATCTCTCCACTTCTGCCTTGCTCAACCTCTTCGGCGGGGGCGGTCGCATCGTCGACATCGTCATCGTCGTCGTCGTCATCTTCATCTTCTTCTTCTTTGAACTCTTTCCCTATGTATCCTCAGTACACTCAGTCATCTAGGTTTCTTCCCTTCACCTCTTGGCAGGATAATCAAGGTCTACCAGACCCATGGAACCAGATGCTGCTGTAATATATGATCTCTCTCTCTCTCTCTCTCTCTCTCTCTCTCTCTCTCTCTCTCTCTCTCTCTCTATCAAAATCAAGGAGGATTTTGATGTGGAACTAACAACTCACTGTATTGAGTTCTTTTCATTCAACAAACACAGTATCATAAATTGCTTTTTATATATGTTTTCTTACAAAACTCCAATCAACTATATATATCTGTTAAACTTTTTGAAAAAAAAAAAAAATTCGACGTAATATATCTATTGTTCTTGGCCTCATGAAACATCCCACCATGCAATTCTCATGCATGTCTATCTTTTTTCCTTGGAGTTTTTTCTTTTTCTTTTTCTTCCAACTAAAATTATGATTATAATAATGGCTACCACTTAGCCTTTACAAGAAGAACAATATACATATATACATGTGTTGGGCATGTTGCAGGGGAGGAGTGATGGAAGAAGAGGAGAGATGCGGCTTTGGTCTGCAAACAAAGAAAGTAGAGAATTGGGAGGATCATCAACTGTATCCATCAGGAAACAGCCATGTATTTGGTGCAAAGCAAGAGAACTTGGAAAGATCAGCATACCTATGTAAGAAGTATGAAAATGATCAACAAGTTCTCAAAACTACTTCTCCTTGGGCCCAATCCCTACCAGCTTCTTCTACAAAGTCTTGTGTCACCACAAACAGTTTGAGCACCAACATGCTGGACTTCTCCCACAGCAAAGAAGGGAAAAGGCACCTTCAATCAGAACAATCATCTGAGGTTAATTAGCCATAGCTTTTGTTTGTTCTTGTTTGATTCCTACAAGTGCATCAACTTACTTATTCTTATTACTTCTCCATGAAAGCTATAAAGTTTTCAATTCATAGAGGCTTAAATATGAAGATCATAGATGTTAATTATGAAATTCTTTATTTACTGAATGATTTCCACATGTTTTTAATTTCTTCTGGCAGTCCAACAGCACAGGGACTGGATCACCTTTCAAGAAGGCCAGAGTTCAAGCATCATCAGCCCAATCTCCCTTGATCAAGGTTTTATTCAATTTGTTAAATTTTTTTTTTAGTTTTTTAACATTTAAGAAGATGCTTTCATGCCCACAACTTAGAATGATAATTGATTAATTAGTAACTGAGATAATGACCATGAAAAATAATAATAATGATGAGGGTATTAATTATTATTATTCTTTCAGGTGAGGAAAGAGAAGCTGGGAGATAGAATATCAGCTCTTCACCAGCTAGTCTCTCCATTTGGAAAGGTATATATTGCTGCTGCTCCATGCATGCAACAACTGTAGATCTAATGCAGTAGTACTAGAAAACTTCATAATTAGCTTACAGTTAATTCTCCCCCACTGTTCTAATCTTAATTTGTTTCCTGTGCTTTTTTGACCTCAATCTTTGTTCCCTACTCTCCCTAAAAGTGTTTCAATTTATCCTTATCTTTATTTATGGTTAAAAAACAAAAATAATTATCAGCGAGCAACCGGTGCATATCTATACATCCAAGCACTAAGTTTTCGATTCAGAGCCGTACCTACACCATATAAATTTTTTTTTTTTTTTCTCTAACAAAATGACTAAATAAAACAAATCTAATAGCTCGGGATAGGAAATAAATGTTAAACTCATAGTTGAGCTCCAATGAACTTTTAAAAGCACAGGAGCATCTGTGCTGGTGGAATTTTACCTGTTGAATCGTCTTAACGTGACCTACCGTTAATAGTGTTGGACGAATTTTGAGACAATTCGGCAGCTAAAAACTCACAAGCTCTTTAATGTTAATGTTGGGGGATCAAAATAAATTATCCGAATAAAGTTAGTCTGACAAAAGCGCCTTTAATTTGCAGACTGACACTGCTTCTGTCCTGCTAGAAGCCATTGGCTACATCAGATTCCTCCAGGGTCAAATTGAGGTCTCTCTCTCTCTTTCTCTCTCTCTCTCTCTCTCTCTCATACCTGGACTGAAGCAGATCATTTTGGGAGTGCTTCAGCGCACGCAGTAAAAGTTGCTGCACTCCCCAAAGTGTTGTTTTTGTGCATCACATCTTCTTGTTTTTTAGTTGTCCTTGTGTTGTCATGTGTTTCAGGGATTATATATATATATATATATATATATAACCAACTCCTCACTTCCCCAGTACACTCCTTAACCTTTTCTTTTTTCTTTTTTTTTTTTACTTTTTTAATGTAGGCCCTCAGCTTGCCCTACCTGGATAGTGGGTCAAGGAACCCCAGGCATTGTGTAAGTAAACTATATATATATATATATATATAAACACCATCCACTTCACTGTTCCTACCATACATAGATATATATGTGTGGCATCTATCTCTCTCTCTCTAGTGGTTAATTGCACCATTGTTACCTTTACCTACACAATTGTTCACTTATTAAATACTTAATCTCATAATTTTTTAATTGAATCTCTTATGTTTTTCAACTGTTTGAGTCAAGTTAATTAATTAATTTCTTCTATTGAACATTTATTTATCGGAACGAAAGTTAATTATCGCAAGAAACAACACATTTATATCATGACAAATTCTTATCAAAAATAAAGAATTTACCAATGGAAGGGAAGAGACTCGATTGAAATAATTAATAAATAATTAAAAGGTTAGGAACCAATGAAAAAGTACTGAAAAAAGAAAAGGTTAGGGACCAATGGTATTATTTAAGTATTAACCCTTGTTCTAATGGTTTTTTGGTATCTTAGAGCAAGATTTATTGACTCCTAAATTTGGTATCTTAGCAGCAAGTTATATATATTATATATATATATAGAGAGTAAAGCTACTGTACTCTTATGAGTATTGGGTCCTTCGTATTCATAAGTTGTTTTCGATGATGGGCTTCGAATCGACGATCCACTCATTAAATATGATCTAGAGCATTTGAAATTTCTAAAAAATAAATTTCGTGATTTTCGAAATCATAATAAAGTCCATCAAACTGGCATAAAAATGAACGGTCAAAATCGAACGACGTCCTAAAAATGGTATTATCGGATCCTTCAATTTAAGATCGGAGTTATTGATCTTTATCTAGGTAGTGAATAGAATTTTCTATCAAAAATTCAACCTATTCCGATTCTTTTACACCGTTAAACTAGCAAGTATCCCATACCGGCCCGTTAAAAATTGTTAATTTTGTGAACTTTGATCGTAAGGCAAATGATGTCGAAAAATTATAAAATTGATTTCTAGAAGTTTTAAATGCTCTAGATAATGTTTAACGCGTATGGATCGTCAATTCGGAAGCTCTATCATCAAAAAAACTTATGAGTACGAGGGCGATCGTACTCATAATTAAAAGTATAGTAGCCGGGCTATATATTATATATAGAGTTGGACTGGGTTACTATTAGTAGCAAAATTTCATTTGTTGCTACCAGTTATTTAGCCTTTGGATCAACTCTTTTCATCATTTCTAACCATTGGATTAAATAGTATAACCCAGTGGGGACCACTCAACCCTAGGGGACCACTCAACTGTAACTGACTAATATCATCCTAACCCTATTTATTTCATCTAGGGGTTAAAAAACTTGATAGCGAAAAGAGGTTTTACTATTAATAGTATTCCAGTCTAATTCTATATATATATTATATATTATTATATTATATATATATGGAGTCCGAACGTACGGTGCTTGTAAAAGTATCAAATATTTGGTGTTTGTAAATTTTTTACCGTTTAGATCTACGCCTTTGATCATTTTCATCCGTTAGATTATACTATTCAACCAACCACCCACTTTAACCCTAGGGGGCCCACATCATCTTAACCACACATTCTTTAATCCAAGGGCTAAAAACCTACAAGCACCAATGACTTGGTACTTTTAAAAGCATAGGAGCCTCAAATTTTATTTATATAATATATATAATATATATATATATATATATGTATGACTACTATGCTATTAGGAGCACAGTAGCCCTTATGCTCCTAAGTTTTTAACTTTCTAACCATTCATCAATTTTGATGGGTGATTAGGGTGAGAGAGAGAAGAGAAATTGGAAACAAATTCAAAGAGAAGAGAAATTGAGATACCTAATTTTTCCTCAATTTCTCTTCTCTCTCACATCCTAACTACCTATCAAAGTTGATAAATAGTTAGAAGCTTAAGAGCACAAGGGCTACTGTGCTCCTAATAGCACAGTAGCCCTATTCTCTCTCTCTCTCTCTCTCTCTCTCTCTCTCTCTCTCTATAGAGAGAGAGAGAAACTACTGCACTATCTATAGTATCGAAGCTTCAATACTATAGTGTCATTTTGTATCTTAGGTTGCTCAAATCAACAATCCACACCATTAAATATGATTTAGAATAAATAAAGTATCAATGACTTGGTGCTCCAAGTTACGCGCGCACACGCGCACACACACACACACATATATATATATAGAGAGAGAGAGAGAGTGTCCGCTATGGTGCTTTTGAAAACACCAAGTACTTGGTGCTCACAAGTATTCCATCATTAGATCTATCAGTTTGACCAATTTCATCCTTTAGATCATGCTATTTCATCAACCACTCACTCAATCCTAGGGCACTACCATTATCCTAACCCTACAAATCTTATCCAAAGGCCAAAAATCCAAAAGCACCAATGCTTTAAAAAATATAAGAGCTCAATTTTATATAAATGGAACTAGGTTATTATATTATTAATAGCACTAAGTCATTAATGCTGTTAGGTTTTTGCTATTGGATTAAGATATATGTAGCTAGGATGATAGTAGTCCCCTAGGCTGCGTTTGGTTACAGGATATAATAGGAATAATACATTTTATTCCTGCAATTCTGCCAAACATAAGTAAAATTGGAAGAGATATTTAATTCCGGTCAAAGCGGGTTATTCTTGACAATTCTAGAATAACCCGTTAACAACTATTCCTCTCCAACAGTGGAATAGCTGCGTCCTTCAATTTATACCAAAGGCAGTAAGTGAAAAAGTTGCCTCGTTATTTGAGCCCACCTACCCTACTCTATACTCCTTAGCCAAATGCCTCTTTTAGTTAAAACTTTTTACAATATTTTATATTATTGTGTATACTATTTTTGATTAATTATATCTAAGGTAAAAATATTTTATAATTAATTATTAAAATATTTTATAAATAATATGAATATTAAAATATTAAATATAATTTTCTTTCAGTAAAATATTATATTGCATATATTATGTTTTATTTGAAATGTTGTTCATTTTATTAAATATTACATATAAAAGAGGCACACACAAGGACAATATTGGCATTTTAGAAAAATTCAATCTACTTTTACTATTATTCTCTATTATTCCTAAGCAATTTTCCAAACATAAATTTTCTTATTCTTCTGAAAAAGAGAATTTCATACCAAATATAAAATTCATTTTATTCTTTTGTATCCTAGAATACACCCTAAATCCAAGAATAAAAATATTTATACTTCTAAACATTATTCCATTAACAAACACAACCTTAGGGTTGAGTGGGTAATTGGTTGACTAGTATGATTTATTGGTGGAAATAGTCAAAGGGATAAATCTACCAGTGGAAAACTTGGTAGTATACCAAGTGCTTGGTGCTATTAATAGCATAGCAGCCGGACTAGTCCAACTACTATACTCTTATAAGTATGATCGTTTAGTACTCATAAGCCGTTTTTAATGATAGAATTTTCGTATCGACGATCCATATTGTTAAACAAAATTTAGAGCATTTGAAACTTCTAGAAATTAAATTTTATAATTTTTCAACGTCATTTACCTTATGATTAAAAAAAATCTCAAAATTTATAATTTTAACGGTCGATATGAGATACTTACTAGTTTAACCATGTAAAAGAAATGGAGTTGTTTGAATTTTCGGTGAAAATTCTATTCACTATCTACATCAATAATTCCAATCTCAAATTAAAAAACTTTGATCCTCTATTTTTTTGGAGGTCTTTCAATTATAAGCATTCATTTTACGCCAGCTTGATGTATTTTATTATAATTTTAAAAAAATTACGAAATTCATTTTTCATATGTTCAAATGCTCTAGATCATATTTAATAATATGAATCGTCGACTTAGAAGCTCCGTCATTAAAAACAACTTATGAGTACGAAGAAAGAGAGAGAAAGAGAAAGCAAAGAGTTACACTAGTATGCTTTCAAAAACACGAAGGACTCCGTACTGTCAAATTTATTTTCGATGATTGGACATTCAAATATTAGAATTATCTGGAGAGCAAATTTGATTTATAGATATTTCAGTATAGATCATATCTAAAGAAATCGATCGTCAAATCAAATGTGCTATCATCGAAAACTAAAGTGAAAGTATGAAAGCCTTCATACTTTCGAAAACATACGAGCCTACTTATGTAGAGATAGAATATTATGGTCTCATGAGTAGAAAGGCCTCTGGAAATTTTTTTTCGATAATGAGACATCCGATTTGATGATCGGCTCTATTAAATATGATTTACACTATTGAAACTATTTAGAAACTCAATTTTATAATTTTTTTTTCTTATTTGCCTAGTGAACAAATGGTCTCAAAATAAACGGTTGAAAATAAAAATCTCACAAAAAATAGTCCCAGATGACTTAAATCGAAAGTATTGATCTTGCTCTTGATGGAAAAATAATTGTTTATTAAAATTCACCTAATTTGAATTTTTGTATACCTTTGAACTTACAAACATACCACATTGGCTACTAAAATAGTAATTTTTGAGATTCTAATCACTTGGCAAATGATGTTGAAAAATTATAAAATTAATTTCTAGATAGTTCCAATAGCGTATATCATGTCTAAAAGAACTGATCGTCAAATTAGATATCCCATCTTAGTCTTCCAAGAACATGATGTGTCCAAAATTGTAGGAGAGAAGTGACTCGGTTGTTTTTTTCTTTTTTTAAGATAAAAGCATGCTATCTTTTTCATTTTTTAGGAAAATGAATTTAGCTATACGAGTAAGACAACTAGATCTCCAAAATGGAAGATCAAAATACTAATAAAAAAATATAACAAAAAAGAAAAGAAACTGAACAAGAACCCTAACGCATCATTACCTACAATTAATGACTATTATATATATTTGATTTGATGCACATGCTTTGTGTTTAACATCAGGATGCCGCTGCTTATGAGGAACCAAAGAAGGATTTGAGGAGTAGAGGACTATGCCTGGTTCCTGTGTCATTCACCCTTCATGTTGGAGGAGACAATGGAGCTGATTACTGGGCTCCGGCTTTCGGCGAGGAATTCCGATGAGAAGCTCGATCGAACGCGCGCGCACCGATCGACCAATCAACTCGCGCCTGATCAGTTTCGATGCATGCATGGCGGAGAAAACATGAACAATTGTTCAAGGTGAGATAGATTGGTAAATGTAGGACATCAAACTGTAACATAAACAGCAAAATTAGGTGGTAGGTACATAATTAAACTAATTAATAATTTGGTGAGGGGCTCCTTTGCTCTCTTGTAACTTGAATTTTATTGAATGTATTGTAGTGGTATTGTAGTGGTATTGTAGTATGTTAGATAAATATCATGAAGATTTATGTATGGTTGACGATTTTATACATATATATAAAATCAATAGCACCAAGAGCTTGGTGCTGTTAGTTTTTTAA

>Aco000179; *Ac*bHLH78

TCTCTCTCTCTCTCTCTCTCCTTCAATACTCTTCTTCCATACCTCACCTCACTCTCTCTTGTCTCTCAAAACCTAGCTGGTTCACACTCTCTCTATTACTTGCTTACTATATATCTCTTGCTTAGGTACTTCACTCCCTTCAAACTCTATCTAGTTCATCACTAGTTACTCAACCTACACTAAAACACCACATTCTTCATTGACAAAGATGTCAAGTCGGAGGTCGCGATCACGACAGTCGAGCTCGTCGCGAATCACCGAAGAGCAGATCAACGATCTCGTGTCCAAGTTGCAATCGCTTCTCCCCGAAGCTCGCATTAGGAGCAACGATAGGGTATATAAGTTTCTAGTTAACCCAACTTACAGAGAAAAAAAAAAAATATCTATCAGATGCGTCTGAATGAGACCAATTTGACAGATCTTGTGTGCGTGTGTGTGTGGTGTGTGTATGTGTGTTTCCACTGCAACTTTTCCTATAGTTTTCCTCAACCTTATTTTACTCTTGCTTAGCTTAATATATATTGCAATATCGCTTGCTAATTACCAGAGTTATATATTCTTCTTCAATGTAGGTTTCCGCGGCTAAAGTGTTGCAAGAAACTTGCAACTATATTAGGGGCTTGCATCGCGAGGTCGACGACCTGAGCGAGAGGCTCTCGGAGTTGCTCGCCACCACCGACATGAGCAGTGCTCAAGCCGCGATCATCCGTAGCTTGCTCATGTAAAATCGATCATTTTCCTAGAGTGACTAGGCTAGTAGATGAAAAAAAAAAATTAGGTGTTTATGTGCTTGGTTAATTTGCTGTGTTAACTACAAGATGTGTTCATAAGCACTCTTGGTTTGGTTGTAGCTTATTAATTACTTAGACTTGTCTTTCACCACCTTTGGTTGATGTACTATTAAGCCAATGCTTGAATATCCTTCTCTTCTTCTTCTTTTTGTGCAAATGCATGCACCTATGAGCTAAATTTTTTGTTTTAATTAATATTGTAAGAGTGGTATGAGCCCCATAACTTTTATTCTTTTGGTCTAAC

>Aco000186; *Ac*bHLH79
GGGGAAAACGAAAACCCTAGATTCTTCCTTTGTTCAATTCTCCCTTCGATTGCGTCCTCTTCGATCTCGTTTCCCCCAATTTCGTAAAGGTAAAATGGTTCCGATCTCGAATCCCCAATCTTTTCCGCGGTTTCTGAGATGGCGATTTGGTGGGTCCGTGTTGGAGGGGTTGATTCGGGTTTTTTTCGGGTGTTTTCGATGCGAATTTGGTGAATTGTTTCTAGTTTTGATGATAAATTTAGATTTTTTTGGCCCCGGTGTTTAGATTTTGGGAGTTGGAAATTCAGGGGTTTTGATTTGGTTTGTGACTGAGAATTGGTAAGAGAGGAGAGGAGTTTCATGAATTCGGTTTAATTTATGGAAAAAATCTGATAATTTGTTCGCAATTATTGATGATTTAGCCGCATTTTTTAGACAAAATTGTATCTTTTTTAATGTTTTTTGTTTCTTAAGGTCATGGTCTTCAAAAGATGGAGTTTTATGAACAAAACACACACACACACACACACACACATATATATATATATATATATATATATATCACTAACTTTTTATCTGAAAATTTGATATAAAAGGGGAAGAGTATTAGGAATTCGATGTAGCTATTTATAGGATGTTTCAAGTGAGATGCAATTACTTCTTATTTATTTATTGATCTATTTAATTTAACTTGTGTTATATATGATAAAGAAGTGAAAAAGGGGATTTGCGTGATGCTGGACAGCAGCCAGAGCACGGTCTCCGAAAATCCAACTTCGACGGCTAACAGTGTCACGGAAAAATCGACCGATGGGTAGATCTAAAACTCGCCATAGTTTGATTATAGTTGTTGCAACTTACGAATTACTTACCGTACAATTCATTAATCCATTCGTGTGCTTTATATTTAGTTATCTTGGACGACCATTTGTATGAAACATCGATTGCTTTTACGGAGCGTAATCTACAATGAACTTCATTTCTTTGGTTGTATACGCATTATAGTTTTATGTAATTTTTTCCCCCTCTTCTTTTGGGCATTTACTTCCTAAAGGGGCTTTTCTCTGTTAATCTACGGCTCTGTTCAGTTGCATGGTAGGGTACGTGGATCCTTATGCGGTACACATTGTTAGAAGCATGTCGGCTTGTATAGTGGGATAAGCGGTTGCGAACGGAGAAAATTGAACACAACTGCCATCCTGTACTGTTCCGAATGGAAAGCAAATGAGCGTGCACACAAGTGAAACGTGAAATGCTACGCCGCATGCCCAAATGAGCCTTAAATTTTACGTACATATTTGGAAGGAGTCGCATATTGCAATTAAGTTACATAAGGATTTTCTGGTGTACAAGGCTCTCGCCACCCTTATTTGGAGAGGCTGTTTCTATGTTTTGAACCCGTGCCACTAAATAATATAGTGGCAAGCTCTTACTTCACGCATAAAACTACACTTTAGTAGCATCTATATACATTCATGATGAAACCTCACCAAATATGTTGCTAGCATCGGAAGAAGCTTGTAGCAATTTTTTTCCCCCCAAATACAAGTTCTTAGCAATTATTAATAGTGAACAACAGGAAAGCTTACTTACAGTATGAAAGTAATGTTAGTTATTTCATTTTTTCAGCCGTTTCCTTATATGTTTATGAAATCTTTTGTTGTTTTTTTCACCGAAATTGTCTCTTCACCATTACTTTTCCCATGCTTCTGCTTGTGAATCTTGTATAAACTTTTTGTTTATCGAGTTCTTATAAACCTCATTTGCTGACACTGCAGCAAATTTTGGTCCGTATCTGAAAATTCCCATGATGCAGGTCTCCTCCTAACAGAAAATGCCAAGGGAAGGTCCCGAAGAAAATACACAAAGCTGAGAGAGAGAAACTTAAACGCGACCAGTTAAATGACCTCTTCCTTGAGCTGGGTAATTTGCTAGGTAAGCATATTTATAATCTGTGATTTATTCTACTTAATCAGCATTTATTGAGAGCTGTTATAATTGTGCGTGGTCCGAATCCTATCTATTAAAAAGCTCAAGTTAATTGTTGTTCCAGTCAAACTTTACTTTTCATCAGAATATCAATTCTAACTTTTATGTTAGAGTAAGGAGTTATGCGAGAAAAGAAACACTTTTCTCCCTAGACTCTTCGTCTGATGTTCTAAAGTTTTATCCACATTCTCTATTCAATGCAAATCCCAATATTAAAGCCAAACAAATGACGAGCATAAAGGTAAAAAGTAACAATCATCATGAGAATAAAATATCTTATGCCAATCTCTCTAATTTCTCAACTAAACATGTATTTTTTTCCTTTTTGGCGCGTCTGGATAAACGCTACCATGACTTAACAGTTTAATGTTATCACAGATTCTATTCTATAGCTTTATAAGTAGAAATTGCTGCTTCAATGAGCATCTCAGCTGAATATAGTTATTTTATCATTTATTAGGTGTATATTGGTTGTACATATAACAGATATAACTATATTTGAGGCGCGGTCTCTTATGCTCTCTAGCCTATACCCACTGTTCAATCCCTTGTGAGGATGGAAATATATCGACATTCGTTTTAATAATGGTAGTAATTTGTGTAATACCATTTAATCCAATATCTATTAGCATCTAATTTCTCAATCAGAATTGTGTAGACAATTATTTGTTTATTTAAGCATAAATAGCACCAAATCTACACATCATATGCATAAACTGGATAGATATTTTCAGATGGAACTGCATTATATTATGGGAAAACTTCAAAAACCTCCCCTGTGGTTTCATAGTTTCTCACTTTGCTCCCCTGTGGTTTAAAATGTATCAATTTGCTCTCCTGTGGTTTCCTTTTTCTCTTTTTCTTATCAATTTCATTATTTTTTTTTTCTTAAATCAGTGATAAAGTTAAAATTAAAGGGCACTAAAGTGAATATTTGATAAATCTAGATGGGTATCTGAAGTTTTTTGTATATAATTTAATGAAATATTAACAAAAAAGCTATCGAAAAGATAAAAACGAAACCACAGGAGGGCAATTTGATACATTTGAAACCACAGGGGGCCAAAGTGAGAAGCTACGAAACCACAGGGGGGGTTTTTGAAGTTTTCCCTTATATTATCTTAGAAGTAAATAACGAAAGAATACATGAGAGGAGCTTTTTGAAAAATAACATTGCGTCTTCTGTTTGGTTCTCTGGTTGTTGCAGAATAACTTTCGAACTATTTGTTTTCCATTTTTCCGATAAGAACTAAATTTTGATTTGCAGAATCGGACCGACAAAATAGCGGAAAGGCATCCATATTGGGCGACACTACTCGAATATTGCGCGATCTCCTTTCTCAAGTGGAGTGTCTCAGAAAGGAAAATGCTGCTCTACAGACCGAATCTCGCTACGTAAGTTCTTTGATGTGATGTTGTGCTGGATTTTCATGGTATCACTATGCTCTAAATGGTCGCCGGTGTAGATGCTGACAATTTGTAATTAGGTAAATATGTATGGAGACCCCTCAACTATAAGCTATTCTGAAACAGACGCCTGAACTTTTTCTTTTTTGGATTGAGAACCCTCACCTTTCATTTACTTTTATAACTACATGCAAAATCAATAACTGTCCTAAAATTTTCGACCTATTCGGGACAGTAGTAAAATGCAGGGTTAAATTGATGCAACTTTTTATGCGTAAGGGTAATTGAGACACGGGCGAGCGAATTTGTACCCTTTTTTCATTTCTTCTTTAATTTAGCCGAGTCTTTAATCACCATGCATTGGATATTTGACAATAGGAATTGACCCTTCTGTTTTTTTTGCATCAAATGGGAATTCTAACATAATGCTCATGAGCAGGTAACTACAGAAAAGAACGAACTAAAAGACGAGAACACGGCACTCCAAACCCAAATTACCGAACTACAAAACGAGCTTCAGGCAAGAATGGGCTATGATCATCCGGTATGGACCAACCGCACAGATTCTCTAAGCCGTCTCGCCACCCCTCACCCTGCAATTTCGATCCAACCGCCCCACCACCACAACCGCCATCACCCCCAGCCACTGGCGCAGCCTCCGCCTGCCGTTATCGGGCTGGTCTGCCCAACCCCGCCCCGAGAGCTCCAGCTCTTCCCGGTGTCGGAGCCAGCACGGGAGGAGGGCGAGGCGCCCTCCAACGTGAGGCGGCCGCATGCGAGATACCCAGCTTCATTGGATTCTTGGCCAGGGCAACTGCTTCATACTAGCCTTGTAGTAACAAGAACACCAGATGAGCAATGTAGTAGTAGTGGCCTTACTCTCACAACCAACACTCTGGAAGAGAACCCTAGATAACTGATCTAAAAAGAAAACCCAGGCTTGTTGTCATTCATGTGAATAGAGACCTGCCATTTTGAGTTATGTAATTCGGTCTTCATGCAGATTGAGTTTCTATTTTGTATCTTCAGGTATCTGAATTCTTTTTGTTTCATCGGTGAAAATAATATTTGC

>Aco000272; *Ac*bHLH80
CCCCCTCCTCTCATAGGTTGGTCTCCTCTCCCTTTCTCTTAACCAATATTTTTGCTTAGTAACATCCCTCAATTTTGTGCATTTCCTAGGGTTTATTCCAACATCAACTTGGTTGAAAACAAGACCAGTTATTATTATTATTATTATTATTATTATTATTATTATTTTGTCACTCAATTTCTCTTCCATTTCTTCAAAATCTCATATTTCCTTTCCCCCTTTTATTTCTAGTACAATTTTGACATGGAGAAAAGGGACCCAAAAGCCTCTTCATCTTGGTTGATCTTAAATTTCCCACTATACAGCAGCATTTGATTCCTCACACCAACTTGATACTTTTCTTATTCAATCAGGGATCTACTTTCGTATCTATATAAATCATCAAACAGGGGAGAAATAATCAATCTACAAATGGAAGCATCCGCGGCGCGGTGGTTCGCCGATTTGGTAAGGAAATTCTGATCAATAATCCCCTTCAAAACTCCACATGATTGATGCCACGCATAATATCATCATCCTCATAAGTTGGATCTAATTATGTATATGTGAATCAGGGAATGGAAGATCCTATGTTCTTTCATCAGTGGGAGACAAGCCAGTTGGATCACTTCACCACACAGCAAATTGCCGCGGAGTTTGATCAAGACCTGCAGCAATCCCTCTCCTCGGAGAGCTGCACCAATTCATTCCCCAATTCCTACCATCCACCCACCACCGCCGCCGCCGCCGCCGCCGCCGTGTCGAACACCTTCCACTCCGCTTCCGCCGCGAATTCGAAGCCCCCCGAGAGGCCGAAGAAGGCGCTGAAAACCAACAGCTGGGACTCGTGCATGACCGCCGACCGCAGCTCCGCCGCCGCCGCCGCCGCCGCCGCCGCCGCGCCCGACGCTTCGTCCCCGACCATCCTGTCGTTCGGCAAGCCAGTCTCGCGGAAGAACGAGCCGAGCCTGTATTCGGAGTTCCTCGCGACCGTGAAGCCGAAGGAGGAGCTGGAGTTCTCTAACGTCCCTCATGGCCTGAAGAGAAGCTATGAGGCAATGAGTTTTCACGGCGTCAAGAAGGCGAGCGCGAATTCTCGACCGGCTTCTCATAACCAGGAACATATAATAGCCGAGCGGAAGCGCCGCGAAAAGCTCAGCCAGAGATTCATTGCACTTTCTGCAATTGTTCCTGGCCTCAAGAAGGTAACGTTTTTTTTTCGCGAGTTTTTAATAATCCTTCTTTTCTTTTTTTTTGTTTTCCAAGAAGGTTAATGAATCTTCTTCTTGTGGCAGATGGATAAGGCTTCTGTTCTTGGGGATGCCATCAAGTATTTGAAGCAGCTGCAGGAGAAAGTAGACACCCTTGAGGAGCAGGCTGCGAAGAGAACGGTCGAATCCGCCGTTCTCGTGAAGAAGTCGCGGTACTCCCCCGACGACGATAGTTCGTCGTGCGACGAGAGCTCCGGCGATCACCAACACGGCCAATCACTGCCCGAGATAGAGGCGAAGATATCGGAGAAAGCCGTGCTAATCAAGATCCATTGCGAGAACCGCAAGGGAGTGCTGGTGAAAGCGCTTTCGGAGATCGAGAGGCTGCATCTTTCGATCGTCAACACGAGCGTCGTTCCCTTCGCTAACTCGTCGCTCGATATCACCGTCATGGCCCAGGCAAGTTAGAAATGGACATTCAAATGCACTTCTTAGTGCCCCATAGTATTTGTATGCATGGCATTTAAATTCCTTTATGTTTCGATGTTATGCAGGTTGAAGAGGAGTTCTCTTTGACTGTGAAGGATCTTGTGAAGAAGCTGAACATGGCTTTCAAGCAATTCATGTGAAGGAGCTTCAAACAAAACAAGCATAGCTACCTCTGATCCCTACTCTTTTTTTGCATGGTGGAGATAATTGACCATGCTATATACATGGGTTTTTCTTCTTTTTCTCTCTCTCATCTCTCCAATTTTCTTGTTTTTGCATGGTTGAGATTATTAACTAACCATGTTCATTGGGGAGGTTGGGTGATAAAGCTAGAATAATCATTTCTCCTTTTTTTTTTTTTGGTTTCTTTTTTTGAGGGCTTATCAAGGGGAGTTTGACTTTTCATATTCCTTTTCCTTCCCCCCACCCAACCCTATGTTGCCCTTTTTCCTTGGAGGTGTTTTGAGTTTTTTCAAACCTACCACTTTGCCTTCATTTCAAACAATCACTCTTCCCAAAAGCTTGACTGTGGGAAAAAATGGTGCTCCTTTTCACACTGAGGAATCAACAGGCCTTGTTGTAGAAGTTGAAGAGGGGGTCCTTTTTGAGGGTCCTACACATAAAAATCCTCCTCTGTTATGGCTCTTTAATTCTCTTTTCTTGTTATTGTTCTTGTTGTATCCTAGTGGAAAGATATCTCAAAAGGAAAAAAAAAN

>Aco000605; *Ac*bHLH81
ATGTCGGCCACCAACAGCGGCGGTGATATCCCGGTGCCGGCCGCCTACTTCTCGATGCCTCTCGAGACATCTCCCGCCACCGACCACCCGGATATCTCCGACCGAGCCCTCACCGCGCTGCAGAGCCACCGCGAGGCCGAGCGGCGCCGGAGGGAGCGCATCAAGTCGCACCTCGACCGCCTGCGAACCGTCCTCGCCTGCGACCCGAAGGTTACATAACTCGCTAGGTTGAGAAAATGTTCGTGCTGATTCTTTCTAGCTGCGTTTTGGAATTTATAAATCTTTTCATGAGCGTGCAGGTCGACAAAGCTTCTTTGCTGGCGAAGGCAGTGGAGCGAGTGAGAGAGCTGAAGACTCGCACCGCCGACATCCCTGGCTCGGCCCGTCTGTTGATGCCCACCGAGACCGACGAGGTTATCGTCCTCCCCACCGCCACCGCCAACCCCACCGCCTCCGTGTTTGAGGCTTCTGTGTGCTGCAATGACCGATCCGATCTCTATTCGGAGCTCGTCGCGACGCTCCGGTCGCTCCGGCTGAAAACTCTTCGGGCGGAGATCGCGACTGTCGGCGGCAGGGTCCGTAGCGTGCTTGTTCTCTCCAGAGAGGCCGGCGATGACGAAGAAGAAGAAGAAGAGAATAGACATGTTGATGGAGAGGAGGAGGAGGAGGAGGAGGAGGAGGATCGCGACGTCGAATGTGGCAAAGGTGGCGGTGGCGACTTCTTGAGAGATGCTCTAAGGGGTCTAGTGCACCGACCGGCGCCCGCGGCCGACCGGTCGAAACGGCGGAGAGTGGTGGACAGGAGATCGACATGA

>Aco010917; *Ac*bHLH82
CTCGTAGAACTAGAATCTACACAATATAGGCACAGCTCACGCCTACAATTCCTTACTAGTTACAATATGAGACAGCCCACATTCAAATCAAGGAATGCTCACACCCCCCTCTTATCCCCTCCGTATCCATCTTGCACTATTCTTAAAGAATCATCCTTCAAATTAAGATCTCTCCCCATCCCCACATTCATGGGAGATCATCACTTTGTGGACAATCTCAACAGTACCACCACTCTTAAGCCCTCTCCCCACTTCATGGAAATGGATCCGTCGTGCATGGGTTTGGTGATCACCGATCACCTCGCGGAGCTCCACGGCAACGGCGCCGGCGCAGCATCGATGGCTTTCTGCGACGAGAGTTACGCTTACTTCCCTCACCATCACGATCAGTTTCCGCTGCAGCTGCAGCAGCAGCAGCAGCCGCCTTTCGCCGATCAAAACTCGTCACCGGGCCTGCTGCCGATGCTCGGAGACCTCGCCCCGATAACCCTGCCGCCGCCGCCGCCTGCTGCTGCTGCTTTTGTCGGAGTCAGGAAAAGAAAGGCGGCGGCGGCGGCGGATGCTGCGGCGGAGACAAGCTCTGCGAATTCTTCGTCGCAGCCTATGGAGAATAGCTTAAGAGATCATCAATACAGGGCTATAAAGAAGATCGTATTGCTCAGTGTCTCTCATGCCTTTTTGTTTGTTTTTTTTTTTTTTTCTTTTTTTTTCTGCTGTTGTTGTTGTTGTTACTGCTGCTTTGCATTCCGTGCGTGGGGCGTACCACGTCACTCCATGCTGTGACTCCATGCACCATATCTCGTGGTCAGCTCTTCATTCCATCTCATACTTTTAAAAACAACTGATCGATAGGGAGCGACGCGTTGCATGGGGACGACGTAGTGCGGCATTCGCATGCGCATGCAGCATTATTTTCTTAATTTATTCATGTTGTGAATTAATAATTAATTTGATGTTTGTTTTAAGTAAATGGGAGGGCTATTTTCTTTTTTTAAAGAAAAAAAAAAATCTCAGTGCTCAGATAGTACTGCTGCTGGGAAGAGAGGGAGAGGCAATTCAAAATCAAAAAAGGAGGTGGAGAAGCCGAAGGAGGTGGTTCATGTGAGGGCAAGGAGAGGCCATGCCACTGACAGCCACAGTCTAGCTGAAAGGGTAAGATTGTCTTTTCACAGCAGAGCAGCTTCTGGCACAACCCACTCATCATGAGACTGCTGAATGGGCCCACTGATTTTGTGCCTGATCTCACCGCATTCAATGTATCGGAAGAAAATAGTAATGTTATGTTCTGGAAAAGCATGTATAAGAAGAACTTCATTATAAGAATCCAGTTTTCTCATTGGTTGAATGTGCCATCAAGGACCAAAAGTGCAGTTGATGTCATTTTCAGTCACTATGGTGAAAGAATTTTTGTAAAACTCTGAGAACATTTTCACTGTCGCAGATATTTCAATCGACCATAAAAAAGTATTAAAAAAAAAATTTTTTTTTTTTTTTTTGAGTGCTTGTAACTTAAGTTTATCATATTATTCTTAATTAGAAGAATCAGATAGCGGCGCCCTCAATGACGTGTAAGTTCGTATAAATTTTATATTGGAGTCGTATTTGTCATGCTCTTTAATATTGCTTTTTGTAAACGAATGTTCTTGTAAATAGAAAAAATTCTCTTTGTTGTATATGTTTCAGGTGAGAAGGGAGAAAATTAATGAAAGAATGAGATGCTTACAAGAACTAGTTCCTGGTTGTTACAAGGTACCAAACATACTAAAACAGAATTAGATTATAGAGACATATTTTTAGAACACTTTTATGTAAATTTTTTATTTATATCAAAATTTTTTTAAAAAAATTACTAAGGCCTAAATATAAAGTTCAATCAAATCAAAAACAAAAAAATTTCTCTGCTCAACCAATACCATCCGGTCCATCTCCCCTTCTCCTCTTCCGCCACCGCAACCGACGAGGTAGGGTTCCGGCAGCTGGAGTTCGGCTGCGAGGATGTCGTAAGGATCGTAGAGGATTACAATTGCCACGTGTCCTTTCGCCACAGATCGCTAGAGGGAGTGGGTGCCAAACATCGAGACGGCCTCACATATAGCGACCCCATATATAGAAACACTTTTAAAAAATATCGGTAATTTAATCAACAACACTTTTTTTGACATGTATCAGCATTTAAAAGTATCGCTATACACAGTTTTTGTTGTAGTGACAACAGTAAAAGCCCTCTAACCTATACTTTAGCCCCAGTAAAATTGAATTTTGCCCGTTCTTTGGTTAAAAAAAAACTTATGTAAAATTATAGAATTTATCTTCTTAATTAATTAACTATATTTGCAAATTAATCTACAGGCAATGGGCATGGCGGGAATGCTCGACGAGATTATAAACTACGTGCAATCGCTACAAAACCAAGTTGAGGTGATTCATGATCTCTCAATCGATTCGATCGCTTGTTAAGCAAAATGCCATATTAATCTCATATTTAGTTGAAGTTGTCTGATCATCATTAAATAACTTAATTTTCTTTGATCATATAGTTTCTTTCGATGAAGCTTTCGGCGGCGAGCTCATTCTATGACTTCAATGTGGATATTGAACCCATGACAACACTACAGGTGATAAATAAATATGCATACCTTTCGAATGTGACTTCAAATTTTTCATCAACAATTAATCTCTTCATGTGCAGTATTTAATAGATATGGATCACGATAGCTCTTCAATATATTGCCCAATAAATAGGAATTATAATTAGAGGAGTGTTCTAATTTAGGAAATGTAGGCAAAAACATGTCCATTTAGGAAATGTGAAAGTGGAGAGCAAGATTCTAGTAGCCTTATCAAAACTAAAGCCTATATAAATAACTATGGACTCTCAACAGATGACTCACAATGGTTCGTGCTAGCTTTGAAGATGGTATATTGTTTAATTAATGTAAGTTAGAAATATCTTTCTTCTGTTTTTCTGGGTAAAAATGTATAGAACTTAAATAAACTATAGACTATTATTTAACTATGTAACTTTTCAAAAAATTGATTTTAGTACCAAACCTTCCAATTTGCTATTTTAAGTCATCTGTCAACAATTCTGGTGCAGAATTTAAGTTGCTGTTTACTTTTAACTAATTTTATAGTTACATAAATTGCGTAAATATACTAATTACATCAGCAAAGATAAATAATAATTCAAAATTTGAAGTTAAAAAGTCATTCAGTAGTTCAAATTAAATAAATTAAAAAGTATAGGTATAAAAAATCAAAATTTTTAAAGGTTATATAGTCAGATCAAAATGGGTATAGTTGAGGTAAATTCTATATTACCTTTTTTGGGGGGGGGGACATTGTTTGCTTCCTCTTTATGTTTTTTAGACTTGTTACTTTAATTTTACTTTACTCTTGATTACAAATAATATCACCCATAAGGATGTATATATATATATATATATATATATATATATATATATATATATATATATATATATATATATATATATATATATAGTGCATAAAGAGTTTTTATTCACACAAGAATCTGAATATAGAAAAATTAAAAACGCCTGACTTGATGCAATTAATAGATTTAGAAAGGCAATAAAAAAACAAAATGAATAATTGAGGTAAAAATTTACTACTAATTAATCTCAATTTCGTCCCCATCTAAAGTTTCTAATAGGAGAAATCACTCCATGATATCTGTTTTACATGACAATTAATTCCATTACTTAATCTATGGTTCAAAAAATTGTCACATTTTTAGCACCAATATTATTATTATTTATTTCGTAGCTTAAAATTGAAACTCTAAGATGAAATTTTTATCCAAGATCAAACTTCATACAAAATTAATTATATTGAGGCCATACTTCAGTATTAGAATTAAAACTTTCATAAGGATTTGATGATTGATACGTGAGGCCCCAACATTGGAAAAAAAAAAAAAACATGCTCTTAATTTATAACTATTATTTAATTGTATTATCTAGTGTGCAAGATAGTGATATATATACTTAAATATATAAATGTGCAGATGATGAGTATTAATGCAAATGAGGCTCAAGTAATGGAGAAGGTGAGGGAGTTAGGAGGATATGGTGGTGGTGGATGCCCTAGCTTTTTTGAGTAGTACTCTAGTATAGGCCCTTTTTTTTTCTTTTTTTTTTTTTCTGGTGGATTTTGGCTTTTTGGCGTTGGCTTTGGGCTCAATGTTTTGCTTCTGTGGTCGAACACATTTCCATGCACATCACACGTGCATTTATCAAAGCCATGTACTCACCCAAGCACATGTGTGGGTCTACACAATTATATATATGAACAAATGTATCTTTGCTCTCTTATCTCTCTT

>Aco012482; *Ac*bHLH83
ATGGATACATTTGGAGACCACCGAAACAACACTAACCTTAACCAATCGATCAGTGAGAAGAGGAGAGGAGTTGCGGGAGGGGGCAGTAGAGTGGGCTCTTCTCGGACGCGCGTGAAGCTGTCGACGGACCCGCAGAGCGTGGCGGCGCGGGCGCGGCGGCACCGCATCAGCGACCGGTTCCGGACCCTCCGGAGCCTCGTCCCCGGCGGCACCAAGTTGGACACCGTGTCCATGCTGGATGAGGCCATCCACTACGTCAAGTTCCTCGAGGCGCAACTGTGGCTCCTCCGCCAAGTCGAGCTATCCCTTCCAGCAGATTTTGCCGCCGCGCAGATGAGTGGCGATTTCGATAACAATCATGAGTTTGAGATGACGCAGCAGCTGTTCTGCGATTCGGTGCAACTGCAGCCGCCGCCGCCGTCGCCGTCGCCGTCGCCGTCGCTCCCGCCTTTGCCGACTTGCTTTATACAAGGAGAGGAGGAGATGATCTTGGATCTTATGTATAGCTAA

>Aco013643; *Ac*bHLH84
TCTATCTATCCATCTATTGAGTGCTGTTGAGAAAGAGACAGGAGAGGGTGTTATGCATTCACTGTGAGAATCTTGCAAGGAAGTCATTGTAGAATAAGCAGTCCGGACCTTATCTTTTAATCCCAGGATATTGCAGTCAGGACAGGAGGATCAGCAAATCCTAGCCTTGCTGGTAATCCCTCCACCAATCATTCCTGCTAATATCCCCAAGACATTATTATTAATATTCTTATTATTATTCTGGGTGGTCATAAAAGGGTGGGAGTAGTGATATTTTCCTCTTTTCATTTCCTTCTCCTTTTTTTTTTCTTTTTTTTTTTTTTCTTTCTTTCTTTTTGTGATTGAAGTTTAGAGGAGTATAGTTTGATTATCTCTTTGTTTGTTTTGGGTGAATTTGGTTTGGGGGATAAAAATGGGATTTTGTGAGAGAGGGTTTTAACTTTAACTTTTAAGTGCTGCAGGGAAAAAAAAAGAAAGAAGAAGAAGCTTCTAGAGTTTCAGAATCTCCTGGTGGCTCTCTTTTAGGCTGATTTAGTGGTGGGTTTGGGAGAAAGATGAGATCTTGTGAGCATCTGCTCTGATCAATCTGCACTGCTCTGCAGTAAAAGTTGGATCTTGGAAGGAAAAGAGTTGTCAAATTATTTATTGAGGGAAGCATTTGATTGCCAAACTCAGGAAGGGAGATTTTGCATTGCAATCTTCTGGTGGTGGTTTTGTATCATCAAGCTAGGGTTTTTTTTTTTTTTTTTTTTTTTTTTTTTTTTTTTTTTTTTATGAATTGGGGGTAGAAACCACCATGCCCCTCTCTGAATTCCTGCTCAAGACCACGAAATCGAAGCCCGGAACCGCGCAATCCAAGATGCCCATCCCCAATTCCTCCTCATCTGATCCCTTTTCTATGTAAGTTTTGGCCTCTGATCTCCCTGCATTCACTTGTTCTTTGTGTCACCCTATTTCCCTGTTGATTGATTAATTAATCTGTTGTGTTGAATGAATATCTCTGATCTGTGGGATTGTAATTGTAATTGTAATTGTAATTGTAATTGTAATGAAGATCTGATCACGAATTCGCGGAGCTTCTGTGGGAGAATGGCCAGATTGTGTTGCATGGTCATGCCAGTAGATCCAGCAAGAAGAGCTGGGCGGCGGCATCCGCCGCCGACCACCCGGAGAAAGCTCCGGCGAAGGACAATGTGGATTCCGTCGACGCGAAGAACGAGCAATTGGGCGCCGACCTTTCGGTTCATCAAGAGGATGACATGGTCCCGTGGATCAATTTCCGGATCGACGATGAGTCTCTCGATGCCGATCCCGTGCACAACGATTTCTACTCGGAGTTCCTGGAAGACATCGGCGTCCTAGGGCAGGAAGCTAAGAGCCCGCCGAACGCACCGCAGGAGATTCCTTCGAAAGGAGCTCCGGCGGCTTCCGAGCGTGGCAGGGGTAATAAGCCCAACCAGTTGATTCAGCTCTCTCAAAATTGTCAAAGCGCGGTTACGAACAATAACTCGAGAGTTTCGGAACTCGGTAATGGCGGTGGTAAAGCCCAGATGGGCTATTGCATGGAATTAGTGAAAAACACAAGGTTGGCGCCGAGACCGAACACGGGCGTGAACTTTGTCAACTTCCCCCTCTTCTCGAGGCCCCACGCTGTTAAGATCAACGATCGAGCGAGAGTAGAGGAGAAGGCAGCTGCTGCTCCAGTGAATAGAAATCTCGTCGAGTCTAGTAGTGGAGTTAAGAGTGTCGCCGGAATTAAAGCCCTGCCGCGTAAGTTGGAGCTGGAAAAACCCGATGCGCCCGGACGTCAACATGTTAAAGTCCCTAGCGAGCGAATTGAGGCTAATCGCGAAAGAGGTGAAGATCATGAGACCGAAAAGGACGATAAGAATGCTGCTGCTATTAAAGATTGTAGCTTCGCAAAGCCAGAGACGGAGAAGGATCCAGTAGCAGTGGTTGCTTCCTCTTCCGTGTGTTCCGTTAATTCCGCAGGAATTGAATCGTATGAACCCAAAAATAATAGAGAGAAGAGGAAAGTTAGCGAACGCGAAGAATCCAGCGGCGAGCAGAGCGATGTAAGTGTTTGACAAAAAGAGTGCGAACGATCGGCTAGTTCCCTCAATGTTTGTCAGTAAAGGAGCTCTTTTAACTAATCCATCCTTTTTTGTATGCGGTAAGGATCTTGAAGATGAATCGGTGGGAACAAAGAAGCCCGCGAATGGGCGAGGCACGGGTGCAAAAAGAAGTCGAGCCGCAGAAGTACATAATTTGTCTGAAAGAGTGAGTCACTAGTTAACTAATAATGTACTCAATTCTTTTATGTGTTTCTTTGTTTTCTAATTTAGATGTAATGTATTTCTATGCGGTTTGTAATAACAGCGAAGGAGGGACCGGATAAATGAGAAAATGCGGGCGCTACAAGAACTGATACCTAACTGTAATAAGGTATCATTTTTTTTTTTTTAATTGCCCGCTCTCTGCCGTTCTAGTTTATCAGATGATTCAGGAACCACCTAATTTTGATTTTTCTTTTCTTTTTTTTTTTTTGTTCATACTTGCTTAAAGACGGATAAAGCTTCAATGCTCGAAGAGGCCATCGAGTATCTTAAGACACTTCAACTACAACTTCAGGTAAAGCTTAATTTTTTCGCTATGTTCTAGTGGGCTCTTTTGCGTGGTTAGAGCTTTTCTAGTGAAGTACGGTTTGAGTAGCGCTTTTCAAAGAAGCTCTGGTATCTTTTTCATCAAAATATCCTTTAAAGGTACTCATATTGTTGGAGCTAAAACCGAAATTTCAAATTTGAGCTTCTACTTCTGGCATCCAAAAGCTCATATTAGTTTTCTGCAAAAGCAAAAGCTTTGGATTACTTCGTAGTCAAATACTTGGTTTGTAGAAGCTCATGCTTTTTTGGAGTAAAGAAATTGTTTAAGAAGCCAGGCAATAGGCATGAAAGACCAATTTGGCCCTGCGATAGGGTGCAGTCACTCTGTTTGACTAGTGGTACTACAAGAAGCCCTATAATAAGACCTCTCAATTGCTTCCTTCAACAGCAGCGGTAGAAGGTCCAAATTGAATATTCTACTTTTGGCCTCATAATCTTATTTCAACTTTCCATACTAGCTAAAAGTTCGAGGCTTTTCTAAAGTTCGAGGCTTTTCTCGGCACAAGACGCACACTAAATGCGATTCACTGTCTCAAATTCCGTAACTTCTCTTATGTTCTCTCTGGCAGATGATGTCCATGAGGAATGGGATGTACATGCCAACTCCTATGATGTTGCCGCCAGGGATGCAACACATAAACGGACCGGCGATGGCCCATTTCTCCCCGATGGGTGTAGGGATGGGCATGGGCATGGGCATGGGCATGGGGATGGGGTACGGCATGCACGTGAATGGATCACCAGGTTGTTCTTTAATTCCGGTGCAGCCCATGCATGGGCAGCAGTTCCCGTGTCCTGCAATCGCAGGCCAGGCTGCCTTGCACCGGATGATGCTCGGACCAGCTAATCCTCCAGTCTTTGGGGCAATTGCGCCACACCAACTTAGTTCGCCACCATGTCATGTTGTCAAGGGAAATACAGCGCCTGATGTTACCATGCCAGCGGCTGCTCCAGCCCCGCCAACCTCAGATCCGGCGGCGGCTCCAGTTTCTAGAGATCAGAAGGAAGAAGGCAAAAACTTATAAATATTGTAGAGATCAAGAATCAATCGTGCGGGGAAGATGGGTAAGTCTTGTGAGATTTATATATTGATTCTCGATCCATATCATTATCTTCACTTTGTTTGATACCAAATCAAGATTGATTTGTAGTTTAAGGAACTCAAATGGGTCGAATATGGGGAGCTAGATTTTCTTTTGCTCGGAAGGAGATATGATTCATCGAATTATATTATTTACTTCTCCTATCTTACCTGTCTAGTAGTATTTAGATTTCATGAGTAGAGGTTTATTTTGGATAGAGAAGTTTTCTTATGTTTGAGAATAGTATATTACCACAATTCAGGCCTTATATCTACCAATTTGCTTACAGTTATCAAAGGCCGCCAATTTGTTAAATTCAGTTCAAATCATGGATTAATGAGATTCGAAACAACGAAATTAAGAAACTACATTGCAATTCCATCCAAATCGGCAAATTCGGGTGTTGAAGTTTTCACCATTCAGTAGTGTAATTACTTAGAATTGATGTTAATTACCTGCTACACTTATTGTTGGTGTTATCTGTTCTTGTTGTAGGTAGCAATAAGGAAGGATTAACAGCTAAAGAGTATCTGATGTACATTACTAATCGAATAGCGACAACTCAATCTGTGGGGATCCCTCAACCTTGCAAGTGTAAATGGGACAAGACATCGCAACAGGTGAGTTCGAAACTTTTTCAGTGAGCTGTAAATCACTTAATTAAATTTATATTCTTCACATAATTCGAGTCTTTTACGTTCTATCCGCAATCTTGCAGGCTACTGTTGATGGGTTCCATGCAATGGGGGTTGCTCAAAATGTTCCATAAATAACAACTTATGTATGTGATCTAACATGTGAAGGATTCTCTCTCTATAAGAGTACACCTCAAGTGCCAATTTTGCTTTTTCCCAAGTGGAATTGTGGGACAATGTGTAGTATATTAGCATGTTAAGAGTAGCTTAAGAGTTAAAAAAAACTTTGTTTTGCATCTATAAGTCGCGGATCCGTGCTGTTATTATATAAGGAGCATGACCTTCAACTGTGTGTACTAAAAATGGTTTGATATGAATAAAAATTTTCTTAAATGGATTTCACAAGAATTTCAATTAACTC

>Aco014156; *Ac*bHLH85
CAACAACAGGGGAGCTCCGCCAAAAAAATGATTTAAAATTGATCGCAAACCAATAGAATAAGAAATGCCAACATGTTACTGTTTAAGCACTATTAATGCGTAAAAAATATCCCTCCCCCTCCCCCACCCGAATAAAAAAGTCTCCTCTTTTAGGATAAAAGTAAAAATCTAAATGCTAAGAACCGTCCCAATCAAGGCTTGTGTTCCCAGCACGATCCCATCCCTATCCCCGCGTTGCCTTTTTCCCTGTCCCCTCCCCAAATCTTCTACTTTTGAGTCCCAAATCCTAGTTTCCCTTTCAAAATAGGATTTGTTTTTAGGGATTTTGAGGCCTTAATTTCGATTTGGATTGGTTGTTTGATCCGGGAGTGTTCAATTTTTTTTGGGGGCGTTTTAGAAGATGAGCGGGGGTCAAAGCAACGGCGGGGGATGGCTTATAGATTATGGGCTCGTTGATGAGATCCAAGGCTCGGATTTTATCTGGGGTTCTCAGATAAACGATGATCCCATGGTCTCAAGGTGATTTATAATATCTTACATCAATTTAATGTTGTAATTTTTTTTGTTTTCTGTTCGATATGTTGACCTGATCAGTGAGATCAGATCACAATGATCTCAAAAAATTCTCTTTTTTTGTTTTTAAAGTATTTGAGCAGTTTCAGATCCCTATAGTTTAGATCGGTAAGCAAAAAATTCATCTTGAGTTGTTAATATTTAGATTGATCCACATAAGGTTGAGAACTAGAGAGATCAGATCAACAACTTTTGTCTATGTTAATATTGAGATGAAATAGAAACATCATATGCGATGATTCTCTCACTAACTAAAATTTCTGTACCTTTAACTCTCTCATTTGCAGTGTGATGTTTGGGTTCGACGTTTCGCACAAGCAGGAAGGTTGTGCAGATAATTCTTGTCCTAAGAAAAGGTAATTGACTGATTTATAACATCTCCAACATGCTCTATATTTCTATTTGAATTGGTGAATGTCACCGTATAACTCGAGCTCAGGGTATGATGAAAATAATGTAGATTGCTGACTCATAGACATCTTTACGTCACTGTTTCAACTTTCAAATAATAACAGATGTAGAGCATTCCTCAAATGATATAAATCAGTGAATTGAAAGGCCCCTGAAGGGTCGGATATGAATTTGAGGGAATTTTGCCTGGATCTAGGTGAAGCAAGCACAAACGAATATTGCTTATGGGGTTCTGGCAAAGAAAAAGAATGCCAAACTTCGAAATTCAGAATAAGTATTATTGGAAGACATAAAAAAATTATTTACTATTCGTTTGCGGAAACAGAATCTCTGGATTAGTTGGCTCTGTTATTATTCTTTTTCATGAAAGAGAAGAAGATATAGATTGGAGTTAATTAATGAGGTTGTCGATATCGCCAAGTTTAGTGGTAATTATTTGTCACTTGCTACATCAATAAAGATCCAGGCTTCATTTCGTTGGTGAAGAACCAAACTTGGCAGGTGTTACTCAGAAAGTATACGCATTTTTCAGACTTCTGATGCTATTTATCAAACAAAATTTGTTAGGAGAGCTTCCTTTGTTTAATCACCAAAACTAATACTTTCTTTCGATTTGCTTTTAACAGAACTCGCCCTGAGTCATGTGCTGCACCAGGGACTAAGGCTTGCCGTGAAAAACTGCGAAGGGATAGACTTAATGATAGGTAGATTCTAGCATTCTATATCATGCTTTTTCTGGTGATGCTCTGATTAGAACATATGGAAGCAAAACTTGTTTGAGTACATCATGCCTGTTTGGCATAGTTGGTACTTCCATAGAAGCATTGTTTGAGTGGAGTTACGTGAAGAAGCACTATTTGCTTGTCCATTAAAATTTCTTCAGTATCATCTAGCTAAAGCAAGAGTTTTTTTATTTTTATTTTTTTAAGGCAGAGGAAAAGCCTTACCCATCTTCATTCTTAAGTAATGAACAAAAGCATACAGGGAGGCAACTGGGCCTCATGAAACCCACACACAAGTGCACGCGCACATGCATGCACACATCGTGTGTACACCCACTCACTCACAGACACACACACTCCCCCACACACTCACAGAGACACAGAGACTGGCGCACACACGCCCACATGCATGCGCAGGCATGCACAGACCCAACCTCCCTCCCTCCCGCGCTAAAGCAAAAGTTAAAGTTTCAAACGAGCATCACCTTTTCGCCACTGTGAGAGTTTCAGATGTTTTATTAGCCAAAATGCCACCACATAACTCTTGGTTTGCGAACGCTTCAGCTTTCTTGAATAGGGATGCTGTTCAAGAGGCAACACAAACGAGCACTTAGAAATTACTGGCAGTTACTAGGATCTGTGTATCTCACAGCTAATCATTTGTAAGTTCTGTTTTCTTTCCATCTCAAGAGAAGCTTTTTTTTTTTTTCCAGGTTTACAGAGTTGTGCTCTATTTTGGATCCTGGGAAGCCTCCAAAGGCTGACAAAGTTGCCATTCTAAGTGATGCAACTCGTCTTTTGAACCAGCTACACCTTGAAGCTCAGAAACTGAAGAAATCGAATGAAGCACTTCAAGATAGCATAAAAAATCTCAAGGTAAAACTGATATGACTAAGTTGTGCGCCTTGACTGAATGGCGTTTTCTTATAATGGCTTGGCTGAGTTCCAAGTTTTTTAGTACCAAAGGAAAAGAAGACCCATAGTCCTCATTTTTTAGCTCAGCAGTTGCAAATAACCAATGAATGATTCGGTCGATCCACCTTTTGAGCCTCTCTTCTCTTTGAAATATTCAATCCATCTATCCTGCACGTGTGGATAGTTCTTTTTCTTTTCCCTTTTGGATGTAGTGCATTATGTATATGTCAAATAAAGAACACTAAACTTGCTAAGAAATAACATAGAATGTAATAATATTCTTTGTGAATGAAAGATGCCATATCCCATCTGCTATTGATGACACCTTAAATGGAAAGCATTATCGCTAAATAGTGGGTCTTCTTTTTTTGCAGCTCTGAACAGAAAAAGGAGATTTTAATAGTTGATCTTTGCTCATACTTTGTACACTATTTGGTATGAACTACAGGCGGAGAAATCAGAACTAAGGGACGAGAAGATGAGGCTCAAGGCCGAAAAGGATCGATTGGAGCAAATGCTGAAGGGAGTTAGCGCCGCTCCCCAATTCATCCCTTATCCTACTGCTCCTGTCGTCGCATACAACAAGGCCGTCCCGTATCCAAACTACCCACCGGCAGGCGTATGGCAATGGGTACCTCCGGCCGTTCTGGATACTTCGAAAGATTCAGTGCTCTGGCCTCCTGTTGCATAGTCTTCTTCTATTTCTCTCTTACTTTGCCATTACAAGGACCAAAATAGTCCTTGGTGGTTGTTAGTGACCCATTTATGTTTCACTAAGCTGTTTTCTACTGTATTATTAACCATGGAGCTGTATCGAAGGCCCGACAAAGCTCCCAGGTCTCATGGAAAAATTTAGCAGTCGTATTGGTACATATATCGTGTAATCGCAATGTAAGCTGTATTTGGTGGCATGTCGTGTGCTGCTTAATTCGCCAGAGTGATTAATCTCACTAATGTCTGAATGATTAAAATAGTAGCCCTGTGTTACACATTTCATTGCCTGTATATGTAGTATTGATAGCATTGAACGATAAGTGGATGCTGAT

>Aco014880; *Ac*bHLH86
TCAGCAACTATAACCGTGCCCCCTATCAAAATCCGAGCCGAAAAATTTCCCCCAAAATTCCAATAAACCCTAGAAATTAAAAGAGGGGAAAAAAAGTTCTTACCTTTATCGTGCTTCTCCCCACCGATTCCCCCGGAAATCGACGATTCCGGGGAAGAATCAGAGATGGGTTCCCCCGAGAACCCTAATTGGTACCTGGATTGCGCCCTGATCGACGAATTGCCCGTCGCCGATGGGGGATTGTGTTGGAGCGCTCAAGGGTTCAATTCCTCTTCCAATGTAAGGTAATTGGGATCGAATGTTTAGAGATTTAGAGCCATTTGATGTTGGGGGGTCGATGTTGTTGTTTGGGTTTCCTTTGGATTGGGTTATCTCATGTAGTTTTTGTAATTCTCTTCTTTTTGATGAGATACAGATATGGAAAATCCATTATGGGTATAATTTGCTTTATTAGTTCTTGCATACTTTGATTGTATTGCTTCATTTCAGTTAATTCGATAGAGATTGCTTATTTTAGTGAAAAATACGATCTTTTTGTGATTATAAACCAAGTACACTTGAGTTCAAGTATGGTTGTGGAATGAGATGTGAGAATTTTGGTGATATTATGTGGTTGGAACATATCTTTGATGCAATCACCTAAGTCTAGGTTCGGTTTGGCTGCACGAACTTCTTTTTTTTTTTTTTTGAGAGAGAGAAAGGTAGCATTGTACCCGTTCCATTTATTTTTTAAAAAAAATAAACTTAGCTGAAAATATGAAGCAACTATGTTTCGAATTTGGAACGTCAGGTACCAACCACCAAGTTCTTTCTAACTTGCGCTAGGGACGGTTAGTGCTGCACGAACATTTTGTTTCGCACATTTCCTTGTTTAGTCAAGGGAATTACAATGGAATAGTACATGCAGCCTTTACATCATTTTCAGTGAATAAGGTCAATCACCTTGCAGGTGTAATGATATATCCGAGAAAACAATGGCTTCCAAAATCTTGTATTTTAATACCATGCTAGTTTTTGAGAGGATATTGTAGCTTACATTTTCCACCTGTCCCTGTTAGAACTTACAAGAGTGGTTTCTCTATGTGAAGTGCCTCTCTTTGTTCTTTTCGCTGTTCTCCTTCTTTTCTTCATTCTTCAGTATTATAGGTTTCTAAGCTTTTATATTTTCTGCCAGCTTGGAAACTGATGGCCCTTGTGTAAATTCTGGTGGTTCCAATGAATCGGGTTCTCGGAAACGGTTTGACTCACTTAACATACACTTCTGTATTTAATATCCAATATATTCTAAGATGCATGGCTCTCTTCTTTTGCTAATTTCGTAATAAGAAAGCCATTATGTTTGGCAGTATAAGGTCAGAGTCCTGCCGCAGGCCTGCTTCAAAAGCATGTAGGGAGAAAATGAGAAGGGATAAGTTGAATGAGAGGTAAAATAACTAAATTTACATCATGATGTTGTCCATGTATAAATACTGAAGACCATGGTCTGGGATGAGATGTTTTTCTCACTTATATTTTTATTGCAATTGAGGTTCCTGGAGTTGGGTTCTGTTCTGGATCCTGGAAAGCCACTAAAGGTCAACAAAGAAGCTATCTTAAGTGATGCCACTCGTATGGTTATTCAGCTGCGGGACGACGCACAAAAGCTAAAAGATTCAAATGAGAGTCTCCAAGCAAAAATAGAGGAGTTGAAGGTCAGAATATCTTTTGAATTTCTGCTGCTTAACTGTTTTATTTGGCATTGATGTTTTGAATGATCAGAAATTTGGGTTATTTGAATGGATCATTTGCCTACATATCGGCAACTTGCTAGAGAACTATGAAAAAGTTTGTGAAATAACTAATGAGAGCCTTTTACTTTGCTATGCTCGTCTGCAGCCGATGGGCTAAATTACAATTTCAATTATTGTTTTACAAGTTTATTTTTCTATTTATGTGAATTTTCTATTTGCCTTAAAAAAGTAATTTCTTGCATATGTGCCCTTGTGCTTGCAAATGTGTACCTCTTTCAAAACTTTCTAATGAAAACTAACCTCCCTCCAAAAATTGAATGGCTTCCAAACTTAAAGATACGGGAATTTTGTTAAGGAACTGTTTTTTTTTTTTTCTGTTGTATATATTCCTGACTTGATTGATTTTTTTTTATTTTTTTATTTTTTTTTATGTAAATGCTTTCCCTTTTAAGGACAACCTCTTAAAAATACAAGCAAACGGTCTCACTAAATTAATATCATTAGTTAATTCATCTTTGGAATAATTTAGCAGATAATGTAAAGGTTTACTTGTCCTCCTTTTGATGAACTTGTATTCTATTATTTTAGGCGGAGAAAAATGAGCTTCGCGATGAGAAGCAAAGGTTGAAGGCTGAGAAGGACGGACTAGAGCAACAAATAAAGATTTTGAATAGTCGGCCCTCTTTCATACCTCAACCTCCTTTGATACCAACTGCTTTTGCTGCTCGAAATCATTCGGAGGGACAGAAATTGGTGATGCCTATTATTGGCTACCCCGGGTTTCCTATGTGGCAGTTTATGCCTCCTTCTGATGTGGACACTTCACAGGACACCGAAAATTGCTCACCTGTTGCTTGAGATCATTCCTAATCAGCCATCCTGCTCGAGTTGTTCTTCTTCTTTTGTTGCCTGTGTTAAGCTCATTTGCAAGGCAAATCTTTCTTTCATGGTATGGATATTTTAGTTAAAAGGGGATAGCTTTTGAATTAATTGTAAGTTGAATGTAAATAATTCAGCCTCAATTTTTCAATTTTAGTGAACATGGGAGTAACTCTCTCCACCCAGTTTGTTAATGCAGAGTATCCCAAAATTTCTTGCAGTGGTATAATTGTATAATATATACATCTATATGATCTTCATGTAGG

>Aco014894; *Ac*bHLH87
CCTATATATCCACCACTAACTCACAACACGAAACCAAAGAGTGTGTGTGTGTGTGTGTGAAAGGAGAGAGAGAAAAAAGAAAAAGAGGACCATGGGAGAGGATGTCATTTATAGCAGAATGTATAGTGGCCAGGAGGGGTGGTGGAATAGCAATATTCCAAGGACTAGCTGTTTCAACGGTTCTGCCGTGATGTCGGCATCGATATCATGCTCGACGGAGTTCACCGACGTCATGGGCGGCGGCTTCAACTGGGCGGCCTTGGCAGCCGACGTGCCGCTGTCGCAGTCGCAGTCGCAGTCGCAGTCCTCCTCCGGTGAGTCGCCCGGCTCGGCGGTCTCGAACAACTCCTTATCTTCTTTCCAAGACACTTATCATGCGGCCACCGCGCAGATGACGGATCTGGCCGCAGTATCCTCTCCTGCAGTTGATTGGAATCAAAAACCTCTATTGTAAGTAAGCCTCCTAAATTCAGAGTTCTACATACATACATTAAAAAATATATATATATATATTATTTTTGCGGCTCGGTAATTAATTAATTTAATTTTGGGGTGGCTTAATTAGTTATTCTATGATGCATGTCTAATGTAATTCGGGTGACATGACAGGAGTAGGGAATCAGGGTACCATGGCTTGTTTCAAGAAGGCAACAAGAGTAGTAGTTTGAGAGGAGATGATCTCCAGCTGCTTAATCCCAATCCCAATCCCAATCCCAATCCCAATCCCAATCAGAAAAATAACATGTGCGATGAGAACATCTCATCATCAGTCTCATTATCACACAACATCAATCAAGGGCTCCCCCAATACCAACACCACTACGAGTCCTTGTTAAGAAGCCTCCTGGAACCTGAGATATTGGAACCTTATTACACACAGCCGCTCCCCGAGCCCTCACCTCCGAAGCAGCAGCCACTTCAATTCTCTACTCACATTAACACATCCTCCGCGATTCGGCCGGCTACGACTCCGATCAGCAAGGATCTTCGCCTTTCATCGCCCCGTAATGATGTAGTAGTCTCACATCATGCTCTCAAGGAGATCAACTTGAATCGCAGCAACCGTGCCACGAAGGTCAGTAGTGTATATCTATACATATATCTATACATATATCTTCTAATTACATAACTACGTACTGATCATTAATAATTTAATGATTGATTATTTAAGTAATTAAGCCCTTAAATCTTTTGTTTTGGGTCTAAATTTGATGCATAATTTTCAGTCAGTATCGGAAGGAGCTGGCGGATTGAGCCCAGACATCGCTGCGCAGCAGCCTGCTCTCAAGAAGCCTAGAATTGAGACACCGTCGCCGTTGCCGACTTTTAAGGTAAGTAAGTTGGTTTGGATAACTAACCTTTGAGATCCAATCCCTCTTTCATTAGATACACATATATATATATGTGAAACACAATGAAGAGCTTATATACATAACGTAATAACCCCAAGTTTTAATTAACATGCATATCTATCTATCTCCAGGTGAGGAAAGAGAAGATGGGGGACAGAATCACTGCTCTTCAGCAGCTGGTCTCGCCGTTCGGAAAGGTGCGAATGCTTTCGATGCTTTGCTTTAGTTAATTCCACATCTAGCTAGCTCATACGTACTGCATGCCTAAAAGATTATTTTCTTGTCGATCGCTTAAAAGTTTTTCTCTTACCGCAGACCGACACAGCATCAGTACTCACTGAAGCTATAGAATACATCAAGTTTCTTCACGATCAAGTCGGCGTAAGTGTCCCCTTTCTTCCAAGCTTCTTTGTTCAGTTTACAGAAAAACTAGCTACAATAAACGACATTATTATCTAAACATCTTCGTGACTTGATCAGGTCCTTACTGCTCCGTATCTAAACAATGGCAACCAGATGCTGCACCTAAAGGTATCTATATATATATATATATATATATATATATTGGAGATGTACTAACTTTTTTTTTTTTGATTGATTTTTGTGACACAACTGTGAAGATCAGTGTAAAATATTTCTAATTACTGGGTTTGTGTATACTCAGAGCCTGGAGAAATTAAACGGTACACAAGGGCCAAAGAGAGACCTCGGAAGCCGAGGGCTATGTCTGATGCCAGTATCGAGCACGCATGCTGTCGCTAGCGAAATACCTTTCGATTTCTGGGCTCCTACGTTCGGAGGAACCTTTAATTAACAGCGAAGATTATACGGTTTGAAGCGCTGATCTACAAAAAACATGGTGTAAAAATAGCCAAAACTGTTCGTTCAATCTAATCTTTCATAAAGAATGAGAGATTACAGTAGGGTATGTAGTGATATAGGTAAATTTTAATAGGCCTTTTTCAGAGTTAGTGCTTGTTGAGGAGTAGCTATATATGCACTTGTAGTATTAAAATTGCAATAAATAATTTCTGTTGGAGTAGTATAACAGATTAATTTAGAGTGTGGGGTTTGCTAGTTCCAGC

>Aco025362; *Ac*bHLH88
CTAATAAAGAGCTAGTTGTTGTTTGTTGGTGGTAGAAAACTTGGGTGTAAATATGGCTAAGTGCTATGCTTCCACTGATGAAGGGAAACAAGTAGTAGTGAGCTTCCCCTCACTGTACAAGGGCTCTCTCATCCTCCCTCGCCCCACCACCGCCGCCACCACCACCACCACCACCACCACGCCGCGGCCGTTGCTGCGGTCGGGATCTCCGTCGTGCTCGTCGGCGTCGTCTTCGTCATCATCGGTGGCGGCGGCGCGGGCGTCAAAGATCCACAGTGAGGCAGAGAGGAGGAGAAGGGAGAGGATCAACTCCCATCTCTCCACATTGAGATGCATGATCCCTGATGCTCACAAGGTGTGTAAATTTTTTAGCTAAATATATATAAATATAAAACTTAGCTGAATTATGGACAAATTTTGACTAATTTTTTAAAAGTTTGATCTTAATACTTAAATTTTTTAATTTATTTAATTTAAGTAATTCGATGACTTTTCAACTCGTCCAATTTTAATGTCCAACATCTTAATCTATTTAATTTAAGTGATTTGACAACTCTCTGGCTTTTCTCTTCGGATATAAAATTTAGCCTGATTGTTTATTTTAATGAATTTATAGTTTATAAATTGTATAAAATATATTAATTAAATCTGTGAAGATAGAGGACGTTTTTAAATTTTGAAGTTAAAGAATTACTAATTTGCTCAAATTAAATAAATTAGTAAAATTAAAATGTTTAAAGATTAGATAGTAAAATGCTAATTGGTGATAGTTTTGATAAGTTTTATATATTTTTATTTATTTTTTAAGTTTCACAATATCGTAGGTGTTAAATTTAAATAATTAGAACATTATTTCACTTTATTCTCTGCTATTTAATTTAAGTTTGAGTGTTAAAAATTTTAAGACTCGATAATGTTGAATAAAAAATATTAAATTACCGTTGTCTAATAATTTAAGCTTTAATATAAAATACTAAAAACACTAACTCAAATATTATTTAATTTTATCTTTTCTATTTAATTTAAGTTCATATCCTGGGTCTATATATTATTCGAATTTTGAGACCTAATTTCATAGTATTCGAATTTTTCGCACAATAGTTTTGCTCCAAATTAATTTCCAAGAACTGTAACAGATACTGGAATTAGTAAAGGAATGTCTCCTTTTTTTTCTGAATATAATTAATGGTGAATAGGAAACTTCATAAATATGTTTCCTATTGGTGTTTCCACATTTAATAGTTAGGATAATTTAAGAAATTAATGCGAATCAGATGCATCAAAGATTTTCAAAATTTATAATAATTCATTTTCATTTATTGAACTTATATATATATATTATAGATTGTTATTTTAGTTTATATAGGTTTTAATTTTTTAAAAAATATTAATTCTCAAACTTTAAAGGCAATGCATTAGGTGTAAGGATATATACACGTAATGGTGCAGGCAATAAATTCACTAATTTAAGATGTTGCTTAGTTGCTTCCTTAAATTGTTGTAGTGCTGGTATAGTATCACTATATATAGATGCACTAAAAATGATGAAAAGTAAAATAAAATCTTTTTTTCAAGAAGCAAAAGCTAGGTTTTTTTGGTAAACATAAAATTTAGAGCTTTTAGTTACAGTGAAAAGCAAAAATAAGCTTTCGAGCTACCGAGAGCTTCAGCGTGAAGCTACTGAACAGCTTTTACAATTAAAAAGAAAAAATAACTAGTGCTTCTTCTAATAACTACATTGAAATAAACTTTTTGCAAAGCTTTGAACAACGTTCTCTAGCAGCTTAAGCTTTTAAAAAATAACGGTGGTTTCATATTATTTAACATATAGTATCAGAGCAGGAGGTTCTGACACTACAATAAAATATGTTTTAGACGACGGTTTATTCTAGCGTCTTCAATATTTGGCATGCGTCGACACTATAATTAACCGTCGCCAATAAACATATTTTTACAAAATACCAATAAAAATATTCCGACGCTATATTAAAGTGTTGGTATATTTATCCCAATATATTTACTTAATGACTCTTTGCTTGACACTTTTATAAGCATCGGAATTATTTTTGTCGGCGTTTTACAGTGTTTCCCGTCATTATTTAAAGTGTCCTTATGTGCACGATTTATTGTAGTGTGAGTTTGAGGCATATCAGACTCCTAGCATTATTAATTATTTTACGTTTACTTCTTCCACATCATGAGCCTAGCGAAGAGTCTCGAGCTCAAACATAAGAAAGAGTGTTAAATATAAATATATTTGAACGCCGCTCTCTATCAGTTTAAGCTTTTAGAGAAAAATGATTGTTTCATATTATTTAAGAAAACAAGGATCAGGGGCTTTTGCCAATCAAAAATTAATATATAGTGTTCAAAGAGTCCAACATTATTCTAAACTAGAACTGTAATATATATAATCTAAAGTGACTTTAATGTTGTTAAACTGCAGATGGATAAGGCTTCTTTATTAAGCAAAGTGGTTGATCAAGTGAAGGACCTAAAGAGAAAAGCAAATGATACAAGCAAAACGGTAAACGTTCCGCCGGAAATCGACGAAGTAACCGTCGAACGCCACAAGGGTGGCCGAGACCATACTTTCTCCGGCGACGAAACCCTGCGCATGAGGGTTTCGGTCACCTGCGACGATCGGCCCGACCTCTTCGCTAGCCTAATCCAAGCATTCCATAAGCTCAGGCTGAGGACAATAAGGGCAGATATTACTTGTATAGGGGGCAGGGTGCAAAATGTGTTCATACTTTGTGGGACAGGTGAGGGAAGTGAAAACATGTGCTTAGGTTCTTTGAAAGAGTGTTTAAAAGAAGCCCTGGCTAAAGCTGCTTCTCCAGAAGTGCTTTTAAGCAGCAGCAGCAGCAGCAGCAGCAGCAGCAGCTTCTCAAGCAAGAGGCAGAGGCTGTTGGGGTCTCATTAA

>Aco004054; *Ac*bHLH89
AGAGAGAGAGAGAGAGAGAGAGAGAGAGAGAGAGAGATGGCATTAGAAGCCGTGGTGTTCCCACAAGACATATTTGGTTACAATTGTAAGGAATTATACACCAATATGGGAGGAATATGGGGCAATGACTTAGCCAATATGGTGGTGGTGGAGGAGATGGAAATGTCAGAATGTGAGATGGGTGGTTGGGTTGGTGAGAACTGGAACCCTTCATCTTCTTCTCTTGTGCAAAATCTAGATGAGTGGGAGATTAACTCCTCGTCGGCTGAAGTTTGGGGCGTGGCGGTGGAGGCGGAGGGGGAGGAGGCTATGAAGGCTGTGGATGGACGGAGGAAGAGGCGGCGGACGAGGAGTGTTAAGAATATGGAGGAGGTGGAGAGCCAGAGGATGACCCACATTGCTGTCGAGCGCAACCGCCGTCGGCAAATGAACGAGTACCTGGCCATTCTCCGGTCACTTATGCCCCCCTCCTATGTCCAGAGGGTACATATATTCATCTCGTCGTTTTAGTTGTGTCATAAACATTTTTCGAATGATGTAAATACTAAAGCATCCAAGTCCATCAGAGCTTATCTTTCTGTCCTTGGAGTATAGGGTGATCAAGCATCAATTGTCGGTGGCGCGATAAATTACGTGAAGGAGCTCGAGCAACTTCTCCAGTCCCTCGAAGTCCAAAAGAGGCTAAAGCAAGTGCAGACTGATTCGACCAGTCTCGCTTCTCCCTTCGCCAACTTCTTTGCCTTTCCCCAGTACTCGAGCTTCTCTGGCAGAGGCGGCGGCGGCGGCAGCAACAGCGGAACGGTTACTGACAACCAATCAGCAGTAGCTGACATTGAGGTGACGGTGGTCGAGAGCCATGTCAATCTCAAGGTTCTGTCGAAGCGGCAGCCAAAACAGCTTCTGAAGATGGTGGCAGGGTTGCAAAATCTCCGGTTCATACCGCTGCATCTGAATGTGACCACTGTGGATGACATGGTTCTCTACTCATTTAGCCTTAAGGTATAATACAATGGAGGTGACATTTTTTCCCCCCTTACCAACGTTTTGGTATTGTGAAATTGCAAAGCTAATGCATCTCTTTGTCTTTTATTTGGCAGGTAGCGGATGATTGCCATTTTACTTCTGTGGATGAGATTGCAACAACTGTGTATCAGATGATAGGGAGGATTGAAGAGGACGCTAAGTTTAGTAAATAAATTCCCAGCATTTAGAGTGATTTCGAGTTACAAGCATTGTGGAGGTCTCGTGTGTGAGATTAAGTGTGTGCACGATTTATAGCTGATCATGATGGCCCTGAAAGGTTTGGCATCAGAATCTCTGGAGATCCATTTCAGCAAAAATAAAACAGGCTCTCTAAGATAAGAAATGGGTTTCTATTTCTAGAATCCATTGATCAAAGACCTGTTTCGATGTCAGAAACAAGTTACTGAGTGATAACTTGTGTGGATTATATATTCTTCTGGAATTGAAATGTAGGAAACTGCTTCCAGTGCCCTGTAAGGTCTCAACGCCGTGAATTTAGAATAGTATATTCCACATATGAGATGATTGATCTCAGTAGGAGAAAATGGTTTTGACAACTGAACATAATGTGCACACTGTTATAAACATTGTAATTTCTTGTAACACTTCACTGTTCATTCTCTCTTAATTTGCTTAAG

>Aco004136; *Ac*bHLH90
CCCCACTTCTCCTTCTACTTGTCTCTCCTTCCATCCTCAACTCACCATCTCTTGTCCCTCAACATGCCAATCTGATCACTATACTGCACCATCCAGTACTCCACCAACATAACTCCCACCATCTCTGTCTCATCCTCTATATATCCCTCACACTTACCGACCTTACCCTCTTCTATATATACCCTCTCTCTCTCTTACTCAATCCTAGCTTACATTGAAGTAGCTAGCTAGCTATATATATAGTAGTTGCTAGTAGTCCACATTCCGAATTAAGCACAACATCATGTCCAGCAGGAGGTCGAGATCGAGGCCGTCGAGCTCGTCGAGGATCAGCGCGGAGCAGATTAATGACCTCGTGTCCAAGTTGCAAGCGCTTCTTCCTGAAGCTCGCATCAGGAGAAATGATAGGGTAAGTAAGTTGTGAAATGCAACTATTTTTCACTAAAAACTTAAACTGCTGTAGAATTGTATTTAATGTATTATAATCAGCAATGTCGCTTACGCCTATATTTAGGCTAGCGCTCTCGATTTAATGTAAAAGAAAAGTTAATTAGATTACAAGGCTCGAAAAACTTGACCTCGATCATGAACTCCACGTCCGGTATTATGTGAAATAATCATTATTCTCTACAAGTTTAAGCTCCTAAGTAATGCTACATTTTATGATCTATACGTATAAATGCAACCAAAGTTTTGGTTTTTGCAGGAGTCTGCGGCTAAGGTATTGCAGGAGACTTGCAACTACATTAGAAGCTTGCACCGAGAGGTCGACGATTTGAGCGAGAGGCTCTCGGAGTTGCTCGCCACGGCCGACAGTGCCCAAGCTGCGATCATCCGTAGCTTGCTCACGCAGTAGATCTCTATTTTGGCTGGGCATGCATAGTTTCAAACCTTGGTGTTATGTTCTTGGATTTCATGGCTAAACTTTTGTCAGAAGAAGCATCACCTTAATTTCATTTGATTGTAGCTAGTGAGCCTTTTGTTAGTTGGTCCGTGGTGTACTAAACCTTAATTCGACCTCTTATAAGAGCTAGAGTTTTGATTCCAT

>Aco004138 *Ac*bHLH91
ATGTCGAGCCGCGGGAGGAGCAGGATCACCGACGACGAGATCAATGAGCTCATCTCGAAGCTCCAGTCGCTTCTCCCCGAGTCCCGTCGTAGGAACATTGACAGGGTAAGCGGCCTCTCTATCTTTGTCGTTGTTGTTATTGTTCTTTGCATTCTTAATTTTCCTTCTTTTGATTCGTCAAAAACTTTCAACATAATCACATACTTCTCTCAAAGATCAAACTATACTCTTTTTTTTTAAAAAAAATAATAAATATGAACTAATTCAGACTGTGGAACTACAAATGGCTGACGTACTAATTATATCGATCATATTTTGTTCTTATATAAACTTTGCTATGAAAATAGGCATCGGCTTCGAAGCTACTAAAGGAGACATGCAGCTACATAAAGAGCTTGCACCGCGAGGTGGACGACCTCAGCGACCGCCTTTCTGGCCTAATGGCGACGATGGACGACAACAGCCCGCAAGCGGAGATCATTCGGAGCCTTCTTAGATCCTAA

>Aco013347; *Ac*bHLH92
ATTGCTTGCTCCTCCCCTTTTATGCTGTGTGTGAAAGTTCCACTTTTTTTGTCCCTGGGGGGCTCTTTGCAGTGAGAATAGAATACATTTGATCCTAATGTTCTGATAAAGTTATCCAGCTGGTGCATTGTAAGATTATATGCCTTCTACTTGGCTCCTCTTTTTTTTTCTTTTTTTTTTTTTTTTTCCTTAATGTTTTCTTTGGGATCCTCTCTCTCTCTCCTCTCCTCTCTCTCTCTCTTGACAAGGAATTACTTTTGCTTTTTTACCTCTTTGTGTAAAGCTGCCAGTTTTAGTTGTTCCTTGTTGAGCCCTGCAAGAAATTTACCAGTCAATCTCTCTCTCTCTCTCTCTATCTCTTATCTCTATCTCTTTAGGTAAAGATGTTGTCTCAGAAGCTTCATATAAGTTGCTTCATAACTTCATCTCAAGCATAGCATACTTCAGTAGCTGTAGATAGGATTTTGCTTGGGTAATTGAGATCATAATTTCTTTCCCTTTTCTTTTTTAACTTACTGATTTTCTGGGTTTTTGGGTGAAAATTTGTAATCTTTGTGTTTTCAGAGGTGTTCAGCAATGCAATCTGATCAGATTGTCGGGGCTTCTTCTGTAAACACTCCGTGTTCTTCGGCACGATTCGGCGACCAATACTTCGGGCAAGCTATGGACTTTAATGGGCCAAGTGGTTTTTCAAAGTATAATAATTCTGCGACGCAACAAAGACTCCAATTTTACAATCCCTCGGCCGGTAAAAGTTTGTTTAAACTGTAAATTCTCTTTTCTGAAAACAATACTGACGAACTGCAAAATTTTCTCTCCTCTCTAGATTTTTCTACGGGTAGCAAGCGCAGTAATGATAAAAATTTCAAAGGAAAGAAACGAAAAGGCGCCGTCGATGGTGTTTCTATATATTCCGAAACGCCGCAGGATAGGATTCAAGTAATATGGCTCGAAATATCTTATTGTTTACCTGTTTTCTTCTTCTTCTTTTTTTCTTGTAATTGAATTTGATGATGGCTCAGCAAATGGAGGTTAAGATGGAGTCTCTGAGCAACGCTGTCGAAAAAGATGAGATAAAATTACACAGTGGAAATCAAGCTAAAGAGAGTACTTTCGACGGCGATGCTTCTAAAGATGATTACATTCATGTTAGGGCAAAGCGCGGCCAAGCGACCAATAGCCACAGCCTCGCGGAAAGAGTGCGCCTTTATTCTACGAAAACCATGATTTGTTCTTATTTTCTCCTTTTGTCCTTCTTAGCGTAATTTCTTTTTTTATCGCAGGTGAGAAGGGAAAAGATTAGCGAAAGAATGAGGCTGCTTCAAGATCTTGTTCCAGGTTGCAGTAAGGTAACCCTAACATATAATTTTGTATCTATCCCCTACTATTTTGTGACATGTTTGAAAGGGGCTACTGTTTAATGTATGTTCTACATGGTATTTATTTGATAGATTACTGGCAAAGCGATGATGCTTGATGAGATTATCAATTATGTGCAGTCGTTGCAACGACAAGTCGAGGTACATTCCCCGATTTTCGAATCTTAGTTGTTTAAAAAAGTAATTTGAGACTAATTGAATGGTTAGGCCAATAATTCAGGTCTTTTGTAATCTGCAGTTCCTATCGATGAAGCTCGCGGCTGTGAATCCAGAGATGAGTTTTAATCTCGAGCAGATTCTGACTAAAGATGTAAGAGATAGAAATGTTCAGAAATTGGAAACTAATTCTCCTTAATAGTATAATTCCTCGCATGTTCTAATTGTAGTTTAACAATTGAAAGAGTTTTCTTTTTTTAAAAAAAAACTTTTGAATAGATTCTTCAATCACGAGATATGCGCCCGGCTCTTCTTGGATCTGGTTTGGGATTAAGCAATGTTCAGAGTAGTTTTCACGGGTCGTCGCCAAATGGAATCGTGCTTCCGGAAATGTTGTTCGGTATCCAAAATCCGGGGAACACTCCTGCGACATCCATTCCCCAGTTCCCCACCATCTCTCAAGTAATATACAGCATCTTGTTGCTGCATTTCCTTTTATTTGCCTTACAATGCTCCGCGAAAAGCGGAAAAGAAGGCGAAAGAATGATGTGATAATCTTTTTCGAACTTCATTCGTCTCAGATACCCGGACTGTGGGACGACGAACTTCAAAACATCATACATATGGGTTTCATACCGAGCGCGGTGACCGGCGGAACAGAGAACAGCAGTACAAATCTGAACTTTGGCTTCGAATCCCATTCTCGCAAACTTTTTACGTCCTAGTTTAAGTAATTTGAGTTTTGTTTAGATGTATCAGGTTCCATGAAGGTGGTGGAATAGCAAAAGCACTTGCAATTTCCCTCTAATTGATATGCTGTATATCTCGGCGGATTCTCCGACAAGTGTTGTACAGTTTCATAGCCCTAACCATTGATCTCTCTTTTTGTTCTCTTTTTCATATCTCATATAAAAGATGCAGTACACTGAGGATCACTGGGTGGGTTAGATTGGATTGGAGTTGATGTTTGAATCTTTGATTGGTGTTGTATTATTTCTTGTATTTGGAGAACTTGGGAAAAAATTATTGGTCTCTAGGGAGTTATCAGTTATGTTTAGCAAAAG

>Aco013365; *Ac*bHLH93
GTGATCATAATTTCACAGGAAAGTGAAGAACCCACCTTCATGTACCAACCTCATGATATGGGCTCTATGGATCCACTATCACTTGTTGATGAGGAGAACTCACCATTTCCCGTAGAAAACTATGCTCCTGATTACTGCTTCGATCCCAGCTTCAGCGGTTCGTCGTCGTCGCCGACGAGTTTACGCTTCAATCCATCCATGAATGCTGTTAAGACTGAAGGACCCCCCACTGCAGCAGCATCTGCTGCATCTGCATCTTCCAACATCCTCTCATTTGGAGGGGATGATCATTTACTAAGGCCAATACTAAATAGTGATCATCACAGTGGAGCAGCTACTATGTATTCTCATGGTGTGGTGGGGAAGGATGAGACTATTGATTTCCAGCAGAGAGAGAGGAGCAGCAAAGGGAGTAGTAGAGCATCTTACTGTACTGCTCAAGAACATGTTATCGCAGAGAGGAAGCGGAGAGAAAAGCTCGCGCAGCAGTTCATAGCCTTATCAGCAATGATCCCTGACCTAAAAAAGGTACAAAAATTGTACAAATTCACGTTCATTAATCATGTATATTATAACTTACTTTTGTGTAGATGAAATTGTTCTTCTGAGTGTAATTTTCCTAGAAAACACTATTATTAAGAGCAAAACGATCGTTTTAGAAGGTGGCGGAGCGGTAGCTCGAAGTTCAAATGATGACTTACTCTCTTAAAAGTTTAGTATCATTTGCGACAAAAAAAAATTTGTGGCGTAATTTCACTCTGAAATGAAGCCATAGGGAATCCCTAGCTATATGCATGCATTATGCCACAGTTTAATTTGTGACATAAATATTCAATAAATTTCCATTCAGTGCCAATACATTGAGGAATTTTTGGAAACATGTAGTTGTTGATTAACTTACCATTCACTTTATATATTGATTCAAAAAAATTACTCTTTAAAAGATGAATGCTAATTAACAACTTGAAACCAATACATTAGAAAACAATAAATGTAACGTCTTTTGTCGGAATGTTTTCGACCAAAACCAAGTACTTAAGCACCAAAATTCCACCCATTAATTGATTTTGCAGACCGATAAGGCCTCTCTTCTCGGAGGCGCCATCGACCACCTCAAGCAACTCGAAGAAAAGGTGAAGGTCTTAGAAGAACAAGCCACAAGGAAAACCACTGAATCCACCATTCTCCTGAAAAATTCTCATGCAATATCCAATGCTTCATCAGCTGATCATGAGACCCTTCCTAAGGTTGAGGCCACACTTCAGGGAAAGACTCTTCTCTTAAGGATCCACTGTGAAAAGAGGAAAGGAGTGTTGGTGATGATTTTGTCCGAGATCGAGAAGATGTCCCTTACCGTCGTCAACACAAGTGTCGTGCCATTCGAAGGATCGTCGCTCCACATAACCGCAACTGCACAGGCATGTTGA

>Aco019856; *Ac*bHLH94
GGCGGTGGTGGCGGTGGCGGTGGCGAAGGCGAAGGGTTCGCGTGGTCGATTCCTTCTATGCATCACCATCATCATCATCATCAAATAAGTTCCTTTCAAAACCCGTTATTCCATTCACTCCCACCGCTACCGCCGCCGCCGCCGCCGCCGCCTCCTCCCCCTCCGCCGCCCCCTACGTTCTACGGAGACCTCTATGCAAGAAGGGCATCCGCCGCCTTACAATTCGCTTACGACGGAGGCGGCATCGGATCATCGTCATCGGATCCCTTCGGACTAGCGGGGCTCTACATGGGACCGGAGGCGCTGGTCCGCACCGGGGGGTTGGTGTCGTCGTCGTCGTCGCCGTTCAACATCGGGCTCCATCGGGAGCTGGGGAAGATGACGGCGCAGGAGATCATGGACGCCAAGGCGTTGGCCGCTTCCAAGAGCCACAGCGAAGCCGAGCGGCGGCGGCGCGAGCGAATCAACGCGCACCTCGCCAAGCTGCGCAGCCTCCTCCCCAGCACCACCAAAGTAAGCACGCATACAAACGCACAAATCTGAAGTTCACGGTCATTTTGATCACCATTCTCGATATATACGTAGCTAGGCATAAAGATGTAGTAATTCATAATAAAATAAGATGGGATTAAGCATTGCTCTATCCAATATTACTTAGATTTCCAAAGAGTAGGGAGCTAGCTAGTGACATGTACATACACTAGGTATGCTATAGCTAGAGAGAAAGAGAGAGAGAGAGAGAGAGAGAGAGAAAGAGGGAGGGACAAAAGGGTAGAGAGGAGCCGCCTAGTTGACATGAAAAAGAGGAACCAGGGAGGAGGGAGGAGGCAGTGGAGCAAAAAGCTTACACAGAAGATGCTGTTGCCGGAGCACAGTAATCCTAAGAAAGACAAACACGCGCACTAGTGTCAGAACAGAGAAAGAAAAAAAAAAAAAAGCCACCCACTACTCCCAATATATATCCTTCTCCTCCTCCTCCTCCTCCTTAACCACCCCACACAGTTACCCACATATACACAAAAATTCCATCCCCACATCTGCAAGCGTGCATGATGCTTAAAGCCTCTCTCTCTCTCTCTCTAGATCTCTCTCTCTCTCTCTCTCTCTCTCTCTCTATAATTCCACTACTATTAAAAAATGACTGTGAACCAGGCCATGTAGTGATCGTGTTACTAATATATTCTACTTATGATTACCCGTGTCTGAATCCCACTAGGATGGATCCCTAGCTTATTGCTGTTGTTGCACTGCATGCTACTTTCGTTGTCAGGCTCATTTGCATGCTCTTGGTTATTATTATTATTCTGTACTGGAGCTCATCTTTCCCACCAACCCACTTCCCCCTCTCTCTAATAAGTTAATCATGCATTTAGTTTAATTAGATTTAAATTCAATTCAATATAGTCTCTTGAATTATATTTCGATGTGCGTACGTACGTAGACGGACAAGGCGTCGTTGCTTGCGGAGGTGATCCAGCACGTCAAGGAGCTGAAGCGGCAGATGTCGGAGATCGCGGAGGAGAGCGCGCCGCTGCCGACGGAGTCCGACGAGCTGTCCGTCGACGCCGCCAGCGACGACGAAGGGAAGCTCGTCGTCCGCGCCTCGCTCTGCTGCGACGACCGCTCCGACCTCCTCCCGGACCTCATCAAGACCCTCAAGGCCCTCCGCCTCCGCACCCTCAAGGCCGAGATCACCACCCTCGGCGGCCGCGTCAAGAACGTCCTCGTCGTCACCGGCGACACCGACACCGACAACGACAACGACAACGACAGCGCCGCCTCCGCCTCCGCCTCCGCCTCCGCCGCCGACGACCCCCAGCCACCGCCGCCGCCGCCGCAGCAGCAGTCTCTCGCCTCAATTCAGGATGCGCTCCGGGCCGTCATGGAGCACCGCGCAGCCTCGGCCGAGGAGACGACGTCGGCGGGCGGCGGAGTCAAGCGGCAGCGCACGACAAACCTCTCCAGCATACTCGAGCACAGGTCCATCTAA

>Aco005839; *Ac*bHLH95
ATGGTGATGGGGAGGCTATGGTCCGAGGAGGAGCGGGCCATGGCGGCGGCGGTGTTGGGGCCCCGCGCGTTCGAGTACCTGTCGGGGTGCCGCGTGTCCCCGGAGGGCGTGGTGGCGGGGGACGTGGACGCGGAGCTCCAGACGAAGCTGCAGGAGGTGGTGGAGGGCCCATCGGGCCGCCCCTGGACCTACGCCATCTTCTGGCAGGCCACGCGGGCCAGGACCGGCGAGGCCCTCCTCGTCTGGGGCGACGGCCACTGCCGCGACGCCGAGCCGCGCCGCCGCGGCCGCAGCATCGTCGCCCGCGCCGGCGAGGCGGAAAGAGAGGAGGAGCGGCGGCAGCGGATGATCAAGCGCGTGCTCCAGAAGCTGGACGCGGTGTACGGGTGCGGCGACGAGAACGCGGCGATCCGCCTCGACCGCGTGACCGACGCCGAGGCCTACTTCCTGATCTCCATGTACTTCTCCTTCCCCCTCGGGGAGGGGGGACCCGGGAGGGCGCTCGCGTCGGGGAAGCCCTTCTGGGTCTCGGACGTGGCCACGTCGTCGTCCTCCTCCTCCTCGGAGTTCTGCGTCAGGGCGTTCCTCGCGAGGTCCGCGGGGTTCCGCACCGTCGCGTTCGTGCCCCTCGACGGCGGCGGGGTCGTCGAATTGGGCTCGCTCGATCGTGTGCCCGAGGGCTTCGAAGCCATGAAGATGATCCGGTCCGTGTTCTCCCGCGGCGAAAATCGCTTGAAGGAGTCCATTCCCAGAATCTTCGGAATGGACTTCACCAGCATCGGTCGCCCGCCCCAGATCTCCGCCTCCGGGCCTGAAGAGAGGCCGGTGCATGCCGGTGTTAACGGGCTGAATTGGAACCGGATGGATGATAACAAGTTCCTCAACAGCGTGCTAGGAGGGTTCGGCGCCGACCGATTGGGCTCAACGCCGTCGCCGTCGCCGTCGCCGTCGCCGTCGCTGCAGCGACCGATCCCTCGCCCCGCCGACCTGTTCACGGAGGAGCACCCTCGCATCAGCCAATCCCTGCTTCAAAAGCCGCGTCCGCGTCCGCGTCCGCGTCCGCAACAGCTGCCGCCGCCGCCGCCGCCGCTTGGGACAGTGGATTTCGGCAATGGTGGTGGGGCGACTTCGGCGGCGGCGGCGGCGTCCCCCGCTGCAGCCCAATCGGGCGGCGCATTAGCATTAGACTCGGACCTTCCGGACGTGGAATCGTCGAGCAAGGATGATCGGAGGATTACGGCGGCGGCGGCGGCGGCGGCGGAGGAGCGTAGGCCGAGGAAGAGAGGGCGGAAGCCGGCCAATGGGCGGGAGGAGCCGCTCAACCACGTGGAAGCGGAGCGGCAGCGGAGGGAGAAGCTGAACCAGCGGTTCTACGCCCTCCGGGCCGTGGTGCCCAACATCTCCAAGATGGACAAGGCCTCCCTGCTGGGCGACGCCATTGCCTACATCCAAGAGCTCCAGAGCCGGCTCAAAGAGTTCGAGTCCGACCGAGCCCGCTGGAACAACCCCGGCCTCGGACCCAGACCCCCCGAGCTCGAGCCGCCCTCGGTCGAGATCCAAGCGCTGCACGATGAGGTGATTGTTCGGGTCACCAGTCCTTTGGATGGCCATCCCCTGTCCAGAGTCATCCAGGCTTTCAACGAGTCTAACGTCAGTGTCGTCGATTCTAATGTATTGGCCACCAATGGCTCCGTGCTGCATACTTTCGTCGTGAAGTCGCCGCCCGATTCCGAGCAAGCGACGCGAGATAAGATTGTCGCCGCCATTTCATGTGAGATGACCACGTCGCAGTCCCAGTCGCAGTGA

>Aco005872; *Ac*bHLH96
ATTTATGCGTAATTAACTCTCTCTCTCTCTCTCTCTACATGTACTTTTATGTTCATTGATCATTAGAAGCCCATAGGAATCTTTGCAGAGTTGAGTATTAATTCTCTCTTAATCGATTCTCTGTTGATTCTTTTTCCAAGCAGTTCTTAATTAATTTGGACTCAAATGGAAACTGGGTGGTACTACTCTTCCGAGGGCAGTGACATGGACTTCTCCCAGACACTCTCTGATGGCTACTGGGGATCCTTCTGCAGTATTGATAATGGGTATATATATAGTTTCCCTTTGTTAATTTTTCTAATTTTTATTTTTATTTTTAACAGTGATGATCTGTAGATTTTATTAAAACCACTCTTGGGTTCGAGTCATAGTTGGGGGGCTCGTGATAACTTTGTAGAGATACAAGCAAGCTTCTAAGCCTCAGAAAGAAAAGAAAAGAAAAGAAAAAGTATATGGTGGGTGTGTTTTTTAGTATCCATATATGTTCATATAAGGGTATACATGCATATTGATTTTTTTTTTTTTTTTTTTTTTTGGGTTCATGGTTTTTGTTACTAGTTTGCTTTGTTCTTAATCTTTCAGTGCTTTTTCTAGATTAACATTGCACTGCAACGAGAATGGTGGTGCAAATTCCATGAATTTAGGCGCAGATCAAGAACAAATGCAGCTTTATCAGGTGAGGTTTTATAACGATCTATCAATATTCTTTTCCATATAAATTAATCTGATTTTTATATTAGAAACTGGAGGTCTAACTCTTAATGCATATATGGCGTCCAGTTACTATCTTCTAAAATGATTTGATAGGACGGAAGTATTCGTTCTTTTAAAAAAATCTCTCTCTCTCTCTCTCTCTCTCTCTCTCTCTCTCTCTATATATATAATTATAATTTAGCTCGGGTTACTGTACTCTTATAAGTATAGACTCTTTTGTACTTAAAATTTTCAGCCGTGGATAAAAAGATATGCGGTTGGATGATAATGATCTTCTAGAATTGAGTGGGTGGTTAGTTGAATAAAAATGATCAAAATAGTAGATCTAACGATCGAAAACTTATGAATATAAAAAAATCTATGCTCATAAGAATATACTAGCGCGAACTATATATATATATATATATATATATATATATATATATATATATATATATATATATATATCCAGGCTGCTGAAATTCAGGATTGCAACATATATATTCAATAATCACAGTTCATTCTTATTCTTTCCTGATGACTGTAGTGCTCATCATAATATATTTGTGAGTTCCATCAACAAAGATAATATTTTAATTTATGGGTGAAAAAAAAAATTTTCTGGTCGGTAATTCTATCTAAGTATTAGCTGTAATGGTGAATTCTATATAAGTATATACTAGTTGTAATGGTGAAGAGATTAACTCTAAGATGTCAACTACGAGTAATGTAATTCTAATTAACTAAAGCTGTTGAAATTTCAACTCAACTTTAATTTAATTTAATTTATCCTTTTCCTTAATTTAATTAGAAACATAAACTTAAAAATATATTATATTATATATTTTTGACAAGTTGGCTATTGACAGATGAACATGCAAACGGACAATAAACAACTTGCAGAAGAAGAAGAAGCAGCAACAGCAACAGCAGCAGCAGCAGACGAAGACTTAGTCTACGAGAAGAGAAAGCCCTCATCATCCTCATCATCCAAGAAATCATCCTTCTATTTCCCAACTCCCTCTTTCGATCTCGATCCGTCGAAGGCGAAGCTCATCACTCCCACTCCACACAGCAGCATCACCGCCATGAGATCTCACAACCAAAACCTCAATCCTAAATTCCCAACTCTGGAGAGAGATGGCGATGCCGACGACGTCGCGATCATGAGGGCGATGGTCGTCGTCATCTCCTCGACTTCCTCGTCCTCGTCTCCGTCCTCCCCCTCGGTCTTGTCCAACCCGCCATCGCAGGACTACTTCCCAACCACGCAATCGCAGCGACGAGGAAACGGCAAAATTGGAGCCTTTAAACGTTACAGTAGATCAGATCTCACCGCGAAATCGGAAGCTGAGGATAATTGTAATTATGGTCAGAGCATGATGAAGAGGTCTATCCTTATGCTGAGGAGGTTGAGCTCCTTCGAGGGCCGATCGCGGGAGTCGAAATCGACCAGTAACCAGTTGCACCATGTGATATCGGAGCGGCGAAGGAGAGAGAAGCTAAATGCAAGCTTTGAGGCGCTAAGGATGCAGCTTCCTCCAGGAACTAAGGTAATTTGATTTCATGAATAATTGTATTGTATAACTCCATATATTCAACACTTCAATATATCATTTAAAACATGAGGTTTTGAACTATTTTAGACTACAAACTACTTTTCGATCCTTTAACGAAAAATTAAGATTGAAAAGGTTTTCAGTTGAGATCCAGTTAGTTAATTATCTGTGCTTCGCTTTCCCTGCTGATGATAATTAATGATTACTAGTACAATAATTTTAAGAACCAAATATTAATAATTACAAATTTACAATTCCTTTCGCTCATCTCTCTTTGTTATATATTATTATAGCTTTTTTTTTTTTTTTCTGATCAATTTGAGTAGAAGGACAAAACTTCGGTGCTCATCAACACAAAGAACTATGTAAACACGCTTCGATCGCAAATCTCCGAGCTCAAAGAAAAGAATCGAAGATTAGAGACGTGTCTTATGATCACAGATGAGGGTAAAGAAGCTAGTCATGGATTAAATGGAAGGGCTCAAGTTCGACTGATCAAGTGCTCGTCTCCGACGACGTCGGAACTGCAACAAATTAACCTGACAATAACACTCAGGGTGGAGTGTGATATCATTGAGCTGGTTCTCCACATTCTTGAATGCCTCAAAGTGATTAGATCTATCAACTTGATATCAGTGGATGCGTACACTTATTCTCCTCAGATGAATTTATTCGCGAAAGCGAGCATAAAGTTTGGGATTAAGGTATGGATATATATATATATATACTGATCATCTGAATATATTTTCGATTTATTCCATGACAATTCAGTTTAAAAAAATTTTAAAAAAATTAAAACTCTACCTAGTGGTTAATTTGTGTCTCTTATTAGCCACATGAGAACTAGTTATTCTTATTAACTAATGGTAGATGTTTCCATGTATACTTGGTGGTGTTCATGGACATCAAGTCTAAATGTAAAGTGATGATCAAAAATTTTTTTTTTTTTTTTTGAGAGATAGGTAGCACGCTATACTCGCTTCATTTATTTCATTTATTTAGAAATAAACTTAATTAAAAATGTGAATCAACTAGGATTGGAACTTGGATTTCGGATACCAACCACCAAATCCTTTGCCACTTGCTCTAGGGACCATCGGTGATGATCAAAATATTAATGCTAACTTAATTTGTAGGCTTCTTCCTTGTAAACATATGAAAATTAATTCATCGCAATTCGCAACATCTAAGCTCGGTTATTTTTTCTATTTTTTGTATTTTTCTCTTGTGCTCATAAATGAAGCATCACTAGTTTTGCAAATTGCTGTACGCGATGGGCGGTAAATTTGACCGACCACTGAGGGGGACATCATTAAATTCATAAACCCAACCAACTAATCCCCTCCAACTTATCTCTCATAATAAATTTAGGATATATAATTGATTAATTGTTGGCTAGATACTAGATTATTGCATCAAGCCAAATTAAATTGGGTTCATTACTTCATTATCTTGAATTAGATTAACGAGGTGTACGTCGTTGTTAATATAATTAATATAATAATAGTGGTTGTTGATAATGGAACAGGACTGTGACTGGGACGAGGCACTCTTCCACGAGGCGATGACCAGGGCTGTTGATGACGTCATACTTAACACCGCCACCCCCATTATCTCAGAACTCAGCCGCCTCTTCTGATCGCCGCCCCATCCGTTTTGCTGATTGTTTTGCTAATTATTTACGTATATATATATACATGGAATGCTTCAAAAGGTGGGTTAAAGAGGGCTCGCTCTGTATGTACAGTTGCGCGTACAATTTGCTAATTATTTGCACGCATTCAAAAGCAGTGGGATGCTTCTCGGATGTATCATATTTACATTGGAG

>Aco003204; *Ac*bHLH97
ATGTCTCAGGAAGGAGATGGAGGAGGAGGGCAAGGTGGATTCCCATGGGAAAATCCACAATGGGCCTTTTCAAATTCGGATAACTCCGGCAGCGGAAGAAATGATCCAAAAACTGGTACCCGGCCTGTCGAGTCGACCACAAATAGCCCGACGAACCAGAAAGATCCGGGCGAGCGGAAGCGCAACCGTCCGGGGCCGTCGAAGAGCTCCTCCTCCAAAGGAGTTGGCGGTGTAGGGGATTCGGACCATGAATTACATATATGGACCGAGAGGGAAAGGAGGAAGAAGATGAGGAACATGTTTTCTAACCTCCATGCCCTCCTTCCTCAGTTGCCTCCTAAGGTATAAATGTCCGAACCATGCACATTGGCTTAGTCAAAGGTTTTTATGTTACTTTTTTCTTTTTTCGCTTCTAGTTAGGATTTGTTCTTGGACAAGGTCTTGCTCTCGTTCTAGATCAATGTTTATGTACAATTTTCTCTCTCTCTCTCTCTCTCTCTCTCTCTCTCTCTCTCTCTCTCTATATATATATATATATATACATATATAGATCGATTATGCATGCAACTTAAAAAGTTGATTCTTATTTTGAACTTCTTGGTTATTTAGTGAGTTGGAAGTGAGTATCCGTTGGGCTAATTTTTCAGTGCATTTATGGCTGTTTCACTTCCTCAAAAAGACATATATATATATATATATATATATATATATATATATAATATATAATATATATATATATAGGATGTGCACTTGCCTGTAAGTAACCTATTCTTCTCATAATTATTCTCCTCATAACAGAGAGAGAGACGACCAGGTCCGGTCAAAGTTTCTTTTTTAAAATTCACTGGGAATTTATTGTTAATTATAATGCTTAATAGGATCTTCTTAGCAAAATTTTCAAGGTTATATATCATTCTTATTATGAATAAACCCCATTTCTAATGATTAATATTGAAAAGTTTCACCCTAAATCAACAAATTTATGATTATCTGATTAAGTAGTTTCTTATTTTATATATATATATATATATATATATATATATATATATATCCATGCATAGGCCGACAAATCCACCATTGTAGACGAAGCCGTGAACTACATCAAGACCCTGCAACAGTCCCTCCAAAAGCTCCGGAAGAAGAAGCTGGACCGGCTCCGCGGAGTAGTTCTGTCGGACCCGTCCTCGTCCTCCATCGTCGGATTCAACACCGCCAACATCGCTGACCCGACGAGGGAGGGGTTCATGGCCGAGCAGGGAAAGAAGTGGGCGGCGATGAGCTCTCCCACCGCGCTTTCGATCCCGCATTTCCCCTTCTGCTTCCAAACTTGGTCGACGCCCAACGTAGTGTTGAGCGTGGCAGGAGATGATGCACACATAAGCATCTGCTGCCCCAAGAAGCCGGGGCTTTTCACCGCCGCACTGCATGTTATGGAGAAGCATAAACTCGAGGTGGTGTCGGCGCAGGTCTCCTCGGATTACTTCAGGAGCATGTACATGATCCATGCCCGTGTAAGTATATAACCAAAGCAAGTATTCCTTTTTTCATGTCCTTTTTCCTTCAAAAAAGAAGTTGATTACTTATTTTTTCTTATTGTCCTCCAAATTTTTCTTTAGAAAACCTGGTGCAATTAAAGAAGGTAAAAATGTATGAATTTTATCTAAATTAAGGTCCGTTTTAGAATGACTAATCAACCTTAAAAGGTTTTAATTTAAGCACAAAATATTTTAATTTATTTAATTTGAACAAGTTGATAACTTTTCTATTATAAAATTTGGATGCACTATTCAGTTTAAAGGAAGCTATATATATGTGAAACACTCTTATCAAGTGTGTTGGAAATAAGCAGTGCATCCAAAATTTTTTGATAAAAATTGCCATCAAATAACTTAAATCAAATTAATTAAAAAAATAAAATAGTAAAGCTAAAAGCTTTTGAATATCAAATAATTAATTCTTTCAAAATTAGATCATTAATATTCAGATAATTAGATCATTAATAGTAAAAGCTTTTTGAAGGATATTCATTTTCAGCCACTCATTTTTCTGTTCACTTGATGGACAAGAGAACAATATCGACAATGTATGAAATTTGATTTTTAGATAATTTAAATAGTTTAGATTATGTTTAACTGTGCCGATCATCAATTTGGAGGCTCTATCATCGAAAATAAATTGATAGTAAACCGAGTCGTTTACTACCGATAGTATTCTAGCAAAACTCCCATATATATATTTTTATCAGCCGAGAATGCAAAGAGACAACTATATACCCCATTCATTCTTGCCATTGGCTATGGACATAATTAATTGTAGTTTATCACAAAAGAAAAAACATCATTATGTTGCTAATAAGAATGATTTTTTTTAATAAGAACATCGAAAGGTTTGGTTTTCTCATTGTCCTAAGTTTAGTCAAATATTTATGTTTAGAATGATATGGAGTATTAAGGTAATTAAAAGATGGTTGGTTATTAAGATTATCGAAATGCTGATTGTTGCATATATTATATACACAAATTATTTTATGTGATCGATCGAGAGGCTACTAATTAAGTTCATTGAGAGAAAAATAAAAAATAGGCTATTAAATTAATACTTGTCTAACATTTATTAGCATAGAGAGATACATAATTTAATTTAGAGTTCTTTCTTATTGTTTGGCTAACCTTTATCCCCACATTCTTTAGCCCAAAAACGGTTCTATTTAGAATGATATTTACTACAACAGTTGTTTTATACAGTGATACTTACTATATGTAAATAAAAATTATCATGGTAAAATTAGCTGATGCTTTATAAGTAACGAGTCATTGCTTTTAAATCTCATTTTTCTGAAAACCGTTGGTTATTCTCATGGGACGTAAACTGTCAAGTACTTGTCAACAGTTCGAAGCATATATTATTCCTTTTTATTTATTCGTACTAAGGATCATATTATAATGCTATTTTTTATGCTTATATAATTCACTTTATTAATCAATCCTCTCTCTCTTCATCTTCTTCTTAATAATTCTTCTTCTTCTTCTTGTTTGCAATGAACAGGCAACCCGAGTTTCTGATCAGTTCCCAGAGACGCTAATGATCGAAGAGATATATAAACTAGCGGTAGGCGAGATGATCCTTTGGATTTCTACATGATCCATGAGAATTAATGCTA

>Aco003613; *Ac*bHLH98
ATGGAACTCTTCTCAAACCAACACCACCAAGCTTCCCTCCTGAGCCCTTCCAACCTCCCAAACAGCTTCATGGAGAAGAACTTTCCTCCACAACAGCTTGGGGAGATGAGCAATGAAACCTCCTACTGCTTCCCTTACTGCTACTTGTCAGAAGCCATTCCAGAGTTCTCAAACAACAGTGACAGCACTGCTAGAGCCTATGAGAGTAGCAGCTCTCTTGACACTGTGAGAAATGCTTCTTCAGCTGGGACTCAGATGAGTCATTCAGCAGTGATCACTGACCCAGGAAGCCCCTCTGGGAAGAGGAGAAAGAACAGAGATAGCACTTCCTTGTGTTTAGCTCCATTATCCAATGTAGGATCAAGCACATATATATATATATATATATAGACACACACACTAGTTGCATTAGTAGAAGTACTTTTTTAACAAGTTGGGTCTTGTTTGGTGTAAATAAATAGGATGCAATGGAAAGCAAGACCAAGAAACAGAAGAGACCCAATGGTGGTTTGAAGAAAGTAGAGGAGAAGAAGCCAAAAGGTGATGAGATAAAGCACAAAGAGGTTTGTGGGGAGCCACATGAGGGGTACATTCATGTGAGGGCAAGGAGAGGACAGGCCACAGATAGCCATAGCCTTGCTGAGAGGGTATTTTTTCTCTTCCCCTCTCTTTTACTCACTCTCCCTTCTTTTTTTTTTTTTTCTTTTTTTTTTCTTTTGTGGTGTTTTGGAAAAAGAGAAGGTACTTATTTTCAAAAATCCTTTTACTTACATAAGTTGTATATGTCATGTCATGGATGTGTTTGTAGGTAAGAAGAGAGAAGATCAATAAGAGGATGAAGATGCTGCAAAGCCTTGTTCCTGGCTGTGACGGGGTGAGGATTCCCAATCTAAATGCGCTTCTTTTTCTATACATGCTTGTTTCTTTGGTGAATTAATGAACTTCTTTTTAATTATGTTGAAAATTTGTTGATGGTTTTGATGAGTAGGTCAGTGGAAAAGCCCTAATGCTGGATGAGATTATCAACTATGTACAGTCCTTACAGAACCAAGTTGAGGTACTTGTTGTCCTTCATTCAATATGATTATTATCTTATTAATTTCTCACTGAATTTTATCCATCTGAAACTACTGTTCATGTGACATGTCCAGTTCCTCTCAATGAAGCTTGCTTCTATGAATCCCATGTTTGATGAATTTGGTGTGGATTTTGAATGCCTCATGAATCACCCAGAGGTAGCTAGGACAAAGGCTAAATGCCAACACACTCTCCTTTTAATTTGGCCCATCTCTTTAGGGTTTGATATTGATCTGACTCTTTATGTTTTTATAGAGCATGCCACATGAACAAGTACCAACAGTGGATCAGACCAATCACAGCCAAGCCACTCCTTCTGAGGCTACTACAGTCAATTATCAAATGGTGGACAATTCAACACCCATTTTACAACATGGACAAGGACCCACATACTTCCCCCAGGTTTGTTATATCTATAAGCAGCTTCATGCACCAAGCAGAAGCACATGCCATGTAAACAACCCATGTCTCTGCTAACTCATGTCCTGCACATCCTTTTCAGGATGGTGGCAATCCAATGATGCAAGTGGGAGGACATGATCAGGGGTGTCTGAATCCATTAGGGTTTTACAGCATCTGCTCTTTTCAGTAGAGATATTGAGAGACAAATTGTATTATGATTGGTGAGTTACAGTTGATCTGTGTCTCCTATTTGTTTCCTTTATTTATATCCTCTCACAAGTTTAACTTCTCATTCAGCACAGTTATCTCTTGCAGCTTGTGCAGTAATTAGGAAGAACCACCATATTTTAGTAATTAAATAGGAGGCAAATAGTACAAGACACAAGGATGGAACATAA

>Aco016776; *Ac*bHLH99
GGANGCCGGGCGCGGATCGTGCCGCGGGAGCTAGGGAGGCGGCGGCGGCAGAGATCGCCGGAGAACGGCGGCGCCGACGTGTCTGGATTTCCTTTTCCTCTCTCTAGGTTTCTCTCCTTGCTTCCTCTGAGCTCTCCTCAAGTGTCTCCCTTCGCGAATTAGGGTTTCGATAGCGCCGCGGTTCGTATGAGCGGTTTTGAGATGTTCTCTCTGAGAGAAGTGCTTCGGCGCCTCTGTGCTGAGATCGGGTGGTCGTATGCAGTTTTTTGGAGGGCGATTGGGCCCTCTCACCGCATGTAAGCTCTGATTTTTCCCATTTTTCTTCTTGTATCTCCGATTTTTTCAGATTGATGAGGTAGGGTTTACCTTCTTCTCTTTTAGTTAGTGAGATGGTTTGATTTGTTTGCTTTTTATTGATTGTCTTGAACAGTTAGTTTGCTTTGGTGAGCTTTCATTGAATCCGAAAAGACTCGCCTTTTTTTTGTTTTTGGTTTTTAAACTAATTTTGGAGCTAACAGATGGGGTTGTGGATACACTTGTGGTGTTAATTACTAACCAGTGAGTAATAATGTGTACTTTTTAGTTGCTTTTGCCAGAAAAGACGTACTTTTCTACGGTTTTATTCTCTATTATTGCTATCATTAACTTTAAAGGTTTAGATTTTTTTTTTCTTTTTTTTCATTTTTTTTTGGTGGGTTGAGGTGGGTGGGTTATGAATCGTAAGGTTCTTGTAGTAACCTATAAGGGAATAATAGAGAAGGAAATAAGTAACTGATCTCAACATGAGAAAACCTCTTGATTTTGTAACGGTTAAAAGAAACACAATAGAAGAAATTGCAATTTAGTTAACCGAAATTTCAATAATAATAAAAAACTGCCTAATGAGTTTTTATTCCTTTGAAAAGTGAGGAATGAACTTGTTAAATGAGTCTAAGCTCTAATTTAGTTGAAATTAATTTTTCGCTTGGGCACCGACTGCTTTAGCGCAAGCGGCAAGCTTGATGAAACTTGATGGTTGTTATCAGAGGTTTCAAGTATGAAGCCTGGTTGCTTCACATTTTCAGTTAAATTCATTTTTCAGAAAATTGAATGAAGCAGAAAGTATGCTACCTTTCTCTCTCTCAAAAAAAAAAATCGCTTTGGCCTGGTAAGATGTTTGAACTGTTACAGTTGAGTTGTTCTTAATGCAGTATGTACTGTGGCAGGCTTTTGGTTTGGGAGGATGGGTGCTGTGGACATACATCAGGCATTTTGGGGTCTGAAGCTTCTGATTTACTACTTGAGGAGCATGGATTGGTTCAGAAGTTGCACAACCAACAAGCTGCTAAATATGGAAGTCAAACAGAGGAAAGTGTAAATGCGCTGGTAAGGAAGACGATGATGACGCAGGTTCATGTTATAGGAGATGGGTGAGTGCATACGTTTCATGCTAACTTCTATATTTCATCCTCACCTTTAGGTTTTGTTTTACATGTTTTAGCTAGGTTTGTGCTTCATAGCCTGTTCGGTGTAAACTTATCGGATAGTTGTTTTTCAGGATAGTAGGGGAAGCTGCTTTTACTGGAAATCATCGTTGGATTGTTCGAGATTCTTTAAAGGATTATGGATCTCTATCAAAGGTATAATTTTTTATTAACTCTGTTAGAAGTTCGCCATATGCTATTTATGTTGCCAAAGTTTAAGCTATTAGAACATTGCACGCCTTATTTATATGCACACCAATATTCATGTGCAGTACTTCTTTACTTGAGGCTTTTGCTTTTGAGGGTTTAGGGTGTAGAATTTCAAGAATTCCTTGTTTATTCTGCCTTTTGGTTGTGGATGGAGCTATAATGTTTGATTTAGATCGCAAAGAGAATTGAGTAGCATTTTTTTTTTGCTTCAATAGGATGCTGATGAGATGAATCAGCAGTTTGCAGCTGGCATTCAGGTCAGCAAAGTTCTTCCTTATTTTTTACCCTCTTCTTGTAATTATTTATATATCAACTTAACTCTGATAAGTTGTTTGTTTTGTATGTTATGCAGACGATCGTTATCATTCCCATACTACCACGTGGTGTTTTGCAACTTGGCTCCACTCAGATGGTAAGTAATTCTTTATGAGCTAGCTTCATGTTTGAGCTAAAGATTTTATGAGGCATCAAAATATTGGGAGAAGAATGATGGATGAGTTTGAAATGTACATTACATTTCTTCTGTTAAAGAAGTTTCAAATTATTGTGCTTTGCTTTGCTGTACTATATGCCTCTGTGTCATAGTTGTTAATATTGGCTTATCCTCTCTGCAGCTTATGGAAAATGTTGGTTTTTTAATACACACGAAGAATTTACTTTCACAATTAAATAATCGATCAGGGTCTTCATTTTCAAGACTAAGTTTAGATACCAATCCCTCCTTTGTTGAGCCTATCCTTCACTGCAGTAGACAGCTGGAAGGTGAAACAGTTGGAGCACGTGTATACACGAAGGCCAATCTGAGTCTTGGCAAGCAGGGAGTGACTAATGATAATGGATCTAAAATTCAGACTCAGCCTTCAGTAATGGACTCAGATTTTACTTCTCGCTTAACATCACTCGAAGAGCAGCTCTTATTCATGTCTCATGATGGATTATTAGAACCTGGAAATAATGCAGGATCAGCAGTACAGGGCACTGGAGCTGTTCGGTTAATATTGAATGGACGTGATAACCCTAATTTGCTTGAAGATTCCAACATTGTCTCACCACATTTGCAAAACAAGTTACTGAATACTGTAGACAGTCTTGGAAAGTTTGGATCACTTCTACATGGCAGTAATGAAATCACCAGGGTCTTATGTGCTAATTCATCTGGCACAGGGACAAGTCAAATGATAGACCAAAGATACAATAATGCAGAGAGTTCACAGCAAAATCAGCCTTCTTCGCCCTACAGAGTTACTCAAGCTGTTCCGATTGAGGGGAATGAGGGGAGACATGAAAATGATTTGGTTGAGGCTGCAGGACTAAGGCGTTCAATTCAAAATGATCGACAAACCCGTTCTATTGCAAATTCTATTGCAAATGAATCAAACAAGATTTATGCTACTGAAAATGGGAATGGCGTGAAGGATGAGAATGACTGCAAGCTTTCACATATAAGAACAGAGAAAACTTCACTTTTGCCTCTTGATCGTTCCATGGAAAGTGATCTGTTTGATATGTTGGGTCCCAAGTTTCGTCAGTTTCATGATGGTTCGGATAATAGCTTGGGTATAAATGCATTTATCCACTCGATTAAGTCTGACGCTTCTCCTGCATTTGATTCAGCAAATGATGATTTCTCTCAATCAGGAATTTTTTCTGTAGCTGACACTGACCAACTATTGGATGCAATAGTCTCAAGCATCAATCCAACTGCAAAACAGAATTCAGACGACAGTATATCCTGTAAAACTTCACAGACAGATATTCATAATTCTGCTTGTTACGGTGGATATGCGCCTTCATCAGAGGTGAAACAAGATGAGTTATCCTACCTTCCGCCTGTGCCTATCAAGAATGAGACAGCAATATCAAGTTATGTTAAACCACAATGCTCTCATCCTCAGAGTACTGGAGTTCACAAATCTCAGATCCATCTTTGGCTTGAAAGTGGCCAGAACATGAAATGTGATAGTATGCCGGCTTCAAATAGTAAAAAACTGGACATAACTGGTAAGGCAAATAGGAAGAGATCTAGACCAGGAGAAAGTCCGAGGCCACGGCCAAAAGATCGCCAAATGATACAAGACCGTATTAAGGAATTGAGGGAAATAGTACCAAATGGTGCGAAGGTAATGTGTGTGCTGCTTTTGACCTTCATCTATTCATGTAAAATTACAATAAAATATTGGTTATGAAGTTAGGCAGATAGCCATACTCGATTGCACTTAAAAACTACTATTCGGTTTCTGATCAATGATCATGGTCGCAATGTAGTATTGCATACTTCTATATATGGTTTGTTCCATTATTTCTATTTAATTGTTGCCTTTTTTGTGTTAACAGTGTAGCATTGATGCCTTACTGGAGAAGACCATTAAACACATGCTTTTCTTGCAAAGTGTGACAAAGTATGCTGATAAACTCAAGGAAAAGGGGGAGTGTAAGGTTTGTATGCTTTAAATTCCCTTTCTCTAAACACATTTCCTGATTTGAATCCAGTTCTCAATATGTTAAGGCTTTGGTTCAGGGGCATATACAGCTTACGCAACTATCTTGCTGCATCAACCTTCTCTCACTACTAAGAGAGCTAAATATAAGGCTATATTATCCATTACCTATTTGCAAAGTGTGATTCTTGATGCTTCTAGATGGTGTCTTCTTCACCATGTAAAGCATTTCAATGGCTACTAATGGCTCATAGTTATGTTTCAATGGAGAACATTGTGCTGACCGTACCTATAATTTTATCGCCTAAAAAGCATTTCATATTCTGCAGAGTATAATTGCATAATTCACAGATTTTAATGCAATCACTAGTATTAGGCATAAAACTTGGCTTTGTACTACTGTTACTTTCAAGGCTAATTCATGTGAAGAACTGGAGAAGCATGCAAAACTTCAGTGTTATGAAAGCTCACTAAGTAAGAAATGGTATTATGTTAAATAAGTGAATTTTTAGAAGGAAATGTCTTCATTCTTAAGTGCAGATAGTTGATAAGGATGGTGGTCTGCTCTTGAAAGACTGTTATGAAGGTGGTGCAACTTGGGCATTTGATGTTGGTTCTCAATCTATGATCTGCCCGATAATAGTGGAGGATCTCAACCAACCTCGCCAGATGCTCGTGGAGGTAAAAGATGTTCTCTCTGTATTTTCTTATTTTTTCCTCCTTATTCTACGAGTTGTTGCCCTATTTTTTTTAAGTATGATTTACAACCATTAATTGCCCAGAATATATGGTCTTTTTGCAGATGCTTTGTGAGGAGCGAGGTTTCTTTCTGGAAATAGCTGACTTCGTCAAGGGTCTCGGATTGACCATCTTGAAGGGGGTGATGGAAGCACGTAAGAGTAAAGTCTGGGCTCGATTTGTTGTGGAGGTATGTTTATTTAATAATTCATGTCCTCACTTTTTTGGCTTGGCGCCTGTTGGGTCCCACTGAAGCTTTTGTAAGAATGTTGTGCGAGTAAACAATTCAGAAGAAGCATTAAAAACTTTTTCAGTGAAAGCTTGCCCATCAGTTTCCTTCTATAGCAGAGGAAAAACCTCTAATCAGGTTGCAGAAGCTTTTCCTGTCACAGAAAAAGTTTTAAGAATTTTTGTTTGCCGAACATCTGGTTGCCCCTTCTTGGAGGGAGAAGCCCTGACCTTTAACTTGTAAGAGTAAGATGAAAAGACAAGTCTCCCCCATTGGCTAAATTCACACGACCATTCCGTACGCAGGCAAATAGGGACGTTACAAGAATGGAGATATTTCTATCACTTGTTCGCCTGTTGGAACAAACAGCCGGAGGCGGTATAATACCAATGAACGTCAATAATGTCGACGCGACTCACACATTGTTGTGCCAACCTAGTAGTATTCCCGCAACCGGCTTGTGAGATTGCCATCAATGAGGAGATCGGCGCTTTATTCAGCCACTTTAGAGATGCTAAGAGTGGGATCGACTTCGCCCTGCCATGACTAGTGTTTCAGTGGCCATTAAGGTTTCCCATTTTGCGGCTTTTTGCACTCTTCTGACTAATTCTGGGAGCCCTACTTGCTTGAACATTCGAATGCTCGGGTTGGCACGAGAGCTTCGTTTTGTAACATTTCTCGTTGTAATCTTTATGTCACTCCTTTTCCATGTACTATTTATTGTTGGATAATAGTAGATTAATTTTGTTGTATTTTTACTTTTTTTTTATCTTTCTTGTATGGTGGGGCTAATGTTTCTGGCTAATGTGTAGAACTATCATTTCAATGCTAATTTAAGCCTAAACCGCTGATCTGATCTGATCAGTCCCTCG

>Aco022880; *Ac*bHLH100
ATGCTCTCGAGGGTCGATGGAATGGTGTGGATGGGCGAAGAAGAAGAGCGAAGAGCAAGCGTAGGTGGGGGTGGAGGTGGGGGAGAGGAGGAGGGCGACTCTCTCTCCTGGACCCGAGCAAACCCTAACCCTAACCCTAACCCTAACCATAACCATAACCATAACCACCCCCACGGAGCCAAGGAGGACGAGATGGCGGGGCTCTCGAGGTTCAAGCCCATGCTCGACGACGACGACGGCGACGACGACGACTGGTACCTGGCCCCGAACCCCGCCCACCAACCGTTCGACGCTTTGCCCACTCACCAACACGGCATCAAAGGCGCCGCCTTTGCTACTGCGCCGCAGCACGAGCCCTCCCTGCTCCTCCCCCCGCCCCAACCGGGCATGGGCATGGGCGTGGGCGTGGGCGTGGGCGTGGNTCTACTCCCCCTCCTCCTCGTCCTCCTCCTTCTCGGGTGCGGGTGCGGGTGCGGGTGCGGGTGCCTCTTCAATCTCGACCACGCCCACCACGCCTTCTTCCCCTCCAAACCCAGCTTATCCTCGCTCCTTAGCGTCGTCTCTTCGAACCCGTTCGACGCCGGCTTCGATCTCGGCTGCGATGCTTCCGGATTCGCCCACGTCTCCGCCTCCCCTGTCCTCCTCAACAAGGGCGGCCAAGGCCAAGAATTGCTCGGCTTCGGTGGCTTCGGGCCAAGCGGCCCGATGGCCGGTTGCCCCGAACTGAGCTCCTTAGCTCAATTCCCCGCCGGCCCGAGCTCCGTCTCGCCGTTCGGGCAATTGGGCTTCGATTGCTTCGAGAATTCGCCCTTCCTGAGCCGCAATAAGGTTCTTCGGCCGCTGGAAATCTTCCCGCCGGTCGGTGCGCAGCCGACTCTTTTCCAGAAAAGGGCTGCCGCCGCGCTGAGGCAGAGCTCGGCGACGACCGAGGAGAAAGGCGGTAGCTTGTGGTTGCGAAGTGGCATTATGGACGAGGAGAGTGAGAAGAAGCGGAGAGGAGGGACCGAAGAGGATGAGGAGTTGGATGAGGGGAGCATTGACGGGTCGGGTTTGATCTACGATTCGGACGATGCCATTGCGGAGAATGCGAAGGGCGAGGAGAATGCTAGAAATGGTACTAGTGGTGGGGGAGGGAATAATTCGAATGCTACTAGTACGGTAACAGGGAGCGGAGACCAGAAGGGGAAGAAGAAGGGGCTTCCGGCAAAGAATTTGATGGCCGAGAGGAGGAGAAGGAAGAAGCTCAATGACCGGCTCTACATGCTTCGATCGGTCGTCCCAAAGATAAGCAAGGTGAGAGTTTTATGTCTGATGAGTTATTACTTGTTGCATTTGACTATACTGTGGTGAATTTTAGCATCCTTCTTTTTGCTCATTCAAAGGTTATTCATGAATTTGAGAGTTATTCTAACTTAACATAATAATTCTTTACTTTTATACATCTACCAATTAAAGAAGATCAACTGCTTTCTATAGGCGAGTAAACAATAATGGTGAGATTGTTTCTTTGCAAAATTAACTAGAATGTCTTTCGATATATTATATACTCTTTTTCTAAAGATTATTTATTAGCGATATCCCACCTTATGTTTGAGACTGAGTCCAATTTTTGCGAAGTTGATGAGTCACTATGTCCGTGTGTTGTATCATGTTTTGTGCCCCATGTTCGTGTCTTTGAAACTTAGGTAAAACACTTCTTGTTTTCAACCAATAAGGACACAGCTTTAGCATTGAGAAAAAGTGGCAATTTGAGACTTTACAAGATAGTGTATAACTTTTAATCTTCTTACTTGTTTGGCCCGATTTATGGGACAACTTCTCTATCTTCGGAGGCAGGAAGGAGGCATTTTGGCGAATAGAAATTTCGAAGCTTTTCCAGTGGTCAGAAGCTAAAGTAACCATTGAGCCATTTGCCAGATAGTACTCTCAAAGCTTTTGTTATGGCGGGAAGCTAAAATGAGCATCCGGCCCCAATAAGAGAAACTCCAATTCTAAGCCTGTACTTCTGCTGTCACAGAATAGTATTGAGGGATTTTGATGGAAAAAACTGTTGTGCCTCTCTACATTGCTTTGCTCAAACAACTGTAAACTACGGATGAACGCTAATCAGTCCAAACTAGCGAACCAAAGTGAATCATTTCAGTTTTTTCTGTTTTATCAGTAATATAGTTTGGCTTATACAGTTGCGGTGCTTGACTACTACTTCTGGTAGATCAAGGTTCTAATGGATAAGTTTCTCAAGCTCATGTCTGTTTTATAATAGGTGAACTTATCCTAGCCCATTCAGAGCTTCCTAGCCTACTACCAAAATAAACCATCCAAACCAAAACGGTTTTTTTTTTTTTTTTTTTTCCTTTTGAAATTAGCCCTTAGTCTCGCACACACACACCTACTTCAATTGAACTTGACAGCTGACGCTCAAGGGACTCAAAGTAGCTACGAAATGGACTACCTTTTGGTACCAAAGCAAATTGTTTTATTCGTTTTGGTTTATCTGGTTCCATTTTTTTAATTTCTTGCCGCTACCATTGCACAAGTATTTTTTTTCCCACCGTAATTTACTTTGTTCTTGCTATTACCACAGATGGATAGAGCATCCATTTTGGGTGATGCAATTGAGTACCTAAAGGAACTTCTGCAAAAGATCAACGACCTCCACAATGAGCTCGAGTCGACACCTTCAACTTCGTCATTGCCTCCCTCTTCTACAGCAACAACACCGACAAGCTTTCATCCTTTGACACCCACACTGCCTACCTTACCTTCTCGAGTAAAGGAGGAGCTCTGCCCTAGTTCGTTGCCTAGCCCTAATAGTCAATCTGCAAGGGTTAGTTTTTATTTACTTAACCAATTTCTCACTTTAATTTTGTGTGCTTGTTTTTGTGGGTGTTTTCACGGGTTTCTAGTTTTATGCAACAGAATCATCTCTTCTCTTTACATATTAAATGTAGAATTAGAGTTTGTTGTGCCCTTTTATCGGATTTGTTTTTTTCTTTCTTTTTACATTGAGCTAACTTTAGTTTGTTCATGAAAATTTCCCATTTTGCATATATACCCTTCCAATTATATGTGAAATTGCACAAATACGCCTAAAAAGTTGGTATTTGCATGCATGCCTTGGAAAATCTTGCTTGTTTGAATGAGAACCCCCTGTTGGGTATACATTACAGGAAATGGTTTTTGCATAAATACCTCTGCAGGGGCATGGATGCAAATAAATATGCTTTTCAGCGGCACATATGCATGTACCAATTTTACAGGGCTATGTAGTCAATTTTGTATATTCGTAGATGTCGTGAATCCGTAACTTGCTTTGGTACCAAATTTGCTCTATAACCAATTTATTACGACAAAAACAGAAAAGATCTAGTTCGTAGGATGCGAGAATATTGAAAAAAAATCAAGAAAGAGAGAAGAAAAAGAAATCATACAAATATTTAATTGGTTCATTCGCTTGGCTTACGTCCATGAACGAAGACGGAGCAGAAACTATCATTATAATCAAAGTGGTATACAAAAGATCTCTCTTATAAAATCCTAGGCCCAAAATACACTCCATACTCACTCTCATCTGCCGTACCAAATCAATGAAATAAAAGGCATATTTATATTAATGACTAAAATCCAATAGGACTAGAAATAATCTCAATAAGATTAGAAATCATCCTAACTTAATTTAATGAATTAACCAAACGTATATAGTGTTAGGTAACCTTGCGGTTCTAGAATTCAATACATATCTACCAGTACAGTTATATAGGTGCACAACAAATATTTTGTATGACTGGGAATTTAGTTTTCTCTGTGCCTTGCTACCCTTCTGCATATTTCTTCTTGTTGATAGTATGTAATGAGTTTTAACTAACTGCCAACAGGTTTATACTCTGTTAACCTAAACTAAATATACATTCTTTTTTTGTGTAAATTTAACAGTTTGCTTCCATTTGCCCCATCTTTTACTATTTTTAAAAGATTATTTGTTGCATGCAACCTAAAGTTCTAAACGTTTGAAAAATGGATATGAATTGAGATCCTACGATCATGAGGTATGATCAATGAGGCATAGTTCTATTTGTATTAGAAAAGCAACTTTTCACTTATACTTCATCATTTGATATATATATATATATATATATATATATATATATATATATATTGCATATATACTGTGCAAATATGTAAAATTGCATAATTACCCTTTTGTATTTACATATGCACGTGAAAGACATACTTGTTTGCACCTGTGCCCCTACAAGGGTATCTATGTGAATACTATGCAGTGGTATTTGCACAACTTCGCATATTTTGTGGGTTATACATCTCCAACAAAAAAGCCTTTTTTTTTCTGTGTGTCGGCATGCTCTTATTCTTCTTGTGCTCAATTAAAGGTTGAAGTTAAGCTAAGAGAAGGGCGGGCAGTCAACGTCCATATGTTCTGCGCCCGTAGACCTGGTTTATTGCTCTCTGCCATGAGGGCACTCGATGGGCTCGGACTGGACATTCAACAAGCAGTCATCAGCTGCTTCAACGGGTTTGCCATGGATATTTTTCGAGCCGAGGTATGCTTTTCCTCTGTTCACAAGTACGAACAAACAGTGCCAAACTTATTCTCTGTTTTCTTGGATGCGTTAGGGTAAACTGCTGAAGAGCCCTCGACCTTCTTGAAAGGTTTCAGTTTGGTGCCCAACCTTTCAATAGTTACAATTGTGTCCCCACATTCCATCGCTTTGCAACCTTACTTTCTCTGTAAAATTTTCTGATTATTACCATACGAGGATTTCGAAATCGCTTGTACAGCAGCCACATGGGAAGAACATGGATATTTCCATCAAATTGAATGGAGGTGTTTGGTGGAAAGAGCAACAAGATTGCTATGCGACGGGCAGGTTGAGGACCGAATTATAAACATTTAAAAGTTGAGGACCAAAAACTGAAACTACGCTAGAAAGTCAAGGGCCTCTGGTGCAAAATTACCCGAATCAATATTTGTTGCTTAGTAGTAAAATTTTCCTTAATACACCTTATGCTCTTTGACAGCAATGCAAGGATGGCCCCGGTGTTCTGCCTGAAGAGATCAAAGCCGTGCTCTTACAATCTGCAGGCTTTCACACCGTCATGTAGCTGAGTTTCTAGCAAGAGTTAAAATATTTCTAGTGCGCCAAAGAGTCGGCAGTCGCAGTTTAATAGTTAGCTCGCATTTTCAGCAGTTGTAATATGAGAAAGTAACAGCGTTAGAACTTTTGTTGCTCTTTTTAGTTCTCAGAATAAAGAGTGTTATCTAGTGAATAATTGTTCTTCCTAATCTGAAGTTCCTTGGTTTTGCTTTCCTGTTCGGAGAAGCAGGTTTTAAGTAGGCATTAT

>Aco026511; *Ac*bHLH101
ATGCATGCTTTTGGTGGAACAAGACGTAGTAGTAAAGCTGAATCTGATATGCCGAAGAGACTTGAAACGAGCAGTGTAGAGCCGGTGCGGGAACCATACAGAGATGTACCGGTGAATCTTATAACTGCCCCAAGAAATGACATGCCTACTCTTCAAAATATTTGGGCTTGTCAGAAGAGTTTTCTAAATGTATGTGGTAATGATGCCGATATGCTTTTCCCTGGTCCTTTTTCAAGCAGTACAAGAATACTTCATGCAAATCCCCAAGCAATCAATAGATTGCATGACGTAAGAGGGTTCTTTGAACTAAAAGATGGTCATTTTGACGATTCAAAGTTCTCATCAAATCCTTTAAGAAAAGTCTCTGGAAACTCATCACTTGCGGACATTGGTGCTTCAGGAGTTAAATCTCTTGGTCCTATAATCAGTAGTAGGATTACAAGCACTGCAAGTGGTTCTTCAAAGGCTGTAGATTCAGTGGAATGCAGAACACATATTGAGGTTTACAATCTTCCTATGGTATTACTGCCAAAAGGATTTTGTACTATAGAATTTCATCTTTTGGGTCAAAATATGTTCTCATAATTCAATATATATTGCTTTGTTTTTCTAGCAGATCACACATTCGATGCTAGATGCAGAAGAACAAACAGGCAGAATGTCCAGTAATGGTGAAAATCAAAATTTCAATCAGGTAATCTTTTCCTTGTTCTGTCTATCTTTTGTGTAAATTGTTGTACATGCCGAAACAATTTTCAGTTGTTTAGATCATCTACACGGGGGAATATCTAGGTACCAACTTGCTGCATTCTAATCCAAATACTGCTTATACCTGACAGAGTTATGCACTGATGAAGCCAAAATCGAATGAGCATTACTTCAATCTAGCGGAGGCTGATTGTAAGGAGATAGTTCGATCAAGCAAAAGCAAAAACAAGAGTGTAGCAAATGATAAGGTAAGGATCTATGTACAAACCATGTAGAGATATTAACTTGTATACCCCTGCCAAATCATCTATCTACATTTATTACCTGAAAATTTTAGATCTGCTCTTCAACACCATAATTTCTTACATATAGATCTTGTGCTAGCATTAGTGTCCCTCTTTAGAAGTAGTTTTCTAAAACGAAACCAACTTGGCCCTTGAATAGGACTACTTCCCAAGGTAAGGAGGGTGTTCTTTTTTTCTTTTTTTTTTTTAACAGAATCTGCATTTGGAGGACTGTATATGAAAATTGAGAGTTTACAATGGTTTGGGGCATGTATGAAGTGGATAATTTTATAGAGAAGCGTGCCAAAAGCCCCAAAATGTATTCCAGAGTATCCATCTGGTTAACATTGAATTATCTATTGAAGAAAGTGGTGCAGAGTTGTGAAACTATTTTAACTTGACTCTATTTGTCCTTTCAATCTCTCTCTTAGGGATGTTGACAATTGATAGTGATGAATAGCTTTATTAAACTCCTGTGTCATGCACAGGCCAATTTCATTATAATATAGGTTTTTTGGAACCCGAATTTATCTATCAAATATGTGCTTACATTTGTTATTTTGCTAATCATAGCTTCGAAGAGCCAGGATATCAGAAGGAATAAAAGCACTTCAAAATTGCCTACCATATTCAAAGAAGGTACATACATGCCACCGAATCTTTCATTCGTAGATGGTTGCGAAGCCTCATCGTAGAATATAATTTTATGGTTAGTTCTCCTTTACATCTCAAATGTTTCCTATGTCGGTTTTGCACTGTCATTGTTTTGTGTGCTGAAGCAAAGCACAATCCTATATACTATCAATGCAAACAAAAGCATCCCATCAAACATTGCCAAGTGTGGCTGTGCATTTCTTTATTGATTTTTGTTATTGCATGTAATTAAAATATGTTTTGGGGTTATACATCTAGTACTTTTGTTAGATTTGCATTGGATATTGGACATTGTTCTTAAATATTTTACATCTTAATTTTTAACACCCTACATACACGAATCCTTGTTGCCTGATGTTTATGAAGTAGGTCATAATTGAGTGTGAGTAGTGCTATTCTCAGTGTTTTAAAAAGCCAAGGCGCAAAAGGCTGCTAGGCGCAAGGCGCAAGGCGTAGGCGTGCGCCTGAGAGAGGTGGGGCGCACACTTGAAATAAACAAATATGGAGGGCCTCTTTTGGATGCTTCCGAATCCATTCAAGAAATTCAACAGCAAGTTAGAACTCCAAAAATATGGAAAAGAAATCTAAAAACTAAGCAATGTTTCAGCAAAATTTCTAATTCAGCAAAAATGCAAGTTTTACTAGTCTTTTTCCAAGTTGAAACGGTGACATATGGTCTTATATTATTTATTTTTTAATCACTTATTCACTTATCAGACAAAAATGCTGTTAAAAAAAAACTAAGCGGAGAGAGGTGTTGCAAGTGTTTATTGTTTACCAAAAAATAAAAGTGTTTACTGTTCACCCAAAAAAGGAAAAATAGAGAAAATAGTGTTTACTGTTTACAAAAAAAAAAGAAAAAAGTAGAGAAAACATGGAGAAAACTTTACTGTTTACAAAAAAGAAAAAATAGAAAAGAGTGTTTACTATTTACAAAAAAATGGAGAAAACACACACTATTTACTTTTTTAAAAAAAGGAAAAAAAAAGAGAACAGAGAAAGAGAGTGTATTTACTGTTTACAAAAAAAAAGAGAGAGAACAGCGAAAGAGCTCTCTGTTTACAAGAAAAAAAAGCGTGAGAGGTAACATGCGAGAGGAAGAGAAATTAAAAAGTGTGAGAGAGGGAGAGGATTGCACCTGCAGCATGATGGCGGAACCTCGGAGAGTGAACGAGAAAGAGAGAGAGAGGTGATCCATGCTCGAGCCGTCAAGCGGAGCACTAGAACCGTGGAGAGCCAGCGAGAAAAATAGAGATTCTTGCTCGAAAGGAGGCTGTGGAGCCGTGAAGAGCTAGCGAGAAGGAGAGAGATGATGGCATGATACTTGCTCGAGTGGCCAGCGAGAAGGAGAAAGAGAGATGATGGCGTGATGCTTGCTCGAGCGAAAAACTCAGGAGTCTGCTCAATTTTTAGGGCTTCTCATGGTTTTTTTTTTCTTTTTTACACTTAACGGGTTTGTTGGATGAGTTGGATCCAAAGTCCAAACCCATCAGGCAACCTTTTTGCTTTGGGCGCGCCTTGCGCCTCAGGGTAGGCGTGCGCCTGGGCGCATGAGGCGTGCGCCTTGGCGAGGTCGCCCGCCTAGGGGCTAGGCATGCGCCTGAGGCACGCTTTTTAAATCACTGGCTATTCTCACTCCGTGGAGCAAAGGGAGTAGTATCTAATAGTTGGAAACTATATCATCAAGTGATTTTCACCAATCGCTGGGAGATAGGTCCTACGAAAACTGCAGAAAGCTAGAAAGGAGCTAATCACTTAAGCAAAGATTCCATTCCTATCTACTCTTTGGAGTAGATTATTATTATTATTCTTGAGCATAATGTTTAACTAGTTTAGAGCCCCACACGATGCGGCGGATAGGCATTATTATAGTTTATTATTATAAATTGTTTGTTATACTTTTAATAGAATTAACGAATATATTTATTTGATATAAAATAATTCTGAAATATTTGTTTACAACTTTATCTCAAATATTGATTCAAAAATATCAAATCATCAAGTCATCTTTATATTTGGTAATTGGGCCAGCCGTAACACTGTTATCATCAAACAAACACTGTATGCACTAAAGCAAGGAAACACACAAATATGCAACATGTGACAAAAAAATGCAATATGTTCTTTATATCAGGCCATTTATTATCCTGATTTCAATCTTATTTAAACTAAAGGGGTATGAAAAATTTAAAACCTACCGATGATAATCGCAAAGAACCTAACTATATGGAACTGAACAATTTGATTTTTTTTGAGCAATTAAAAAAATTAAATAAACTTTATATAGCACGTAGATAAATTTAAAGATCTATATATGACGAAGTAAAACTTAGAAAACTTGGTTATTCTCAATTTTATAAAAGCAAGAATGACAAAACTTGGTTTATTCTACCTTTTAGAGAGAGCCTCTGATTTGTGCTCGCTCCCAGTAGTAACAACATAGCCTGCTCCAACCTGGAAAGGCGATGAGTAAATAGATTACGAACCAAAAGAAATAAGCTAAGCATGCCAGCATGCATGACTAAGAATACTGACATTGTTGATGAAATCAATAACACATCATTTTGAATTGTCGAGAATATAGAAACCAAGATGTCGTGAGAATGCTTTAAGATGAGAACTTAATTGTGCAGGATTTGGTATTAGCATTCCCAGTTTGTTGAAGTTGGAGAAGATAACATGGTTTCTCAAGTTGTCAAAGATATACTCAAGTGGATCTACTCGTTCTTAAGAAGCATACCTATCTTTAGACCGGACATTTGCTTAAGCTCAAACATGTTTTATTCCGACCAACTGTTAGTGCCACATTCCAAGTTAAATATAGTCTATCAAAGTTTGTTACACTGAGGTTTCTTAATCAAATATTTATGCTACCACATAGGCATATATATGAACAATTTCAACAATATAGCAGAAAGCTTGGAGGAGCAATGTGCAAGTTTAACCACCCTTTATAATAGTGCAGTGAAGAGAATGCCAAAAGGAGAAAAAAAAAGCCAAAAGAAAGGAGCACAGGACAAAAAGTAATCACCTGACAATCTTTCAAAACTGTGCACTTTTGAAGCTGCACATTACTGCATATATAGCTGACATCCATCTTCAATAGTCGCATGATCCATCACAACAGAATTCACAATCTGCAACACTGAAAGGAATAGACAACGTAATAGTACCTTTACTATCACATCAGTATGCCAATTTCCGAATTCTATTCATAATCTGATACAAATAGCTATTAATGGCTAACATTAGATTAACCATACTGAAAGACAAAGCAGGTGATGAAGGCTTGACATAAGAAAAATAATACAAAGGTAAAAATTTCTCTCATTACCTTTACATTGGAGCCGATTCTGCAGTAGTGACCAATTACAGATCGCTTCACACTACACTTGTCCAAGAAGAGTGGGCGGGCCCCACTCTCTGTGAAATGCGGAGAGGGCTTCAAGGTTCTCAAATTATAAGTTTATAAGTTCTTAGGGTGTGTTTGGTTCGCACTATGAAGCATTTCTAGTTATTTCAACATAACTTGCAATCTGATTCTTGTGAATTAGATTACTATTAATGCTGCATTACTGAGATTGATTTAGTACTGTAGTGTGATGGCAAATTACTGTACATGTAAGATTGCTGTGTTTGGTTTGTCTTACAGATTTTAGCACTATTCCAAAAATACCCATGCACTCATATTCATTGAAATTTGAATAAATCTTGAATTCAAGTTCAAATTTAAATTCAGATTTAAGTTTTAAATTTAAACATAGGTTAAAATTTTAAATTCAAATTTTGATTTTGAATTCGAATTACAAATTAAAATTTAGATTTTGAATTCAAATTTAAAAATTGAATTCAAACTTCAAATTCAAATTCAGATTTTGAATTCCAATTTCATATTTAGATATTCAAATTGAATTTCAATTCAAATTCATATTTGTAATTCAAGTTCAAATTCAAAAATTGATTTTGAATTTGAATTTCATATTCATATTTGATTTTGGATTCAGAATCAATATTCAAATTCAGTTTTTGAATTCAAATTTCAGATTCAAATTCAAATTTTTGAATGATTGAAATTTTAAATTCAAGTTCAGATTTTGAATTTATATTTCAGTTTGAGTTCATATTTTTTATCTATATTCTAATGAGAAATTAAATTTTGAATCTATTTTAAATTTAAAATTGAGGCAATTGGGGGTGGGGAGAGGAAATTTTGAAAATTTTCATAAGTTGCAGGAGTACCTGCAATTAGTGATGCAAGATACCCACCCTTCTCATCGATAATATTAAAGGGAAATCCTGCGCTGCTAGTAATCCTACATTACTTAGAGGATTACATGTGGGATGAACCAATTGCAGTAATTTGAAAATATTCCTATAGGATTCCCAGTCATCATGGCAGGATAGTGTGAACCAAATGCGTCATTAATGCAAAGTCAAATCTTTATGGTGAATCCATGTAATGCCTATAAAATCGCTGTTGCAGATTTAGATTTTGCTTCTTGTAAGATACATTGTTGATTAATGCTTTTCTTCATTTTCTCTTGACAACCGACACATCTGCATAAAGGGTTAAATGCCTGGATCGAGGATAGTGTAGGCATAGTTTGAATTGAATAGAAAATGAAAATGCTTCACGAAATTGGATAAATCAAAGAAAAAAATGATGCAAACCGAGGTATTGCTAAATATGAATTTTCAACTTTTTTTTTGTTTAAAGGTATTCCTTGAGATAAACTAGGTTCAATTTATCGATTCCGTCGAACTTTACTAAAATTAAAGTAAGGAAGCAAAAAATTTCAGCAAAACGAAAAATTAAAATTGAATCAGAGCAATACTAAGATGGTGATAAACCTTCAATATATAAGATAAAAACACCCAATTGAAAATACATATAATAGTTCCAAAGAGGTAAATTTAGTTTTATCGGACTGAAAGCTTTTAGATTTAGTTGTTTTGATAAAAGTAGTTGGTAATGTGGAAGGCTAGATTGAAAGGGGGATAATAGGCTATCGGGAGATGAAGGAGAAGATAAGATACAAGAAATGCACCGATCACACTATTGCGAGGGATCCATGATCGACGAGGAGGGTCATACACCATGGGTAGAAGTAACGGGAAGATGTGAGGAGGTAGGGATCCGGAAGAAGAGGAGTGCTAGAAGCAAAAGCTGATCTTGCCACGAGGAAGAAGCGACAACTATCAAATTGCGGAAGAAGACCATGTAGAAGCAGAAGCCGATCTTACTACAAGGAAGAATTGACAACGATCAAGTTGAGGAAGAAGACGATTTCGGCACGAGGAAGTAGCATCGATGATCACACTAGGAAGAAGAACACCATGGCGAGGACAAAGAACAAGAGTAGAAATTTTTATTCATCAAACCTGCACTTAAAGTATCAAGAGCCAAGTATTTATAGTGTTAAGAGCCTATCCTGTAATAAATTCTTGTCCTTTCGTTTTCAACACAATATGATCACCTAAAGGTCATGTGATGTAGGACAGAATATAGAGTCCTATGTTTTTAGAGGGCTCCAAAATATAATTTATGATTTTGAAAGCGTTTTGCAAATTTGAAAGTTTTTGAAATACTTTTTTAGAAATTTTCGTAATTAAAAAAATGAAAATTGAAGAAAATAGAAAAATTCAAAAAAATAGAAAAAAATAGAAAAGATAATGAGGACTCACTCCTGAACCGAACTCACTTTGGGATTGAACTCACTCCAGGGCGATACATTCGTGAAGGAAAAAAAAGATATCATTGCCAAAGTATTTGTCTTCATTATAAATTCATAAATTCATATTCATCCCCGTACAAAGAAGCTTTATGTCTTATTTATACTATTCTTAACCTTATCTATAGATAACCAACTTAGAATTTAATCGTATTCTAAATAACCAACTAAAACAACTTTATTTAGAGATTAACAAAAAAATTCTCATTTGACTAACGAAAAGATATTCTCAACTAATCTAATCTAATTTATAAAACGATGAACTAAATCGGTTTAATTCGATCAACAGAGGTTCAGGCTTGTTCTGCATCATTCGCTTTCGTTGCGCAGGATCTCGTACTTGAGATCAGTTGGATTTGTAAGGAGATAGGAAACAACCAAGCGTGCAGAATACAGTGATGCAGCCCAACGCCTGCATGTCGAACCACTTGTATCATATGTAGCCCAATGCCTGCCTGTCGAACTACTTGTATTATTTGAGTTTCCAATTTGGTATTGAAGTAGGAAGAACAACTGATAAGTTCATGAGATTTCGTAAGGTTTGATACTGAAAGAAGGGCTAAAGTGCATACCCTCTTGAGTTGTATTAATGAATATATATAGTACAAGAGATTGTCTTACTGTAGTAACAATGACTACTATAGTAAGAGTATAACTAGGATTACATACCATATAATCCTACTCTATAGAAGGCAATGATACTACTCTATATAAGGTAATAATACACAATATCCTAATATATCGTAACATCCTAATATAGATAAAATATATCGTAACATCCCCCCGCAAGCTAAGCATCCGGTGGTCCGATGCGAAGATTGAGCCGCAATATCGAAAACCGAGAGGATAACAGGGGCTTGGTGAAAATGTCGGCAAGTTCCTTGTCAGAAGGGATATGAATTAAGGCCAAATCACCAAATTGAACTTTTTCCCGCAAAAATTGATGATCAAGTTTAATGTGCTTCGTGCGAGCATGGAGAACAGGATTGGCCGCAAGGTAGGTGGTGCTGAGATTATCGCAATAAATACTGATAGAGCGCCATAAGAAAACACGAAGGTCACGAAGGAGACGACGAATCCAGACTGTCTCAGCAAGTGTGTAGGCTACAGCACGATACTCNAGCACAAGTCATTTCTGACTTATGTTTTACTTCTCTAAATCCAATAAAAATAGCTTCTCACACTATTTTTATTTATTTTTATTTTTATACCAAAATCTGGTATGTTACATTTATGGTGGATGGTGGCTATGGATCGATATCCCCACATTAGGGTTCCCGCTCTGATACCATGTGAAACAACCGTTGTACTCTAAAAGCTTAAGCTGTTAGAGAACGGTGTTTAAACATTTTTATATTTAACACTCCCCCTCACGTATGGGCTGGAGACTTTTTTAGTCGGCCCATGACGTGGGCTTGAATTAAATGGGAGGAAATTAATATGCTAGGAGCCTGGCGTGACTCGAACTCAGGACCTCCTGCTCTGATACCATGTGAAACAACCATTGTACTCTAAAAGCTTAAGCTGTTGGAGAACGGTGTTTAAATATTTTTATATTTAACACTCCCCCTCACGTCTGGGTTCGAGACTTTTTTAGTTGGCCTGGCCCATGACGTGGGCTTAAATTAAATGGAAAGAAATTAATATGCTAGGAGCCTGGCGTGACTCGAACTCAGGATTTCCTGCTCTGATACCATGAAAGAAGGGCTAAAGTGCATACCCTCTTGAGTTGTATTAATGAATATATATATAGTACAAGAGATTGTCTTACTGTAGTAACAATGACTATTATAGTAAGAGTATAACTAGGATTACATACTATATAATCCTACTCTATAGAGCAATGATACTAATCTATATAAGATAATAATACACAATATCCTAATATATCGTAACATCCTAATATAGATAAAATATATCGTAACAGATACCAATAAGAAAGATGCTTTCGATGCAATCTGAAGCTCTAGTATAGTGGACTCATATTGTTGGGGCAATAAAGGTAACTCGATCGACAATCTTATCTTTTCAACATGCCGATCGACATTGGCTTCATGATTTATTTGCGGATGTTCAAATTCTAACTCGATCAAATCTAGAACTTCTTTGCAATCAGATAGTGTAGATGGAATTGAATCAGAATTGTCAACTCCCAATTGTGCTATAGCTAGCATACTCTGTTAAGCAATTAGGTTCTCCAGATGAACTTTTAGTTGGTTCAATTGACACTCATATCTGACCTTACCCATACATTGAAAATCATCTTCAGCAATTTGAGCACCATCTTCAATATCTTTTAACCTTTTTTCTTCTTCCTCAAAAATTATTCTGTTAAAATCAAGATGAAATATTTGCCTTTGTCTATACCACCATCTCAAGTCATCTAGTATTTTCATTCGAATATCATTTTTTTTTTCTCTCAAAATATGATCTCTGATTGTACAACCATTCATCAAACTCAACTCTTGTCTTCTCGCATATTTCTTTTAAAGACTCAATATGAATTCCTTTTCTTCCACTATACTTTTGAAAAACATTTTGAAAACATTCTTTTGCATCCATAATGTTTTTTTTCTTTTTTTTTGTTTTACACACTCTCCATTTCTTTTTCTCCACTCACTTTTCTTTCAACTTTTTTTTTTTTTCACTCCACTCTTTTTTATAATTCTTTTTCTCACCTTTATTTTTTACAATTTTCACCTTGTTTTCATTCACAAGCCCAAAACTTTGACAAAATAGAACCGTCAGATAGCCTAAGCTTTGATTCCAGATCTGATGTAGGACAACATATAGGGTCTTACGGTTTTAGAGGGCTCCAAAATATAATTTATGATTTTGAAAGCTTTTTGAAAATTTGAATGTTTTTGAAATACTTTTTTAGAAATTTTCGTAATTAAAAAAAAGAAAATTAAAGCAAATAGAAAAATTCAAAAAATAGAAAAGATAATAATCTGGACTCACTCTAGGGCGACGCATTTGCGAAGGAAAAAAAAGACATCATTGCCAAAGCATTTGTCTTCATTATAAATTCATAAATTCATAAATTCATATTCATCCCCATACAAAGAAGCCTTATGCCTTATTTATACTATTCTTAACCTTATCTATACATAAACAACTTAGAATTTAATCTTATCCTAAATGACCAAACTAAAACAACTTTATCTAGAGATTAACGAAAAAATAAAAATTTAAAAACAGATAAAAGCAAATAAGAGGATAGCTAAAAATCCTCATTTAACTAACGAAAAGATATACTCAACTAATCTAATCTAATTTAAAAAACGATAAATTAAATCGGTTTAATTCGATCAACATAGGTTCAAGCTTATTCTGCATCATTCTATGAATTACTTTCCATGTTTTGTTTATTGCCAAGAAATTTAGATTCTTGTGAAAATTCATTAGCAAAGTCTAATCTTCATTTCTTTTATTTTCAAACACATCTATTTAGAGGAACAAAGAATCTGCCTTTGATGATTTAATTGACTACGTCAAGTTTTTGAAACTACAGCTGAAGGTACGTGATTCTGCTGTCCTTAAGTGTCCAAGGCATTTTTGTGCTTAGTAAATCTTGTTATAATCACTTGAATGGCAGGTATTGAGTGAGAGCCGCTTAGATGGTGAAGAGACTGATCATCGCTTTGTTCATGTTGAGGTATCACTTGCTTTTTCGTACCTCTTTTTTAGTTGATTAAATTAAAAAAAATGTTGTCAATGCTTTCTTCAAAATTTTCTACTCGTGATAACTTATTGAATAGGCTGGCCCTATCATTTTTATTGTAGAATCATGGAATTTTGATTAATCTCGAGTAAGGTTGCAAATCACCTTTTATTATGATTAGTGAATGAGTGCTTTTCTAAATTTCAATGCATTCCTCAGATTTTTCCTCAGATTTGATGGGCATTACTAGATAAAGCTGGATAAGACATTACATGAAGAACCTTGAATAATAGGTGTAAGGCTTTCTTGTTGTTTAGGACTCTTTCTATACAGGTCGATGTCATTTGCTGTGCTTATGGCTTTACAGAAGGTGCCATGCCATGAAAGTGGCAAGGGATGGTGTTTTGTTACATTATACAAAATTAGCAGTTTTTTAATATGTTAAATAAATTCAAATAGACAGCTATAGAAAGAACTGAATGTCAAGTATGCTGAATAAATTCATGAAGACAACTCTAGAAAGAACTGAATGTTAAGTACTTAAACAACTAAGCCTAGTTAATTTTGTCACGGAAGTTAGAAATTTAGAATCTTACCCAGCATATGCCTTGGAGAGCACTAGAGTTATTCTCGCAGAGGTGTTTTCGGGGGTGGGGGTGTGGGGGGTGGTTAGACGCCTCACCCTTACACAGTTCATCACTACCCAACCAAACTAATGTCCAACAACAAGGGCAAGAAAATAAAATTATGCCATCGCTGTATCTACACTTCTCATATAAATCCAACCAAAAGACTAAGCTTGAATACCTTTCAACCTATTATAATCATTTTCACAAAGACATAACCACCAAAAAGTGAATAATAGCAATGAAAAGAGCTGGTCGGAGAAACTTGATCTGCTCCTCAAAGTGTCCTCTTTTACTTGACCTATATATTGAAATCTTCATTAGTAGGCAGGGTCATACTTGAGTGGTCATAGGTGTAGTTTTTTGTTATCTCTATATGAGTTAGATAATCTGTTAAAATTTCTGTTGATGGCTTCCACTAAACTATCCATGATGCAGGACATATTTTGAACTATAATATGATTTATTGCATGATCTTTCAACTAATCATCATCATGATAAATTTCCCATGCTTTGCTCATATGATTCATAATCTTTTTTTTTGTTCATTCGATTGCTCCTTGTAGGAGCAAGGAAAAGTTATCGCACTGAGCCCCGACTTCTATAATTTTGTTTTTCAAACTTTTTTGTATTCTATGATAGTAATACGTGATCTATTGCCTTTCTGTCATTGCTACACATTGAAGTATGCATCTTCTCCATTCCTTCTTAGTTCTGTCTTGATACGTTTCCCTTTCAGTAAAATTCTGTTTTGATTGGAAAGCCATAAAAAAACATTAATCATTAGCTGCGCAAACACTTTCCATAAGAATTTGGCCACAGCATGTCTTTCACATCCGTTAAAGATATACTTATACTGTCTTAGTGGTATATTTCTTACTACCTATTCGCTACTCCTTTTTGTCAATGCCTTGATTTAGTTCTTCCTTTAACAATTAACATCATCTTCAGCACTTTAAAGTTCCCTCTCAACTCTTATCTTCTATCCTTGCATTCATCGTGAGTCTCCACAAATGTAACTTTATCACCCACCAAATCATTCAGGTTCGAAATTTAGTGGTTTGGGTGCTCTCACCTGTGGAAGAAGGTAAGGGGCTGTTTGGTATATCGAAAAAGTATGGAAAAAAAAATTTCGAGAAAAAAGTTTTCCATGAAATGAGAACACAAGTTTTCCATGTAATTTGGAAAAAGCTTTCCAAAAAAGCTAGTATTCCTTGGAACCAAACGAGCAAAAACTGAAAAAGAAGTTTTCTACTGAAAACATTTTTCTTGAAAAACTTTTTTTTATACTTTTCTGAGGAATCAAGTTGCTCCTAAATTTTTGTACCCACAAACCTGAATTGATAGAAGCTTGTTTCATGACCCTTTTTGTGACGCCCCGCTTCCCAGAGGGGTCATCCTAGAATTTCTCTAGCTCTAGACTCGCCCAACTCTCTTAACCTCTTGGACCTAACCACGGCCTAAAATGCCATAGTCAAGTTAAGATATTTCGCTCTAATATACTTCATATATAGGACTATTCCAAATCCTGGGGGTGTCACATCACTGACATTCTCTTTAGGCTTAAGCGCATTTACAAGTACCAGGTGATGTGAGACTCGTCTCGCTAGCCTTAGCTGACCTTGTGGGAAGTCGCACTTCACCCCTAATAAGCTTCAGCCGGGGTGCCGCGCTCTTGCCCGCTGTATGATCTTGGCTCAACTAAAACTCATCCCAAACTTTCGGCTTGTGATTGCCTCTGATATCAACTGTGACGTCCCGCTTCCCAAGGGGGTCAACTATCCTAAAACTACTCTACCCCTAGGCTCGCTCAACCCTCTTAACCTCTTGGGCCTAGCTATTGCCCAAAATGCTATAACTGGGTTAAGAGGTTTGGTCCTGCTATACCTCATATATAGGATTATTCCATATTCTGGGGGTGTCACACTTTTGATATCTTTTGTGCCTGTGATTAAGGAAAGGTCTACTTGAGTGCTGAAATATGCATACTAAATCTTAGATGCCTAGGATGGTGTTTCAAGAGAGAATAGGATGTGGCATCTAAGGTTTCTATGTGGCAGAGAATATATCTTGATATACTCTAGGGTCTCCTTTATATAACACTCAGTTATATTTGTATCTGAAATCAAATTCCATTATATTAGGAATCCTTGATTGAGGAATCCTTGATAGGAATTTGTATAAGAAAGAATCAAACCCCCATATTATTAGGAATCCTTTGCTGGGGAAAATAATAAGAACAGTCTAGAGGTCGCCAAAAGTCTATATTCATCTCTTTATGTAAACCGAGTTATGTTTCATATCCGAAATCAAATTCCATTGTTTTGGGAATCCTTGATTAGGGAAATCCCTGATAGGAATTCGTATAATAAAAAAATCAAACCCCTATATTATTAGGAATCCTTCATTGGGGAAAATAATTATAAAAGTCTAGAGGTTGCCAAAAACTCTTATATTCACTCCTTATGTGGACGTATTTCCCCTACATCTCCCAACCAACTGTCATGCTTAAAGTCCCTCTGGCATTCCTTAACGAATTCCTAGTGCCTACTTTATAGATTTTGTTAGCACCTATAATACACCTCCAGAATTGGGAGTTTTCACTTGATTTAGAAGTTCTCTCTTTTTTTGAATTTGTGTCACAAGCTCTTTCTGTATAAGCTGATTGTTTGCTTCGAAATGTCAGTTTAAGTTTAGAGTTTGAAATGTGCTCTTAAATTTTTGGTTACAGAAGTTTTATTGGCTTCAAGATTTTCACCCTTGGTTGAGAAGTCTCTAAATTTTTCTAAGAGAAACCATATGAAAAATATAGAGTTGTAATGATCATAAAAAACTTGATTTTACAATAGCTGGTATGTGAAATATTATTGTTTATGCTACTTTAAATGGAGTATATGATCAACTTTATTAAATATAGCAAGATATGATGAATGAAATAGTACAAATGATTAGTCGATTAGTCAAATTTGGTCTCTATGTCCTAGTTTCGGCAATTTAGGCATGCATGCTCAAGCTGAGTTCTCTTTTAATTTATTTAACCCAAAAAAAAAGTTCTGGTCTCGGATTCGTTCAGGGCGATTGTTCATAAGAGCCAATTAGAATATAGGATGAATATTCAAACCTTGTAGGAAAACTTTTTTCCACTAGCAAAACAATTGCATGTATTTGCACGCTTACCTCTTCTAATAATATTTAGCAGGATGTGATTTGTGCAACATGTGCTGCTTGTTATGGTACCTAGACTACGAAGCCTGATTGGCTACCATGTTACCACTTGTCTGTCAGAAGCGGGCTATTAGTATTAATCAGTAAGCAACTTTCGGCCCAAACACATGTAAAACACATTGAAAGAAATTTCACCAGGATTTTCCTAGAAAGGGACCAAAGTTTAGATAACAATGTCTGGAACTTGCCTAAATTATCACCCATTTTGATTTGACTACTTGACTTTTAATAATTTTGATTTTGCTATCTAACCTTTTAATTCATTTGAATGAGCCATTTAATGACTCTTGCTGCTTCAAATTTTGATGCGTTGTTTATCTTTTCGGGCTTGATATAAATCCAATAAAATTAAACAAGTCCATTGTTTAATTCGTGTGACCATAGGTAAGCCATGAGCTTGTAAATACAAAATTTGGATGCGTTGGTTATCTTTCATGTAATTCCTGGGACTATAAATTAATTAAAGTAAGTGATTAGTCTAAATTTTGTCCTGGAAGAACTGTCAAATGGCTTAAATTAAACAAATTGGCAAGGCTGGGTACCAAAATCAAAACTTTAAAAGGTTGCATAGTCGGATCAAAAGGGACCATCTTTCAGGTACTGTACTTTTTAACCAAAAGGTTACTATTATTTTCTCTTATACTCTACTTAATAGTTTTATCGATATTTACATAGATATCTTTGTGTTGGCAAAGAGAACCTGCGAAATGCTAATCACCGGCTTCACGGCATGCTTTTGGCTTAAAGTGGAGAATAATTCTTCCTAAAACTCAGATTCTGATTCTTCCATGTCGGAGTGCAGTAAAACAGACCGGCTTATTAATGCAGTAAAACAAACCAGCTATAATAAGCCTGCAATTTAATTTGTTCTCCCCCTTTTCATGAACTCCAGTAAACTATGAACCGACATTGAAAGTTAAAAGAGTGTCAAGATTTGGCCTGTAAATTGTCTATTCAAAAATATTTTGGGACATTTTAATATCTCACATTCAATAGATCTCAATATGCTGAGCAGGGTTATTCTGTGTTGAAGTACTGTAATAAAGCAAACCAGCTACAGTAAGACAGCTATTTAATTTGATATCTCCCTTTTCATGGACTCCAGTAACTTTGATCTGACATTGAAAGTTAAAACACTGTCAAGATTCGGTCTGTAAATTATCTATTCAAAAAATAATTTTGGACATTTTAATATCTCACATTTAGTTGATCTCAATGTGCTGAGCAGGGTTATGGGCACTATTTGCTTCATCCGCAGATGTCTGCAGAGCCGCTTGGAGAAATGATGGGCCAACTGATGGAGTCCAACATGCAGGTTGCGAATCAGCTGCTCGAGAGCAAAGGAATCGCTTTGTTCCCCTTGGAATTCGCATATACCCTTCTCCGGAGCAGTTGAAACTACTTGGTGACTAATAATTGATATTTTTGAATATTCATGTTAATATGATCTATGAGGTCTTCACTCTTTGCAGCACTATAGTTGGATCTCAGTTGGGAGTTGAATTGCTAGGCTTACAACTCTTTTGTTCCTTGTATTTTTGCTGAATGATTCCCTGTGGAGGCAATTTTGAAATATTCTGGGAGCAACTGCTGGGCGCGGTACTCCTTGCCCTGGAAATATTCTGGGGCAATTTTTCTACAAAATATACAATGATAGCCACACTGGC

>Aco027966; *Ac*bHLH102
[truncated: 99,194 more chars]
